# Activation of Cyanate Anions by Phosphine Radical Cations Enables Formal Hydrocarbamoylation of Alkenes

**DOI:** 10.1002/anie.202524782

**Published:** 2025-12-03

**Authors:** Petra Vojáčková, Armido Studer

**Affiliations:** ^1^ Organisch‐Chemisches Institut Universität Münster Corrensstraße 40 48149 Münster Germany

**Keywords:** Hydrofunctionalization, Iminophosphoranes, Phosphoranyl radicals, Photocatalysis, Radical reactions

## Abstract

Catalytic methods that enable functionalization of alkenes with radical intermediates generated from common feedstock chemicals are valuable in synthetic chemistry. In this study, we disclose a photocatalytic formal hydrocarbamoylation strategy for preparation of *N*‐acyl iminophosphorane products from activated alkenes through isocyanate‐derived phosphoranyl radicals. Mechanistic investigations suggest generation of the phosphoranyl radical by addition of a cyanate anion to a phosphine radical cation and provide support for its reactivity through the isocyanate moiety. This redox‐neutral method enables hydrofunctionalization of diverse alkenylarene and electron‐deficient alkene substrates containing sensitive groups such as epoxide, unactivated alkene, amine, or electron‐rich and electron‐deficient heterocycles. The synthetic versatility of the *N*‐acyl iminophosphorane functionality is demonstrated through one‐step conversion to other valuable nitrogen‐containing functional groups.

## Graphical abstract

A photocatalytic formal hydrocarbamoylation reaction that employs a cyanate anion as the C1 source and provides *N*‐acyl iminophosphorane products from activated alkenes is described. Mechanistic investigations suggest generation of a phosphoranyl radical by addition of the cyanate anion to a phosphine radical cation, which enables the delivery of the carbamoyl group across alkenes.

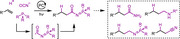

Radical hydrofunctionalizations of alkenes provide a powerful approach to the direct installation of diverse functional groups across carbon–carbon double bonds with high chemo‐ and regioselectivity.^[^ ^1^, ^2^ ^]^ Among these transformations, radical hydrocarbamoylation has emerged as a mild strategy for the synthesis of amides, offering a valuable alternative to traditional alkene carbamoylation protocols that often require the use of CO gas.^[^ ^3^ ^]^ Several approaches have been developed for generation of carbamoyl radicals, including hydrogen‐atom abstraction from formamides,^[^ ^4^, ^5^ ^]^ homolytic cleavage of various carbamoyl–X precursors,^[^ ^6^, ^7^ ^]^ and fragmentation of redox‐activated oxamic acids,^[^ ^8^ ^]^ 4‐carbamoyl‐1,4‐dihydropyridines,^[^ ^9^, ^10^ ^]^ or *N*‐hydroxyphthalimido esters.^[^ ^11^ ^]^ However, some of these methods suffer from poor selectivity,^[^ ^12^, ^13^ ^]^ and the preparation of most of the precursors requires multiple synthetic steps or the use of sensitive reagents. We envisioned that in situ generation of carbamoyl radicals from cyanate anions, where all three atoms of the anion are incorporated into the corresponding amide product, could provide an attractive strategy for radical hydrocarbamoylation.

Notably, the conjugate acid of cyanate serves in Nature as an electrophilic carbamoylating agent for post‐translational modification of amino groups of proteins.^[^ ^14^ ^]^ In a recent study, Hu and Radosevich reported activation of inorganic cyanate salts by bromophosphonium reagents to generate reactive isocyanatophosphonium ions that promoted electrophilic functionalization of carbon‐centered nucleophiles, such as electron‐rich (hetero)arenes and alkenes (Figure 1a).^[^ ^15^ ^]^ Inspired by this work, we hypothesized that phosphine radical cations could activate cyanates to form isocyanate‐derived phosphoranyl radicals capable of mediating radical hydrocarbamoylation of alkenes. Furthermore, we reasoned that substituents on the phosphorus atom might provide useful handles for tuning the reactivity of the carbamoyl radicals, offering an additional advantage over existing approaches.

**Figure 1:**
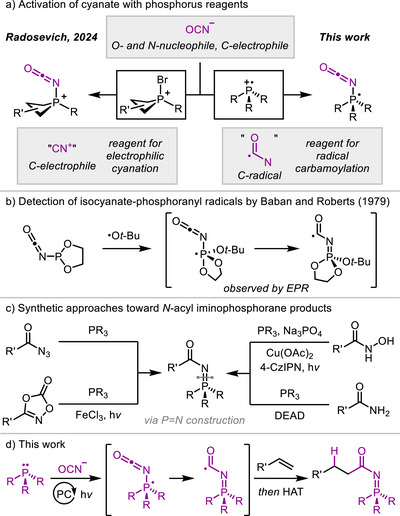
Activation of cyanate anions with phosphorus reagents and methods for preparation of *N*‐acyl iminophosphorane products. a) Cyanate as a versatile reagent. b) Reaction system studied by early electron paramagnetic resonance (EPR) studies.^[^ ^24^ ^]^ c) Known methods for preparation of *N*‐acyl iminophosphoranes proceeding through P═N bond formation. d) The method developed in this work enabling preparation of *N*‐acyl iminophosphorane products through C─C bond formation. 4‐CzIPN = 2,4,5,6‐tetrakis(9*H*‐carbazol‐9‐yl) isophthalonitrile. DEAD = diethyl azodicarboxylate. HAT = hydrogen‐atom transfer. PC = photoredox catalyst.

Activation of nucleophiles by phosphine radical cations to form phosphoranyl radicals that undergo α‐ or β‐scission fragmentation has emerged as a versatile platform for accessing diverse carbon‐ and heteroatom‐centered radicals.^[^ ^16^, ^17^, ^18^ ^]^ This strategy has enabled the activation of strong N─H bonds^[^ ^19^, ^20^ ^]^ and the deoxygenation or desulfurization of various nucleophilic functional groups.^[^ ^16^, ^17^, ^18^ ^]^ In addition, our group and others have demonstrated that phosphine radical cations can activate water as the nucleophile to realize formal hydrogenation of closed‐shell π‐systems through a hydrogen‐atom‐transfer‐type (HAT‐type) β‐fragmentation.^[^ ^21^, ^22^, ^23^ ^]^ Importantly, in contrast to these known α‐ and β‐scission fragmentation pathways, the proposed reactivity of phosphoranyl radicals through the π‐system of a substituent on the phosphorus atom represents an unexplored mechanistic possibility.

Supporting evidence for our proposal comes from electron paramagnetic resonance (EPR) studies by Baban and Roberts, which suggested delocalization of the unpaired electron of isocyanate‐derived phosphoranyl radicals on the isocyanate moiety (Figure 1b).^[^ ^24^ ^]^ However, this structural feature of isocyanate‐derived phosphoranyl radicals has never been exploited in synthesis. We envisioned that isocyanate‐derived phosphoranyl radicals could be generated under mild conditions by photoinduced single‐electron‐transfer (SET) oxidation of phosphines followed by nucleophilic addition of cyanate anions and enable applications in formal hydrocarbamoylations of alkenes to yield diverse *N*‐acyl iminophosphorane products. The ambident nucleophilicity of the cyanate anion posed a potential challenge for the proposed strategy; however, we reasoned that delocalization of the unpaired electron on the isocyanate substituent might favor formation of the *N*‐bound adduct.^[^ ^15^, ^24^ ^]^

The *N*‐acyl iminophosphorane moiety is a valuable functional group that can serve as a directing group for metal coordination,^[^ ^25^, ^26^ ^]^ a ligand,^[^ ^27^ ^]^ a hydrogen‐bond acceptor, or a Brønsted base,^[^ ^28^ ^]^ and can be readily converted into other useful functionalities such as amide, acyl imine, nitrile, or guanidine groups.^[^ ^29^, ^30^ ^]^ However, a vast majority of known methods for the synthesis of *N*‐acyl iminophosphorane products target the formation of the P═N bond and use highly reactive substrates or reagents (Figure 1c).^[^ ^31^, ^32^, ^33^, ^34^, ^35^, ^36^, ^37^ ^]^ We envisioned that a mild method that allows preparation of these products from feedstock chemicals through construction of the C─C linkage between the functional group and the substrate would represent an important synthetic advance.^[^ ^38^ ^]^ Here, we report a new catalytic method for preparation of *N*‐acyl iminophosphorane products by hydrofunctionalization of activated alkenes proceeding through phosphoranyl radical intermediates derived from phosphines and cyanate salts (Figure 1d).

We initiated our investigations using styrene **1** as the model alkene substrate (Table 1). Based on our previous work, we selected tris(4‐methoxyphenyl)phosphine and iridium photoredox catalyst **PC1** as a promising system for generation of phosphine radical cations under blue‐light irradiation.^[^ ^22^ ^]^ Tetrabutylammonium cyanate salt was identified as a suitable source of the cyanate anion because of the high solubility of this salt in organic solvents. Trifluoroethanol was chosen as a mildly acidic proton source. We found that *N*‐acyl iminophosphorane **2** formed under these conditions in 94% yield (Table 1, entry 1). Control experiments revealed that light and **PC1** were essential for the observed reactivity (entries 2 and 3). Running the reaction in the absence of trifluoroethanol additive or under air was found to be detrimental to the yield of **2** (entries 4 and 5). Although iridium‐based photoredox catalysts were found superior to other catalysts tested in our system (see Table S3), the reaction promoted by the organic photoredox catalyst 2,4,5,6‐tetrakis(9*H*‐carbazol‐9‐yl) isophthalonitrile (4‐CzIPN, **PC2**) furnished **2** in high yield (entry 6). Sodium or potassium cyanate salts could be used as less expensive alternative sources of the cyanate anion, but solubilizing additives such as tetrabutylammonium chloride or 18‐crown‐6 were required for obtaining **2** in comparable yields (entries 7–10).

**Table 1:** Optimization of reaction conditions.^a)^

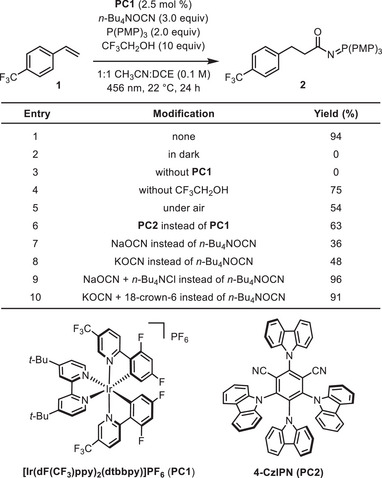

^a)^ Reactions were performed using 0.10 mmol of **1**. Yields were determined by proton nuclear magnetic resonance (^1^H NMR) of crude reaction mixtures using 1,3,5‐trimethoxybenzene as an internal standard. PMP = 4‐methoxyphenyl. DCE = 1,2‐dichloroethane. Equiv = equivalent. [Ir(dF(CF_3_)ppy)_2_(dtbbpy)]PF_6_ = [iridium(III)‐bis‐(2‐(2,4‐difluorophenyl)‐3‐trifluoromethylpyridine)‐4,4′‐di‐*tert*‐butyl‐2,2′‐bipyridine]hexafluorophosphate.

Next, we evaluated the substrate scope of the developed hydro(phosphaneylidene)carbamoylation reaction under the optimized conditions (Scheme 1). The phosphine reaction component was varied first, and we found that electron‐rich aryl and alkyl phosphines provided the corresponding products in higher yields than phosphines bearing electron‐withdrawing substituents (**2**–**8**). Styrene derivatives with various substituents on the arene ring successfully underwent the carbamoylation reaction, but substrates containing electron‐donating groups displayed reduced reactivity (**2**, **9**–**19**). Importantly, many potentially sensitive functional groups such as ester, halogens, boronic acid pinacol ester, or carbamate were well‐tolerated by the method. Since unactivated alkenes were not suitable substrates for the developed hydrofunctionalization reaction, a substrate containing both styrene and 3‐allyloxy groups underwent a chemoselective functionalization at the activated alkene to provide **19**. Furthermore, pyridyl derivatives proved to be suitable substrates for the developed protocol (**20**, **21**). Functionalization of styrenes bearing α‐substituents was achieved in high yield (**22**, **23**), but substituents at the β‐position lowered the efficiency of the process (**24**–**26**).

**Scheme 1:**
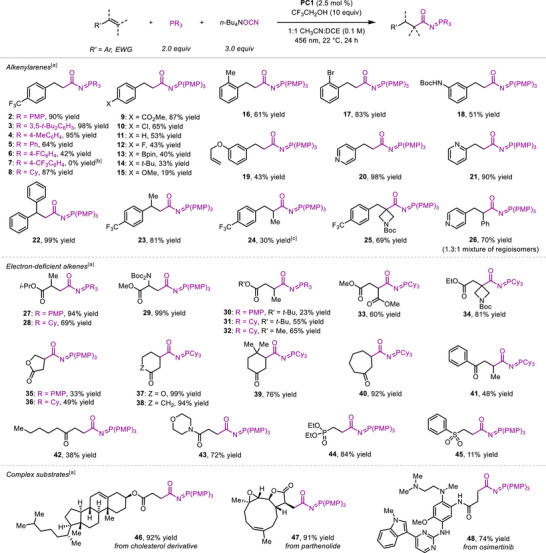
Product scope of the hydro(phosphaneylidene)carbamoylation reaction. ^[a]^Reactions were performed using 0.30 mmol of an alkene substrate. Reported yields represent yields of pure isolated products. ^[b]^Reaction was performed using 0.10 mmol of **1**. The yield was determined by ^1^H NMR analysis of the crude reaction mixture. ^[c]^Reaction was conducted with 5.0 equiv of trifluoroethanol. EWG = electron‐withdrawing group. Bpin = pinacol boronic ester group.

In addition to alkenylarenes, α,β‐unsaturated esters with α‐substituents successfully underwent the hydrofunctionalization reaction to provide 1,4‐dicarbonyl products **27** and **29**. In contrast, more hindered β‐substituted substrates converted in diminished yields under the standard conditions (**30**, **35**). However, the substitution of tris(4‐methoxyphenyl)phosphine for tricyclohexylphosphine delivered the corresponding *N*‐acyl iminophosphorane products in good yields (**31**–**34**), highlighting the ability of the substituents on phosphorus to tune the reactivity of the corresponding carbamoyl radical. The modified conditions were also successfully applied to lactones and cyclic ketones (**36**–**40**). In contrast, acyclic α,β‐unsaturated ketones provided the corresponding products in slightly lower yields (**41**, **42**). Extension of the method to other electron‐deficient alkenes illustrated that an acryl amide derivative and a vinylphosphonate were suitable substrates to provide **43** and **44** in high yields, in contrast to phenyl vinyl sulfone that yielded **45** with poor efficiency. Application of the developed method to late‐stage functionalization of complex substrates revealed that even very sensitive functional groups such as epoxide, unactivated alkene, basic amine, or electron‐rich and electron‐deficient heterocycles were well‐tolerated and furnished *N*‐acyl iminophosphorane products **46**, **47**, and **48** in excellent yields.

To demonstrate the synthetic utility of the *N*‐acyl iminophosphorane products, **2** was converted into the corresponding amide **49** and carboxylic acid **50** under acidic conditions (Scheme 2). The application of the method developed by Kundu and co‐workers allowed us to convert the *N*‐acyl iminophosphorane group of our products to provide nitrile derivatives **51**, **52**, and **53**.^[^ ^25^ ^]^ We found that a selective reduction of the carbonyl group of the *N*‐acyl iminophosphorane moiety can be performed using diisobutylaluminum hydride (DIBAL) to produce iminophosphorane products **54** and **55**. Iminophosphorane **54** was further functionalized to the corresponding amine product **56** through the aza‐Wittig reaction with (*R*)‐citronellal followed by reduction of the corresponding imine group with sodium borohydride in methanol.^[^ ^39^ ^]^

**Scheme 2:**
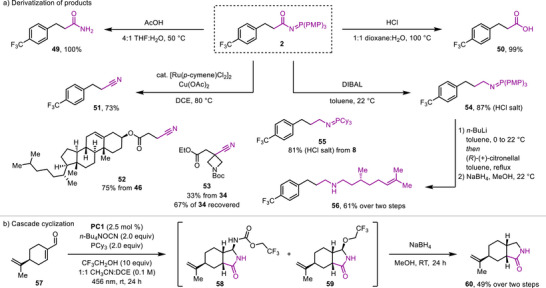
a) Derivatization of *N*‐acyl iminophosphorane products. b) Cascade reaction. THF = tetrahydrofuran. DIBAL = diisobutylaluminum hydride. Boc = *tert*‐butyloxycarbonyl.

During the substrate scope explorations, we observed that carbamoylation of (*S*)‐perillaldehyde (**57**) did not provide the expected product containing the *N*‐acyl iminophosphorane group, but instead products **58** and **59** were isolated. We reasoned that these products resulted from an intramolecular aza‐Wittig reaction between the generated *N*‐acyl iminophosphorane group and the aldehyde of the substrate forming the corresponding acyl imine intermediate that was trapped by cyanate and/or trifluoroethanol present in the reaction mixture. Addition of sodium borohydride and methanol to the crude mixture of products allowed us to isolate **60** as a single diastereomer in 49% yield over the two‐step cascade process.

Although our observations during reaction optimization and substrate scope studies were consistent with the initially proposed mechanism, other mechanistic scenarios leading to the observed products could be envisioned (Figure 2a). The putative phosphoranyl radical **62** could instead arise from addition of cyanate radical to neutral phosphine. Additionally, the C─C bond formation might occur through pentacoordinate P(V) intermediate **65** generated by either addition of the phosphoranyl radical **61** to the alkene or by nucleophilic addition of a cyanate anion to phosphonium salt **64**.^[^ ^19^, ^40^, ^41^, ^42^, ^43^, ^44^, ^45^ ^]^ Although this type of rearrangement of a P(V) intermediate is not precedented in the literature to our knowledge, we hypothesized that involvement of the π‐system of the isocyanate ligand might enable such reactivity.^[^ ^40^, ^44^ ^]^

**Figure 2:**
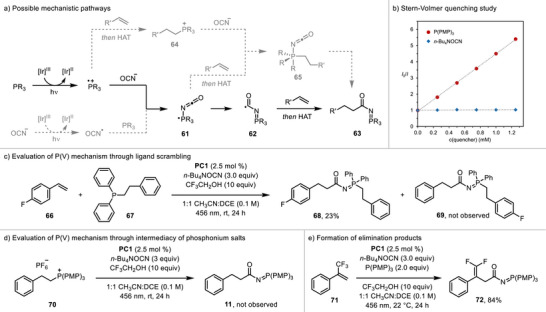
Mechanistic studies. a) Depiction of mechanistic possibilities. b) Stern–Volmer quenching study. c) Evaluation of the mechanism proceeding through pentacoordinate P(V) intermediates. d) Evaluation of the mechanism involving phosphonium intermediates.

To discriminate between the two possible pathways for generation of the proposed phosphoranyl radicals **61** and **62**, we performed Stern‐Volmer quenching studies using tris(4‐methoxyphenyl)phosphine and tetrabutylammonium cyanate salt (Figure 2b). We found that the phosphine was more effective in quenching the excited photoredox catalyst **PC1** than the cyanate salt. This result is inconsistent with the mechanistic scenario initiated by formation of the cyanate radical and supports the pathway proceeding via the phosphine radical cation intermediate.

To investigate the proposed mechanistic pathway including P(V) intermediate **65**, we performed the developed catalytic reaction using diphenyl(2‐phenylethyl)phosphine (**67**) and 4‐fluorostyrene substrate (**66**) (Figure 2c). This combination of substrates would afford P(V) intermediate containing 2‐phenylethyl and 2‐(4‐fluorophenyl)ethyl substituents, and thus formation of a mixture of products **68** and **69** would be expected if the P(V) mechanism was operative. However, the desired product **68** formed in 23% yield while product **69** was not detected under these conditions (see Supporting Informtion page S35 for details). The alternative pathway for formation of the P(V) intermediate by addition of a cyanate anion to the corresponding phosphonium salt was probed by subjecting independently prepared phosphonium hexafluorophosphate salt **70** to the catalytic conditions in the absence of a phosphine and an alkene substrate (Figure 2d). *N*‐Acyl iminophosphorane **11** was not observed in the reaction mixture, suggesting that phosphonium intermediate **64** was not a catalytically relevant intermediate.

These results provide strong evidence that the developed hydro(phosphaneylidene)carbamoylation reaction proceeds through activation of the cyanate anion by a phosphine radical cation intermediate to generate the phosphoranyl radical that adds to alkene substrates, thus forming the C─C bond and a new C‐centered radical. The following transfer of hydrogen atom likely proceeds by SET reduction of the radical to the corresponding carbanion intermediate followed by protonation. This proposal is consistent with the observed substrate effects and formation of **72** as the only product in the carbamoylation reaction of **71**, presumably resulting from elimination of a fluoride anion (Figure 2e, see the Supporting Information for an additional example).^[^ ^46^ ^]^

In summary, we have introduced a new strategy for preparation of *N*‐acyl iminophosphorane products by exploiting a previously unexplored reactivity of phosphoranyl radicals derived from phosphines and cyanate salts. Mechanistic investigations suggest generation of the phosphoranyl radical by addition of a cyanate anion to a phosphine radical cation and provide support for its reactivity through the isocyanate substituent. The developed approach enables construction of the C─C bond between the *N*‐acyl iminophosphorane group and substrates, thus providing a complementary strategy to the known methods. We anticipate that the mild conditions of the formal hydrocarbamoylation reaction and the versatility of the *N*‐acyl iminophosphorane group will enable applications in various synthetic contexts.

## Supporting Information

The authors have cited additional references within the Supporting Information.^[^ ^47^, ^48^, ^49^, ^50^, ^51^, ^52^, ^53^ ^]^

## Conflict of Interests

The authors declare no conflict of interest.

## Supporting information



Supporting Information

## Data Availability

The data that support the findings of this study are available in the supplementary material of this article.
