# Supplementary material for: Activation of Cyanate Anions by Phosphine Radical Cations Enables Formal Hydrocarbamoylation of Alkenes
Source: Angew Chem Int Ed Engl. 2025 Dec 3;65(4):e24782. doi: 10.1002/anie.202524782 (PMC12828471; doi:10.1002/anie.202524782)
Supplement: Supplementary file 1 — Supporting Information [file ANIE-65-e24782-s001.pdf]

Supporting Information

Activation of Cyanate Anions by Phosphine Radical Cations Enables Formal Hydrocarbamoylation of Alkenes

Petra Vojáčková and Armido Studer*

Organisch-Chemisches Institut, Universität Münster
Correnssstraße 40, 48149 Münster, Germany

*Corresponding author. Email: studer@uni-muenster.de

Table of contents

General information S3
Procedures for hydro(phosphaneylidene)carbamoylation reactions S4
Optimization of reaction conditions S22
Cascade reactions and derivatizations of products S26
Synthesis and characterization of substrates S32
Mechanistic experiments S34
  Stern-Volmer quenching study S34
  Evaluation of mechanistic pathways proceeding through P(V) intermediates S35
  Evaluation of mechanistic pathways proceeding through phosphonium intermediates S37
NMR spectra S39
References S172

References 1–46 are cited in the main manuscript.[1–46]

General information

Procedures

All reactions were performed in screw-cap Schlenk tubes or round-bottom flasks fitted with rubber septa under a positive pressure of argon, unless noted otherwise. Photochemical reactions were performed in screw-cap Schlenk tubes using the EvoluChem PhotoRedOx Box equipped with a 456 nm Kessil lamp. Reactions were monitored by thin-layer chromatography (TLC) using TLC Silica gel 60 F_{254} glass plates impregnated with a fluorescent indicator (Merck). TLC plates were visualized by exposure to ultraviolet light ($\lambda = 254$ nm) and/or by submersion in aqueous ceric ammonium molybdate or aqueous potassium permanganate solutions followed by brief heating. All solutions were concentrated by rotary evaporation, unless noted otherwise. Flash column chromatography (FCC) was performed using Silica gel 60 (40–63 μm, Merck).

Materials

All reagents purchased from commercial suppliers (Sigma-Aldrich, Alfa Aesar, TCI, Acros Organics, Fisher Scientific, ABCR, Fluorochem, Fluka, and BLDPharm) were used without further purification unless noted otherwise. Commercial substrates containing stabilizers were purified by filtration through a plug of basic alumina (2 cm) prior to use. Extraction and chromatography solvents were purchased in technical purity and were distilled prior to use. Reaction solvents (*N*,*N*-dimethylformamide (DMF), methyl *tert*-butyl ether (MTBE), benzene, toluene, 1,4-dioxane, acetonitrile, 1,2-dichloroethane (DCE)) were purchased from Acros Organics (extra dry grade). Other reaction solvents were dried and distilled under argon atmosphere prior to use (dichloromethane (DCM) was dried over P_2O_5, diethyl ether was dried over Na/K alloy, tetrahydrofuran (THF) was dried over Na/K alloy). 2,2,2-Trifluoroethanol (TFE) was dried and stored over activated 3Å molecular sieves (MS).

Instrumentation

Nuclear magnetic resonance (NMR) spectra were recorded using Bruker Avance II 300 (300 MHz), Bruker NEO 400 (400 MHz), Agilent DD2 500 (500 MHz), or Agilent DD2 600 (600 MHz) NMR spectrometers. Proton chemical shifts are expressed in parts per million (ppm, δ scale) and are referenced to residual protium in the NMR solvent ($CHCl_3$: δ 7.26 ppm). Carbon chemical shifts are expressed in parts per million (ppm, δ scale) and are referenced to the carbon resonance of the NMR solvent ($CDCl_3$: δ 77.2 ppm). $^{13}C\{^1H\}$ NMR spectra are abbreviated as ^{13}C, $^{13}C\{^1H,^{19}F\}$ NMR spectra are abbreviated as $^{13}C\{^{19}F\}$. Fluorine, boron, and phosphorus chemical shifts are expressed in parts per million (ppm, δ scale) and are referenced according to the proton resonance of TMS as the primary reference for the unified chemical shift scale. Data are represented as follows: chemical shift, multiplicity (s = singlet, d = doublet, t = triplet, q = quartet, quint = quintet, sept = septet, m = multiplet and/or multiple resonances, app = apparent, br = broad), coupling constants (*J*) in Hertz, integration. Fourier transform infrared (FTIR) spectra were obtained using JASCO-FT-IR-6800 spectrometer. Data are represented as follows: frequency of absorption (cm^{-1}), intensity of absorption (vs = very strong, s = strong, m = medium, w = weak, br = broad). Mass spectra were recorded on an Exploris 120 Electrospray Orbitrap spectrometer (electrospray ionization (ESI)), a Thermo-Fisher Scientific LTQ Orbitrap LTQ XL spectrometer (ESI), or a Thermo Fisher Scientific Trace 1310 GC Exactive Orbitrap GC-MS system (electron ionization (EI)) at the Mass Spectrometry Department of the Organisch-Chemisches Institut at the University of Münster.

Procedures for hydro(phosphaneylidene)carbamoylation reactions

Product scope

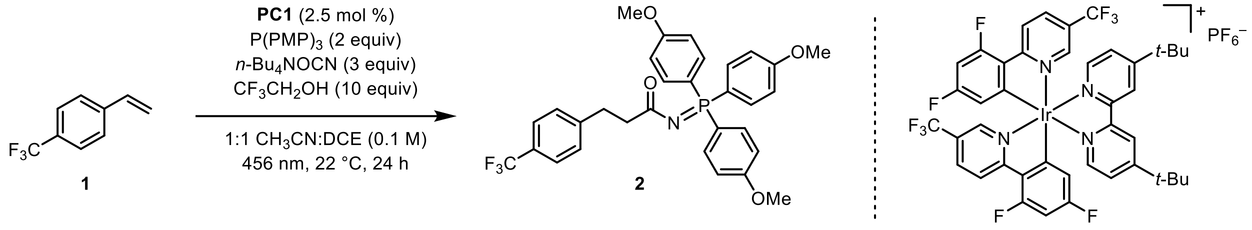

[Ir(dF(CF$_3$)ppy)$_2$(dtbbpy)]PF$_6$ (**PC1**)

<u>3-(4-(Trifluoromethyl)phenyl)-*N*-(tris(4-methoxyphenyl)-λ^5-phosphaneylidene)propenamide (**2**)</u>

A mixture of tetrabutylammonium cyanate (0.26 g, 0.90 mmol, 3.0 equiv) and iridium catalyst **PC1** (8.4 mg, 7.5 µmol, 0.025 equiv) in acetonitrile (1.5 mL) was added to a mixture of tris(4-methoxyphenyl)phosphine (0.21 g, 0.60 mmol, 2.0 equiv) and 4-(trifluoromethyl)styrene (**1**, 44 µL, 0.30 mmol, 1 equiv) in 1,2-dichloroethane (1.5 mL) in a Schlenk tube at ambient temperature under argon atmosphere. 2,2,2-Trifluoroethanol (0.22 mL, 3.0 mmol, 10 equiv) was added via syringe at ambient temperature. The reaction tube was placed in a photoreactor equipped with a 456 nm Kessil lamp. The reaction mixture was stirred at ambient temperature (22 °C) for 24 h. The reaction mixture was concentrated under reduced pressure, and the crude product mixture was purified by FCC (40–100% ethyl acetate in pentane) to provide **2** as a yellow oil (154 mg, 90%). ^{1}H NMR (500 MHz, CDCl$_3$) δ 7.59–7.52 (m, 6H), 7.49–7.45 (m, 2H), 7.39–7.34 (m, 2H), 6.94–6.89 (m, 6H), 3.83 (s, 9H), 3.09 (t, *J* = 7.5 Hz, 2H), 2.81 (td, *J* = 7.5, 1.5 Hz, 2H). ^{13}C{^{19}F} NMR (126 MHz, CDCl$_3$) δ 183.4 (d, *J* = 9.8 Hz), 162.7 (d, *J* = 2.8 Hz), 147.0, 135.0 (d, *J* = 11.5 Hz), 129.2, 127.9, 125.1, 124.7, 119.8 (d, *J* = 105.9 Hz), 114.3 (d, *J* = 13.3 Hz), 55.5, 41.4 (d, *J* = 19.2 Hz), 32.8 (d, *J* = 1.8 Hz). ^{19}F{^{1}H} NMR (470 MHz, CDCl$_3$) δ −62.14. ^{31}P NMR (202 MHz, CDCl$_3$) δ 18.52 (m). FTIR (neat), cm^{-1}: 1593 (s), 1500 (m), 1322 (m), 1252 (s), 1104 (vs), 1019 (s), 825 (s), 800 (s), 528 (s). HRMS (ESI): Calcd for [$C_{31}H_{29}NO_4PF_3Na$]$^+$: 590.16785, found: 590.16598.

<u>3-(4-(Trifluoromethyl)phenyl)-*N*-(tris(3,5-di-*tert*-butylphenyl)-λ^5-phosphaneylidene)propenamide (**3**)</u>

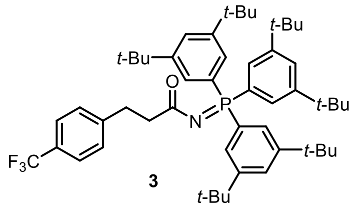

N-Acyl iminophosphorane **3** was prepared according to an analogous procedure as described for the preparation of **2** on page S4 using tris(3,5-di-*tert*-butylphenyl)phosphine (0.36 g, 0.60 mmol, 2.0 equiv). The product was purified by FCC (20% ethyl acetate in pentane) and isolated as a colorless semi-solid (239 mg, 98%). ^{1}H NMR (500 MHz, CDCl$_3$) δ 7.57 (app q, *J* = 1.7 Hz, 3H), 7.50 (d, *J* = 1.8 Hz, 3H), 7.47 (d, *J* = 1.8 Hz, 3H), 7.45 (d, *J* = 8.1 Hz, 2H), 7.37 (d, *J* = 8.0 Hz, 2H), 3.16 (t, *J* = 7.7 Hz, 2H), 2.83 (td, *J* = 7.8, 1.3 Hz, 2H), 1.24 (s, 54H). ^{13}C{^{19}F} NMR (126 MHz, CDCl$_3$) δ 183.1 (d, *J* = 10.3 Hz), 151.0 (d, *J* = 11.9 Hz), 147.4, 129.0, 128.1 (d, *J* = 97.7 Hz), 127.9, 127.6 (d, *J* = 10.5 Hz), 126.1 (d, *J* = 2.9 Hz), 125.2, 124.7, 41.7 (d, *J* = 19.4 Hz), 35.2 (d, *J* = 0.8 Hz), 33.1 (d, *J* = 2.2 Hz), 31.5. ^{19}F{^{1}H} NMR (283 MHz, CDCl$_3$) δ −62.21. ^{31}P NMR (202 MHz, CDCl$_3$) δ 22.99 (m). FTIR (neat), cm^{-1}: 2960 (m), 1579 (m), 1323 (s), 1123 (s), 1067 (m), 707 (m), 598 (m), 487 (m). HRMS (ESI): Calcd for [$C_{52}H_{71}NOPF_3Na$]$^+$: 836.51176, found: 836.51160.

__*N*-(Tri-*p*-tolyl-λ^5-phosphaneylidene)-3-(4-(trifluoromethyl)phenyl)propenamide (**4**)__

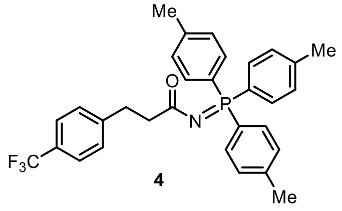

4

N-Acyl iminophosphorane **4** was prepared according to an analogous procedure as described for the preparation of **2** on page S4 using tris(4-methylphenyl)phosphine (0.18 g, 0.60 mmol, 2.0 equiv). The product was purified by FCC (30–70% ethyl acetate in pentane) and isolated as a colorless oil (148 mg, 95%). ^{1}H NMR (500 MHz, $CDCl_3$) δ 7.57–7.50 (m, 6H), 7.46 (d, *J* = 8.1 Hz, 2H), 7.36 (d, *J* = 8.0 Hz, 2H), 7.24–7.19 (m, 6H), 3.10 (t, *J* = 7.5 Hz, 2H), 2.81 (td, *J* = 7.5, 1.3 Hz, 2H), 2.39 (s, 9H). ^{13}C{^{19}F} NMR (126 MHz, $CDCl_3$) δ 183.5 (d, *J* = 10.1 Hz), 147.0, 142.7 (d, *J* = 2.9 Hz), 133.2 (d, *J* = 10.2 Hz), 129.5 (d, *J* = 12.6 Hz), 129.1, 127.9, 125.3 (d, *J* = 101.3 Hz), 125.1, 124.7, 41.4 (d, *J* = 19.4 Hz), 32.7 (d, *J* = 2.2 Hz), 21.7 (d, *J* = 1.4 Hz). ^{19}F{^{1}H} NMR (283 MHz, $CDCl_3$) δ −62.17. ^{31}P NMR (202 MHz, $CDCl_3$) δ 19.37 (m). FTIR (neat), cm^{-1}: 1585 (s), 1322 (vs), 1105 (vs), 1065 (m), 805 (s), 656 (s), 503 (s). HRMS (ESI): Calcd for $[C_{31}H_{29}NOPF_3Na]^+$: 542.18311, found: 542.18365.

__3-(4-(Trifluoromethyl)phenyl)-*N*-(triphenyl-λ^5-phosphaneylidene)propenamide (**5**)__

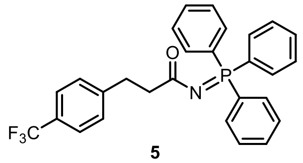

5

N-Acyl iminophosphorane **5** was prepared according to an analogous procedure as described for the preparation of **2** on page S4 using triphenylphosphine (0.16 g, 0.60 mmol, 2.0 equiv). The product was purified by FCC (40–80% ethyl acetate in pentane) and isolated as a colorless oil (92 mg, 64%). ^{1}H NMR (400 MHz, $CDCl_3$) δ 7.72–7.61 (m, 6H), 7.58–7.50 (m, 3H), 7.50–7.39 (m, 8H), 7.36 (d, *J* = 8.0 Hz, 2H), 3.11 (t, *J* = 7.5 Hz, 2H), 2.88–2.79 (m, 2H). ^{13}C{^{19}F} NMR (126 MHz, $CDCl_3$) δ 183.7 (d, *J* = 10.2 Hz), 146.9, 133.2 (d, *J* = 9.9 Hz), 132.3 (d, *J* = 2.9 Hz), 129.1, 128.8 (d, *J* = 12.2 Hz), 128.3 (d, *J* = 98.9 Hz), 128.0, 125.2, 124.7, 41.3 (d, *J* = 19.4 Hz), 32.7 (d, *J* = 2.2 Hz). ^{19}F{^{1}H} NMR (283 MHz, $CDCl_3$) δ −62.17. ^{31}P NMR (202 MHz, $CDCl_3$) δ 19.53 (m). FTIR (neat), cm^{-1}: 1584 (s), 1437 (w), 1321 (s), 1105 (s), 1065 (m), 831 (s), 718 (m), 690 (s), 529 (s). HRMS (ESI): Calcd for $[C_{28}H_{23}NOPF_3Na]^+$: 500.13616, found: 500.13651.

__3-(4-(Trifluoromethyl)phenyl)-*N*-(tris(4-fluorophenyl)-λ^5-phosphaneylidene)propenamide (**6**)__

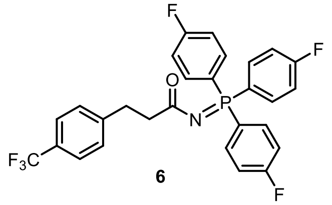

6

N-Acyl iminophosphorane **6** was prepared according to an analogous procedure as described for the preparation of **2** on page S4 using tris(4-fluorophenyl)phosphine (0.19 g, 0.60 mmol, 2.0 equiv). The product was purified by FCC (20–60% ethyl acetate in pentane) and isolated as a colorless oil (67 mg, 42%). ^{1}H NMR (500 MHz, $CDCl_3$) δ 7.67–7.59 (m, 6H), 7.47 (d, *J* = 8.1 Hz, 2H), 7.34 (d, *J* = 8.0 Hz, 2H), 7.17–7.10 (m, 6H), 3.08 (t, *J* = 7.5 Hz, 2H), 2.81 (td, *J* = 7.5, 1.5 Hz, 2H). ^{13}C NMR (126 MHz, $CDCl_3$) δ 183.9 (d, *J* = 10.2 Hz), 165.5 (dd, *J* = 255.3, 3.3 Hz), 146.6 (q, *J* = 1.5 Hz), 135.6 (dd, *J* = 11.5, 8.9 Hz), 129.1, 128.1 (q, *J* = 32.2 Hz), 125.2 (q, *J* = 3.8 Hz), 124.6 (q, *J* = 271.6 Hz), 123.8 (dd, *J* = 103.2, 3.5 Hz), 116.5 (dd, *J* = 21.6, 13.6 Hz), 41.2 (d, *J* = 19.5 Hz), 32.6 (d, *J* = 2.2 Hz). ^{19}F{^{1}H} NMR (283 MHz, $CDCl_3$) δ −62.21 (3F), −105.33 (d, *J* = 1.5 Hz, 3F). ^{31}P NMR (202 MHz, $CDCl_3$) δ 17.51 (m). FTIR (neat), cm^{-1}: 1589 (s), 1496 (m), 1322 (s), 1229 (s), 1158 (m), 1106 (vs), 1065 (m), 826 (vs), 518 (s), 468 (s). HRMS (ESI): Calcd for $[C_{28}H_{20}NOPF_6Na]^+$: 554.10789, found: 554.10861.

N-(Tricyclohexyl-λ^5-phosphaneylidene)-3-(4-(trifluoromethyl)phenyl)propenamide (**8**)

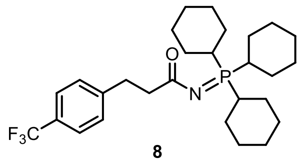

8

N-Acyl iminophosphorane **8** was prepared according to an analogous procedure as described for the preparation of **2** on page S4 using tricyclohexylphosphine (0.17 g, 0.60 mmol, 2.0 equiv). The product was purified by FCC (40–100% ethyl acetate in pentane) and isolated as an off-white amorphous solid (130 mg, 87%). ^{1}H NMR (500 MHz, $CDCl_3$) δ 7.47 (d, J = 8.1 Hz, 2H), 7.36 (d, J = 8.1 Hz, 2H), 3.00 (t, J = 7.5 Hz, 2H), 2.64 (t, J = 7.4 Hz, 2H), 2.27–2.12 (m, 3H), 1.88–1.73 (m, 12H), 1.73–1.63 (m, 3H), 1.48–1.35 (m, 6H), 1.30–1.17 (m, 9H). ^{13}C{^{19}F} NMR (126 MHz, $CDCl_3$) δ 183.0 (d, J = 10.8 Hz), 147.2, 129.0, 127.8, 125.0, 124.7, 41.0 (d, J = 19.2 Hz), 34.1 (d, J = 54.3 Hz), 32.8, 27.2(1) (d, J = 2.6 Hz), 27.1(5) (d, J = 10.8 Hz), 26.1 (d, J = 1.3 Hz). ^{19}F{^{1}H} NMR (283 MHz, $CDCl_3$) δ −62.23. ^{31}P NMR (202 MHz, $CDCl_3$) δ 38.88 (m). FTIR (neat), cm^{-1}: 2927 (s), 2850 (m), 1568 (s), 1446 (w), 1357 (m), 1321 (s), 1308 (s), 1155 (m), 1117 (s), 1065 (m), 824 (m). HRMS (ESI): Calcd for $[C_{28}H_{41}NOPF_3Na]^+$: 518.27701, found: 518.27661.

Methyl 4-(3-oxo-3-((tris(4-methoxyphenyl)-λ^5-phosphaneylidene)amino)propyl)benzoate (**9**)

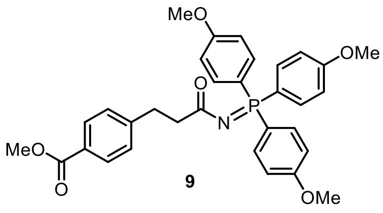

9

N-Acyl iminophosphorane **9** was prepared according to an analogous procedure as described for the preparation of **2** on page S4 using methyl 4-vinylbenzoate (49 mg, 0.30 mmol, 1 equiv). The product was purified by FCC (40–100% ethyl acetate in pentane) and isolated as a yellow amorphous solid (145 mg, 87%). ^{1}H NMR (400 MHz, $CDCl_3$) δ 7.90 (d, J = 8.3 Hz, 2H), 7.61–7.49 (m, 6H), 7.32 (d, J = 8.3 Hz, 2H), 6.97–6.86 (m, 6H), 3.89 (s, 3H), 3.83 (s, 9H), 3.09 (t, J = 7.6 Hz, 2H), 2.80 (td, J = 7.6, 1.5 Hz, 2H). ^{13}C NMR (101 MHz, $CDCl_3$) δ 183.5 (d, J = 10.2 Hz), 167.5, 162.7 (d, J = 2.9 Hz), 148.6, 135.0 (d, J = 11.3 Hz), 129.7, 128.9, 127.6, 119.9 (d, J = 105.8 Hz), 114.3 (d, J = 13.3 Hz), 55.5, 52.1, 41.5 (d, J = 19.3 Hz), 33.0 (d, J = 2.3 Hz). ^{31}P NMR (162 MHz, $CDCl_3$) δ 18.41 (m). FTIR (neat), cm^{-1}: 1712 (m), 1593 (m), 1564 (m), 1500 (m), 1359 (m), 1285 (s), 1252 (s), 1177 (m), 1109 (vs), 1021 (s), 827 (s), 800 (m), 527 (s). HRMS (ESI): Calcd for $[C_{32}H_{32}NO_6PNa]^+$: 580.18595, found: 580.18811.

3-(4-Chlorophenyl)-N-(tris(4-methoxyphenyl)-λ^5-phosphaneylidene)propenamide (**10**)

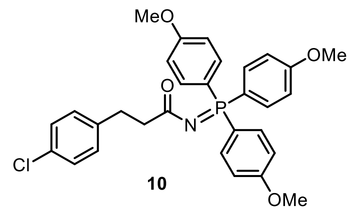

10

N-Acyl iminophosphorane **10** was prepared according to an analogous procedure as described for the preparation of **2** on page S4 using 4-chlorostyrene (38 μL, 0.30 mmol, 1 equiv). The product was purified by FCC (40–100% ethyl acetate in pentane) and isolated as a colorless oil (103 mg, 65%). ^{1}H NMR (400 MHz, $CDCl_3$) δ 7.61–7.50 (m, 6H), 7.21–7.15 (m, 4H), 6.97–6.88 (m, 6H), 3.83 (s, 9H), 3.00 (t, J = 7.5 Hz, 2H), 2.77 (td, J = 7.5, 1.5 Hz, 2H). ^{13}C NMR (101 MHz, $CDCl_3$) δ 183.6 (d, J = 10.0 Hz), 162.7 (d, J = 2.9 Hz), 141.3, 135.0 (d, J = 11.2 Hz), 131.2, 130.2, 128.3, 119.8 (d, J = 105.8 Hz), 114.3 (d, J = 13.2 Hz), 55.5, 41.7 (d, J = 19.1 Hz), 32.3 (d, J = 2.1 Hz). ^{31}P NMR (162 MHz, $CDCl_3$) δ 18.67 (m). FTIR (neat), cm^{-1}: 1593 (s), 1499 (m), 1254 (s), 1177 (m), 1108 (vs), 1023 (s), 824 (s), 800 (s), 527 (s). HRMS (ESI): Calcd for $[C_{30}H_{29}NO_4PClNa]^+$: 556.14149, found: 556.14327.

3-Phenyl-*N*-(tris(4-methoxyphenyl)-λ^5-phosphaneylidene)propenamide (**11**)

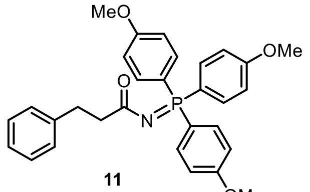

11

N-Acyl iminophosphorane **11** was prepared according to an analogous procedure as described for the preparation of **2** on page S4 using styrene (35 μL, 0.30 mmol, 1 equiv). The product was purified by FCC (40–100% ethyl acetate in pentane) and isolated as a white amorphous solid (80 mg, 53%). ^{1}H NMR (600 MHz, $CDCl_3$) δ 7.62–7.56 (m, 6H), 7.28–7.21 (m, 4H; overlapping with the solvent signal), 7.18–7.14 (m, 1H), 6.96–6.92 (m, 6H), 3.84 (s, 9H), 3.05 (dd, *J* = 8.9, 6.9 Hz, 2H), 2.80 (ddd, *J* = 9.0, 7.1, 1.5 Hz, 2H). ^{13}C NMR (151 MHz, $CDCl_3$) δ 184.0–183.6 (m), 162.8 (d, *J* = 2.9 Hz), 142.7, 135.1 (d, *J* = 11.3 Hz), 128.8, 128.3, 125.7, 119.5 (d, *J* = 106.7 Hz), 114.4 (d, *J* = 13.3 Hz), 55.6, 41.9 (d, *J* = 18.5 Hz), 32.9 (d, *J* = 2.0 Hz). ^{31}P NMR (242 MHz, $CDCl_3$) δ 18.94 (m). FTIR (neat), cm^{-1}: 1584 (s), 1497 (m), 1253 (s), 1225 (m), 1178 (s), 1104 (s), 1016 (s), 826 (m), 801 (m), 527 (s). HRMS (ESI): Calcd for $[C_{30}H_{30}NO_4PNa]^+$: 522.18047, found: 522.18247.

3-(4-Fluorophenyl)-*N*-(tris(4-methoxyphenyl)-λ^5-phosphaneylidene)propenamide (**12**)

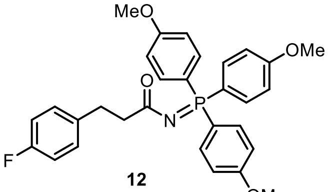

12

N-Acyl iminophosphorane **12** was prepared according to an analogous procedure as described for the preparation of **2** on page S4 using 4-fluorostyrene (36 μL, 0.30 mmol, 1 equiv). The product was purified by FCC (40–100% ethyl acetate in pentane) and isolated as a yellow oil (67 mg, 43%). ^{1}H NMR (400 MHz, $CDCl_3$) δ 7.65–7.52 (m, 6H), 7.24–7.15 (m, 2H), 6.99–6.85 (m, 8H), 3.83 (s, 9H), 3.01 (t, *J* = 7.7 Hz, 2H), 2.76 (td, *J* = 7.7, 1.6 Hz, 2H). ^{13}C{^{19}F} NMR (126 MHz, $CDCl_3$) δ 183.8 (d, *J* = 10.2 Hz), 162.6 (d, *J* = 2.9 Hz), 161.3, 138.4, 135.0 (d, *J* = 11.4 Hz), 130.1, 119.9 (d, *J* = 105.8 Hz), 114.9, 114.3 (d, *J* = 13.3 Hz), 55.5, 42.1 (d, *J* = 19.2 Hz), 32.2 (d, *J* = 2.1 Hz). ^{19}F{^{1}H} NMR (376 MHz, $CDCl_3$) δ −118.59. ^{31}P{^{1}H} NMR (162 MHz, $CDCl_3$) δ 18.36. FTIR (neat), cm^{-1}: 1593 (s), 1500 (s), 1252 (s), 1178 (m), 1108 (vs), 1022 (s), 824 (s), 801 (s), 528 (s). HRMS (ESI): Calcd for $[C_{30}H_{29}NO_4PFNa]^+$: 540.17104, found: 540.17277.

3-(4-(4,4,5,5-Tetramethyl-1,3,2-dioxaborolan-2-yl)phenyl)-*N*-(tris(4-methoxyphenyl)-λ^5-phosphaneylidene)propenamide (**13**)

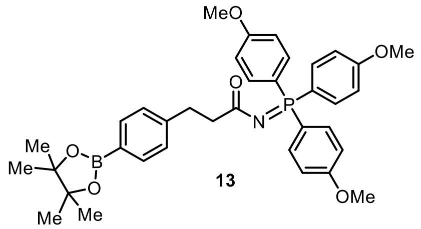

13

N-Acyl iminophosphorane **13** was prepared according to an analogous procedure as described for the preparation of **2** on page S4 using (4-vinylphenyl)boronic acid pinacol ester (69 mg, 0.30 mmol, 1 equiv). The product was purified by FCC (40–100% ethyl acetate in pentane) and isolated as a white amorphous solid (75 mg, 40%). ^{1}H NMR (400 MHz, $CDCl_3$) δ 7.70 (d, *J* = 7.9 Hz, 2H), 7.63–7.53 (m, 6H), 7.27 (d, *J* = 7.9 Hz, 2H), 6.96–6.89 (m, 6H), 3.83 (s, 9H), 3.05 (dd, *J* = 8.8, 6.8 Hz, 2H), 2.78 (ddd, *J* = 9.0, 7.1, 1.6 Hz, 2H), 1.33 (s, 12H). ^{13}C NMR (101 MHz, $CDCl_3$) δ 183.9 (d, *J* = 9.9 Hz), 162.7 (d, *J* = 2.9 Hz), 146.3, 135.0 (d, *J* = 11.3 Hz), 134.9, 133.8 (app d, *J* = 12.0 Hz), 128.3, 119.8 (d, *J* = 105.8 Hz), 114.4 (d, *J* = 13.3 Hz), 83.7, 55.5, 41.8 (d, *J* = 18.7 Hz), 33.2 (d, *J* = 2.0 Hz), 25.0. ^{11}B NMR (128 MHz, $CDCl_3$) δ 31.49. ^{31}P NMR (162 MHz, $CDCl_3$) δ 18.57 (m). FTIR (neat), cm^{-1}: 1594 (s), 1500 (m), 1356 (s), 1252 (s), 1110 (vs), 1022 (m), 825 (s), 801 (m), 658 (m), 529 (s). HRMS (ESI): Calcd for $[C_{36}H_{41}NO_6PBH]^+$: 626.28436, found: 626.28458.

3-(4-(*Tert*-butyl)phenyl)-*N*-(tris(4-methoxyphenyl)-λ^5-phosphaneylidene)propenamide (**14**)

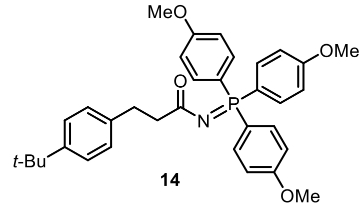

14

N-Acyl iminophosphorane **14** was prepared according to an analogous procedure as described for the preparation of **2** on page S4 using 4-*tert*-butylstyrene (55 µL, 0.30 mmol, 1 equiv). The product was purified by FCC (40–100% ethyl acetate in pentane) and isolated as a yellow oil (54 mg, 33%). ^{1}H NMR (400 MHz, $CDCl_3$) δ 7.66–7.57 (m, 6H), 7.27 (d, *J* = 8.0 Hz, 2H; overlapping with the solvent signal), 7.20 (d, *J* = 8.3 Hz, 2H), 6.97–6.90 (m, 6H), 3.83 (s, 9H), 3.08–2.98 (m, 2H), 2.78 (td, *J* = 7.5, 1.3 Hz, 2H), 1.31 (s, 9H). ^{13}C NMR (101 MHz, $CDCl_3$) δ 184.4 (d, *J* = 10.4 Hz), 162.6 (d, *J* = 2.9 Hz), 148.3, 139.8, 135.1 (d, *J* = 11.3 Hz), 128.4, 125.2, 120.2 (d, *J* = 105.6 Hz), 114.3 (d, *J* = 13.3 Hz), 55.5, 42.1 (d, *J* = 18.9 Hz), 34.5, 32.6, 31.7. ^{31}P NMR (162 MHz, $CDCl_3$) δ 18.13 (m). FTIR (neat), cm^{-1}: 1593 (s), 1500 (m), 1252 (s), 1177 (m), 1108 (vs), 1023 (s), 825 (s), 801 (s), 529 (s). HRMS (ESI): Calcd for $[C_{34}H_{38}NO_4PNa]^+$: 578.24307, found: 578.24518.

3-(4-Methoxyphenyl)-*N*-(tris(4-methoxyphenyl)-λ^5-phosphaneylidene)propenamide (**15**)

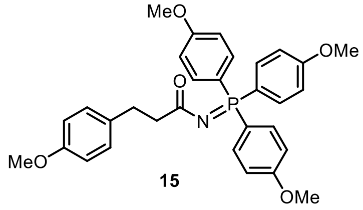

15

N-Acyl iminophosphorane **15** was prepared according to an analogous procedure as described for the preparation of **2** on page S4 using 4-methoxystyrene (40 µL, 0.30 mmol, 1 equiv). The product was purified by FCC (40–100% ethyl acetate in pentane) and isolated as a white amorphous solid (31 mg, 19%). ^{1}H NMR (400 MHz, $CDCl_3$) δ 7.63–7.53 (m, 6H), 7.16 (d, *J* = 8.5 Hz, 2H), 6.98–6.89 (m, 6H), 6.79 (d, *J* = 8.6 Hz, 2H), 3.84 (s, 9H), 3.78 (s, 3H), 2.98 (t, *J* = 7.7 Hz, 2H), 2.76 (t, *J* = 7.4 Hz, 2H). ^{13}C NMR (101 MHz, $CDCl_3$) δ 183.7 (d, *J* = 11.4 Hz), 162.8 (d, *J* = 2.9 Hz), 157.8, 135.1 (d, *J* = 11.4 Hz), 134.7, 129.7, 119.2 (d, *J* = 105.7 Hz), 114.4 (d, *J* = 13.4 Hz), 113.8, 55.6, 55.4, 42.0 (d, *J* = 17.9 Hz), 32.0 (d, *J* = 1.9 Hz). ^{31}P NMR (162 MHz, $CDCl_3$) δ 19.44 (m). FTIR (neat), cm^{-1}: 1579 (s), 1501 (m), 1363 (m), 1253 (s), 1107 (s), 1022 (s), 826 (s), 801 (s), 538 (s). HRMS (ESI): Calcd for $[C_{31}H_{32}NO_5PNa]^+$: 552.19103, found: 552.19285.

3-(*o*-Tolyl)-*N*-(tris(4-methoxyphenyl)-λ^5-phosphaneylidene)propenamide (**16**)

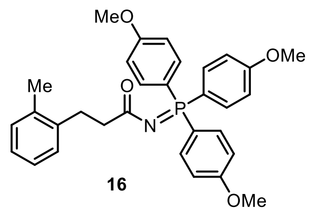

16

N-Acyl iminophosphorane **16** was prepared according to an analogous procedure as described for the preparation of **2** on page S4 using 2-methylstyrene (39 µL, 0.30 mmol, 1 equiv). The product was purified by FCC (40–100% ethyl acetate in pentane) and isolated as a white amorphous solid (94 mg, 61%). ^{1}H NMR (400 MHz, $CDCl_3$) δ 7.69–7.57 (m, 6H), 7.27–7.22 (m, 1H; overlapping with the solvent signal), 7.15–7.05 (m, 3H), 6.99–6.90 (m, 6H), 3.83 (s, 9H), 3.04 (dd, *J* = 9.4, 6.6 Hz, 2H), 2.76 (td, *J* = 7.8, 1.6 Hz, 2H), 2.35 (s, 3H). ^{13}C NMR (101 MHz, $CDCl_3$) δ 184.3 (d, *J* = 10.2 Hz), 162.6 (d, *J* = 2.9 Hz), 141.0, 136.4, 135.0 (d, *J* = 11.2 Hz), 130.1, 128.8, 125.9, 125.7, 120.1 (d, *J* = 105.7 Hz), 114.3 (d, *J* = 13.2 Hz), 55.5, 40.8 (d, *J* = 19.1 Hz), 30.3 (d, *J* = 2.2 Hz), 19.6. ^{31}P NMR (162 MHz, $CDCl_3$) δ 18.01 (m). FTIR (neat), cm^{-1}: 1585 (s), 1497 (m), 1360 (w), 1254 (s), 1178 (m), 1104 (s), 1019 (s), 827 (m), 801 (m), 534 (m). HRMS (ESI): Calcd for $[C_{31}H_{32}NO_4PNa]^+$: 536.19612, found: 536.19804.

3-(2-Bromophenyl)-*N*-(tris(4-methoxyphenyl)-λ^5-phosphaneylidene)propenamide (**17**)

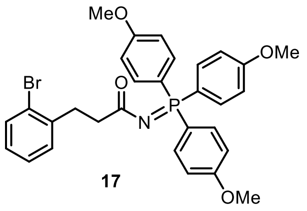

17

N-Acyl iminophosphorane **17** was prepared according to an analogous procedure as described for the preparation of **2** on page S4 using 2-bromostyrene (38 μL, 0.30 mmol, 1 equiv). The product was purified by FCC (40–100% ethyl acetate in pentane) and isolated as a colorless oil (144 mg, 83%). ^{1}H NMR (400 MHz, $CDCl_3$) δ 7.66–7.56 (m, 6H), 7.51 (dd, *J* = 8.1, 1.5 Hz, 1H), 7.31 (dd, *J* = 7.6, 1.8 Hz, 1H), 7.15 (app td, *J* = 7.4, 1.3 Hz, 1H), 7.01 (app td, *J* = 7.7, 1.8 Hz, 1H), 6.97–6.89 (m, 6H), 3.83 (s, 9H), 3.17 (t, *J* = 7.8 Hz, 2H), 2.85–2.73 (m, 2H). ^{13}C NMR (101 MHz, $CDCl_3$) δ 183.6 (d, *J* = 10.2 Hz), 162.6 (d, *J* = 3.0 Hz), 142.1, 135.0 (d, *J* = 11.2 Hz), 132.7, 130.6, 127.3(3), 127.3(1), 124.9, 120.1 (d, *J* = 105.8 Hz), 114.3 (d, *J* = 13.2 Hz), 55.5, 40.3 (d, *J* = 19.3 Hz), 33.3 (d, *J* = 2.3 Hz). ^{31}P NMR (162 MHz, $CDCl_3$) δ 17.94 (m). FTIR (neat), cm^{-1}: 1592 (s), 1499 (m), 1250 (s), 1177 (m), 1107 (s), 1022 (s), 825 (m), 800 (m), 527 (s). HRMS (ESI): Calcd for $[C_{30}H_{29}NO_4PBrH]^+$: 578.10903 and 580.10749, found: 578.10835 and 580.10649.

Tert-butyl (3-(3-oxo-3-((tris(4-methoxyphenyl)-λ^5-phosphaneylidene)amino)propyl)phenyl)carbamate (**18**)

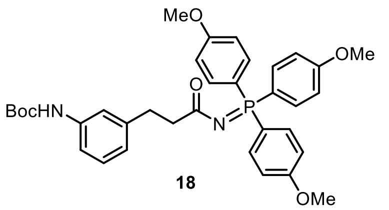

18

N-Acyl iminophosphorane **18** was prepared according to an analogous procedure as described for the preparation of **2** on page S4 using *tert*-butyl *N*-(3-ethenylphenyl)carbamate (66 mg, 0.30 mmol, 1 equiv). The product was purified by FCC (40–100% ethyl acetate in pentane) and isolated as a yellow amorphous solid (94 mg, 51%). ^{1}H NMR (400 MHz, $CDCl_3$) δ 7.63–7.53 (m, 6H), 7.30 (d, *J* = 8.2 Hz, 1H), 7.16 (app t, *J* = 7.8 Hz, 1H), 7.13–7.10 (m, 1H), 6.98–6.89 (7H), 6.42 (br s, 1H), 3.83 (s, 9H), 3.01 (t, *J* = 7.7 Hz, 2H), 2.77 (t, *J* = 7.4 Hz, 2H), 1.50 (s, 9H). ^{13}C NMR (101 MHz, $CDCl_3$) δ 183.9 (d, *J* = 10.2 Hz), 162.7 (d, *J* = 2.9 Hz), 152.9, 143.7, 138.3, 135.0 (d, *J* = 11.3 Hz), 128.9, 123.6, 119.8 (d, *J* = 105.5 Hz), 118.9, 116.0, 114.4 (d, *J* = 13.3 Hz), 80.4, 55.5, 41.8 (d, *J* = 18.9 Hz), 32.9 (d, *J* = 2.0 Hz), 28.5. ^{31}P NMR (162 MHz, $CDCl_3$) δ 18.46 (m). FTIR (neat), cm^{-1}: 1716 (m), 1594 (m), 1555 (m), 1500 (m), 1251 (s), 1109 (s), 1023 (m), 828 (m), 801 (m), 529 (s). HRMS (ESI): Calcd for $[C_{35}H_{39}N_2O_6PNa]^+$: 637.24379, found: 637.24357.

3-(3-(Allyloxy)phenyl)-*N*-(tris(4-methoxyphenyl)-λ^5-phosphaneylidene)propenamide (**19**)

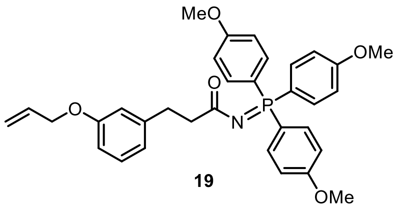

19

N-Acyl iminophosphorane **19** was prepared according to an analogous procedure as described for the preparation of **2** on page S4 using alkene **79** (48 mg, 0.30 mmol, 1 equiv). The product was purified by FCC (40–100% ethyl acetate in pentane) and isolated as a colorless oil (72 mg, 43%). ^{1}H NMR (400 MHz, $CDCl_3$) δ 7.65–7.53 (m, 6H), 7.15 (app t, *J* = 8.1 Hz, 1H), 6.98–6.90 (m, 6H), 6.88–6.81 (m, 2H), 6.76–6.69 (m, 1H), 6.03 (ddt, *J* = 17.3, 10.5, 5.3 Hz, 1H), 5.38 (app dq, *J* = 17.3, 1.7 Hz, 1H), 5.24 (app dq, *J* = 10.5, 1.5 Hz, 1H), 4.46 (app dt, *J* = 5.3, 1.6 Hz, 2H), 3.83 (s, 9H), 3.02 (dd, *J* = 8.8, 6.7 Hz, 2H), 2.79 (ddd, *J* = 9.0, 7.0, 1.3 Hz, 2H). ^{13}C NMR (101 MHz, $CDCl_3$) δ 183.6 (d, *J* = 9.8 Hz), 162.8 (d, *J* = 3.0 Hz), 158.7, 144.3, 135.1 (d, *J* = 11.4 Hz), 133.7, 129.2, 121.4, 119.4 (d, *J* = 105.1 Hz), 117.6, 115.0, 114.4 (d, *J* = 13.5 Hz), 112.3, 68.8, 55.5, 41.7 (d, *J* = 18.2 Hz), 33.0 (d, *J* = 1.7 Hz). ^{31}P NMR (162 MHz, $CDCl_3$) δ 19.17 (m). FTIR (neat), cm^{-1}: 1593 (s), 1499 (m), 1251 (s), 1178 (m), 1108 (s), 1022 (m), 826 (m), 801 (m), 528 (s). HRMS (ESI): Calcd for $[C_{33}H_{34}NO_5PNa]^+$: 578.20668, found: 578.20882.

3-(Pyridin-4-yl)-*N*-(tris(4-methoxyphenyl)-λ5-phosphaneylidene)propenamide (**20**)

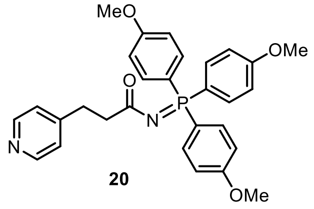

20

N-Acyl iminophosphorane **20** was prepared according to an analogous procedure as described for the preparation of **2** on page S4 using 4-vinylpyridine (32 μL, 0.30 mmol, 1 equiv). The product was purified by FCC (5–10% methanol in ethyl acetate) and isolated as a white amorphous solid (147 mg, 98%). ^{1}H NMR (400 MHz, $CDCl_3$) δ 8.41 (d, *J* = 6.0 Hz, 2H), 7.60–7.50 (m, 6H), 7.16 (d, *J* = 5.9 Hz, 2H), 6.95–6.88 (m, 6H), 3.82 (s, 9H), 3.02 (t, *J* = 7.5 Hz, 2H), 2.79 (t, *J* = 7.5 Hz, 2H). ^{13}C NMR (101 MHz, $CDCl_3$) δ 183.1 (d, *J* = 10.1 Hz), 162.7 (d, *J* = 2.9 Hz), 151.9, 149.6, 134.9 (d, *J* = 11.3 Hz), 124.3, 119.8 (d, *J* = 105.9 Hz), 114.3 (d, *J* = 13.3 Hz), 55.5, 40.5 (d, *J* = 19.6 Hz), 32.2 (d, *J* = 2.2 Hz). ^{31}P NMR (162 MHz, $CDCl_3$) δ 18.60 (m). FTIR (neat), cm^{-1}: 1592 (s), 1499 (m), 1251 (vs), 1178 (m), 1108 (vs), 1022 (s), 825 (s), 801 (s), 528 (s). HRMS (ESI): Calcd for $[C_{29}H_{29}N_2O_4PNa]^+$: 523.17572, found: 523.17479.

3-(Pyridin-2-yl)-*N*-(tris(4-methoxyphenyl)-λ5-phosphaneylidene)propenamide (**21**)

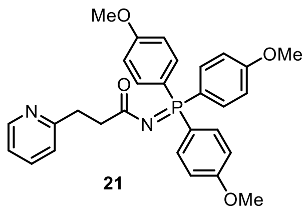

21

N-Acyl iminophosphorane **21** was prepared according to an analogous procedure as described for the preparation of **2** on page S4 using 2-vinylpyridine (32 μL, 0.30 mmol, 1 equiv). The product was purified by FCC (5–15% methanol in ethyl acetate) and isolated as a colorless oil (135 mg, 90%). ^{1}H NMR (400 MHz, $CDCl_3$) δ 8.52–8.45 (m, 1H), 7.66–7.53 (m, 6H), 7.48 (app td, *J* = 7.6, 1.9 Hz, 1H), 7.18 (d, *J* = 7.9 Hz, 1H), 7.03 (dd, *J* = 7.6, 4.8 Hz, 1H), 6.95–6.86 (m, 6H), 3.80 (s, 9H), 3.22 (t, *J* = 7.7 Hz, 2H), 2.89 (td, *J* = 7.7, 1.4 Hz, 2H). ^{13}C NMR (101 MHz, $CDCl_3$) δ 183.8 (d, *J* = 10.1 Hz), 162.5(3), 162.5(2) (d, *J* = 2.0 Hz), 149.1, 136.1, 134.9 (d, *J* = 11.3 Hz), 122.9, 120.8, 120.0 (d, *J* = 105.8 Hz), 114.2 (d, *J* = 13.3 Hz), 55.4, 40.1 (d, *J* = 19.3 Hz), 35.4 (d, *J* = 2.3 Hz). ^{31}P NMR (162 MHz, $CDCl_3$) δ 17.82 (m). FTIR (neat), cm^{-1}: 1591 (s), 1499 (m), 1250 (s), 1177 (m), 1107 (vs), 1021 (s), 825 (m), 800 (m), 528 (s). HRMS (ESI): Calcd for $[C_{29}H_{29}N_2O_4PNa]^+$: 523.17572, found: 523.17530.

3,3-Diphenyl-*N*-(tris(4-methoxyphenyl)-λ5-phosphaneylidene)propenamide (**22**)

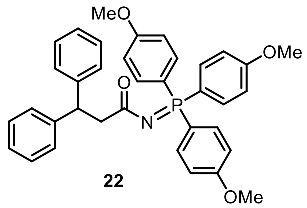

22

N-Acyl iminophosphorane **22** was prepared according to an analogous procedure as described for the preparation of **2** on page S4 using 1,1-diphenylethylene (53 μL, 0.30 mmol, 1 equiv). The product was purified by FCC (30–70% ethyl acetate in pentane) and isolated as a colorless oil (171 mg, 99%). ^{1}H NMR (400 MHz, $CDCl_3$) δ 7.48–7.40 (m, 6H), 7.39–7.33 (m, 4H), 7.29–7.21 (m, 4H), 7.20–7.12 (m, 2H), 6.90–6.82 (m, 6H), 4.78 (t, *J* = 8.1 Hz, 1H), 3.81 (s, 9H), 3.20 (dd, *J* = 8.2, 1.6 Hz, 2H). ^{13}C NMR (101 MHz, $CDCl_3$) δ 182.8 (d, *J* = 10.1 Hz), 162.5 (d, *J* = 2.9 Hz), 145.4, 134.9 (d, *J* = 11.3 Hz), 128.4, 128.4, 125.9, 119.8 (d, *J* = 105.7 Hz), 114.2 (d, *J* = 13.3 Hz), 55.5, 49.1 (d, *J* = 1.7 Hz), 46.9 (d, *J* = 19.0 Hz). ^{31}P NMR (162 MHz, $CDCl_3$) δ 18.26 (m). FTIR (neat), cm^{-1}: 1593 (s), 1499 (m), 1251 (s), 1178 (m), 1109 (vs), 1024 (s), 826 (s), 801 (s), 724 (m), 699 (m), 529 (s). HRMS (ESI): Calcd for $[C_{36}H_{34}NO_4PNa]^+$: 598.21177, found: 598.21258.

3-(4-(Trifluoromethyl)phenyl)-*N*-(tris(4-methoxyphenyl)-λ^5-phosphaneylidene)butanamide (**23**)

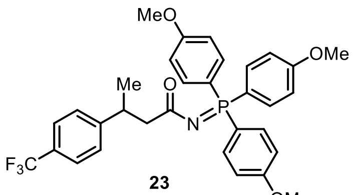

23

N-Acyl iminophosphorane **23** was prepared according to an analogous procedure as described for the preparation of **2** on page S4 using 1-(prop-1-en-2-yl)-4-(trifluoromethyl)benzene (56 mg, 0.30 mmol, 1 equiv). The product was purified by FCC (40–100% ethyl acetate in pentane) and isolated as a yellow oil (141 mg, 81%). ^{1}H NMR (500 MHz, $CDCl_3$) δ 7.54–7.38 (m, 10H), 6.92–6.84 (m, 6H), 3.81 (s, 9H), 3.58–3.46 (m, 1H), 2.84–2.71 (m, 2H), 1.29 (d, *J* = 7.0 Hz, 3H). ^{13}C{^{19}F} NMR (126 MHz, $CDCl_3$) δ 183.0 (d, *J* = 10.0 Hz), 162.6 (d, *J* = 2.9 Hz), 152.1, 134.9 (d, *J* = 11.2 Hz), 128.0, 127.9, 125.2, 124.7, 119.8 (d, *J* = 105.7 Hz), 114.3 (d, *J* = 13.2 Hz), 55.5, 48.6 (d, *J* = 18.7 Hz), 38.2 (d, *J* = 1.7 Hz), 22.8. ^{19}F{^{1}H} NMR (470 MHz, $CDCl_3$) δ −62.01. ^{31}P NMR (202 MHz, $CDCl_3$) δ 18.48 (m). FTIR (neat), cm^{-1}: 1594 (s), 1500 (m), 1324 (m), 1252 (s), 1107 (vs), 1024 (s), 826 (s), 801 (s), 528 (s). HRMS (ESI): Calcd for $[C_{32}H_{31}NO_4PF_3Na]^+$: 604.18350, found: 604.18558.

2-Methyl-3-(4-(trifluoromethyl)phenyl)-*N*-(tris(4-methoxyphenyl)-λ^5-phosphaneylidene)propenamide (**24**)

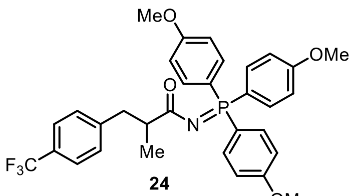

24

N-Acyl iminophosphorane **24** was prepared according to a modified procedure as described for the preparation of **2** on page S4 using (*E*)-1-(prop-1-en-1-yl)-4-(trifluoromethyl)benzene (56 mg, 0.30 mmol, 1 equiv) and TFE (0.11 mL, 1.5 mmol, 5.0 equiv). The product was purified by FCC (20–70% ethyl acetate in pentane) and isolated as a colorless oil (52 mg, 30%). ^{1}H NMR (500 MHz, $CDCl_3$) δ 7.56–7.48 (m, 6H), 7.41 (d, *J* = 8.0 Hz, 2H), 7.29 (d, *J* = 8.0 Hz, 2H), 6.94–6.88 (m, 6H), 3.83 (s, 9H), 3.14 (dd, *J* = 13.4, 8.2 Hz, 1H), 2.97–2.86 (m, 1H), 2.76 (dd, *J* = 13.4, 6.6 Hz, 1H), 1.22 (d, *J* = 6.9 Hz, 3H). ^{13}C{^{19}F} NMR (126 MHz, $CDCl_3$) δ 186.7 (d, *J* = 10.6 Hz), 162.6 (d, *J* = 2.9 Hz), 146.2, 135.0 (d, *J* = 11.2 Hz), 129.8, 127.8, 124.9, 124.7, 120.1 (d, *J* = 105.7 Hz), 114.3 (d, *J* = 13.3 Hz), 55.5, 46.1 (d, *J* = 18.9 Hz), 41.2, 18.9. ^{19}F{^{1}H} NMR (470 MHz, $CDCl_3$) δ −62.10. ^{31}P NMR (202 MHz, $CDCl_3$) δ 18.15 (m). FTIR (neat), cm^{-1}: 1593 (s), 1500 (m), 1323 (m), 1251 (s), 1106 (vs), 1019 (s), 825 (s), 801 (s), 533 (s). HRMS (ESI): Calcd for $[C_{32}H_{31}NO_4PF_3Na]^+$: 604.18350, found: 604.18578.

Tert-butyl 3-(4-(trifluoromethyl)benzyl)-3-((tris(4-methoxyphenyl)-λ^5-phosphaneylidene)carbamoyl)azetidine-1-carboxylate (**25**)

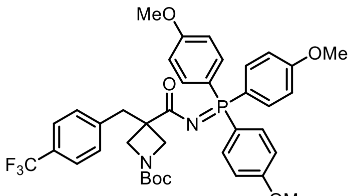

25

N-Acyl iminophosphorane **25** was prepared according to an analogous procedure as described for the preparation of **2** on page S4 using alkene **80** (94 mg, 0.30 mmol, 1 equiv). The product was purified by FCC (40–100% ethyl acetate in pentane) and isolated as a white amorphous solid (146 mg, 69%). ^{1}H NMR (500 MHz, $CDCl_3$) δ 7.56–7.48 (m, 6H), 7.29 (d, *J* = 7.9 Hz, 2H), 7.20 (d, *J* = 8.0 Hz, 2H), 6.96–6.89 (m, 6H), 4.38 (d, *J* = 8.4 Hz, 2H), 3.92–3.75 (m, 11H), 3.57–3.22 (m, 2H), 1.43 (s, 9H). ^{13}C{^{19}F} NMR (126 MHz, $CDCl_3$) δ 183.1 (d, *J* = 9.5 Hz), 162.8 (d, *J* = 2.9 Hz), 156.9, 143.2, 134.9 (d, *J* = 11.3 Hz), 129.8, 128.3, 124.9, 124.6, 119.5 (d, *J* = 106.5 Hz), 114.3 (d, *J* = 13.3 Hz), 79.3, 58.0, 55.5, 46.4, 43.0, 28.6. ^{19}F{^{1}H} NMR (470 MHz, $CDCl_3$) δ −62.30. ^{31}P NMR (202 MHz, $CDCl_3$) δ 18.71 (m). FTIR (neat), cm^{-1}: 1694 (m), 1593 (s), 1501 (m), 1324 (m), 1253 (s), 1107 (s), 1019 (m), 827 (m), 802 (m), 534 (s). HRMS (ESI): Calcd for $[C_{38}H_{40}N_2O_6PF_3Na]^+$: 731.24683, found: 731.24700.

2-Phenyl-3-(pyridin-4-yl)-*N*-(tris(4-methoxyphenyl)-λ^5-phosphaneylidene)propenamide (**maj-26**) and 3-phenyl-2-(pyridin-4-yl)-*N*-(tris(4-methoxyphenyl)-λ^5-phosphaneylidene)propenamide (**min-26**)

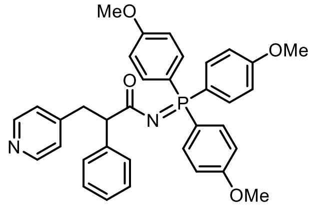

***maj*-26** *(major)*

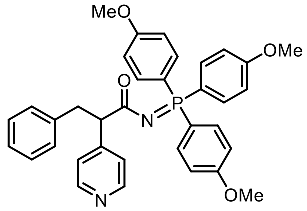

***min*-26** *(minor)*

N-Acyl iminophosphoranes ***maj*-26** and ***min*-26** were prepared according to an analogous procedure as described for the preparation of **2** on page S4 using 4-styrylpyridine (54 mg, 0.30 mmol, 1 equiv). The product formed as a 1.3:1 of regioisomers ***maj*-26** and ***min*-26**, respectively. The mixture was purified by FCC (5–7% methanol in ethyl acetate) and isolated as a colorless oil (120 mg, 70%). ***maj*-26** (major): ^{1}H NMR (400 MHz, $CDCl_3$) δ 8.35–8.29 (m, 2H), 7.48–7.42 (m, 8H), 7.29–7.24 (m, 2H), 7.22–7.17 (m, 1H), 7.09–7.05 (m, 2H), 6.89–6.85 (m, 6H), 4.03 (ddd, *J* = 9.1, 6.5, 2.6 Hz, 1H), 3.82 (s, 9H), 3.51 (dd, *J* = 13.8, 9.0 Hz, 1H), 3.00 (dd, *J* = 13.8, 6.5 Hz, 1H). ^{13}C NMR (101 MHz, $CDCl_3$) δ 182.9 (d, *J* = 10.4 Hz), 162.6 (d, *J* = 2.9 Hz), 150.6, 149.4, 142.2, 134.9 (d, *J* = 11.3 Hz), 128.3(4), 128.3(2), 126.5, 124.9, 119.8 (d, *J* = 105.9 Hz), 114.3 (d, *J* = 13.3 Hz), 57.5 (d, *J* = 19.3 Hz), 55.5, 40.0 (d, *J* = 2.3 Hz). ^{31}P NMR (162 MHz, $CDCl_3$) δ 18.24 (m). FTIR (neat), cm^{-1}: 1592 (s), 1499 (m), 1251 (s), 1178 (m), 1109 (s), 1023 (s), 825 (s), 801 (s), 528 (s). ***min*-26** (minor): ^{1}H NMR (400 MHz, $CDCl_3$) δ 8.46–8.42 (m, 2H), 7.50–7.43 (m, 6H), 7.36–7.32 (m, 2H), 7.17–7.08 (m, 5H), 6.92–6.86 (m, 6H), 4.02 (app td, *J* = 7.7, 2.7 Hz, 1H), 3.82 (s, 9H), 3.52 (dd, *J* = 13.8, 7.9 Hz, 1H), 3.05 (dd, *J* = 13.8, 7.5 Hz, 1H). ^{13}C NMR (101 MHz, $CDCl_3$) δ 182.0 (d, *J* = 10.3 Hz), 162.7 (d, *J* = 2.9 Hz), 151.6, 149.5, 140.5, 134.9 (d, *J* = 11.3 Hz), 129.3, 128.2, 125.9, 124.0, 119.7 (d, *J* = 106.1 Hz), 114.3 (d, *J* = 13.3 Hz), 58.2 (d, *J* = 19.3 Hz), 55.5, 40.1 (d, *J* = 1.9 Hz). ^{31}P NMR (162 MHz, $CDCl_3$) δ 18.34 (m). FTIR (neat), cm^{-1}: 1591 (s), 1499 (m), 1251 (vs), 1177 (s), 1108 (vs), 1023 (s), 826 (s), 800 (s), 528 (s). HRMS (ESI): Calcd for $[C_{35}H_{33}N_2O_4PNa]^+$: 599.20702, found: 599.20756.

Isopropyl 2-methyl-4-oxo-4-((tris(4-methoxyphenyl)-λ^5-phosphaneylidene)amino)butanoate (**27**)

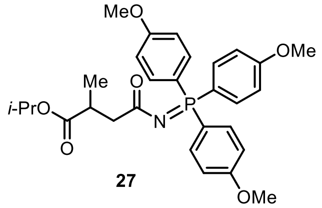

27

N-Acyl iminophosphorane **27** was prepared according to an analogous procedure as described for the preparation of **2** on page S4 using isopropyl methacrylate (43 µL, 0.30 mmol, 1 equiv). The product was purified by FCC (40–100% ethyl acetate in pentane) and isolated as a colorless oil (147 mg, 94%). ^{1}H NMR (400 MHz, $CDCl_3$) δ 7.69–7.58 (m, 6H), 6.98–6.88 (m, 6H), 4.95 (hept, *J* = 6.3 Hz, 1H), 3.82 (s, 9H), 3.03 (app h, *J* = 7.1 Hz, 1H), 2.86 (dd, *J* = 15.9, 7.4 Hz, 1H), 2.47 (ddd, *J* = 15.9, 6.9, 1.3 Hz, 1H), 1.19 (d, *J* = 7.1 Hz, 3H), 1.15 (d, *J* = 6.3 Hz, 3H), 1.12 (d, *J* = 6.3 Hz, 3H). ^{13}C NMR (101 MHz, $CDCl_3$) δ 182.6 (d, *J* = 10.0 Hz), 176.6, 162.6 (d, *J* = 2.9 Hz), 135.1 (d, *J* = 11.3 Hz), 120.0 (d, *J* = 106.0 Hz), 114.3 (d, *J* = 13.3 Hz), 67.2, 55.5, 44.0 (d, *J* = 19.1 Hz), 37.9 (d, *J* = 2.4 Hz), 21.9, 17.3. ^{31}P NMR (162 MHz, $CDCl_3$) δ 18.43 (m). FTIR (neat), cm^{-1}: 1720 (m), 1592 (s), 1500 (m), 1289 (m), 1252 (s), 1177 (s), 1106 (vs), 1022 (s), 826 (s), 801 (s), 528 (s). HRMS (ESI): Calcd for $[C_{29}H_{34}NO_6PNa]^+$: 546.20160, found: 546.20154.

Isopropyl 2-methyl-4-oxo-4-((tricyclohexyl-λ[5]-phosphaneylidene)amino)butanoate (**28**)

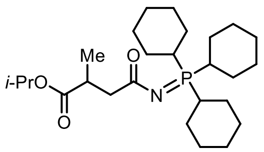

28

N-Acyl iminophosphorane **28** was prepared according to an analogous procedure as described for the preparation of **2** on page S4 using isopropyl methacrylate (43 µL, 0.30 mmol, 1 equiv) and tricyclohexylphosphine (0.17 g, 0.60 mmol, 2.0 equiv). The product was purified by FCC (40–100% ethyl acetate in pentane) and isolated as a yellow semi-solid (93 mg, 69%). ^{1}H NMR (400 MHz, $CDCl_3$) δ 4.94 (hept, *J* = 6.2 Hz, 1H), 2.88 (app h, *J* = 7.1 Hz, 1H), 2.71 (dd, *J* = 15.8, 7.0 Hz, 1H), 2.37–2.15 (m, 4H), 1.96–1.75 (m, 12H), 1.75–1.61 (m, 3H), 1.57–1.38 (m, 6H), 1.33–1.21 (m, 9H), 1.21–1.17 (m, 6H), 1.15 (d, *J* = 7.1 Hz, 3H). ^{13}C NMR (101 MHz, $CDCl_3$) δ 182.2 (d, *J* = 10.7 Hz), 176.5, 67.1, 43.8 (d, *J* = 19.0 Hz), 37.9 (d, *J* = 2.0 Hz), 34.1 (d, *J* = 54.5 Hz), 27.2(2) (d, *J* = 2.9 Hz), 27.1(8) (d, *J* = 11.7 Hz), 26.2 (d, *J* = 1.3 Hz), 21.9(6), 21.9(7), 17.3. ^{31}P NMR (162 MHz, $CDCl_3$) δ 38.99 (m). FTIR (neat), cm^{-1}: 2929 (s), 2850 (m), 1724 (s), 1565 (s), 1445 (w), 1347 (s), 1174 (m), 851 (m). HRMS (ESI): Calcd for $[C_{26}H_{46}NO_3PNa]^+$: 474.31075, found: 474.31029.

Methyl *N*[2],*N*[2]-bis(*tert*-butoxycarbonyl)-*N*[4]-(tris(4-methoxyphenyl)-λ[5]-phosphaneylidene)asparaginate (**29**)

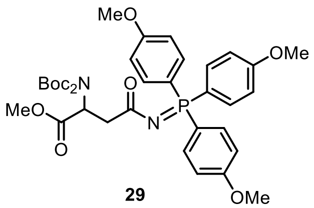

29

N-Acyl iminophosphorane **29** was prepared according to an analogous procedure as described for the preparation of **2** on page S4 using methyl 2-[bis(*tert*-butoxycarbonyl)amino]acrylate (90 mg, 0.30 mmol, 1 equiv). The product was purified by FCC (40–90% ethyl acetate in pentane) and isolated as a white amorphous solid (206 mg, 99%). ^{1}H NMR (400 MHz, $CDCl_3$) δ 7.71–7.58 (m, 6H), 6.99–6.87 (m, 6H), 5.82 (app t, *J* = 6.5 Hz, 1H), 3.83 (s, 9H), 3.69 (s, 3H), 3.35 (dd, *J* = 16.3, 6.9 Hz, 1H), 2.78 (dd, *J* = 16.3, 6.2 Hz, 1H), 1.43 (s, 18H). ^{13}C NMR (101 MHz, $CDCl_3$) δ 180.8 (d, *J* = 9.3 Hz), 171.9, 162.6 (d, *J* = 2.8 Hz), 152.2, 135.1 (d, *J* = 11.4 Hz), 120.0 (d, *J* = 106.6 Hz), 114.3 (d, *J* = 13.3 Hz), 82.8, 57.1 (d, *J* = 2.9 Hz), 55.5, 52.2, 42.1 (d, *J* = 19.2 Hz), 28.2. ^{31}P NMR (162 MHz, $CDCl_3$) δ 18.52 (m). FTIR (neat), cm^{-1}: 1741 (m), 1700 (w), 1593 (m), 1501 (w), 1253 (s), 1110 (s), 1023 (m), 828 (m), 802 (m), 531 (m). HRMS (ESI): Calcd for $[C_{36}H_{45}N_2O_{10}PNa]^+$: 719.27040, found: 719.26981.

Tert-butyl 3-methyl-4-oxo-4-((tris(4-methoxyphenyl)-λ[5]-phosphaneylidene)amino)butanoate (**30**)

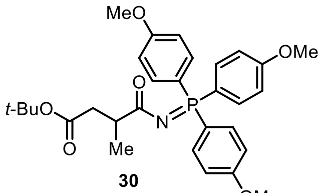

30

N-Acyl iminophosphorane **30** was prepared according to an analogous procedure as described for the preparation of **2** on page S4 using *tert*-butyl crotonate (48 µL, 0.30 mmol, 1 equiv). The product was purified by FCC (40–100% ethyl acetate in pentane) and isolated as a white amorphous solid (32 mg, 23%). ^{1}H NMR (400 MHz, $CDCl_3$) δ 7.68–7.58 (m, 6H), 6.98–6.88 (m, 6H), 3.83 (s, 9H), 3.02–2.89 (m, 1H), 2.83 (dd, *J* = 15.5, 7.0 Hz, 1H), 2.31 (dd, *J* = 15.5, 7.5 Hz, 1H), 1.38 (s, 9H), 1.24 (d, *J* = 7.0 Hz, 3H). ^{13}C NMR (101 MHz, $CDCl_3$) δ 186.5 (d, *J* = 10.4 Hz), 173.0, 162.6 (d, *J* = 2.9 Hz), 135.1 (d, *J* = 11.3 Hz), 120.4 (d, *J* = 105.8 Hz), 114.3 (d, *J* = 13.3 Hz), 79.7, 55.5, 41.1, 41.0 (d, *J* = 21.7 Hz), 28.3, 18.5. ^{31}P NMR (162 MHz, $CDCl_3$) δ 18.02 (m). FTIR (neat), cm^{-1}: 1720 (m), 1594 (s), 1501 (m), 1252 (s), 1110 (s), 1023 (s), 826 (s), 801 (s), 534 (s). HRMS (ESI): Calcd for $[C_{30}H_{36}NO_6PNa]^+$: 560.21725, found: 560.21737.

Tert-butyl 3-methyl-4-oxo-4-((tricyclohexyl-λ^5-phosphaneylidene)amino)butanoate (**31**)

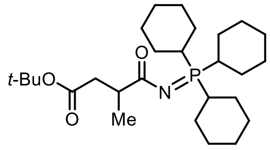

31

N-Acyl iminophosphorane **31** was prepared according to an analogous procedure as described for the preparation of **2** on page S4 using *tert*-butyl crotonate (48 µL, 0.30 mmol, 1 equiv) and tricyclohexylphosphine (0.17 g, 0.60 mmol, 2.0 equiv). The product was purified by FCC (40–100% ethyl acetate in pentane) and isolated as a white semi-solid (78 mg, 56%). ^{1}H NMR (400 MHz, $CDCl_3$) δ 2.80–2.63 (m, 2H), 2.27–2.08 (m, 4H), 1.94–1.71 (m, 12H), 1.71–1.58 (m, 3H), 1.54–1.41 (m, 6H), 1.39 (s, 9H), 1.31–1.15 (m, 9H), 1.12 (d, *J* = 6.9 Hz, 3H). ^{13}C NMR (101 MHz, $CDCl_3$) δ 186.2 (d, *J* = 11.1 Hz), 172.9, 79.5, 41.0, 40.9 (d, *J* = 21.2 Hz), 34.1 (d, *J* = 54.7 Hz), 28.3, 27.1(4) (d, *J* = 11.7 Hz), 27.1(3) (d, *J* = 3.4 Hz), 26.1 (d, *J* = 1.0 Hz), 18.9. ^{31}P NMR (162 MHz, $CDCl_3$) δ 38.40 (m). FTIR (neat), cm^{-1}: 2928 (m), 2853 (w), 1723 (m), 1567 (s), 1348 (m), 1150 (m), 727 (s). HRMS (ESI): Calcd for $[C_{27}H_{48}NO_3PNa]^+$: 488.32640, found: 488.32653.

Methyl 3-methyl-4-oxo-4-((tricyclohexyl-λ^5-phosphaneylidene)amino)butanoate (**32**)

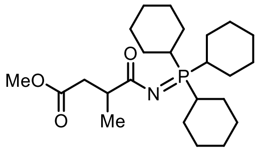

32

N-Acyl iminophosphorane **32** was prepared according to an analogous procedure as described for the preparation of **2** on page S4 using methyl crotonate (32 µL, 0.30 mmol, 1 equiv) and tricyclohexylphosphine (0.17 g, 0.60 mmol, 2.0 equiv). The product was purified by FCC (40–100% ethyl acetate in pentane) and isolated as a white amorphous solid (83 mg, 65%). ^{1}H NMR (400 MHz, $CDCl_3$) δ 3.61 (s, 3H), 2.88–2.70 (m, 2H), 2.36–2.10 (m, 4H), 1.94–1.74 (m, 12H), 1.74–1.59 (m, 3H), 1.57–1.36 (m, 6H), 1.36–1.18 (m, 9H), 1.16 (d, *J* = 6.9 Hz, 3H). ^{13}C NMR (101 MHz, $CDCl_3$) δ 185.8 (d, *J* = 11.1 Hz), 174.0, 51.3, 40.8 (d, *J* = 18.7 Hz), 39.7 (d, *J* = 1.5 Hz), 34.1 (d, *J* = 54.7 Hz), 27.2 (d, *J* = 11.2 Hz), 27.1 (d, *J* = 3.5 Hz), 26.2 (d, *J* = 1.2 Hz), 19.1. ^{31}P NMR (162 MHz, $CDCl_3$) δ 38.49 (m). FTIR (neat), cm^{-1}: 2928 (s), 2849 (m), 2192 (w), 1738 (m), 1725 (m), 1557 (s), 1445 (m), 1353 (s), 1166 (s), 727 (s), 548 (m). HRMS (ESI): Calcd for $[C_{24}H_{42}NO_3PNa]^+$: 446.27945, found: 446.27905.

Dimethyl 2-((tricyclohexyl-λ^5-phosphaneylidene)carbamoyl)succinate (**33**)

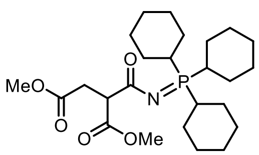

33

N-Acyl iminophosphorane **33** was prepared according to an analogous procedure as described for the preparation of **2** on page S4 using dimethyl maleate (38 µL, 0.30 mmol, 1 equiv) and tricyclohexylphosphine (0.17 g, 0.60 mmol, 2.0 equiv). The product was purified by FCC (40–80% ethyl acetate in pentane) and isolated as a colorless oil (84 mg, 60%). ^{1}H NMR (400 MHz, $CDCl_3$) δ 3.80–3.71 (m, 1H), 3.68 (s, 3H), 3.63 (s, 3H), 3.01–2.83 (m, 2H), 2.30–2.10 (m, 3H), 1.92–1.74 (m, 12H), 1.74–1.60 (m, 3H), 1.55–1.34 (m, 6H), 1.34–1.12 (m, 9H). ^{13}C NMR (101 MHz, $CDCl_3$) δ 177.0 (d, *J* = 10.2 Hz), 173.0, 172.3, 52.8 (d, *J* = 19.6 Hz), 51.9, 51.7, 34.9, 33.9 (d, *J* = 54.5 Hz), 27.1 (d, *J* = 11.7 Hz), 27.0 (d, *J* = 3.1 Hz), 26.1 (d, *J* = 1.3 Hz). ^{31}P NMR (162 MHz, $CDCl_3$) δ 39.07 (m). FTIR (neat), cm^{-1}: 2927 (s), 2851 (m), 1736 (s), 1586 (s), 1446 (m), 1337 (s), 1154 (s), 852 (m). HRMS (ESI): Calcd for $[C_{25}H_{42}NO_5PNa]^+$: 490.26928, found: 490.26865.

Tert-butyl 3-(2-ethoxy-2-oxoethyl)-3-((tricyclohexyl-λ^5-phosphaneylidene)carbamoyl)azetidine-1-carboxylate (**34**)

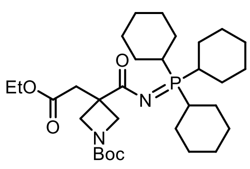

34

N-Acyl iminophosphorane **34** was prepared according to an analogous procedure as described for the preparation of **2** on page S4 using *tert*-butyl 3-(2-ethoxy-2-oxoethylidene)azetidine-1-carboxylate (72 mg, 0.30 mmol, 1 equiv) and tricyclohexylphosphine (0.17 g, 0.60 mmol, 2.0 equiv). The product was purified by FCC (40–50% ethyl acetate in pentane) and isolated as a white amorphous solid (137 mg, 81%). ^{1}H NMR (400 MHz, $CDCl_3$) δ 4.50–4.13 (m, 2H), 4.05 (q, *J* = 7.1 Hz, 2H), 3.81–3.66 (m, 2H), 3.06–2.74 (m, 2H), 2.31–2.11 (m, 3H), 1.96–1.75 (m, 12H), 1.75–1.61 (m, 3H), 1.56–1.33 (m, 15H), 1.33–1.13 (m, 12H). ^{13}C NMR (101 MHz, $CDCl_3$) δ 182.6 (d, *J* = 10.1 Hz), 172.0, 156.8, 79.1, 60.2, 59.0, 58.1, 42.9 (d, *J* = 19.5 Hz), 41.5 (d, *J* = 0.9 Hz), 34.0 (d, *J* = 54.8 Hz), 28.6, 27.1 (d, *J* = 11.8 Hz), 27.1 (d, *J* = 3.4 Hz), 26.1 (d, *J* = 1.2 Hz), 14.3. ^{31}P NMR (162 MHz, $CDCl_3$) δ 39.05 (m). FTIR (neat), cm^{-1}: 2928 (s), 2852 (m), 1733 (w), 1692 (s), 1581 (s), 1362 (s), 1342 (m), 1176 (s), 849 (m). HRMS (ESI): Calcd for $[C_{31}H_{53}N_2O_5PNa]^+$: 587.35843, found: 587.35869.

5-Oxo-*N*-(tris(4-methoxyphenyl)-λ^5-phosphaneylidene)tetrahydrofuran-3-carboxamide (**35**)

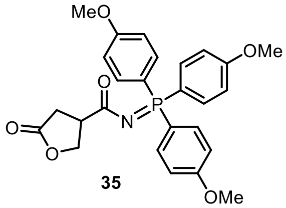

35

N-Acyl iminophosphorane **35** was prepared according to an analogous procedure as described for the preparation of **2** on page S4 using 2(5*H*)-furanone (21 µL, 0.30 mmol, 1 equiv). The product was purified by FCC (40–100% ethyl acetate in pentane) and isolated as a colorless oil (48 mg, 33%). ^{1}H NMR (400 MHz, $CDCl_3$) δ 7.67–7.55 (m, 6H), 7.03–6.92 (m, 6H), 4.63–4.49 (m, 2H), 3.84 (s, 9H), 3.50–3.39 (m, 1H), 3.05 (dd, *J* = 17.8, 6.9 Hz, 1H), 2.71 (dd, *J* = 17.8, 9.4 Hz, 1H). ^{13}C NMR (101 MHz, $CDCl_3$) δ 181.2 (d, *J* = 9.4 Hz), 178.0, 162.9 (d, *J* = 2.9 Hz), 134.9 (d, *J* = 11.4 Hz), 119.2 (d, *J* = 106.8 Hz), 114.5 (d, *J* = 13.4 Hz), 72.0, 55.6, 45.2 (d, *J* = 19.7 Hz), 33.0 (d, *J* = 1.7 Hz). ^{31}P NMR (162 MHz, $CDCl_3$) δ 19.28 (m). FTIR (neat), cm^{-1}: 1769 (s), 1591 (s), 1500 (m), 1252 (s), 1177 (s), 1108 (s), 1020 (s), 826 (m), 801 (s), 530 (s). HRMS (ESI): Calcd for $[C_{26}H_{26}NO_6PNa]^+$: 502.13900, found: 502.13880.

5-Oxo-*N*-(tricyclohexyl-λ^5-phosphaneylidene)tetrahydrofuran-3-carboxamide (**36**)

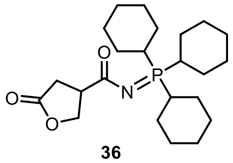

36

N-Acyl iminophosphorane **36** was prepared according to an analogous procedure as described for the preparation of **2** on page S4 using 2(5*H*)-furanone (21 µL, 0.30 mmol, 1 equiv) and tricyclohexylphosphine (0.17 g, 0.60 mmol, 2.0 equiv). The product was purified by FCC (60–100% ethyl acetate in pentane) and isolated as a yellow amorphous solid (59 mg, 49%). ^{1}H NMR (400 MHz, $CDCl_3$) δ 4.53–4.38 (m, 2H), 3.33–3.19 (m, 1H), 2.92 (dd, *J* = 17.6, 6.6 Hz, 1H), 2.62 (dd, *J* = 17.6, 9.3 Hz, 1H), 2.31–2.11 (m, 3H), 1.97–1.76 (m, 12H), 1.76–1.65 (m, 3H), 1.55–1.36 (m, 6H), 1.36–1.13 (m, 9H). ^{13}C NMR (101 MHz, $CDCl_3$) δ 180.9 (d, *J* = 10.2 Hz), 178.1, 72.3, 45.2 (d, *J* = 19.2 Hz), 34.0 (d, *J* = 54.4 Hz), 33.3, 27.1(4) (d, *J* = 3.3 Hz), 27.0(6) (d, *J* = 11.7 Hz), 26.1 (d, *J* = 1.4 Hz). ^{31}P NMR (162 MHz, $CDCl_3$) δ 39.51 (m). FTIR (neat), cm^{-1}: 2929 (s), 2851 (m), 1770 (s), 1573 (s), 1367 (m), 1343 (m), 1270 (m), 1163 (s), 852 (s), 835 (s), 549 (m). HRMS (ESI): Calcd for $[C_{23}H_{38}NO_3PNa]^+$: 430.24815, found: 430.24779.

2-Oxo-*N*-(tricyclohexyl-λ^5-phosphaneylidene)tetrahydro-2*H*-pyran-4-carboxamide (**37**)

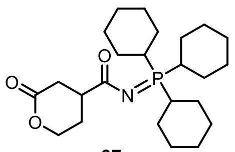

37

N-Acyl iminophosphorane **37** was prepared according to an analogous procedure as described for the preparation of **2** on page S4 using 5,6-dihydro-2*H*-pyran-2-one (29 µL, 90% purity, 0.30 mmol, 1 equiv) and tricyclohexylphosphine (0.17 g, 0.60 mmol, 2.0 equiv). The product was purified by FCC (80–100% ethyl acetate in pentane) and isolated as a white amorphous solid (126 mg, 99%). ^{1}H NMR (400 MHz, $CDCl_3$) δ 4.43 (ddd, *J* = 11.3, 6.8, 4.7 Hz, 1H), 4.26 (ddd, *J* = 11.3, 7.3, 4.6 Hz, 1H), 2.93–2.82 (m, 1H), 2.82–2.72 (m, 1H), 2.72–2.62 (m, 1H), 2.28–2.14 (m, 3H), 2.14–1.95 (m, 2H), 1.92–1.76 (m, 12H), 1.76–1.62 (m, 3H), 1.54–1.36 (m, 6H), 1.36–1.14 (m, 9H). ^{13}C NMR (101 MHz, $CDCl_3$) δ 182.9 (d, *J* = 10.8 Hz), 172.2, 68.4, 41.5 (d, *J* = 18.9 Hz), 34.7, 34.0 (d, *J* = 54.5 Hz), 27.2 (d, *J* = 3.3 Hz), 27.1(3), 27.0(7) (d, *J* = 11.9 Hz), 26.1 (d, *J* = 1.4 Hz). ^{31}P NMR (162 MHz, $CDCl_3$) δ 39.45 (m). FTIR (neat), cm^{-1}: 2925 (s), 2851 (w), 1737 (s), 1557 (s), 1204 (m), 1077 (w), 723 (s). HRMS (ESI): Calcd for $[C_{24}H_{40}NO_3PNa]^+$: 444.26380, found: 444.26333.

3-Oxo-*N*-(tricyclohexyl-λ^5-phosphaneylidene)cyclohexane-1-carboxamide (**38**)

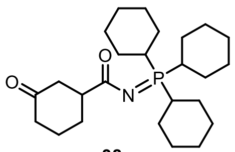

38

N-Acyl iminophosphorane **38** was prepared according to an analogous procedure as described for the preparation of **2** on page S4 using 2-cyclohexen-1-one (30 µL, 0.30 mmol, 1 equiv) and tricyclohexylphosphine (0.17 g, 0.60 mmol, 2.0 equiv). The product was purified by FCC (40–100% ethyl acetate in pentane) and isolated as a yellow amorphous solid (119 mg, 94%). ^{1}H NMR (400 MHz, $CDCl_3$) δ 2.78–2.65 (m, 1H), 2.65–2.54 (m, 1H), 2.48 (dd, *J* = 14.3, 4.2 Hz, 1H), 2.37–2.13 (m, 5H), 2.13–1.96 (m, 2H), 1.96–1.61 (m, 17H), 1.58–1.37 (m, 6H), 1.37–1.16 (m, 9H). ^{13}C NMR (101 MHz, $CDCl_3$) δ 212.7, 184.3 (d, *J* = 10.5 Hz), 48.8 (d, *J* = 18.4 Hz), 45.8, 41.4, 34.1 (d, *J* = 54.3 Hz), 29.5, 27.2 (d, *J* = 3.4 Hz), 27.1 (d, *J* = 11.6 Hz), 26.1 (d, *J* = 1.0 Hz), 25.1. ^{31}P NMR (162 MHz, $CDCl_3$) δ 39.12 (m). FTIR (neat), cm^{-1}: 2923 (s), 2848 (m), 1710 (s), 1563 (s), 1445 (w), 1358 (m), 850 (m), 549 (m). HRMS (ESI): Calcd for $[C_{25}H_{42}NO_2PNa]^+$: 442.28454, found: 442.28413.

2,2-Dimethyl-5-oxo-*N*-(tricyclohexyl-λ^5-phosphaneylidene)cyclohexane-1-carboxamide (**39**)

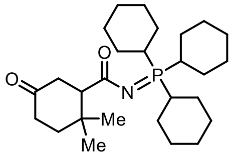

39

N-Acyl iminophosphorane **39** was prepared according to an analogous procedure as described for the preparation of **2** on page S4 using 4,4-dimethyl-2-cyclohexen-1-one (41 µL, 97% purity, 0.30 mmol, 1 equiv) and tricyclohexylphosphine (0.17 g, 0.60 mmol, 2.0 equiv). The product was purified by FCC (40–70% ethyl acetate in pentane) and isolated as a white amorphous solid (103 mg, 76%). ^{1}H NMR (400 MHz, $CDCl_3$) δ 2.59 (dd, *J* = 15.3, 6.9 Hz, 1H), 2.50 (app q, *J* = 4.8 Hz, 1H), 2.43–2.31 (m, 2H), 2.31–2.04 (m, 5H), 1.99–1.75 (m, 12H), 1.75–1.62 (m, 3H), 1.59–1.34 (m, 7H), 1.34–1.18 (m, 9H), 1.16 (s, 3H), 1.14 (s, 3H). ^{13}C NMR (101 MHz, $CDCl_3$) δ 212.4, 183.9 (d, *J* = 11.5 Hz), 57.7 (d, *J* = 17.7 Hz), 42.4, 37.9, 37.7, 34.2 (d, *J* = 54.4 Hz), 32.2, 28.4, 27.2(9) (d, *J* = 3.5 Hz), 27.2(6) (d, *J* = 3.6 Hz), 27.1(4) (d, *J* = 11.7 Hz), 27.1(2) (d, *J* = 11.8 Hz), 26.2 (d, *J* = 0.9 Hz), 25.5. ^{31}P NMR (162 MHz, $CDCl_3$) δ 39.69 (m). FTIR (neat), cm^{-1}: 2924 (s), 2850 (m), 1703 (s), 1570 (s), 1445 (w), 1337 (s), 829 (s), 729 (s), 549 (m). HRMS (ESI): Calcd for $[C_{27}H_{46}NO_2PNa]^+$: 470.31584, found: 470.31576.

3-Oxo-*N*-(tricyclohexyl-λ^5-phosphaneylidene)cycloheptane-1-carboxamide (**40**)

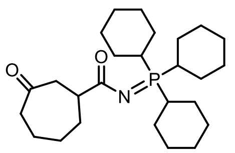

40

N-Acyl iminophosphorane **40** was prepared according to an analogous procedure as described for the preparation of **2** on page S4 using 2-cyclohepten-1-one (35 µL, 96% purity, 0.30 mmol, 1 equiv) and tricyclohexylphosphine (0.17 g, 0.60 mmol, 2.0 equiv). The product was purified by FCC (40–70% ethyl acetate in pentane) and isolated as a white amorphous solid (120 mg, 92%). ^{1}H NMR (400 MHz, $CDCl_3$) δ 2.85-2.64 (m, 2H), 2.64–2.53 (m, 1H), 2.53–2.40 (m, 2H), 2.26–2.02 (m, 4H), 1.96–1.54 (m, 19H), 1.54–1.35 (m, 7H), 1.35–1.12 (m, 9H). ^{13}C NMR (101 MHz, $CDCl_3$) δ 215.5, 185.5 (d, *J* = 11.2 Hz), 48.4, 46.6 (d, *J* = 18.4 Hz), 44.5, 35.3, 34.1 (d, *J* = 54.5 Hz), 29.3, 27.2 (d, *J* = 3.1 Hz), 27.1 (d, *J* = 11.9 Hz), 26.1 (d, *J* = 1.2 Hz), 24.6. ^{31}P NMR (162 MHz, $CDCl_3$) δ 38.90 (m). FTIR (neat), cm^{-1}: 2922 (vs), 2848 (s), 1698 (s), 1571 (vs), 1445 (m), 1338 (s), 870 (m), 547 (m). HRMS (ESI): Calcd for $[C_{26}H_{44}NO_2PNa]^+$: 456.30019, found: 456.30056.

2-Methyl-4-oxo-4-phenyl-*N*-(tricyclohexyl-λ^5-phosphaneylidene)butanamide (**41**)

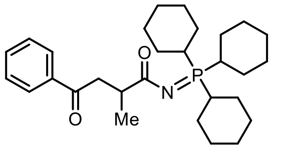

41

N-Acyl iminophosphorane **41** was prepared according to an analogous procedure as described for the preparation of **2** on page S4 using 1-phenyl-but-2-en-1-one (55 mg, 80% purity, 0.30 mmol, 1 equiv) and tricyclohexylphosphine (0.17 g, 0.60 mmol, 2.0 equiv). The product was purified by FCC (50–100% ethyl acetate in pentane) and isolated as a yellow semi-solid (68 mg, 48%). ^{1}H NMR (400 MHz, $CDCl_3$) δ 8.05–7.96 (m, 2H), 7.54–7.50 (m, 1H), 7.46–7.37 (m, 2H), 3.61 (dd, *J* = 16.4, 7.6 Hz, 1H), 3.08 (app h, *J* = 6.8 Hz, 1H), 2.75 (dd, *J* = 16.4, 6.3 Hz, 1H), 2.26–2.09 (m, 3H), 1.91–1.73 (m, 12H), 1.73–1.60 (m, 3H), 1.53–1.32 (m, 6H), 1.32–1.10 (m, 12H). ^{13}C NMR (101 MHz, $CDCl_3$) δ 200.3, 186.4 (d, *J* = 11.0 Hz), 138.0, 132.5, 128.5, 128.4, 43.9, 40.4 (d, *J* = 18.7 Hz), 34.1 (d, *J* = 54.6 Hz), 27.2 (d, *J* = 11.8 Hz), 27.1 (d, *J* = 3.4 Hz), 26.2 (d, *J* = 1.3 Hz), 19.3. ^{31}P NMR (162 MHz, $CDCl_3$) δ 38.52 (m). FTIR (neat), cm^{-1}: 2925 (s), 2851 (m), 1683 (m), 1567 (s), 1446 (s), 1338 (s), 1004 (m), 752 (m), 690 (m), 549 (m). HRMS (ESI): Calcd for $[C_{29}H_{44}NO_2PNa]^+$: 492.30019, found: 492.29991.

4-Oxo-*N*-(tris(4-methoxyphenyl)-λ^5-phosphaneylidene)nonanamide (**42**)

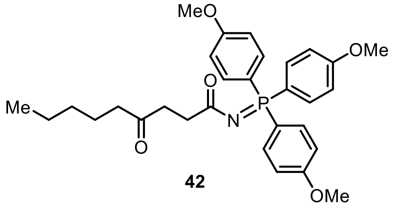

42

N-Acyl iminophosphorane **42** was prepared according to an analogous procedure as described for the preparation of **2** on page S4 using 1-octen-3-one (46 µL, 0.30 mmol, 1 equiv). The product was purified by FCC (40–100% ethyl acetate in pentane) and isolated as a yellow oil (60 mg, 38%). ^{1}H NMR (400 MHz, $CDCl_3$) δ 7.68–7.55 (m, 6H), 7.01–6.86 (m, 6H), 3.82 (s, 9H), 2.83–2.67 (m, 4H), 2.41 (t, *J* = 7.5 Hz, 2H), 1.61–1.49 (m, 2H), 1.35–1.13 (m, 4H), 0.84 (t, *J* = 7.1 Hz, 3H). ^{13}C NMR (101 MHz, $CDCl_3$) δ 211.4, 183.4 (d, *J* = 9.9 Hz), 162.7 (d, *J* = 2.9 Hz), 135.0 (d, *J* = 11.3 Hz), 119.9 (d, *J* = 106.0 Hz), 114.3 (d, *J* = 13.3 Hz), 55.5, 42.9, 39.6 (d, *J* = 2.9 Hz), 34.5 (d, *J* = 19.8 Hz), 31.6, 23.7, 22.6, 14.1. ^{31}P NMR (162 MHz, $CDCl_3$) δ 18.50 (m). FTIR (neat), cm^{-1}: 2929 (w), 1709 (m), 1593 (s), 1500 (m), 1251 (s), 1178 (m), 1109 (s), 1022 (s), 826 (m), 801 (m), 529 (s). HRMS (ESI): Calcd for $[C_{30}H_{36}NO_5PNa]^+$: 544.22233, found: 544.22242.

4-Morpholino-4-oxo-*N*-(tris(4-methoxyphenyl)-λ5-phosphaneylidene)butanamide (**43**)

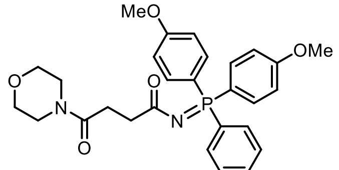

43

N-Acyl iminophosphorane **43** was prepared according to an analogous procedure as described for the preparation of **2** on page S4 using acryloylmorpholine (38 µL, 0.30 mmol, 1 equiv). The product was purified by FCC (2–5% methanol in ethyl acetate) and isolated as a yellow oil (117 mg, 72%). ^{1}H NMR (400 MHz, $CDCl_3$) δ 7.73–7.53 (m, 6H), 7.02–6.86 (m, 6H), 3.82 (s, 9H), 3.68–3.55 (m, 6H), 3.55–3.42 (m, 2H), 2.83 (t, *J* = 7.3 Hz, 2H), 2.67 (t, *J* = 7.3 Hz, 2H). ^{13}C NMR (101 MHz, $CDCl_3$) δ 183.4 (d, *J* = 9.7 Hz), 172.0, 162.7 (d, *J* = 2.9 Hz), 135.0 (d, *J* = 11.3 Hz), 120.0 (d, *J* = 106.1 Hz), 114.4 (d, *J* = 13.3 Hz), 67.1, 66.9, 55.5, 46.1, 42.1, 35.6 (d, *J* = 20.2 Hz), 30.0 (d, *J* = 3.2 Hz). ^{31}P NMR (162 MHz, $CDCl_3$) δ 18.02 (m). FTIR (neat), cm^{-1}: 1640 (m), 1592 (s), 1500 (m), 1251 (s), 1178 (m), 1108 (s), 1022 (s), 827 (m), 801 (m), 530 (s). HRMS (ESI): Calcd for $[C_{29}H_{33}N_2O_6PNa]^+$: 559.19684, found: 559.19705.

Diethyl (3-oxo-3-((tris(4-methoxyphenyl)-λ5-phosphaneylidene)amino)propyl)phosphonate (**44**)

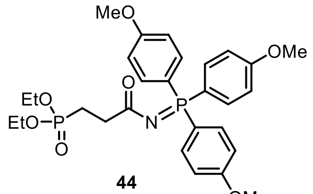

44

N-Acyl iminophosphorane **44** was prepared according to an analogous procedure as described for the preparation of **2** on page S4 using diethyl vinylphosphonate (46 µL, 0.30 mmol, 1 equiv). The product was purified by FCC (2–10% methanol in ethyl acetate) and isolated as a yellow oil (140 mg, 84%). ^{1}H NMR (400 MHz, $CDCl_3$) δ 7.68–7.56 (m, 6H), 6.98–6.88 (m, 6H), 4.15–3.99 (m, 4H), 3.82 (s, 9H), 2.79–2.63 (m, 2H), 2.26–2.11 (m, 2H), 1.28 (t, *J* = 7.1 Hz, 6H). ^{13}C NMR (101 MHz, $CDCl_3$) δ 182.5 (dd, *J* = 19.6, 9.8 Hz), 162.7 (d, *J* = 2.9 Hz), 135.0 (d, *J* = 11.3 Hz), 119.8 (d, *J* = 106.1 Hz), 114.4 (d, *J* = 13.3 Hz), 61.5 (d, *J* = 6.3 Hz), 55.5, 33.3 (dd, *J* = 20.6, 4.2 Hz), 22.9 (dd, *J* = 140.7, 3.0 Hz), 16.6 (d, *J* = 6.1 Hz). ^{31}P NMR (162 MHz, $CDCl_3$) δ 33.07 (m), 18.18 (m). FTIR (neat), cm^{-1}: 1592 (s), 1500 (m), 1251 (s), 1178 (m), 1109 (s), 1019 (vs), 824 (s), 801 (s), 528 (s). HRMS (ESI): Calcd for $[C_{28}H_{35}NO_7P_2Na]^+$: 582.17810, found: 582.17777.

3-(Phenylsulfonyl)-*N*-(tris(4-methoxyphenyl)-λ5-phosphaneylidene)propenamide (**45**)

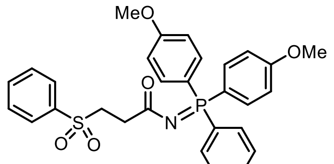

45

N-Acyl iminophosphorane **45** was prepared according to an analogous procedure as described for the preparation of **2** on page S4 using phenyl vinyl sulfone (51 mg, 0.30 mmol, 1 equiv). The product was purified by FCC (40–100% ethyl acetate in pentane) and isolated as a yellow amorphous solid (19 mg, 11%). ^{1}H NMR (400 MHz, $CDCl_3$) δ 7.95–7.88 (m, 2H), 7.62–7.54 (m, 7H), 7.54–7.48 (m, 2H), 6.99–6.89 (m, 6H), 3.83 (s, 9H), 3.61–3.51 (m, 2H), 2.85–2.73 (m, 2H). ^{13}C NMR (101 MHz, $CDCl_3$) δ 179.7 (d, *J* = 9.5 Hz), 162.8 (d, *J* = 2.9 Hz), 139.3, 135.0 (d, *J* = 11.4 Hz), 133.6, 129.3, 128.4, 119.4 (d, *J* = 106.5 Hz), 114.4 (d, *J* = 13.4 Hz), 55.6, 53.9 (d, *J* = 3.3 Hz), 33.7 (d, *J* = 20.9 Hz). ^{31}P NMR (162 MHz, $CDCl_3$) δ 18.78 (m). FTIR (neat), cm^{-1}: 1592 (s), 1500 (m), 1290 (m), 1252 (s), 1109 (s), 1021 (m), 827 (m), 801 (m), 528 (s). HRMS (ESI): Calcd for $[C_{30}H_{30}NO_6PSNa]^+$: 586.14237, found: 586.14255.

(3*S*,8*S*,9*S*,10*R*,13*R*,14*S*,17*R*)-10,13-Dimethyl-17-((*R*)-6-methylheptan-2-yl)-2,3,4,7,8,9,10,11,12,13,14,15,16,17-tetradecahydro-1*H*-cyclopenta[*a*]phenanthren-3-yl 4-oxo-4-((tris(4-methoxyphenyl)-λ^5-phosphaneylidene)amino)butanoate (**46**)

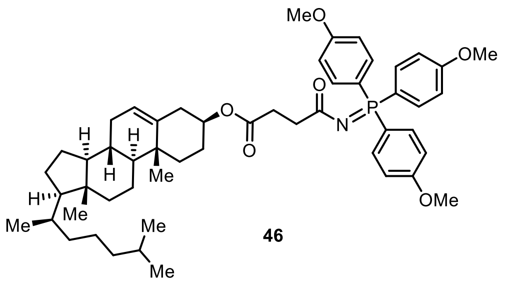

N-Acyl iminophosphorane **46** was prepared according to an analogous procedure as described for the preparation of **2** on page S4 using alkene **81** (132 mg, 0.30 mmol, 1 equiv). The product was purified by FCC (40–100% ethyl acetate in pentane) and isolated as a white amorphous solid (232 mg, 92%). ^{1}H NMR (600 MHz, $CDCl_3$) δ 7.69–7.59 (m, 6H), 6.98–6.89 (m, 6H), 5.30–5.26 (m, 1H), 4.62–4.53 (m, 1H), 3.82 (s, 9H), 2.78–2.71 (m, 2H), 2.71–2.64 (m, 2H), 2.28–2.16 (m, 2H), 2.00 (dt, *J* = 12.6, 3.4 Hz, 1H), 1.94 (dtd, *J* = 17.4, 5.2, 2.6 Hz, 1H), 1.87–1.72 (m, 3H), 1.61–1.19 (m, 11H), 1.19–1.03 (m, 7H), 1.03–0.97 (m, 2H), 0.96 (s, 3H), 0.95–0.88 (m, 4H), 0.87 (d, *J* = 2.7 Hz, 3H), 0.85 (d, *J* = 2.7 Hz, 3H), 0.67 (s, 3H). ^{13}C NMR (151 MHz, $CDCl_3$) δ 182.7 (d, *J* = 9.9 Hz), 173.2, 162.5 (d, *J* = 2.9 Hz), 139.9, 134.9 (d, *J* = 11.3 Hz), 122.3, 119.8 (d, *J* = 106.0 Hz), 114.1 (d, *J* = 13.3 Hz), 73.6, 56.7, 56.1, 55.3, 50.0, 42.3, 39.7, 39.5, 38.1, 37.0, 36.6, 36.2, 35.8, 35.2 (d, *J* = 19.7 Hz), 31.8(9), 31.8(5), 31.8(3) (d, *J* = 2.9 Hz), 28.2, 28.0, 27.7, 24.3, 23.8, 22.8, 22.5, 21.0, 19.3, 18.7, 11.8. ^{31}P NMR (242 MHz, $CDCl_3$) δ 18.27 (m). FTIR (neat), cm^{-1}: 2933 (m), 1728 (m), 1594 (s), 1501 (m), 1253 (s), 1112 (s), 1025 (m), 826 (s), 801 (s), 662 (w), 532 (s). HRMS (ESI): Calcd for $[C_{52}H_{70}NO_6PNa]^+$: 858.48330, found: 858.48316.

2-((1a*R*,7a*S*,8*S*,10a*S*,10b*R*,*Z*)-1a,5-Dimethyl-9-oxo-1a,2,3,6,7,7a,8,9,10a,10b-decahydrooxireno[2',3':9,10]cyclodeca[1,2-*b*]furan-8-yl)-*N*-(tris(4-methoxyphenyl)-λ^5-phosphaneylidene)acetamide (**47**)

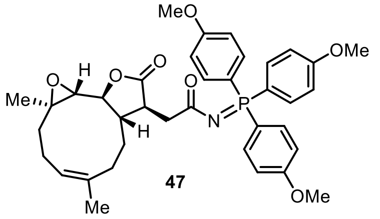

N-Acyl iminophosphorane **47** was prepared according to an analogous procedure as described for the preparation of **2** on page S4 using parthenolide (75 mg, 0.30 mmol, 1 equiv). The product was purified by FCC (30–100% acetone in pentane) and isolated as a white amorphous solid (176 mg, 91%). ^{1}H NMR (600 MHz, $CDCl_3$) δ 7.69–7.60 (m, 6H), 6.99–6.92 (m, 6H), 4.84–4.76 (m, 1H), 3.82 (s, 9H), 3.75 (app t, *J* = 9.0 Hz, 1H), 2.97 (dd, *J* = 16.0, 4.6 Hz, 1H), 2.75 (ddd, *J* = 16.0, 5.0, 3.2 Hz, 1H), 2.66 (app dt, *J* = 12.0, 4.8 Hz, 1H), 2.39 (d, *J* = 9.0 Hz, 1H), 2.36–2.21 (m, 2H), 2.11–1.98 (m, 3H), 1.92–1.78 (m, 2H), 1.61 (s, 3H), 1.57–1.47 (m, 1H), 1.23 (s, 3H), 0.99 (app td, *J* = 12.9, 5.7 Hz, 1H). ^{13}C NMR (151 MHz, $CDCl_3$) δ 180.6 (d, *J* = 9.9 Hz), 177.4, 162.7 (d, *J* = 2.8 Hz), 135.1 (d, *J* = 11.5 Hz), 134.8, 124.7, 119.7 (d, *J* = 106.4 Hz), 114.4 (d, *J* = 13.4 Hz), 82.2, 66.6, 61.3, 55.5, 48.4, 45.9 (d, *J* = 1.5 Hz), 41.4, 38.3 (d, *J* = 19.3 Hz), 36.8, 30.1, 24.2, 17.3, 17.0. ^{31}P NMR (242 MHz, $CDCl_3$) δ 19.28 (m). FTIR (neat), cm^{-1}: 2924 (w), 1769 (s), 1592 (s), 1500 (m), 1291 (m), 1253 (s), 1176 (m), 1110 (s), 1022 (m), 827 (m), 801 (m), 529 (s). HRMS (ESI): Calcd for $[C_{37}H_{42}NO_7PNa]^+$: 666.25911, found: 666.25936.

N^1-(2-((2-(Dimethylamino)ethyl)(methyl)amino)-4-methoxy-5-((4-(1-methyl-1*H*-indol-3-yl)pyrimidin-2-yl)amino)phenyl)-N^4-(tris(4-methoxyphenyl)-λ^5-phosphaneylidene)succinimide (**48**)

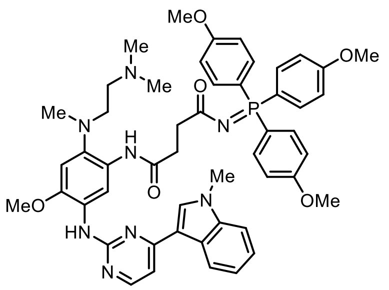

48

N-Acyl iminophosphorane **48** was prepared according to an analogous procedure as described for the preparation of **2** on page S4 using osimertinib (150 mg, 0.30 mmol, 1 equiv). The product was purified by FCC (5–10% methanol in dichloromethane) and isolated as a yellow amorphous solid (199 mg, 74%). ^{1}H NMR (400 MHz, $CDCl_3$) δ 9.84 (s, 1H), 9.41 (s, 1H), 9.30 (s, 1H), 8.35 (d, *J* = 5.3 Hz, 1H), 8.05–7.96 (m, 1H), 7.74 (s, 1H), 7.64–7.48 (m, 6H), 7.32–7.15 (m, 4H), 6.86–6.71 (m, 7H), 3.93 (s, 3H), 3.88 (s, 3H), 3.68 (s, 9H), 3.05–2.89 (m, 4H), 2.83 (t, *J* = 6.5 Hz, 2H), 2.57 (s, 3H), 2.49–2.36 (m, 2H), 2.27 (s, 6H). ^{13}C NMR (101 MHz, $CDCl_3$) δ 183.4 (d, *J* = 10.0 Hz), 170.9, 162.6 (d, *J* = 2.9 Hz), 162.4, 159.7, 158.0, 143.8, 138.4, 135.9, 134.9 (d, *J* = 11.3 Hz), 133.7, 129.8, 127.8, 126.0, 121.7, 120.9, 120.2, 120.1, 119.2, 114.2 (d, *J* = 13.3 Hz), 113.5, 110.3, 109.7, 107.9, 104.4, 57.0, 56.3, 55.4, 45.1, 44.2, 36.0 (d, *J* = 19.6 Hz), 34.8 (d, *J* = 2.6 Hz), 33.3. ^{31}P NMR (162 MHz, $CDCl_3$) δ 18.49 (m). FTIR (neat), cm^{-1}: 2933 (w), 1671 (w), 1577 (s), 1499 (s), 1253 (s), 1178 (m), 1108 (s), 1022 (m), 802 (m), 724 (s), 528 (s). HRMS (ESI): Calcd for $[C_{50}H_{55}N_8O_6PNa]^+$: 917.38744, found: 917.38765.

2-Methoxy-3-(4-(trifluoromethyl)phenyl)-*N*-(tris(4-methoxyphenyl)-λ^5-phosphaneylidene)propenamide (**73**)

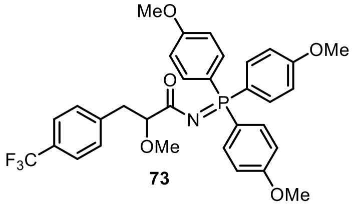

73

N-Acyl iminophosphorane **73** was prepared according to an analogous procedure as described for the preparation of **2** on page S4 using alkene *Z*-**78** (61 mg, 0.30 mmol, 1 equiv). The product was purified by FCC (30–100% ethyl acetate in pentane) and isolated as a colorless oil (23 mg, 13%). ^{1}H NMR (500 MHz, $CDCl_3$) δ 7.59–7.51 (m, 6H), 7.41 (d, *J* = 8.1 Hz, 2H), 7.34 (d, *J* = 8.0 Hz, 2H), 6.96–6.89 (m, 6H), 4.05 (ddd, *J* = 7.5, 5.4, 1.2 Hz, 1H), 3.84 (s, 9H), 3.42 (s, 3H), 3.20 (dd, *J* = 13.9, 5.4 Hz, 1H), 3.13 (dd, *J* = 13.9, 7.6 Hz, 1H). ^{13}C{^{19}F} NMR (126 MHz, $CDCl_3$) δ 181.8 (d, *J* = 10.6 Hz), 162.8 (d, *J* = 2.9 Hz), 143.7, 135.1 (d, *J* = 11.4 Hz), 130.1, 128.1, 125.0, 124.7, 119.7 (d, *J* = 106.2 Hz), 114.3 (d, *J* = 13.4 Hz), 85.1 (d, *J* = 21.1 Hz), 57.9, 55.5, 40.1. ^{19}F{^{1}H} NMR (470 MHz, $CDCl_3$) δ −62.19. ^{31}P NMR (202 MHz, $CDCl_3$) δ 19.82 (m). FTIR (neat), cm^{-1}: 1593 (s), 1500 (m), 1323 (m), 1252 (s), 1105 (vs), 1019 (s), 825 (m), 801 (m), 530 (s). HRMS (ESI): Calcd for $[C_{32}H_{31}NO_5PF_3Na]^+$: 620.17842, found: 620.17823.

N-(Tris(4-methoxyphenyl)-λ^5-phosphaneylidene)-2,3-dihydro-1*H*-indene-2-carboxamide (**74**)

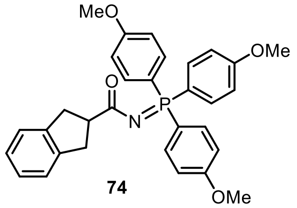

74

N-Acyl iminophosphorane **74** was prepared according to an analogous procedure as described for the preparation of **2** on page S4 using indene (35 µL, 0.30 mmol, 1 equiv). The product was purified by FCC (15–40% ethyl acetate in dichloromethane) and isolated as a white amorphous solid (11 mg, 7%). ^{1}H NMR (400 MHz, $CDCl_3$) δ 7.68–7.58 (m, 6H), 7.21 (dd, *J* = 5.3, 3.3 Hz, 2H), 7.12 (dd, *J* = 5.5, 3.2 Hz, 2H), 6.98–6.89 (m, 6H), 3.83 (s, 9H), 3.54–3.36 (m, 3H), 3.31–3.20 (dd, *J* = 14.7, 8.2 Hz, 2H). ^{13}C NMR (101 MHz, $CDCl_3$) δ 186.5 (d, *J* = 10.0 Hz), 162.6 (d, *J* = 2.9 Hz), 143.8, 135.0 (d, *J* = 11.2 Hz), 126.1, 124.4, 120.2 (d, *J* = 105.9 Hz), 114.3 (d, *J* = 13.2 Hz), 55.5, 49.7 (d, *J* = 18.4 Hz), 38.0 (d, *J* = 1.4 Hz). ^{31}P NMR (162 MHz, $CDCl_3$) δ 17.69 (m). FTIR (neat), cm^{-1}: 1568 (m), 1498 (w), 1333 (m), 1253 (s), 1179 (m), 1110 (s), 1026 (m), 802 (m), 534 (s). HRMS (ESI): Calcd for $[C_{31}H_{30}NO_4PNa]^+$: 534.18047, found: 534.18042.

2,2,2-Trifluoroethyl (tris(4-methoxyphenyl)-λ^5-phosphaneylidene)carbamate (**75**)

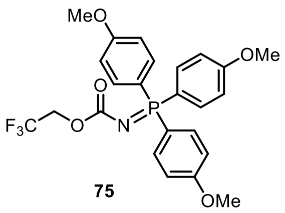

75

Compound **75** was isolated as a side-product formed in the catalytic reactions. Colorless oil. ^{1}H NMR (600 MHz, $CDCl_3$) δ 7.70–7.57 (m, 6H), 7.02–6.90 (m, 6H), 4.41 (q, *J* = 8.9 Hz, 2H), 3.84 (s, 9H). $^{13}C\{^{19}F\}$ NMR (151 MHz, $CDCl_3$) δ 163.0 (d, *J* = 2.9 Hz), 159.9 (d, *J* = 2.7 Hz), 135.0 (d, *J* = 11.4 Hz), 123.9, 119.1 (d, *J* = 109.4 Hz), 114.5 (d, *J* = 13.6 Hz), 61.8 (d, *J* = 4.2 Hz), 55.6. $^{19}F\{^1H\}$ NMR (564 MHz, $CDCl_3$) δ −73.75. ^{31}P NMR (242 MHz, $CDCl_3$) δ 21.46 (m). FTIR (neat), cm^{-1}: 2924 (w), 1650 (m), 1593 (s), 1500 (s), 1250 (vs), 1101 (vs), 1022 (s), 827 (m), 801 (m), 530 (s). HRMS (ESI): Calcd for $[C_{24}H_{23}NO_5PF_3Na]^+$: 516.11582, found: 516.11584.

Optimization of reaction conditions

Unless specified otherwise, all reactions were performed using 4-(trifluoromethyl)styrene (0.10 mmol, 1 equiv) or *tert*-butyl crotonate (0.10 mmol, 1 equiv) in screw-cap Schlenk tubes. The reported yields represent yields determined by a ^{1}H NMR analysis of crude reaction mixtures using 1,3,5-trimethoxybenzene (0.033 mmol, 1/3 equiv) as an internal standard added to the mixture prior to the concentration of the mixture under reduced pressure.

Table S1 Control experiments.

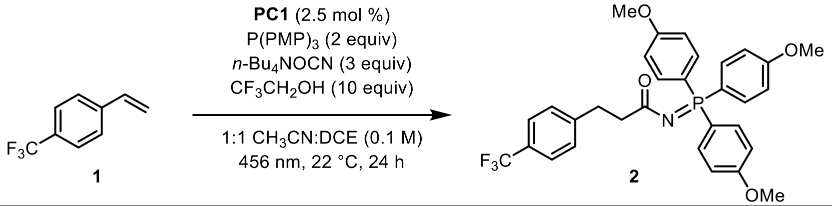

modification	NMR yield (%)
none	94
without **PC1**	0
without CF_3CH_2OH	75
with $(CF_3)_2CHOH$ instead of CF_3CH_2OH	69
in dark	0
under air	54
with TRIP-SH (10 mol %)	93

Table S2 Screening of phosphorus(III) reagents.

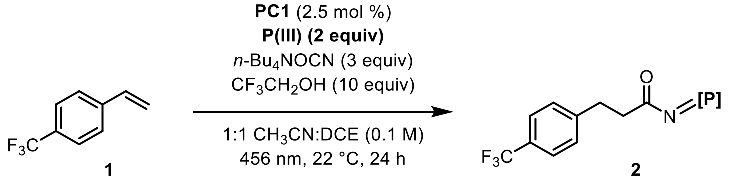

P(III) reagent	NMR yield (%)
$P(4\text{-}OMeC_6H_4)_3$	94
$P(4\text{-}MeC_6H_4)_3$	98
$P(3\text{-}MeC_6H_4)_3$	91
$P(2\text{-}MeC_6H_4)_3$	51
$P(4\text{-}FC_6H_4)_3$	59
PPh_3	83
$P(4\text{-}CF_3C_6H_4)_3$	0
PCy_3	89
Ph_2POEt	78[a]

[a] The determined yield corresponds to a 3:1 mixture of the expected product and the product of hydrolysis of the ethoxy group, respectively.

Table S3 Screening of photoredox catalysts (PCs).

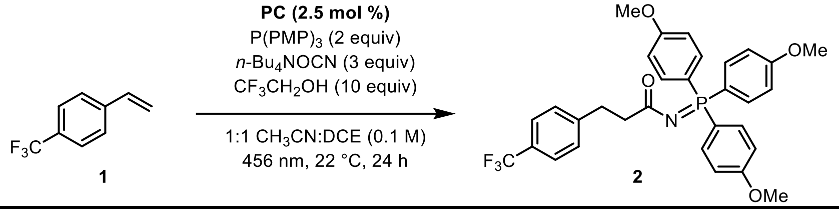

photoredox catalyst	NMR yield (%)
[Ir(dF(CF$_3$)ppy)$_2$(dtbbpy)]PF$_6$ (**PC1**)	94
[Ir(Fmppy)$_2$(dtbbpy)]PF$_6$ (**PC3**)	98
[Ir(dF(CH$_3$)ppy)$_2$(dtbbpy)]PF$_6$ (**PC4**)	98
[Ir(dF(CF$_3$)ppy)$_2$(5,5'-dCF3-bpy)]PF$_6$ (**PC5**)	94
4-CzIPN (**PC2**)	63
4-CzIPN (**PC2**) (5 mol %)	79
4-CzIPN (**PC2**) (10 mol %)	81
Mes-Acr-Ph-BF$_4$ (**PC6**)	0

Table S4 Screening of cyanate sources.

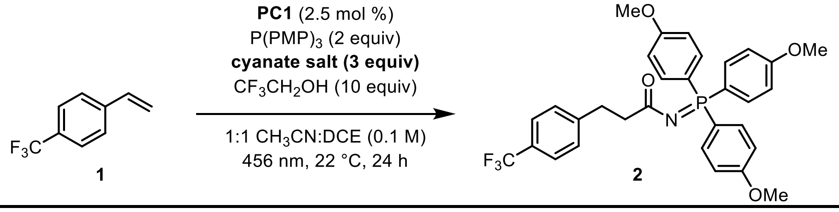

cyanate salt (+ additive)	NMR yield (%)
n-Bu$_4$NOCN	94
KOCN	48
NaOCN	36
KOCN + 18-crown-6	91
KOCN + *n*-Bu$_4$NBF$_4$	25
KOCN + *n*-Bu$_4$NCl	45
NaOCN + *n*-Bu$_4$NCl	96
NaOCN + *n*-Bu$_4$NCl (1 equiv)	56
NaOCN + *n*-Bu$_4$NCl (0.2 equiv)	39
NaOCN + *n*-Bu$_4$NBr	<5

Table S5 Screening of solvents.

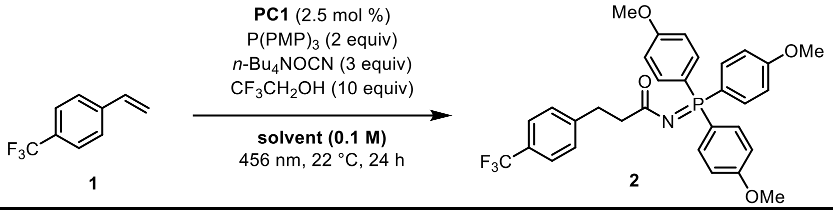

solvent	NMR yield (%)
1:1 CH_3CN:DCE	91
CH_3CN	85
DCE	82
EtOAc	81
$PhCH_3$	53
DMF	83

Table S6 Evaluation of different wavelengths of light.

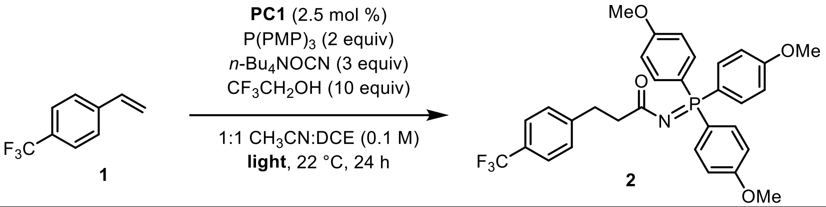

light	NMR yield (%)
456 nm Kessil lamp	94
405 nm Kessil lamp	94
390 nm EvoluChem LED lamp	98

Table S7 Evaluation of different reaction concentrations.

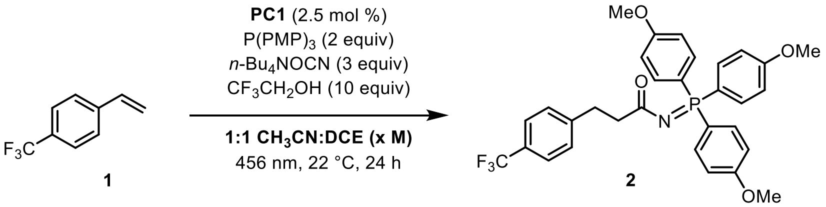

concentration (M)	NMR yield (%)
0.20	93
0.10	94
0.05	96

Table S8 Evaluation of phosphine and cyanate loadings.

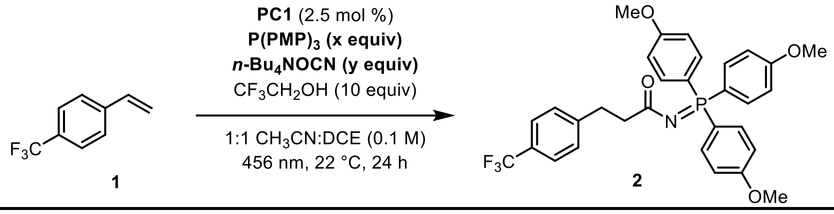

x (equiv)	y (equiv)	NMR yield (%)
1.2	1.5	63
1.5	2.0	75
2.0	3.0	94

Table S9 Evaluation of phosphine loading for three different substrates.[a]

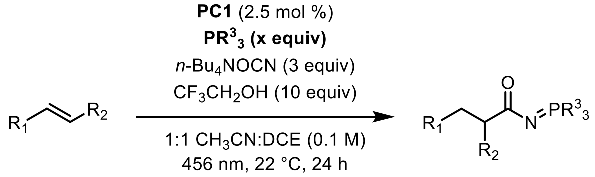

product	**2**	**12**	**31**
x (equiv)	NMR yield (%)		
1.0	53	25	27
2.0	88	39	56

[a] Reactions were performed on a 0.20 mmol scale.

Table S10 Screening of phosphorus(III) reagents.

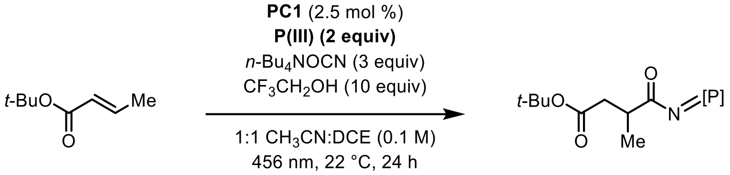

P(III) reagent	NMR yield (%)
$P(4\text{-}OMeC_6H_4)_3$	24
$P(4\text{-}MeC_6H_4)_3$	24
$P(4\text{-}FC_6H_4)_3$	<5
PPh_3	12
$P(4\text{-}CF_3C_6H_4)_3$	0
PPh_2Cy	24
$PPhCy_2$	42
PCy_3	63
Ph_2POEt	<5

Cascade reactions and derivatizations of products

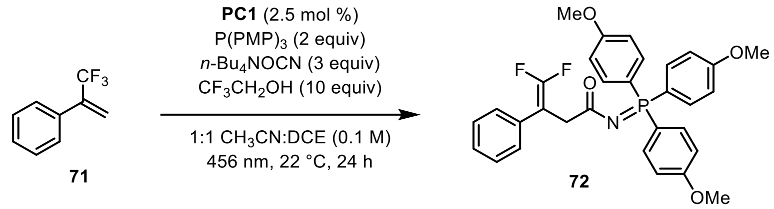

4,4-Difluoro-3-phenyl-*N*-(tris(4-methoxyphenyl)-λ^5-phosphaneylidene)but-3-enamide (**72**)
N-Acyl iminophosphorane **72** was prepared according to an analogous procedure as described for the preparation of **2** on page S4 using α-(trifluoromethyl)styrene (**71**, 44 µL, 0.30 mmol, 1 equiv). The product was purified by FCC (20–70% ethyl acetate in pentane) and isolated as a white amorphous solid (138 mg, 84%). ^{1}H NMR (500 MHz, $CDCl_3$) δ 7.55–7.44 (m, 8H), 7.32–7.26 (m, 2H), 7.24–7.19 (m, 1H), 6.92–6.85 (m, 6H), 3.82 (s, 9H), 3.51 (app q, *J* = 2.1 Hz, 2H). ^{13}C{^{19}F} NMR (126 MHz, $CDCl_3$) δ 180.7 (d, *J* = 9.8 Hz), 162.6 (d, *J* = 2.9 Hz), 154.8, 134.9(4) (d, *J* = 11.4 Hz), 134.9(1), 128.5, 128.2, 126.8, 119.8 (d, *J* = 106.1 Hz), 114.3 (d, *J* = 13.3 Hz), 90.4 (d, *J* = 1.6 Hz), 55.5, 39.6 (d, *J* = 20.6 Hz). ^{19}F NMR (283 MHz, $CDCl_3$) δ −89.70 (dt, *J* = 40.1, 2.4 Hz), −91.94 (d, *J* = 40.1 Hz). ^{31}P NMR (202 MHz, $CDCl_3$) δ 17.97 (m). FTIR (neat), cm^{-1}: 1716 (m), 1586 (s), 1499 (m), 1335 (m), 1254 (s), 1176 (m), 1109 (vs), 1016 (m), 822 (s), 801 (s), 526 (s). HRMS (ESI): Calcd for $[C_{31}H_{28}NO_4PF_2Na]^+$: 570.16162, found: 570.16131.

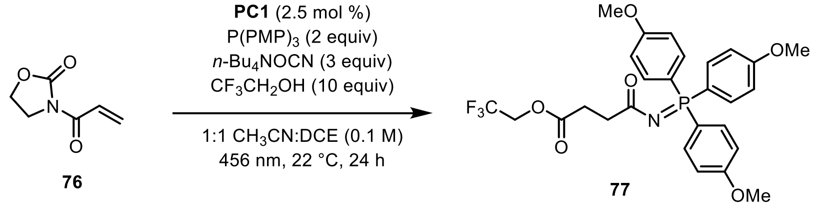

2,2,2-Trifluoroethyl 4-oxo-4-((tris(4-methoxyphenyl)-λ^5-phosphaneylidene)amino)butanoate (**77**)
N-Acyl iminophosphorane **77** was prepared according to an analogous procedure as described for the preparation of **2** on page S4 using 3-acryloyloxazolidin-2-one (**76**, 42 mg, 0.30 mmol, 1 equiv). The product was purified by FCC (40–100% ethyl acetate in pentane) and isolated as a colorless oil (116 mg, 71%). ^{1}H NMR (400 MHz, $CDCl_3$) δ 7.69–7.56 (m, 6H), 7.00–6.88 (m, 6H), 4.39 (q, *J* = 8.6 Hz, 2H), 3.83 (s, 9H), 2.86–2.71 (m, 4H). ^{13}C NMR (101 MHz, $CDCl_3$) δ 182.1 (d, *J* = 9.8 Hz), 172.5, 162.7 (d, *J* = 2.9 Hz), 135.0 (d, *J* = 11.4 Hz), 123.3 (q, *J* = 277.2 Hz), 119.8 (d, *J* = 106.2 Hz), 114.4 (d, *J* = 13.3 Hz), 60.3 (q, *J* = 36.4 Hz), 55.5, 34.9 (d, *J* = 20.0 Hz), 30.9 (d, *J* = 3.1 Hz). ^{19}F NMR (375 MHz, $CDCl_3$) δ −73.67. ^{31}P NMR (162 MHz, $CDCl_3$) δ 18.70 (m). FTIR (neat), cm^{-1}: 1755 (m), 1592 (s), 1500 (w), 1252 (s), 1177 (m), 1108 (s), 1022 (m), 826 (m), 801 (m), 662 (m), 529 (s). HRMS (ESI): Calcd for $[C_{27}H_{27}NO_6PF_3Na]^+$: 572.14203, found: 572.14182.

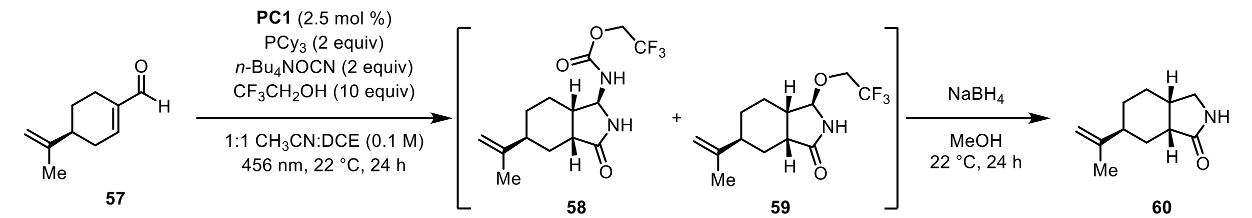

(3a*S*,6*S*,7a*R*)-6-(Prop-1-en-2-yl)octahydro-1*H*-isoindol-1-one (**60**)

A mixture of tetrabutylammonium cyanate (0.17 g, 0.60 mmol, 2.0 equiv) and iridium catalyst **PC1** (8.4 mg, 7.5 µmol, 0.025 equiv) in acetonitrile (1.5 mL) was added to a mixture of tricyclohexylphosphine (0.17 g, 0.60 mmol, 2.0 equiv) and (*S*)-(−)-perillaldehyde (**57**, 52 µL, 90% purity, 0.30 mmol, 1 equiv) in 1,2-dichloroethane (1.5 mL) in a Schlenk tube at ambient temperature under argon atmosphere. 2,2,2-Trifluoroethanol (0.22 mL, 3.0 mmol, 10 equiv) was added via syringe at ambient temperature. The reaction tube was placed in a photoreactor equipped with a 456 nm Kessil lamp. The reaction mixture was stirred at ambient temperature (22 °C) for 24 h. The reaction mixture was concentrated under reduced pressure. The crude product mixture was dissolved in methanol (2.0 mL), and sodium borohydride (113 mg, 3.0 mmol, 10 equiv) was added at ambient temperature. The resulting mixture was allowed to stir at ambient temperature (22 °C) for 24 h. An aqueous saturated solution of ammonium chloride (10 mL) was added, and the mixture was extracted with ethyl acetate (3 × 50 mL). The combined organic fractions were washed with brine (10 mL) and were dried over anhydrous magnesium sulfate. The dried solution was filtered, and the filtrate was concentrated under reduced pressure. The crude product was purified by FCC (80–100% ethyl acetate in pentane) to provide **60** as a white amorphous solid (26 mg, 49%). ^{1}H NMR (600 MHz, $CDCl_3$) δ 6.18 (br s, 1H), 4.71–4.67 (m, 2H), 3.42 (dd, *J* = 9.5, 5.3 Hz, 1H), 2.90 (dd, *J* = 9.5, 1.6 Hz, 1H), 2.57 (app td, *J* = 6.8, 1.6 Hz, 1H), 2.35 (app dq, *J* = 12.1, 6.1 Hz, 1H), 2.27 (app ddt, *J* = 13.7, 3.9, 2.2 Hz, 1H), 1.87–1.77 (m, 2H), 1.73 (s, 3H), 1.72–1.67 (m, 1H), 1.41–1.31 (m, 2H), 1.12 (app qd, *J* = 13.3, 3.1 Hz, 1H). ^{13}C NMR (151 MHz, $CDCl_3$) δ 179.0, 150.0, 108.7, 46.7, 41.6, 41.4, 34.8, 29.6, 28.9, 28.2, 21.3. FTIR (neat), cm^{-1}: 3228 (br), 2937 (m), 2853 (w), 1691 (s), 1434 (m), 1273 (m), 741 (s). HRMS (ESI): Calcd for $[C_{11}H_{17}NONa]^+$: 202.12024, found: 202.12018.

In an independent experiment, products **58** and **59** were isolated and fully characterized. **58**: White amorphous solid. ^{1}H NMR (600 MHz, $CDCl_3$) δ 6.42 (br s, 1H), 5.83 (br d, *J* = 7.2 Hz, 1H), 4.82 (d, *J* = 7.1 Hz, 1H), 4.74–4.70 (m, 1H), 4.70–4.66 (m, 1H), 4.53–4.47 (m, 1H), 4.47–4.39 (m, 1H), 2.85–2.76 (m, 1H), 2.35–2.23 (m, 2H), 2.10–2.00 (m, 1H), 1.81–1.70 (m, 5H), 1.37 (app td, *J* = 13.1, 6.3 Hz, 1H), 1.33–1.20 (m, 1H), 1.20–1.07 (m, 1H). ^{13}C NMR (151 MHz, $CDCl_3$) δ 178.0, 154.0, 149.3, 123.1 (q, *J* = 277.4 Hz), 109.1, 66.3, 61.2 (q, *J* = 36.7 Hz), 41.2, 41.0, 38.8, 28.9, 28.2, 27.7, 21.2. ^{19}F{^{1}H} NMR (564 MHz, $CDCl_3$) δ −74.25. FTIR (neat), cm^{-1}: 3385 (w), 2945 (w), 1738 (m), 1689 (s), 1542 (s), 1436 (w), 1240 (m), 1148 (s), 1042 (s), 974 (m), 635 (s). HRMS (ESI): Calcd for $[C_{14}H_{19}F_3N_2O_3Na]^+$: 343.12400, found: 343.12399. **59**: White amorphous solid. ^{1}H NMR (600 MHz, $CDCl_3$) δ 7.58 (br s, 1H), 4.72–4.70 (m, 1H), 4.70–4.67 (m, 1H), 4.60–4.57 (m, 1H), 3.90–3.82 (dq, *J* = 12.3, 8.8 Hz, 1H), 3.82–3.75 (dq, *J* = 12.3, 8.5 Hz, 1H), 2.95–2.88 (m, 1H), 2.49–2.39 (m, 1H), 2.32–2.23 (m, 1H), 2.02–1.91 (m, 1H), 1.77–1.66 (m, 5H), 1.38 (dddd, *J* = 13.6, 12.5, 6.4, 0.9 Hz, 1H), 1.20–1.07 (m, 2H). ^{13}C NMR (151 MHz, $CDCl_3$) δ 180.5, 149.5, 123.9 (q, *J* = 278.8 Hz), 109.1, 90.1, 65.0 (q, *J* = 34.6 Hz), 41.5, 41.2, 38.5, 28.9, 27.5, 27.2, 21.1. ^{19}F{^{1}H} NMR (564 MHz, $CDCl_3$) δ −74.01. FTIR (neat), cm^{-1}: 3233 (br), 2923 (w), 2860 (w), 1715 (s), 1674 (s), 1440 (m), 1280 (s), 1151 (s), 1113 (s), 977 (s), 898 (m), 739 (m). HRMS (ESI): Calcd for $[C_{13}H_{18}F_3NO_2Na]^+$: 300.11818, found: 300.11813.

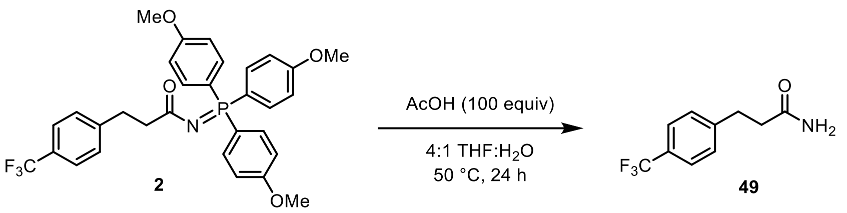

3-(4-(Trifluoromethyl)phenyl)propenamide (**49**)

N-Acyl iminophosphorane **2** (57 mg, 0.10 mmol, 1 equiv) was dissolved in THF (1.2 mL), and water (0.3 mL) was added at ambient temperature. Acetic acid (0.57 mL, 10 mmol, 100 equiv) was added, and the resulting mixture was heated to 50 °C. After stirring the reaction mixture at 50 °C for 24 h, the mixture was allowed to cool down to ambient temperature and was concentrated under reduced pressure. The crude product mixture was purified by FCC (20–60% acetone in pentane) to provide amide **49** as a white amorphous solid (22 mg, 100%). The spectroscopic data of **49** were in accordance with the data reported in the literature.[47] ^{1}H NMR (400 MHz, $CDCl_3$) δ 7.54 (d, *J* = 8.0 Hz, 2H), 7.32 (d, *J* = 7.8 Hz, 2H), 5.60 (br s, 1H), 5.40 (br s, 1H), 3.03 (t, *J* = 7.6 Hz, 2H), 2.54 (t, *J* = 7.6 Hz, 2H). ^{13}C NMR (101 MHz, $CDCl_3$) δ 174.0, 145.0, 128.9, 128.9 (q, *J* = 32.4 Hz), 125.7 (q, *J* = 3.8 Hz), 124.4 (q, *J* = 271.7 Hz), 37.1, 31.2. ^{19}F{^{1}H} NMR (376 MHz, $CDCl_3$) δ −62.42. FTIR (neat), cm^{-1}: 3400 (br), 3200 (br), 1651 (s), 1615 (m), 1418 (w), 1319 (s), 1170 (m), 1120 (s), 1065 (m), 837 (m), 635 (m). HRMS (ESI): Calcd for $[C_{10}H_9NOF_3]^-$: 216.06308, found: 216.06411.

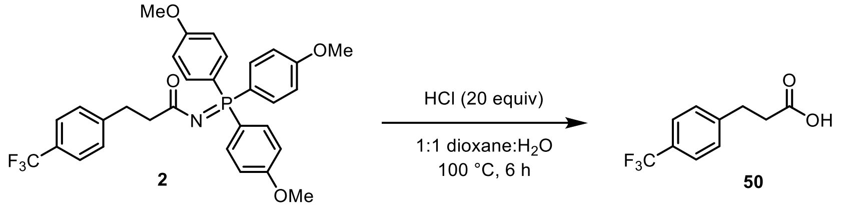

3-(4-(Trifluoromethyl)phenyl)propanoic acid (**50**)

N-Acyl iminophosphorane **2** (57 mg, 0.10 mmol, 1 equiv) was dissolved in dioxane (1.0 mL). Hydrochloric acid (1.0 mL, 2.0 mmol, 20 equiv) was added, and the resulting mixture was heated to 100 °C. After stirring the reaction mixture at 100 °C for 6 h, the mixture was allowed to cool down to ambient temperature and was diluted with ethyl acetate (50 mL). The diluted mixture was washed with brine (5 mL), and the aqueous layer was extracted with ethyl acetate (3 × 50 mL). The combined fractions were dried over anhydrous magnesium sulfate, the dried solution was filtered, and the filtrate was concentrated under reduced pressure. The crude product mixture was purified by FCC (50–100% ethyl acetate in pentane followed by 2% acetic acid in ethyl acetate) to provide carboxylic acid **50** as a white amorphous solid (22 mg, 99%). The spectroscopic data of **50** were in accordance with the data reported in the literature.[48] ^{1}H NMR (300 MHz, $CDCl_3$) δ 7.55 (d, *J* = 8.0 Hz, 2H), 7.33 (d, *J* = 8.0 Hz, 2H), 3.02 (t, *J* = 7.6 Hz, 2H), 2.71 (t, *J* = 7.6 Hz, 2H). FTIR (neat), cm^{-1}: 1703 (s), 1320 (s), 1164 (m), 1119 (s), 1065 (m), 831 (s), 681 (m). HRMS (ESI): Calcd for $[C_{10}H_8O_2F_3]^-$: 217.04819, found: 217.04800.

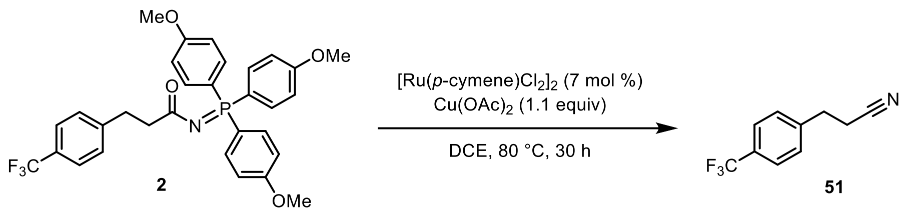

3-(4-(Trifluoromethyl)phenyl)propanenitrile (**51**)

Dichloro(*p*-cymene)ruthenium(II) dimer (6.4 mg, 11 µmol, 0.070 equiv) and copper(II) acetate (30 mg, 0.17 mmol, 1.1 equiv) were added to a solution of *N*-acyl iminophosphorane **2** (85 mg, 0.15 mmol, 1 equiv) in DCE (1.0 mL) at ambient temperature. The resulting mixture was heated to 80 °C and was stirred at this temperature for 30 h. The mixture was allowed to cool down to ambient temperature and was diluted with ethyl acetate (50 mL). The solution was washed with brine (10 mL), and the aqueous phase was extracted with ethyl acetate (3 × 50 mL). The combined organic fractions were dried over anhydrous magnesium sulfate, the dried solution was filtered, and the filtrate was concentrated under reduced pressure. The crude product mixture was purified by FCC (15% ethyl acetate in pentane) to provide nitrile **51** as a colorless oil (22 mg, 73%). The spectroscopic data of **51** were in accordance with the data reported in the literature.[49] ^{1}H NMR (500 MHz, $CDCl_3$) δ 7.61 (d, *J* = 8.0 Hz, 2H), 7.37 (d, *J* = 7.9 Hz, 2H), 3.03 (t, *J* = 7.3 Hz, 2H), 2.66 (t, *J* = 7.3 Hz, 2H). ^{13}C{^{19}F} NMR (126 MHz, $CDCl_3$) δ 142.1, 130.0, 128.9, 126.1, 124.2, 118.7, 31.5, 19.3. ^{19}F{^{1}H} NMR (470 MHz, $CDCl_3$) δ −62.62. FTIR (neat), cm^{-1}: 1322 (s), 1162 (m), 1108 (s), 1065 (s), 841 (m). HRMS (EI): Calcd for [$C_{10}H_8NF_3$]: 199.06034, found: 199.06027.

(3*S*,8*S*,9*S*,10*R*,13*R*,14*S*,17*R*)-10,13-Dimethyl-17-((*R*)-6-methylheptan-2-yl)-2,3,4,7,8,9,10,11,12,13,14,15,16,17-tetradecahydro-1*H*-cyclopenta[*a*]phenanthren-3-yl 3-cyanopropanoate (**52**)

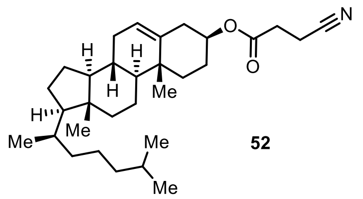

Nitrile **52** was prepared according to an analogous procedure as described for the preparation of **51** on page S29 starting from *N*-acyl iminophosphorane **46** (84 mg, 0.10 mmol, 1 equiv). The product was purified by FCC (5–20% ethyl acetate in pentane) and isolated as a white amorphous solid (35 mg, 75%). ^{1}H NMR (300 MHz, $CDCl_3$) δ 5.45–5.32 (m, 1H), 4.81–4.57 (m, 1H), 2.73–2.55 (m, 4H), 2.39–2.26 (m, 2H), 2.09–1.91 (m, 2H), 1.91–1.73 (m, 3H), 1.73–0.94 (m, 24H), 0.91 (d, *J* = 6.5 Hz, 3H), 0.88–0.82 (m, 6H), 0.67 (s, 3H). ^{13}C NMR (101 MHz, $CDCl_3$) δ 169.6, 139.4, 123.2, 118.7, 75.5, 56.8, 56.3, 50.2, 42.5, 39.9, 39.7, 38.2, 37.1, 36.7, 36.4, 36.0, 32.1, 32.0, 30.4, 28.4, 28.2, 27.9, 24.4, 24.0, 23.0, 22.7, 21.2, 19.5, 18.9, 13.2, 12.0. FTIR (neat), cm^{-1}: 2946 (s), 2869 (s), 1736 (s), 1726 (s), 1468 (w), 1376 (m), 1288 (m), 1205 (s), 1186 (s). HRMS (ESI): Calcd for [$C_{31}H_{49}NO_2Na$]$^+$: 490.36555, found: 490.36558.

Tert-butyl 3-cyano-3-(2-ethoxy-2-oxoethyl)azetidine-1-carboxylate (**53**)

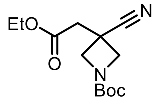

53

Nitrile **53** was prepared according to an analogous procedure as described for the preparation of **51** on page S29 starting from *N*-acyl iminophosphorane **34** (57 mg, 0.10 mmol, 1 equiv). The product was purified by FCC (50–70% ethyl acetate in pentane) and isolated as a colorless oil (8.8 mg, 33%). Unreacted *N*-acyl iminophosphorane **34** (38 mg, 67%) could be recovered from the reaction mixture. ^{1}H NMR (400 MHz, $CDCl_3$) δ 4.38–4.29 (m, 2H), 4.22 (q, *J* = 7.1 Hz, 2H), 3.97–3.88 (m, 2H), 2.99–2.90 (m,

2H), 1.44 (s, 9H), 1.29 (t, *J* = 7.1 Hz, 3H). ^{13}C NMR (101 MHz, $CDCl_3$) δ 168.5, 155.6, 120.9, 81.1, 61.9, 58.1, 40.7, 29.9, 28.4, 27.8, 14.3. FTIR (neat), cm^{-1}: 1732 (s), 1704 (vs), 1692 (vs), 1387 (s), 1227 (s), 1205 (s), 1030 (m), 856 (m), 771 (m). HRMS (ESI): Calcd for $[C_{13}H_{20}N_2O_4Na]^+$: 291.13153, found: 291.13143.

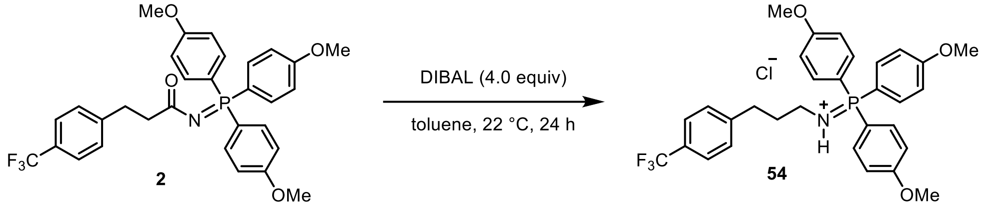

3-(4-(Trifluoromethyl)phenyl)-*N*-(tris(4-methoxyphenyl)-λ^5-phosphaneylidene)propan-1-aminium chloride (**54**)

A solution of diisobutylaluminum hydride (DIBAL, 1.2 M in toluene, 0.50 mL, 0.60 mmol, 4.0 equiv) was added to a solution of *N*-acyl iminophosphorane **2** (85 mg, 0.15 mmol, 1 equiv) in toluene (2.5 mL) at ambient temperature. The resulting mixture was stirred at ambient temperature (22 °C) for 24 h. A saturated aqueous solution of ammonium chloride (0.5 mL) was added dropwise. Anhydrous sodium sulfate was added, and the resulting suspension was filtered through a plug of Celite (0.5 cm) eluting with ethyl acetate. The filtrate was concentrated under reduced pressure. The crude product mixture was purified by FCC (2–10% methanol in dichloromethane) to provide iminophosphorane–HCl salt **54** as a yellow amorphous solid (77 mg, 87%). ^{1}H NMR (500 MHz, $CDCl_3$) δ 8.50 (q, *J* = 7.0 Hz, 1H), 7.71–7.60 (m, 6H), 7.40 (d, *J* = 7.9 Hz, 2H), 7.13 (d, *J* = 7.9 Hz, 2H), 7.07–6.99 (m, 6H), 3.86 (s, 9H), 3.00–2.90 (m, 2H), 2.66 (t, *J* = 7.4 Hz, 2H), 2.00 (app p, *J* = 7.3 Hz, 2H). ^{13}C{^{19}F} NMR (126 MHz, $CDCl_3$) δ 164.4 (d, *J* = 2.9 Hz), 145.9, 135.6 (d, *J* = 12.5 Hz), 128.9, 128.1, 125.2, 124.5, 115.5 (d, *J* = 14.2 Hz), 113.2 (d, *J* = 110.9 Hz), 55.8, 41.6, 32.7, 32.1 (d, *J* = 6.7 Hz). ^{19}F{^{1}H} NMR (470 MHz, $CDCl_3$) δ −62.27. ^{31}P NMR (202 MHz, $CDCl_3$) δ 36.08 (m). FTIR (neat), cm^{-1}: 2936 (br), 1592 (s), 1502 (m), 1324 (m), 1260 (s), 1108 (s), 1016 (s), 829 (m), 803 (m), 530 (s). HRMS (ESI): Calcd for $[C_{31}H_{31}NO_3PF_3H]^+$: 554.20664, found: 554.20643.

N-(Tricyclohexyl-λ^5-phosphaneylidene)-3-(4-(trifluoromethyl)phenyl)propan-1-aminium chloride (**55**)

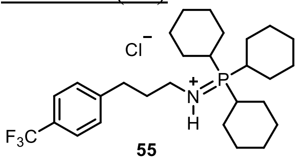

Iminophosphorane–HCl salt **55** was prepared according to an analogous procedure as described for the preparation of **54** on page S30 starting from *N*-acyl iminophosphorane **8** (74 mg, 0.15 mmol, 1 equiv). The product was purified by FCC (2–10% methanol in dichloromethane) and isolated as a white semi-solid (63 mg, 81%). ^{1}H NMR (600 MHz, $CDCl_3$) δ 7.52 (d, *J* = 8.1 Hz, 2H), 7.36 (d, *J* = 8.0 Hz, 2H), 7.25–7.18 (m, 1H), 2.90 (app p, *J* = 6.5 Hz, 2H), 2.82 (t, *J* = 7.5 Hz, 2H), 2.51–2.39 (m, 3H), 2.16 (app p, *J* = 7.4 Hz, 2H), 1.97–1.85 (m, 12H), 1.81–1.75 (m, 3H), 1.56–1.43 (m, 6H), 1.37–1.18 (m, 9H). ^{13}C{^{19}F} NMR (151 MHz, $CDCl_3$) δ 145.8, 129.1, 128.5, 125.5, 124.5, 42.1 (d, *J* = 4.6 Hz), 32.9, 32.7 (d, *J* = 6.8 Hz), 32.2 (d, *J* = 52.6 Hz), 26.8 (d, *J* = 12.4 Hz), 26.7 (d, *J* = 3.1 Hz), 25.8 (d, *J* = 1.6 Hz). ^{19}F{^{1}H} NMR (564 MHz, $CDCl_3$) δ −62.36. ^{31}P NMR (242 MHz, $CDCl_3$) δ 53.96 (m). FTIR (neat), cm^{-1}: 3032 (br), 2939 (m), 2855 (m), 2161 (w), 1447 (w), 1325 (s), 1114 (s), 1067 (m), 927 (m), 721 (s). HRMS (ESI): Calcd for $[C_{28}H_{43}NPF_3H]^+$: 482.31580, found: 482.31498.

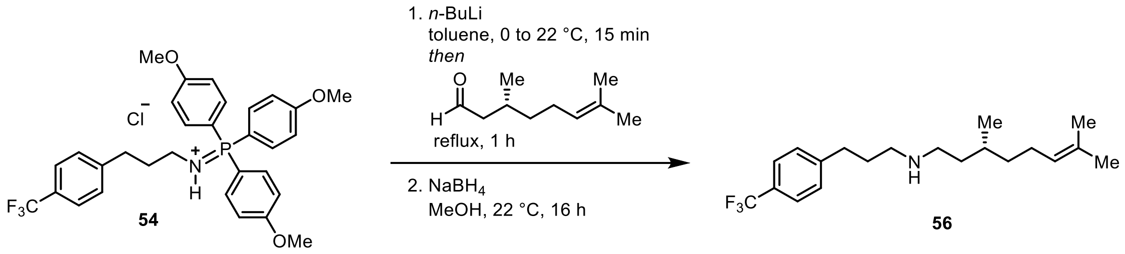

<u>(*R*)-3,7-Dimethyl-*N*-(3-(4-(trifluoromethyl)phenyl)propyl)oct-6-en-1-amine (**56**)</u>

n-Butyllithium solution (1.6 M in hexanes, 63 µL, 0.10 mmol, 1.0 equiv) was added to a solution of iminophosphorane–HCl salt **54** (59 mg, 0.10 mmol, 1 equiv) in toluene (1.0 mL) at 0 °C. After stirring the mixture at 0 °C for 5 min, the mixture was allowed to warm up to ambient temperature (22 °C) and was stirred for additional 10 minutes at 22 °C. (*R*)-(+)-Citronellal (27 µL, 0.15 mmol, 1.5 equiv) was added at 22 °C, and the resulting mixture was heated to reflux for 1 h. The reaction mixture was allowed to cool down to ambient temperature and was concentrated under reduced pressure. The residue was dissolved in methanol (1.0 mL), and sodium borohydride (38 mg, 1.0 mmol, 10 equiv) was added at 22 °C. After stirring for 16 h, the mixture was concentrated. The crude product mixture was purified by FCC (0.2–0.5% Et_3N in 2–5% methanol in dichloromethane) to provide amine **56** as a colorless oil (21 mg, 61%). ^1H NMR (500 MHz, $CDCl_3$) δ 7.53 (d, *J* = 8.1 Hz, 2H), 7.29 (d, *J* = 8.0 Hz, 2H), 5.11–5.05 (m, 1H), 2.75–2.69 (m, 2H), 2.69–2.57 (m, 4H), 2.06–1.91 (m, 2H), 1.91–1.81 (m, 2H), 1.70–1.66 (m, 3H), 1.59 (s, 3H), 1.58–1.41 (m, 2H), 1.38–1.27 (m, 2H), 1.21–1.11 (m, 1H), 0.88 (d, *J* = 6.6 Hz, 3H). $^{13}C\{^{19}F\}$ NMR (126 MHz, $CDCl_3$) δ 146.3, 131.4, 128.9, 128.4, 125.5, 124.9, 124.5, 49.4, 48.0, 37.3, 37.1, 33.7, 31.3, 30.8, 25.9, 25.7, 19.8, 17.8. $^{19}F\{^1H\}$ NMR (470 MHz, $CDCl_3$) δ −62.33. FTIR (neat), cm^{-1}: 2917 (m), 1323 (s), 1162 (m), 1120 (s), 1066 (m), 844 (m). HRMS (ESI): Calcd for $[C_{20}H_{30}NF_3H]^+$: 342.24031, found: 342.23915.

Synthesis and characterization of substrates

Substrate synthesis

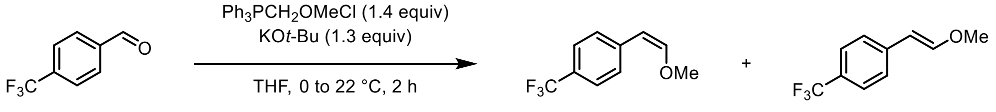

Z-**78** E-**78**

(*Z*)-1-(2-Methoxyvinyl)-4-(trifluoromethyl)benzene (***Z*-78**) and (*E*)-1-(2-methoxyvinyl)-4-(trifluoromethyl)benzene (***E*-78**)

4-Trifluoromethylbenzaldehyde (0.68 mL, 5.0 mmol, 1 equiv) was added to a stirred suspension of (methoxymethyl)triphenylphosphonium chloride (2.4 g, 7.0 mmol, 1.4 equiv) in THF (40 mL) at 0 °C. Potassium *tert*-butoxide (0.73 g, 6.5 mmol, 1.3 equiv) was added at 0 °C, and the resulting mixture was allowed to warm up to ambient temperature and was stirred for 1 h. Brine (50 mL) was added, and the mixture was extracted with diethyl ether (3 × 70 mL). The combined organic layers were dried over anhydrous magnesium sulphate, the dried solution was filtered, and the filtrate was concentrated under reduced pressure. The crude product mixture was purified by FCC (pentane) to yield *Z*-**78** as a colorless oil (109 mg, 11% yield) and *E*-**78** as a white amorphous solid (103 mg, 10% yield). The spectroscopic data of *Z*-**78** and *E*-**78** were in accordance with the data reported in the literature.[50] *Z*-**78**: ^{1}H NMR (500 MHz, $CDCl_3$) δ 7.65 (d, J = 8.2 Hz, 2H), 7.51 (d, J = 7.8 Hz, 2H), 6.24 (d, J = 7.0 Hz, 1H), 5.25 (d, J = 7.0 Hz, 1H), 3.82 (s, 3H). ^{13}C{^{19}F} NMR (126 MHz, $CDCl_3$) δ 150.0, 139.6, 128.3, 127.5, 124.6, 125.2, 104.7, 61.2. ^{19}F{^{1}H} NMR (470 MHz, $CDCl_3$) δ −62.34. *E*-**78**: ^{1}H NMR (400 MHz, $CDCl_3$) δ 7.50 (d, J = 8.1 Hz, 2H), 7.30 (d, J = 8.1 Hz, 2H), 7.13 (d, J = 13.0 Hz, 1H), 5.82 (d, J = 13.0 Hz, 1H), 3.72 (s, 3H). ^{13}C{^{19}F} NMR (126 MHz, $CDCl_3$) δ 150.8, 140.4, 127.7, 125.7, 125.2, 124.6, 104.2, 56.9. ^{19}F{^{1}H} NMR (283 MHz, $CDCl_3$) δ −62.26.

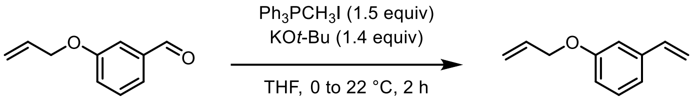

79

1-(Allyloxy)-3-vinylbenzene (**79**)

Potassium *tert*-butoxide (0.79 g, 7.0 mmol, 1.4 equiv) was added to a stirred suspension of methyltriphenylphosphonium iodide (3.0 g, 7.5 mmol, 1.5 equiv) in THF (40 mL) at 0 °C. The resulting mixture was stirred at 0 °C for 30 min. 3-(Allyloxy)benzaldehyde (0.77 mL, 5.0 mmol, 1 equiv) was added at 0 °C, and the resulting mixture was allowed to warm up to ambient temperature (22 °C). The mixture was stirred at 22 °C for 2 h. A saturated aqueous solution of ammonium chloride (50 mL) was added, and the mixture was extracted with diethyl ether (3 × 70 mL). The combined organic fractions were dried over anhydrous magnesium sulfate, the dried solution was filtered, and the filtrate was concentrated under reduced pressure. The crude product mixture was purified by FCC (5–10% ethyl acetate in pentane) to provide alkene **79** as a colorless oil (806 mg, 100%). The spectroscopic data of **79** were in accordance with the data reported in the literature.[51] ^{1}H NMR (400 MHz, $CDCl_3$) δ 7.24 (app t, J = 7.9 Hz, 1H), 7.06–6.95 (m, 2H), 6.83 (ddd, J = 8.2, 2.6, 0.9 Hz, 1H), 6.69 (dd, J = 17.6, 10.9 Hz, 1H), 6.07 (ddt, J = 17.3, 10.5, 5.3 Hz, 1H), 5.74 (dd, J = 17.6, 0.9 Hz, 1H), 5.48–5.36 (app dq, J = 17.3, 1.6 Hz, 1H), 5.29 (app dq, J = 10.5, 1.5 Hz, 1H), 5.25 (dd, J = 10.9, 1.0 Hz, 1H), 4.56 (app dt, J = 5.3, 1.5 Hz, 2H).

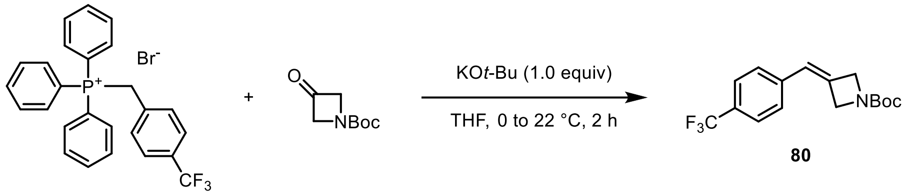

80

<u>*Tert*-butyl 3-(4-(trifluoromethyl)benzylidene)azetidine-1-carboxylate (**80**)</u>

Potassium *tert*-butoxide (0.17 g, 1.5 mmol, 1.0 equiv) was added to a stirred suspension of triphenyl[4-(trifluoromethyl)benzyl]phosphonium bromide (0.75 g, 1.5 mmol, 1 equiv) in THF (15 mL) at 0 °C. The resulting mixture was stirred at 0 °C for 5 min. 1-Boc-3-azetidinone (0.31 g, 1.8 mmol, 1.2 equiv) was added at 0 °C, and the resulting mixture was stirred at 0 °C for 30 min. The reaction mixture was allowed to warm up to ambient temperature (22 °C), and was stirred at this temperature for 1.5 h. A saturated aqueous solution of ammonium chloride (50 mL) was added, and the mixture was extracted with diethyl ether (3 × 50 mL). The combined organic fractions were washed with brine (10 mL) and were dried over anhydrous magnesium sulfate. The dried solution was filtered, and the filtrate was concentrated under reduced pressure. The crude product mixture was purified by FCC (10–20% ethyl acetate in pentane) to provide alkene **80** as a white amorphous solid (444 mg, 94%). ^{1}H NMR (600 MHz, $CDCl_3$) δ 7.57 (d, *J* = 7.9 Hz, 2H), 7.18 (d, *J* = 8.1 Hz, 2H), 6.33–6.26 (m, 1H), 4.88–4.80 (m, 2H), 4.68–4.62 (m, 2H), 1.47 (s, 9H). $^{13}C\{^{19}F\}$ NMR (151 MHz, $CDCl_3$) δ 156.4, 139.6, 134.2, 129.0, 127.4, 125.8, 124.2, 121.4, 80.2, 58.9, 58.3, 28.5. $^{19}F\{^1H\}$ NMR (564 MHz, $CDCl_3$) δ −62.60. FTIR (neat), cm^{-1}: 1680 (s), 1431 (m), 1323 (m), 1114 (s), 943 (m), 860 (m). HRMS (ESI): Calcd for $[C_{16}H_{18}NO_2F_3Na]^+$: 336.11818, found: 336.11811.

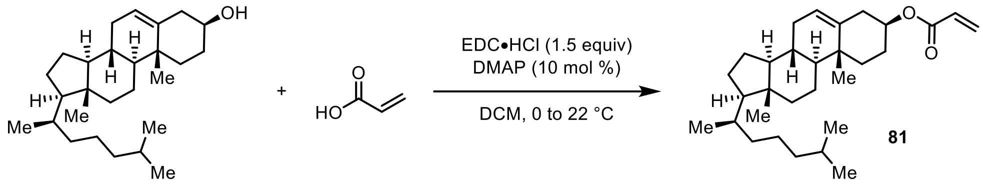

81

<u>(3*S*,8*S*,9*S*,10*R*,13*R*,14*S*,17*R*)-10,13-Dimethyl-17-((*R*)-6-methylheptan-2-yl)-2,3,4,7,8,9,10,11,12,13,14,15,16,17-tetradecahydro-1*H*-cyclopenta[*a*]phenanthren-3-yl acrylate (**81**)</u>

Alkene **81** was prepared according to a literature procedure.[52] *N*-(3-Dimethylaminopropyl)-*N*′-ethylcarbodiimide hydrochloride (EDC•HCl, 0.58 g, 3.0 mmol, 1.5 equiv), *N*,*N*-dimethylaminopyridine (DMAP, 24 mg, 0.20 mmol, 0.10 equiv), and cholesterol (0.77 g, 2.0 mmol, 1 equiv) were dissolved in DCM (12 mL) at 0 °C. Acrylic acid (0.15 mL, 2.2 mmol, 1.1 equiv) was added at 0 °C. After stirring the mixture for 30 min at 0 °C, the mixture was allowed to warm up to ambient temperature (22 °C) and was stirred at 22 °C for additional 17 h. A saturated aqueous solution of ammonium chloride (50 mL) was added, and the mixture was extracted with ethyl acetate (3 × 70 mL). The combined organic fractions were washed with a saturated aqueous solution of sodium bicarbonate (50 mL) and brine (20 mL). The organic layer was dried over anhydrous magnesium sulfate, the dried solution was filtered, and the filtrate was concentrated under reduced pressure. The crude product mixture was purified by FCC (5% ethyl acetate in pentane) to provide alkene **81** as a white amorphous solid (484 mg, 55%). The spectroscopic data were in accordance with the data reported in the literature.[52] ^{1}H NMR (400 MHz, $CDCl_3$) δ 6.39 (dd, *J* = 17.3, 1.6 Hz, 1H), 6.10 (dd, *J* = 17.3, 10.4 Hz, 1H), 5.80 (dd, *J* = 10.4, 1.6 Hz, 1H), 5.43–5.34 (m, 1H), 4.75–4.61 (m, 1H), 2.41–2.32 (m, 2H), 2.05–1.93 (m, 2H), 1.93–1.75 (m, 3H), 1.71–0.93 (m, 24H), 0.92 (d, *J* = 6.6 Hz, 3H), 0.87 (d, J = 6.6 Hz, 3H), 0.86 (d, J = 6.7 Hz, 3H), 0.68 (s, 3H).

Mechanistic experiments

Stern-Volmer quenching study

Stern-Volmer luminescence quenching analysis was conducted using a Jasco FP8550 spectrofluorometer. The following parameters were employed: excitation bandwidth = 5 nm, data interval = 0.5 nm, scan speed = 500 nm/min. The samples were measured in Hellma fluorescence QS quartz cuvettes (chamber volume = 1.4 mL, H × W × D = 46 mm × 12.5 mm × 12.5 mm) equipped with a polytetrafluoroethylene (PTFE) stopper. Samples were excited at λ_{ex} = 380 nm, and the emission was recorded at λ_{em} = 474 nm. A solution of **PC1** (5×10^{-6} M) was prepared by dissolving **PC1** (4.9 mg, 4.4 µmol) in DCE (8.7 mL) and diluting the resulting solution (0.30 mL) with DCE (13.7 mL), CH_3CN (14.0 mL), and TFE (2.0 mL). A solution of tetrabutylammonium cyanate (2.5×10^{-3} M) was prepared by dissolving tetrabutylammonium cyanate (9.7 mg, 0.034 mmol) in a mixture of DCE (6.4 mL), CH_3CN (6.4 mL), and TFE (0.91 mL). A solution of tris(4-methoxyphenyl)phosphine (2.5×10^{-3} M) was prepared by dissolving tris(4-methoxyphenyl)phosphine (8.5 mg, 0.024 mmol) in a mixture of DCE (4.5 mL), CH_3CN (4.5 mL), and TFE (0.64 mL). Samples were prepared by mixing solutions of **PC1** (300 µL) and solutions of the two different quenchers (0–700 µL) and a 7:7:1 mixture of DCE:CH_3CN:TFE to achieve a total volume of 1.00 mL (Table S11). Argon was bubbled through the sample for 50 s. Each measurement was repeated three times, and average values are reported (Table S11, Figure S1). I_0 represents the luminescence intensity in the absence of a quencher. I represents the luminescence intensity in the presence of a quencher.

Table S11 Results of the Stern-Vomer quenching study.

$V_{quencher}$ (µL)	$c_{quencher}$ (mM)	I[P(PMP)$_3$]	I_0/I[P(PMP)$_3$]	I(n-Bu$_4$NOCN)	I_0/I(n-Bu$_4$NOCN)
0	0	3061	1	3061	1
100	0.25	1689	1.81	3019	1.01
200	0.50	1137	2.69	2952	1.04
300	0.75	854	3.58	2975	1.03
400	1.00	680	4.50	2975	1.09
500	1.25	566	5.41	2960	1.03

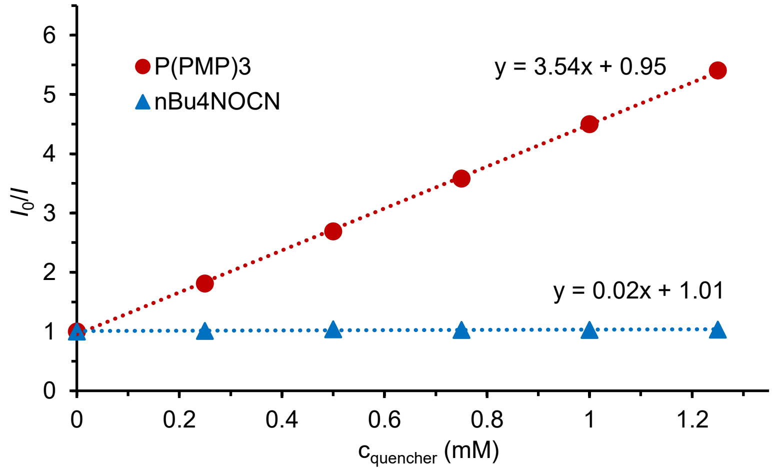

Figure S1 Graphical representation of the Stern-Vomer quenching study.

Evaluation of mechanistic pathways proceeding through P(V) intermediates

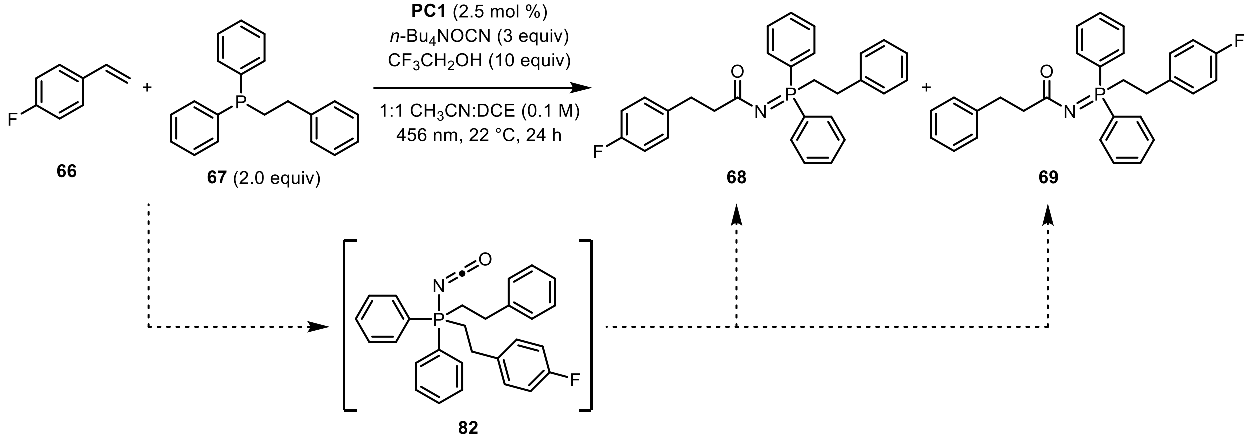

To evaluate whether a pathway proceeding through P(V) intermediates was operative, we performed the developed reaction using 4-fluorostyrene and diphenyl(2-phenylethyl)phosphine (**67**). If **82** represented a catalytically active intermediate that underwent a rearrangement to form the corresponding *N*-acyl iminophosphorane product, both products **68** and **69**, resulting from the migration of the 2-(4-fluorophenyl)ethyl and 2-phenylethyl substituents, respectively, were expected to be observed. *N*-Acyl iminophosphorane **68** was isolated in 23% yield. *N*-Acyl iminophosphorane **69** was not detected in the reaction mixture.

A mixture of tetrabutylammonium cyanate (0.17 g, 0.60 mmol, 3.0 equiv) and iridium catalyst **PC1** (5.6 mg, 5.0 µmol, 0.025 equiv) in acetonitrile (1.0 mL) was added to a mixture of diphenyl(2-phenylethyl)phosphine (**67**, 0.12 g, 0.40 mmol, 2.0 equiv) and 4-fluorostyrene (**66**, 24 µL, 0.20 mmol, 1 equiv) in 1,2-dichloroethane (1.0 mL) in a Schlenk tube at ambient temperature under argon atmosphere. 2,2,2-Trifluoroethanol (0.15 mL, 2.0 mmol, 10 equiv) was added via syringe at ambient temperature. The reaction tube was placed in a photoreactor equipped with a 456 nm Kessil lamp. The reaction mixture was stirred at ambient temperature (22 °C) for 24 h and then was concentrated under reduced pressure. The crude reaction mixture was analyzed by ^{1}H NMR and then was separated by FCC (40–100% ethyl acetate to pentane to 10% methanol in ethyl acetate). All fractions were analyzed by ^{1}H and ^{19}F NMR analyses, and selected fractions were additionally analyzed by HRMS. *N*-Acyl iminophosphorane **68** was isolated as a colorless oil (21 mg, 23%). ^{1}H NMR (600 MHz, $CDCl_3$) δ 7.76–7.69 (m, 4H), 7.57–7.52 (m, 2H), 7.49–7.44 (m, 4H), 7.26–7.20 (m, 4H), 7.20–7.15 (m, 1H), 7.11–7.07 (m, 2H), 6.94–6.87 (m, 2H), 3.03 (t, *J* = 7.6 Hz, 2H), 2.99–2.92 (m, 2H), 2.78 (td, *J* = 7.6, 1.5 Hz, 2H), 2.72–2.65 (m, 2H). ^{13}C{^{19}F} NMR (151 MHz, $CDCl_3$) δ 184.9 (d, *J* = 9.8 Hz), 161.3, 140.8 (d, *J* = 15.4 Hz), 138.2, 132.4 (d, *J* = 2.8 Hz), 132.0 (d, *J* = 9.2 Hz), 130.1, 129.0 (d, *J* = 11.8 Hz), 128.7, 128.3(4) (d, *J* = 97.7 Hz), 128.2(9), 126.6, 115.0, 41.8 (d, *J* = 18.4 Hz), 32.1 (d, *J* = 2.0 Hz), 28.1 (d, *J* = 57.0 Hz), 27.8 (d, *J* = 2.5 Hz). ^{19}F{^{1}H} NMR (564 MHz, $CDCl_3$) δ −118.37. ^{31}P NMR (242 MHz, $CDCl_3$) δ 22.17 (m). FTIR (neat), cm^{-1}: 1580 (m), 1567 (m), 1507 (m), 1363 (m), 1218 (m), 1113 (m), 738 (s), 691 (s), 500 (m). HRMS (ESI): Calcd for $[C_{29}H_{27}NOPFNa]^+$: 478.17065, found: 478.16725.

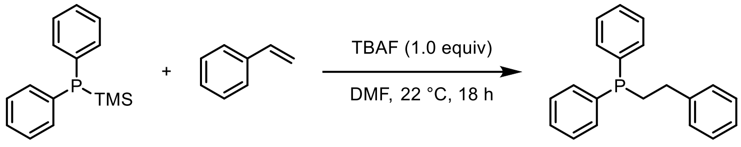

67

Diphenyl(2-phenylethyl)phosphine (**67**)

Phosphine **67** was prepared according to a modified literature procedure.[53] Tetrabutylammonium fluoride solution (1 M in THF, 1.5 mL, 1.5 mmol, 1.0 equiv) was added to a mixture of diphenyl(trimethylsilyl)phosphine (0.45 mL, 1.8 mmol, 1.2 equiv) and styrene (0.17 mL, 1.5 mmol, 1 equiv) in DMF (11 mL) at ambient temperature (22 °C). The resulting mixture was allowed to stir at 22 °C for 18 h. The mixture was diluted with diethyl ether (100 mL), and the diluted solution was washed with water (2 × 50 mL) and brine (50 mL). The organic fraction was dried over anhydrous magnesium sulfate, the dried solution was filtered, and the filtrate was concentrated under reduced pressure. The crude product mixture was purified by FCC (2% ethyl acetate in pentane) to provide **67** as a colorless oil (343 mg, 81%). The product is air-sensitive and was stored under argon atmosphere in a fridge. The spectroscopic data of **67** were in accordance with the data reported in the literature.[53] ^{1}H NMR (400 MHz, $CDCl_3$) δ 7.50–7.41 (m, 4H), 7.39–7.24 (m, 8H), 7.22–7.15 (m, 3H), 2.78–2.66 (m, 2H), 2.42–2.32 (m, 2H). ^{13}C NMR (101 MHz, $CDCl_3$) δ 142.8 (d, J = 13.4 Hz), 138.6 (d, J = 12.8 Hz), 132.9 (d, J = 18.5 Hz), 128.8 (d, J = 12.8 Hz), 128.6, 128.6, 128.3, 126.2, 32.3 (d, J = 17.9 Hz), 30.4 (d, J = 12.7 Hz). ^{31}P NMR (162 MHz, $CDCl_3$) δ −15.94 (m).

Evaluation of mechanistic pathways proceeding through phosphonium intermediates

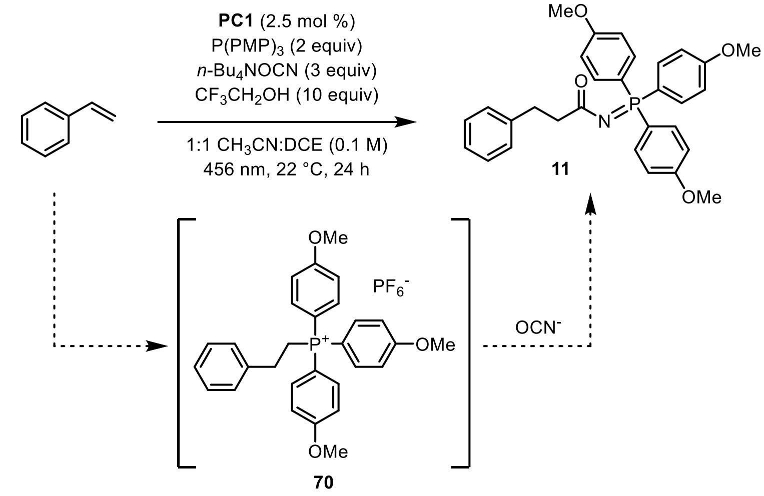

To evaluate whether a pathway proceeding through phosphonium salt intermediates was operative, we independently synthesized phosphonium salt **70** and subjected it to the reaction conditions. Phosphonium salt **70** was found unreactive under the reaction conditions, and product **11** was not detected in the reaction mixture.

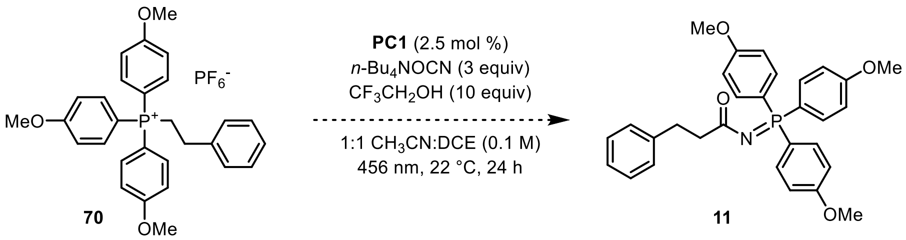

A mixture of tetrabutylammonium cyanate (85 mg, 0.30 mmol, 3.0 equiv) and iridium catalyst **PC1** (2.8 mg, 2.5 µmol, 0.025 equiv) in acetonitrile (0.5 mL) was added to a solution of phosphonium salt **70** (60 mg, 0.10 mmol, 1 equiv) in 1,2-dichloroethane (0.5 mL) in a Schlenk tube at ambient temperature under argon atmosphere. 2,2,2-Trifluoroethanol (73 µL, 1.0 mmol, 10 equiv) was added via syringe at ambient temperature. The reaction tube was placed in a photoreactor equipped with a 456 nm Kessil lamp. The reaction mixture was stirred at ambient temperature (22 °C) for 24 h. The reaction mixture was concentrated under reduced pressure. The crude reaction mixture was analyzed by ^{1}H and ^{31}P NMR analyses. *N*-acyl iminophosphorane **11** was not detected in the crude reaction mixture.

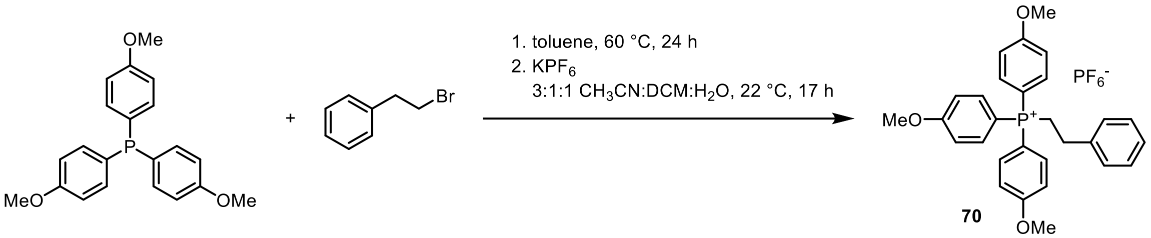

70

<u>Tris(4-methoxyphenyl)(phenethyl)phosphonium hexafluorophosphate(V) (**70**)</u>

2-Phenylethyl bromide (0.27 mL, 2.0 mmol, 1.0 equiv) was added to a suspension of tris(4-methoxyphenyl)phosphine (0.71 g, 2.0 mmol, 1 equiv) in toluene (8 mL). The resulting mixture was heated to 60 °C and was stirred at this temperature for 24 h. The reaction mixture was allowed to cool down to ambient temperature and was concentrated under reduced pressure. The residue was dissolved in DCM (10 mL) and was added dropwise to vigorously stirred diethyl ether (300 mL). The mixture was filtered through a fritted funnel, and the precipitated solids were collected. The crude tris(4-methoxyphenyl)(2-phenylethyl)phosphonium bromide (101 mg, 9%) was used in the next step without further purification. The crude tris(4-methoxyphenyl)(2-phenylethyl)phosphonium bromide (101 mg, 0.19 mmol, 1 equiv) was dissolved in a mixture of acetonitrile (1 mL) and dichloromethane (0.6 mL). A solution of potassium hexafluorophosphate (41 mg, 0.22 mmol, 1.2 equiv) in water (0.3 mL) was added at ambient temperature (22 °C), and the resulting mixture was stirred at 22 °C for 17 h. The reaction mixture was concentrated under reduced pressure, and the residue was dissolved in dichloromethane (50 mL). The solution was washed with water (30 mL), and the aqueous layer was extracted with dichloromethane (3 × 50 mL). The combined organic fractions were dried over anhydrous magnesium sulfate, the dried solution was filtered, and the filtrate was concentrated under reduced pressure to provide phosphonium salt **70** as a white amorphous solid (104 mg, 93%). The phosphonium salt was used without further purification. ^{1}H NMR (400 MHz, $CDCl_3$) δ 7.60–7.46 (m, 6H), 7.31–7.23 (m, 2H; overlapping with the solvent signal), 7.23–7.10 (m, 9H), 3.91 (s, 9H), 3.31–3.16 (m, 2H), 2.99–2.82 (m, 2H). ^{13}C NMR (101 MHz, $CDCl_3$) δ 165.0 (d, *J* = 3.0 Hz), 138.3 (d, *J* = 14.2 Hz), 135.4 (d, *J* = 11.6 Hz), 129.2, 128.4, 127.4, 116.5 (d, *J* = 13.7 Hz), 108.4 (d, *J* = 94.3 Hz), 56.1, 28.4 (d, *J* = 3.4 Hz), 25.7 (d, *J* = 52.4 Hz). ^{19}F NMR (376 MHz, $CDCl_3$) δ −72.96 (d, *J* = 712.9 Hz). ^{31}P NMR (162 MHz, $CDCl_3$) δ 20.71 (m, 1P), −144.3 (hept, *J* = 712.8 Hz, 1P). FTIR (neat), cm^{-1}: 1592 (s), 1502 (m), 1262 (s), 1183 (m), 1110 (s), 1019 (m), 824 (vs), 555 (m), 527 (s). HRMS (ESI): Calcd for $[C_{29}H_{30}O_3P]^+$: 457.19271, found: 457.19195.

NMR spectra

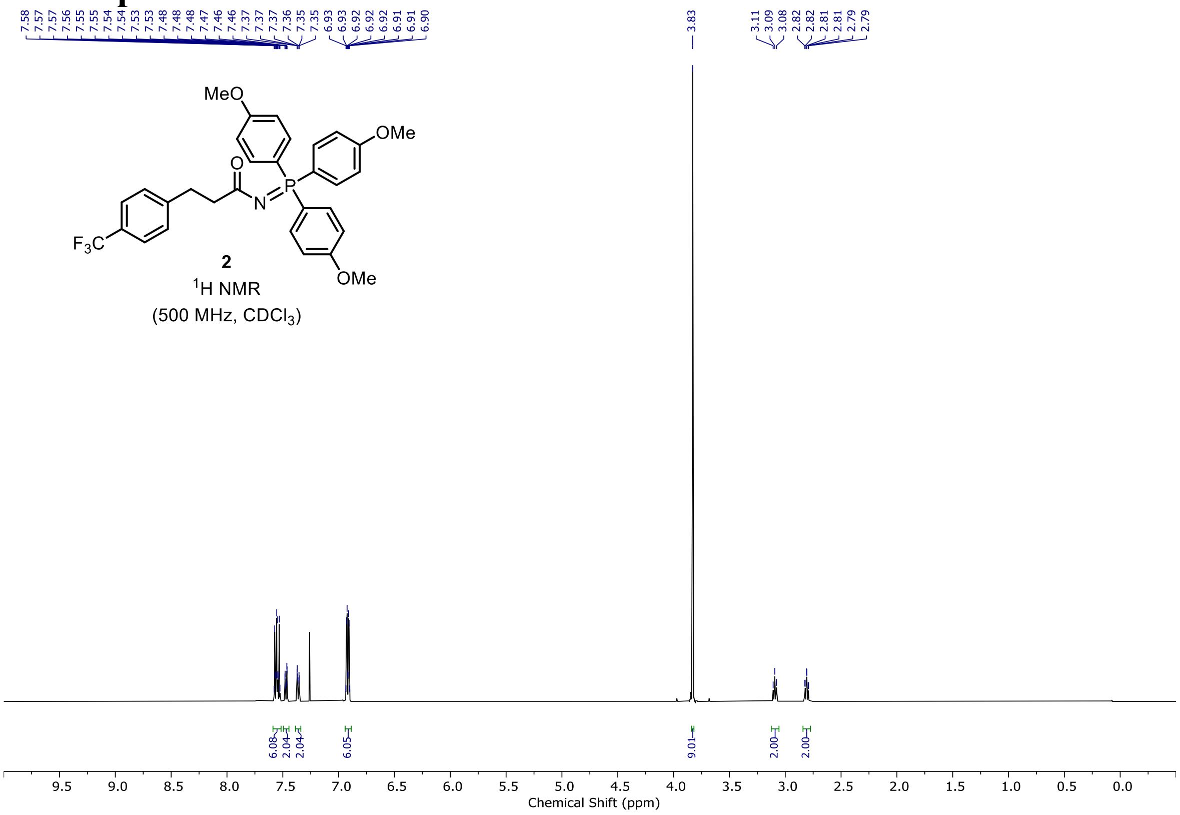

MeO
OMe
O
N
P
F3C
OMe
2
1H NMR
(500 MHz, CDCl3)
Chemical Shift (ppm)

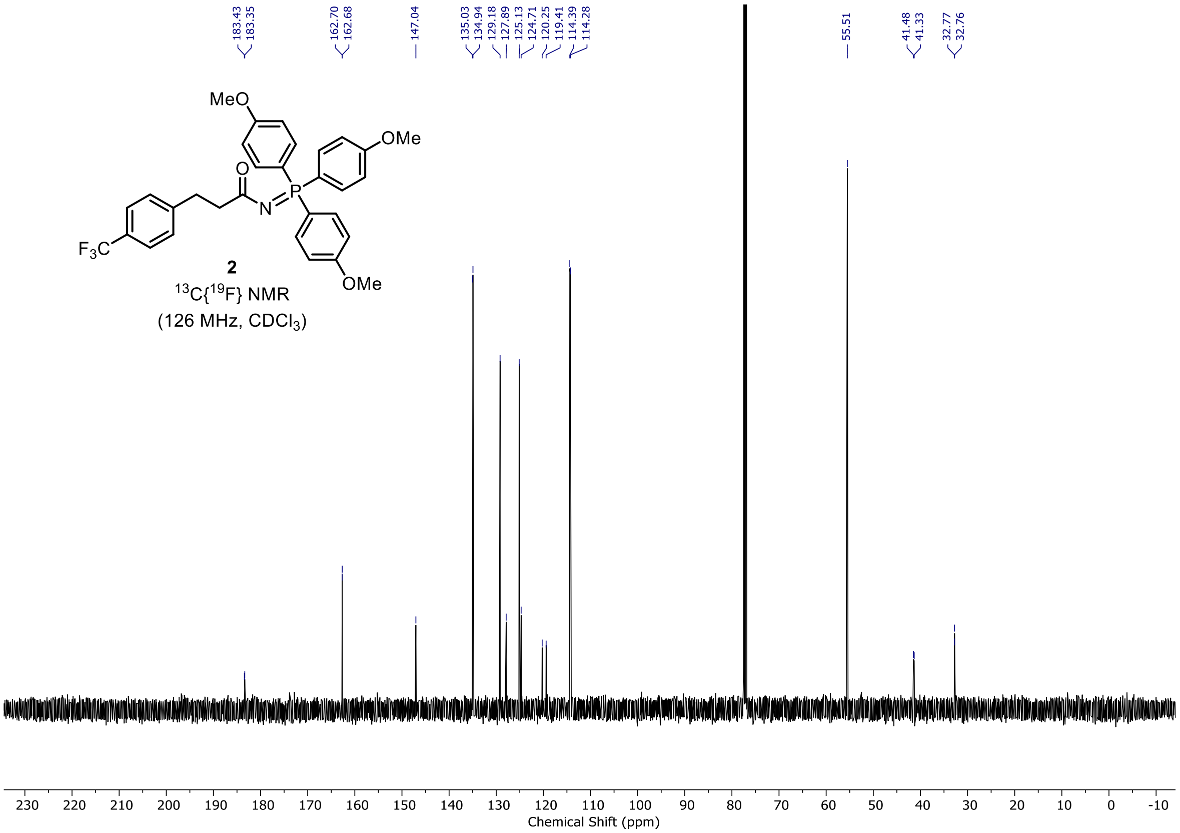

MeO
OMe
O
N
P
F3C
OMe
2
13C{19F} NMR
(126 MHz, CDCl3)
Chemical Shift (ppm)

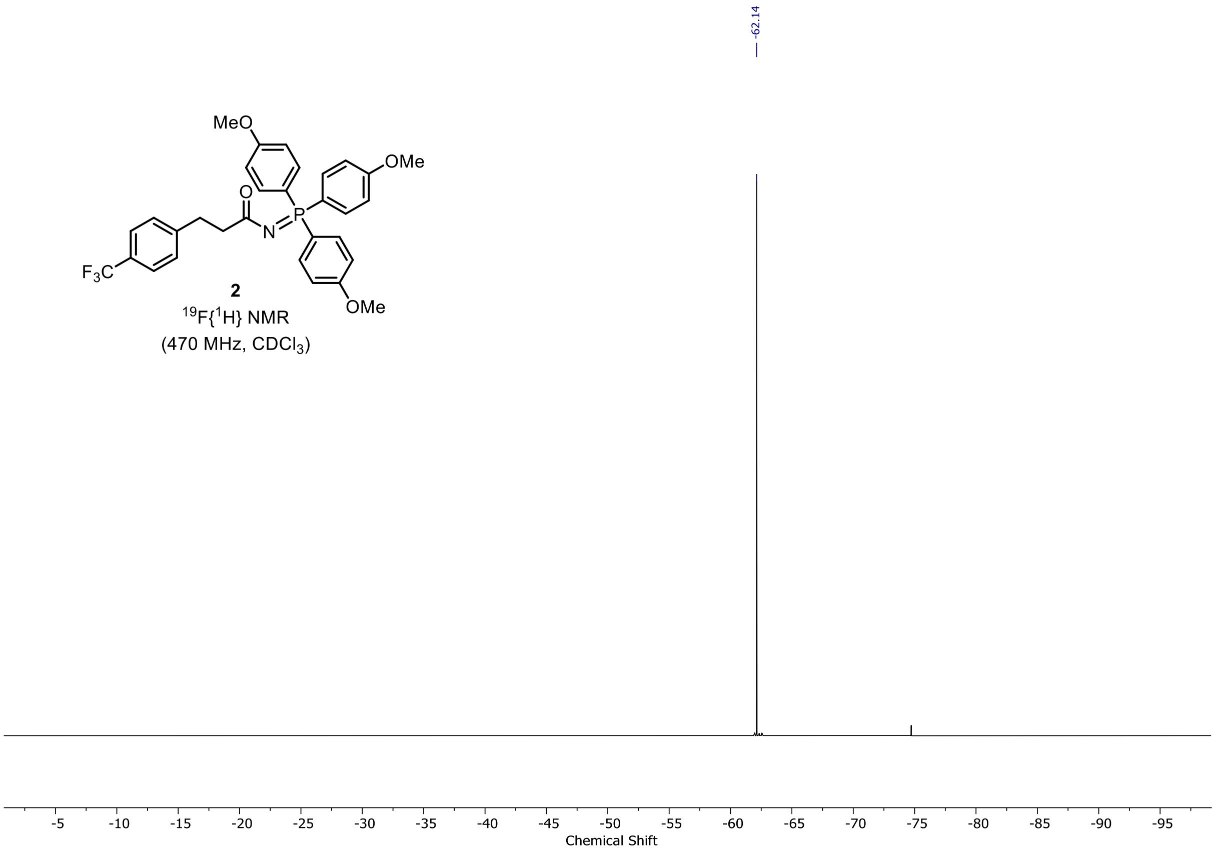
-62.14
MeO
OMe
OMe
O
N
P
F3C
2
19F{1H} NMR
(470 MHz, CDCl3)
-5 -10 -15 -20 -25 -30 -35 -40 -45 -50 -55 -60 -65 -70 -75 -80 -85 -90 -95
Chemical Shift

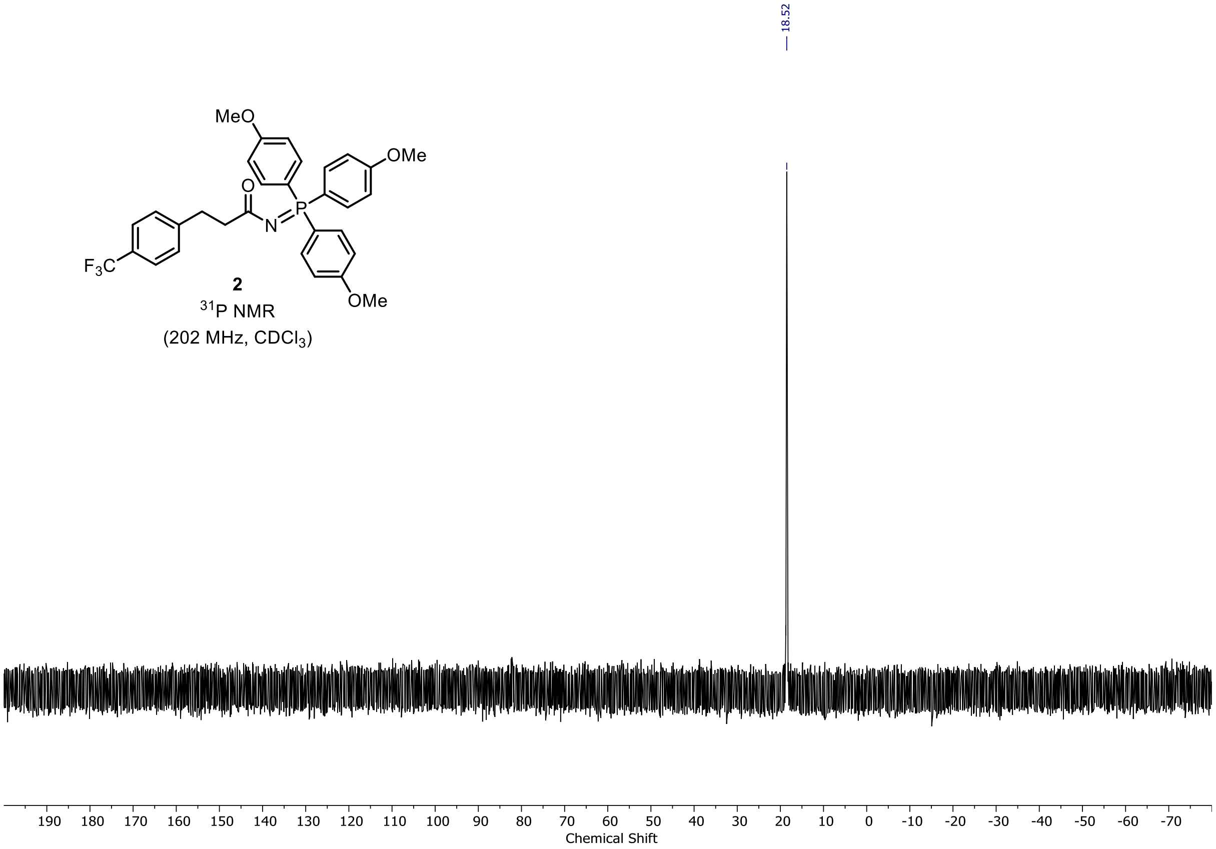
18.52
MeO
OMe
OMe
O
N
P
F3C
2
31P NMR
(202 MHz, CDCl3)
190 180 170 160 150 140 130 120 110 100 90 80 70 60 50 40 30 20 10 0 -10 -20 -30 -40 -50 -60 -70
Chemical Shift

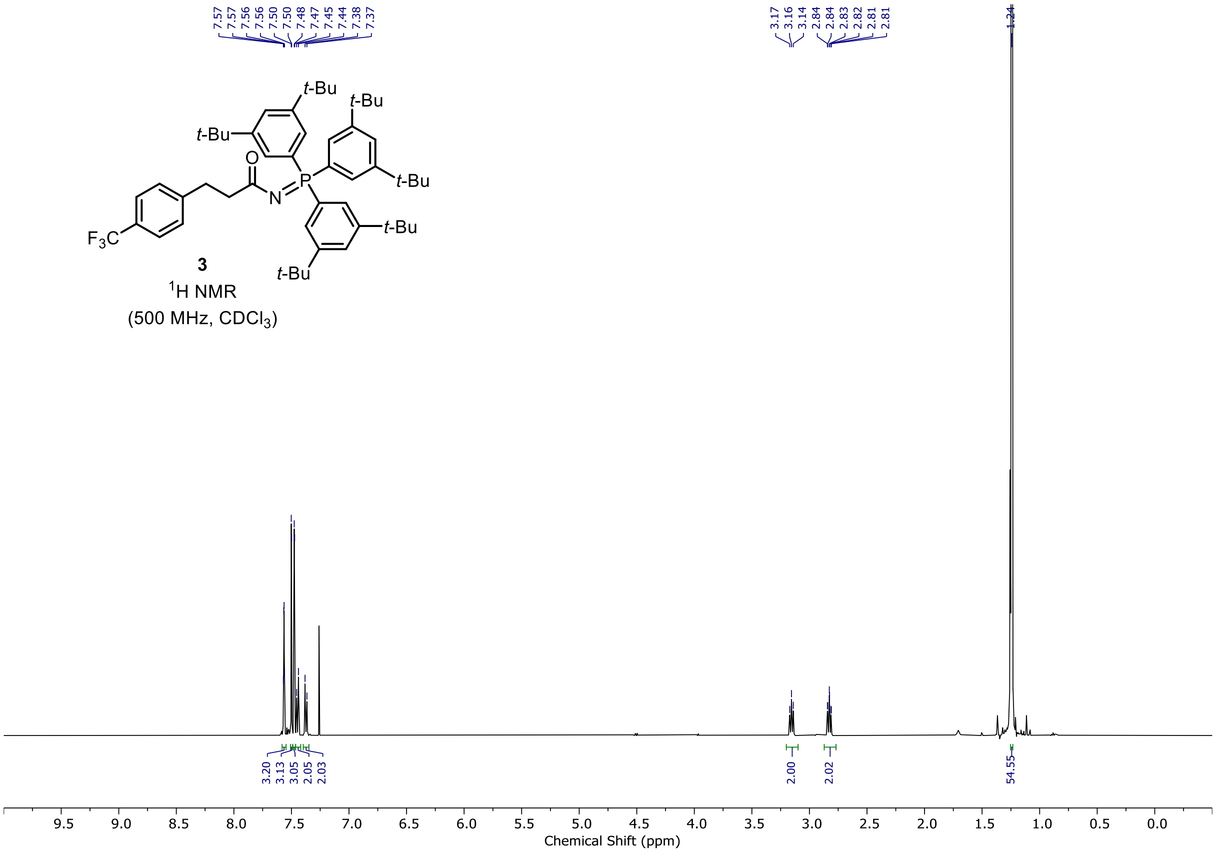
7.57 7.57 7.56 7.56 7.50 7.50 7.48 7.47 7.45 7.44 7.38 7.37
3.17 3.16 3.14 2.84 2.84 2.83 2.82 2.81 2.81
1.24
t-Bu
F3C
O
N
P
3
1H NMR
(500 MHz, CDCl3)
3.20 3.13 3.05 2.05 2.03
2.00
2.02
54.55
9.5 9.0 8.5 8.0 7.5 7.0 6.5 6.0 5.5 5.0 4.5 4.0 3.5 3.0 2.5 2.0 1.5 1.0 0.5 0.0
Chemical Shift (ppm)

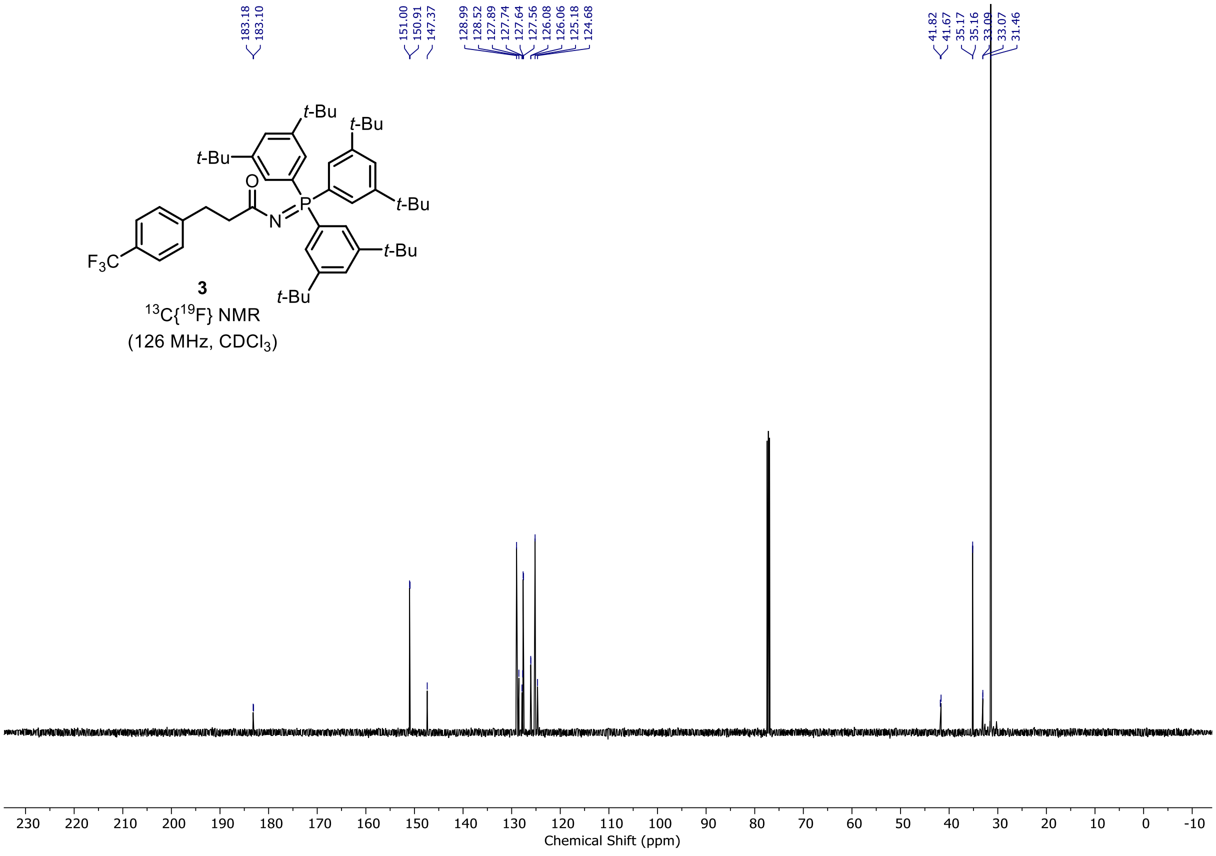
183.18 183.10
151.00 150.91 147.37
128.99 128.52 127.89 127.74 127.64 127.56 126.08 126.06 125.18 124.68
41.82 41.67 35.17 35.16 33.09 33.07 31.46
t-Bu
F3C
O
N
P
3
13C{19F} NMR
(126 MHz, CDCl3)
230 220 210 200 190 180 170 160 150 140 130 120 110 100 90 80 70 60 50 40 30 20 10 0 -10
Chemical Shift (ppm)

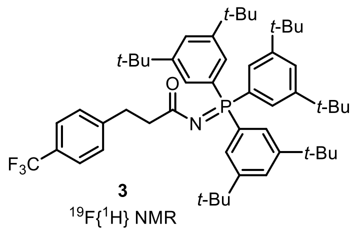

3

$^{19}F\{^{1}H\}$ NMR

(283 MHz, $CDCl_3$)

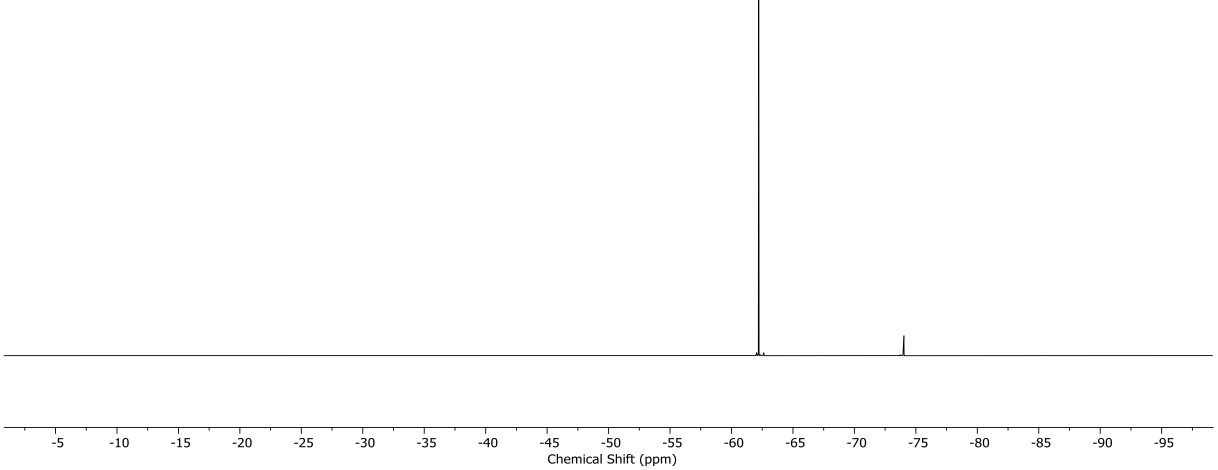

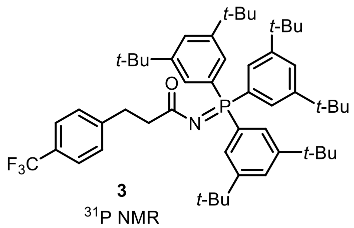

3

^{31}P NMR

(202 MHz, $CDCl_3$)

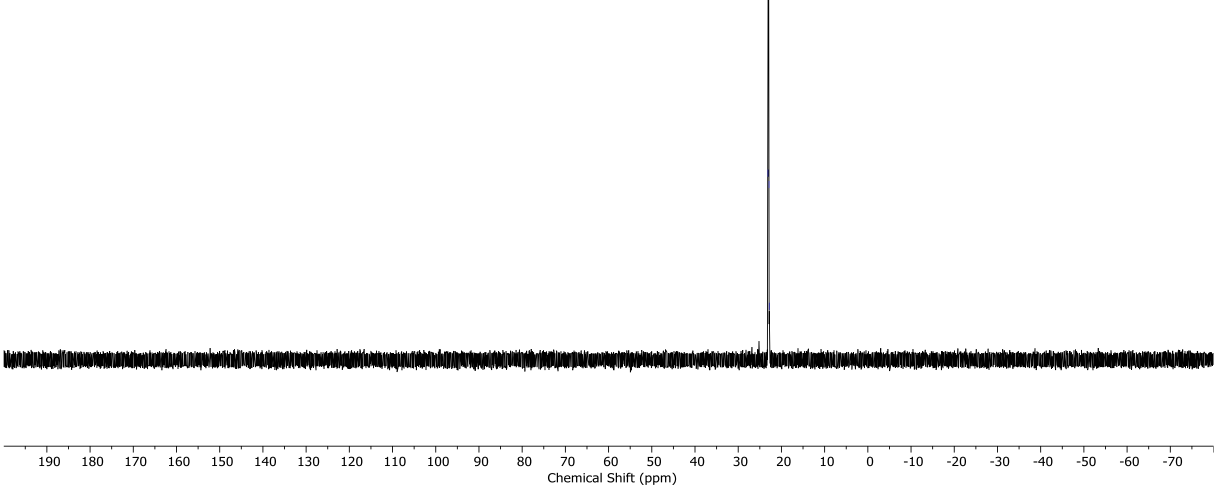

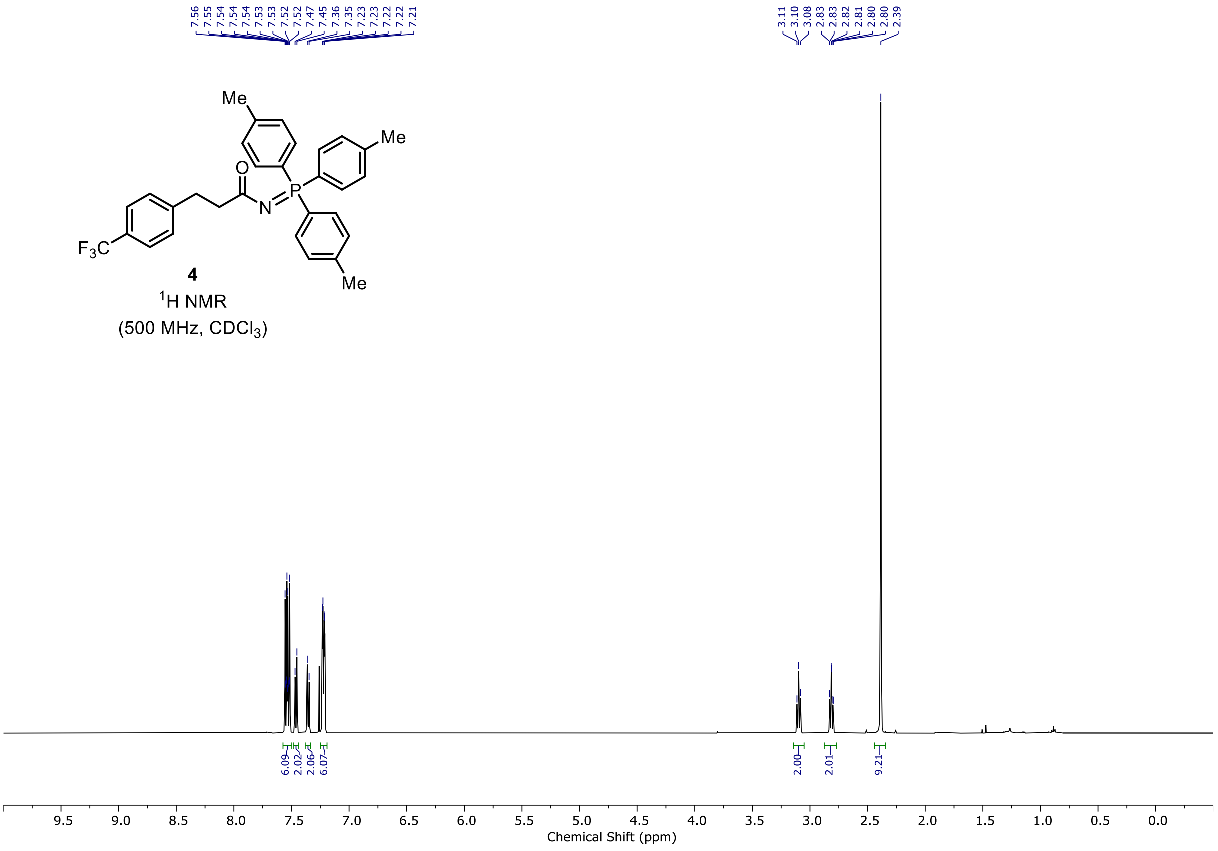

7.56
7.55
7.54
7.54
7.54
7.53
7.53
7.52
7.52
7.47
7.45
7.36
7.35
7.23
7.23
7.23
7.22
7.22
7.21
3.11
3.10
3.08
2.83
2.83
2.82
2.81
2.80
2.80
2.39
Me
Me
O
N
P
F3C
Me
4
1H NMR
(500 MHz, CDCl3)
6.09
2.02
2.06
6.07
2.00
2.01
9.21
9.5
9.0
8.5
8.0
7.5
7.0
6.5
6.0
5.5
5.0
4.5
4.0
3.5
3.0
2.5
2.0
1.5
1.0
0.5
0.0
Chemical Shift (ppm)

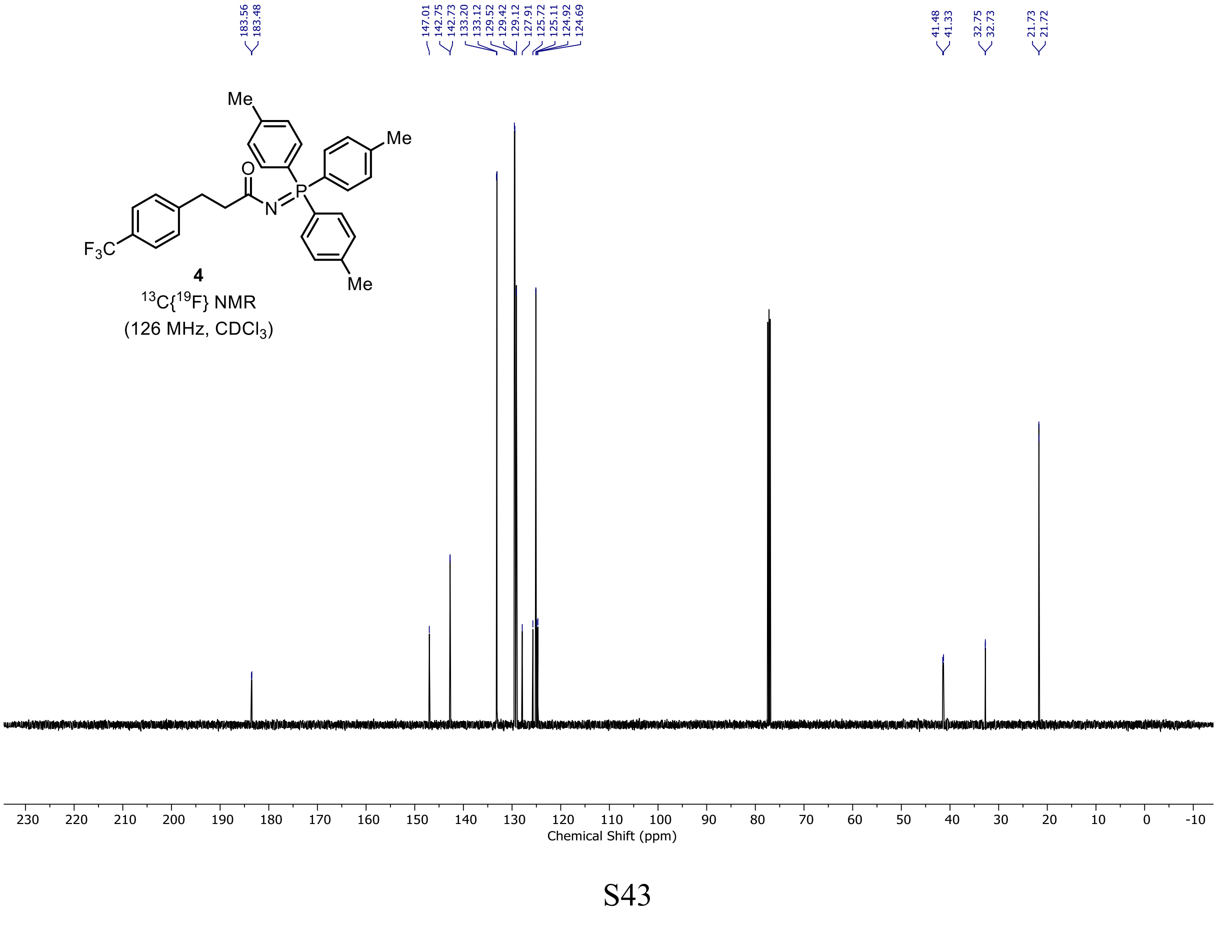

183.56
183.48
147.01
142.75
142.73
133.20
133.12
129.52
129.42
129.12
127.91
125.72
125.11
124.92
124.69
41.48
41.33
32.75
32.73
21.73
21.72
Me
Me
O
N
P
F3C
Me
4
13C{19F} NMR
(126 MHz, CDCl3)
230
220
210
200
190
180
170
160
150
140
130
120
110
100
90
80
70
60
50
40
30
20
10
0
-10
Chemical Shift (ppm)

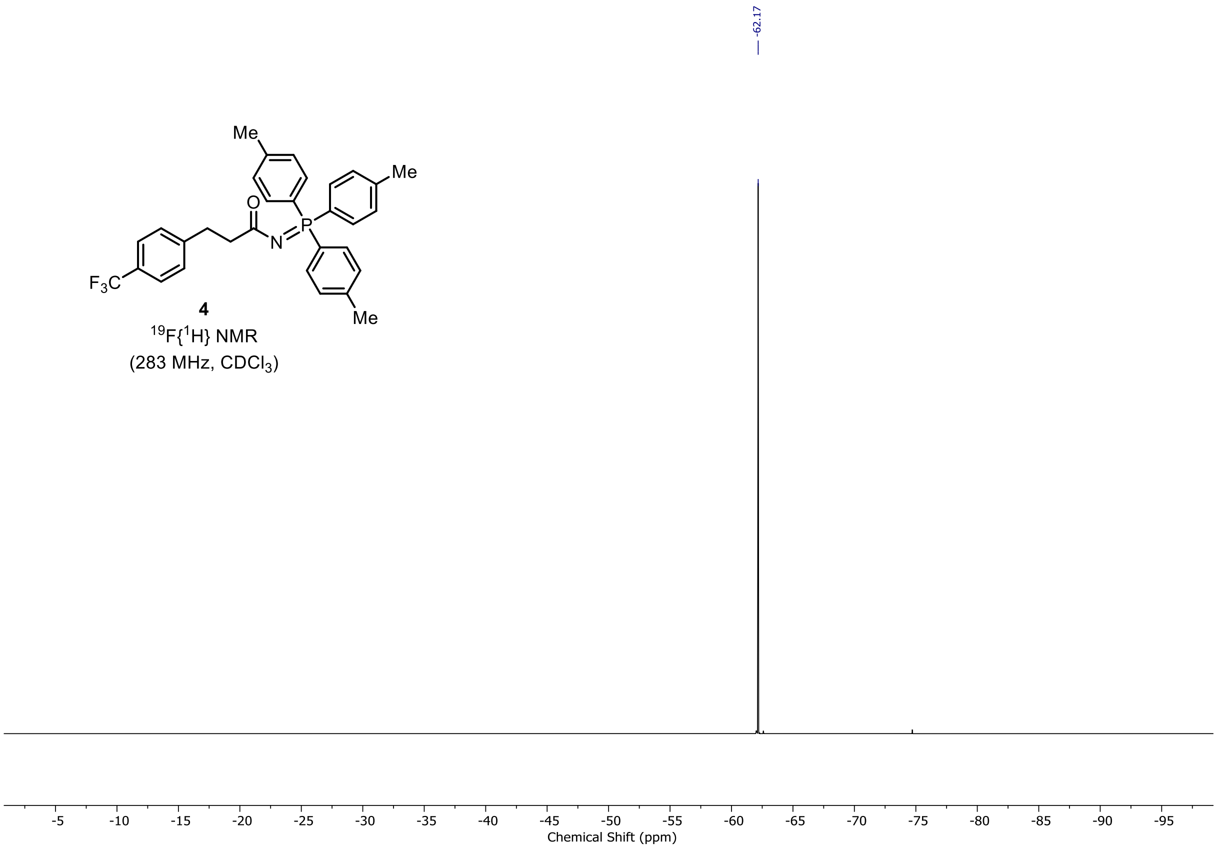
-62.17
Me
Me
O
N
P
F3C
Me
4
19F{1H} NMR
(283 MHz, CDCl3)
-5 -10 -15 -20 -25 -30 -35 -40 -45 -50 -55 -60 -65 -70 -75 -80 -85 -90 -95
Chemical Shift (ppm)

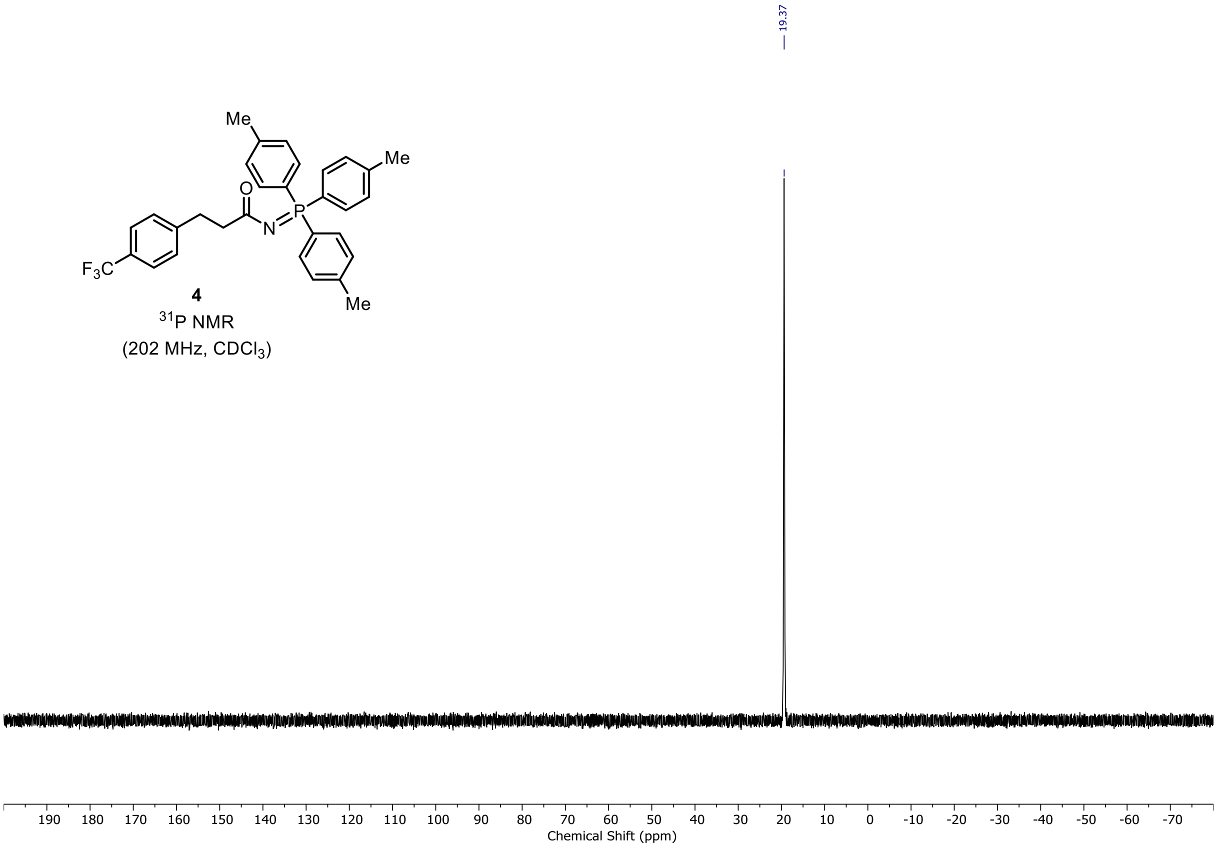
19.37
Me
Me
O
N
P
F3C
Me
4
31P NMR
(202 MHz, CDCl3)
190 180 170 160 150 140 130 120 110 100 90 80 70 60 50 40 30 20 10 0 -10 -20 -30 -40 -50 -60 -70
Chemical Shift (ppm)

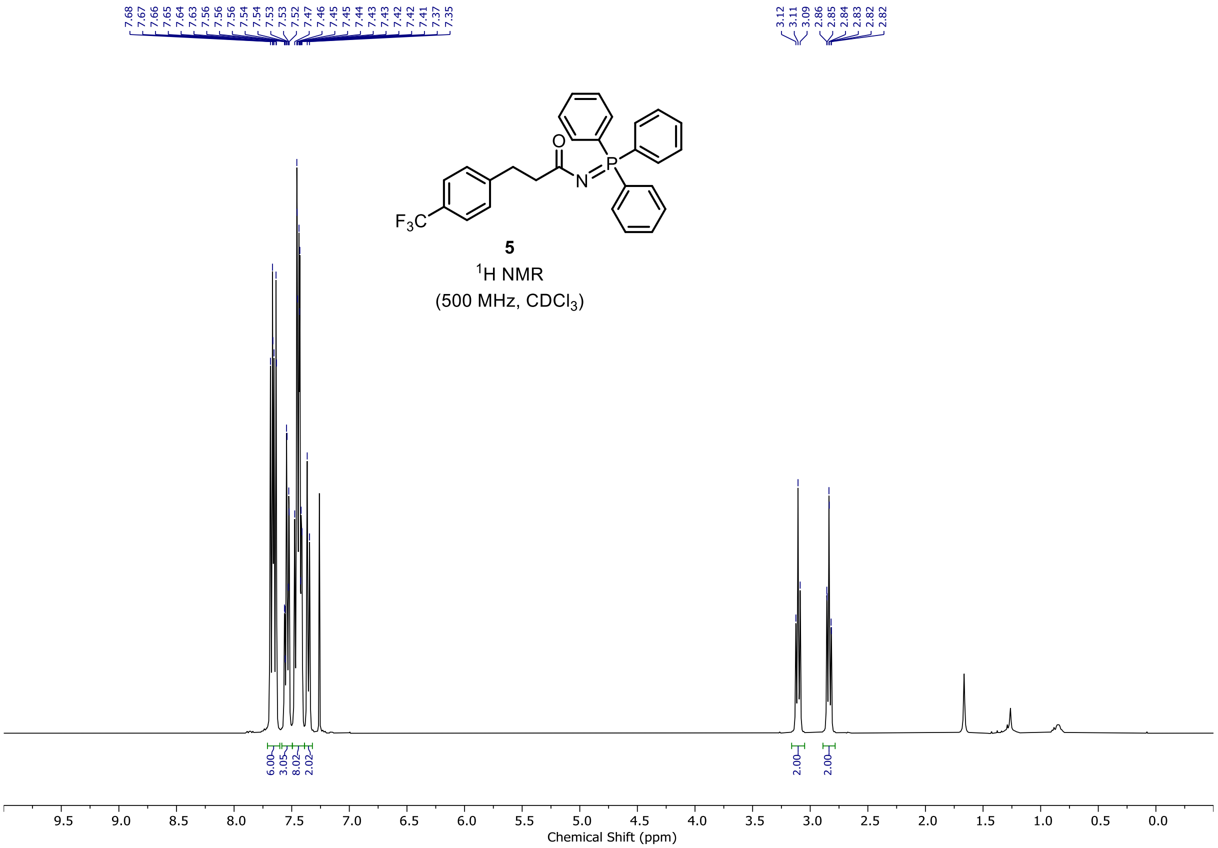
7.68 7.67 7.66 7.65 7.64 7.63 7.56 7.56 7.56 7.54 7.54 7.53 7.53 7.52 7.47 7.46 7.45 7.45 7.44 7.43 7.43 7.42 7.42 7.41 7.37 7.35
3.12 3.11 3.09 2.86 2.85 2.84 2.83 2.82 2.82
O
N
P
F3C
5
1H NMR
(500 MHz, CDCl3)
6.00 3.05 8.02 2.02
2.00 2.00
9.5 9.0 8.5 8.0 7.5 7.0 6.5 6.0 5.5 5.0 4.5 4.0 3.5 3.0 2.5 2.0 1.5 1.0 0.5 0.0
Chemical Shift (ppm)

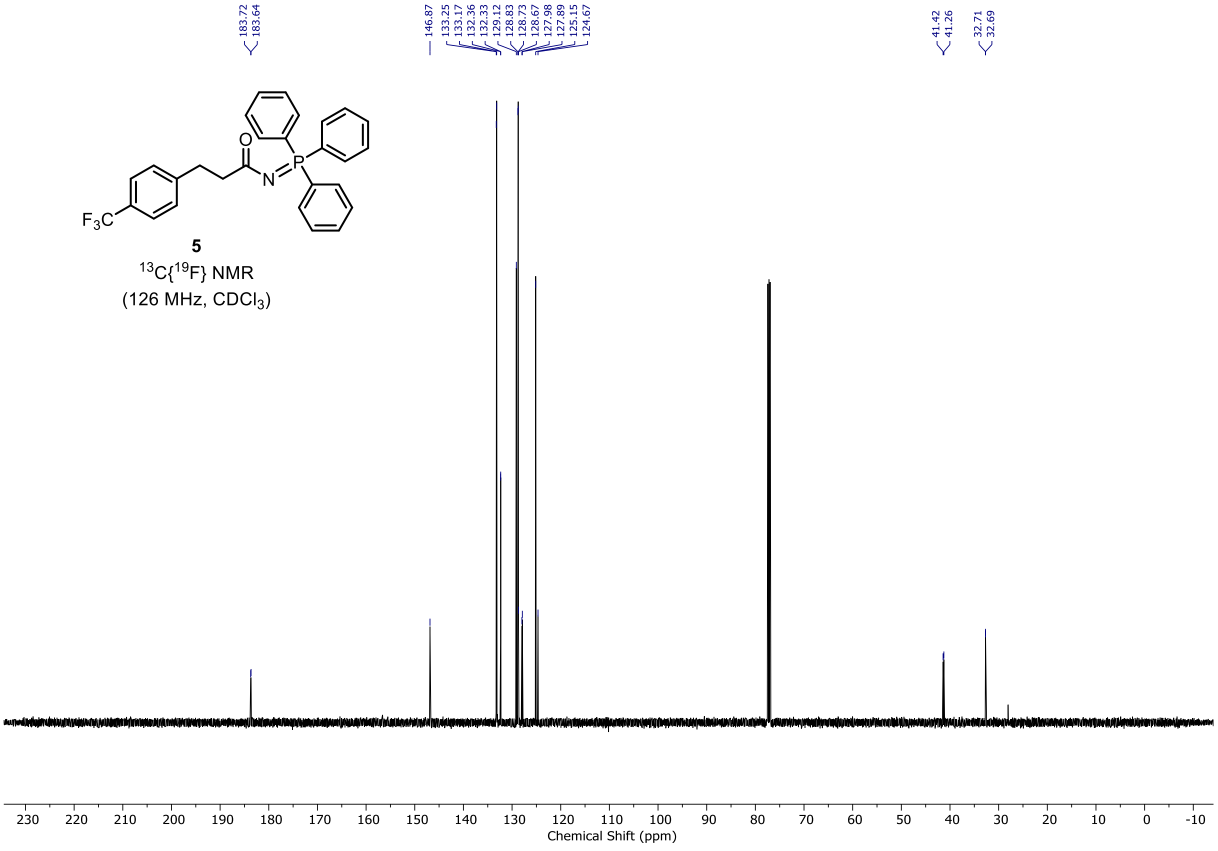
183.72 183.64
146.87 133.25 133.17 132.36 132.33 129.12 128.83 128.73 128.67 127.98 127.89 125.15 124.67
41.42 41.26 32.71 32.69
O
N
P
F3C
5
13C{19F} NMR
(126 MHz, CDCl3)
230 220 210 200 190 180 170 160 150 140 130 120 110 100 90 80 70 60 50 40 30 20 10 0 -10
Chemical Shift (ppm)

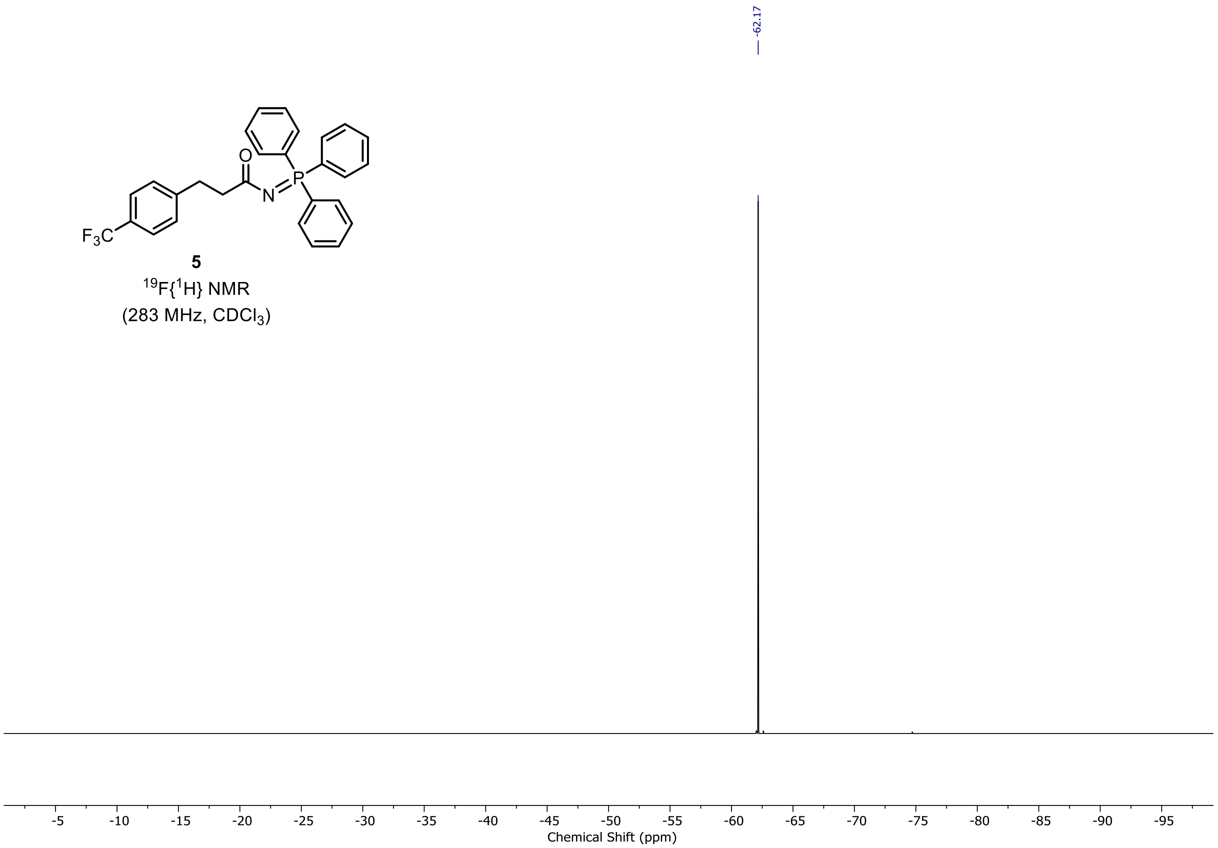

5

$^{19}F\{^{1}H\}$ NMR

(283 MHz, $CDCl_3$)

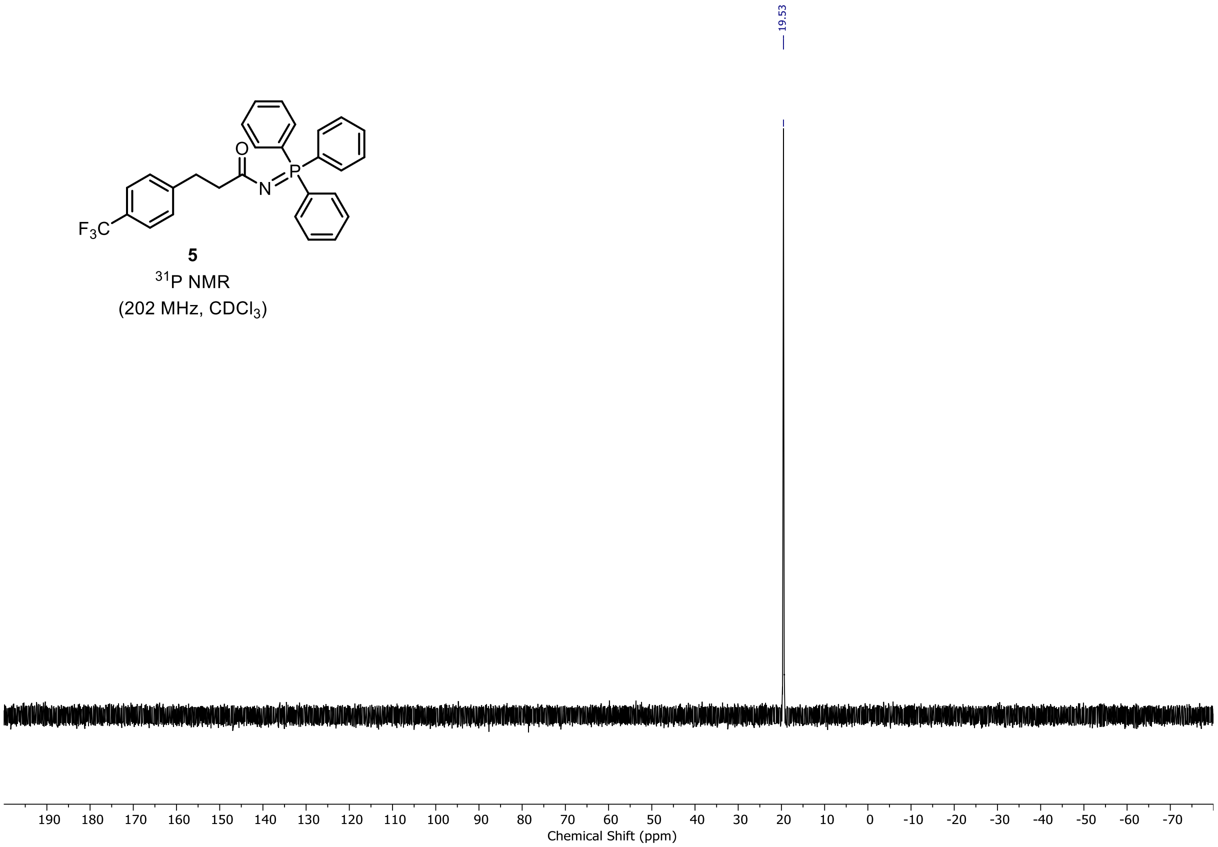

5

^{31}P NMR

(202 MHz, $CDCl_3$)

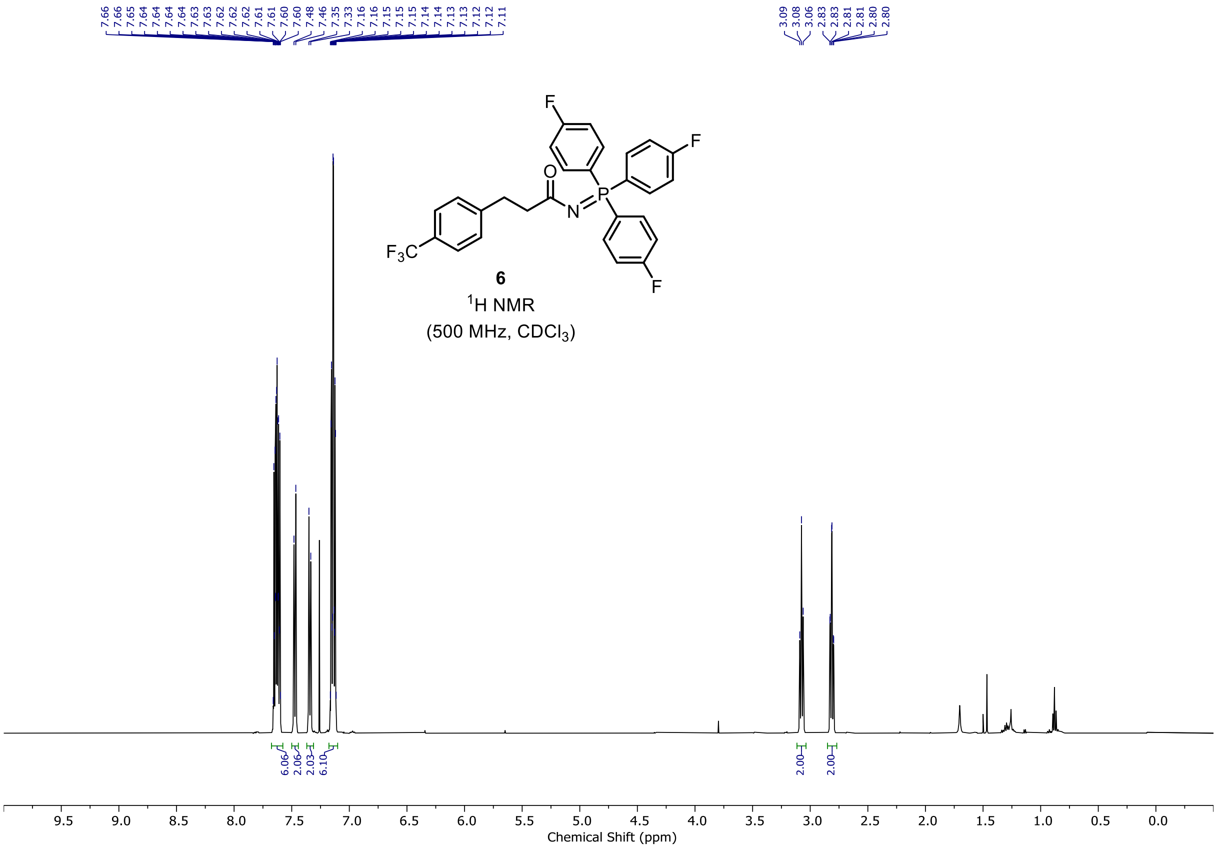
7.66 7.66 7.65 7.64 7.64 7.64 7.63 7.63 7.62 7.62 7.62 7.61 7.61 7.60 7.60 7.48 7.46 7.35 7.33 7.16 7.16 7.15 7.15 7.15 7.14 7.14 7.13 7.13 7.12 7.12 7.11
3.09 3.08 3.06 2.83 2.83 2.81 2.81 2.80 2.80
F
F
O
N
P
F
F_3C
6
^1H NMR
(500 MHz, $CDCl_3$)
6.06 2.06 2.03 6.10
2.00 2.00
9.5 9.0 8.5 8.0 7.5 7.0 6.5 6.0 5.5 5.0 4.5 4.0 3.5 3.0 2.5 2.0 1.5 1.0 0.5 0.0
Chemical Shift (ppm)

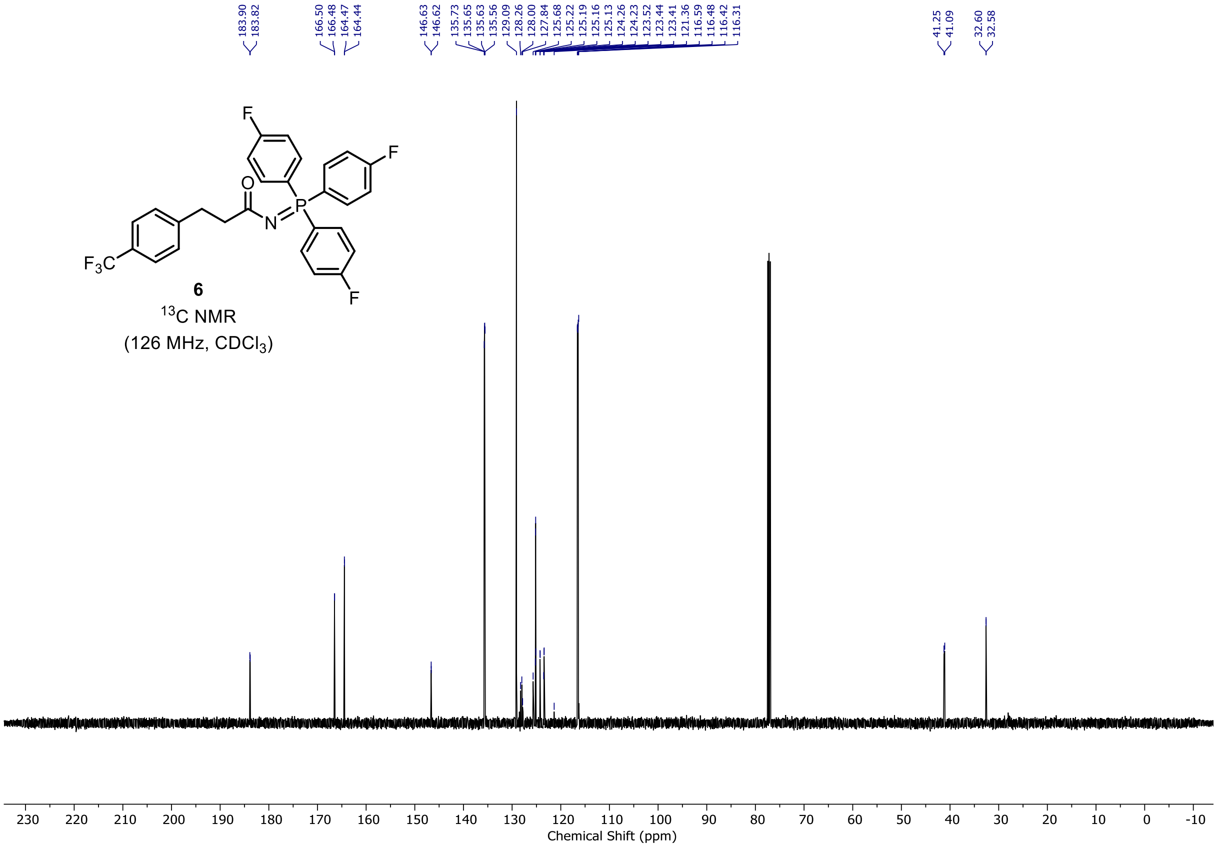
183.90 183.82
166.50 166.48 164.47 164.44
146.63 146.62 135.73 135.65 135.63 135.56 129.09 128.26 128.00 127.84 125.68 125.22 125.19 125.16 125.13 124.26 124.23 123.52 123.44 123.41 121.36 116.59 116.48 116.42 116.31
41.25 41.09 32.60 32.58
F
F
O
N
P
F
F_3C
6
^{13}C NMR
(126 MHz, $CDCl_3$)
230 220 210 200 190 180 170 160 150 140 130 120 110 100 90 80 70 60 50 40 30 20 10 0 -10
Chemical Shift (ppm)

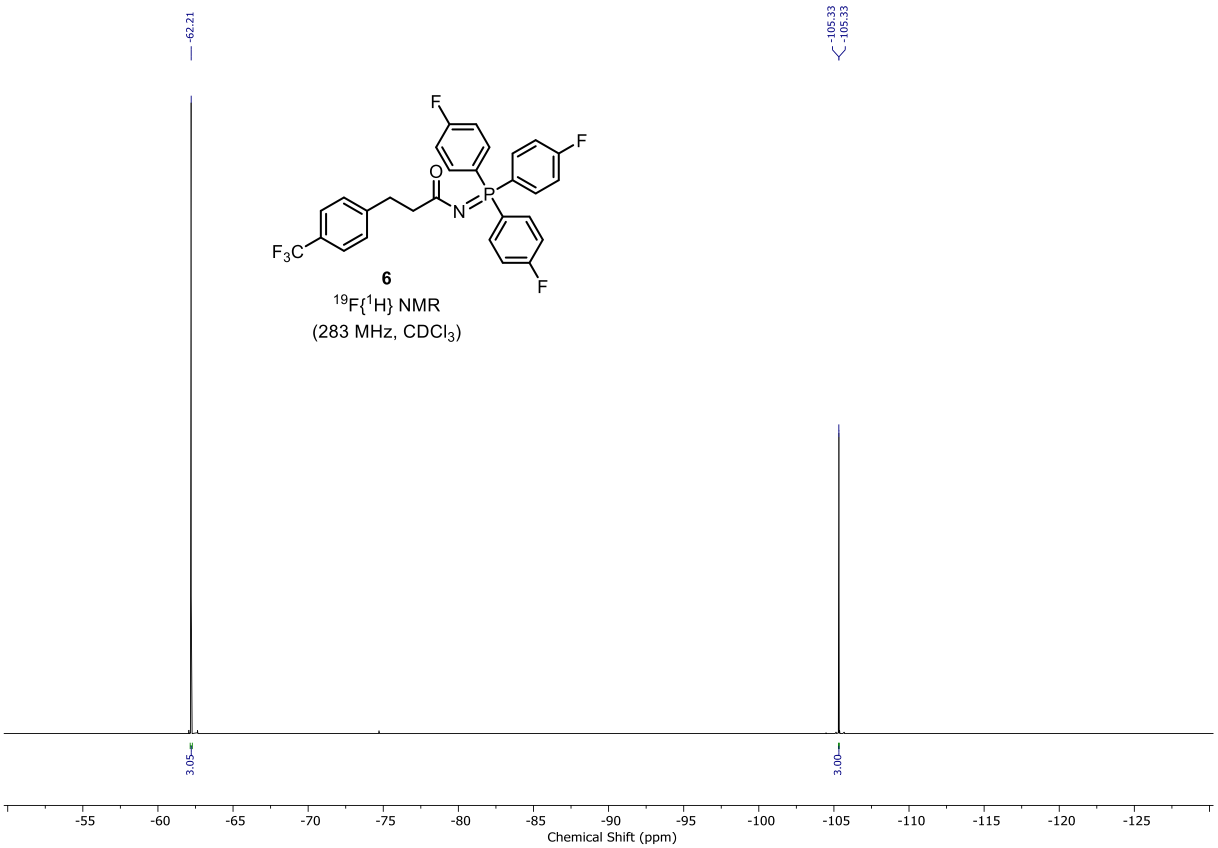
-62.21
-105.33
-105.33
F
F
O
N
P
F3C
F
6
19F{1H} NMR
(283 MHz, CDCl3)
3.05
3.00
-55 -60 -65 -70 -75 -80 -85 -90 -95 -100 -105 -110 -115 -120 -125
Chemical Shift (ppm)

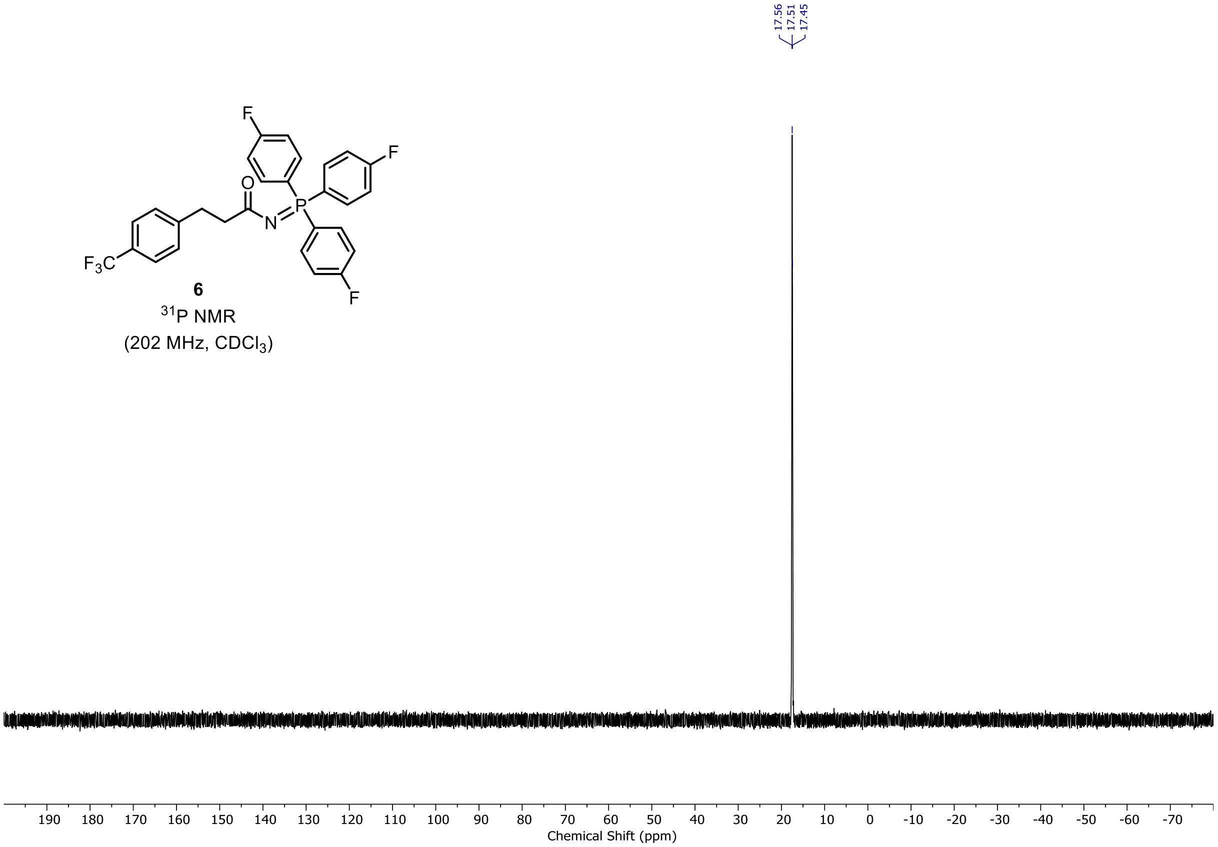
17.56
17.51
17.45
F
F
O
N
P
F3C
F
6
31P NMR
(202 MHz, CDCl3)
190 180 170 160 150 140 130 120 110 100 90 80 70 60 50 40 30 20 10 0 -10 -20 -30 -40 -50 -60 -70
Chemical Shift (ppm)

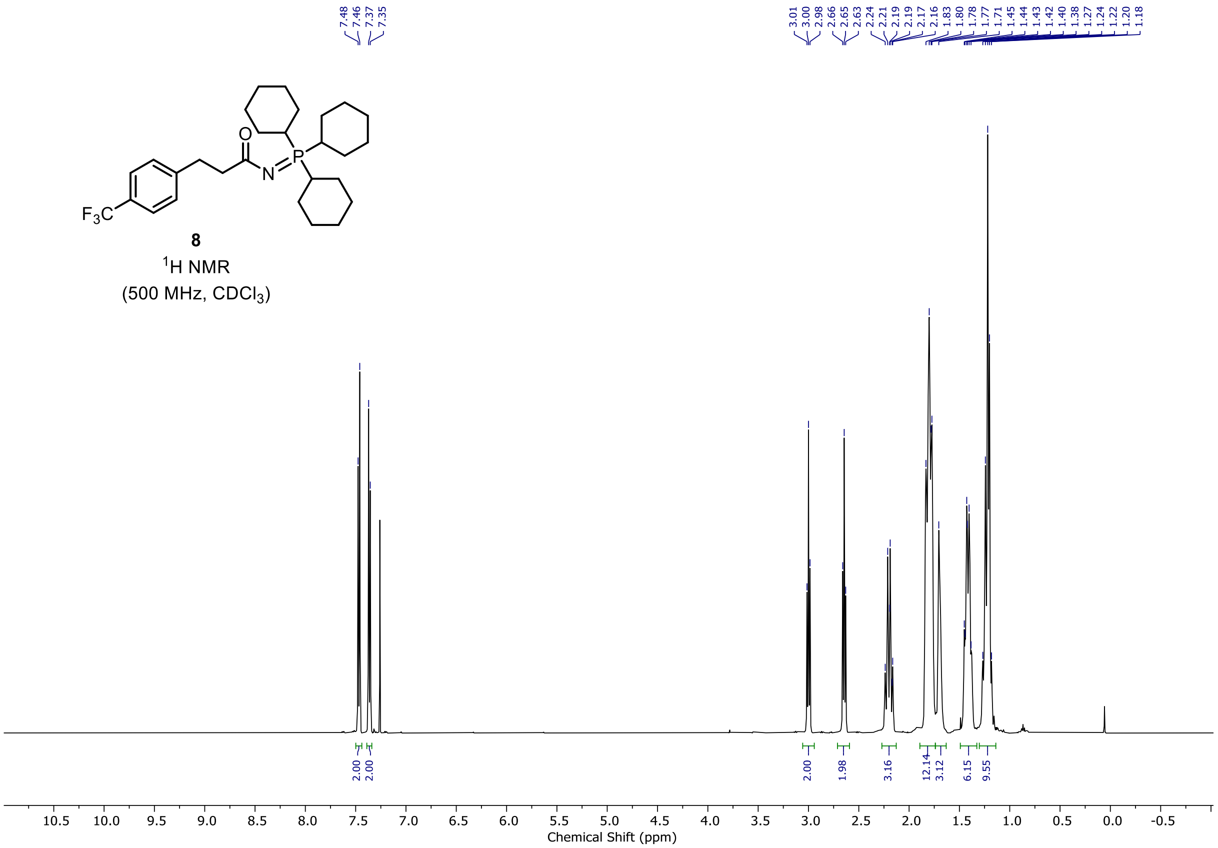
F3C
8
1H NMR
(500 MHz, CDCl3)
Chemical Shift (ppm)

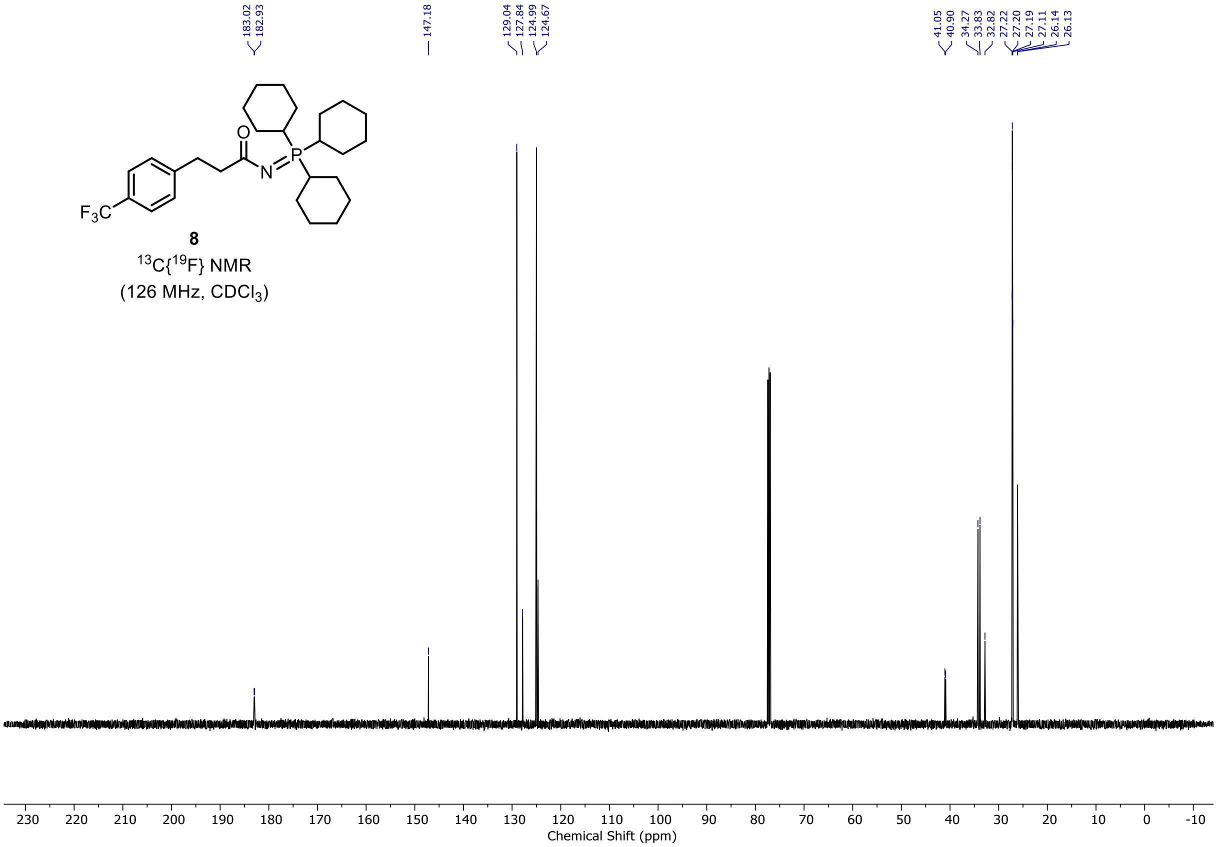
F3C
8
13C{19F} NMR
(126 MHz, CDCl3)
Chemical Shift (ppm)

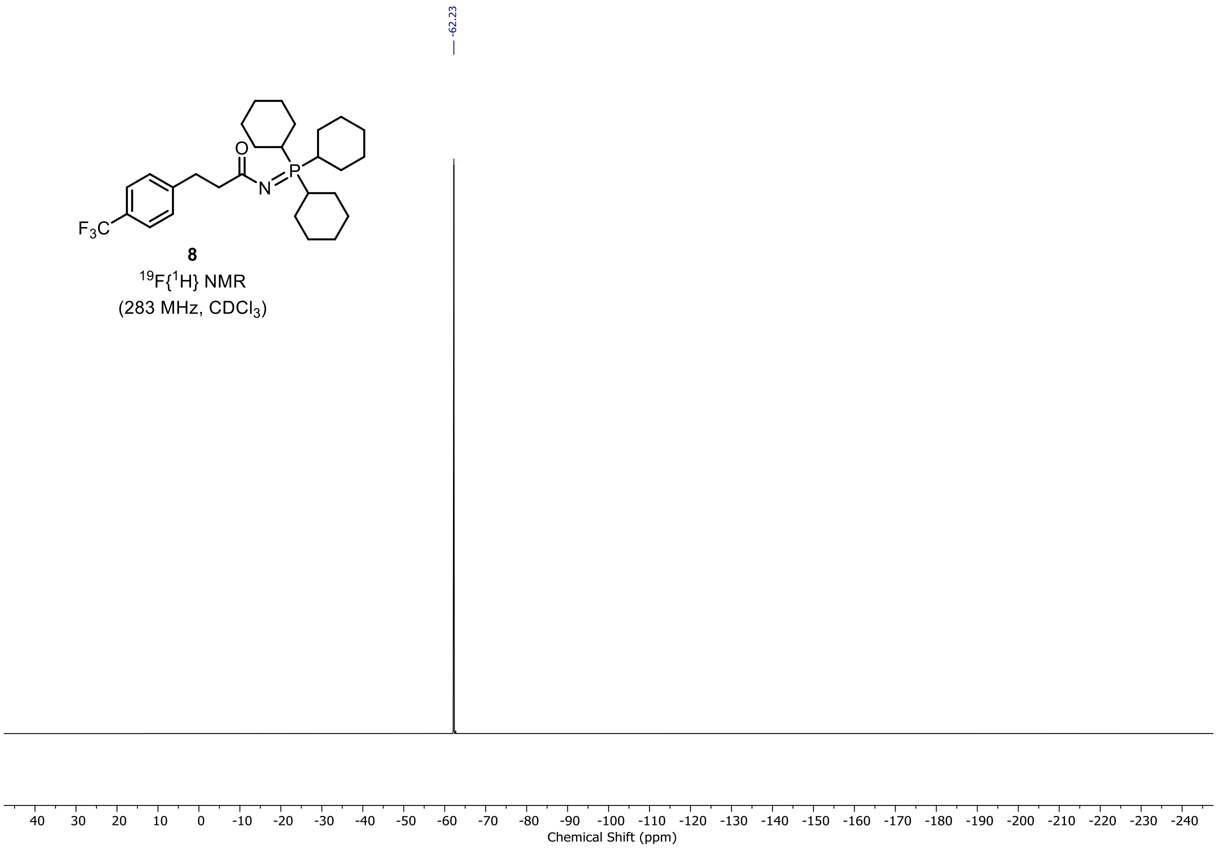
-62.23
F3C
O
N
P
8
19F{1H} NMR
(283 MHz, CDCl3)
40 30 20 10 0 -10 -20 -30 -40 -50 -60 -70 -80 -90 -100 -110 -120 -130 -140 -150 -160 -170 -180 -190 -200 -210 -220 -230 -240
Chemical Shift (ppm)

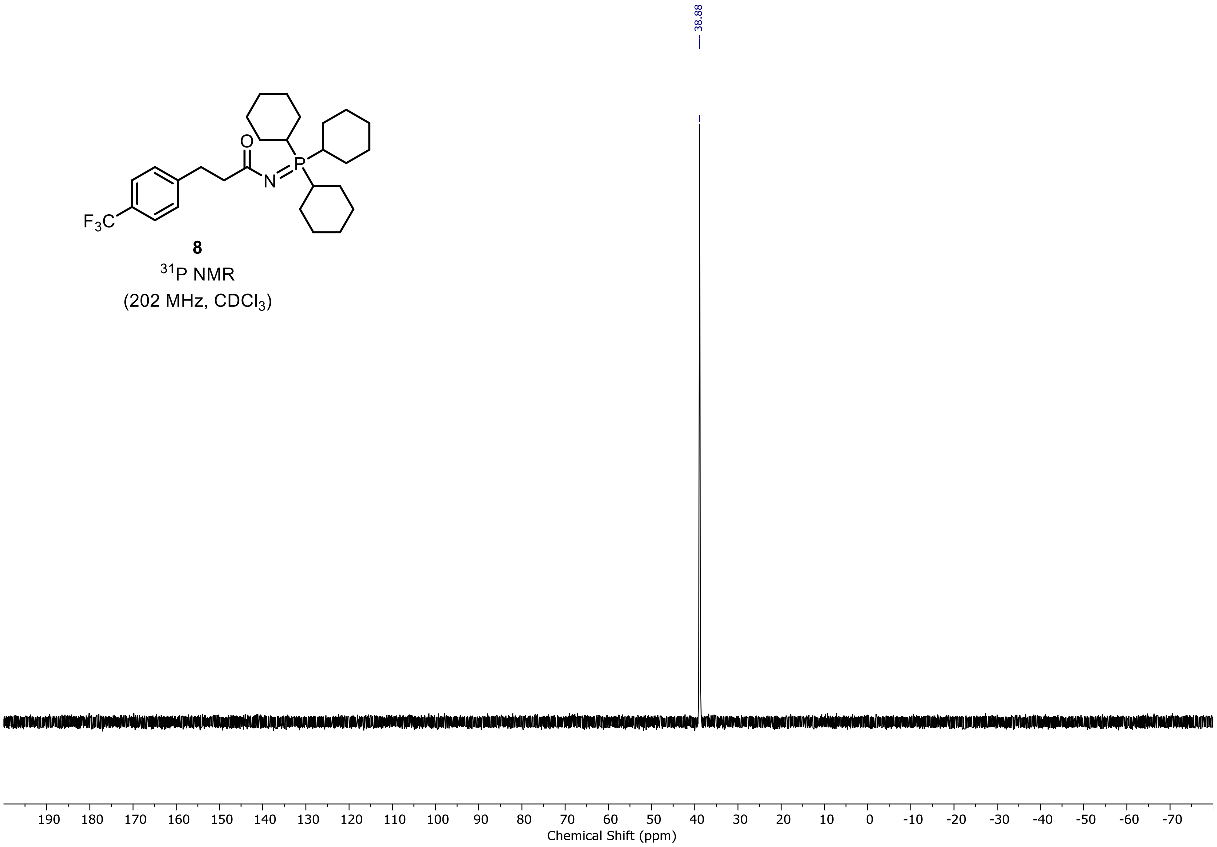
38.88
F3C
O
N
P
8
31P NMR
(202 MHz, CDCl3)
190 180 170 160 150 140 130 120 110 100 90 80 70 60 50 40 30 20 10 0 -10 -20 -30 -40 -50 -60 -70
Chemical Shift (ppm)

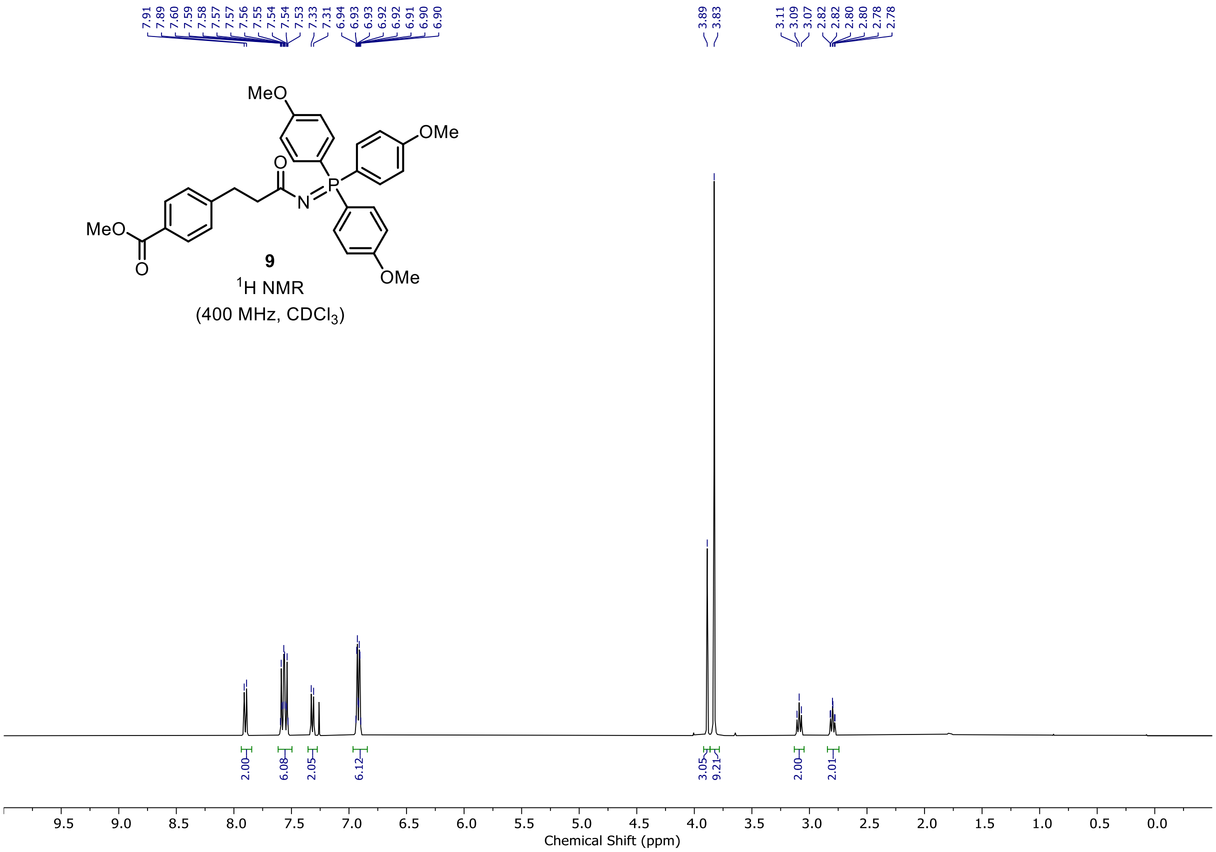
MeO
OMe
O
N
P
MeO
O
OMe
9
^{1}H NMR
(400 MHz, CDCl$_3$)
Chemical Shift (ppm)

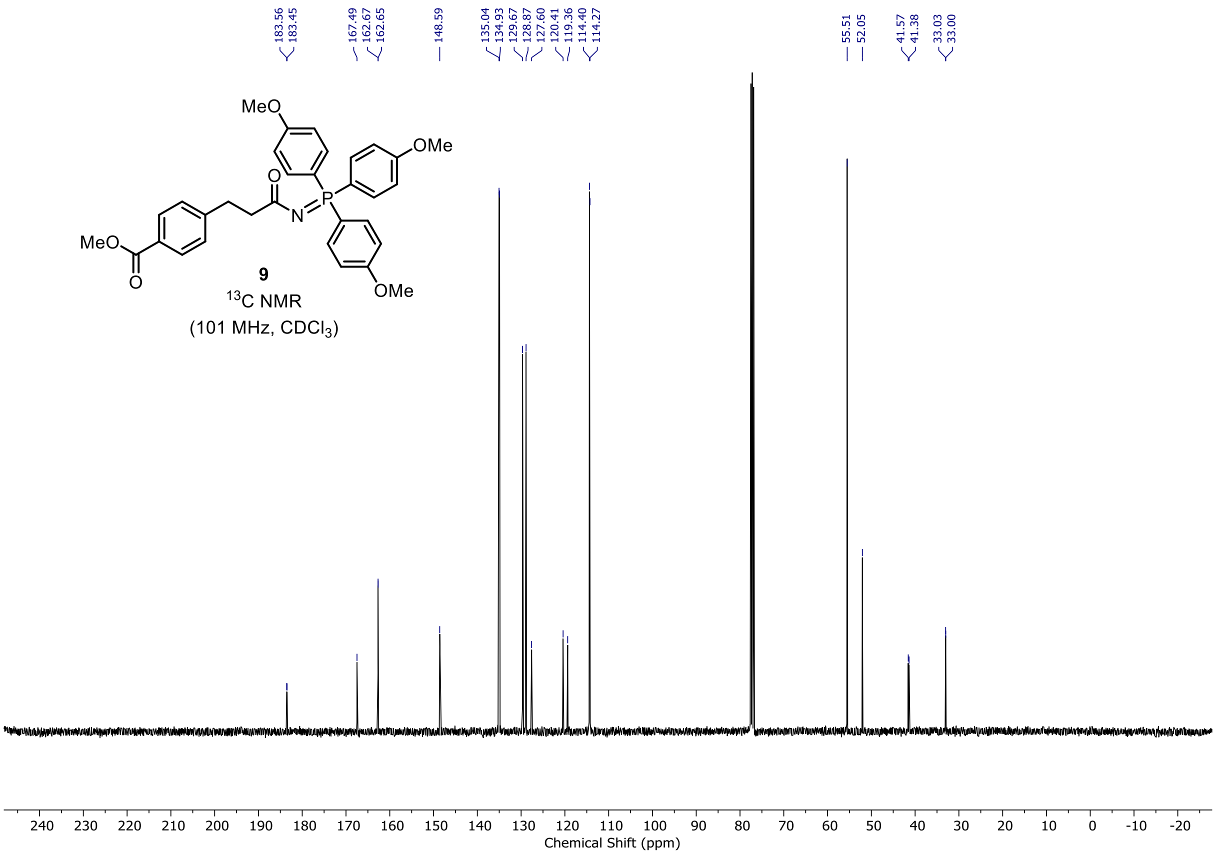
MeO
OMe
O
N
P
MeO
O
OMe
9
^{13}C NMR
(101 MHz, CDCl$_3$)
Chemical Shift (ppm)

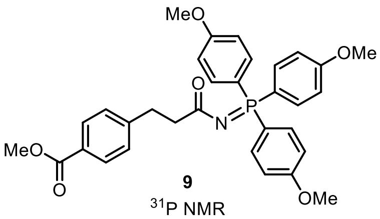

9

^{31}P NMR

(162 MHz, $CDCl_3$)

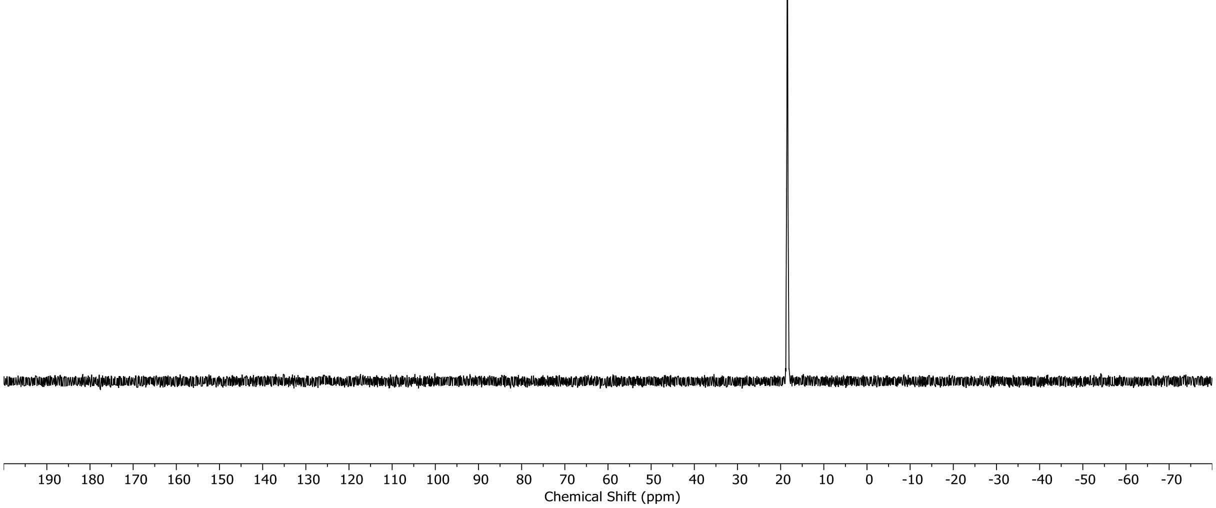

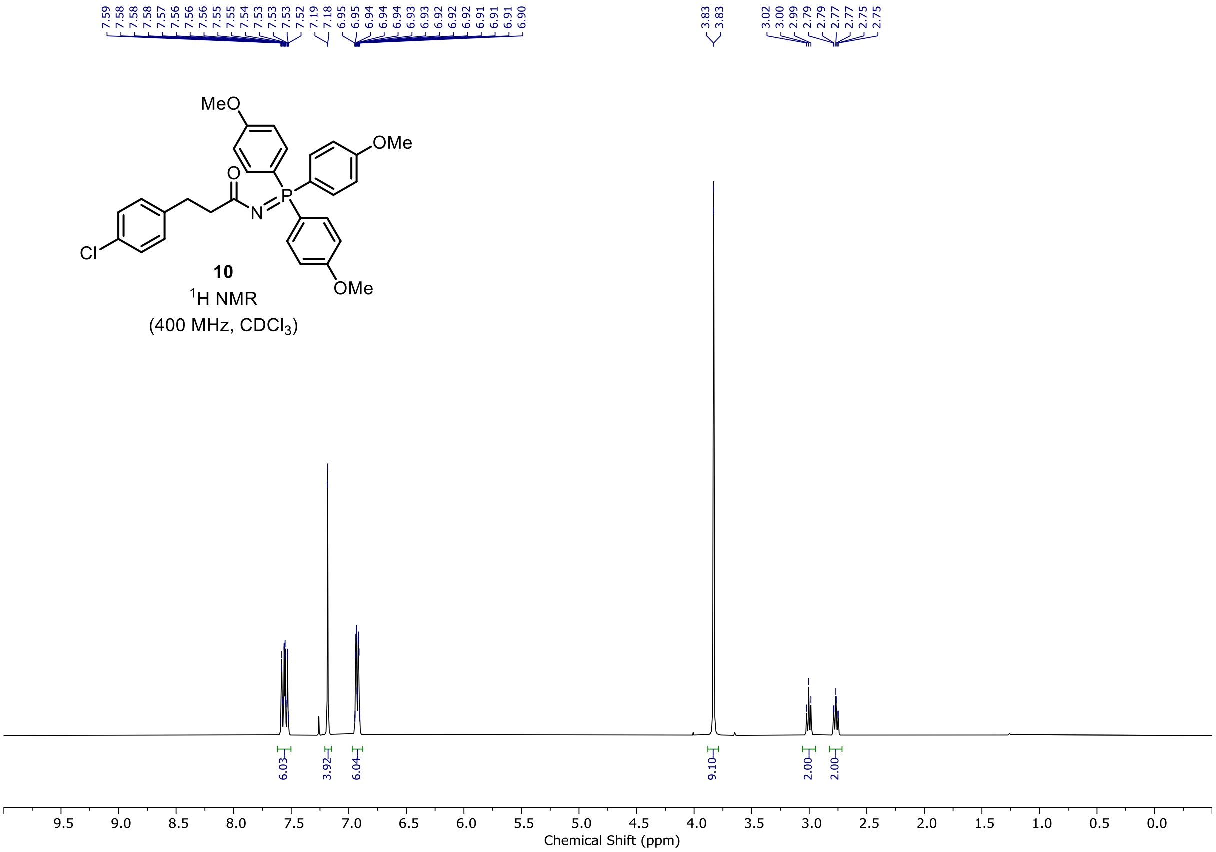
MeO
OMe
O
N
P
Cl
OMe
10
^{1}H NMR
(400 MHz, CDCl$_3$)
Chemical Shift (ppm)

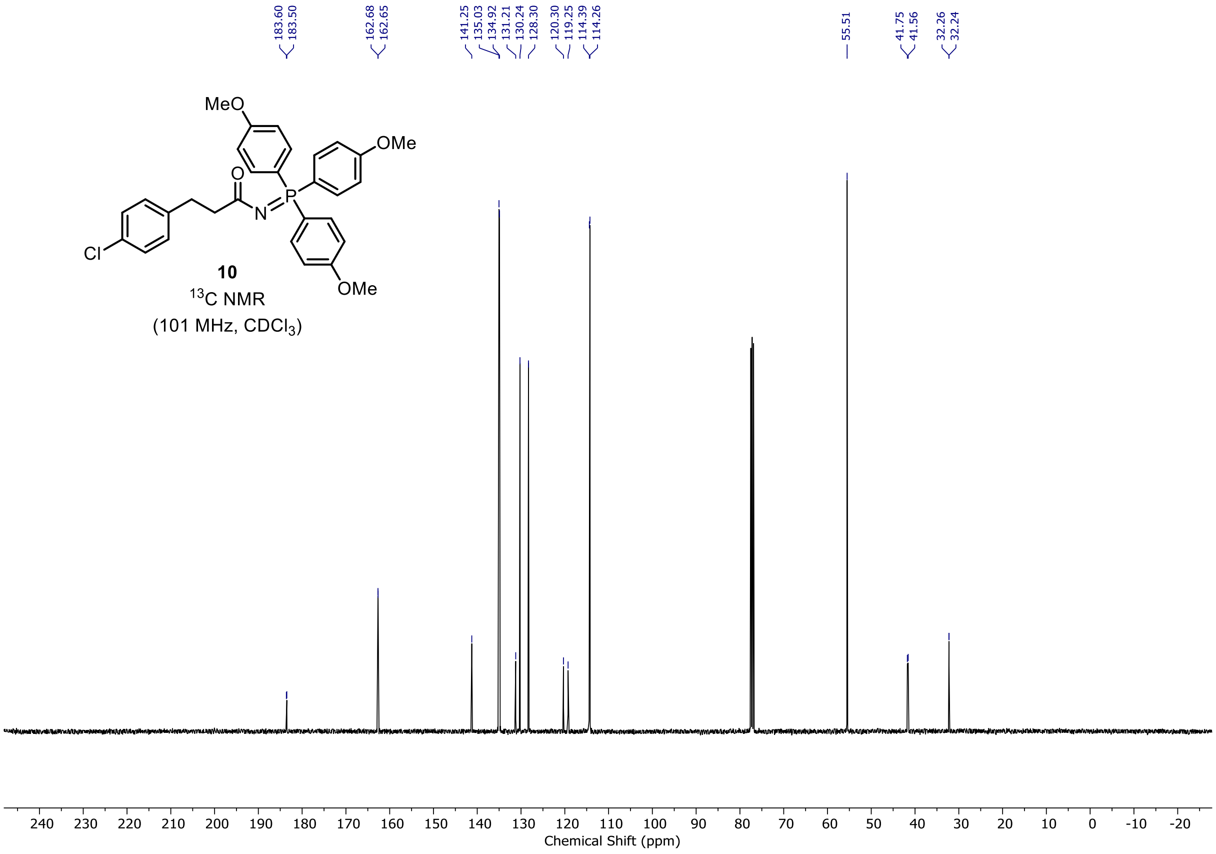
MeO
OMe
O
N
P
Cl
OMe
10
^{13}C NMR
(101 MHz, CDCl$_3$)
Chemical Shift (ppm)

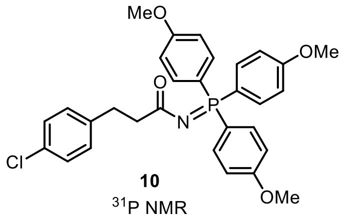

10

^{31}P NMR

(162 MHz, $CDCl_3$)

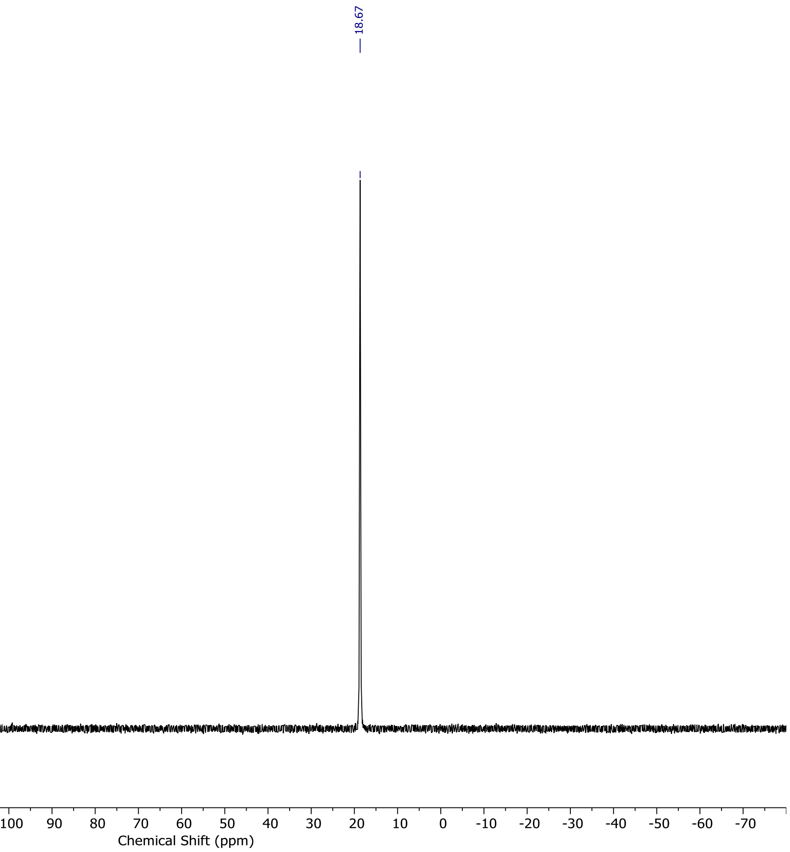

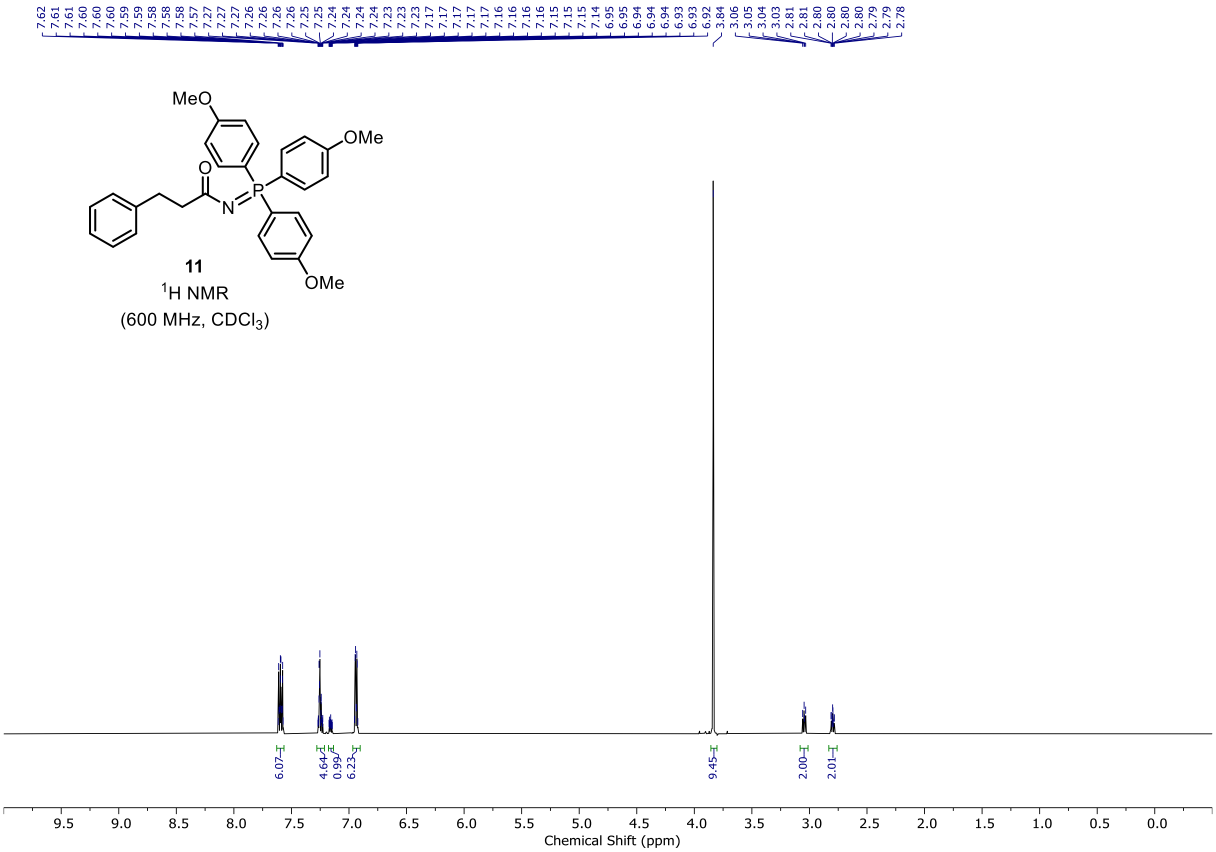
MeO
OMe
O
N
P
OMe
11
1H NMR
(600 MHz, CDCl3)
6.07
4.64
0.99
6.23
9.45
2.00
2.01
Chemical Shift (ppm)

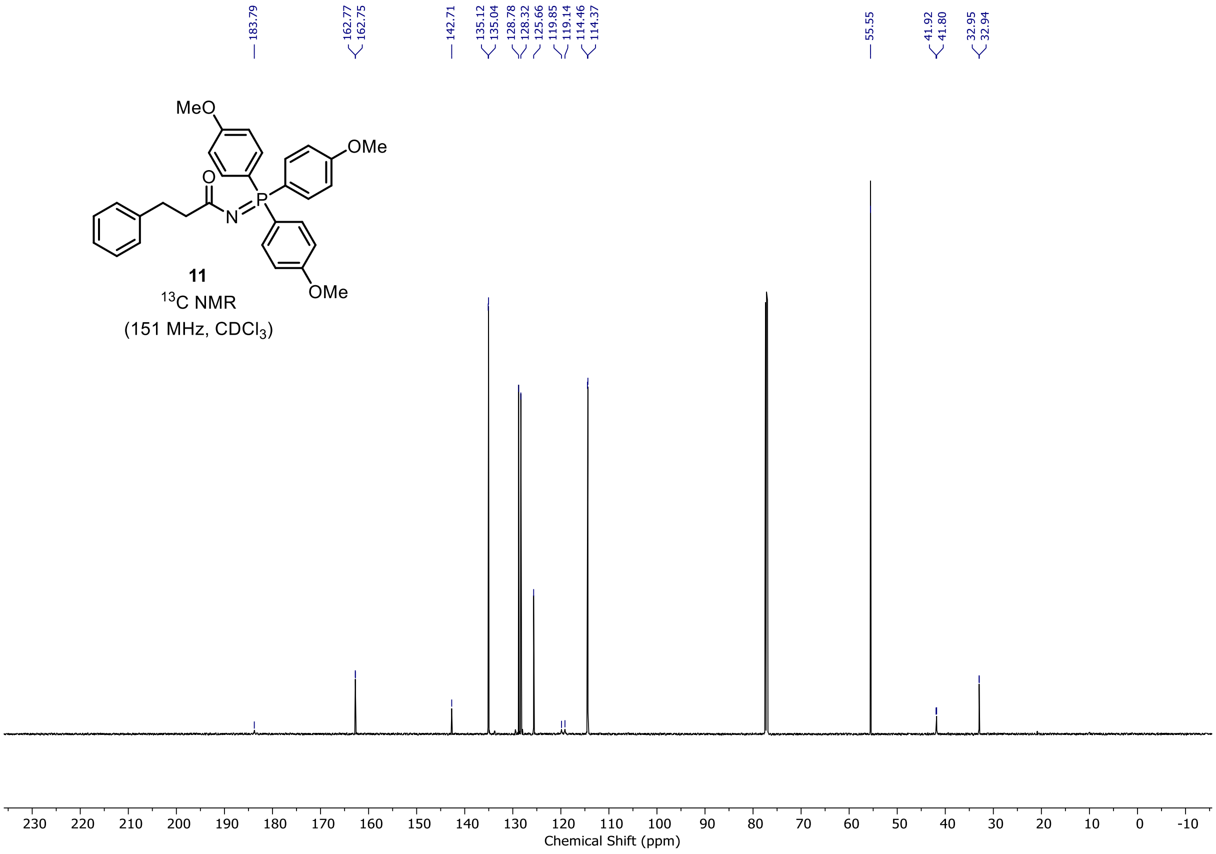
183.79
162.77
162.75
142.71
135.12
135.04
128.78
128.32
125.66
119.85
119.14
114.46
114.37
55.55
41.92
41.80
32.95
32.94
MeO
OMe
O
N
P
OMe
11
13C NMR
(151 MHz, CDCl3)
Chemical Shift (ppm)

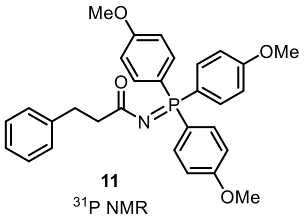

11

^{31}P NMR

(242 MHz, $CDCl_3$)

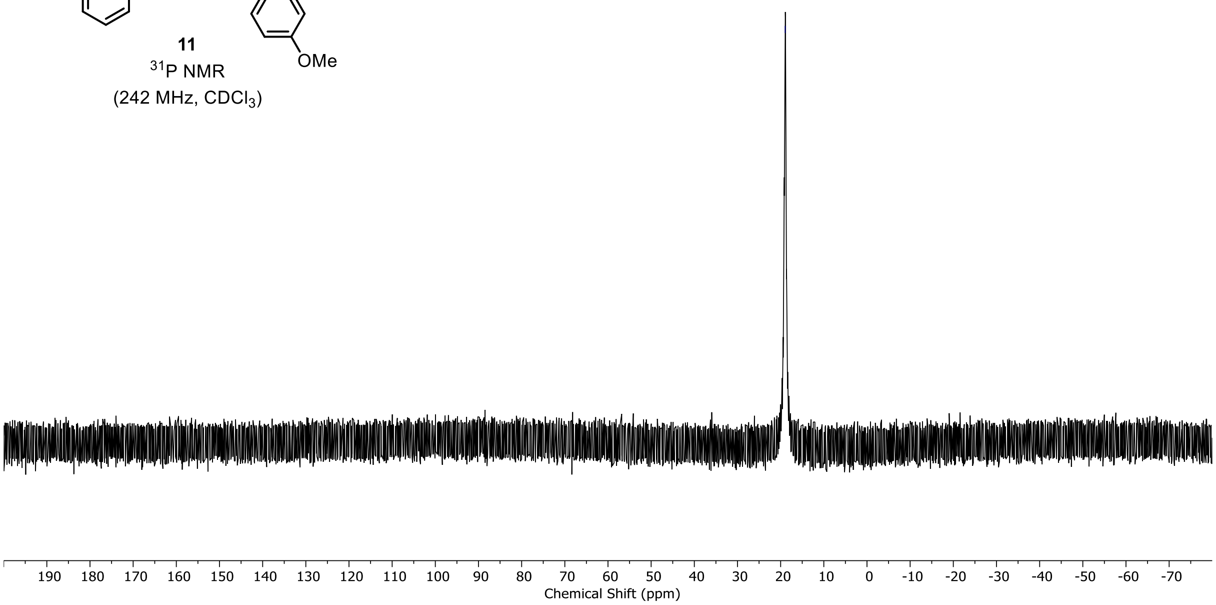

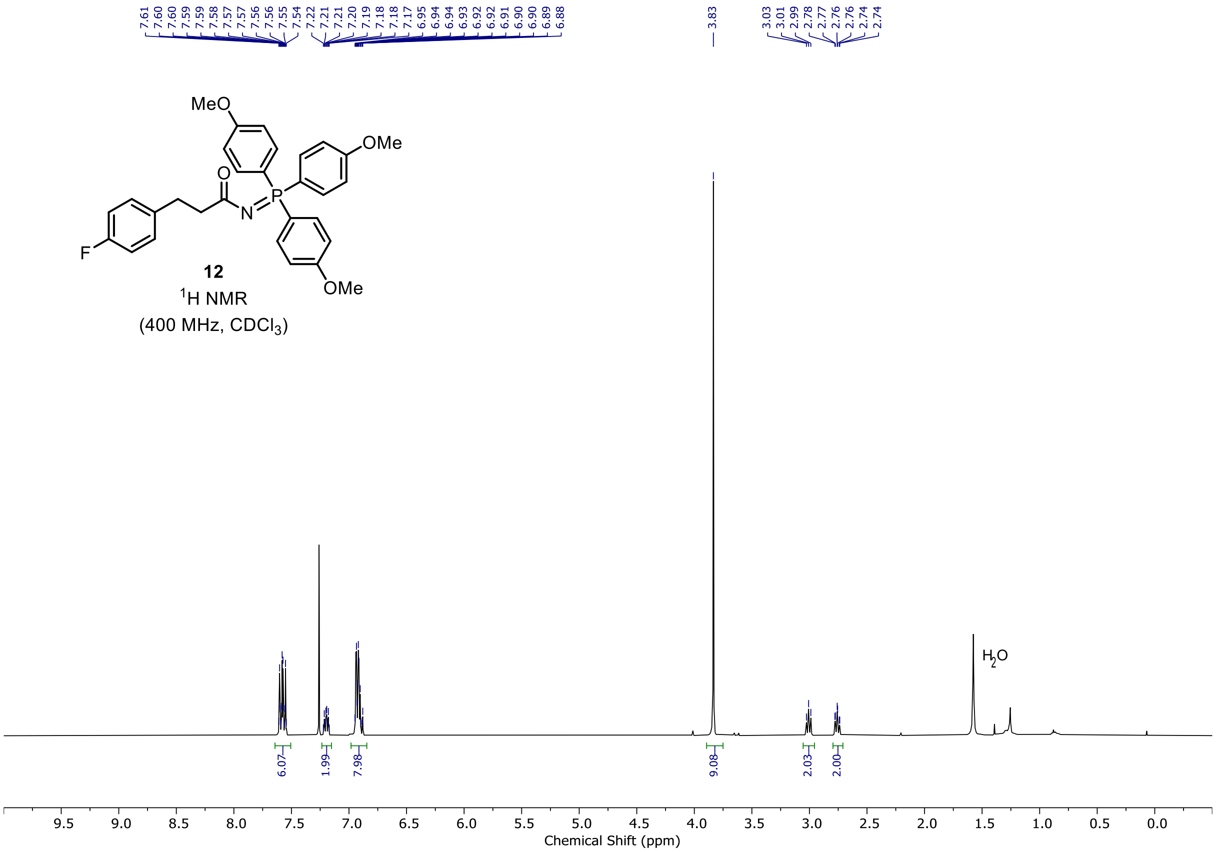
7.61 7.60 7.59 7.59 7.58 7.57 7.57 7.56 7.55 7.54 7.22 7.21 7.21 7.20 7.19 7.18 7.18 7.17 6.95 6.94 6.94 6.93 6.92 6.92 6.91 6.90 6.90 6.89 6.88
3.83
3.03 3.01 2.99 2.78 2.77 2.76 2.76 2.74 2.74
MeO
OMe
OMe
O
N
P
F
12
^{1}H NMR
(400 MHz, CDCl$_3$)
H$_2$O
6.07 1.99 7.98 9.08 2.03 2.00
9.5 9.0 8.5 8.0 7.5 7.0 6.5 6.0 5.5 5.0 4.5 4.0 3.5 3.0 2.5 2.0 1.5 1.0 0.5 0.0
Chemical Shift (ppm)

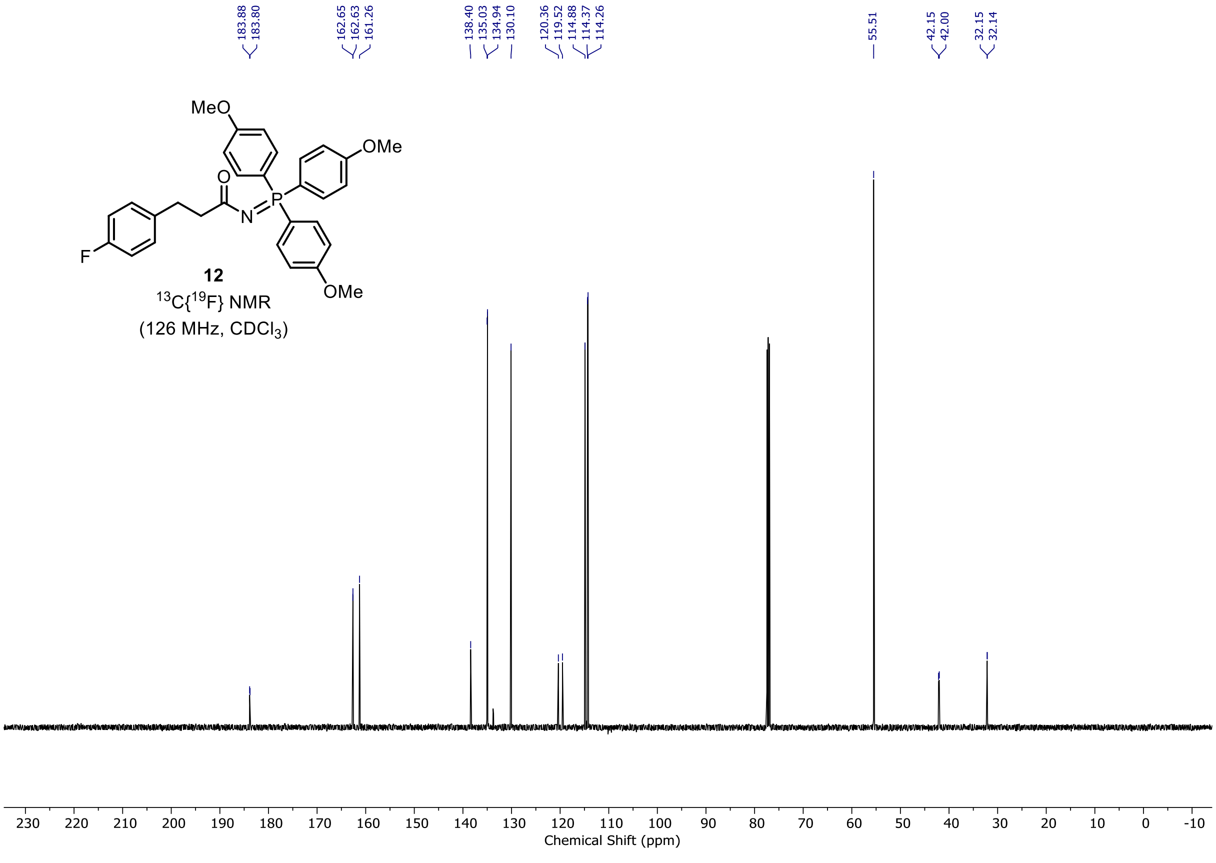
183.88 183.80
162.65 162.63 161.26
138.40 135.03 134.94 130.10
120.36 119.52 114.88 114.37 114.26
55.51
42.15 42.00
32.15 32.14
MeO
OMe
OMe
O
N
P
F
12
^{13}C{^{19}F} NMR
(126 MHz, CDCl$_3$)
230 220 210 200 190 180 170 160 150 140 130 120 110 100 90 80 70 60 50 40 30 20 10 0 -10
Chemical Shift (ppm)

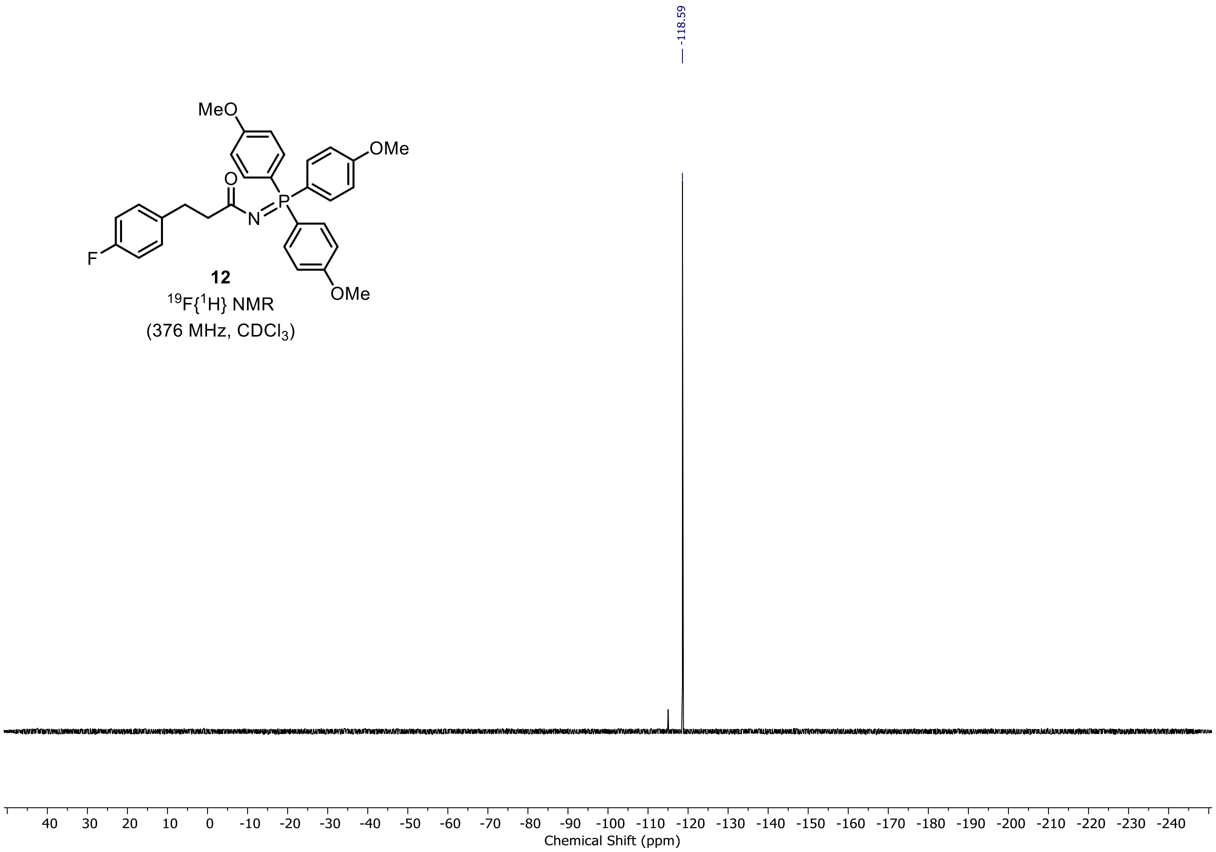

12

$^{19}F\{^1H\}$ NMR

(376 MHz, $CDCl_3$)

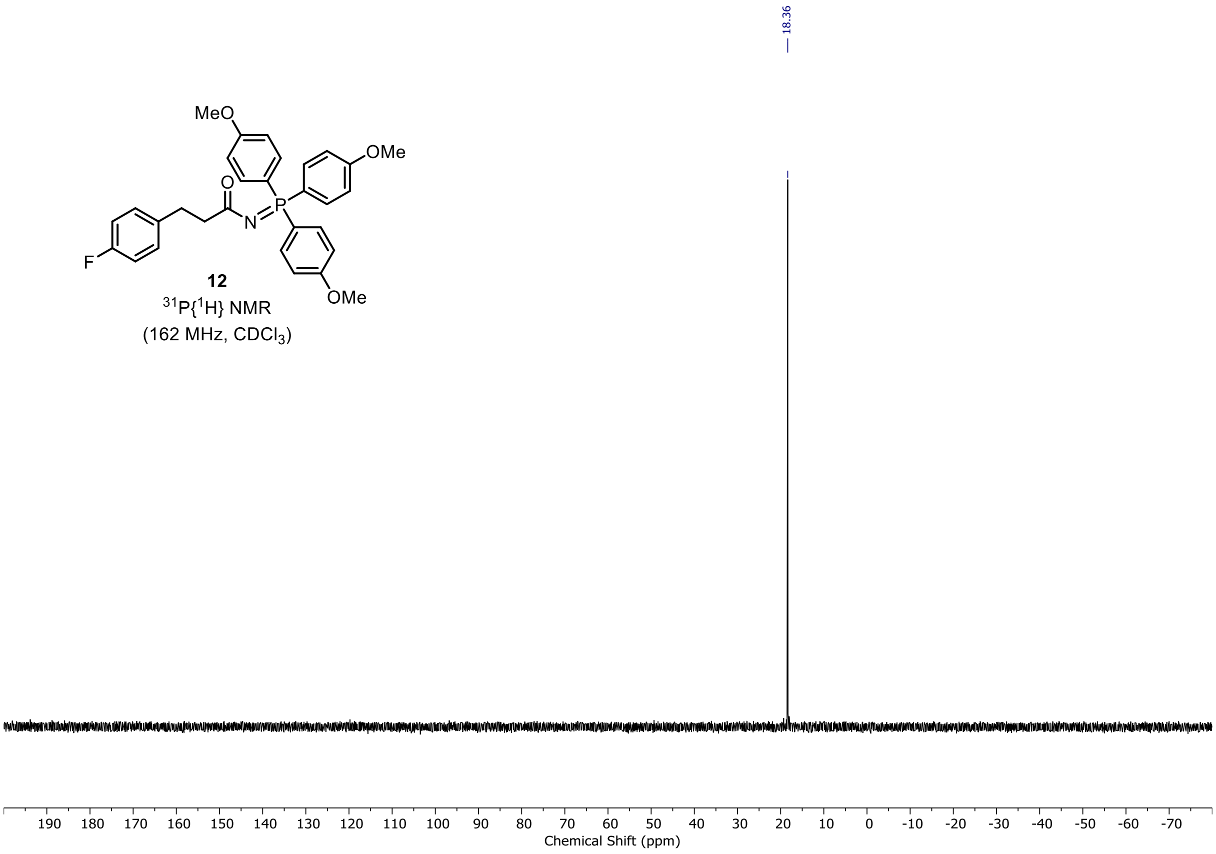

12

$^{31}P\{^1H\}$ NMR

(162 MHz, $CDCl_3$)

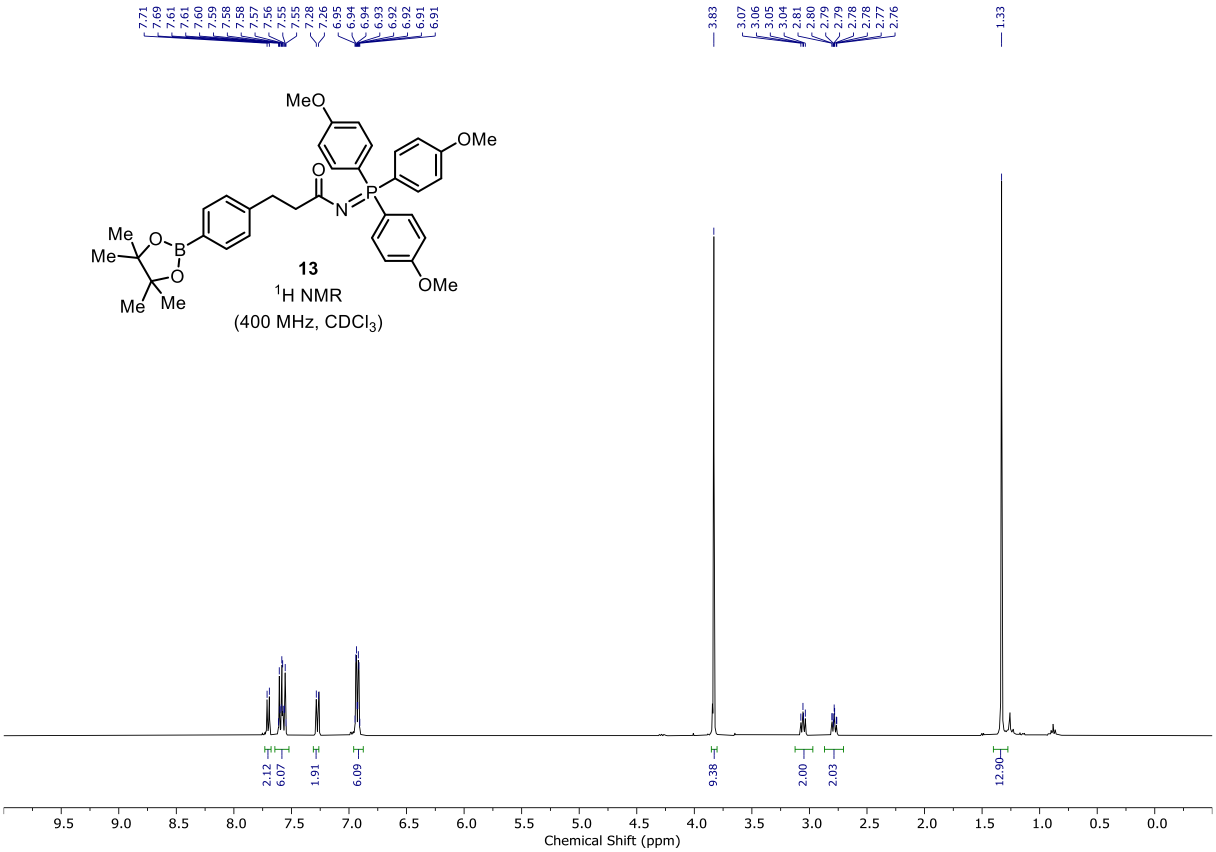
7.71 7.69 7.61 7.61 7.60 7.59 7.58 7.58 7.57 7.56 7.55 7.55 7.28 7.26 6.95 6.94 6.94 6.93 6.93 6.92 6.92 6.91 6.91
3.83
3.07 3.06 3.05 3.04 2.81 2.80 2.79 2.79 2.78 2.78 2.77 2.76
1.33
MeO
OMe
O
N
P
B
O
Me
Me
Me
Me
OMe
13
^{1}H NMR
(400 MHz, $CDCl_3$)
2.12 6.07 1.91 6.09 9.38 2.00 2.03 12.90
9.5 9.0 8.5 8.0 7.5 7.0 6.5 6.0 5.5 5.0 4.5 4.0 3.5 3.0 2.5 2.0 1.5 1.0 0.5 0.0
Chemical Shift (ppm)

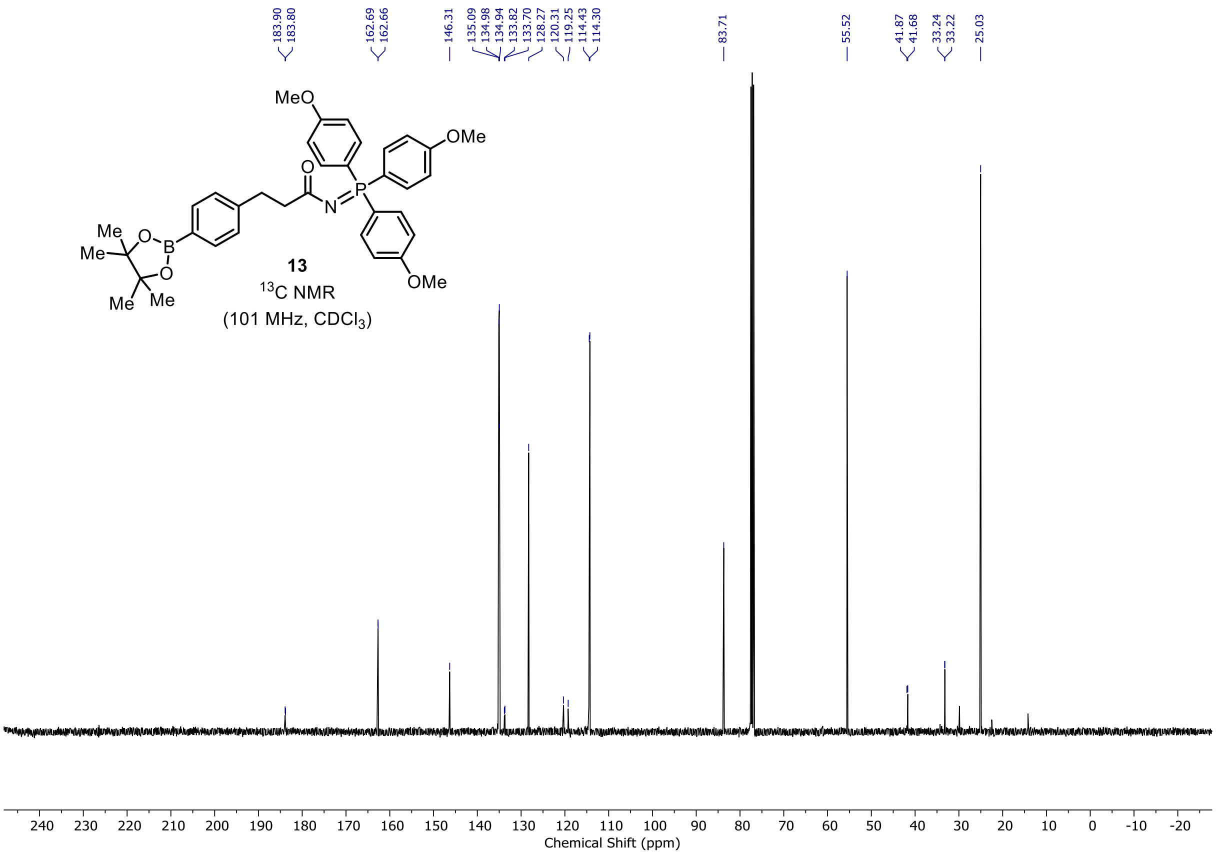
183.90 183.80
162.69 162.66
146.31
135.09 134.98 134.94 133.82 133.70 128.27 120.31 119.25 114.43 114.30
83.71
55.52
41.87 41.68 33.24 33.22 25.03
MeO
OMe
O
N
P
B
O
Me
Me
Me
Me
OMe
13
^{13}C NMR
(101 MHz, $CDCl_3$)
240 230 220 210 200 190 180 170 160 150 140 130 120 110 100 90 80 70 60 50 40 30 20 10 0 -10 -20
Chemical Shift (ppm)

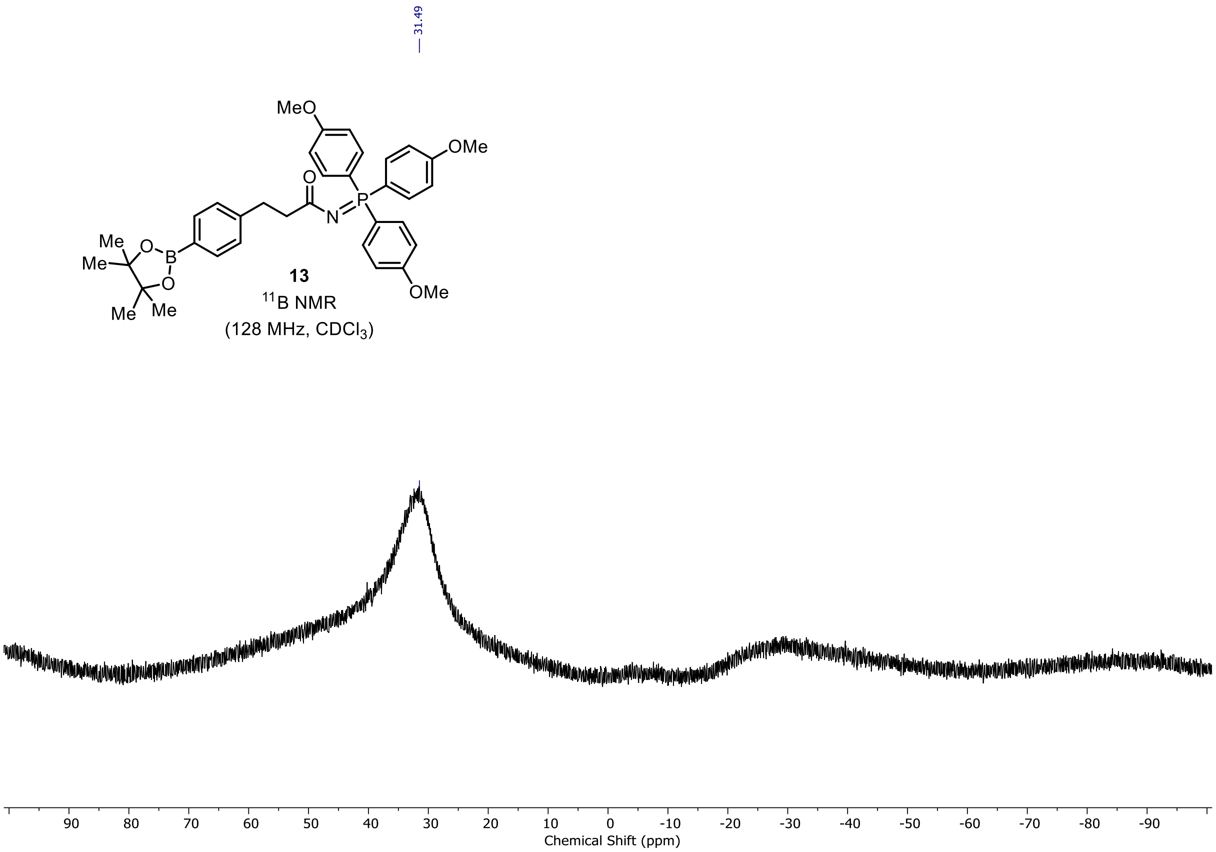

13
^{11}B NMR
(128 MHz, $CDCl_3$)

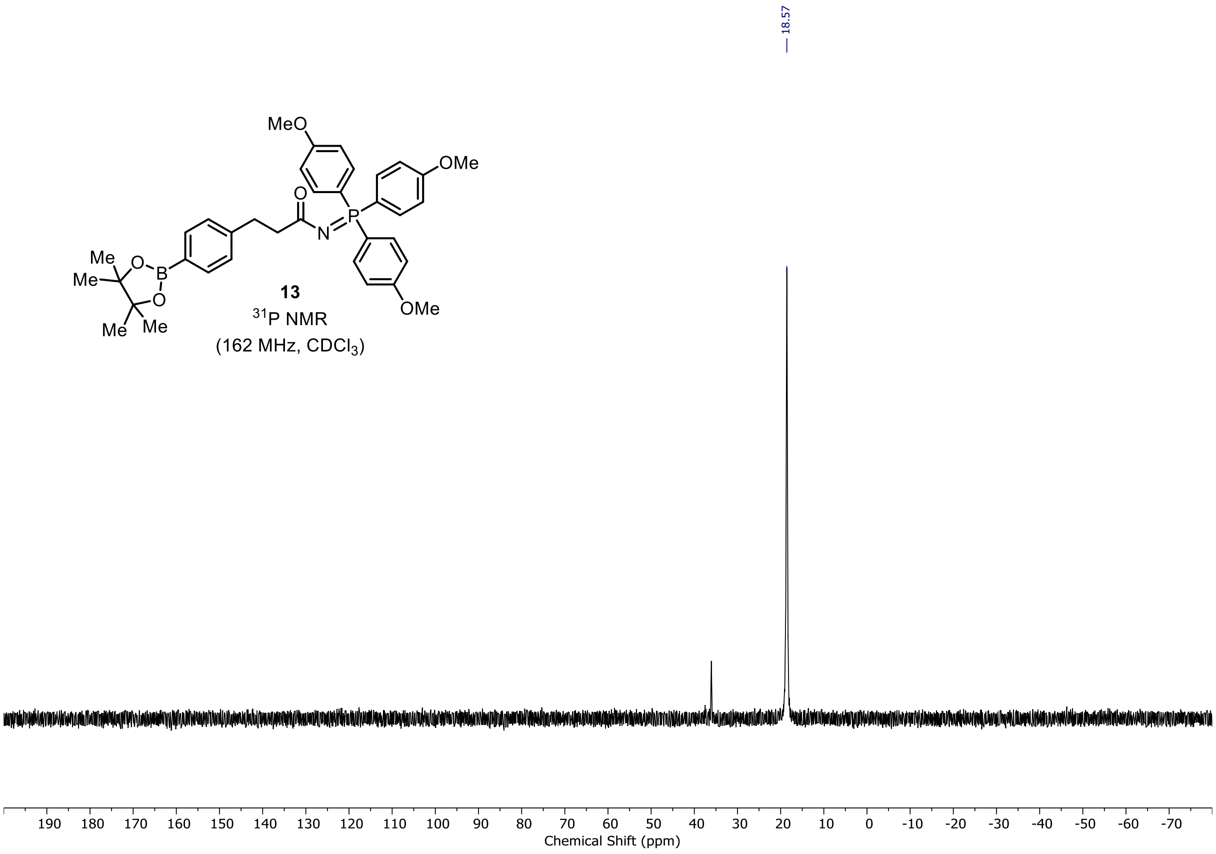

13
^{31}P NMR
(162 MHz, $CDCl_3$)

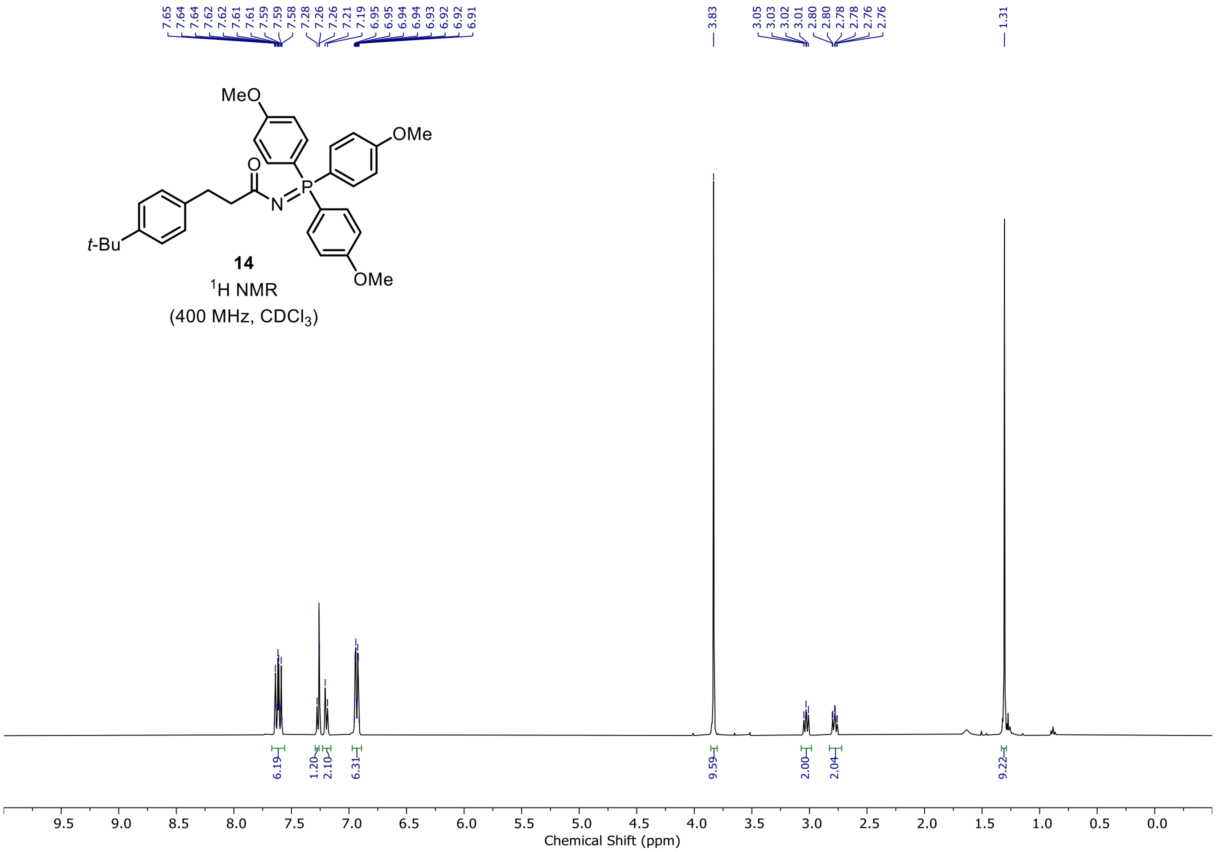
7.65
7.64
7.62
7.62
7.61
7.61
7.59
7.59
7.58
7.28
7.26
7.26
7.21
7.19
6.95
6.95
6.94
6.94
6.93
6.92
6.92
6.91
3.83
3.05
3.03
3.02
3.01
2.80
2.80
2.78
2.78
2.76
2.76
1.31
MeO
OMe
OMe
t-Bu
14
^{1}H NMR
(400 MHz, CDCl$_3$)
6.19
1.20
2.10
6.31
9.59
2.00
2.04
9.22
Chemical Shift (ppm)

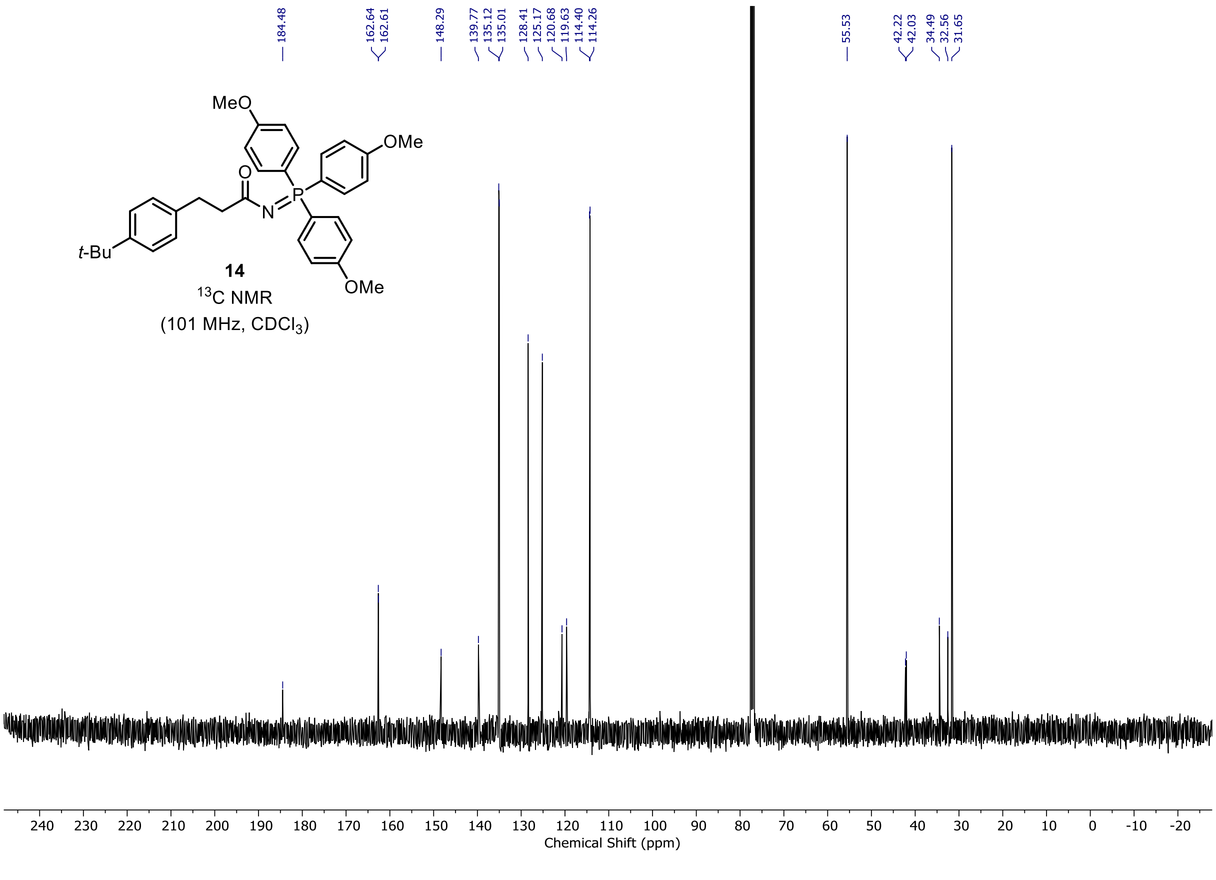
184.48
162.64
162.61
148.29
139.77
135.12
135.01
128.41
125.17
120.68
119.63
114.40
114.26
55.53
42.22
42.03
34.49
32.56
31.65
MeO
OMe
OMe
t-Bu
14
^{13}C NMR
(101 MHz, CDCl$_3$)
Chemical Shift (ppm)

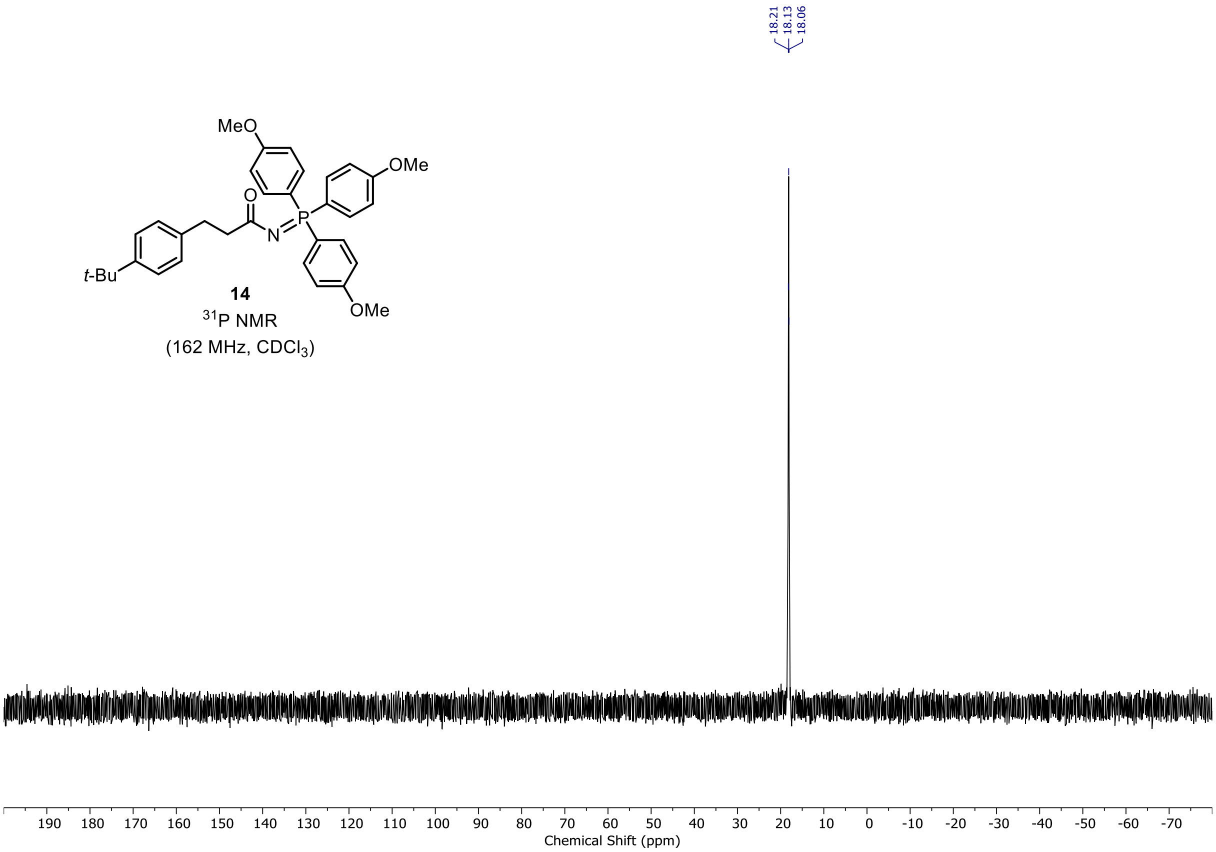

14
^{31}P NMR
(162 MHz, $CDCl_3$)

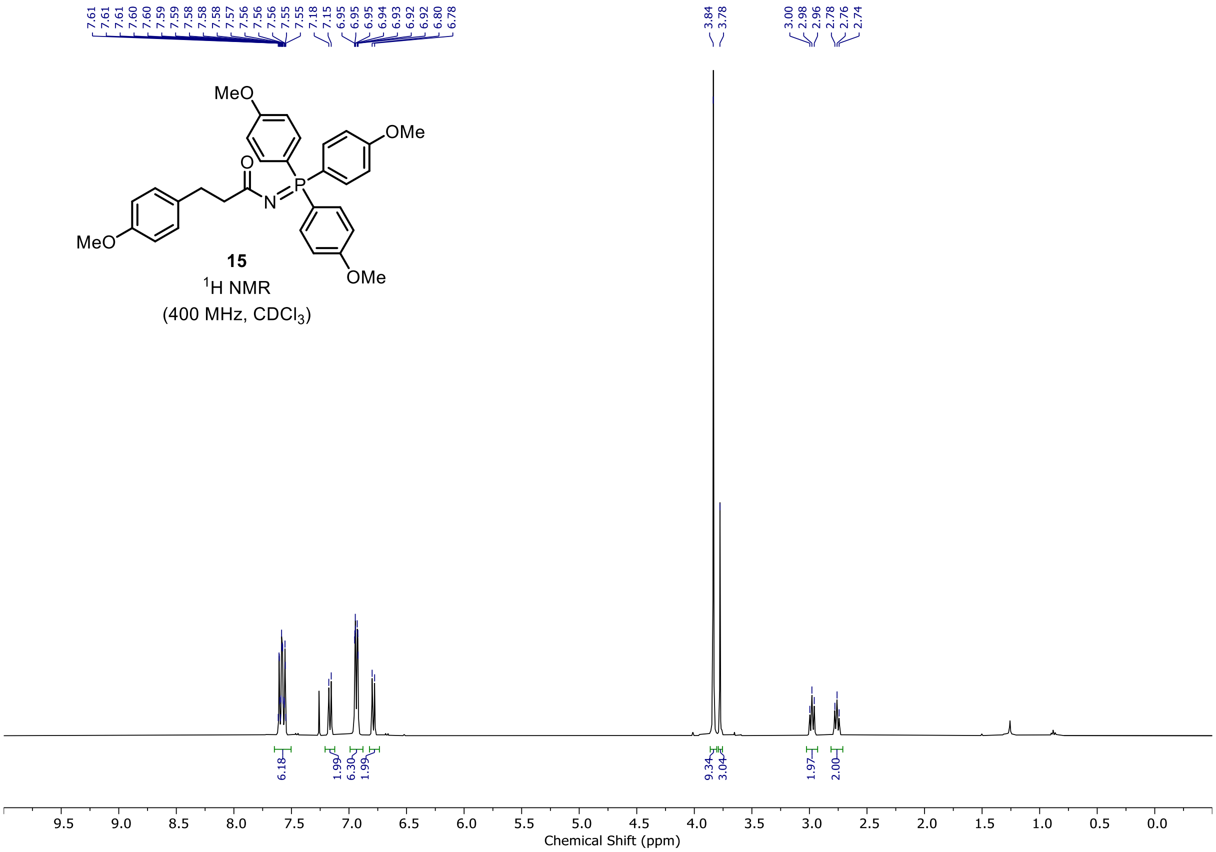
7.61
7.61
7.61
7.60
7.59
7.59
7.58
7.58
7.58
7.57
7.56
7.56
7.55
7.55
7.18
7.15
6.95
6.95
6.95
6.94
6.93
6.92
6.92
6.80
6.78
3.84
3.78
3.00
2.98
2.96
2.78
2.76
2.74
MeO
OMe
O
N
P
MeO
OMe
15
^{1}H NMR
(400 MHz, $CDCl_3$)
6.18
1.99
6.30
1.99
9.34
3.04
1.97
2.00
Chemical Shift (ppm)

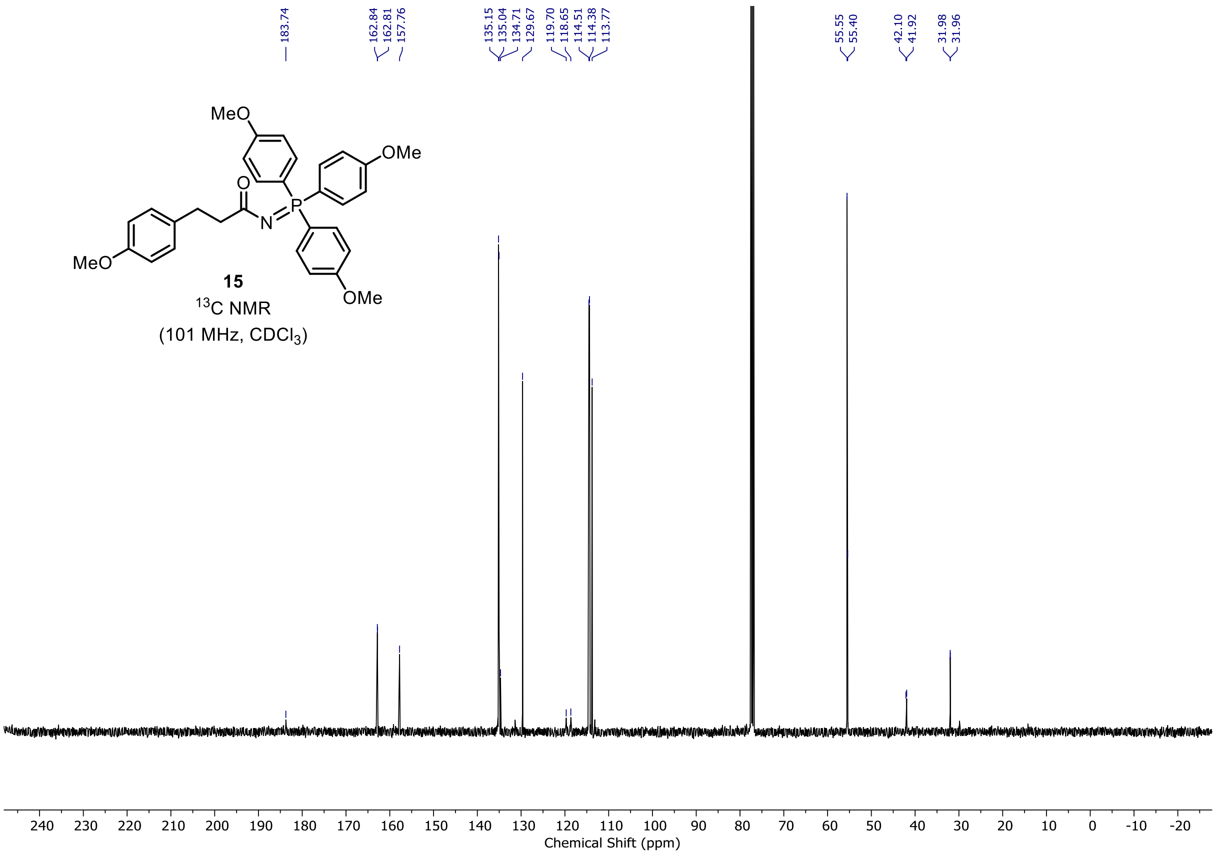
183.74
162.84
162.81
157.76
135.15
135.04
134.71
129.67
119.70
118.65
114.51
114.38
113.77
55.55
55.40
42.10
41.92
31.98
31.96
MeO
OMe
O
N
P
MeO
OMe
15
^{13}C NMR
(101 MHz, $CDCl_3$)
Chemical Shift (ppm)

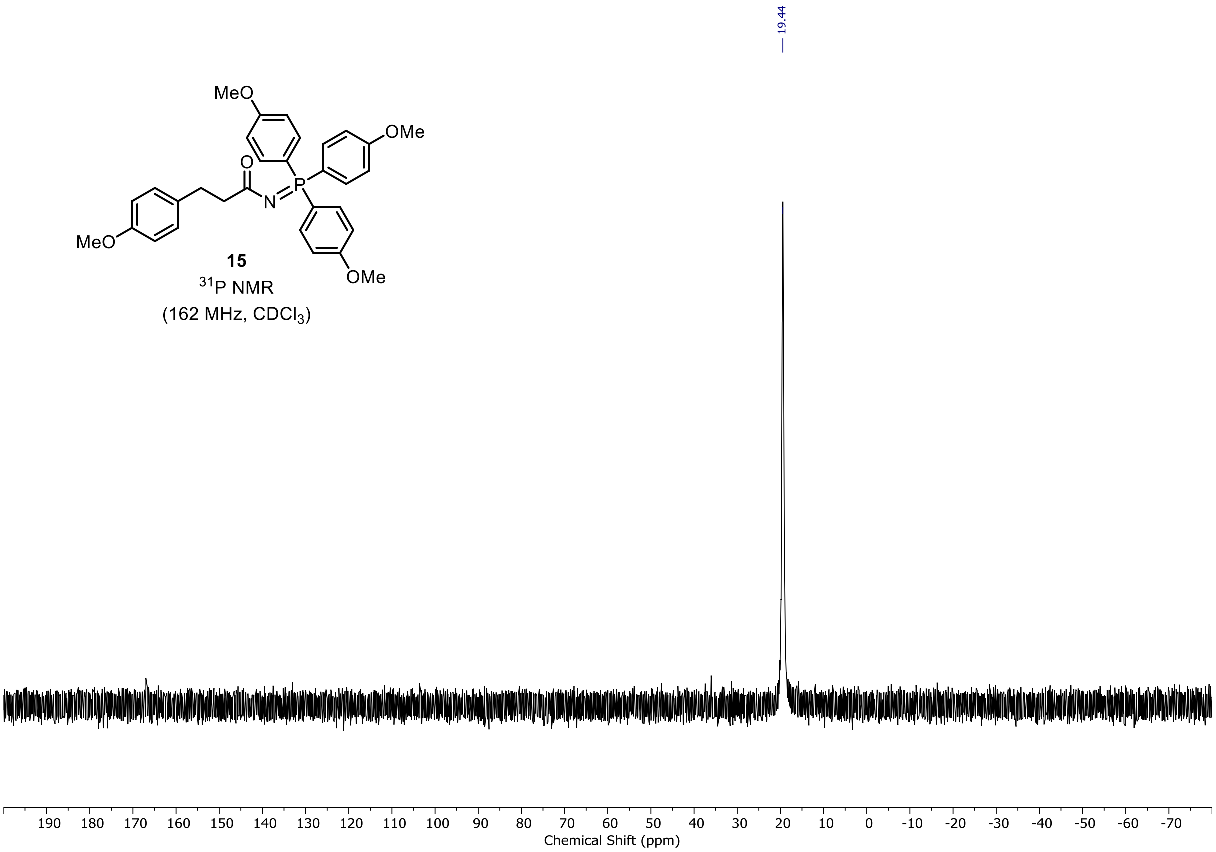

15
^{31}P NMR
(162 MHz, $CDCl_3$)

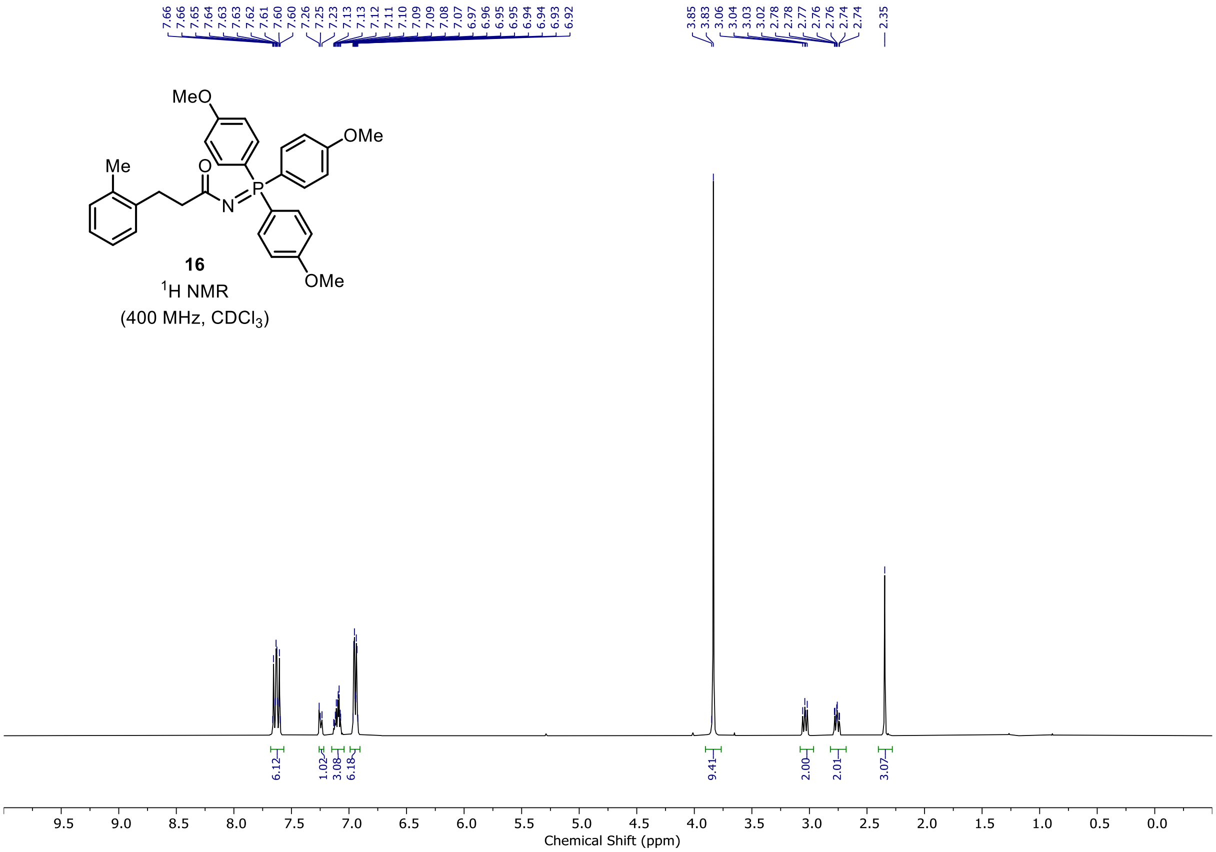
7.66 7.66 7.65 7.64 7.63 7.63 7.62 7.61 7.60 7.60 7.26 7.25 7.23 7.13 7.13 7.12 7.11 7.10 7.09 7.09 7.08 7.07 6.97 6.96 6.95 6.95 6.94 6.94 6.93 6.92
3.85 3.83 3.06 3.04 3.03 3.02 2.78 2.78 2.77 2.76 2.74 2.74
2.35
MeO
OMe
Me
O
N
P
OMe
16
1H NMR
(400 MHz, CDCl3)
6.12 1.02 3.08 6.18 9.41 2.00 2.01 3.07
9.5 9.0 8.5 8.0 7.5 7.0 6.5 6.0 5.5 5.0 4.5 4.0 3.5 3.0 2.5 2.0 1.5 1.0 0.5 0.0
Chemical Shift (ppm)

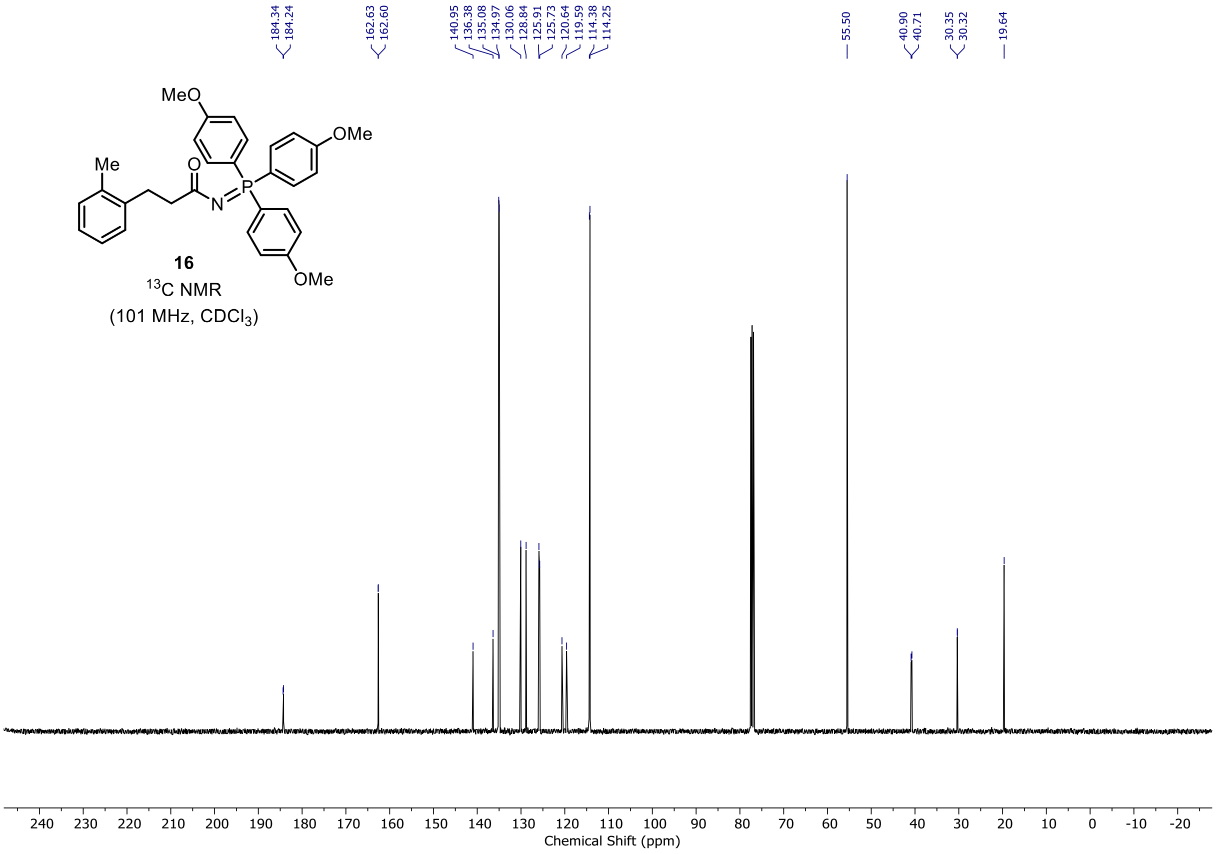
184.34 184.24
162.63 162.60
140.95 136.38 135.08 134.97 130.06 128.84 125.91 125.73 120.64 119.59 114.38 114.25
55.50
40.90 40.71
30.35 30.32
19.64
MeO
OMe
Me
O
N
P
OMe
16
13C NMR
(101 MHz, CDCl3)
240 230 220 210 200 190 180 170 160 150 140 130 120 110 100 90 80 70 60 50 40 30 20 10 0 -10 -20
Chemical Shift (ppm)

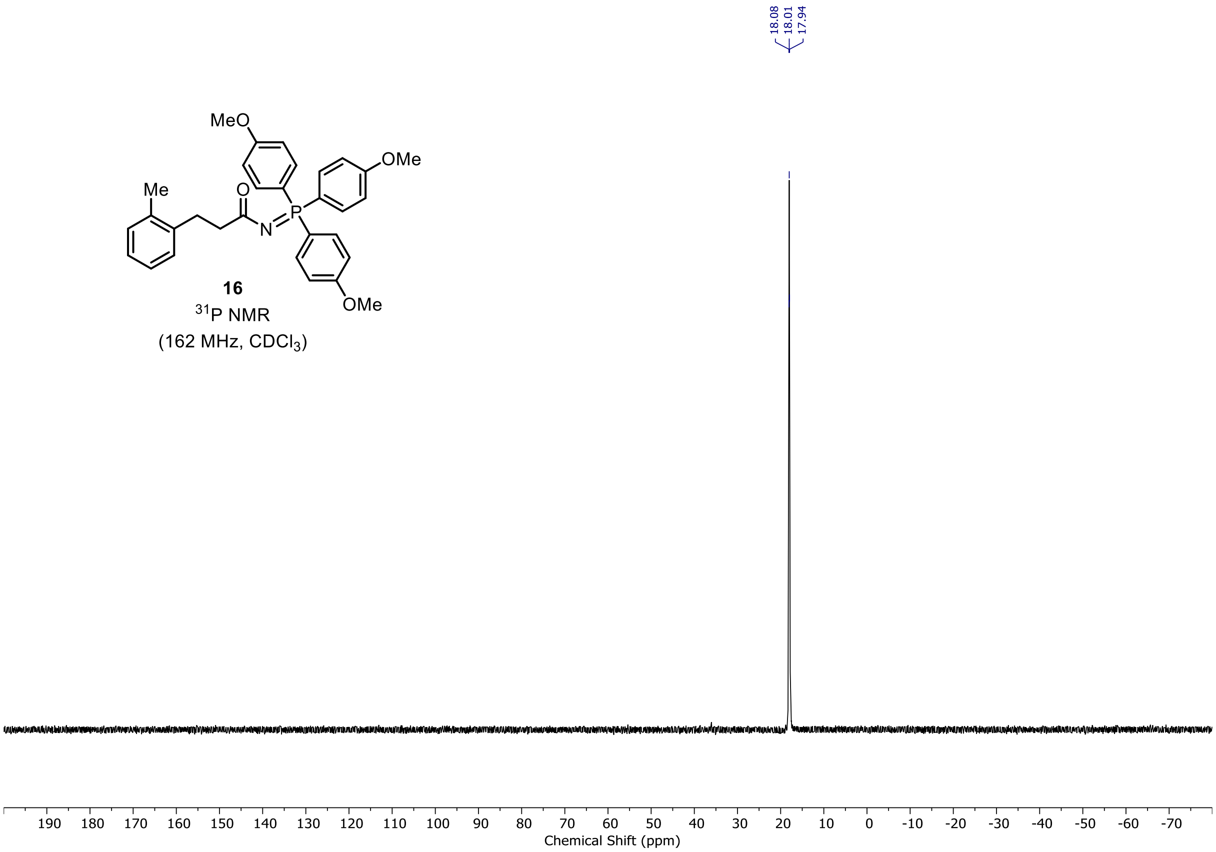

16
^{31}P NMR
(162 MHz, $CDCl_3$)

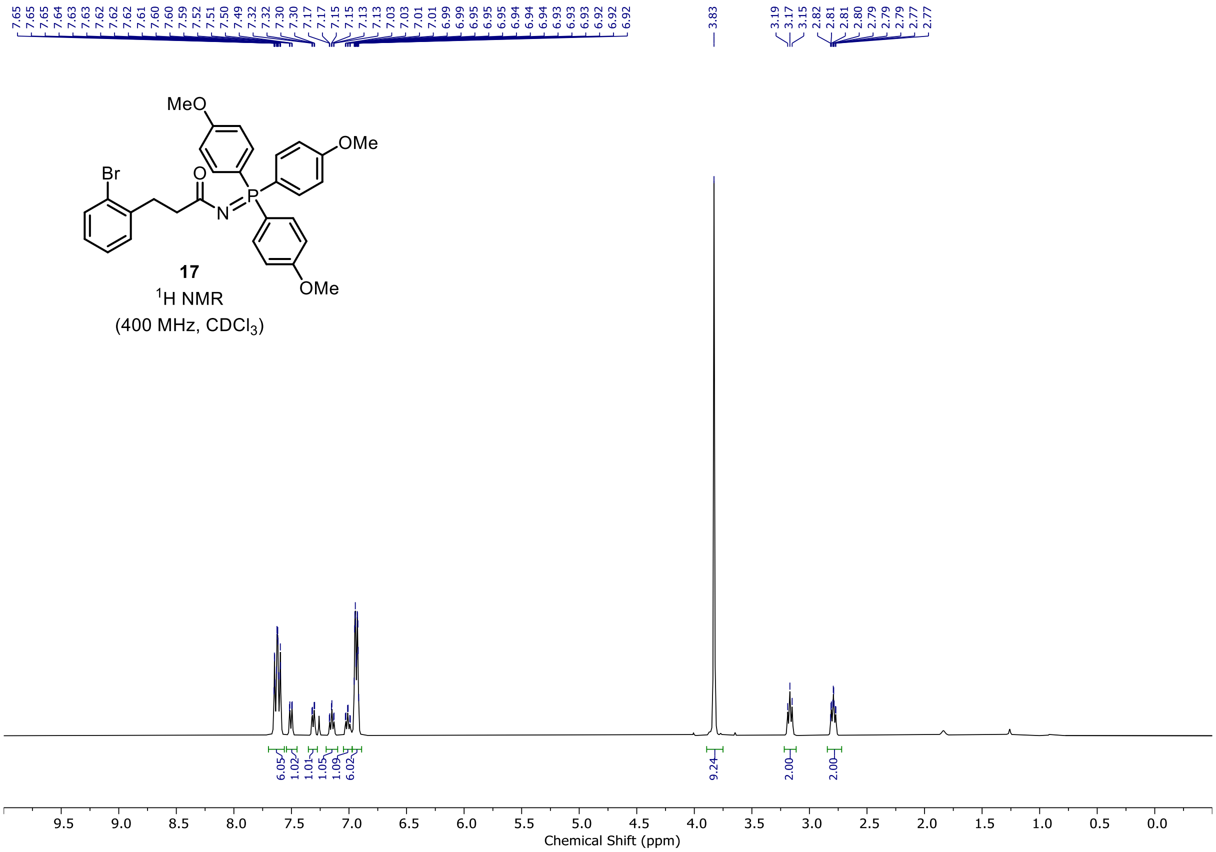
17
1H NMR
(400 MHz, CDCl3)
Chemical Shift (ppm)

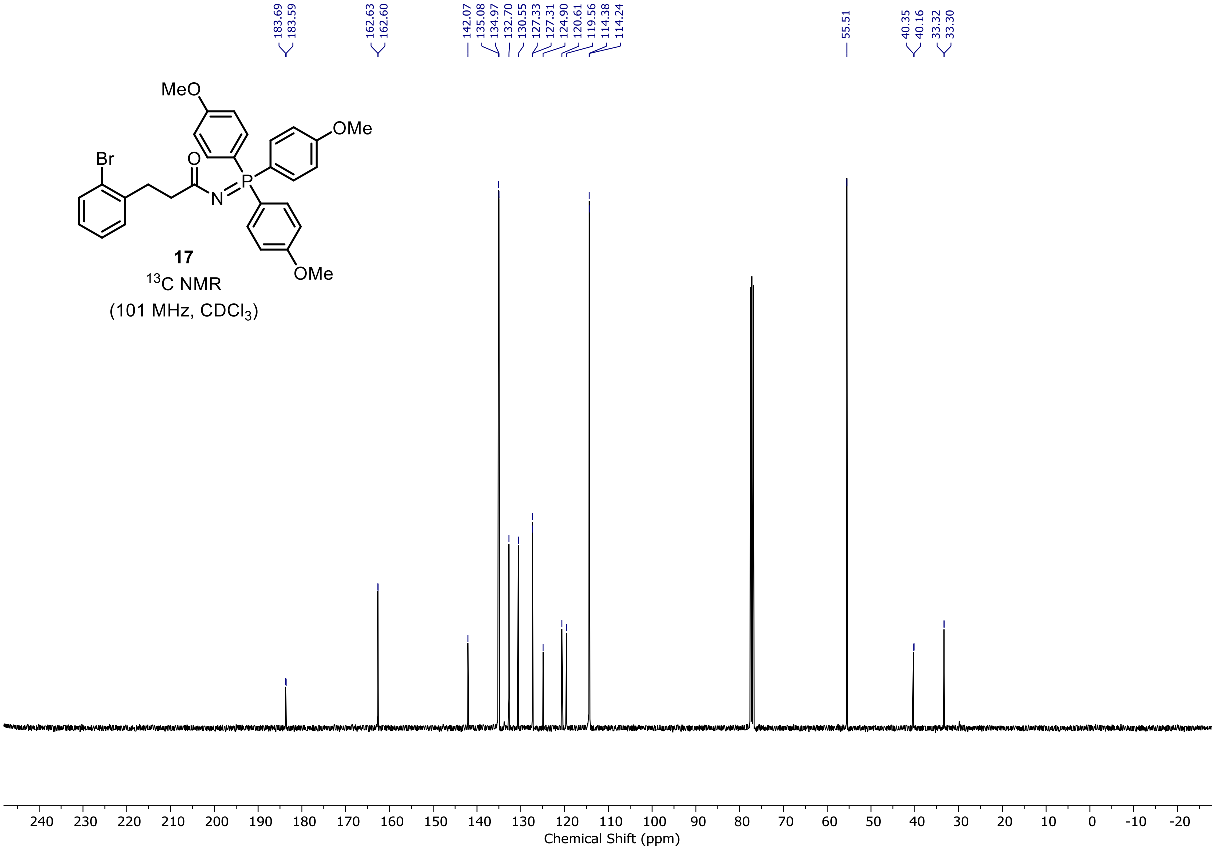
17
13C NMR
(101 MHz, CDCl3)
Chemical Shift (ppm)

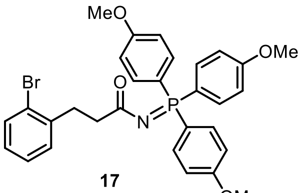

17
^{31}P NMR
(162 MHz, $CDCl_3$)

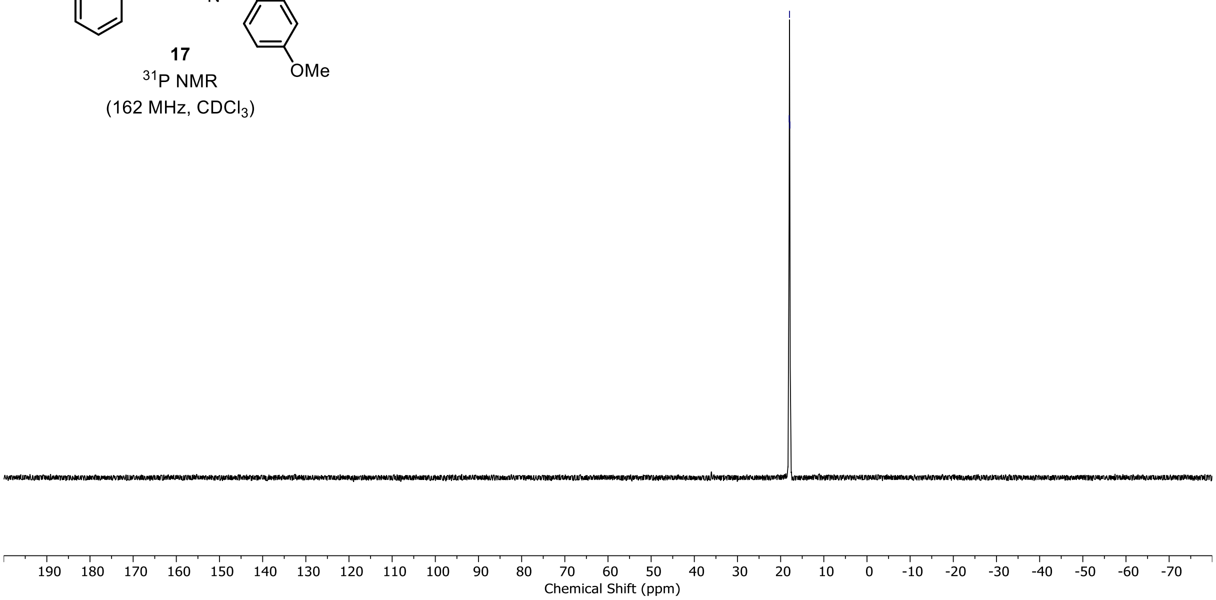

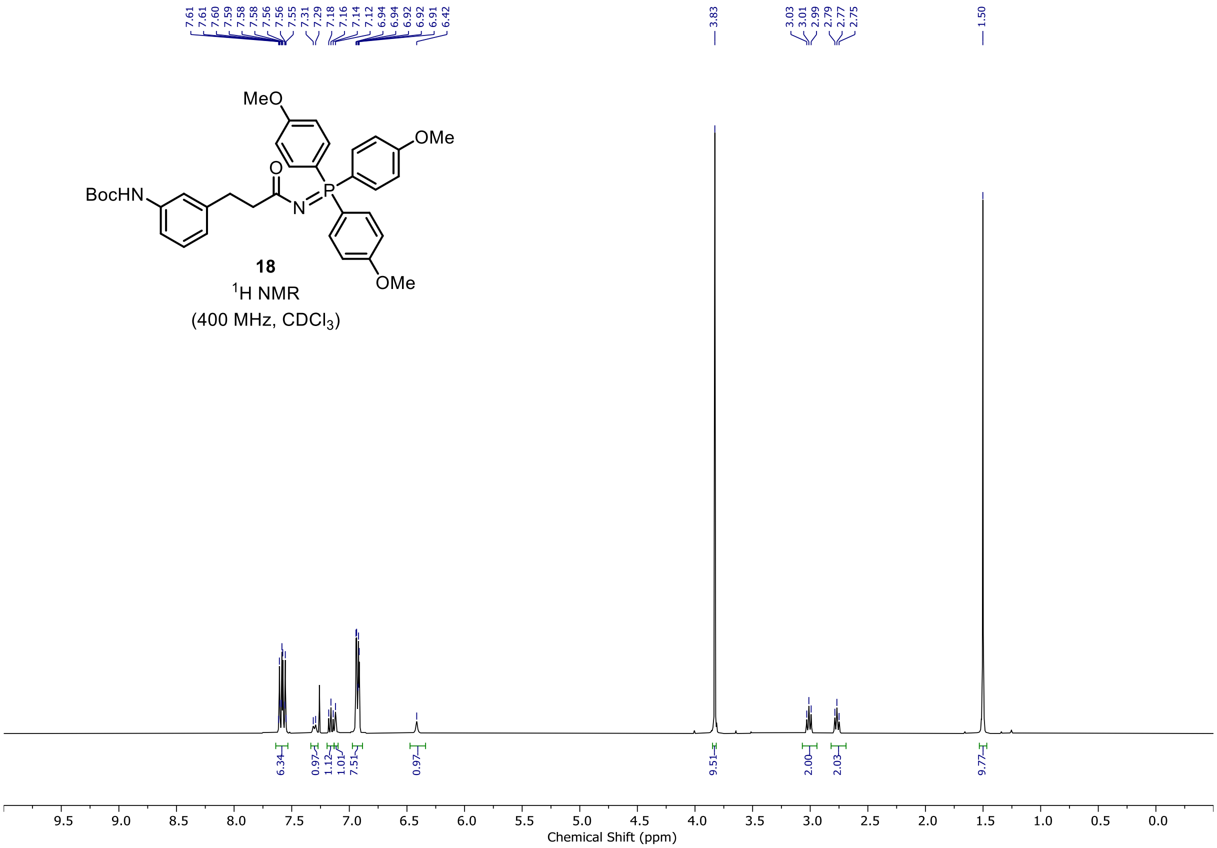
BocHN
MeO
OMe
OMe
O
N
P
18
1H NMR
(400 MHz, CDCl3)
Chemical Shift (ppm)

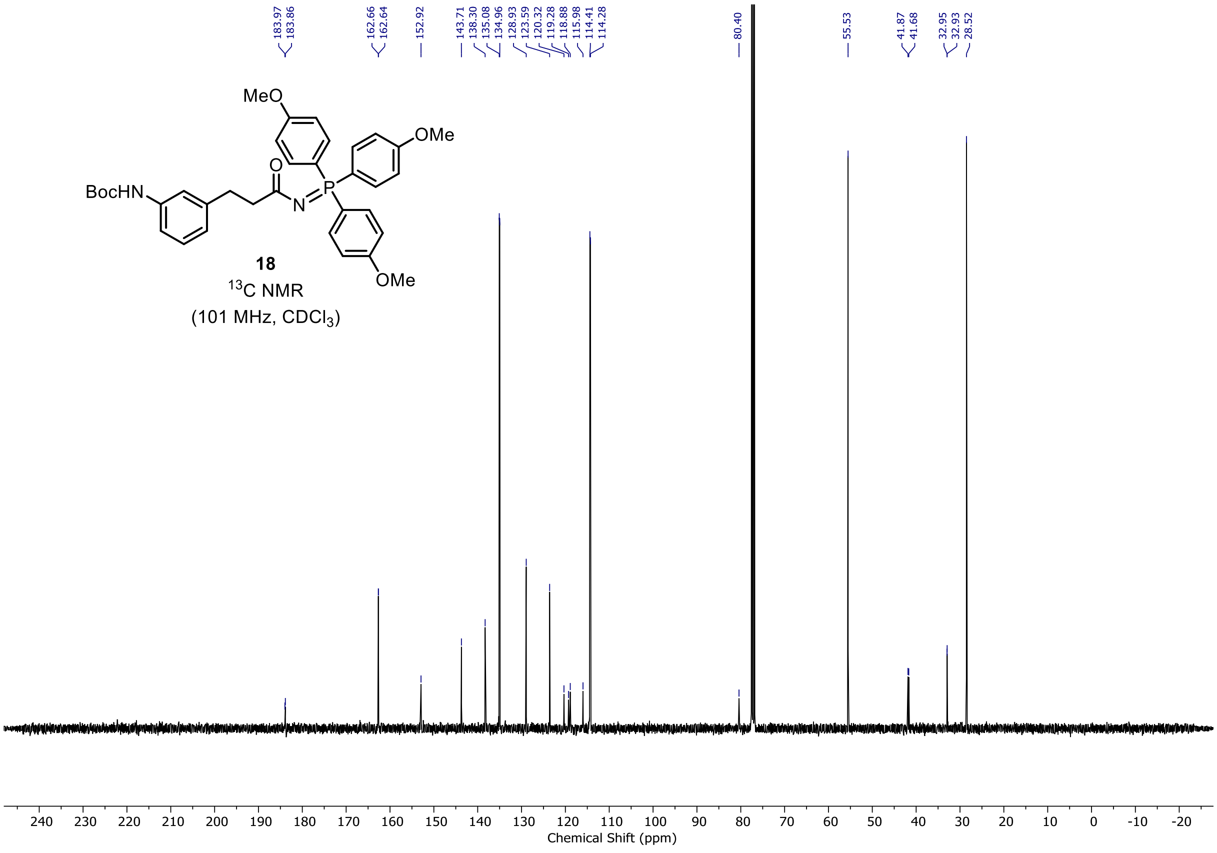
BocHN
MeO
OMe
OMe
O
N
P
18
13C NMR
(101 MHz, CDCl3)
Chemical Shift (ppm)

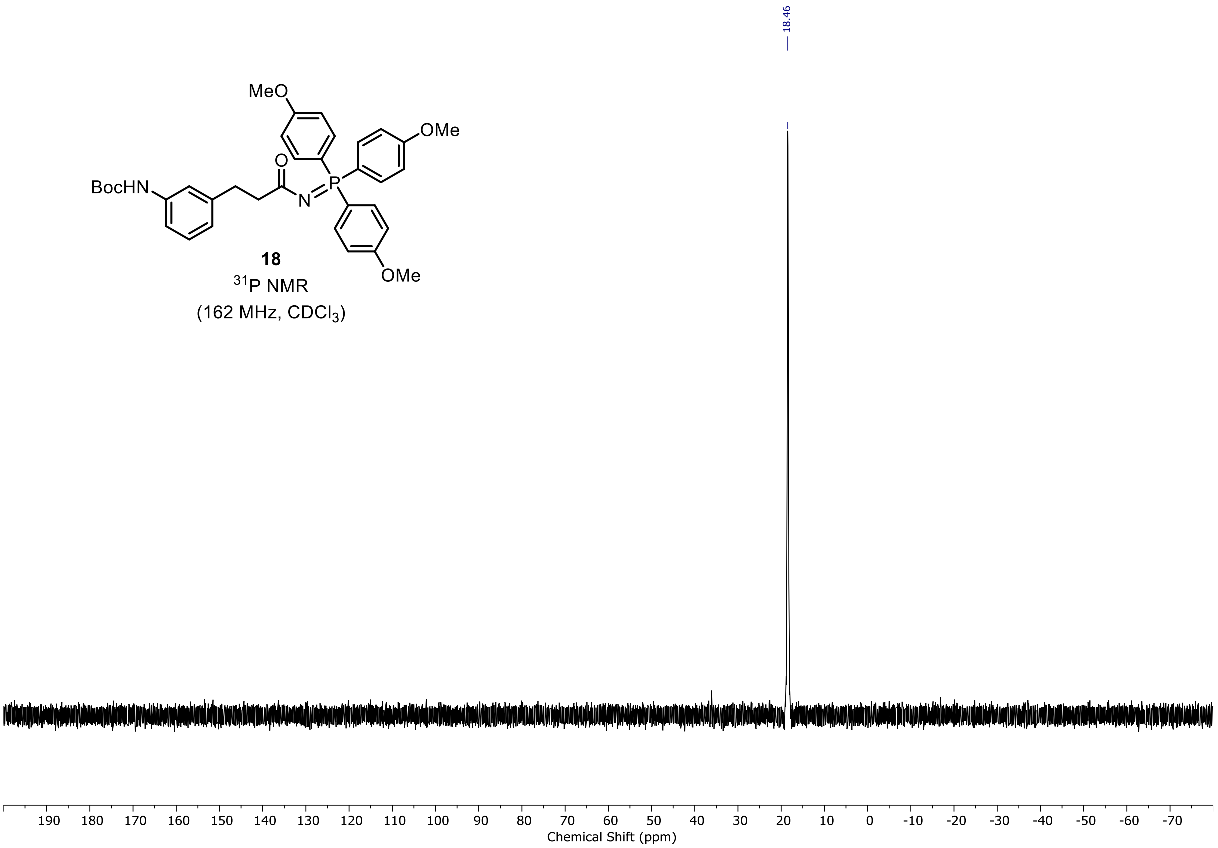

18
^{31}P NMR
(162 MHz, $CDCl_3$)

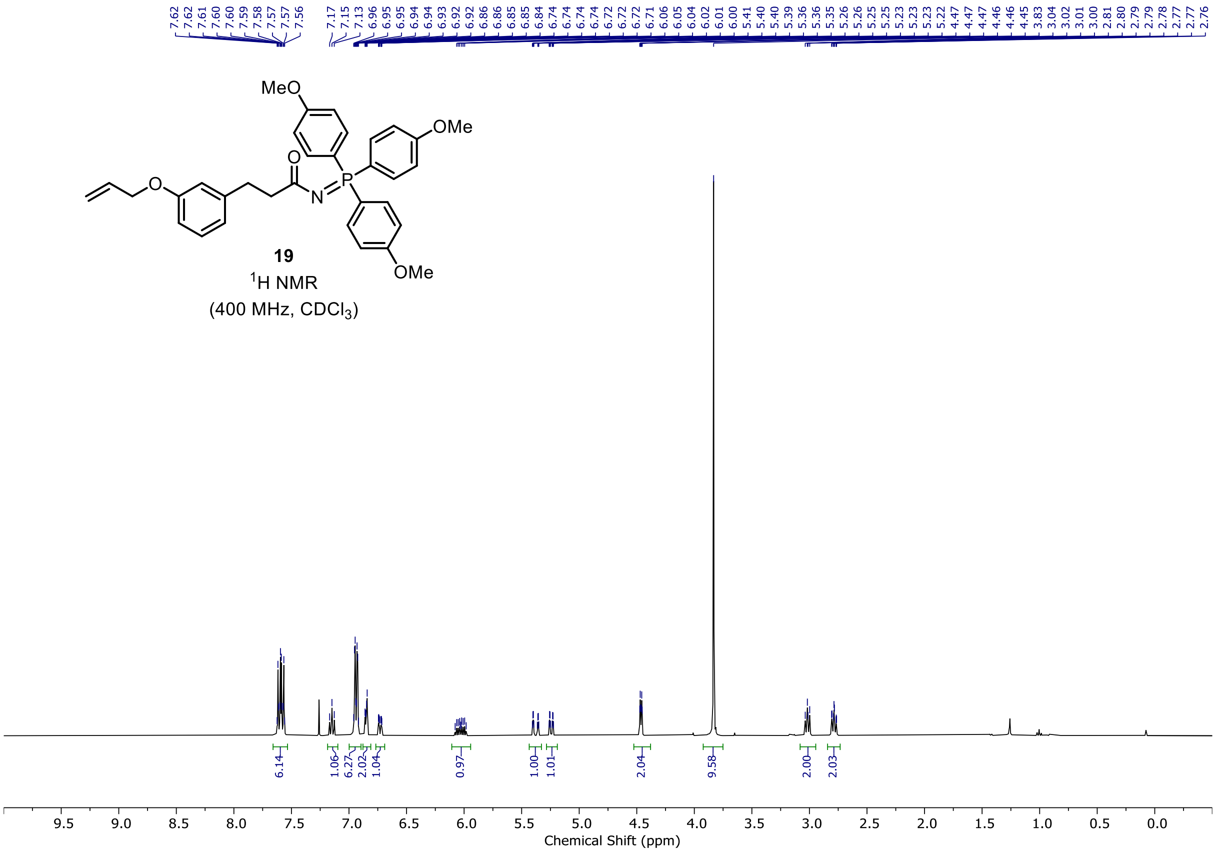
7.62 7.62 7.61 7.60 7.60 7.59 7.58 7.57 7.57 7.56 7.17 7.15 7.13 6.96 6.95 6.95 6.94 6.94 6.93 6.92 6.92 6.86 6.86 6.85 6.85 6.84 6.74 6.74 6.74 6.74 6.72 6.72 6.72 6.71 6.06 6.05 6.04 6.02 6.01 6.00 5.41 5.40 5.40 5.39 5.36 5.36 5.35 5.26 5.26 5.25 5.25 5.23 5.23 5.23 5.22 4.47 4.47 4.47 4.46 4.45 4.45 3.83 3.04 3.02 3.01 3.00 2.81 2.80 2.79 2.79 2.78 2.77 2.77 2.76
MeO
OMe
O
N
P
O
OMe
19
1H NMR
(400 MHz, CDCl3)
6.14 1.06 6.27 2.02 1.04 0.97 1.00 1.01 2.04 9.58 2.00 2.03
9.5 9.0 8.5 8.0 7.5 7.0 6.5 6.0 5.5 5.0 4.5 4.0 3.5 3.0 2.5 2.0 1.5 1.0 0.5 0.0
Chemical Shift (ppm)

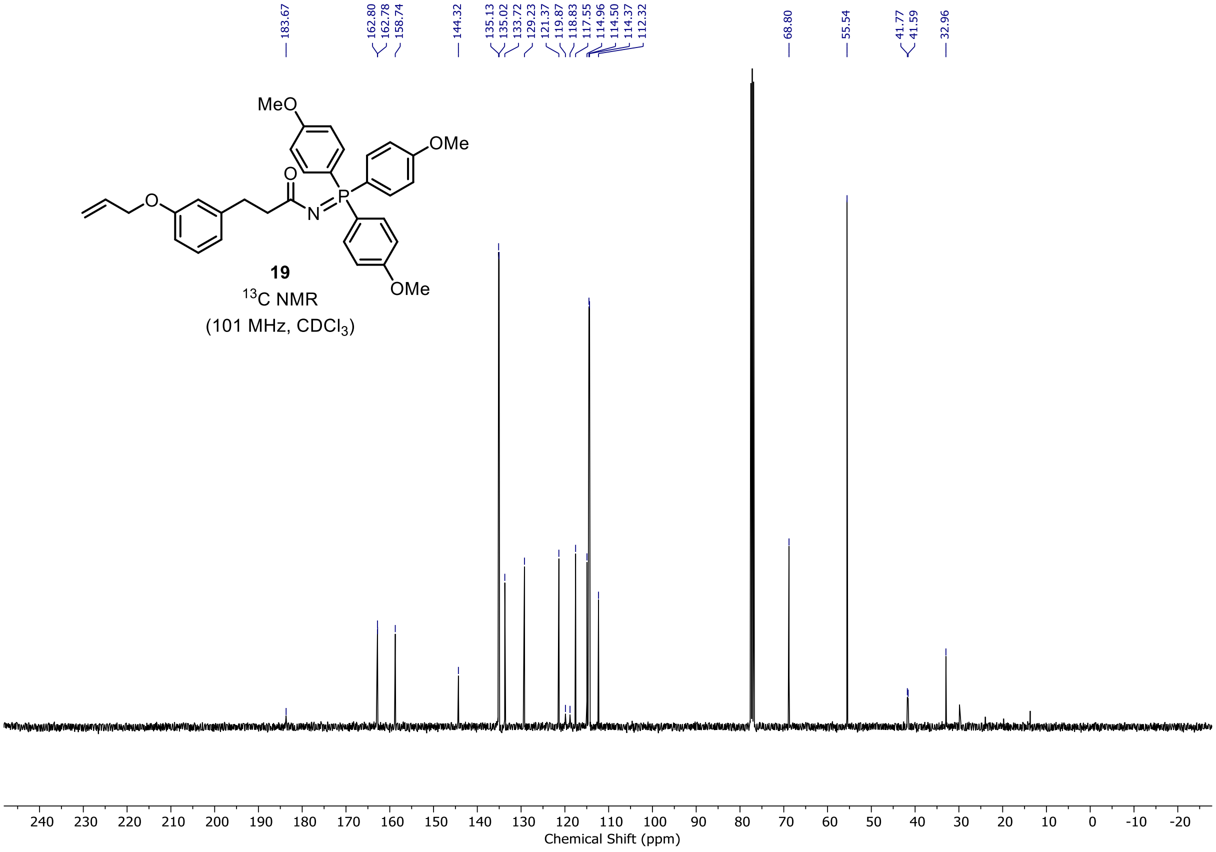
183.67 162.80 162.78 158.74 144.32 135.13 135.02 133.72 129.23 121.37 119.87 118.83 117.55 114.96 114.50 114.37 112.32 68.80 55.54 41.77 41.59 32.96
MeO
OMe
O
N
P
O
OMe
19
13C NMR
(101 MHz, CDCl3)
240 230 220 210 200 190 180 170 160 150 140 130 120 110 100 90 80 70 60 50 40 30 20 10 0 -10 -20
Chemical Shift (ppm)

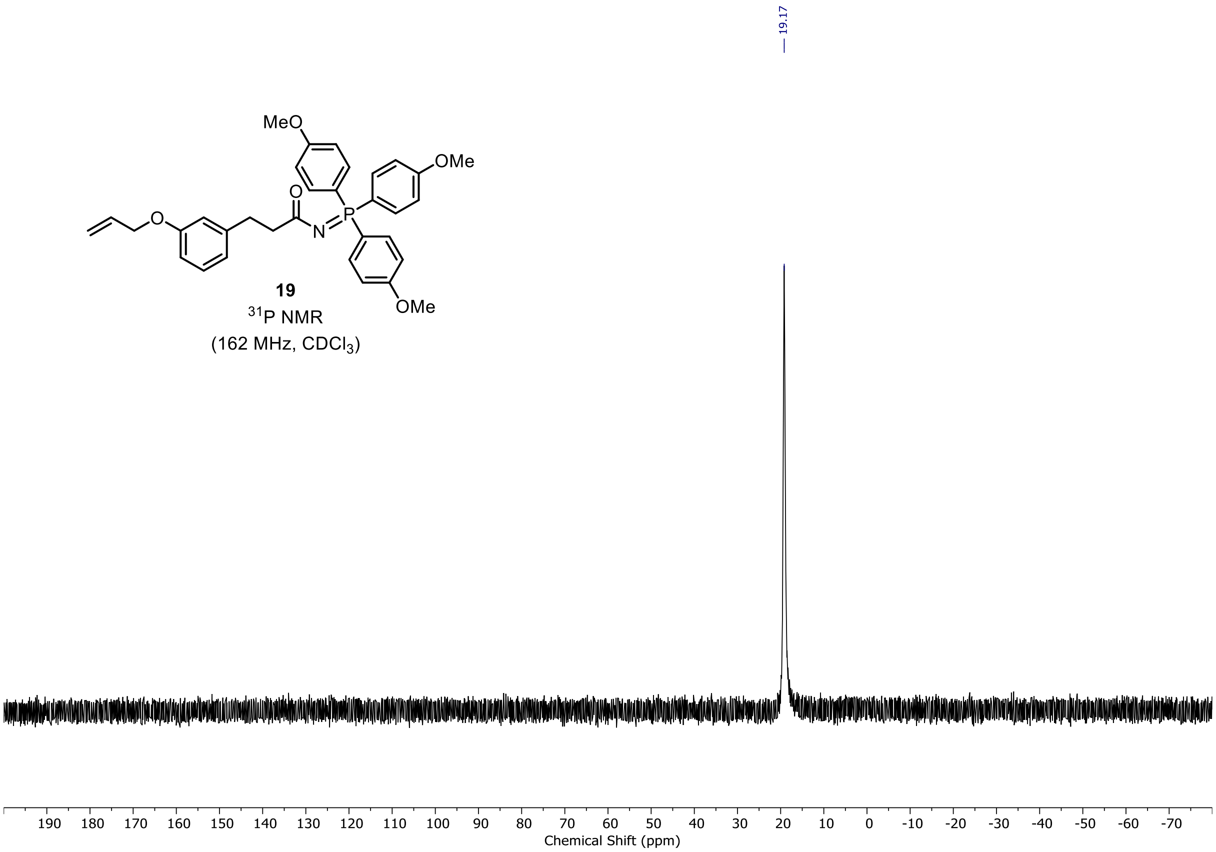
19.17
MeO
OMe
O
O
N
P
OMe
19
31P NMR
(162 MHz, CDCl3)
190 180 170 160 150 140 130 120 110 100 90 80 70 60 50 40 30 20 10 0 -10 -20 -30 -40 -50 -60 -70
Chemical Shift (ppm)

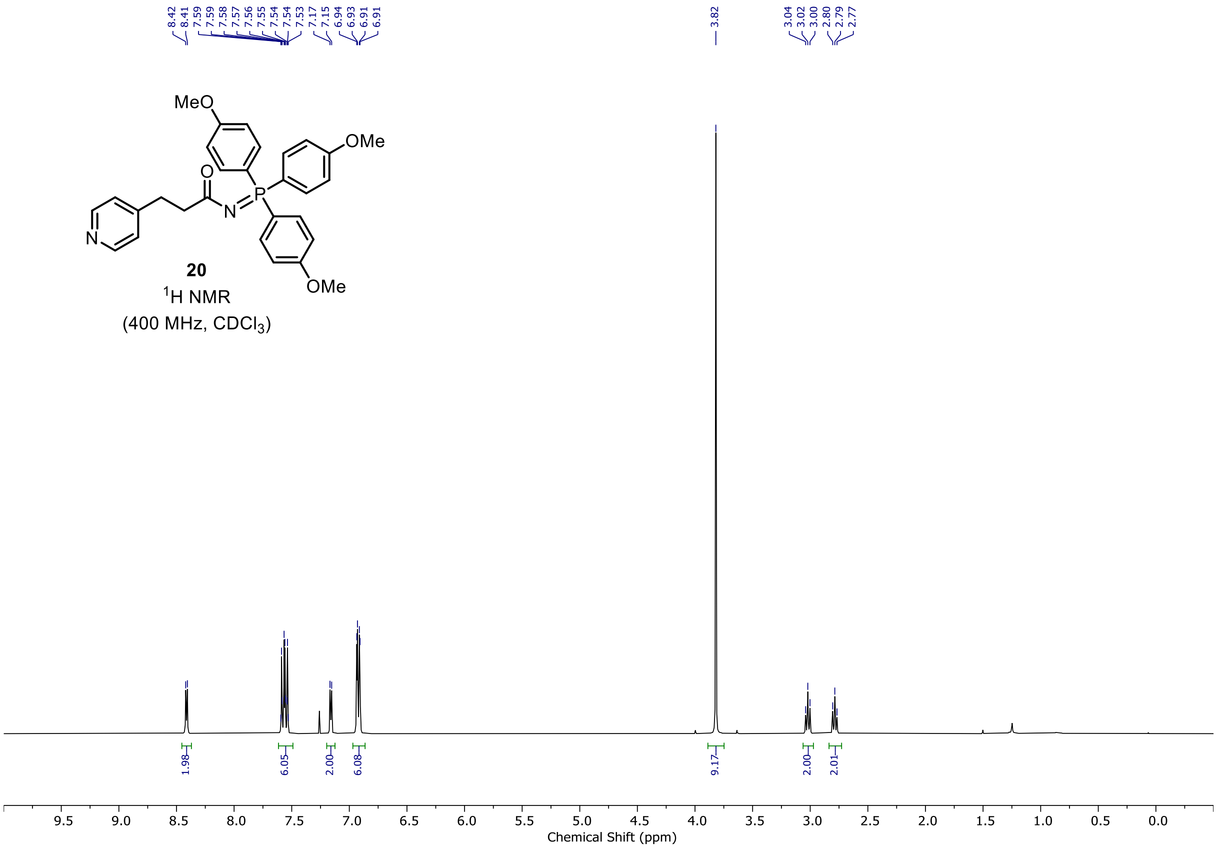

MeO
OMe
O
N
P
N
OMe
20
^{1}H NMR
(400 MHz, $CDCl_3$)
Chemical Shift (ppm)

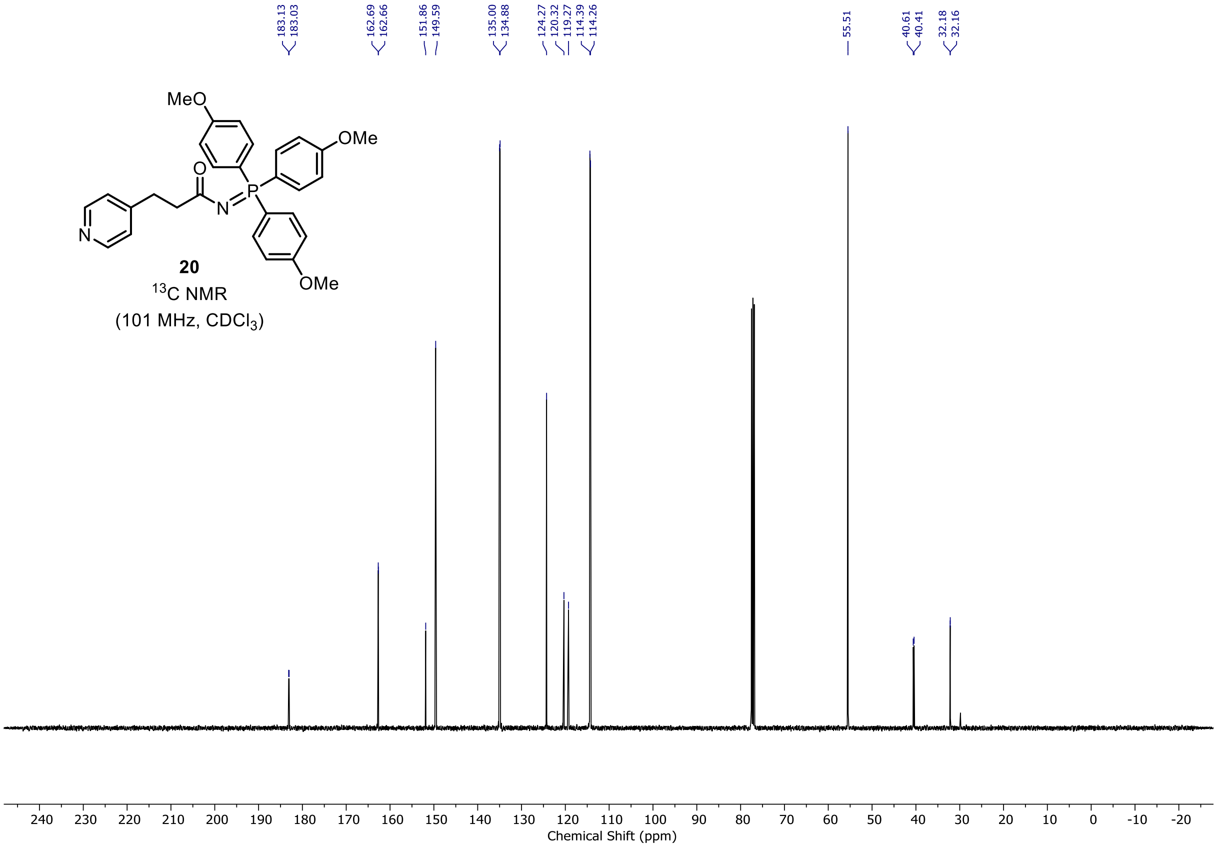

MeO
OMe
O
N
P
N
OMe
20
^{13}C NMR
(101 MHz, $CDCl_3$)
Chemical Shift (ppm)

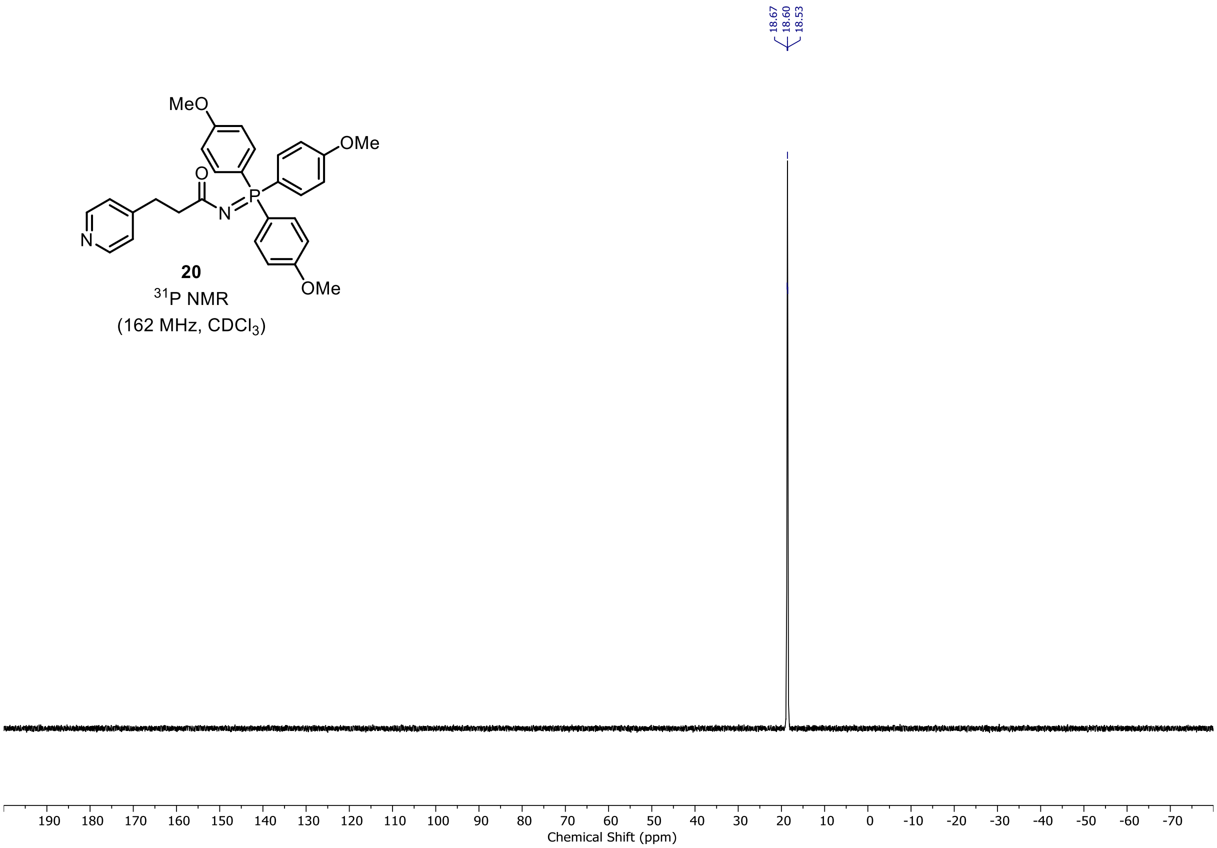

20

^{31}P NMR

(162 MHz, $CDCl_3$)

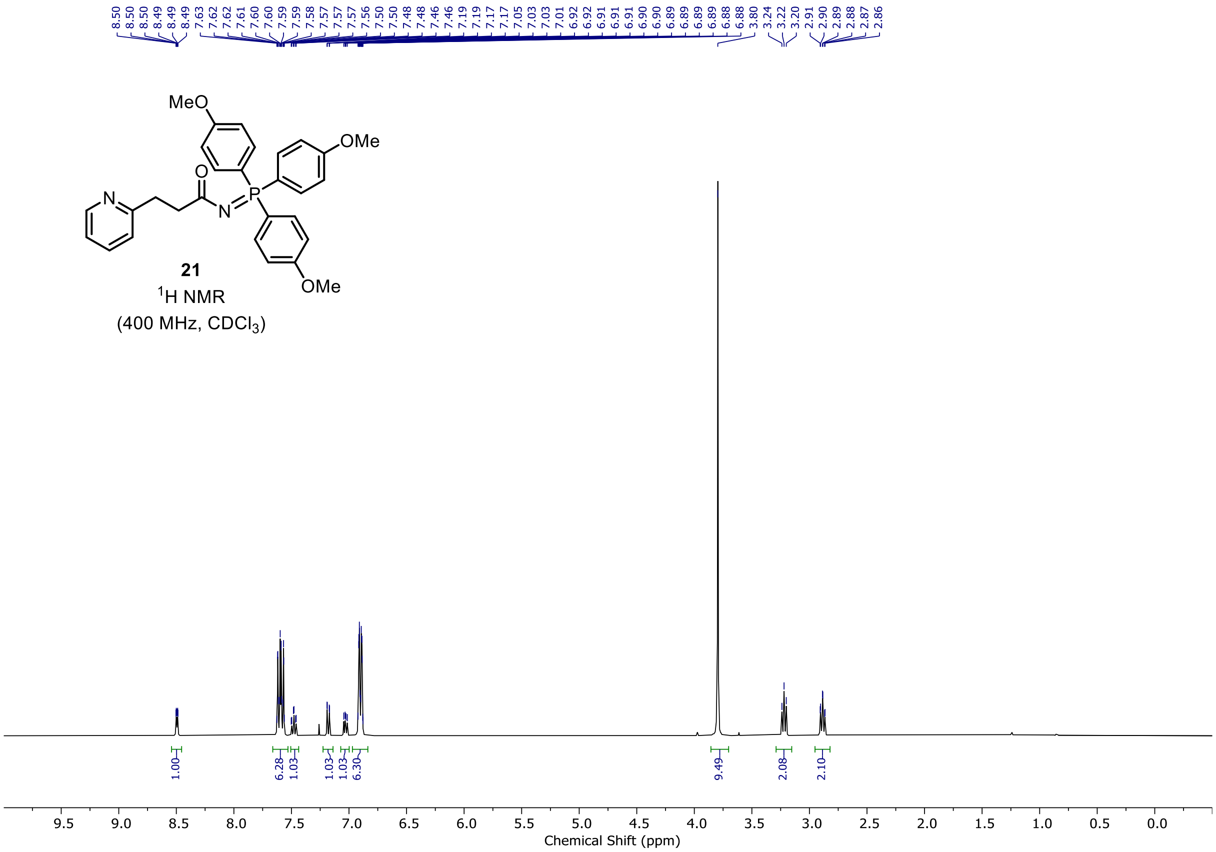

8.50 8.50 8.50 8.49 8.49 8.49 7.63 7.62 7.61 7.60 7.59 7.59 7.59 7.58 7.57 7.57 7.57 7.56 7.50 7.50 7.48 7.46 7.46 7.19 7.19 7.17 7.17 7.05 7.03 7.03 7.01 6.92 6.92 6.91 6.91 6.91 6.90 6.90 6.89 6.89 6.89 6.89 6.88 6.88 3.80 3.24 3.22 3.20 2.91 2.90 2.89 2.88 2.87 2.86
MeO
OMe
O
N
N
P
OMe
21
^{1}H NMR
(400 MHz, $CDCl_3$)
1.00 6.28 1.03 1.03 1.03 6.30 9.49 2.08 2.10
9.5 9.0 8.5 8.0 7.5 7.0 6.5 6.0 5.5 5.0 4.5 4.0 3.5 3.0 2.5 2.0 1.5 1.0 0.5 0.0
Chemical Shift (ppm)

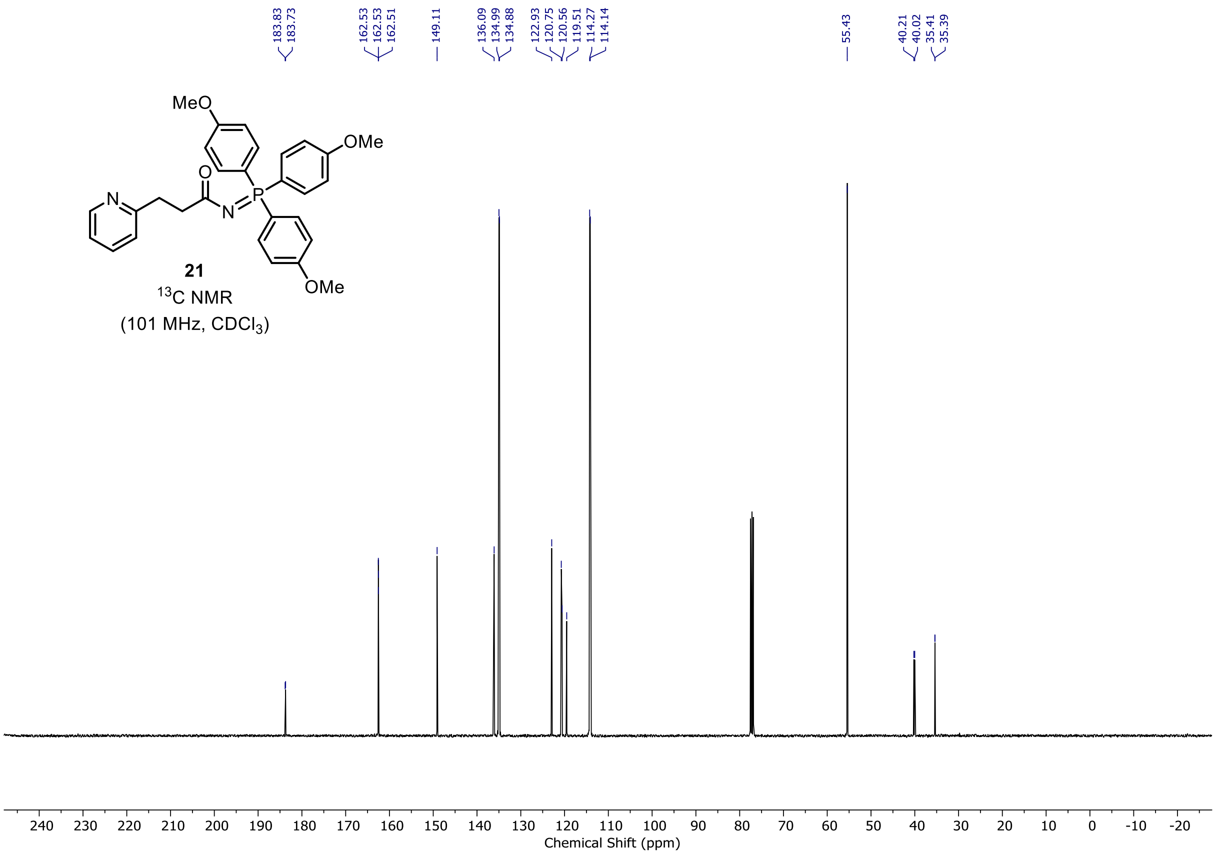

183.83 183.73 162.53 162.53 162.51 149.11 136.09 134.99 134.88 122.93 120.75 120.56 119.51 114.27 114.14 55.43 40.21 40.02 35.41 35.39
MeO
OMe
O
N
N
P
OMe
21
^{13}C NMR
(101 MHz, $CDCl_3$)
240 230 220 210 200 190 180 170 160 150 140 130 120 110 100 90 80 70 60 50 40 30 20 10 0 -10 -20
Chemical Shift (ppm)

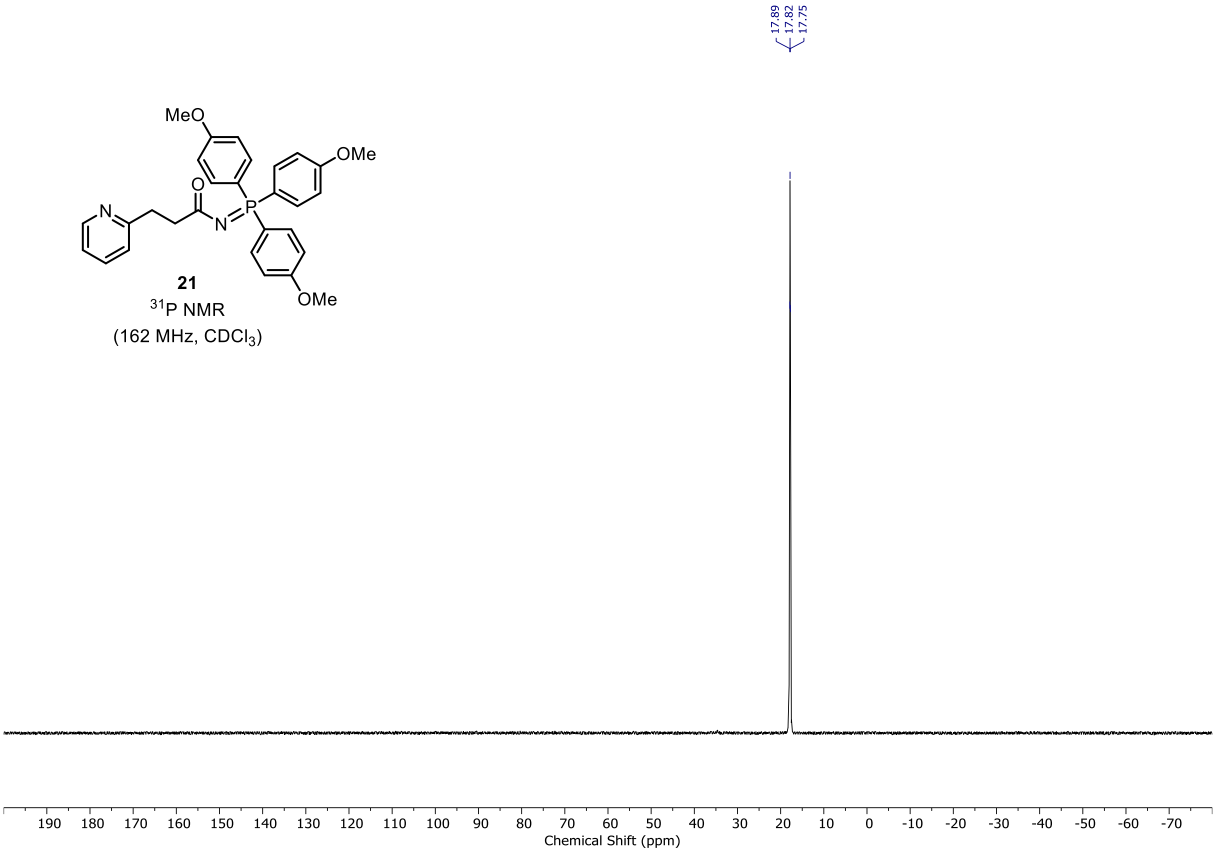

21

^{31}P NMR

(162 MHz, $CDCl_3$)

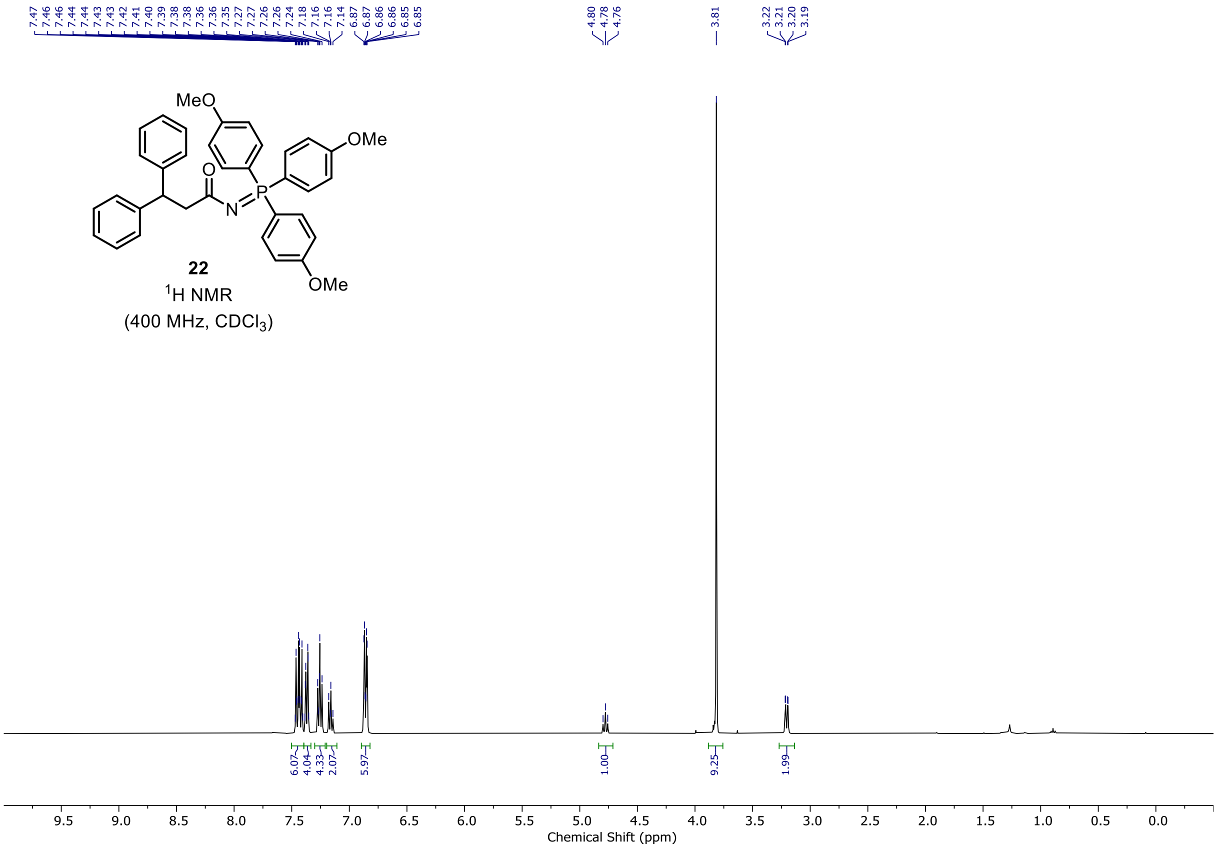
7.47 7.46 7.46 7.44 7.44 7.43 7.43 7.42 7.41 7.40 7.39 7.38 7.38 7.36 7.36 7.35 7.27 7.27 7.26 7.26 7.24 7.18 7.16 7.16 7.14 6.87 6.87 6.86 6.86 6.85 6.85
4.80 4.78 4.76
3.81
3.22 3.21 3.20 3.19
MeO
OMe
O
N
P
OMe
22
^{1}H NMR
(400 MHz, CDCl$_3$)
6.07 4.04 4.33 2.07 5.97 1.00 9.25 1.99
9.5 9.0 8.5 8.0 7.5 7.0 6.5 6.0 5.5 5.0 4.5 4.0 3.5 3.0 2.5 2.0 1.5 1.0 0.5 0.0
Chemical Shift (ppm)

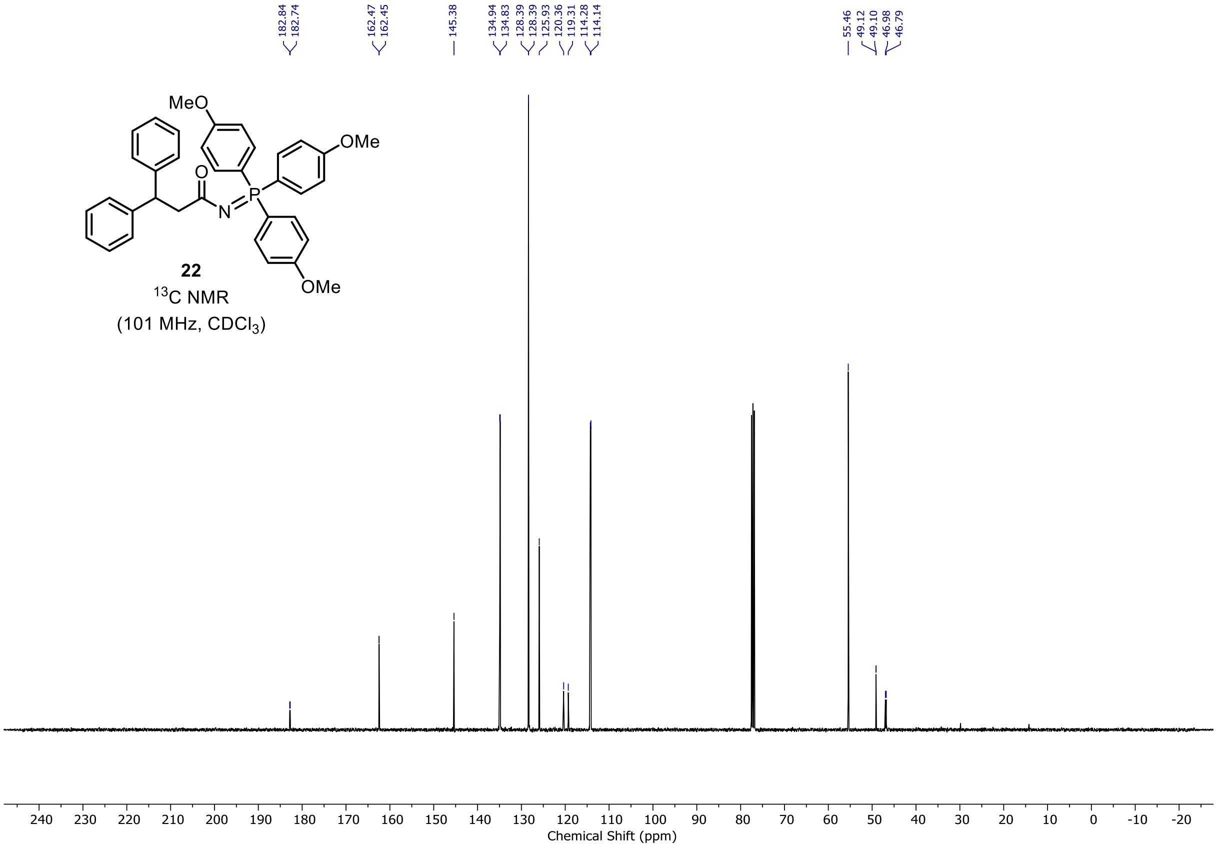
182.84 182.74
162.47 162.45
145.38
134.94 134.83 128.39 128.39 125.93 120.36 119.31 114.28 114.14
55.46 49.12 49.10 46.98 46.79
MeO
OMe
O
N
P
OMe
22
^{13}C NMR
(101 MHz, CDCl$_3$)
240 230 220 210 200 190 180 170 160 150 140 130 120 110 100 90 80 70 60 50 40 30 20 10 0 -10 -20
Chemical Shift (ppm)

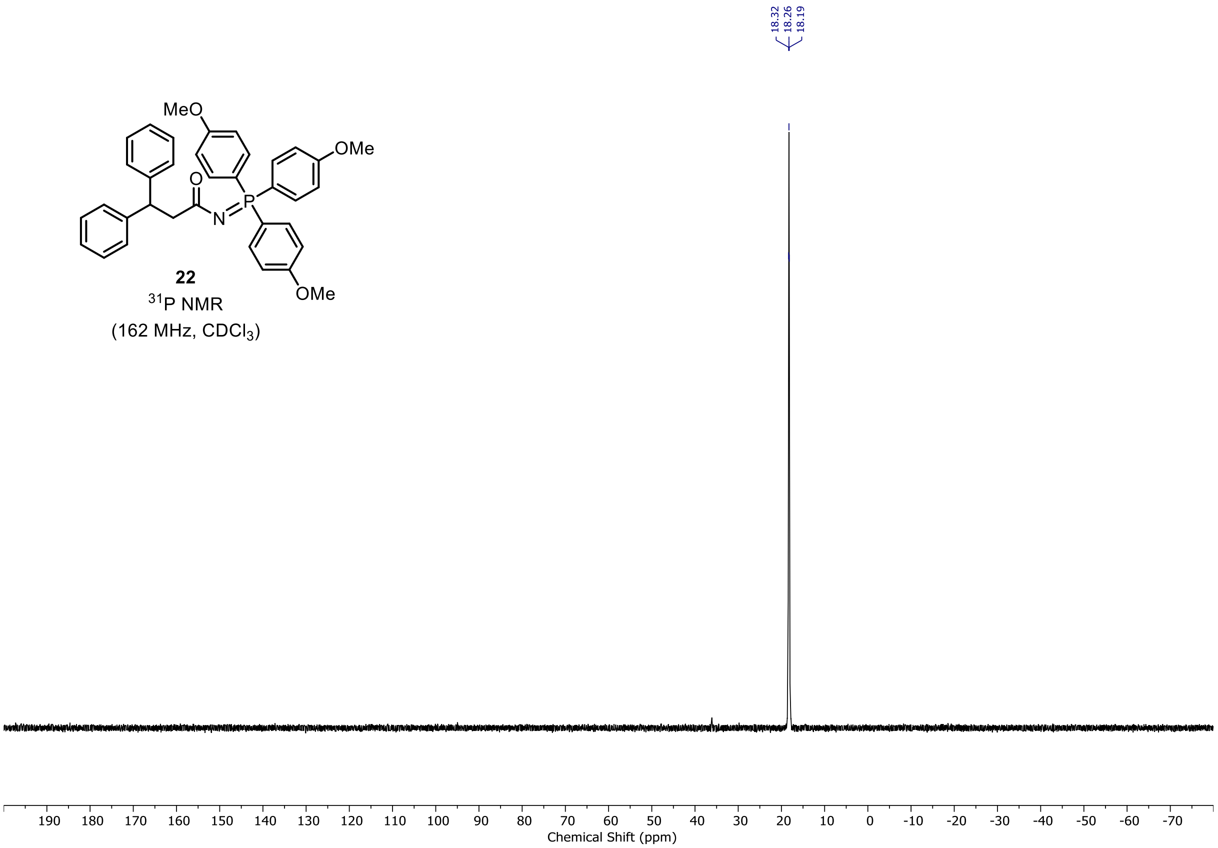
18.32
18.26
18.19
MeO
OMe
O
N
P
OMe
22
^{31}P NMR
(162 MHz, CDCl$_3$)
190 180 170 160 150 140 130 120 110 100 90 80 70 60 50 40 30 20 10 0 -10 -20 -30 -40 -50 -60 -70
Chemical Shift (ppm)

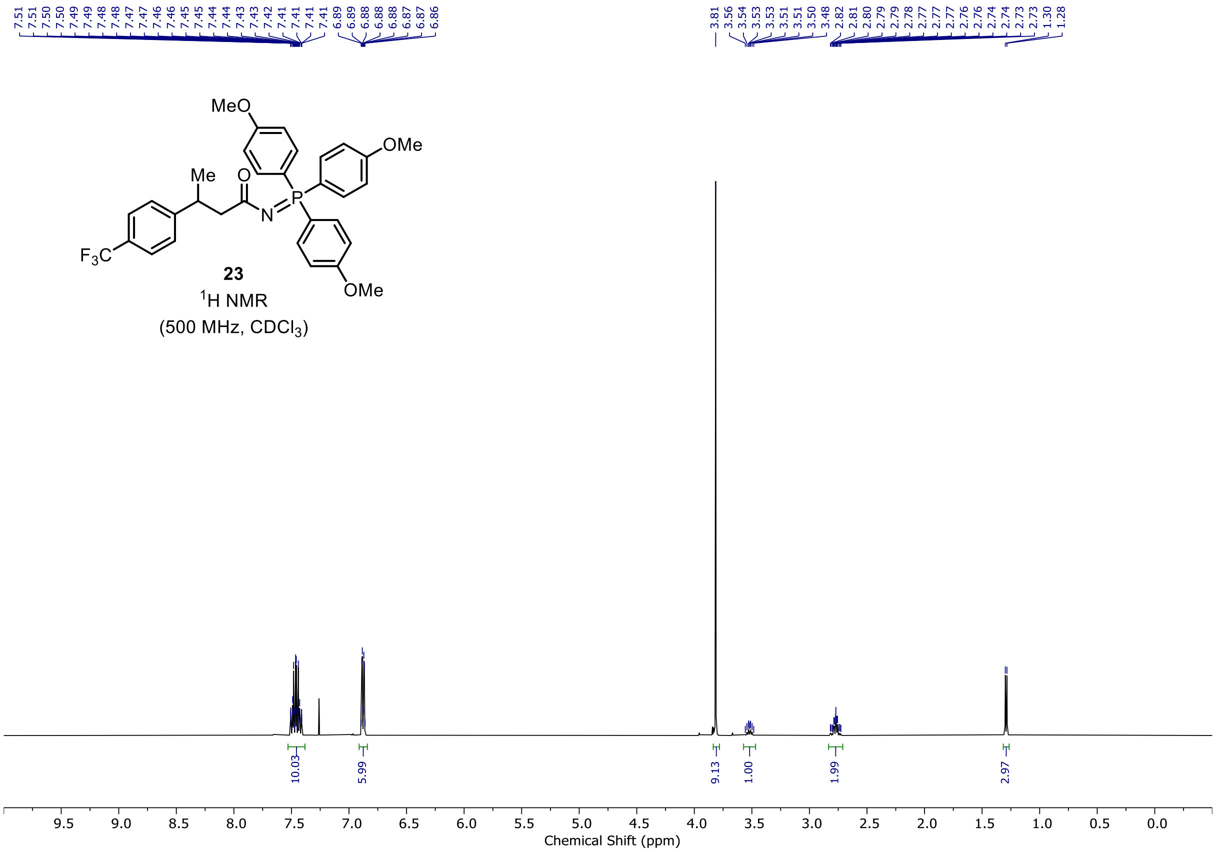
7.51 7.51 7.50 7.49 7.49 7.48 7.48 7.47 7.47 7.46 7.46 7.45 7.45 7.44 7.44 7.43 7.43 7.42 7.41 7.41 7.41 7.41 6.89 6.89 6.88 6.88 6.88 6.87 6.87 6.86
3.81 3.56 3.54 3.53 3.53 3.51 3.51 3.50 3.48 2.82 2.81 2.80 2.79 2.79 2.78 2.77 2.77 2.77 2.76 2.76 2.74 2.74 2.73 2.73 1.30 1.28
MeO
OMe
Me
O
N
P
F3C
OMe
23
1H NMR
(500 MHz, CDCl3)
10.03
5.99
9.13
1.00
1.99
2.97
Chemical Shift (ppm)

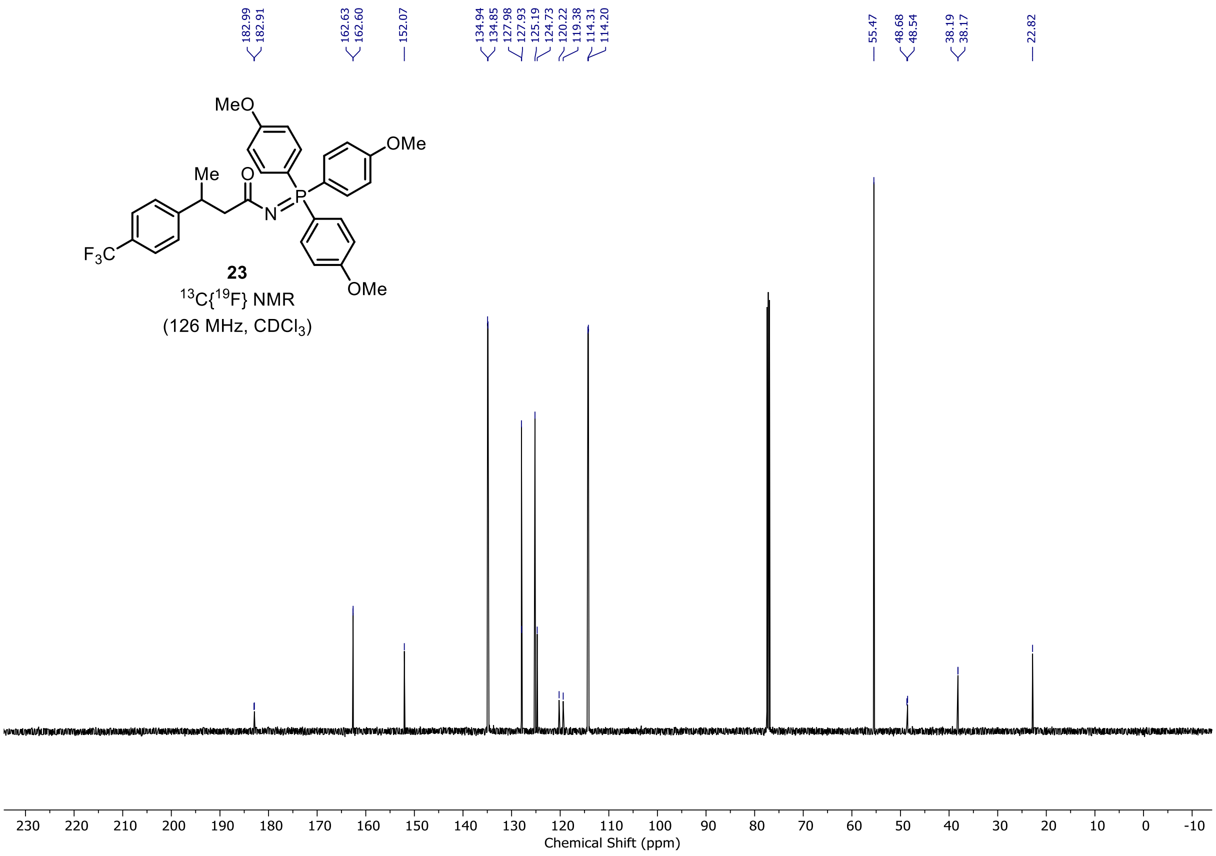
182.99 182.91
162.63 162.60
152.07
134.94 134.85 127.98 127.93 125.19 124.73 120.22 119.38 114.31 114.20
55.47
48.68 48.54
38.19 38.17
22.82
MeO
OMe
Me
O
N
P
F3C
OMe
23
13C{19F} NMR
(126 MHz, CDCl3)
Chemical Shift (ppm)

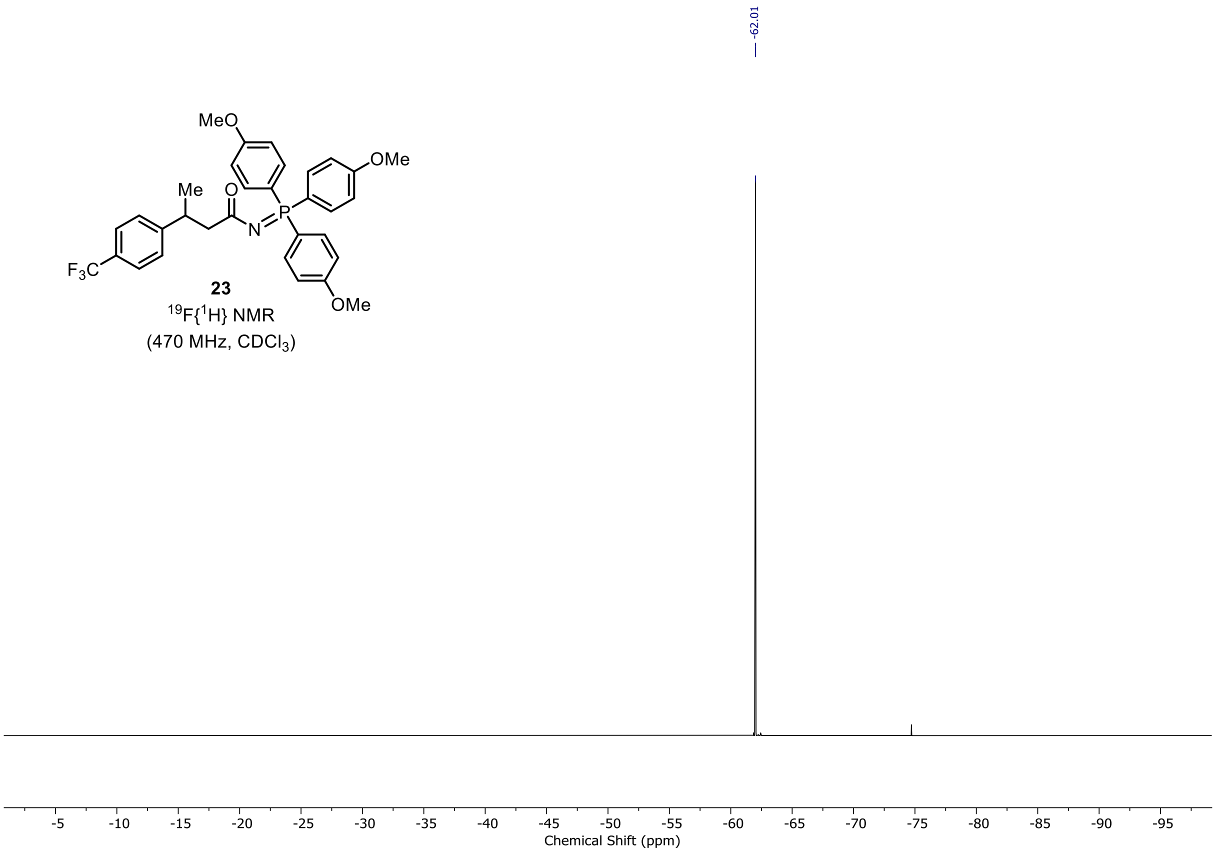

23
$^{19}F\{^{1}H\}$ NMR
(470 MHz, $CDCl_3$)

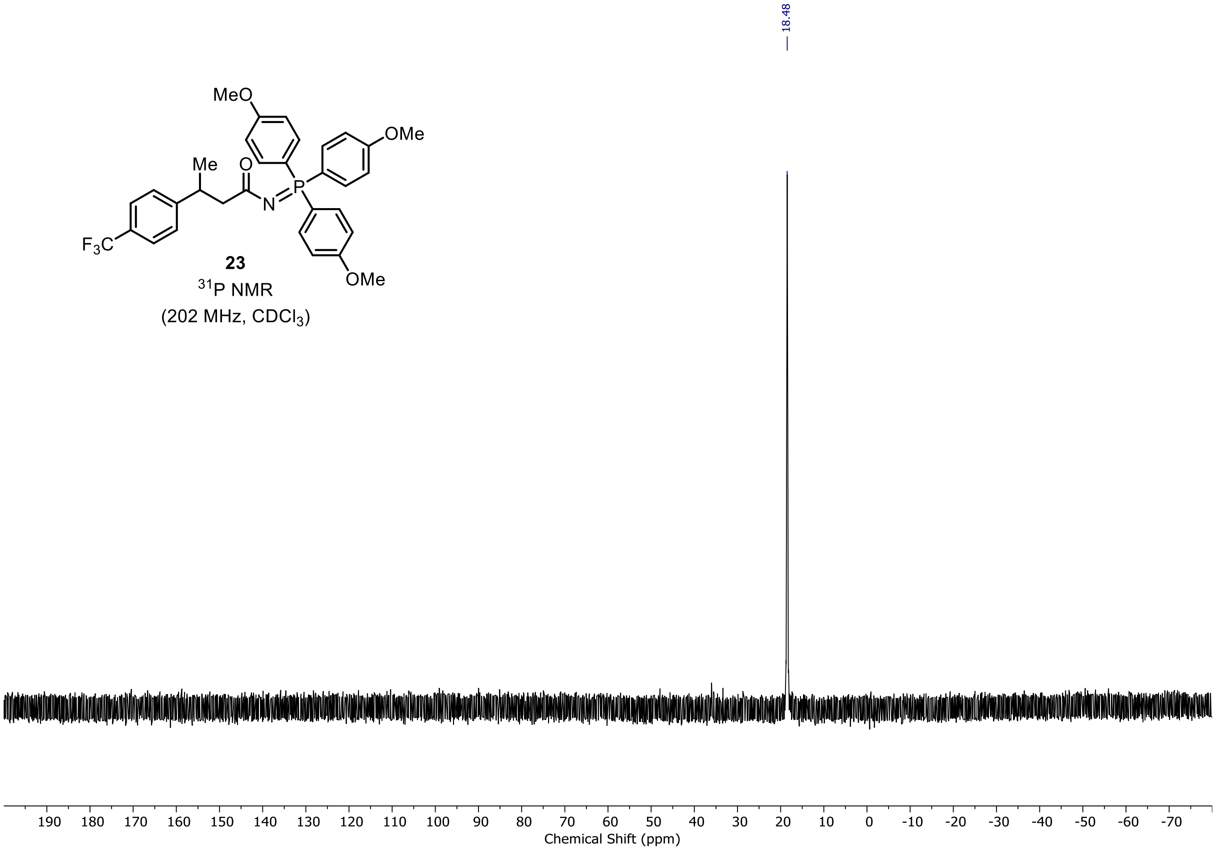

23
^{31}P NMR
(202 MHz, $CDCl_3$)

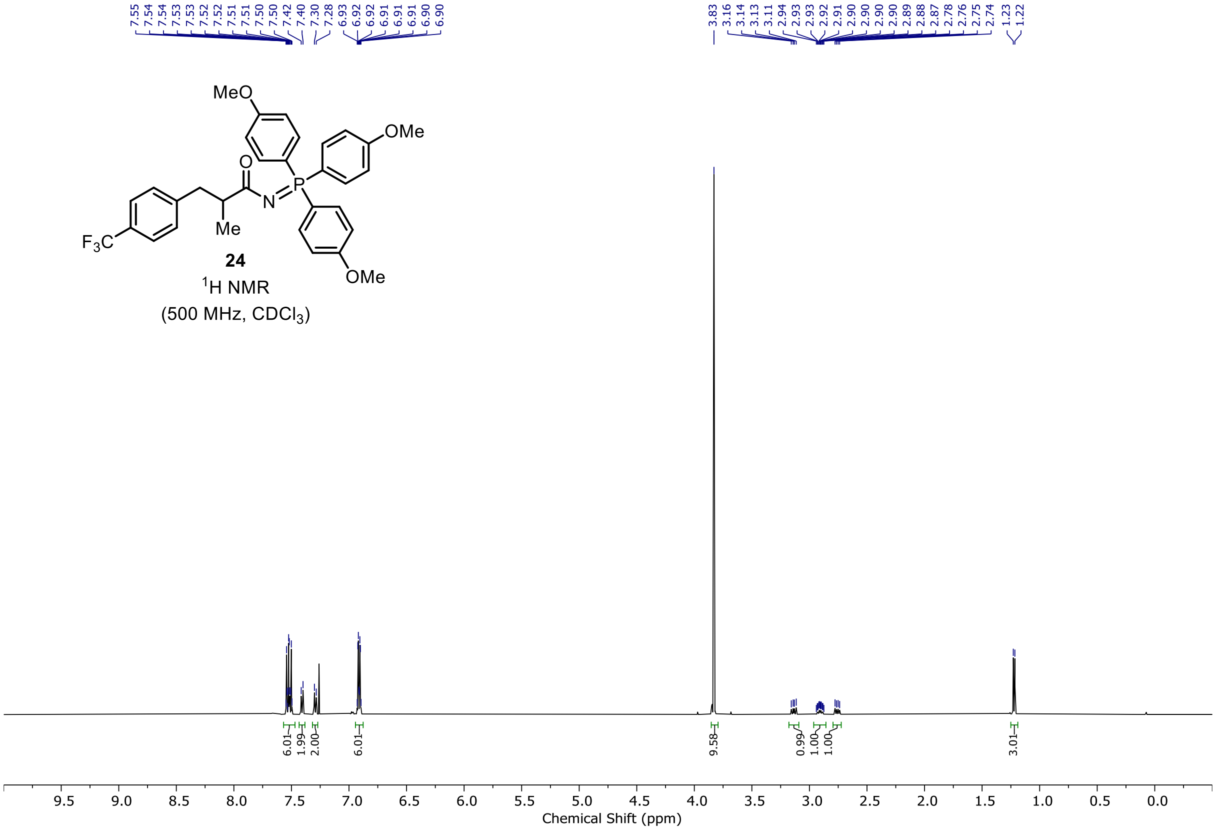
7.55 7.54 7.54 7.53 7.53 7.52 7.52 7.51 7.51 7.50 7.50 7.42 7.40 7.30 7.28 6.93 6.92 6.92 6.91 6.91 6.91 6.90 6.90
3.83 3.16 3.14 3.13 3.11 2.94 2.93 2.93 2.92 2.91 2.91 2.90 2.90 2.90 2.90 2.89 2.88 2.87 2.78 2.76 2.75 2.74 1.23 1.22
MeO
OMe
O
P
N
F3C
Me
OMe
24
1H NMR
(500 MHz, CDCl3)
6.01 1.99 2.00 6.01 9.58 0.99 1.00 1.00 3.01
9.5 9.0 8.5 8.0 7.5 7.0 6.5 6.0 5.5 5.0 4.5 4.0 3.5 3.0 2.5 2.0 1.5 1.0 0.5 0.0
Chemical Shift (ppm)

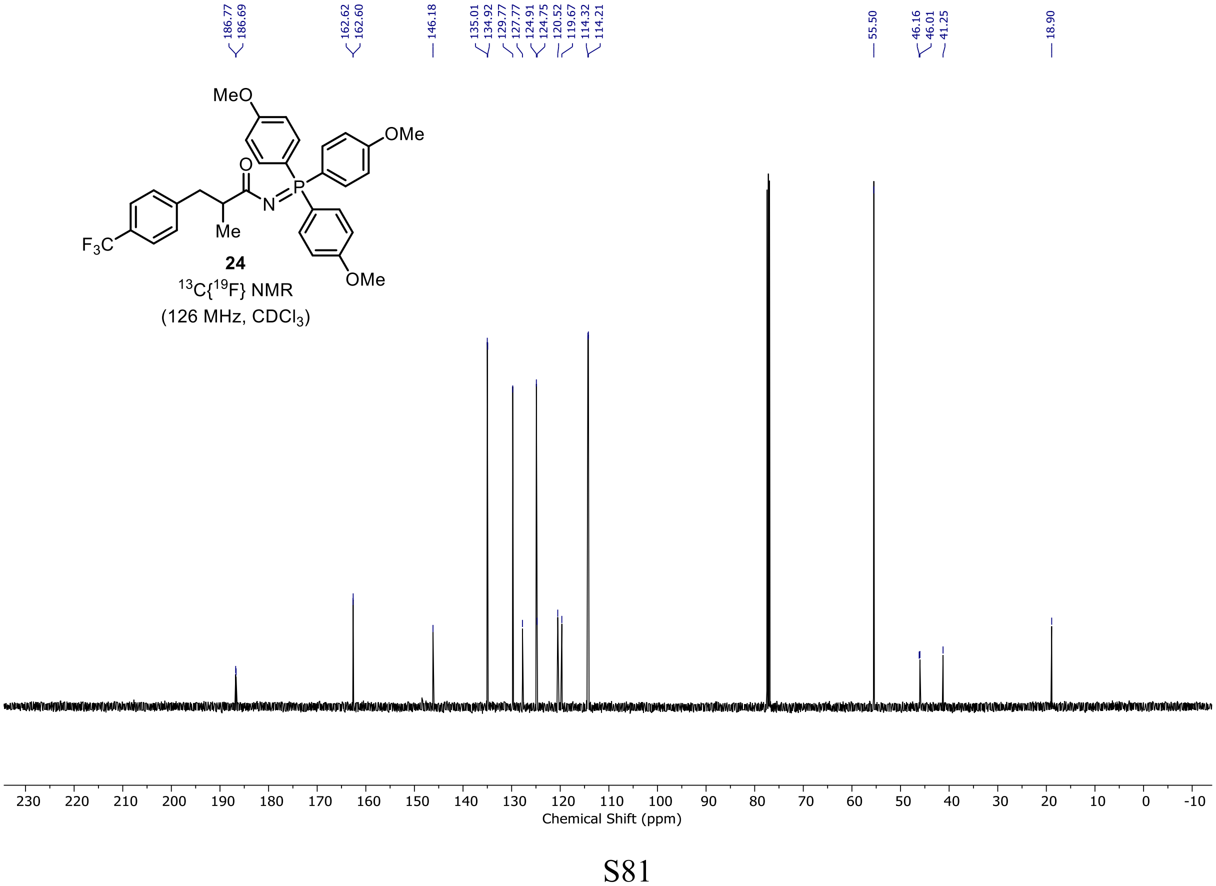
186.77 186.69 162.62 162.60 146.18 135.01 134.92 129.77 127.77 124.91 124.75 120.52 119.67 114.32 114.21 55.50 46.16 46.01 41.25 18.90
MeO
OMe
O
P
N
F3C
Me
OMe
24
13C{19F} NMR
(126 MHz, CDCl3)
230 220 210 200 190 180 170 160 150 140 130 120 110 100 90 80 70 60 50 40 30 20 10 0 -10
Chemical Shift (ppm)

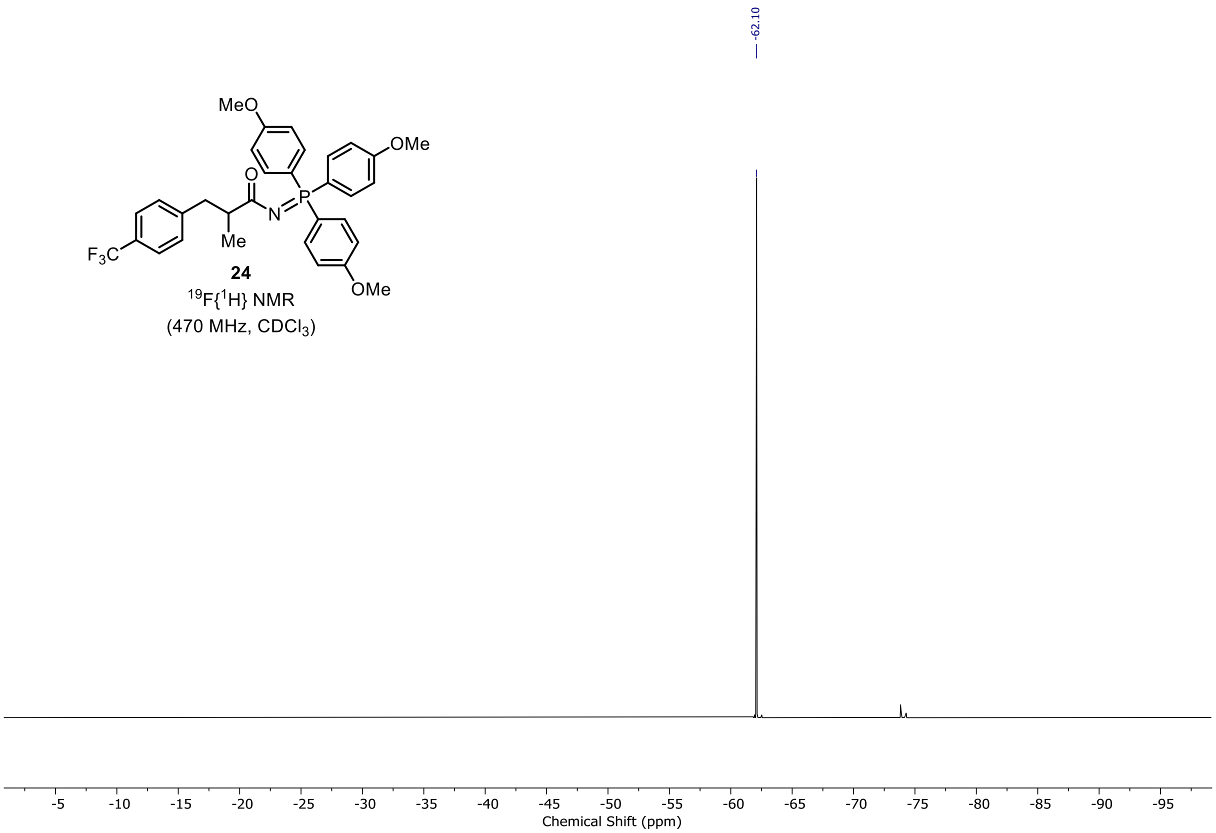
-62.10
MeO
OMe
O
N
P
Me
F_3C
OMe
24
$^{19}F\{^{1}H\}$ NMR
(470 MHz, $CDCl_3$)
-5 -10 -15 -20 -25 -30 -35 -40 -45 -50 -55 -60 -65 -70 -75 -80 -85 -90 -95
Chemical Shift (ppm)

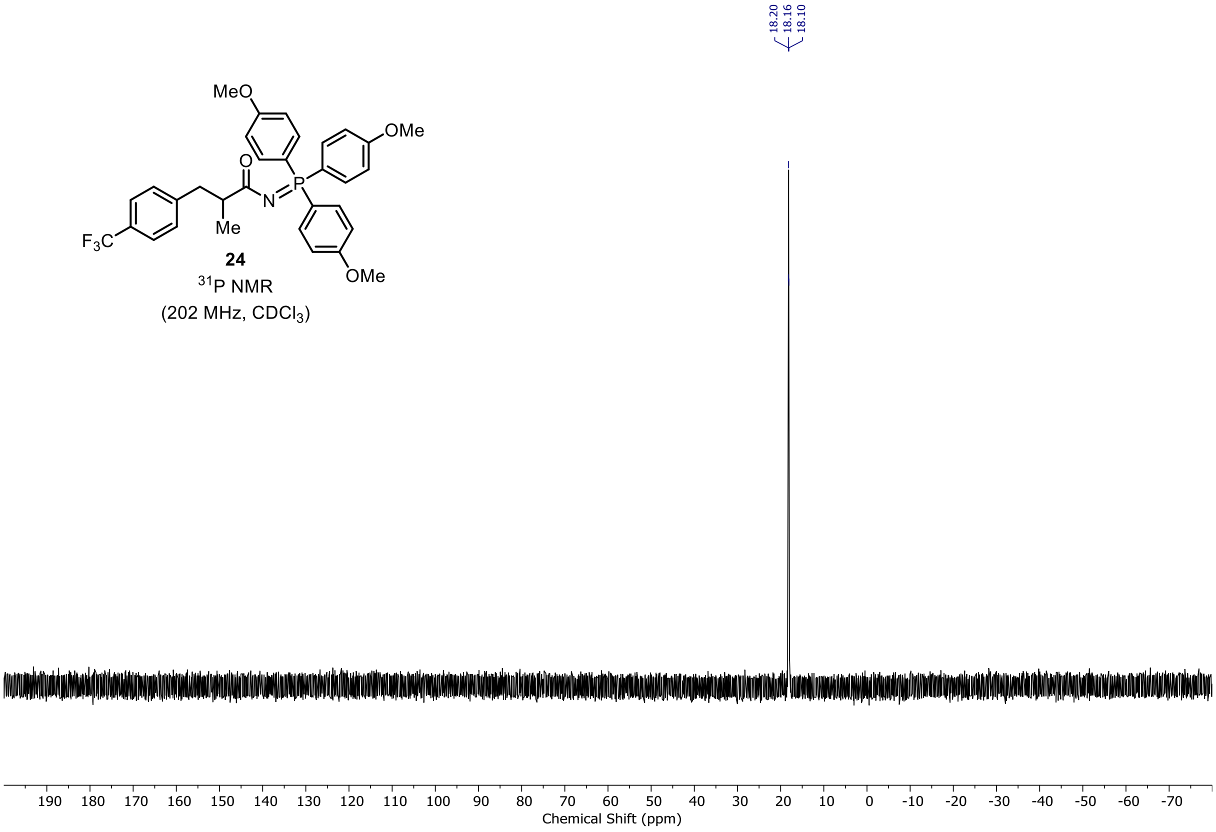
18.20
18.16
18.10
MeO
OMe
O
N
P
Me
F_3C
OMe
24
^{31}P NMR
(202 MHz, $CDCl_3$)
190 180 170 160 150 140 130 120 110 100 90 80 70 60 50 40 30 20 10 0 -10 -20 -30 -40 -50 -60 -70
Chemical Shift (ppm)

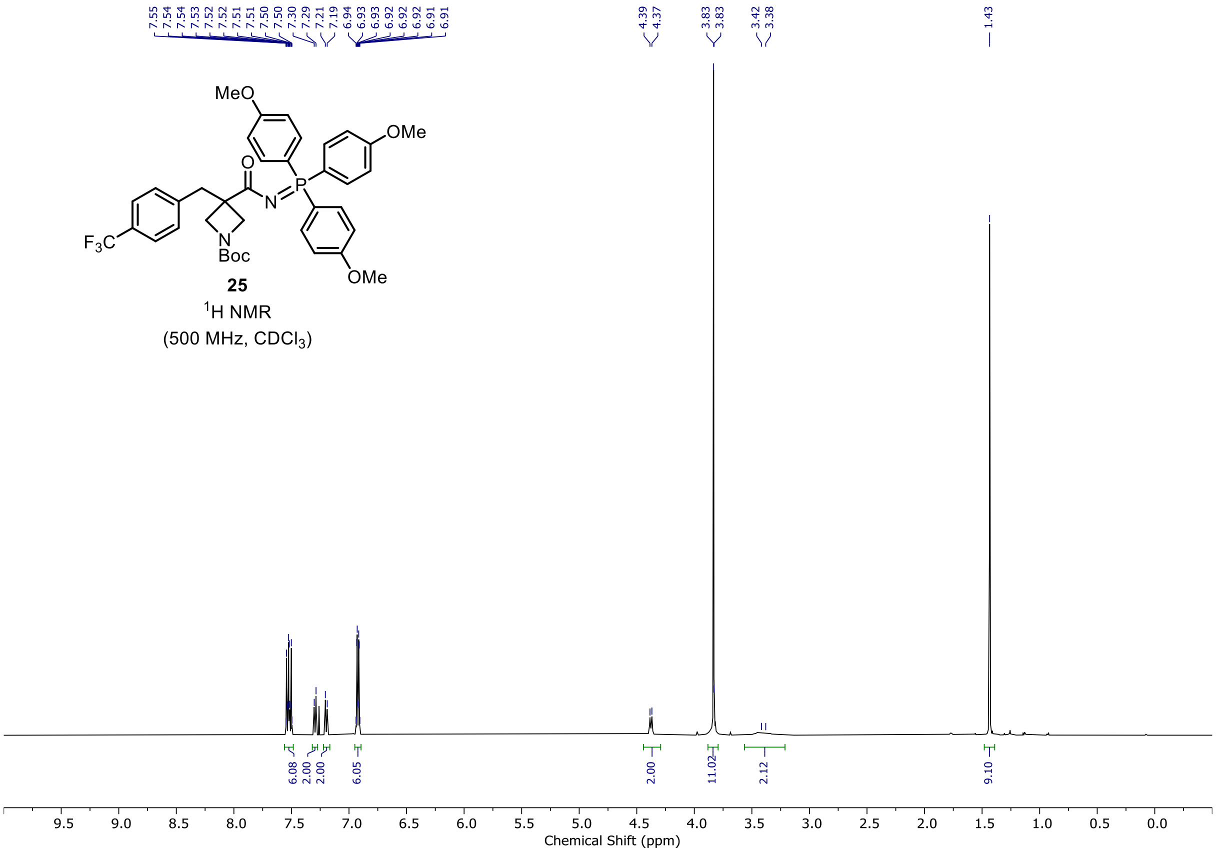
MeO
OMe
OMe
O
N
P
F3C
N
Boc
25
1H NMR
(500 MHz, CDCl3)
7.55 7.54 7.54 7.53 7.52 7.52 7.51 7.51 7.50 7.50 7.30 7.29 7.21 7.19 6.94 6.93 6.93 6.92 6.92 6.92 6.91 6.91
4.39 4.37
3.83 3.83
3.42 3.38
1.43
6.08 2.00 2.00 6.05 2.00 11.02 2.12 9.10
9.5 9.0 8.5 8.0 7.5 7.0 6.5 6.0 5.5 5.0 4.5 4.0 3.5 3.0 2.5 2.0 1.5 1.0 0.5 0.0
Chemical Shift (ppm)

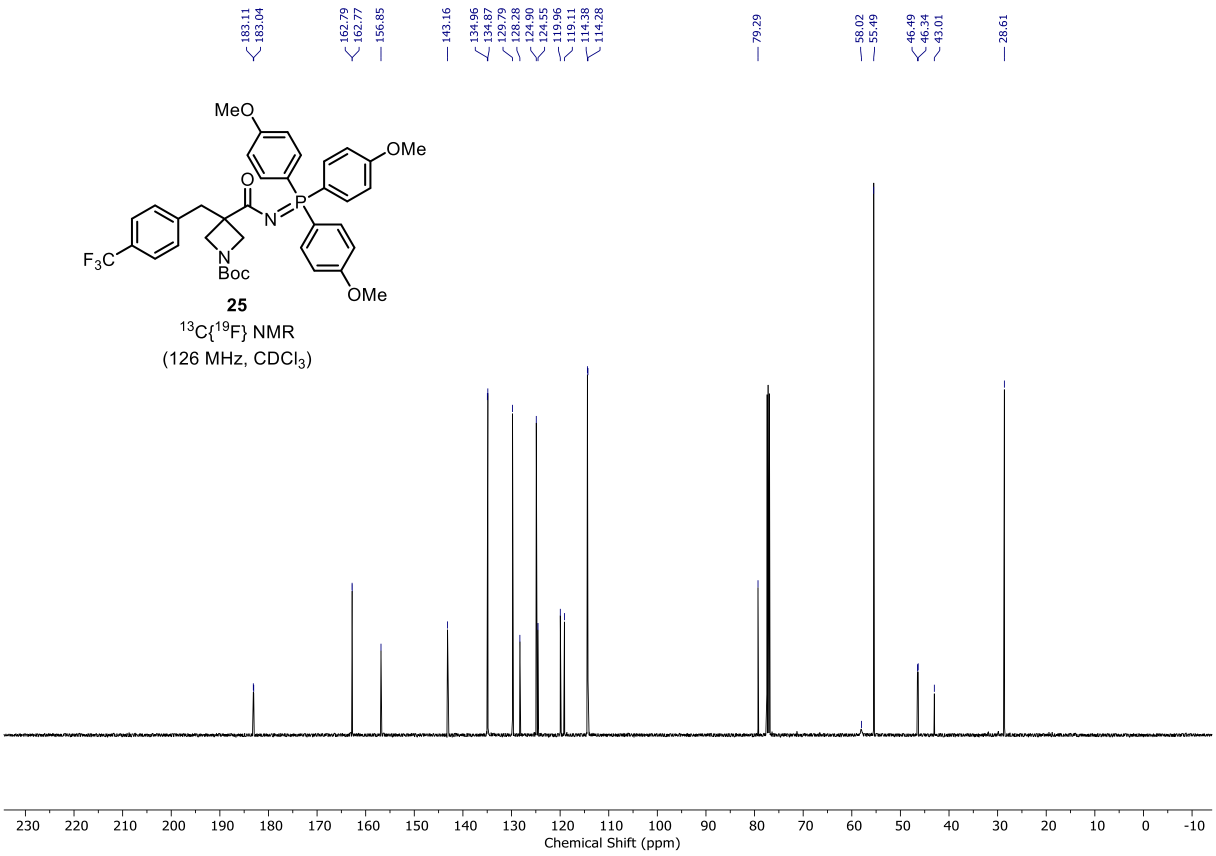
MeO
OMe
OMe
O
N
P
F3C
N
Boc
25
13C{19F} NMR
(126 MHz, CDCl3)
183.11 183.04
162.79 162.77 156.85
143.16
134.96 134.87 129.79 128.28 124.90 124.55 119.96 119.11 114.38 114.28
79.29
58.02 55.49
46.49 46.34 43.01
28.61
230 220 210 200 190 180 170 160 150 140 130 120 110 100 90 80 70 60 50 40 30 20 10 0 -10
Chemical Shift (ppm)

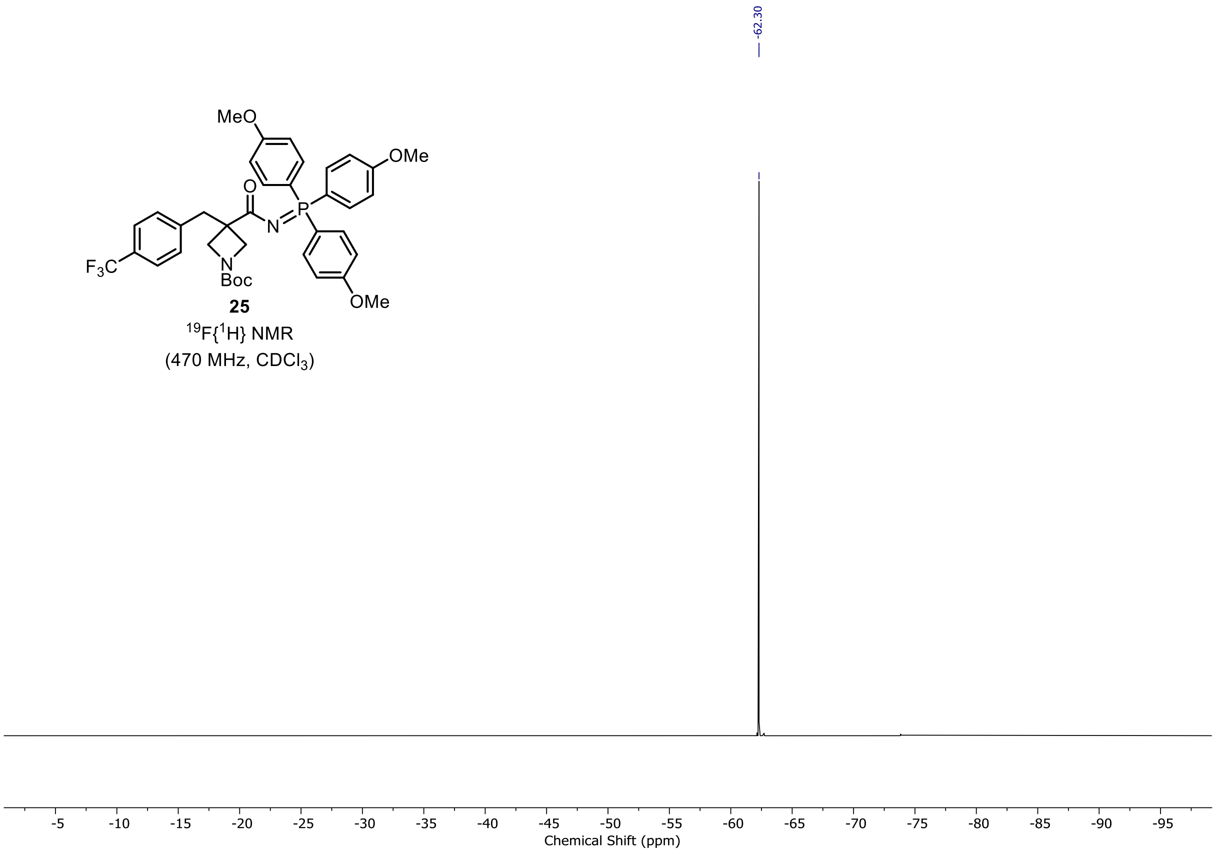

-62.30
MeO
OMe
O
N
P
F3C
N
Boc
OMe
25
19F{1H} NMR
(470 MHz, CDCl3)
-5 -10 -15 -20 -25 -30 -35 -40 -45 -50 -55 -60 -65 -70 -75 -80 -85 -90 -95
Chemical Shift (ppm)

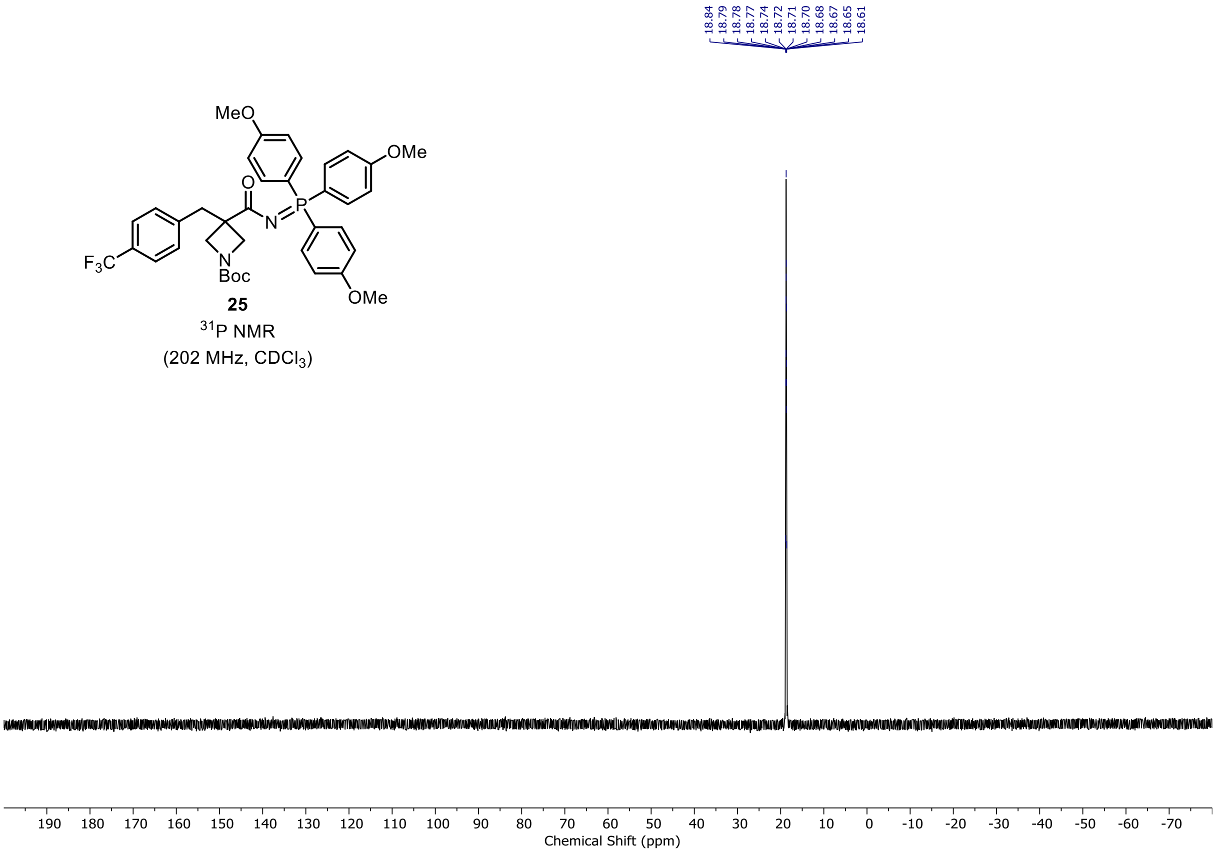

18.84
18.79
18.78
18.77
18.74
18.72
18.71
18.70
18.68
18.67
18.65
18.61
MeO
OMe
O
N
P
F3C
N
Boc
OMe
25
31P NMR
(202 MHz, CDCl3)
190 180 170 160 150 140 130 120 110 100 90 80 70 60 50 40 30 20 10 0 -10 -20 -30 -40 -50 -60 -70
Chemical Shift (ppm)

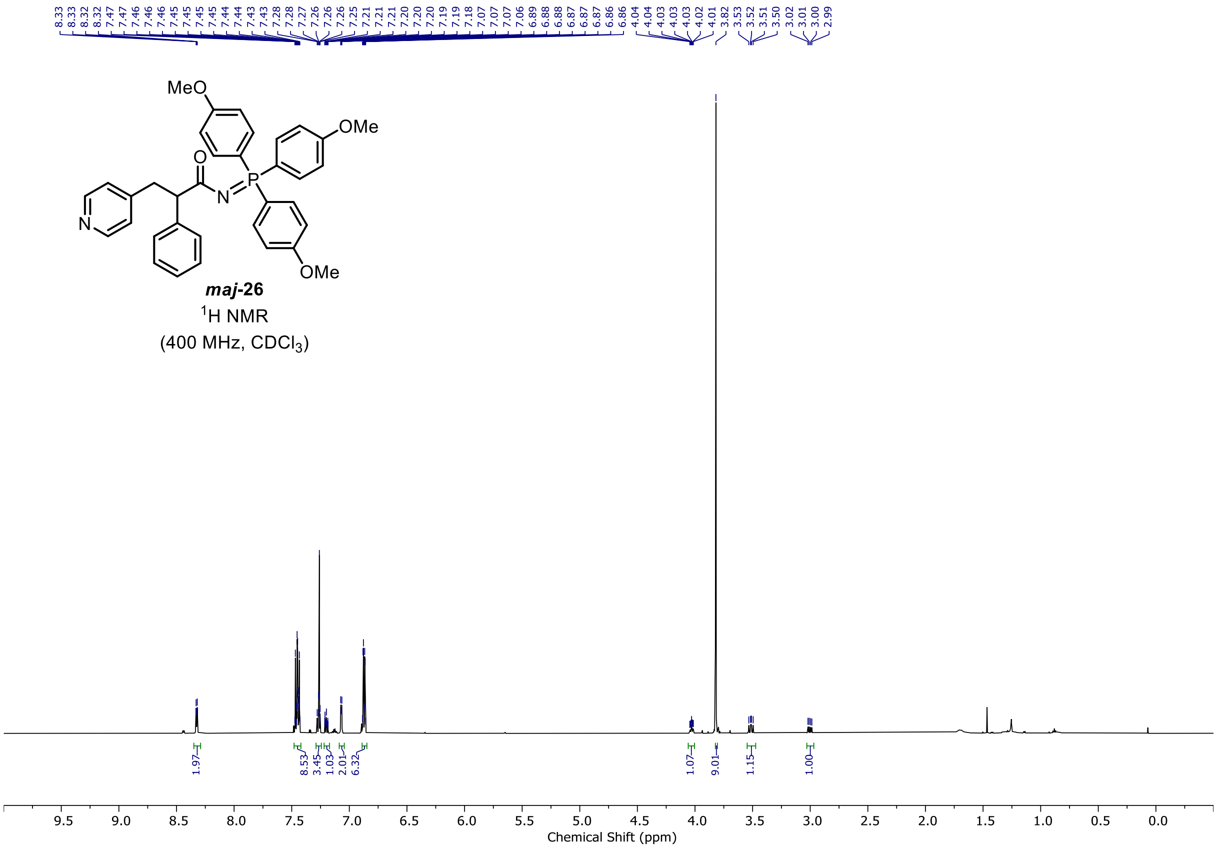
8.33 8.33 8.32 8.32 7.47 7.47 7.46 7.46 7.45 7.45 7.45 7.44 7.44 7.43 7.43 7.28 7.28 7.27 7.26 7.26 7.26 7.25 7.21 7.21 7.20 7.20 7.19 7.19 7.18 7.07 7.07 7.07 7.06 6.89 6.88 6.88 6.87 6.87 6.86 4.04 4.03 4.03 4.03 4.02 4.01 3.82 3.53 3.52 3.51 3.50 3.02 3.01 3.00 2.99
MeO
OMe
O
N
P
N
OMe
maj-26
1H NMR
(400 MHz, CDCl3)
1.97 8.53 3.45 1.03 2.01 6.32 1.07 9.01 1.15 1.00
9.5 9.0 8.5 8.0 7.5 7.0 6.5 6.0 5.5 5.0 4.5 4.0 3.5 3.0 2.5 2.0 1.5 1.0 0.5 0.0
Chemical Shift (ppm)

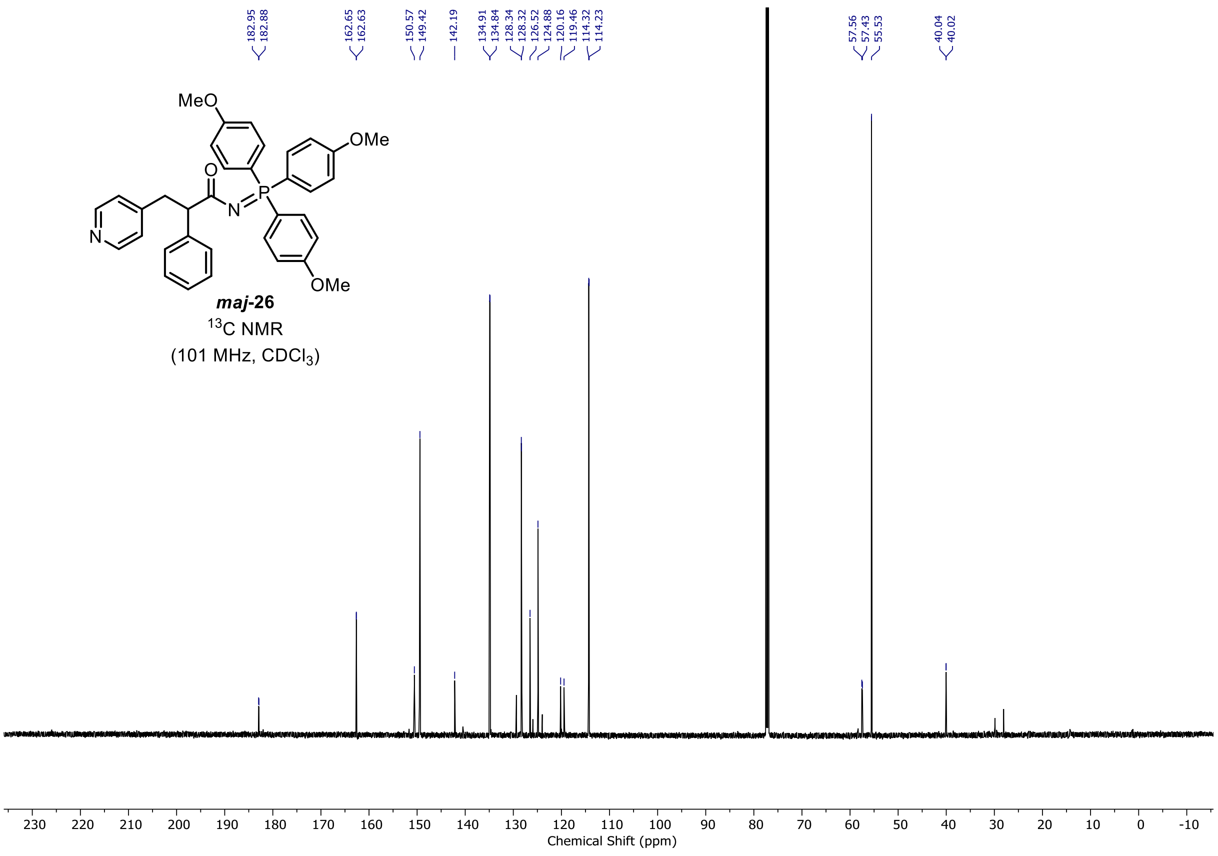
182.95 182.88 162.65 162.63 150.57 149.42 142.19 134.91 134.84 128.34 128.32 126.52 124.88 120.16 119.46 114.32 114.23 57.56 57.43 55.53 40.04 40.02
MeO
OMe
O
N
P
N
OMe
maj-26
13C NMR
(101 MHz, CDCl3)
230 220 210 200 190 180 170 160 150 140 130 120 110 100 90 80 70 60 50 40 30 20 10 0 -10
Chemical Shift (ppm)

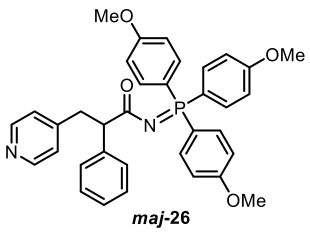

***maj*-26**

^{31}P NMR

(162 MHz, $CDCl_3$)

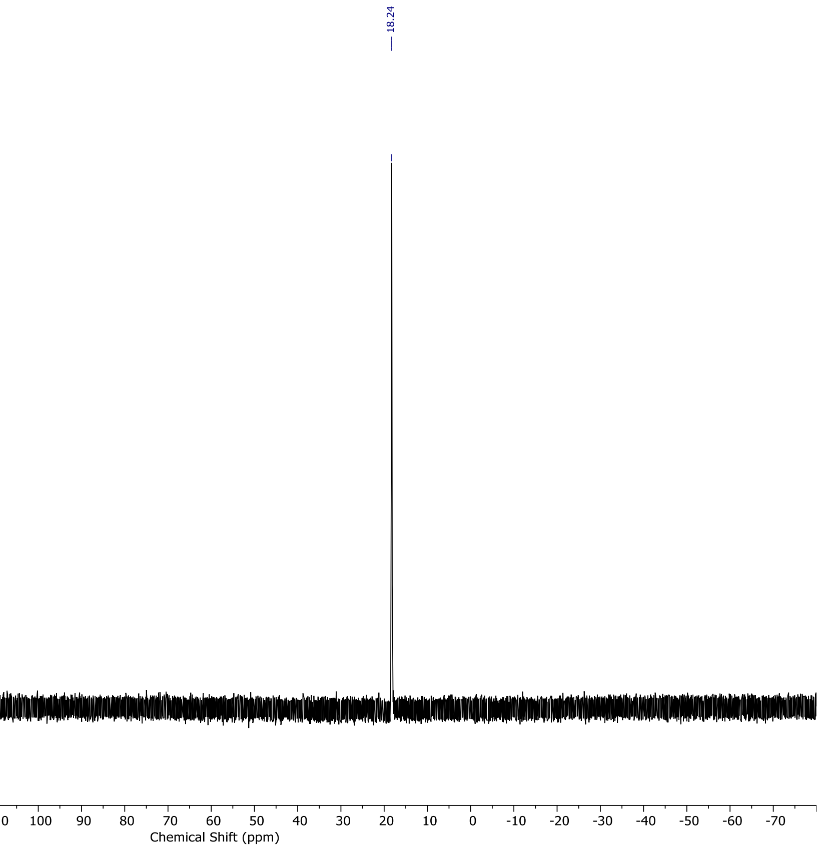

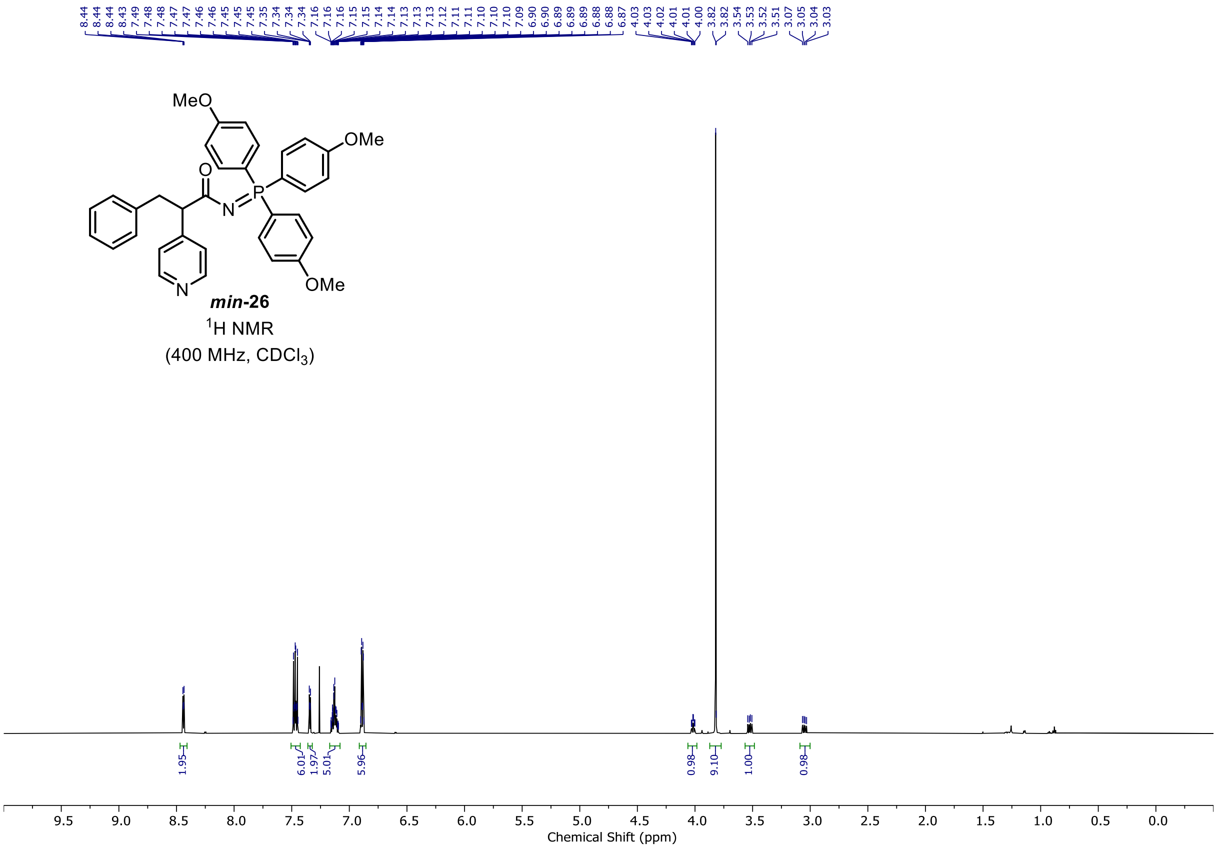
MeO
OMe
OMe
O
N
P
N
min-26
¹H NMR
(400 MHz, CDCl3)
Chemical Shift (ppm)

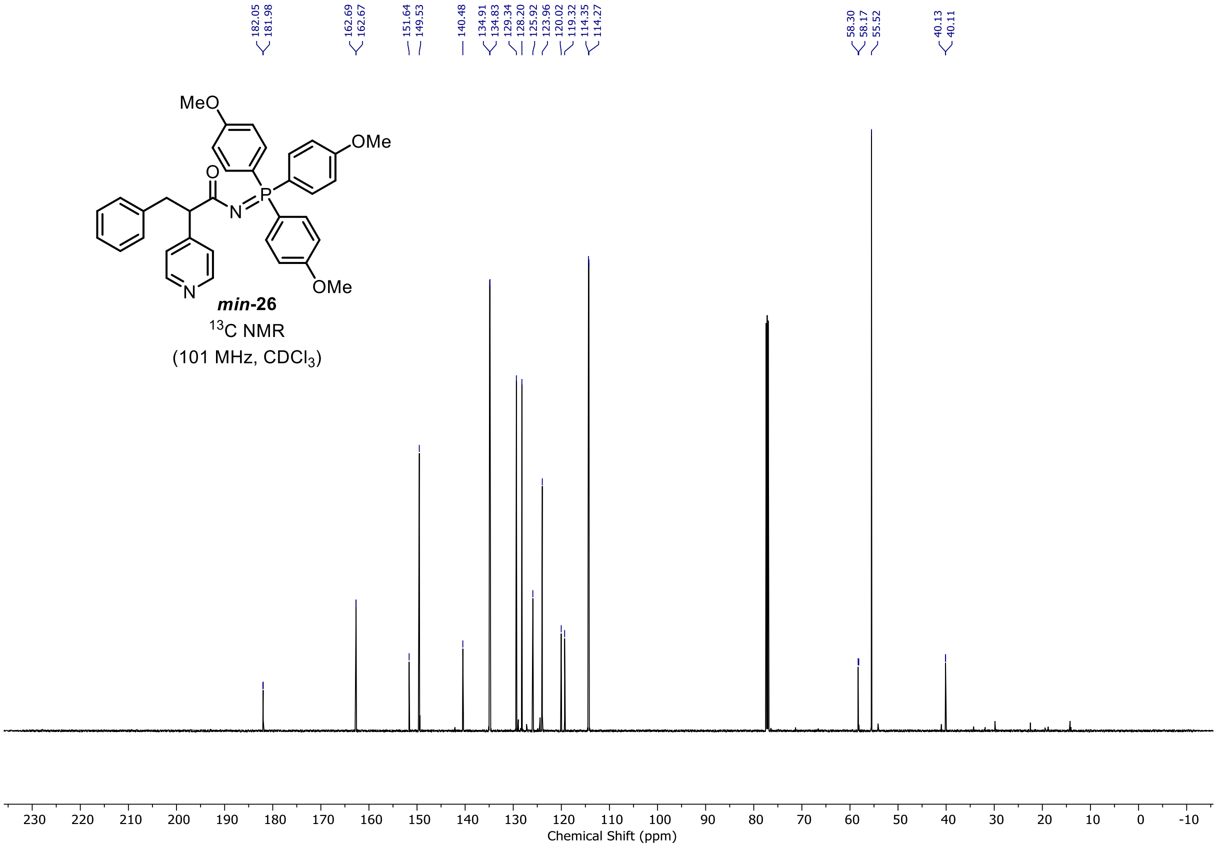
MeO
OMe
OMe
O
N
P
N
min-26
¹³C NMR
(101 MHz, CDCl3)
Chemical Shift (ppm)

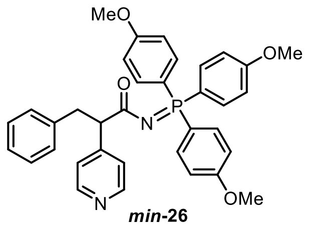

***min*-26**
^{31}P NMR
(162 MHz, $CDCl_3$)

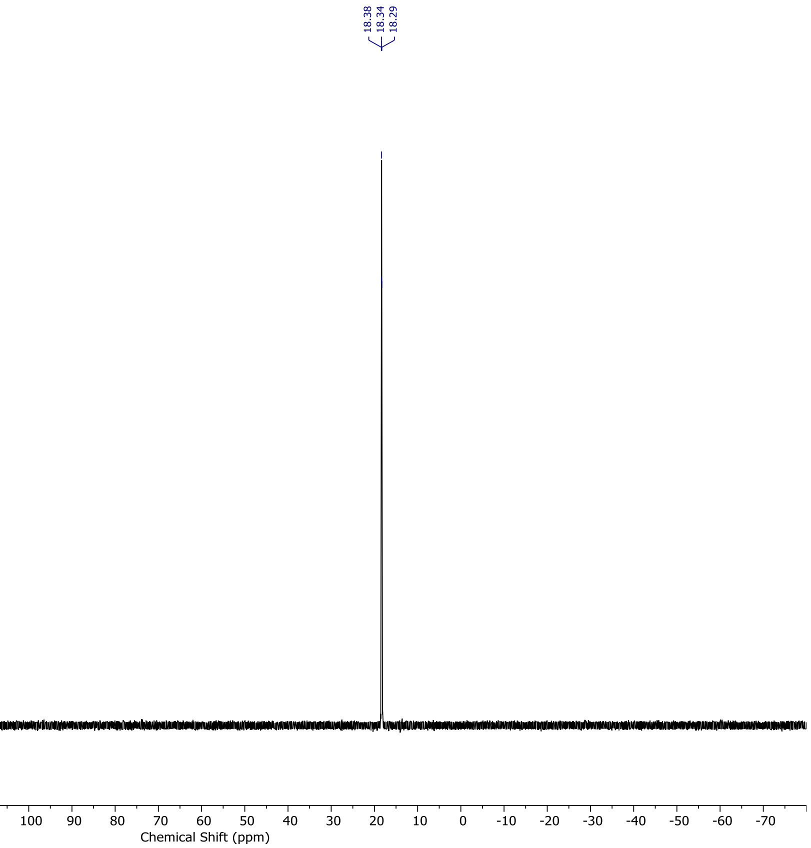

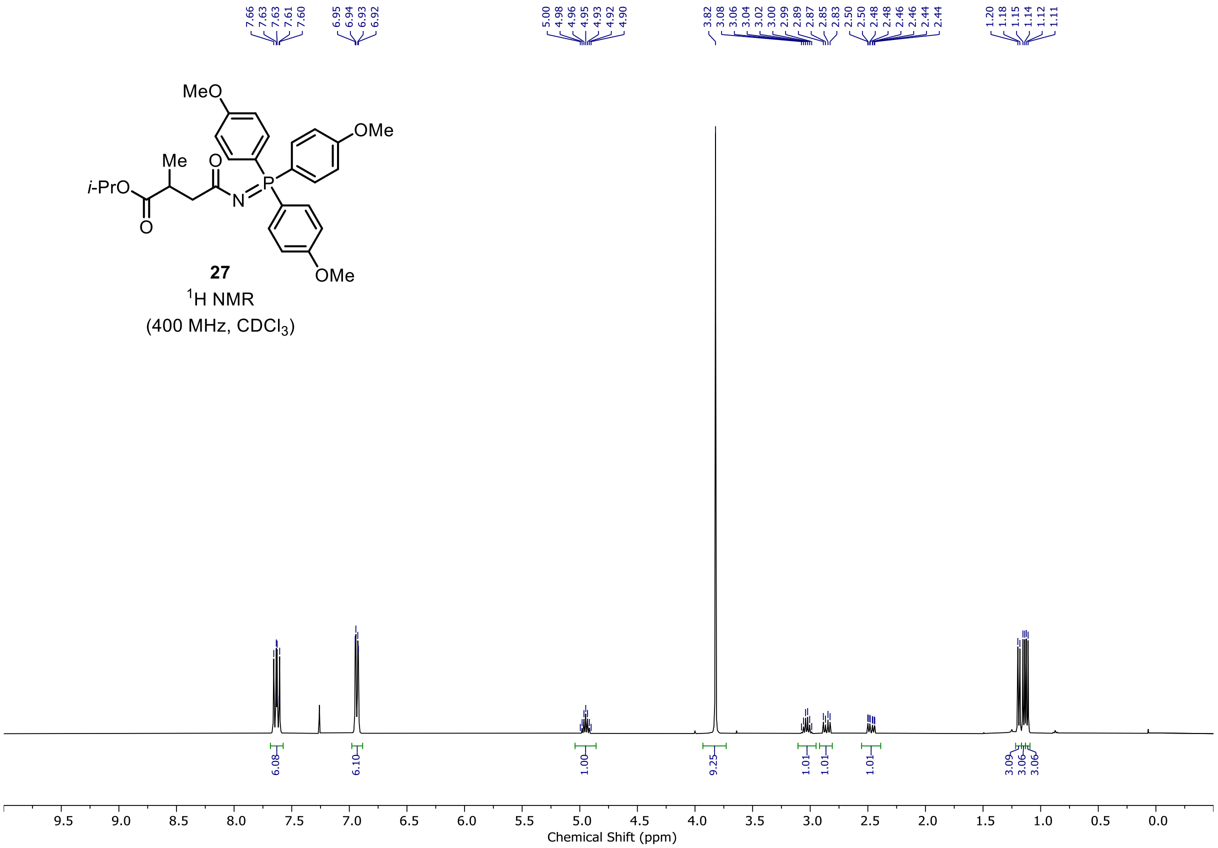

MeO
OMe
Me
O
i-PrO
N
P
O
OMe
27
1H NMR
(400 MHz, CDCl3)
Chemical Shift (ppm)

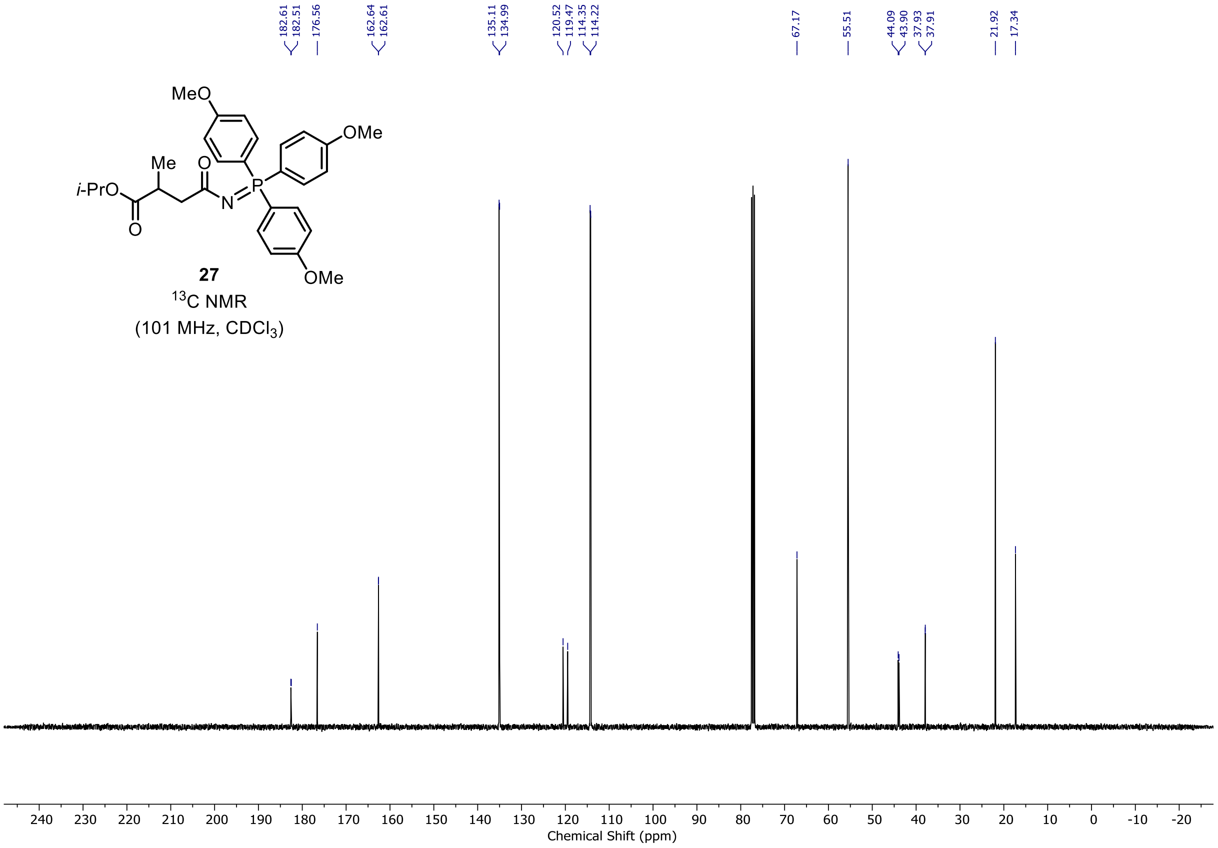

MeO
OMe
Me
O
i-PrO
N
P
O
OMe
27
13C NMR
(101 MHz, CDCl3)
Chemical Shift (ppm)

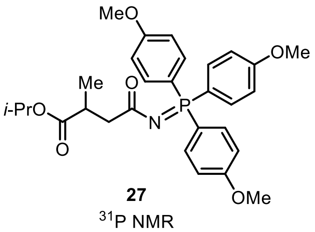

27
^{31}P NMR
(162 MHz, $CDCl_3$)

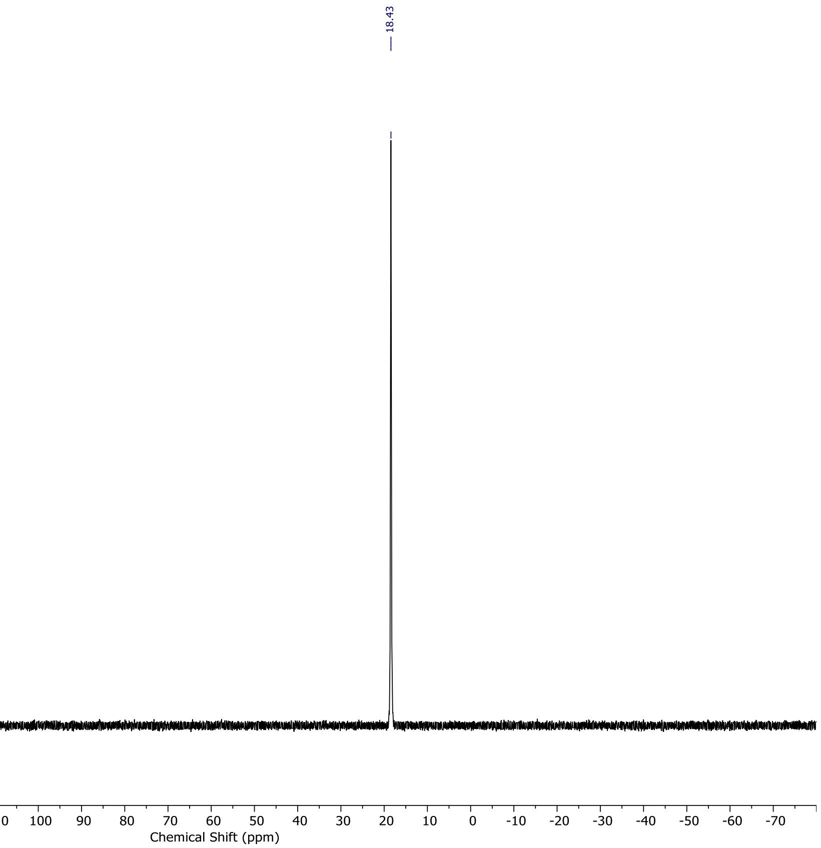

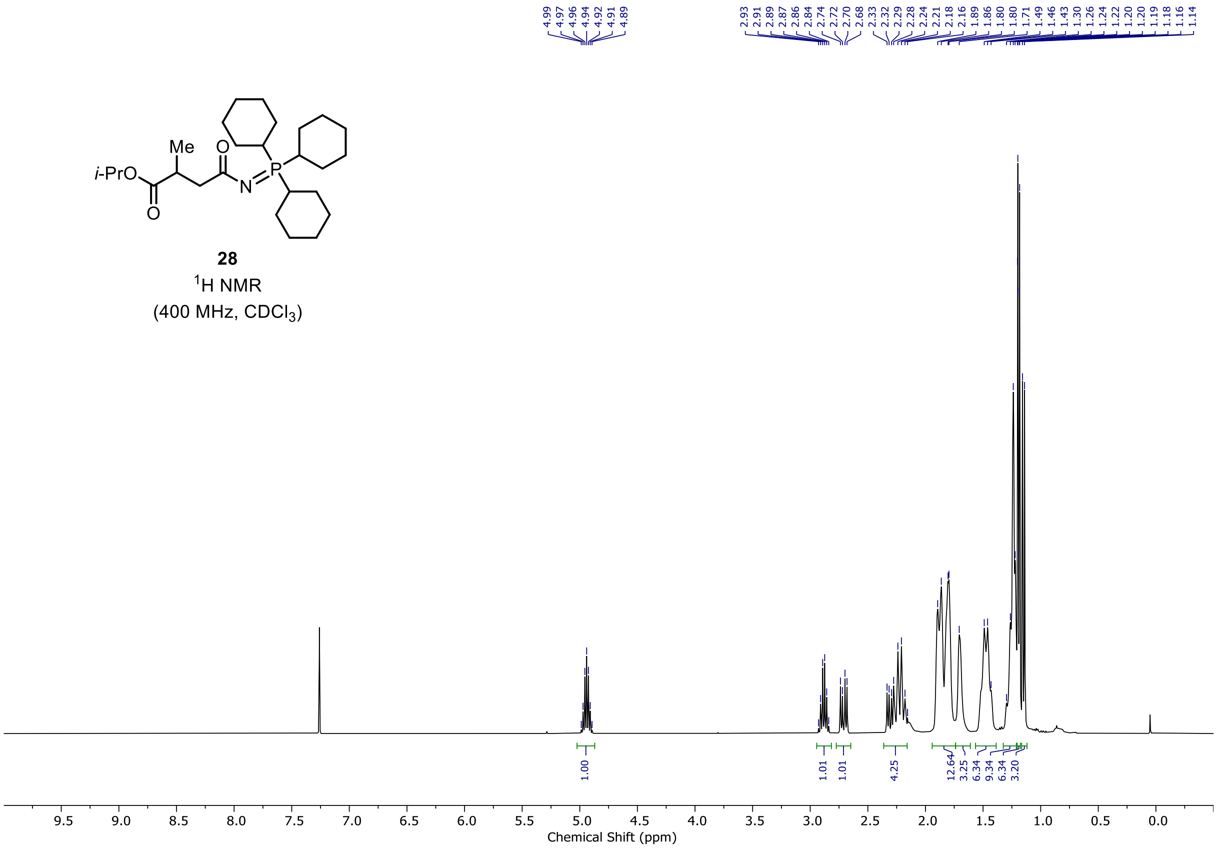

28
^{1}H NMR
(400 MHz, $CDCl_3$)

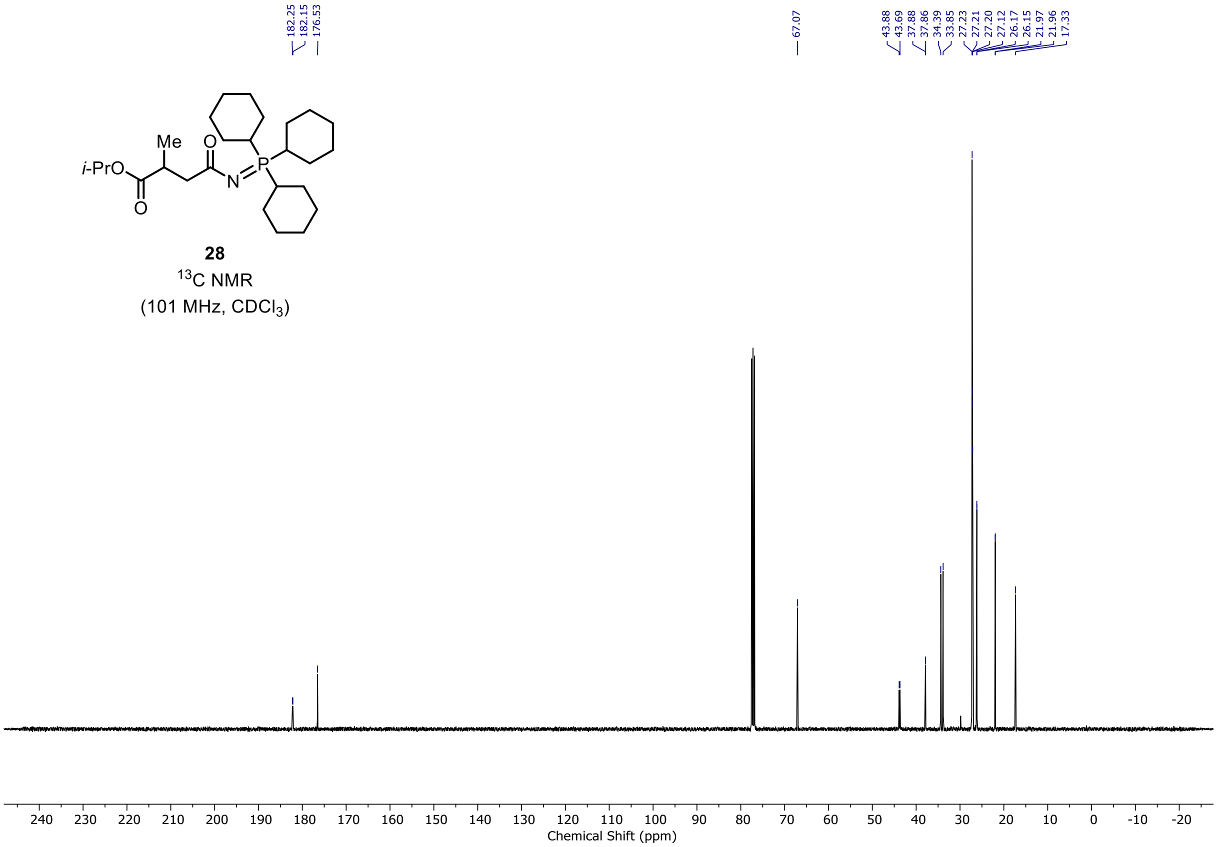

28
^{13}C NMR
(101 MHz, $CDCl_3$)

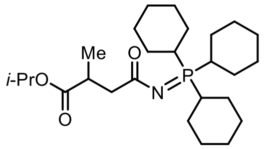

28

^{31}P NMR

(162 MHz, $CDCl_3$)

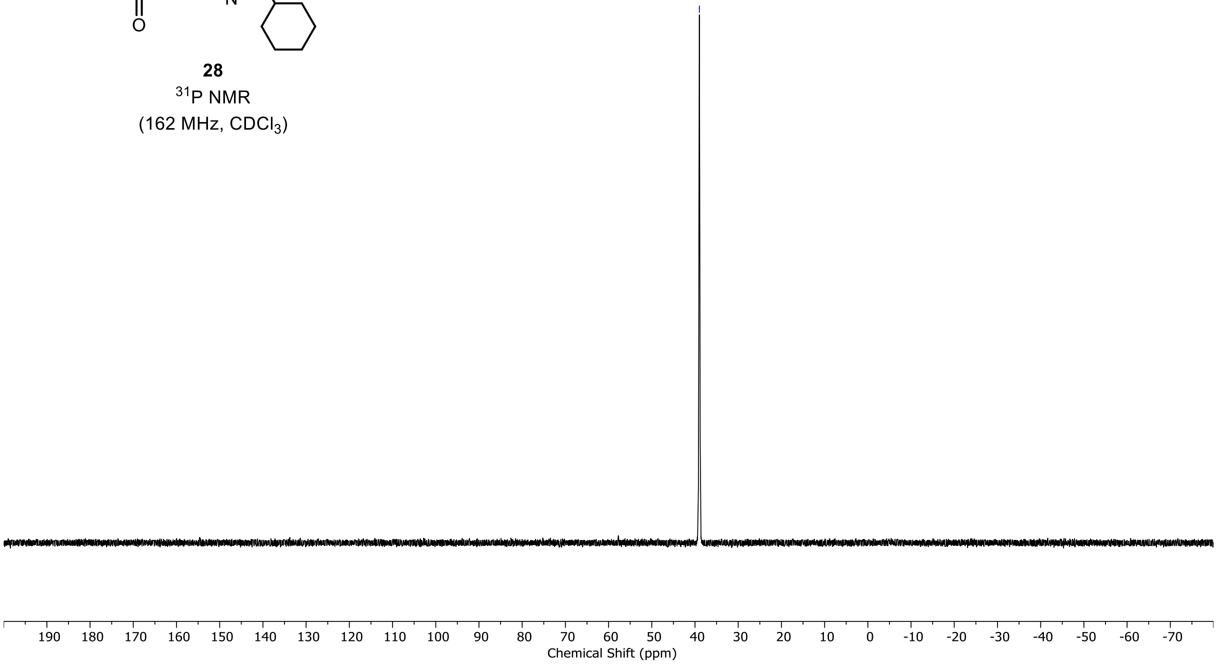

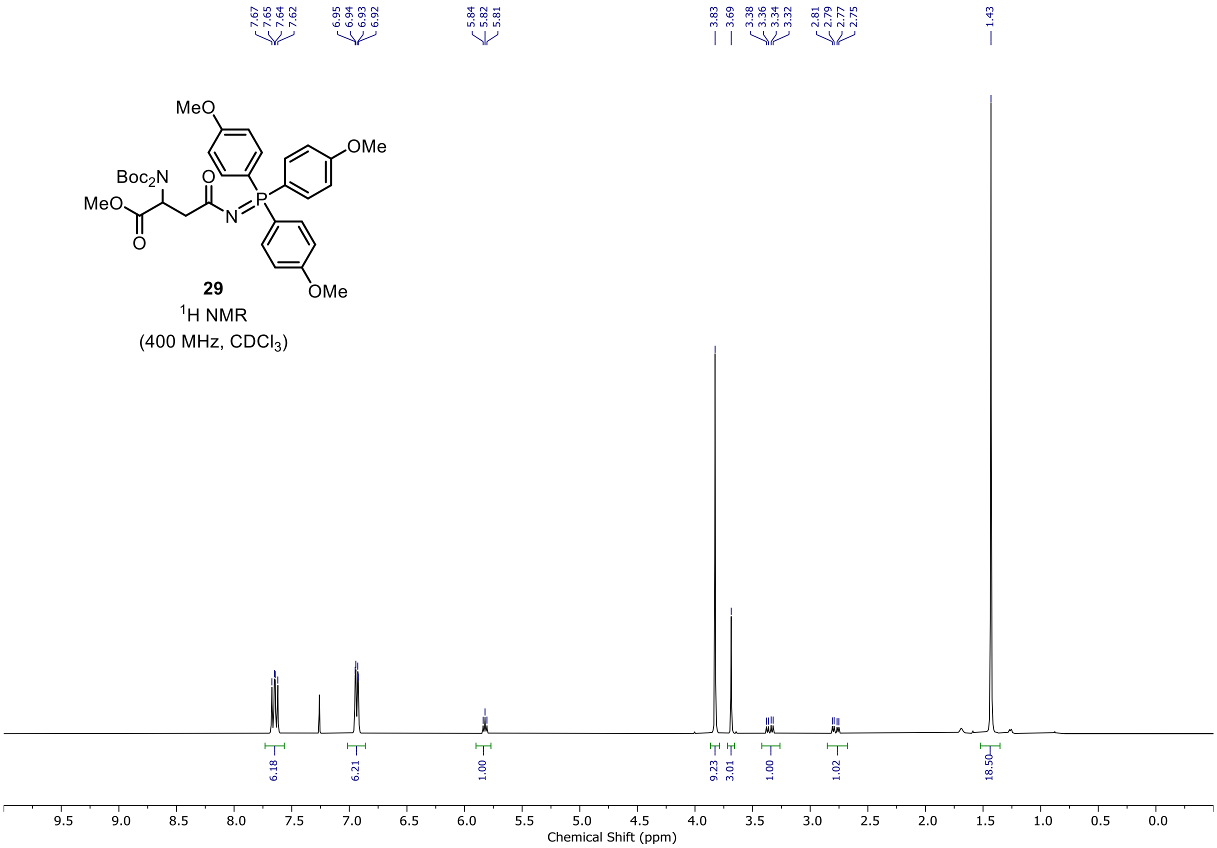
7.67
7.65
7.64
7.62
6.95
6.94
6.93
6.92
5.84
5.82
5.81
3.83
3.69
3.38
3.36
3.34
3.32
2.81
2.79
2.77
2.75
1.43
MeO
OMe
Boc2N
O
MeO
N
P
O
OMe
29
1H NMR
(400 MHz, CDCl3)
6.18
6.21
1.00
9.23
3.01
1.00
1.02
18.50
Chemical Shift (ppm)

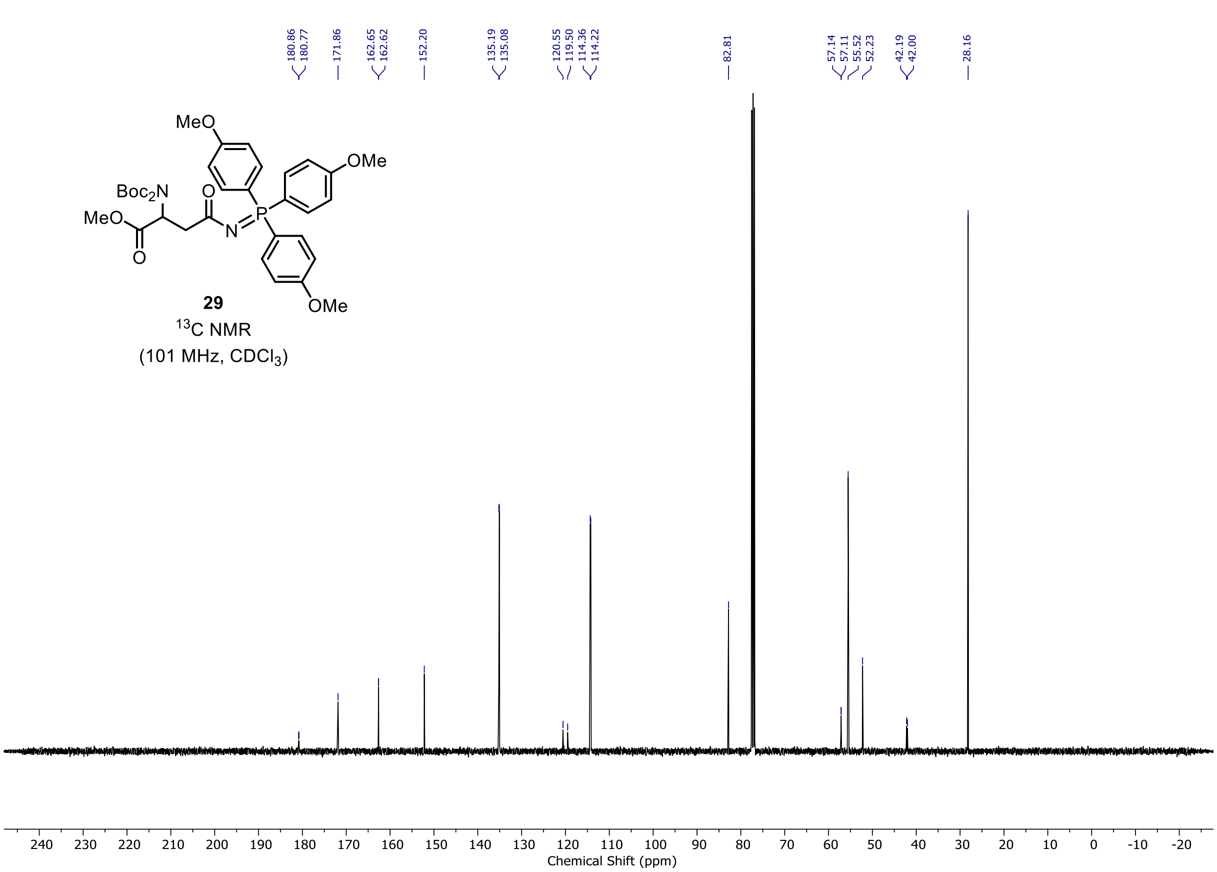
180.86
180.77
171.86
162.65
162.62
152.20
135.19
135.08
120.55
119.50
114.36
114.22
82.81
57.14
57.11
55.52
52.23
42.19
42.00
28.16
MeO
OMe
Boc2N
O
MeO
N
P
O
OMe
29
13C NMR
(101 MHz, CDCl3)
Chemical Shift (ppm)

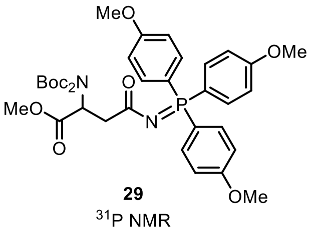

29

^{31}P NMR

(162 MHz, $CDCl_3$)

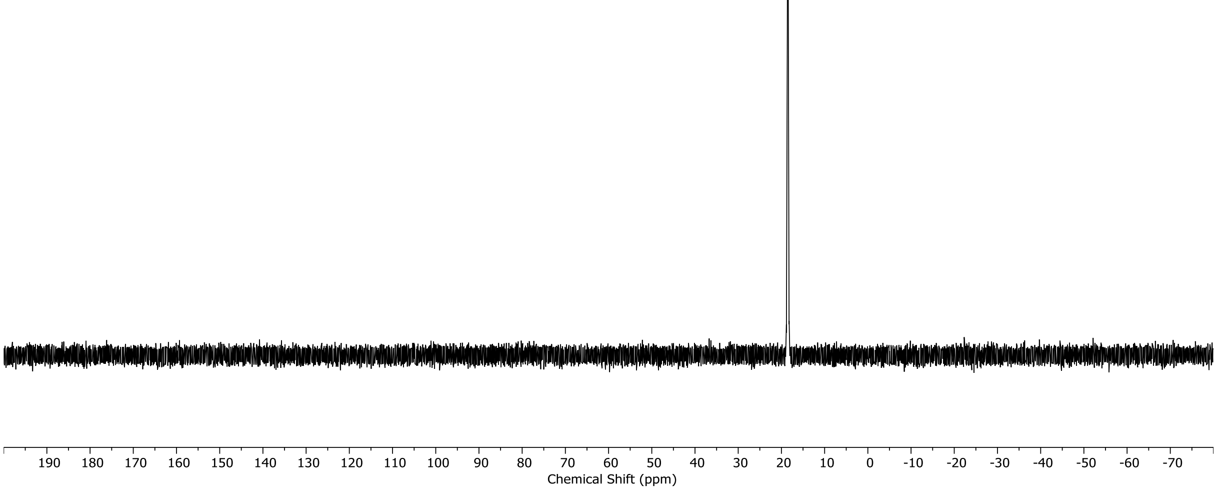

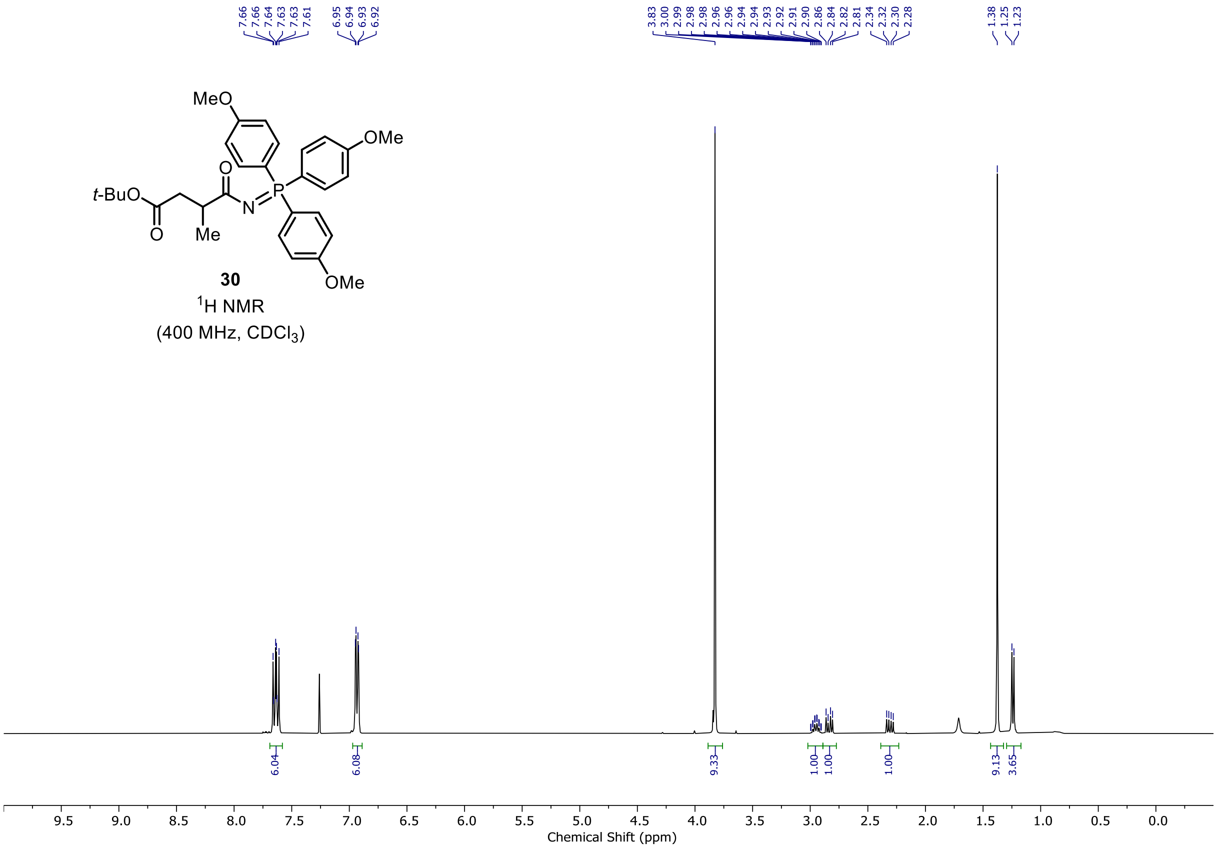
7.66
7.66
7.64
7.63
7.63
7.61
6.95
6.94
6.93
6.92
3.83
3.00
2.99
2.98
2.98
2.96
2.96
2.94
2.94
2.93
2.92
2.91
2.90
2.86
2.84
2.82
2.81
2.34
2.32
2.30
2.28
1.38
1.25
1.23
MeO
OMe
t-BuO
O
N
P
Me
OMe
30
1H NMR
(400 MHz, CDCl3)
6.04
6.08
9.33
1.00
1.00
1.00
9.13
3.65
9.5 9.0 8.5 8.0 7.5 7.0 6.5 6.0 5.5 5.0 4.5 4.0 3.5 3.0 2.5 2.0 1.5 1.0 0.5 0.0
Chemical Shift (ppm)

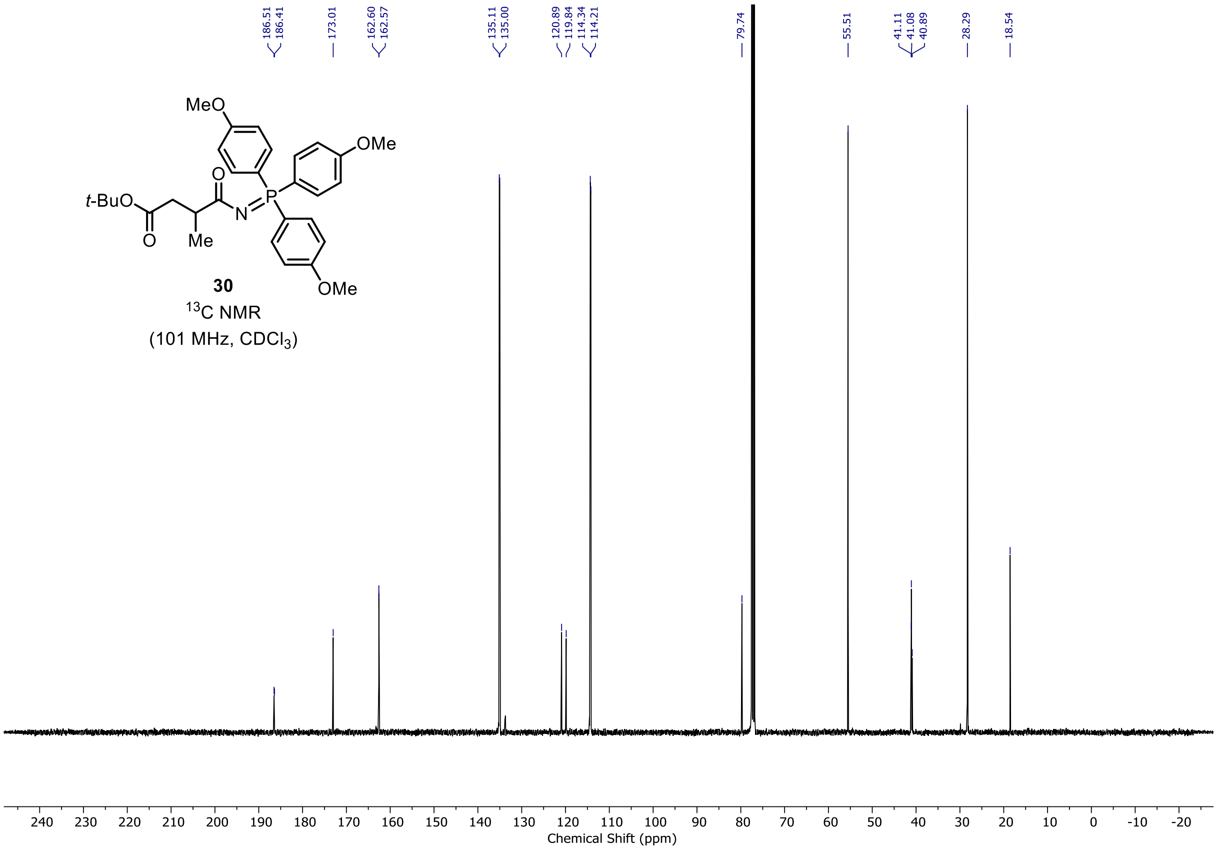
186.51
186.41
173.01
162.60
162.57
135.11
135.00
120.89
119.84
114.34
114.21
79.74
55.51
41.11
41.08
40.89
28.29
18.54
MeO
OMe
t-BuO
O
N
P
Me
OMe
30
13C NMR
(101 MHz, CDCl3)
240 230 220 210 200 190 180 170 160 150 140 130 120 110 100 90 80 70 60 50 40 30 20 10 0 -10 -20
Chemical Shift (ppm)

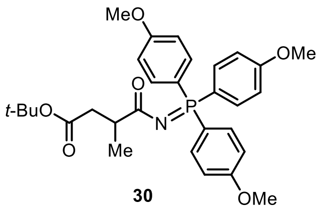

30

^{31}P NMR

(162 MHz, $CDCl_3$)

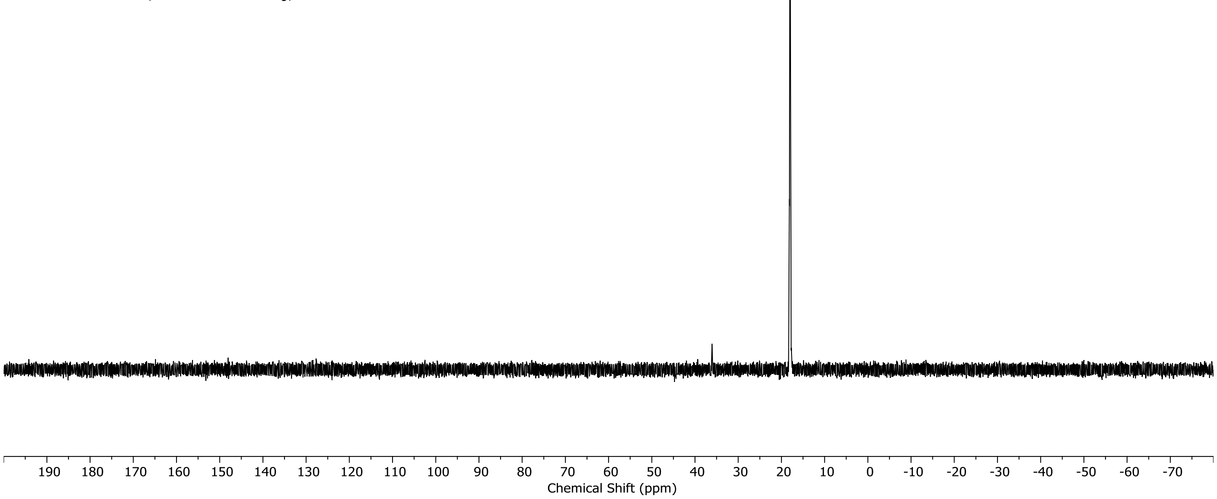

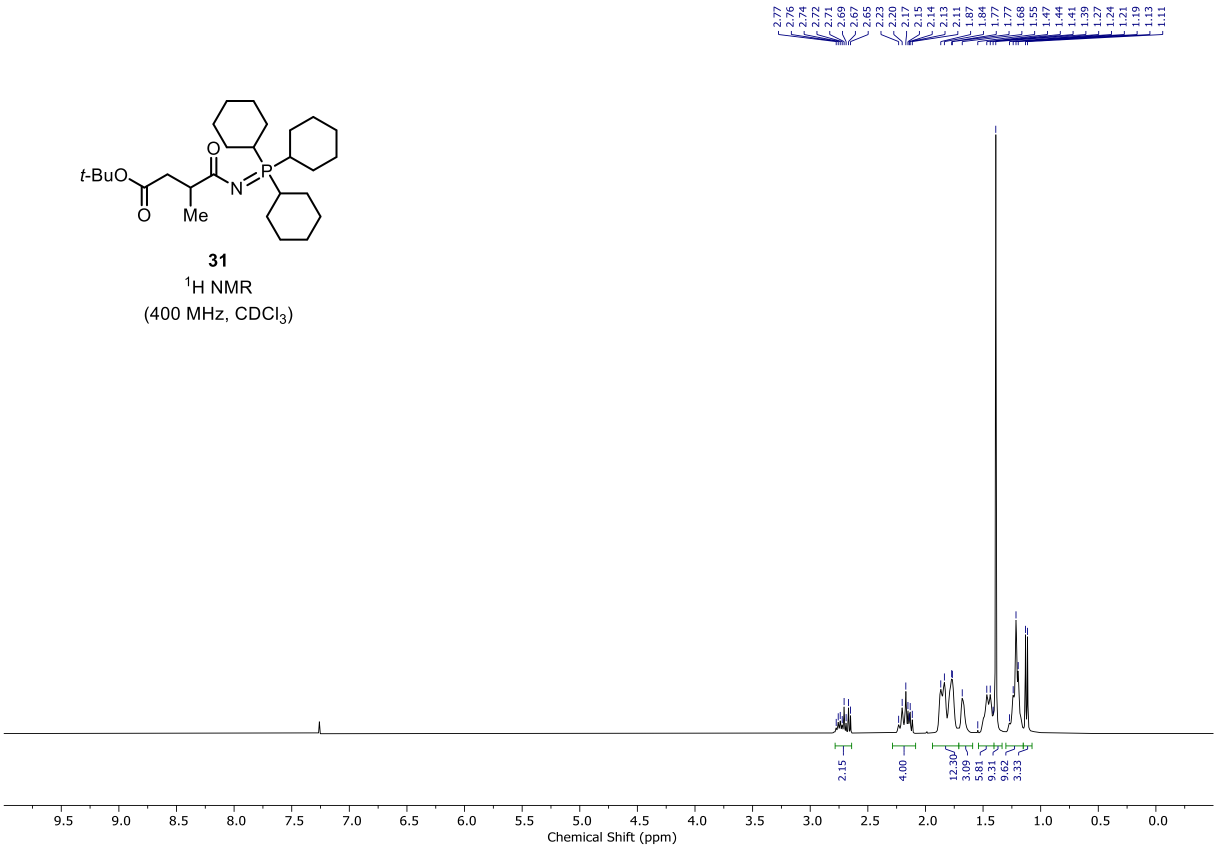
2.77 2.76 2.74 2.72 2.71 2.69 2.67 2.65 2.23 2.20 2.17 2.15 2.14 2.13 2.11 1.87 1.84 1.77 1.77 1.68 1.55 1.47 1.44 1.41 1.39 1.27 1.24 1.21 1.19 1.13 1.11
t-BuO
O
Me
N
P
O
31
¹H NMR
(400 MHz, CDCl3)
2.15
4.00
12.30
3.09
5.81
9.31
9.62
3.33
9.5 9.0 8.5 8.0 7.5 7.0 6.5 6.0 5.5 5.0 4.5 4.0 3.5 3.0 2.5 2.0 1.5 1.0 0.5 0.0
Chemical Shift (ppm)

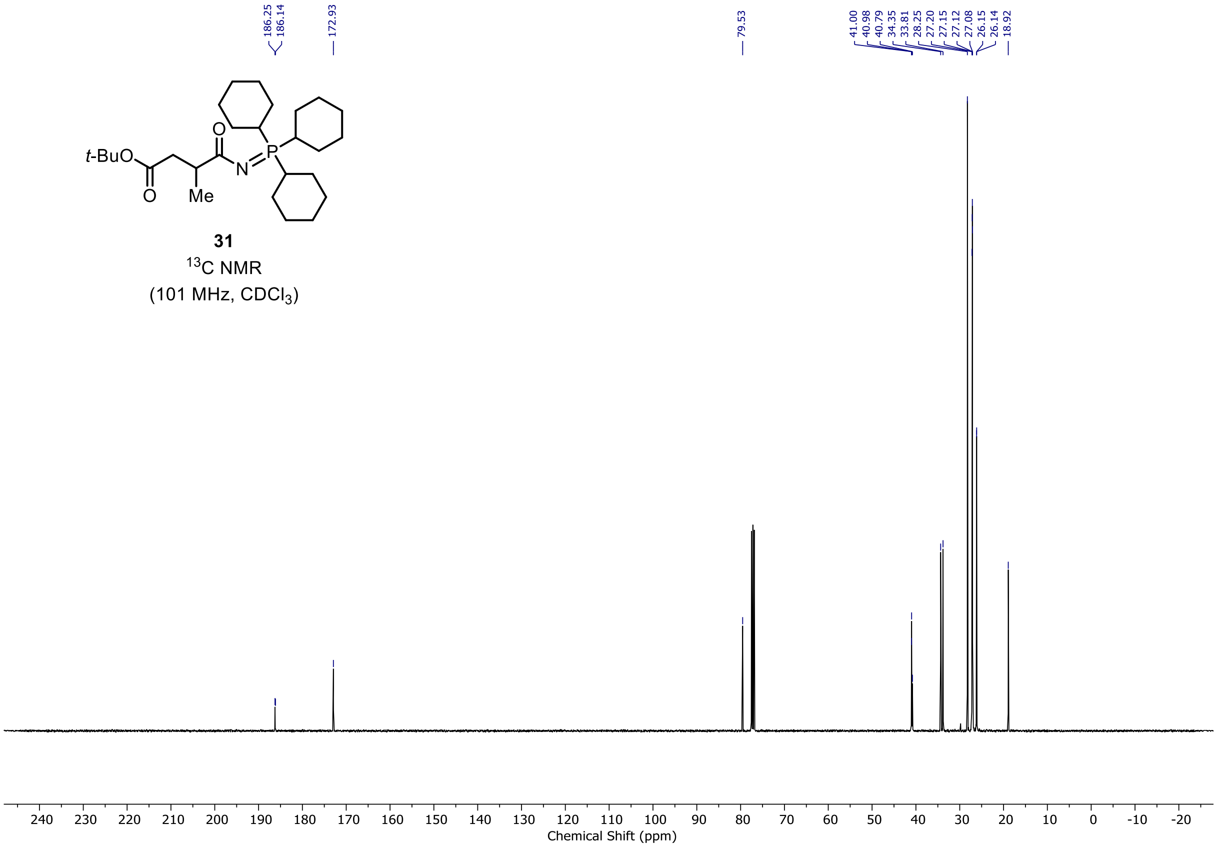
186.25 186.14
172.93
79.53
41.00 40.98 40.79 34.35 33.81 28.25 27.20 27.15 27.12 27.08 26.15 26.14 18.92
t-BuO
O
Me
N
P
O
31
¹³C NMR
(101 MHz, CDCl3)
240 230 220 210 200 190 180 170 160 150 140 130 120 110 100 90 80 70 60 50 40 30 20 10 0 -10 -20
Chemical Shift (ppm)

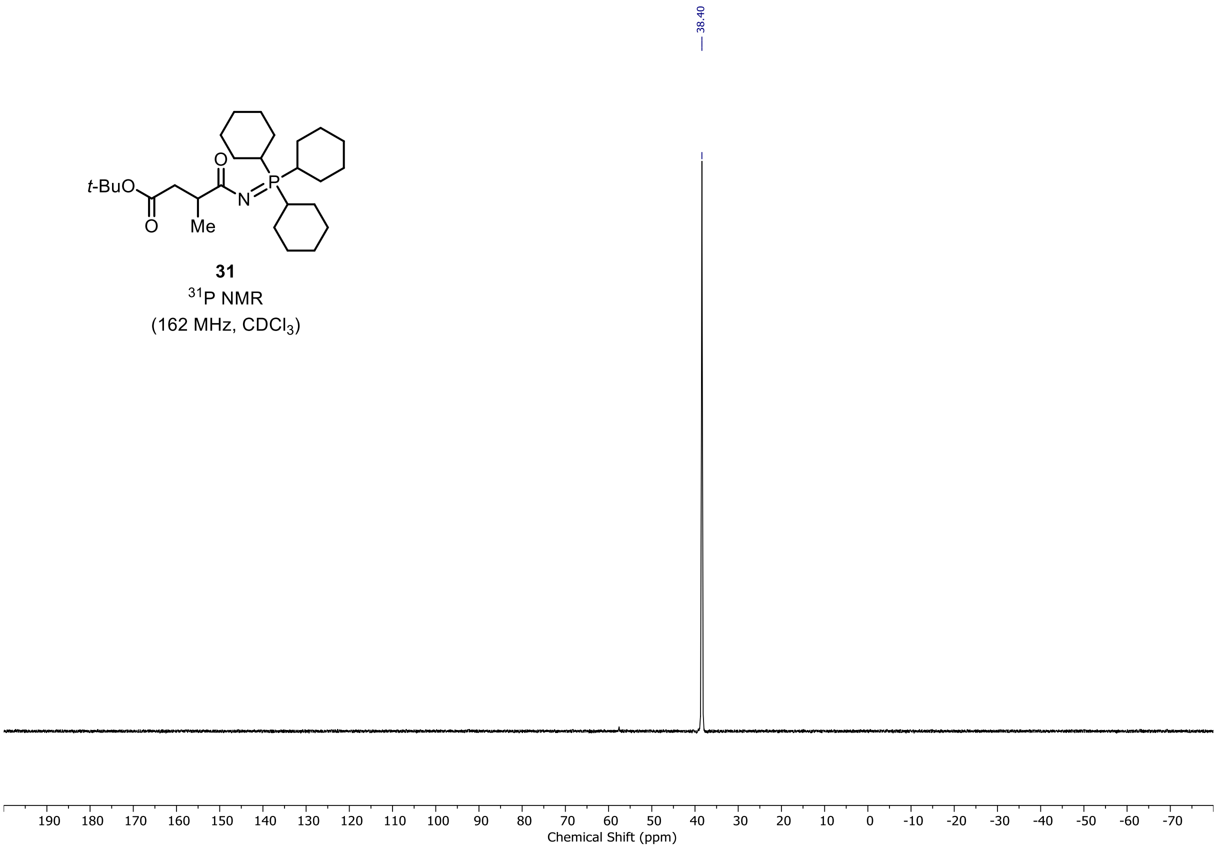

t-BuO
Me
31
31P NMR
(162 MHz, CDCl3)
38.40
190 180 170 160 150 140 130 120 110 100 90 80 70 60 50 40 30 20 10 0 -10 -20 -30 -40 -50 -60 -70
Chemical Shift (ppm)

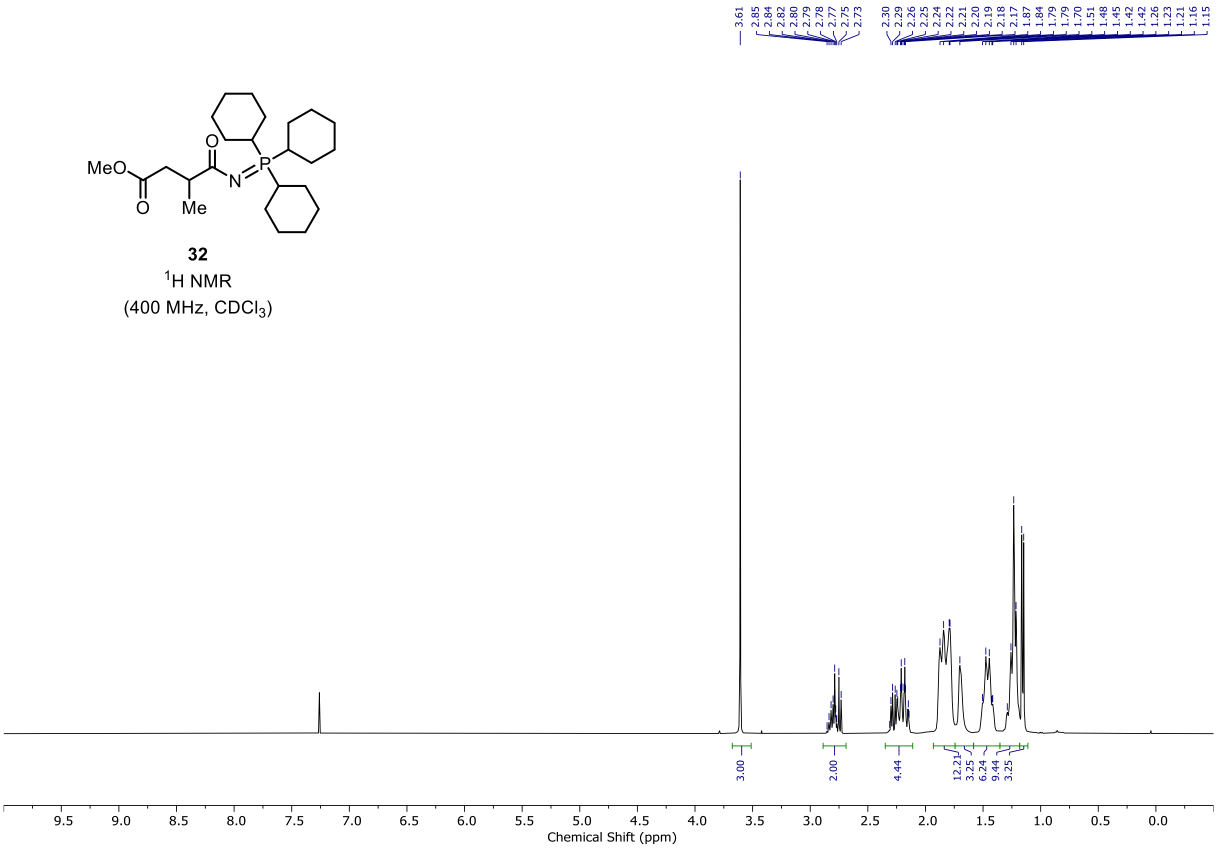

32
^{1}H NMR
(400 MHz, $CDCl_3$)

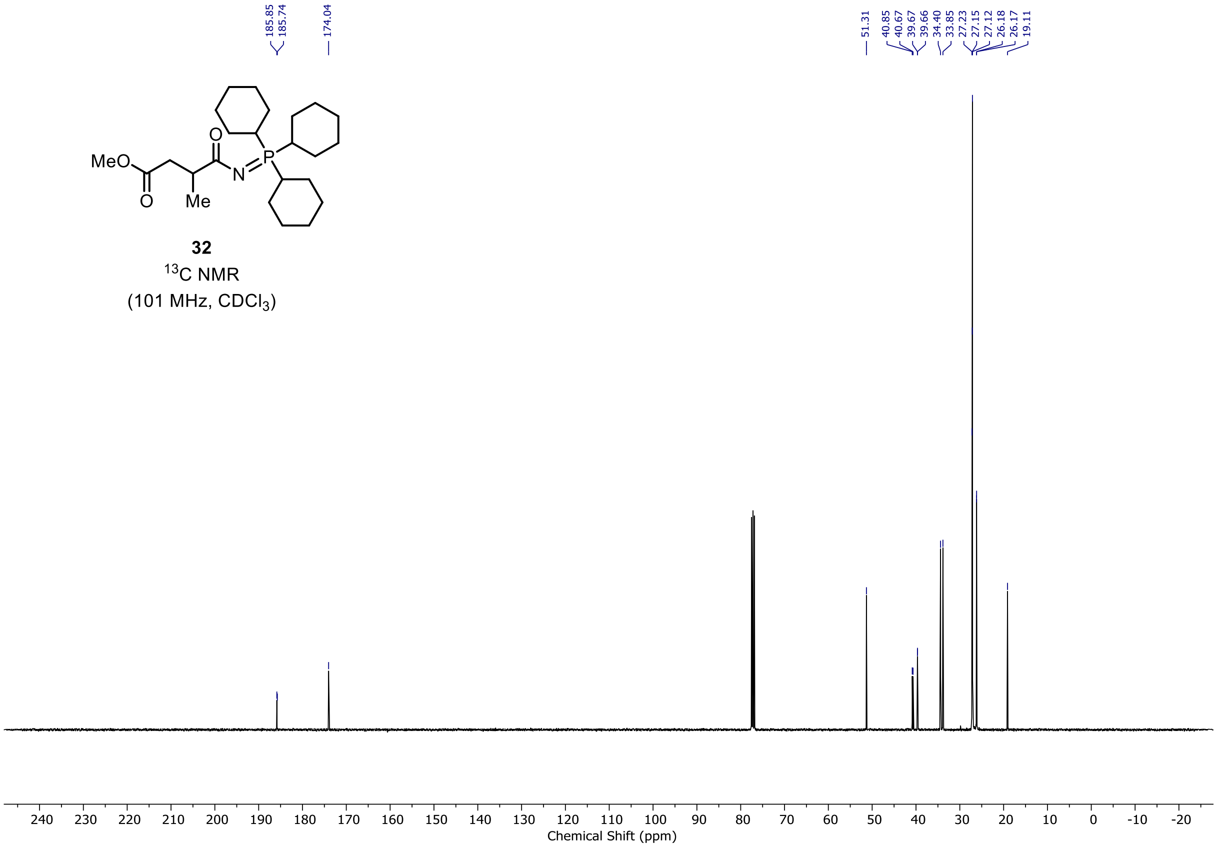

32
^{13}C NMR
(101 MHz, $CDCl_3$)

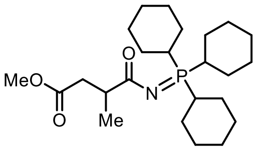

32
^{31}P NMR
(162 MHz, $CDCl_3$)

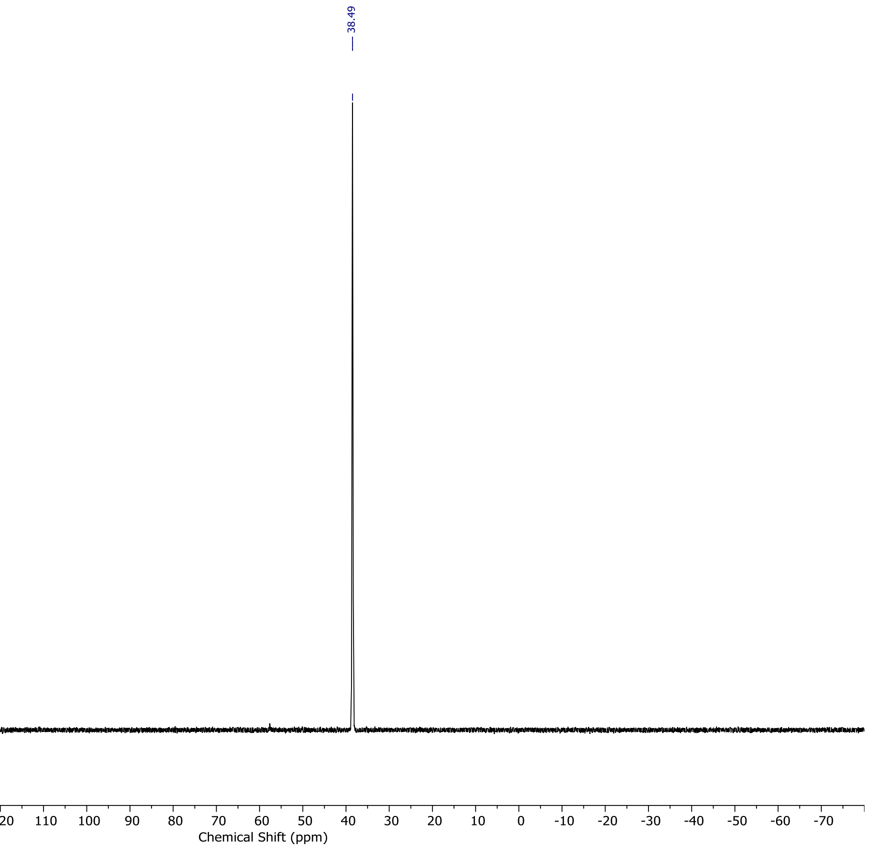

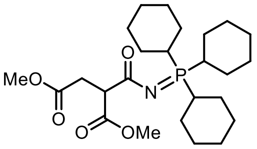

33
^{1}H NMR
(400 MHz, $CDCl_3$)

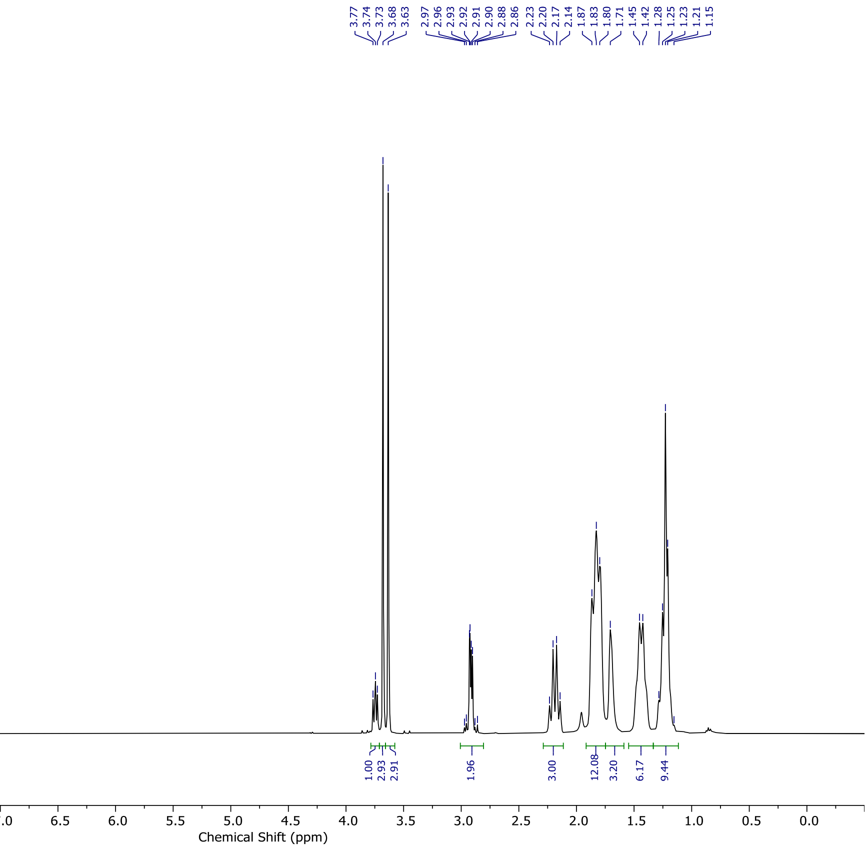

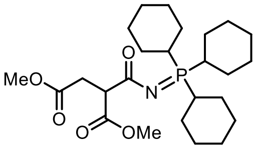

33
^{13}C NMR
(101 MHz, $CDCl_3$)

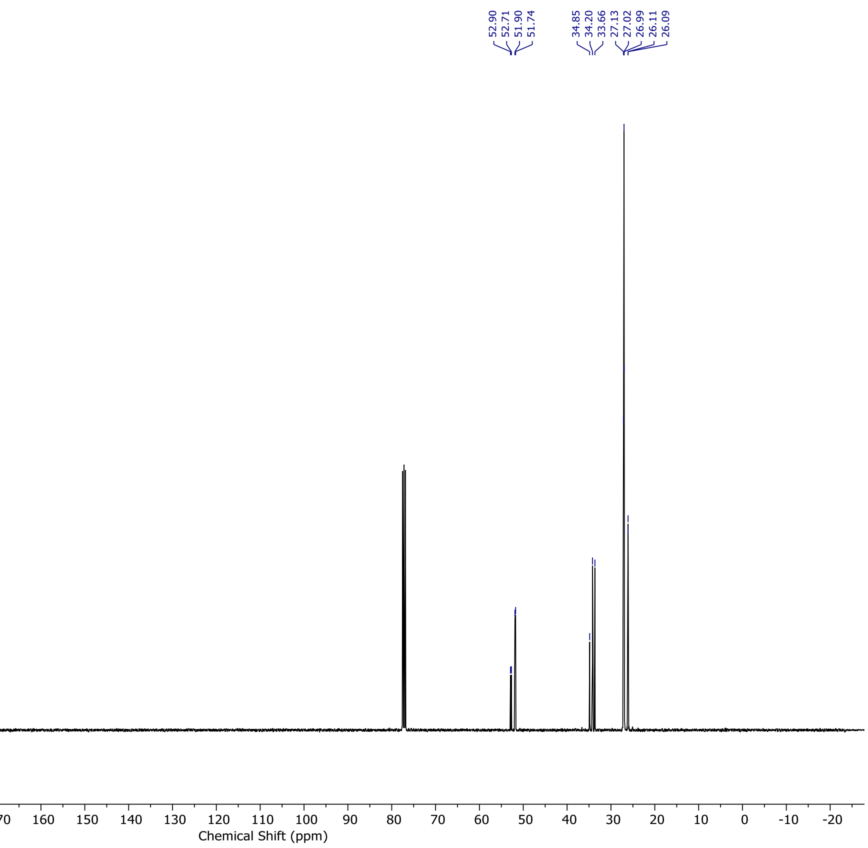

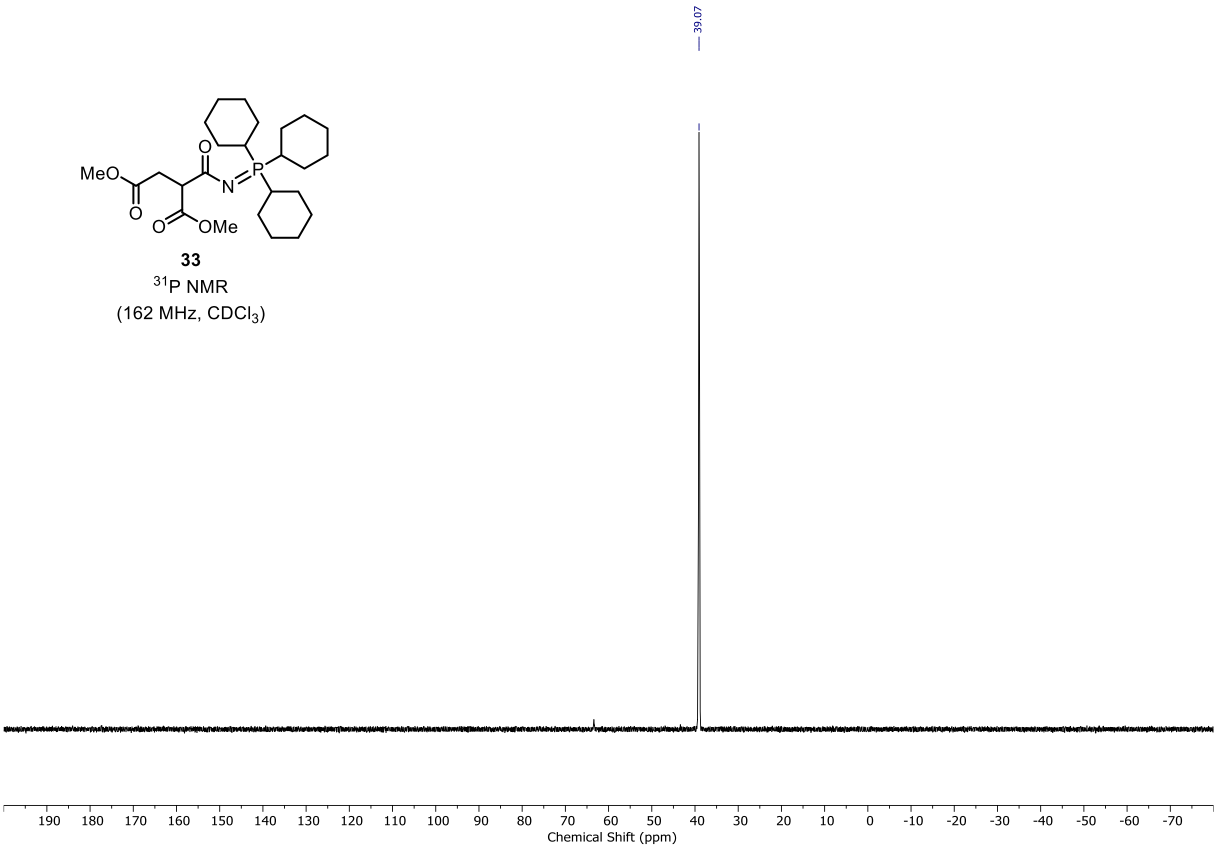

33

^{31}P NMR

(162 MHz, $CDCl_3$)

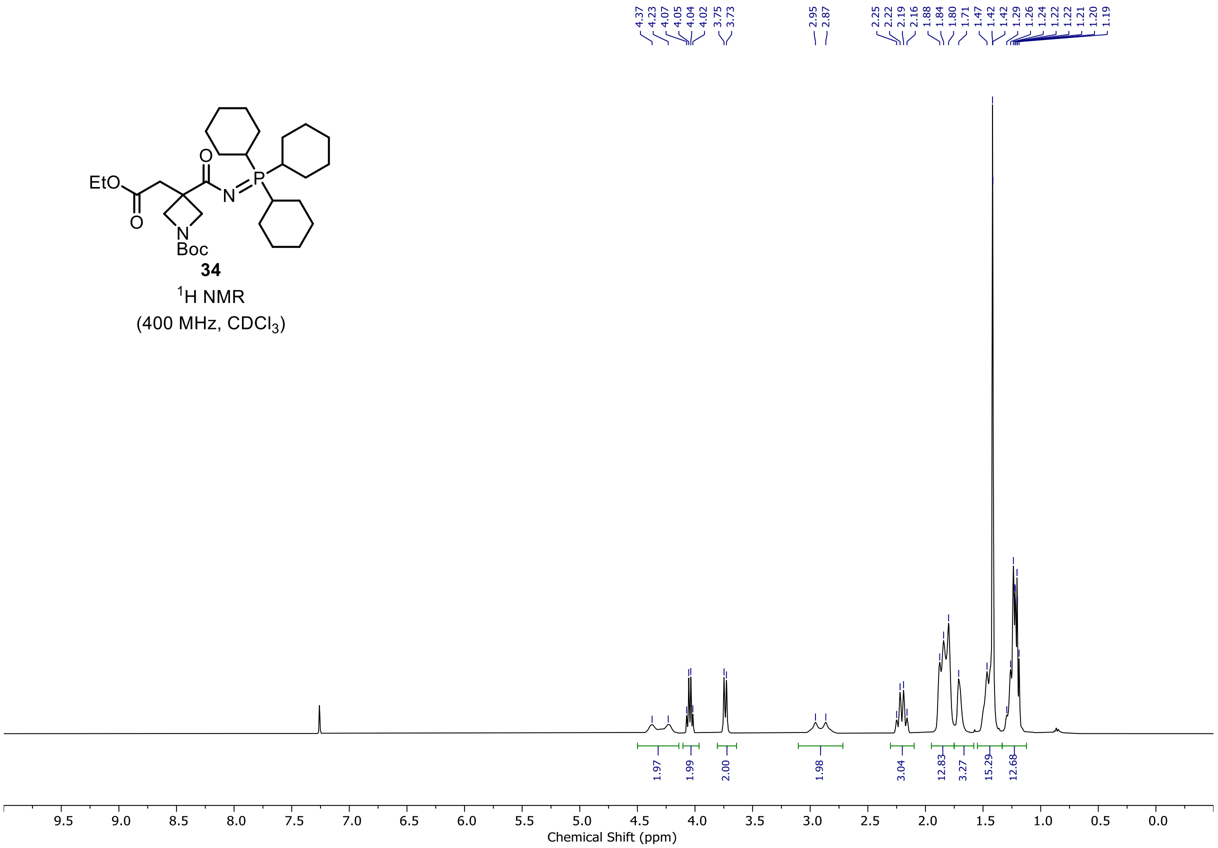
4.37 4.23 4.07 4.05 4.04 4.02 3.75 3.73 2.95 2.87 2.25 2.22 2.19 2.16 1.88 1.84 1.80 1.71 1.47 1.42 1.29 1.26 1.24 1.22 1.22 1.21 1.20 1.19
EtO
O
O
N
P
N
Boc
34
^{1}H NMR
(400 MHz, $CDCl_3$)
1.97 1.99 2.00 1.98 3.04 12.83 3.27 15.29 12.68
9.5 9.0 8.5 8.0 7.5 7.0 6.5 6.0 5.5 5.0 4.5 4.0 3.5 3.0 2.5 2.0 1.5 1.0 0.5 0.0
Chemical Shift (ppm)

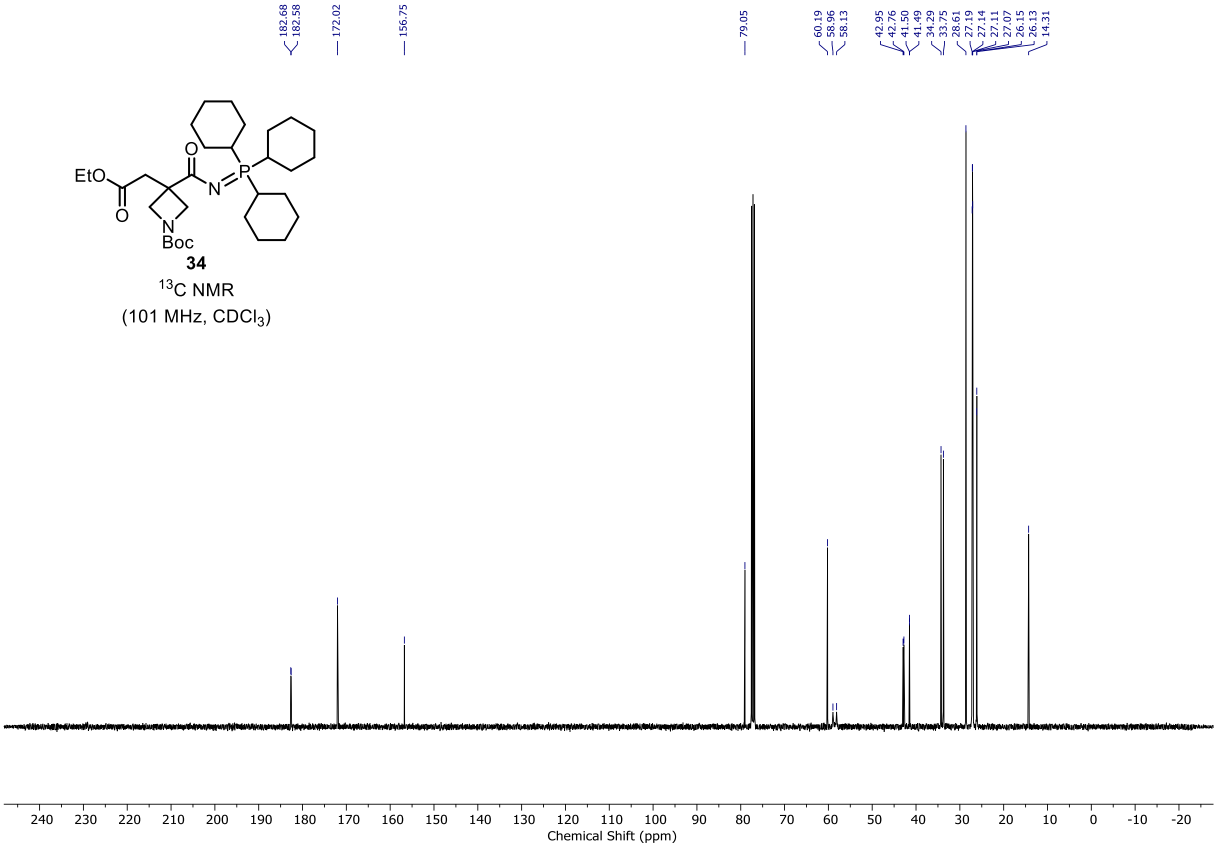
182.68 182.58 172.02 156.75 79.05 60.19 58.96 58.13 42.95 42.76 41.50 41.49 34.29 33.75 28.61 27.19 27.14 27.11 27.07 26.15 26.13 14.31
EtO
O
O
N
P
N
Boc
34
^{13}C NMR
(101 MHz, $CDCl_3$)
240 230 220 210 200 190 180 170 160 150 140 130 120 110 100 90 80 70 60 50 40 30 20 10 0 -10 -20
Chemical Shift (ppm)

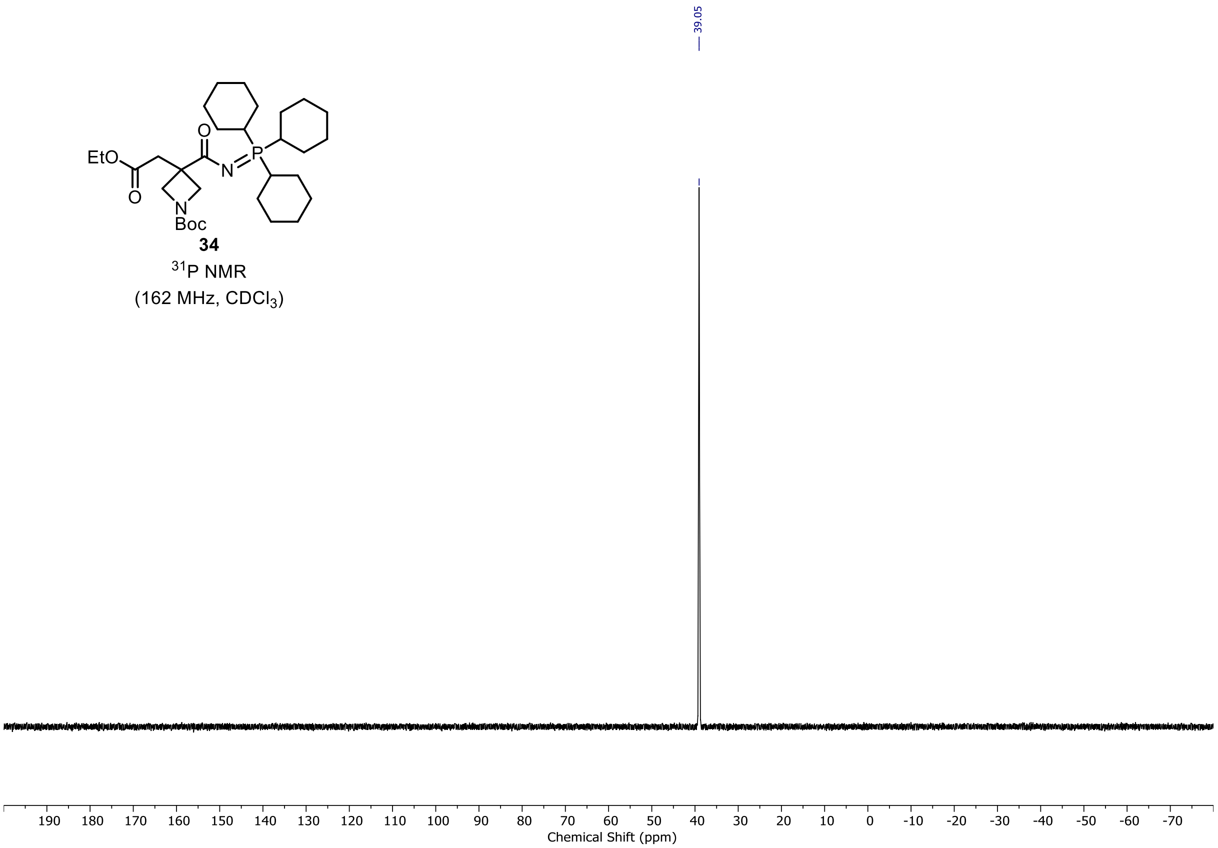

34

^{31}P NMR

(162 MHz, $CDCl_3$)

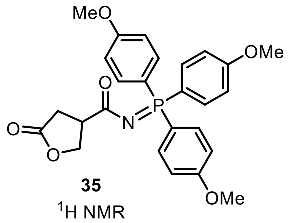

35

^{1}H NMR

(400 MHz, $CDCl_3$)

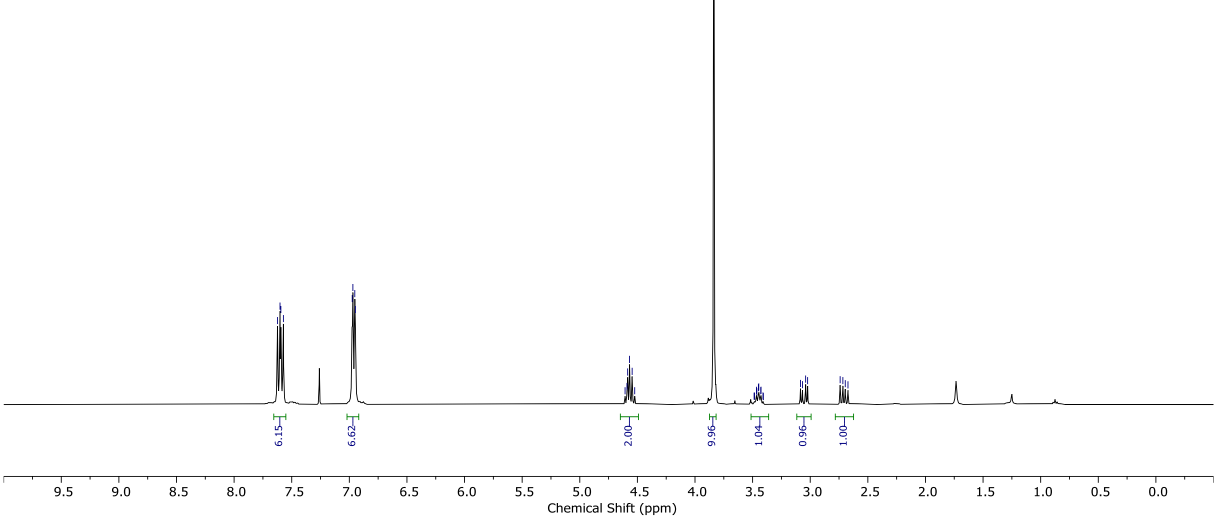

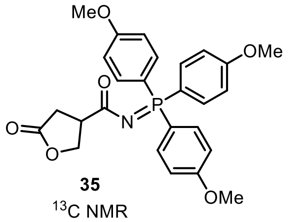

35

^{13}C NMR

(101 MHz, $CDCl_3$)

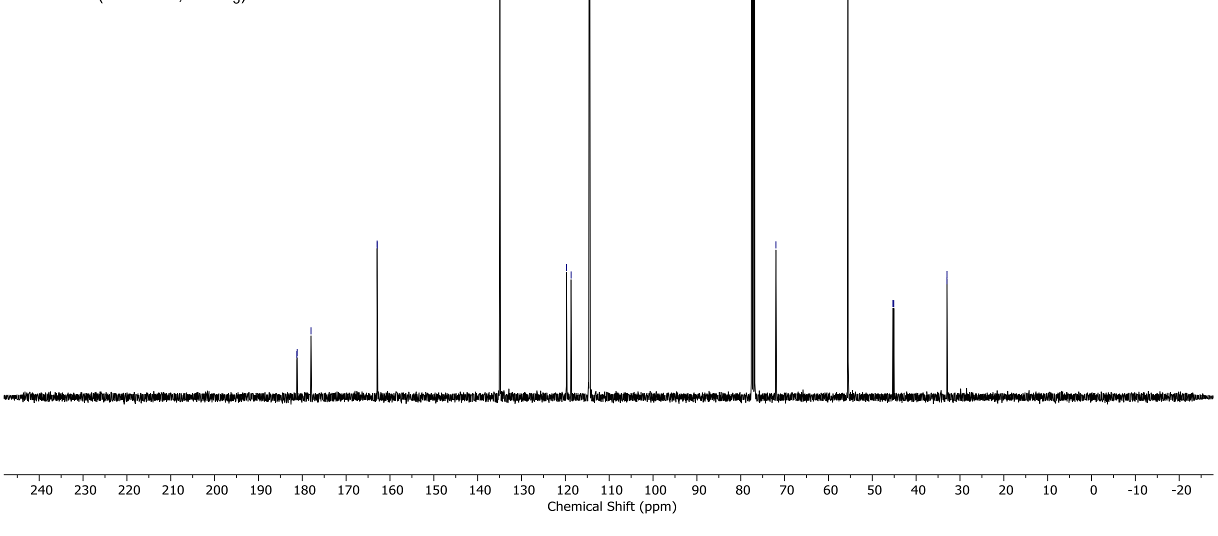

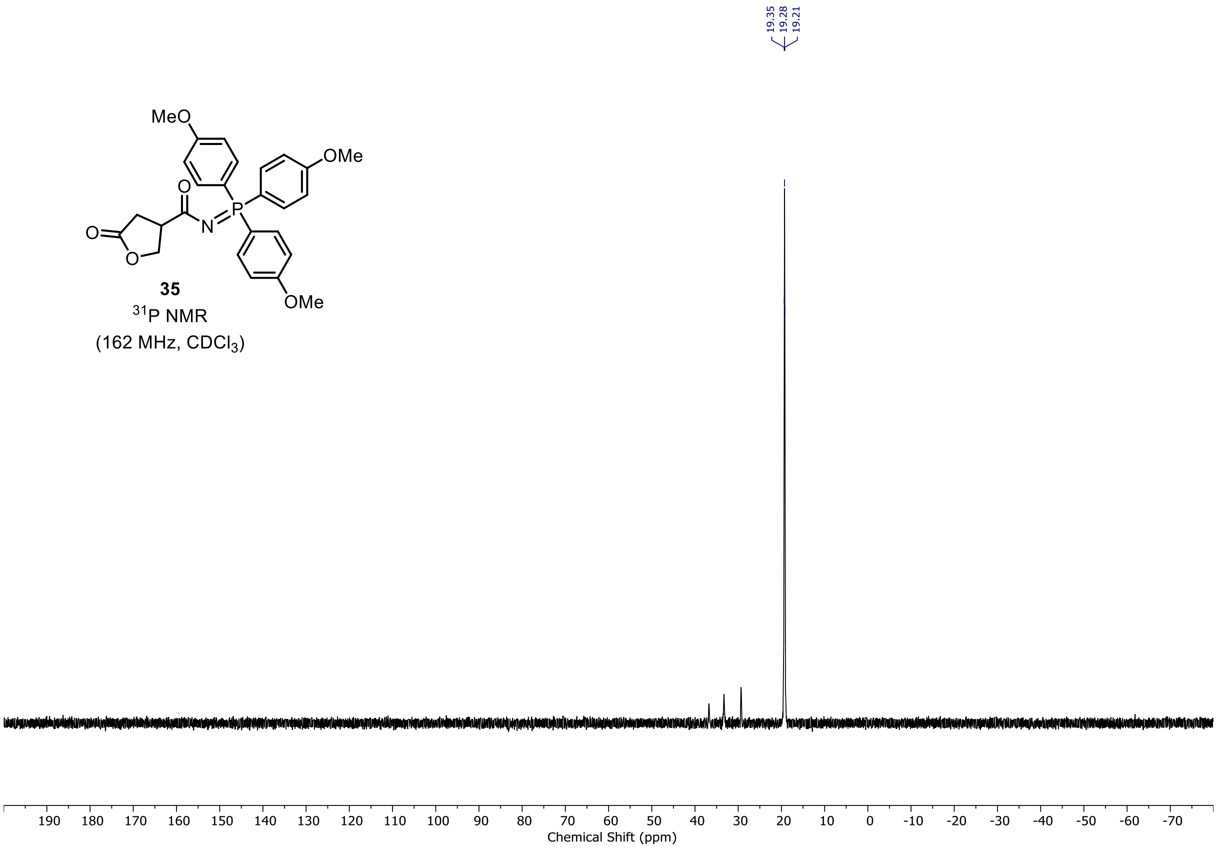

35
^{31}P NMR
(162 MHz, $CDCl_3$)

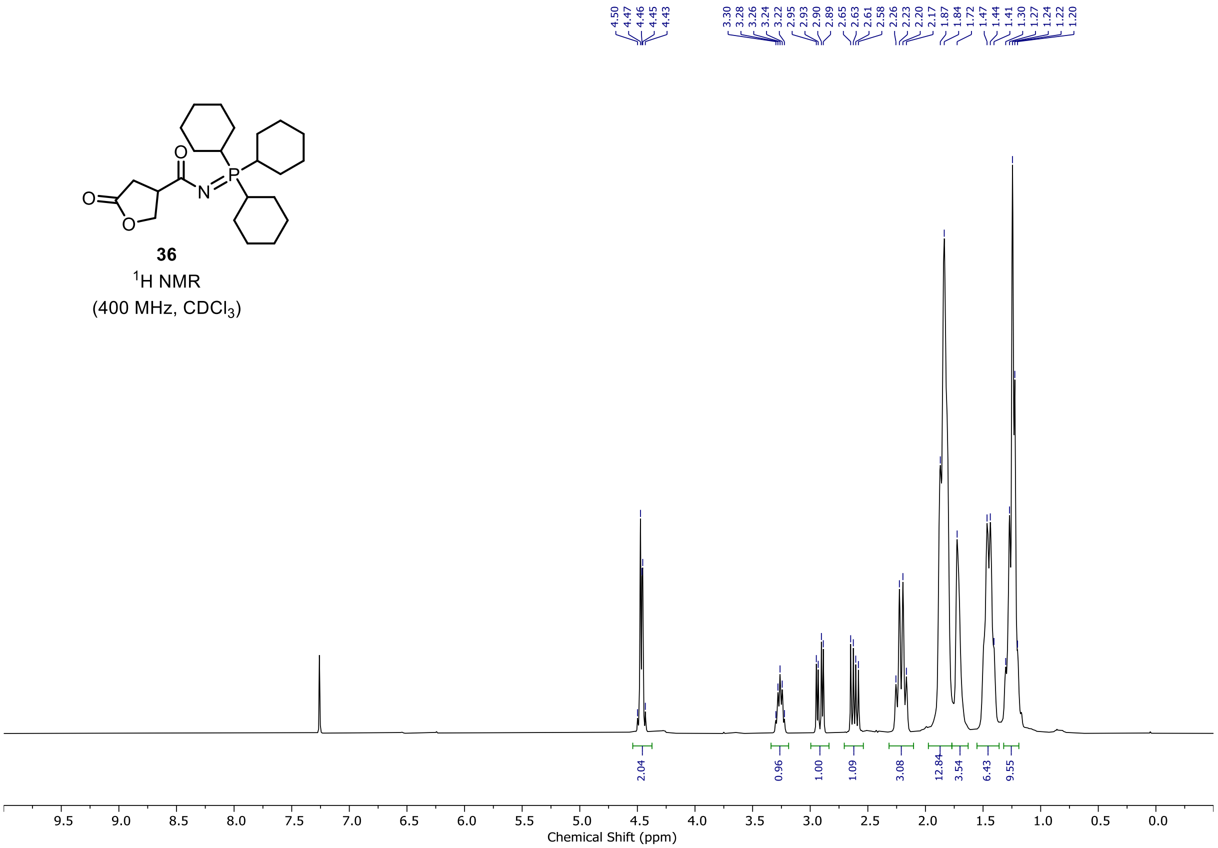
36
^{1}H NMR
(400 MHz, CDCl$_3$)
4.50 4.47 4.46 4.45 4.43
3.30 3.28 3.26 3.24 3.22 2.95 2.93 2.90 2.89 2.65 2.63 2.61 2.58 2.26 2.23 2.20 2.17 1.87 1.84 1.72 1.47 1.44 1.41 1.30 1.27 1.24 1.22 1.20
2.04 0.96 1.00 1.09 3.08 12.84 3.54 6.43 9.55
9.5 9.0 8.5 8.0 7.5 7.0 6.5 6.0 5.5 5.0 4.5 4.0 3.5 3.0 2.5 2.0 1.5 1.0 0.5 0.0
Chemical Shift (ppm)

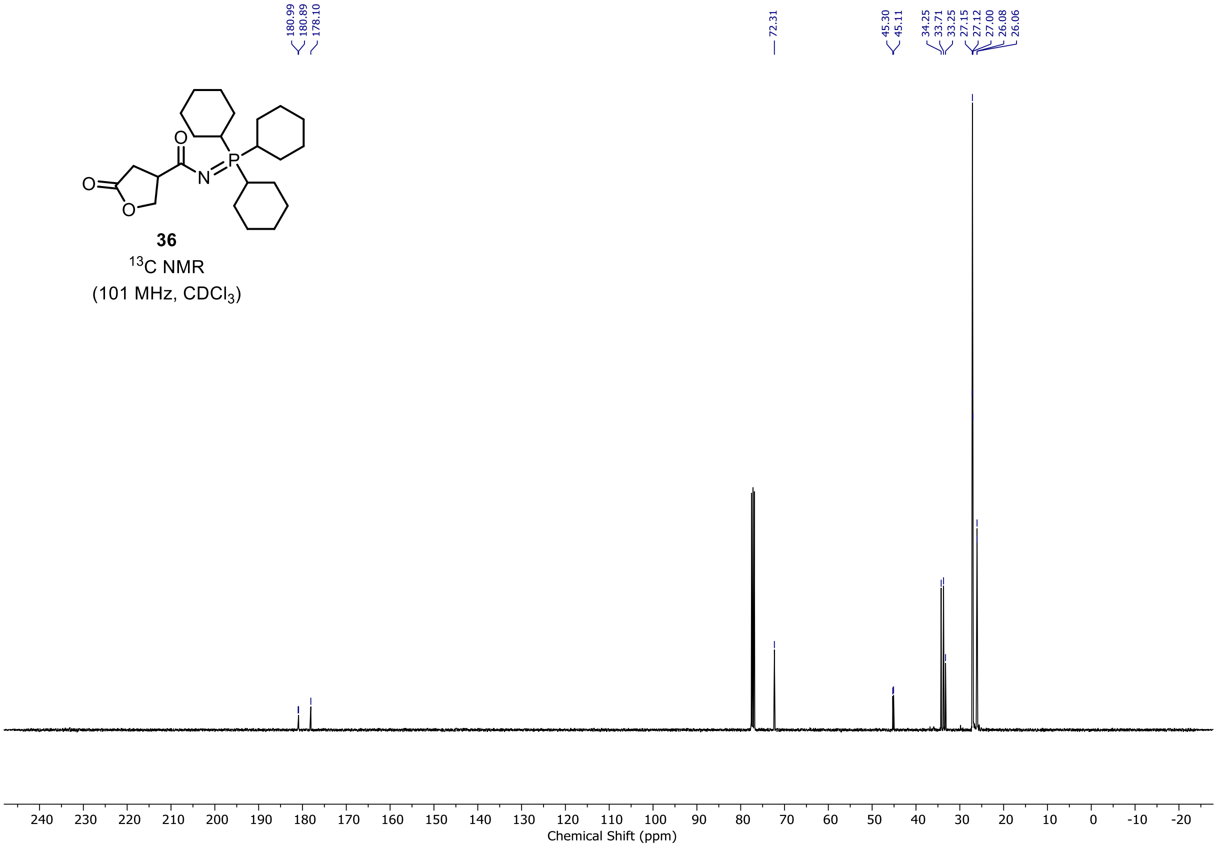
36
^{13}C NMR
(101 MHz, CDCl$_3$)
180.99 180.89 178.10
72.31
45.30 45.11 34.25 33.71 33.25 27.15 27.12 27.00 26.08 26.06
240 230 220 210 200 190 180 170 160 150 140 130 120 110 100 90 80 70 60 50 40 30 20 10 0 -10 -20
Chemical Shift (ppm)

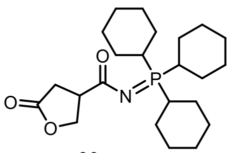

36
^{31}P NMR
(162 MHz, $CDCl_3$)

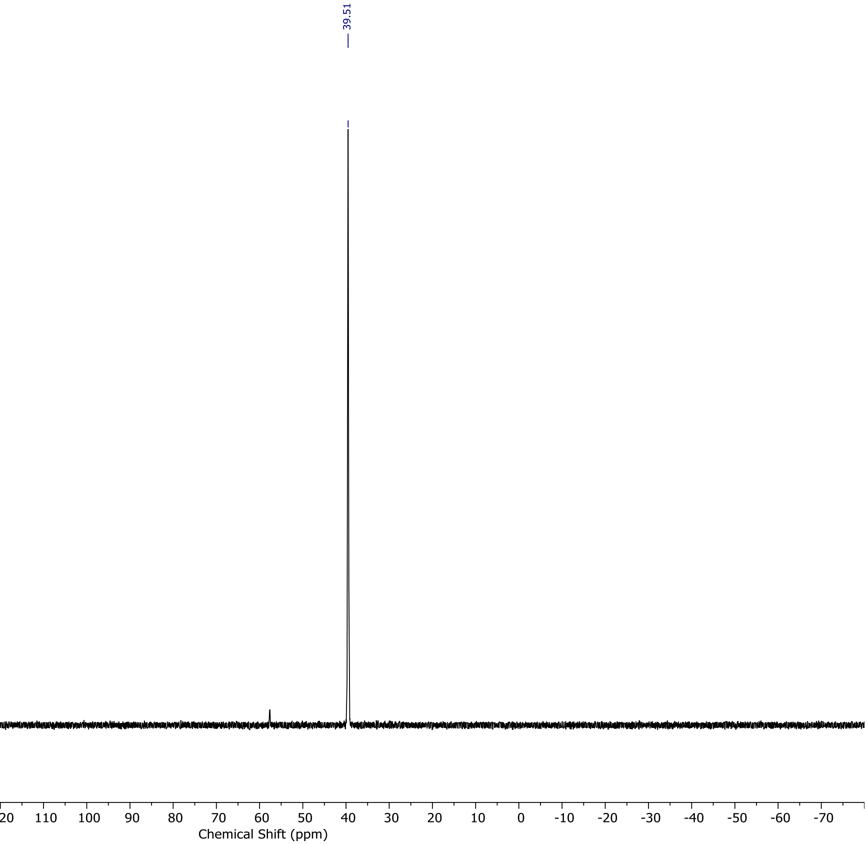

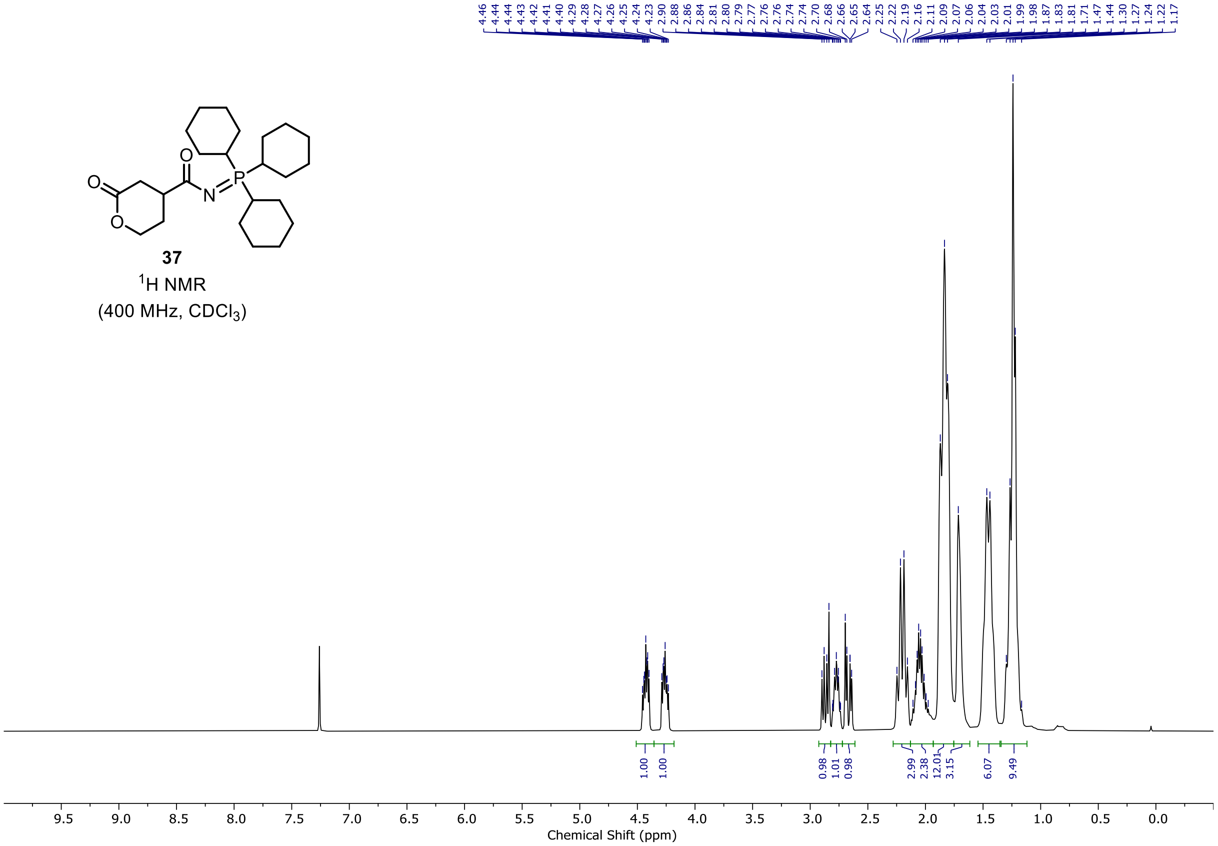

37

^{1}H NMR

(400 MHz, $CDCl_3$)

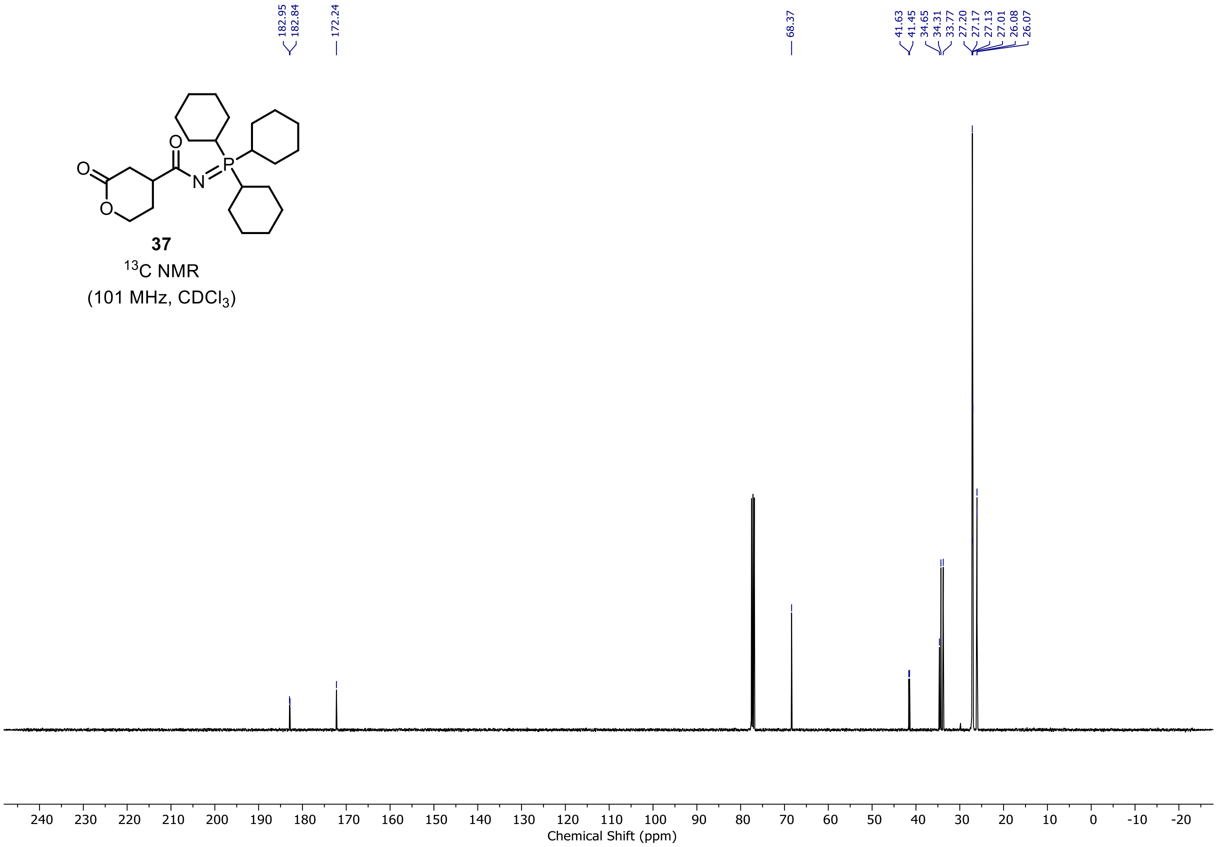

37

^{13}C NMR

(101 MHz, $CDCl_3$)

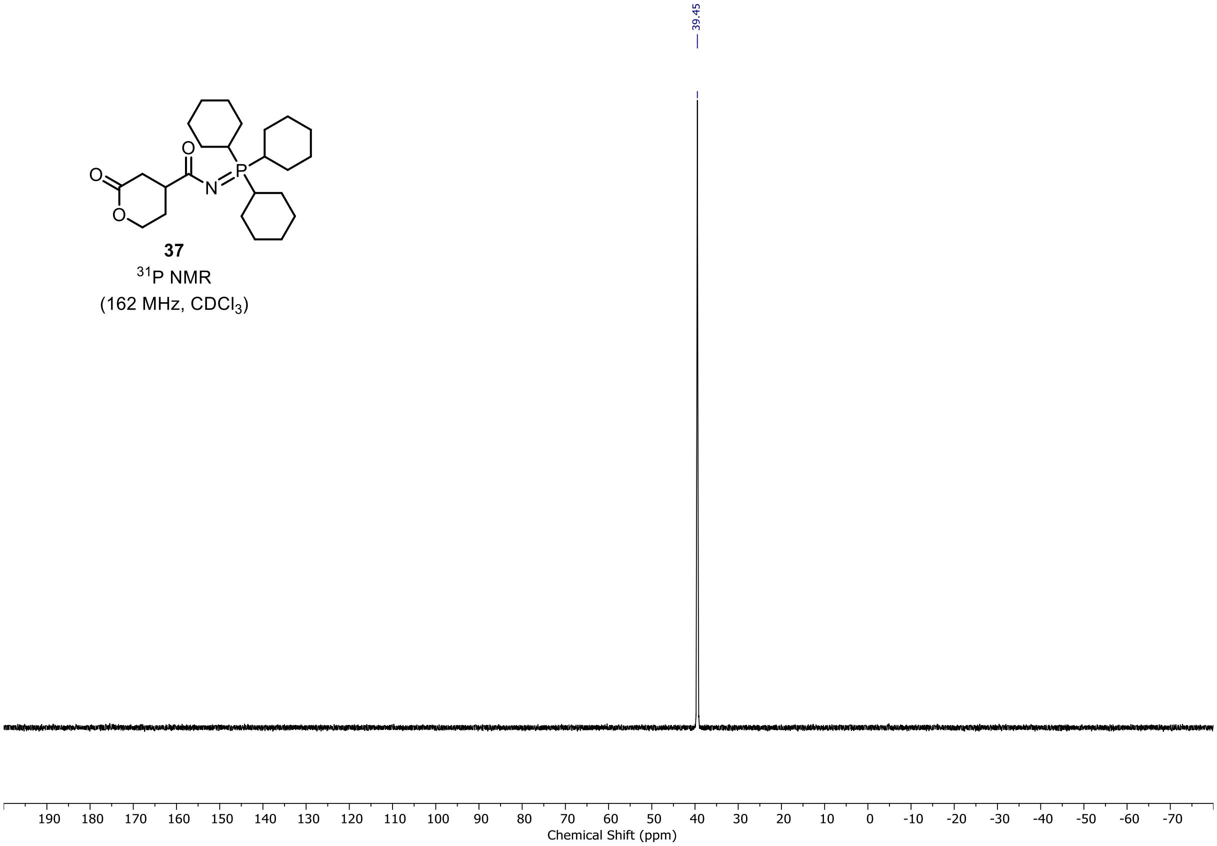

37

^{31}P NMR

(162 MHz, $CDCl_3$)

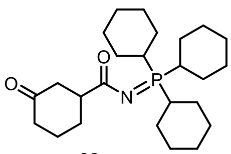

38

^{1}H NMR

(400 MHz, $CDCl_3$)

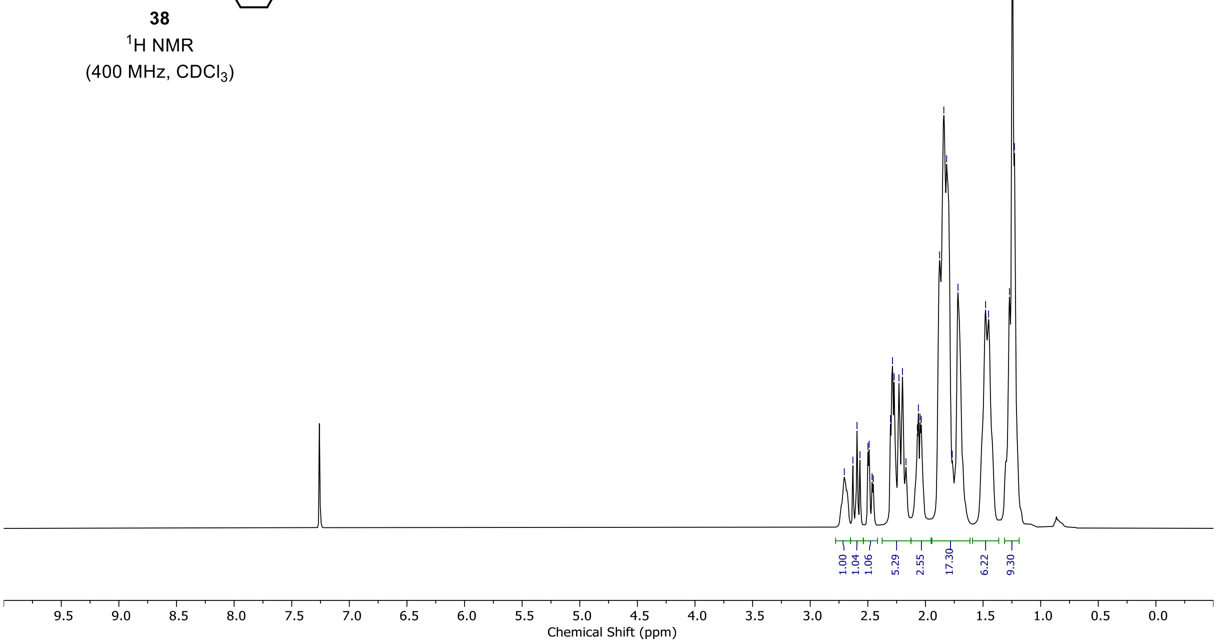

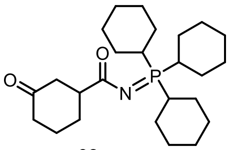

38

^{13}C NMR

(101 MHz, $CDCl_3$)

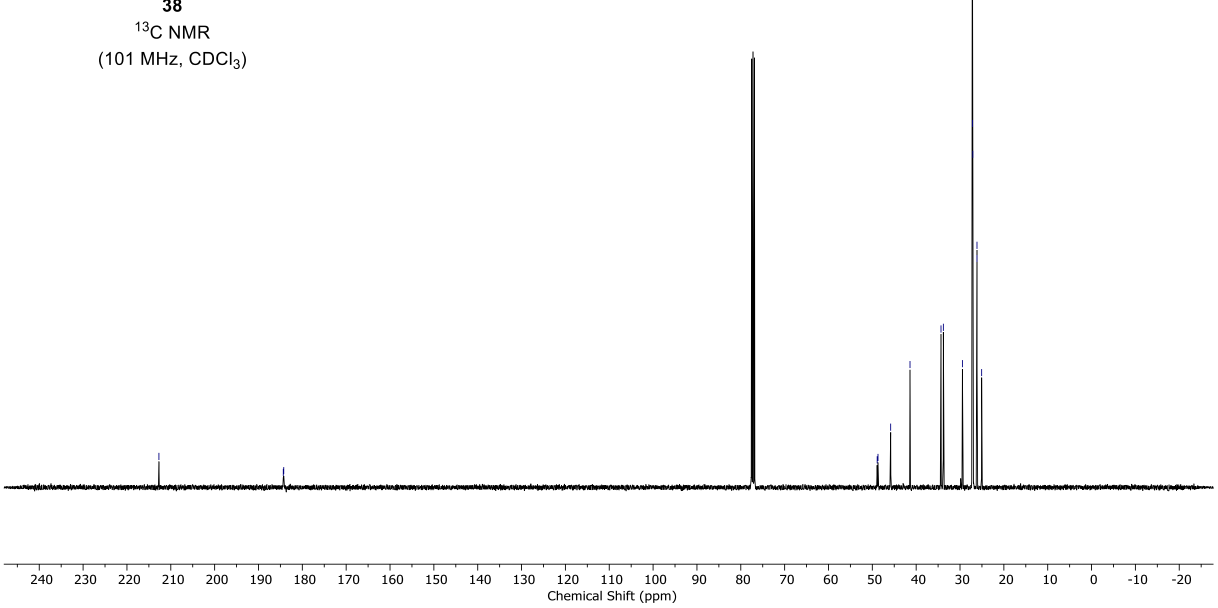

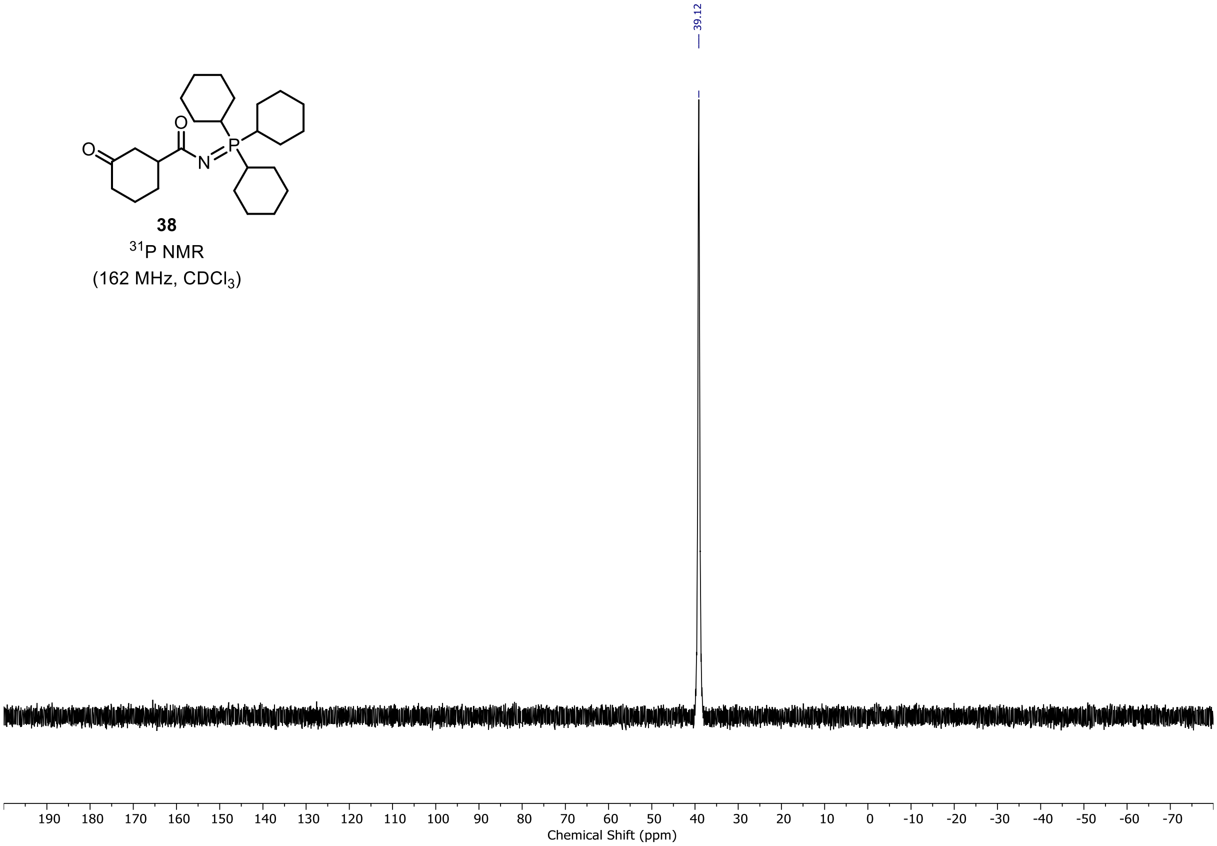

38

^{31}P NMR

(162 MHz, $CDCl_3$)

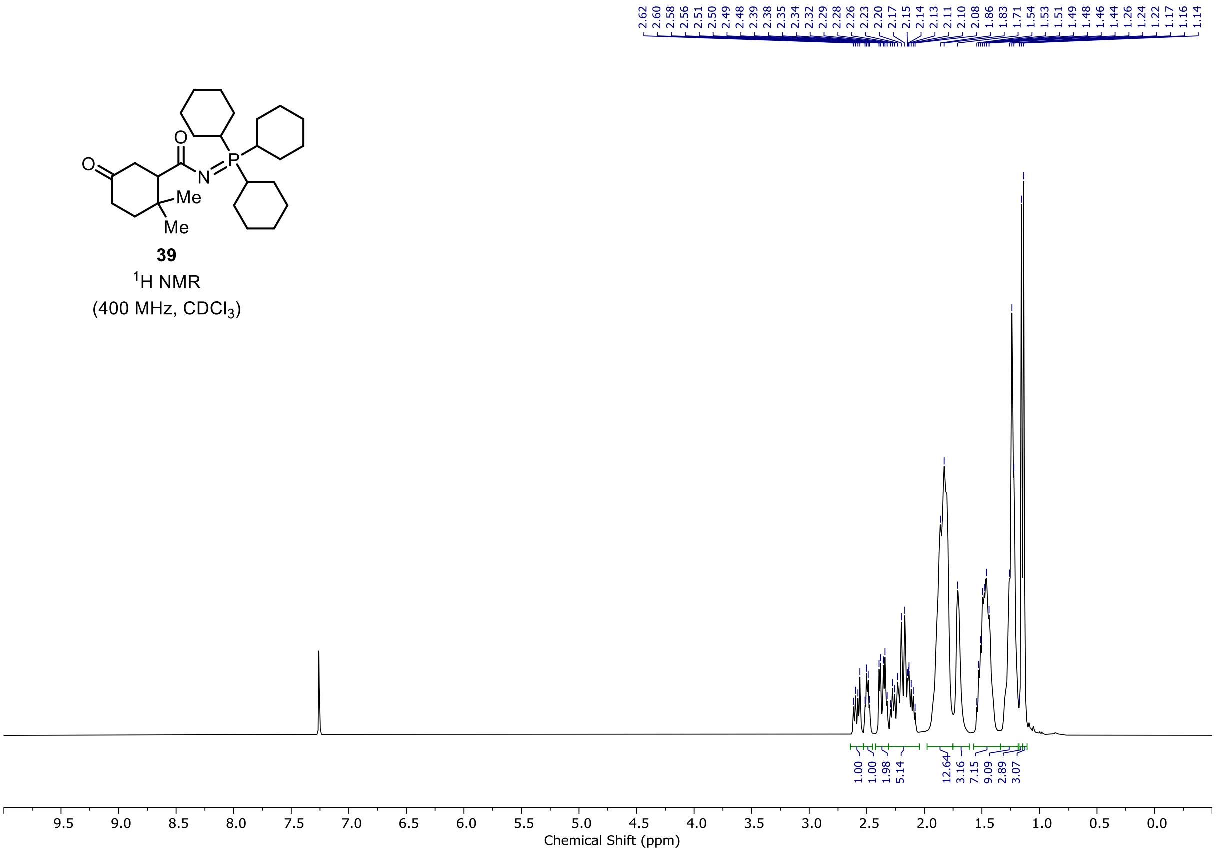
2.62 2.60 2.58 2.56 2.51 2.50 2.49 2.48 2.39 2.38 2.35 2.34 2.32 2.29 2.28 2.26 2.23 2.20 2.17 2.15 2.14 2.13 2.11 2.11 2.10 2.08 1.86 1.83 1.71 1.54 1.53 1.51 1.49 1.48 1.46 1.44 1.26 1.24 1.22 1.17 1.16 1.14
O
N
P
Me
Me
39
^{1}H NMR
(400 MHz, $CDCl_3$)
1.00 1.00 1.98 5.14 12.64 3.16 7.15 9.09 2.89 3.07
9.5 9.0 8.5 8.0 7.5 7.0 6.5 6.0 5.5 5.0 4.5 4.0 3.5 3.0 2.5 2.0 1.5 1.0 0.5 0.0
Chemical Shift (ppm)

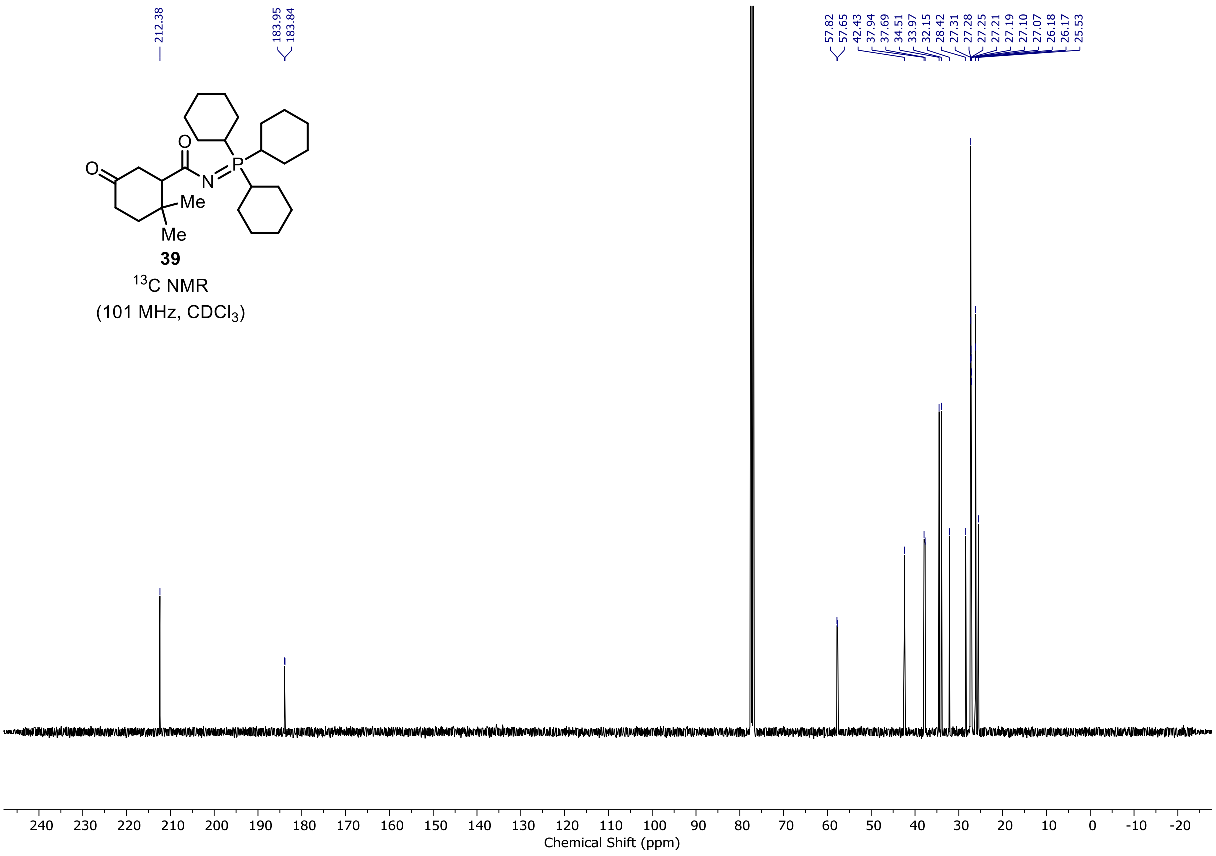
212.38
183.95 183.84
57.82 57.65 42.43 37.94 37.69 34.51 33.97 32.15 28.42 27.31 27.28 27.25 27.21 27.19 27.10 27.07 26.18 26.17 25.53
O
N
P
Me
Me
39
^{13}C NMR
(101 MHz, $CDCl_3$)
240 230 220 210 200 190 180 170 160 150 140 130 120 110 100 90 80 70 60 50 40 30 20 10 0 -10 -20
Chemical Shift (ppm)

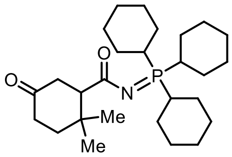

39

^{31}P NMR

(162 MHz, $CDCl_3$)

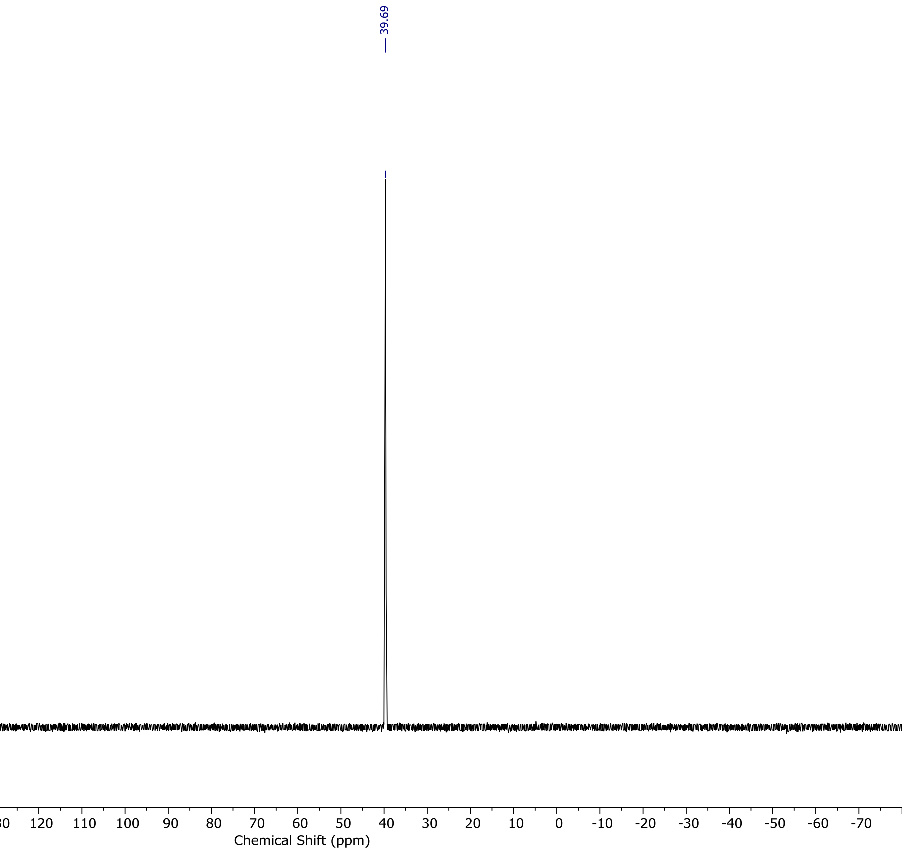

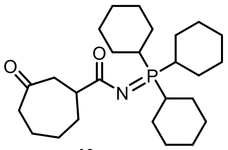

40

^{1}H NMR

(400 MHz, $CDCl_3$)

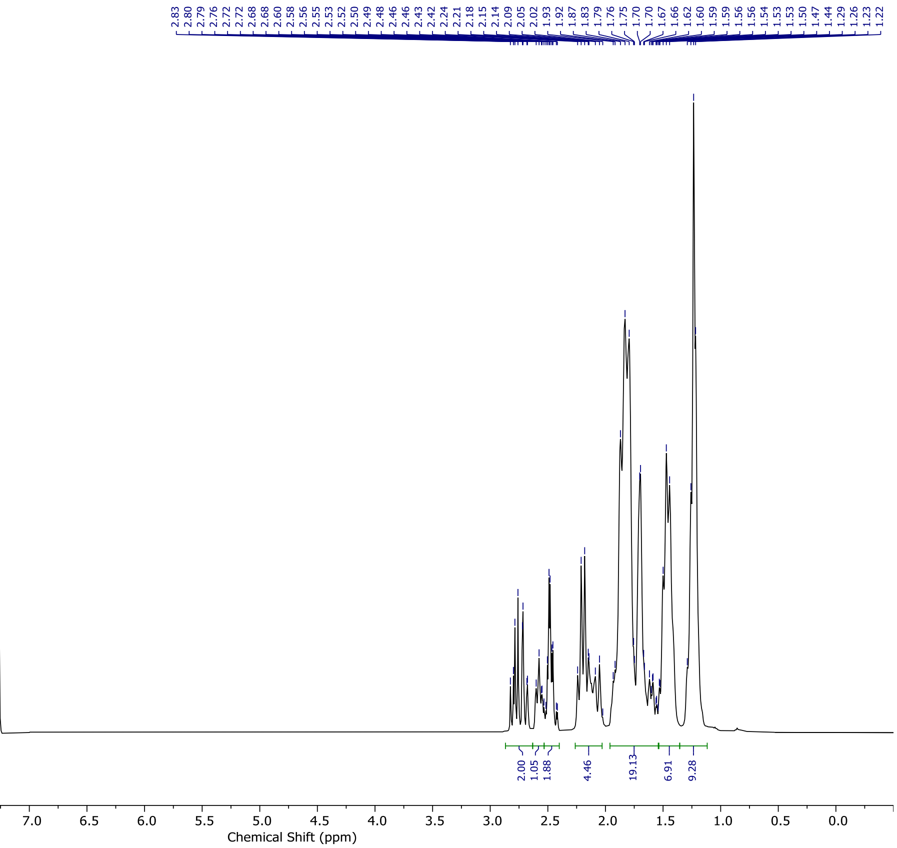

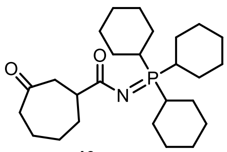

40

^{13}C NMR

(101 MHz, $CDCl_3$)

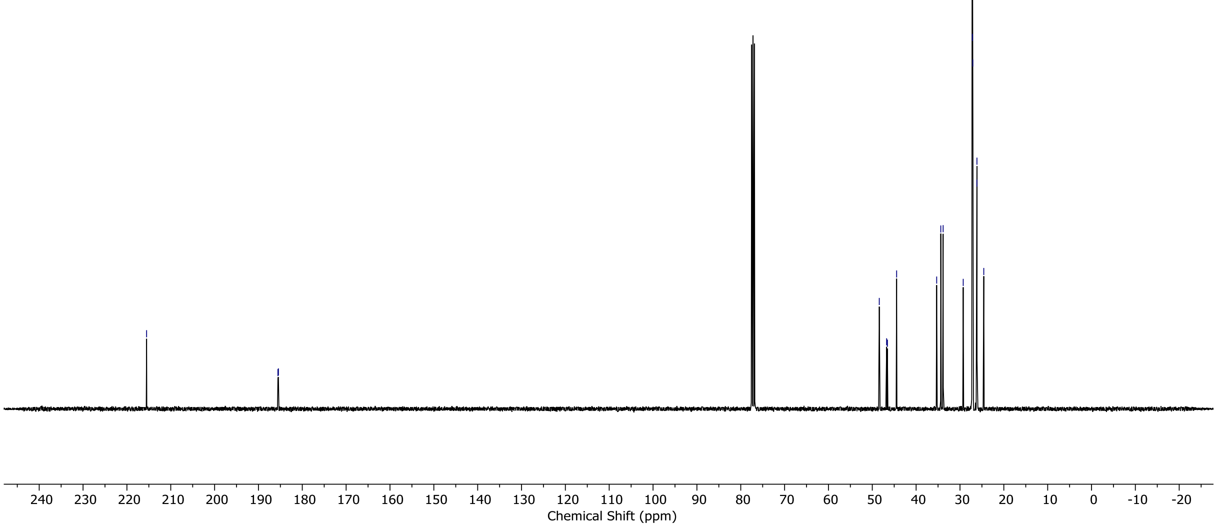

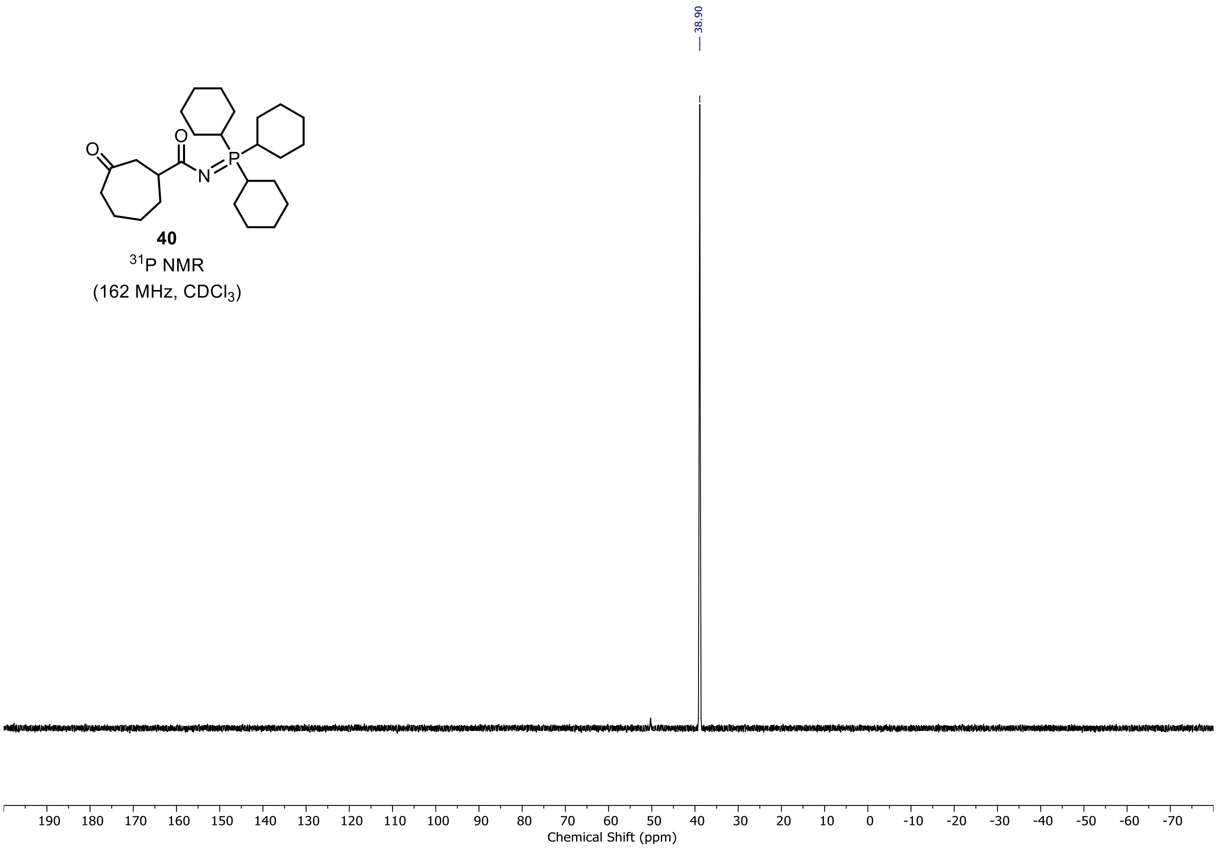

40

^{31}P NMR

(162 MHz, $CDCl_3$)

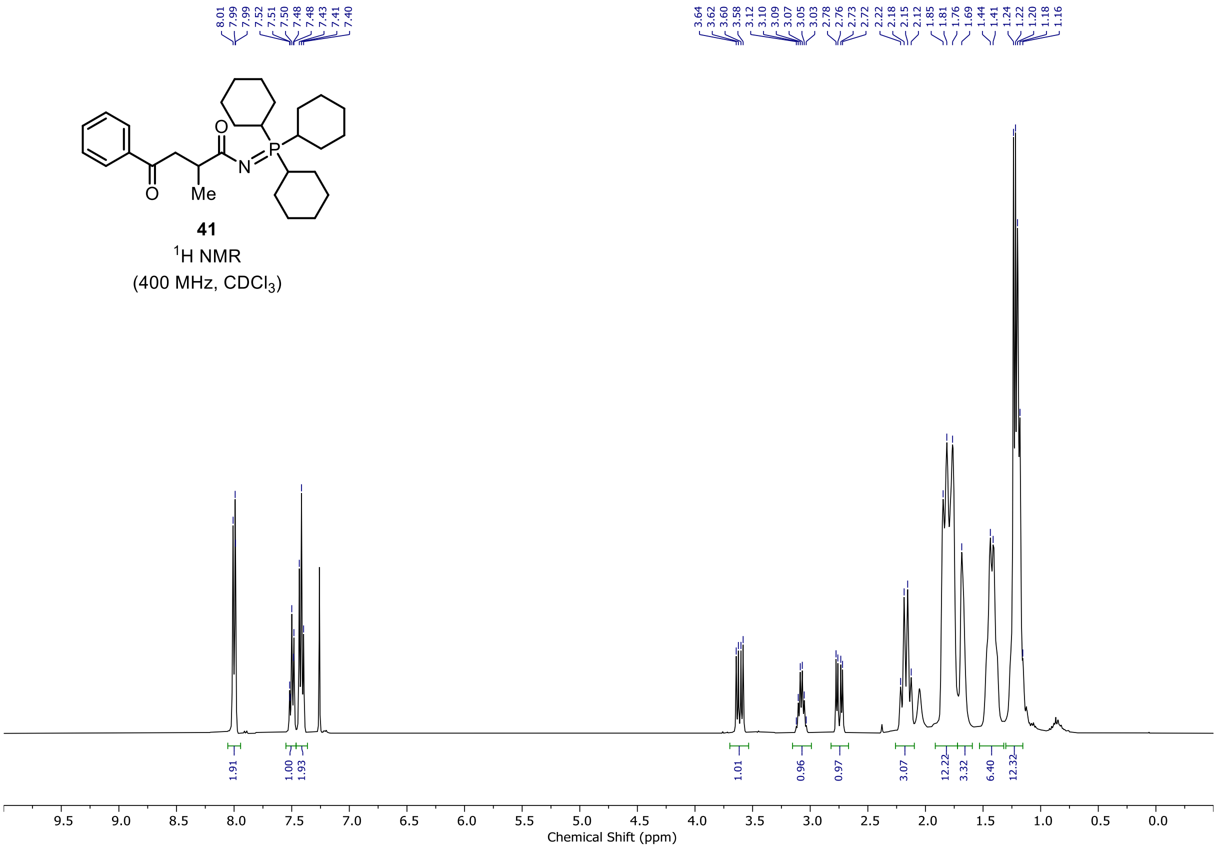

41

^{1}H NMR

(400 MHz, $CDCl_3$)

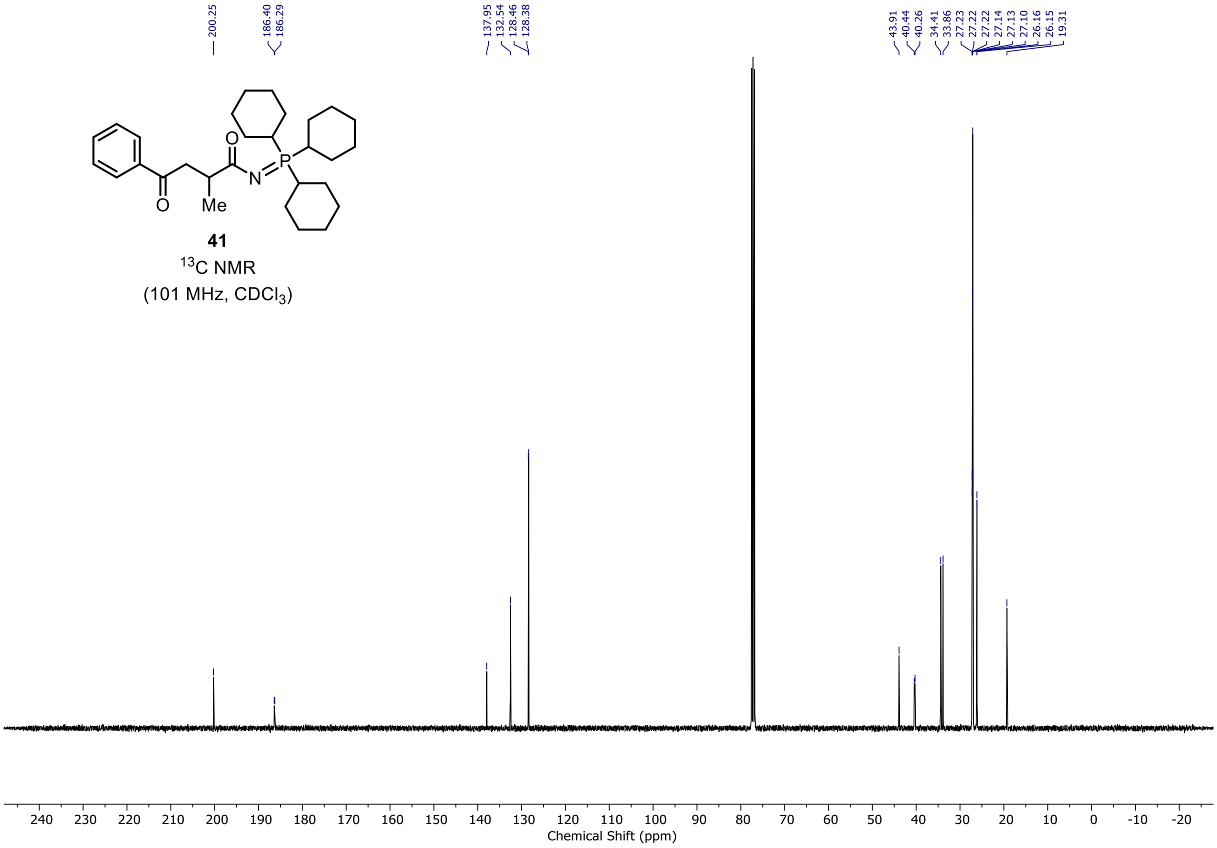

41

^{13}C NMR

(101 MHz, $CDCl_3$)

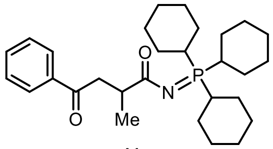

41
^{31}P NMR
(162 MHz, $CDCl_3$)

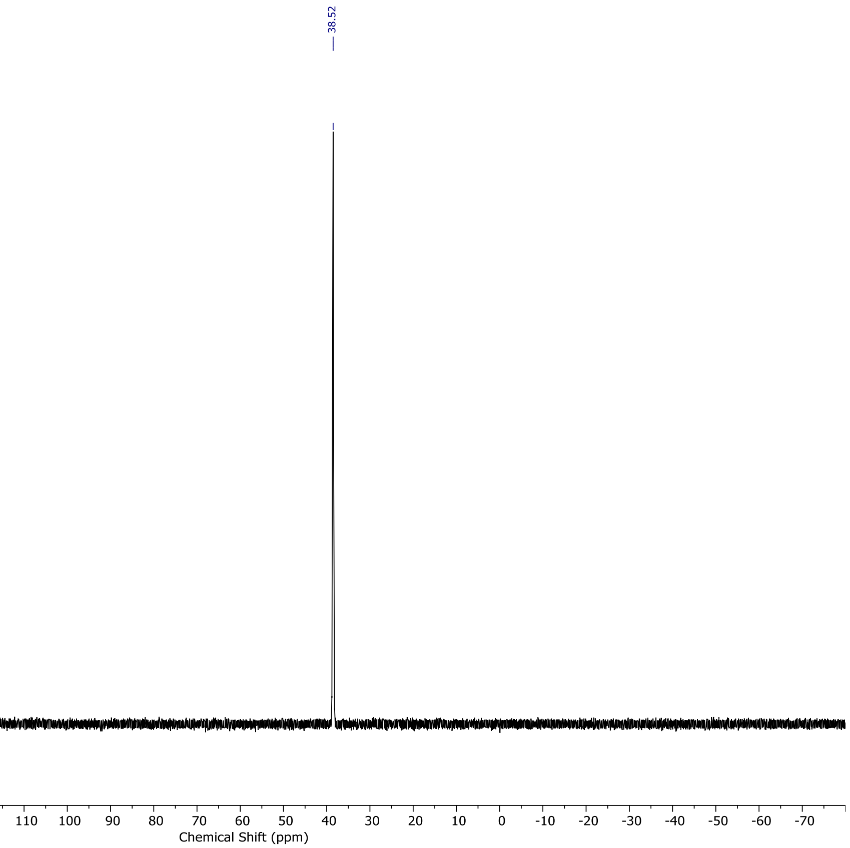

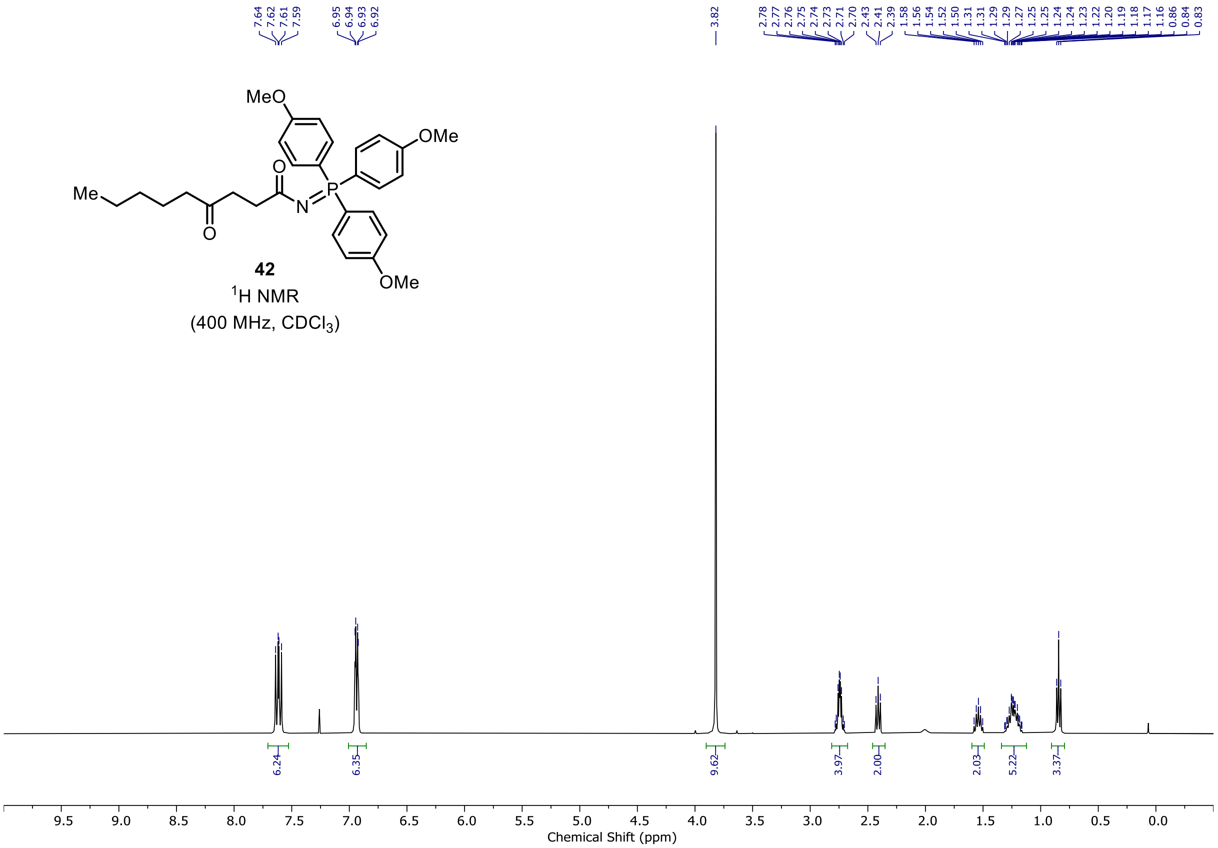

MeO
OMe
Me
O
N
P
O
OMe
42
^{1}H NMR
(400 MHz, CDCl$_3$)
Chemical Shift (ppm)

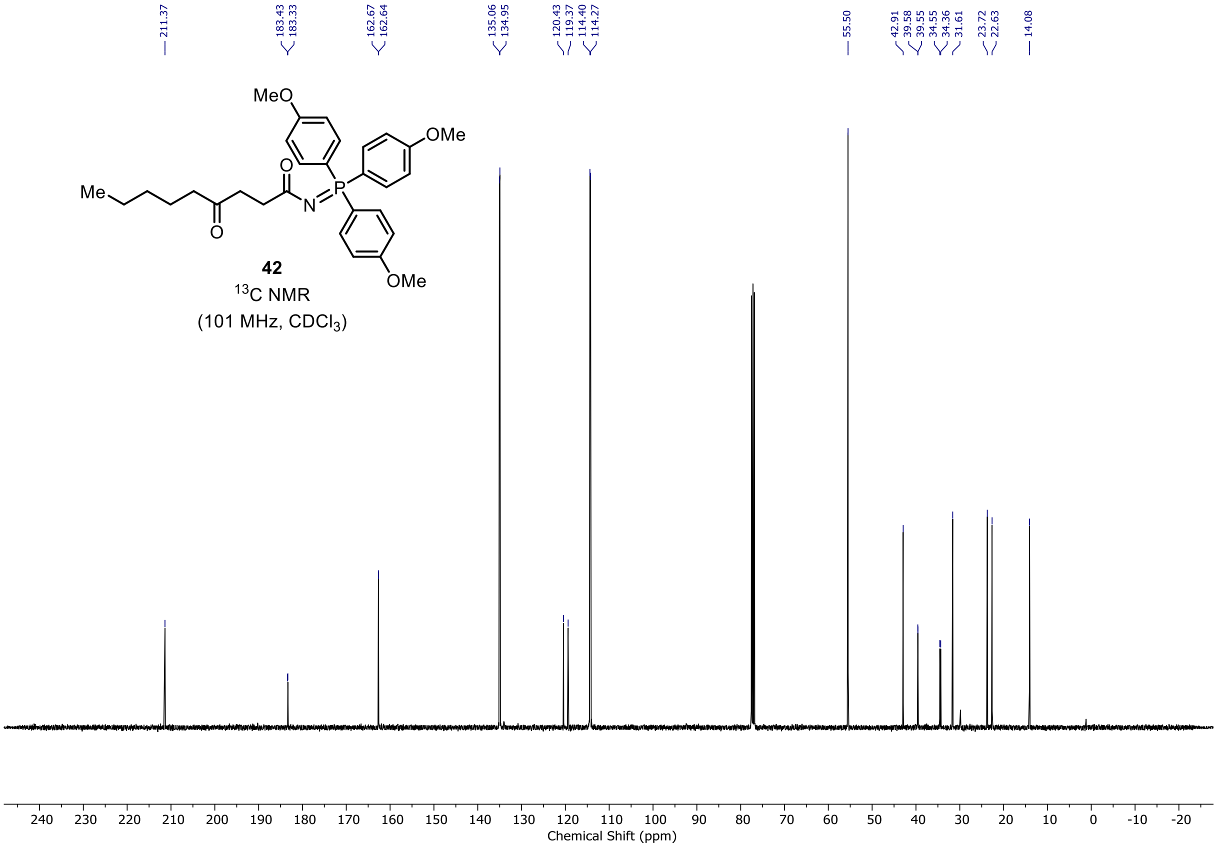

MeO
OMe
Me
O
N
P
O
OMe
42
^{13}C NMR
(101 MHz, CDCl$_3$)
Chemical Shift (ppm)

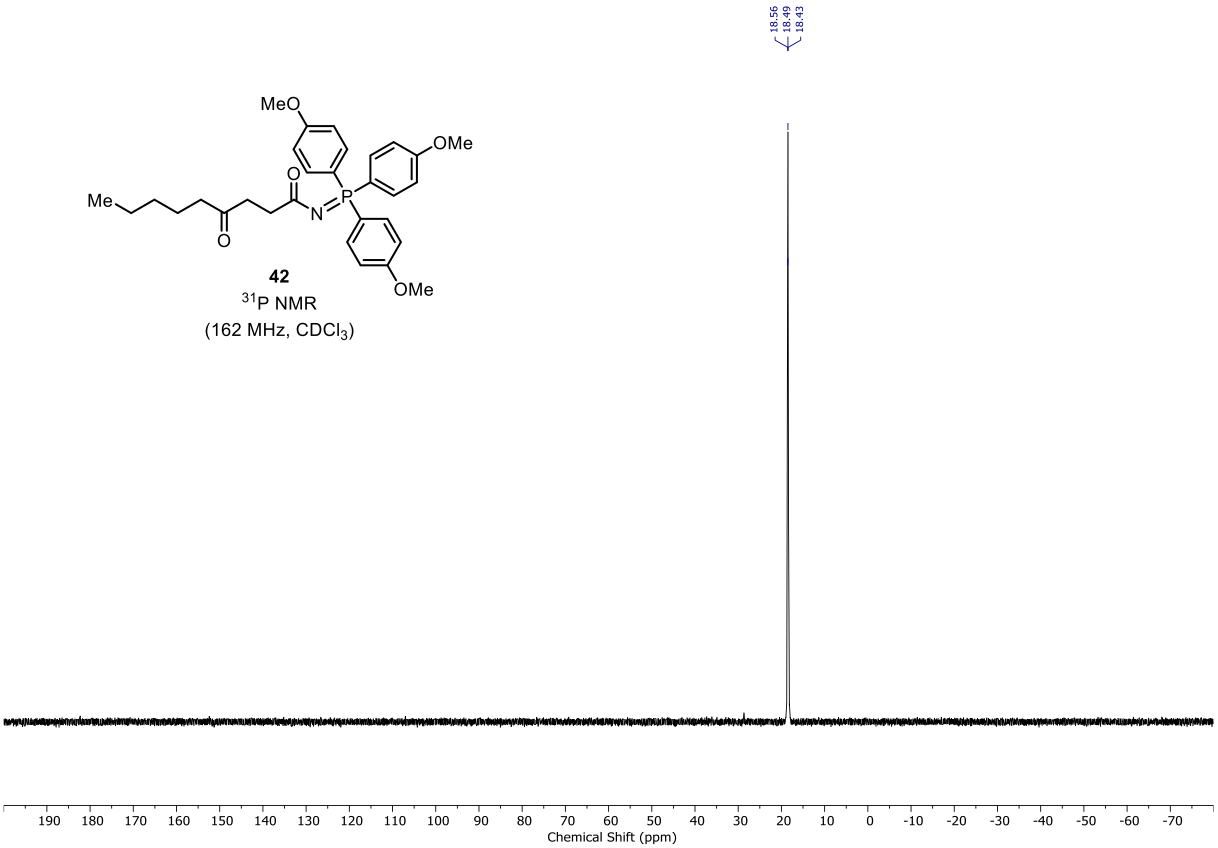

18.56
18.49
18.43
MeO
OMe
Me
O
N
P
O
OMe
42
^{31}P NMR
(162 MHz, CDCl$_3$)
190 180 170 160 150 140 130 120 110 100 90 80 70 60 50 40 30 20 10 0 -10 -20 -30 -40 -50 -60 -70
Chemical Shift (ppm)

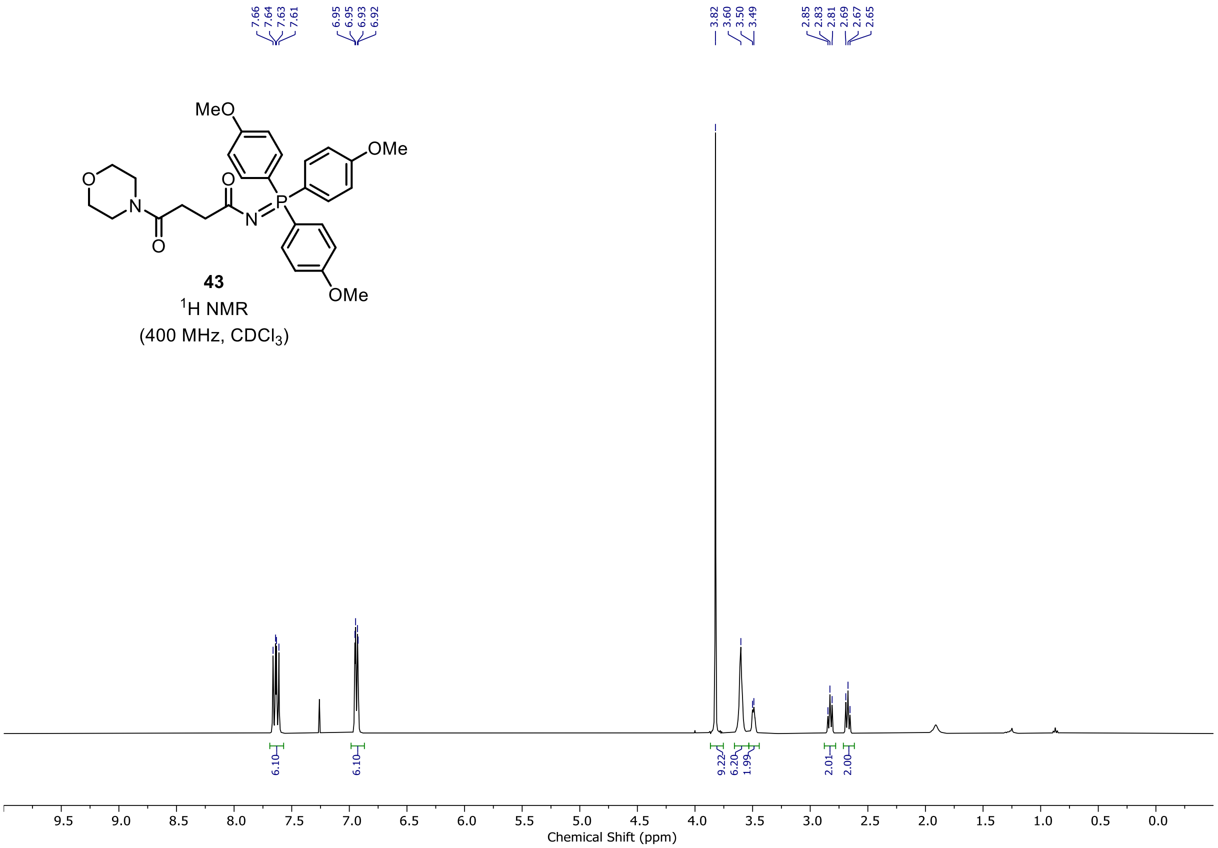
43
^{1}H NMR
(400 MHz, CDCl$_3$)
Chemical Shift (ppm)

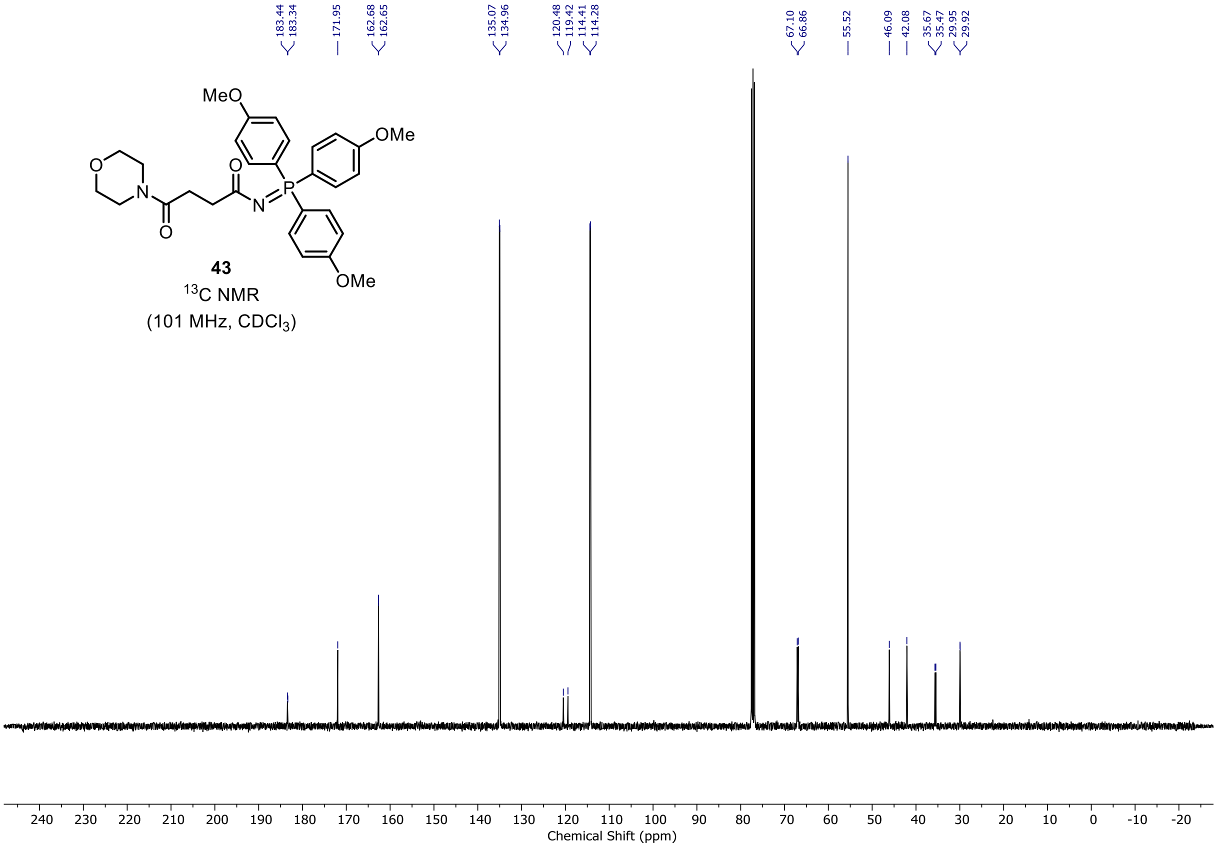
43
^{13}C NMR
(101 MHz, CDCl$_3$)
Chemical Shift (ppm)

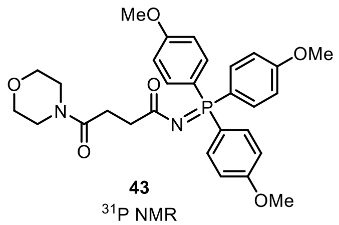

43
^{31}P NMR
(162 MHz, $CDCl_3$)

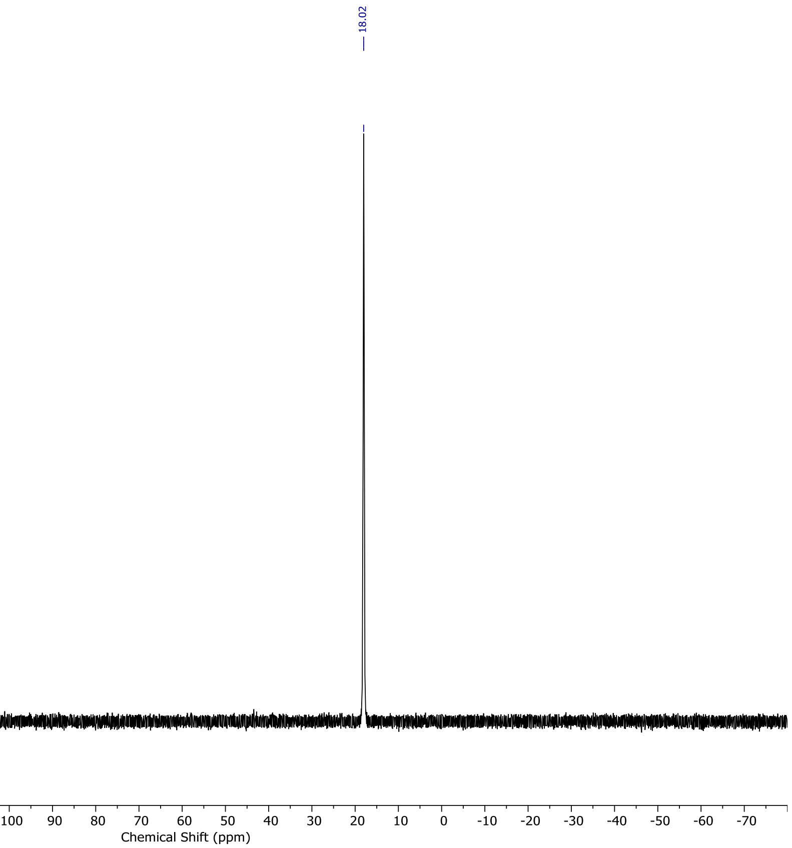

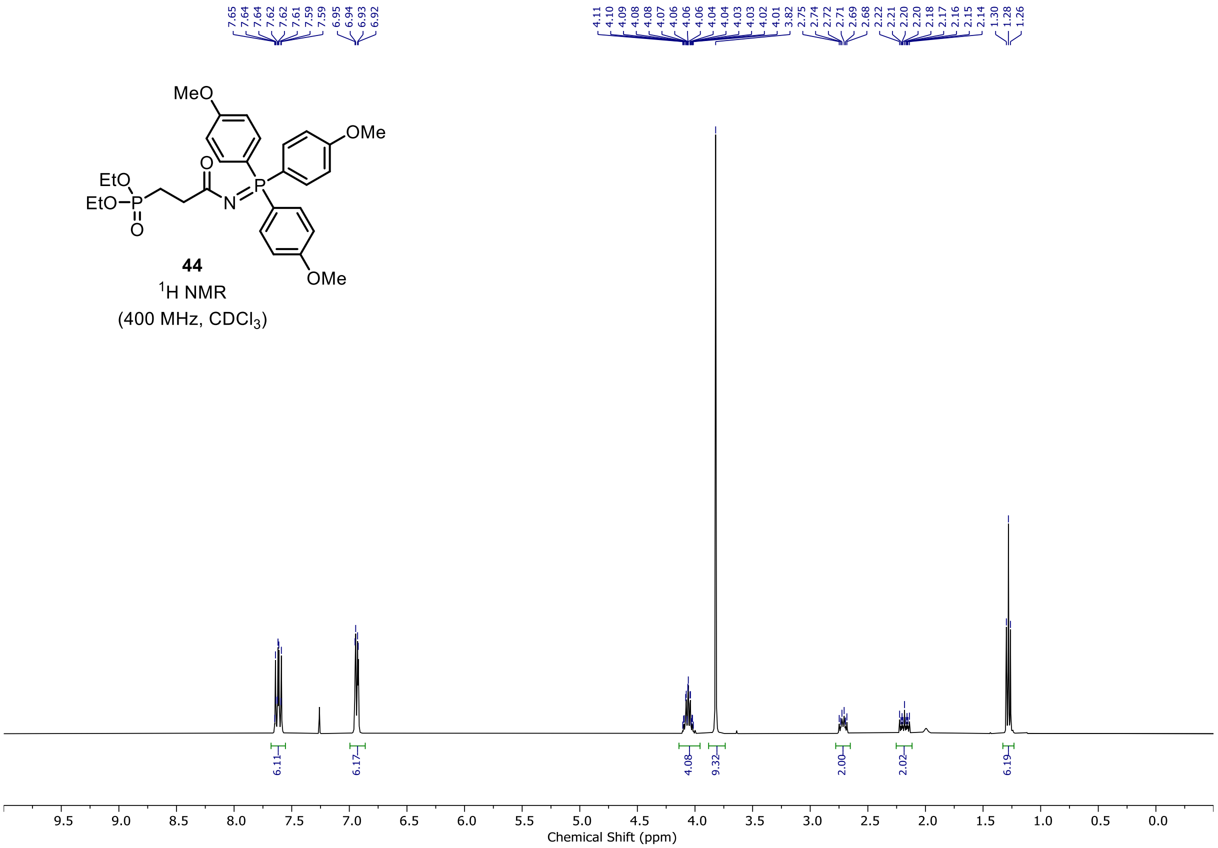
MeO
OMe
EtO
EtO
P
O
O
N
P
OMe
44
^{1}H NMR
(400 MHz, CDCl$_3$)
7.65
7.64
7.64
7.62
7.62
7.61
7.59
7.59
6.95
6.94
6.93
6.92
4.11
4.10
4.09
4.08
4.08
4.07
4.06
4.06
4.06
4.04
4.04
4.03
4.03
4.02
4.01
3.82
2.75
2.74
2.72
2.71
2.69
2.68
2.22
2.21
2.20
2.20
2.18
2.17
2.16
2.15
2.14
1.30
1.28
1.26
6.11
6.17
4.08
9.32
2.00
2.02
6.19
Chemical Shift (ppm)

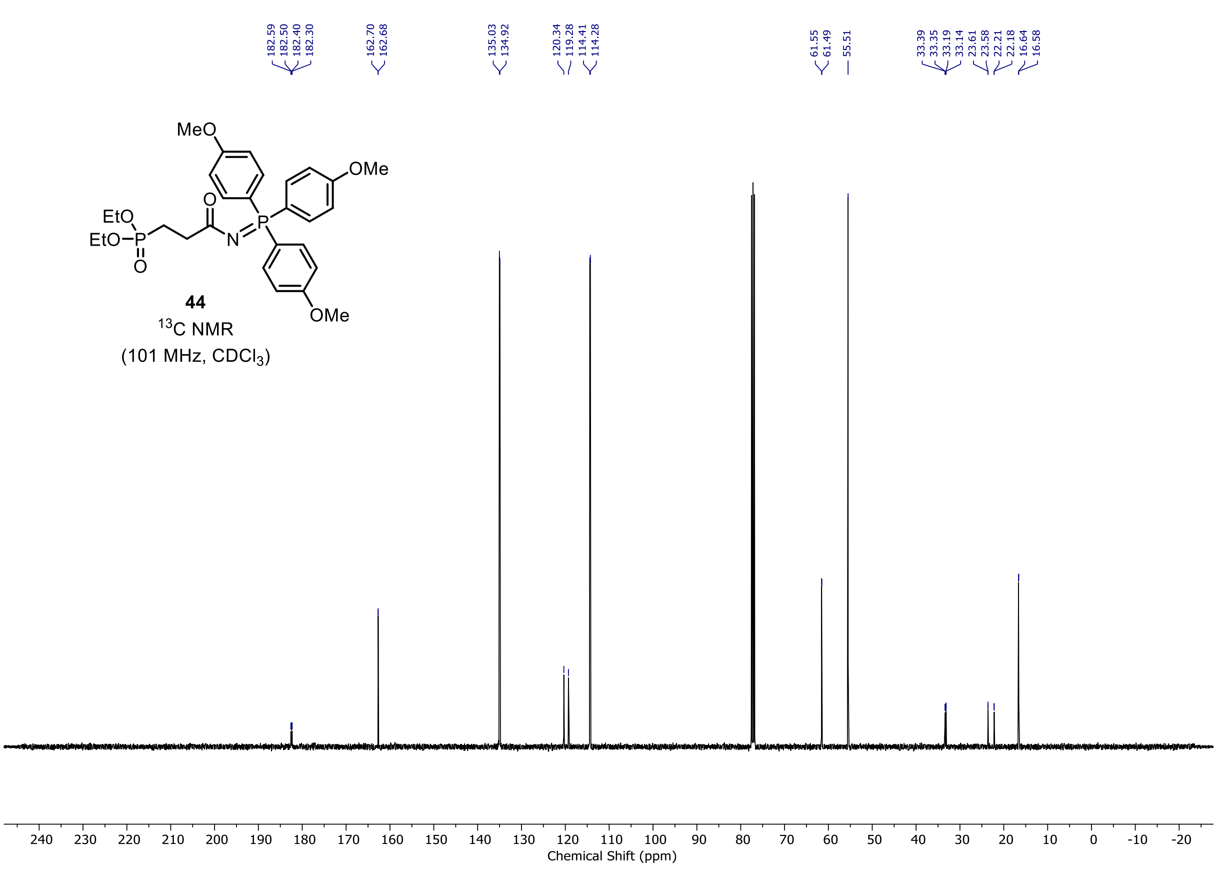
MeO
OMe
EtO
EtO
P
O
O
N
P
OMe
44
^{13}C NMR
(101 MHz, CDCl$_3$)
182.59
182.50
182.40
182.30
162.70
162.68
135.03
134.92
120.34
119.28
114.41
114.28
61.55
61.49
55.51
33.39
33.35
33.19
33.14
23.61
23.58
22.21
22.18
16.64
16.58
Chemical Shift (ppm)

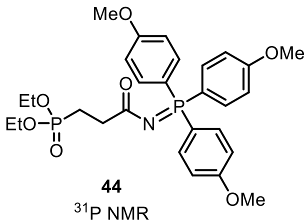

44

^{31}P NMR

(162 MHz, $CDCl_3$)

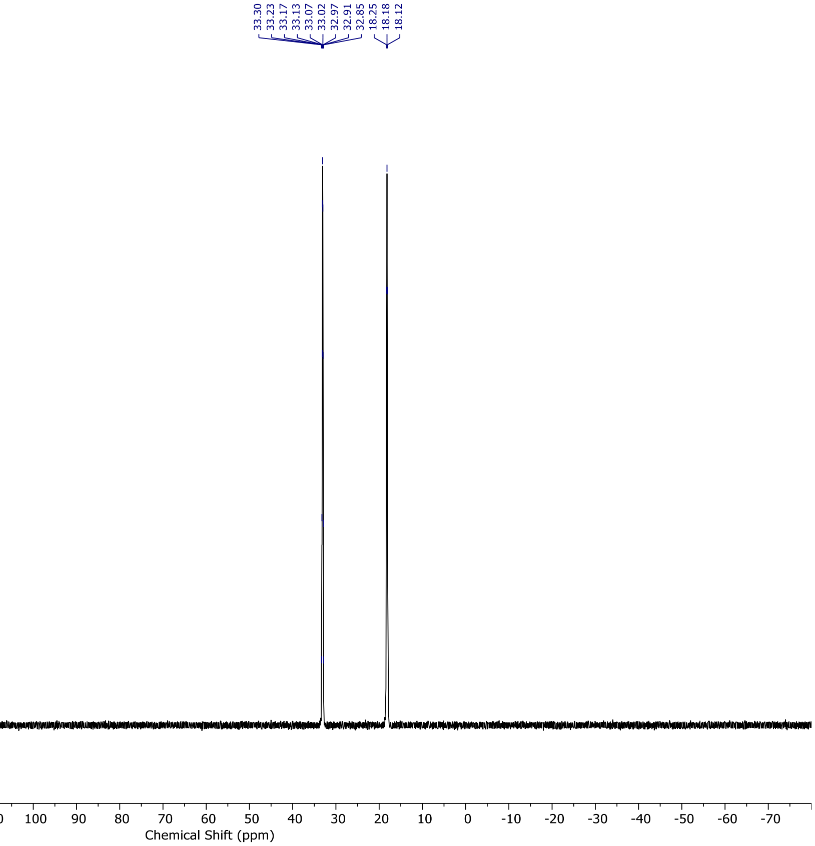

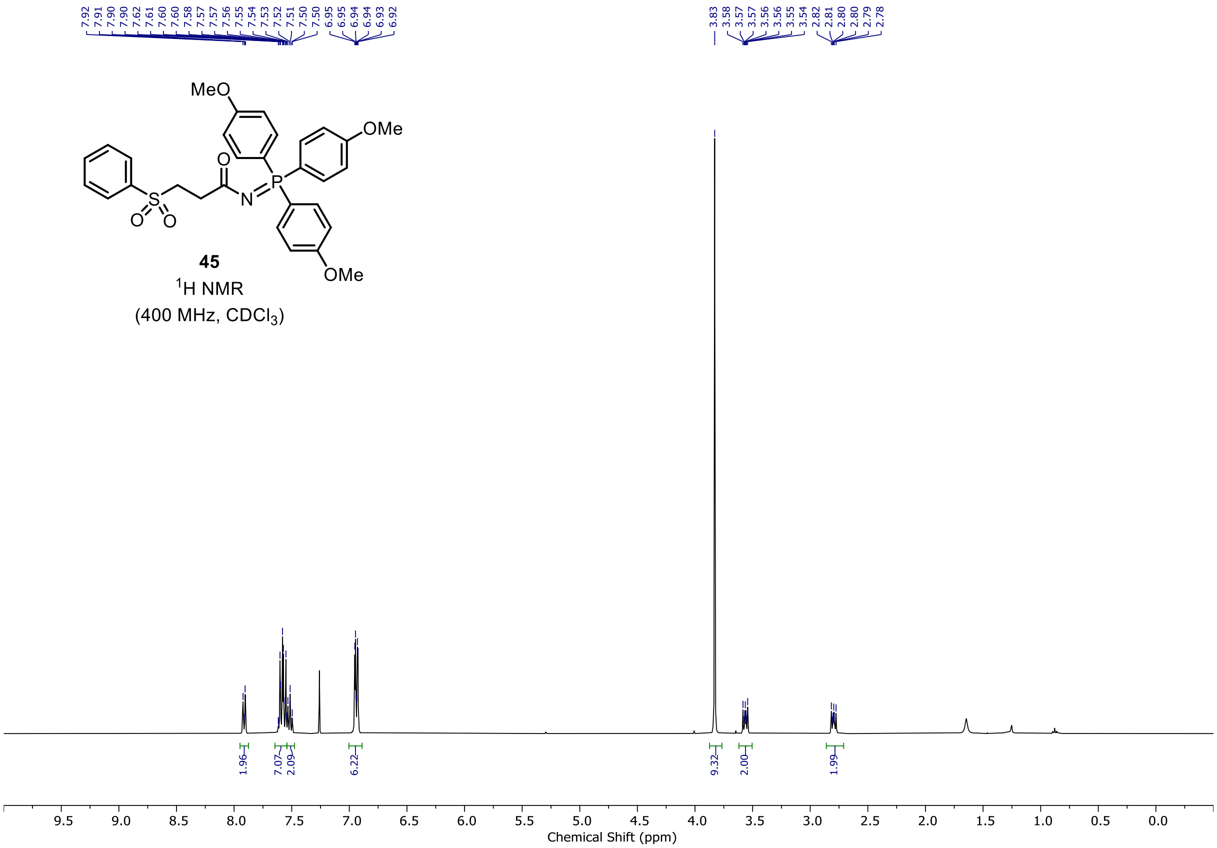
7.92 7.91 7.90 7.90 7.62 7.61 7.60 7.58 7.57 7.57 7.56 7.55 7.54 7.53 7.52 7.51 7.50 7.50 6.95 6.95 6.94 6.94 6.93 6.92
3.83 3.58 3.57 3.57 3.56 3.56 3.55 3.54 2.82 2.81 2.80 2.80 2.79 2.78
MeO
OMe
O
S
N
P
O O
OMe
45
^{1}H NMR
(400 MHz, $CDCl_3$)
1.96
7.07
2.09
6.22
9.32
2.00
1.99
9.5 9.0 8.5 8.0 7.5 7.0 6.5 6.0 5.5 5.0 4.5 4.0 3.5 3.0 2.5 2.0 1.5 1.0 0.5 0.0
Chemical Shift (ppm)

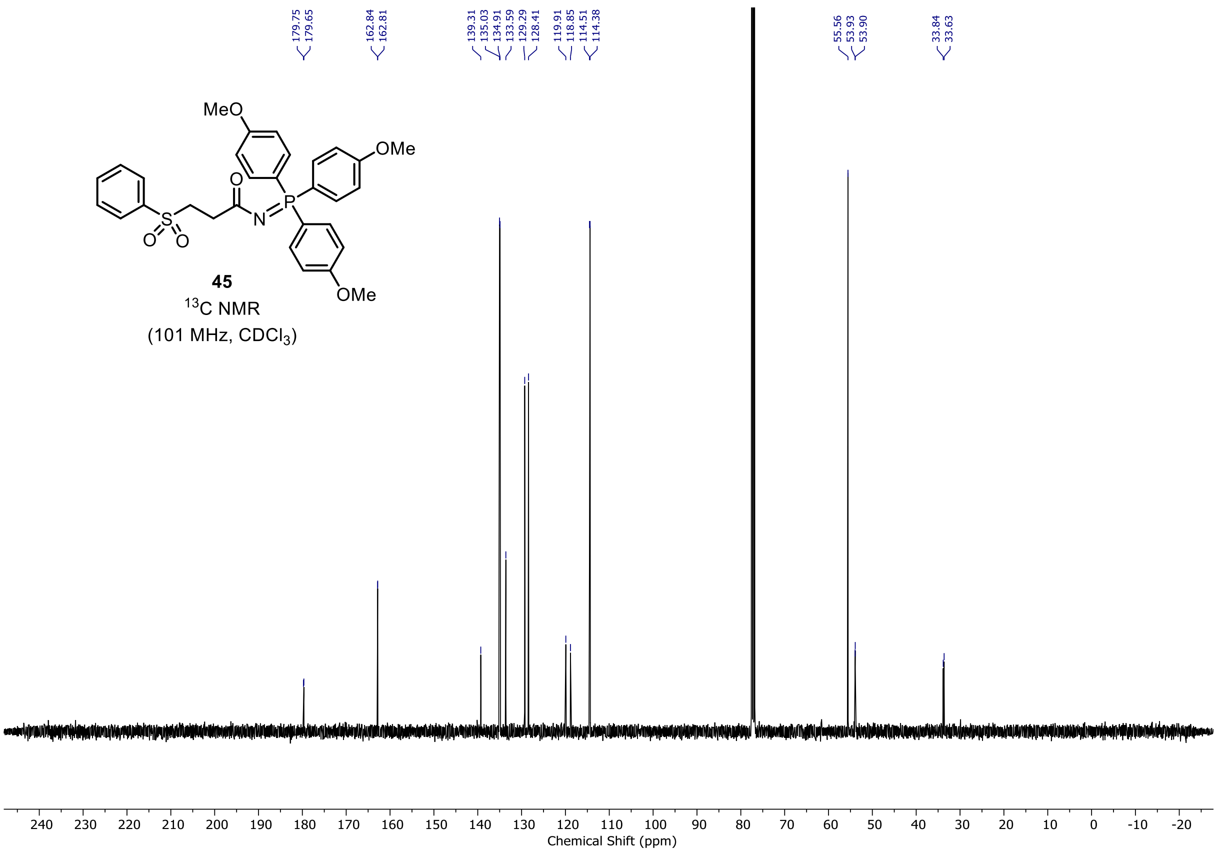
179.75 179.65
162.84 162.81
139.31 135.03 134.91 133.59 129.29 128.41
119.91 118.85 114.51 114.38
55.56 53.93 53.90
33.84 33.63
MeO
OMe
O
S
N
P
O O
OMe
45
^{13}C NMR
(101 MHz, $CDCl_3$)
240 230 220 210 200 190 180 170 160 150 140 130 120 110 100 90 80 70 60 50 40 30 20 10 0 -10 -20
Chemical Shift (ppm)

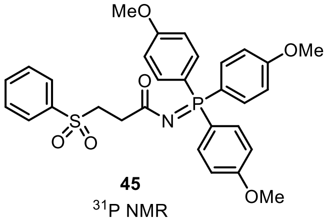

45

^{31}P NMR

(162 MHz, $CDCl_3$)

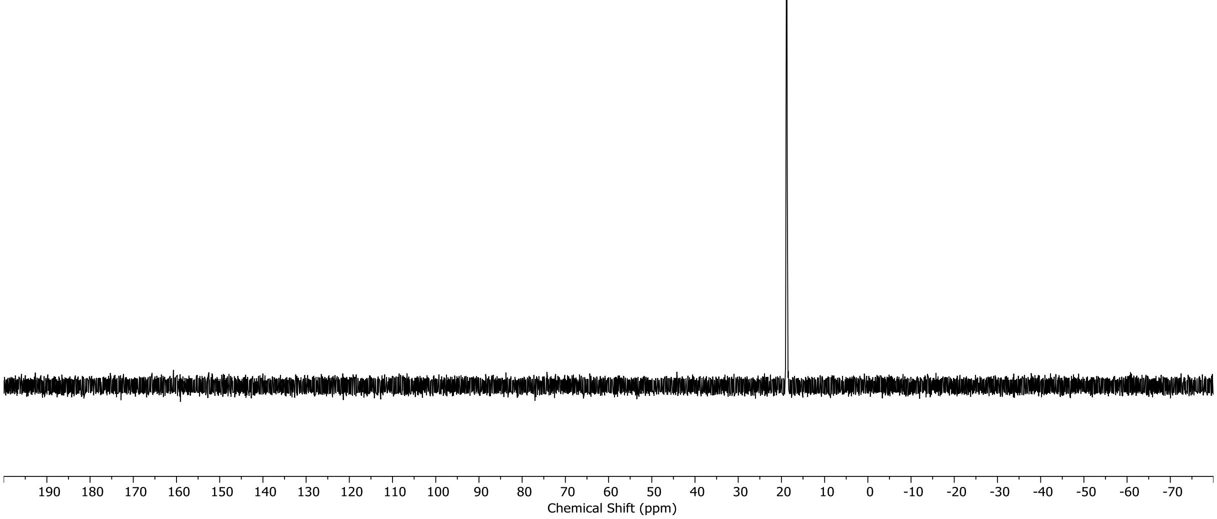

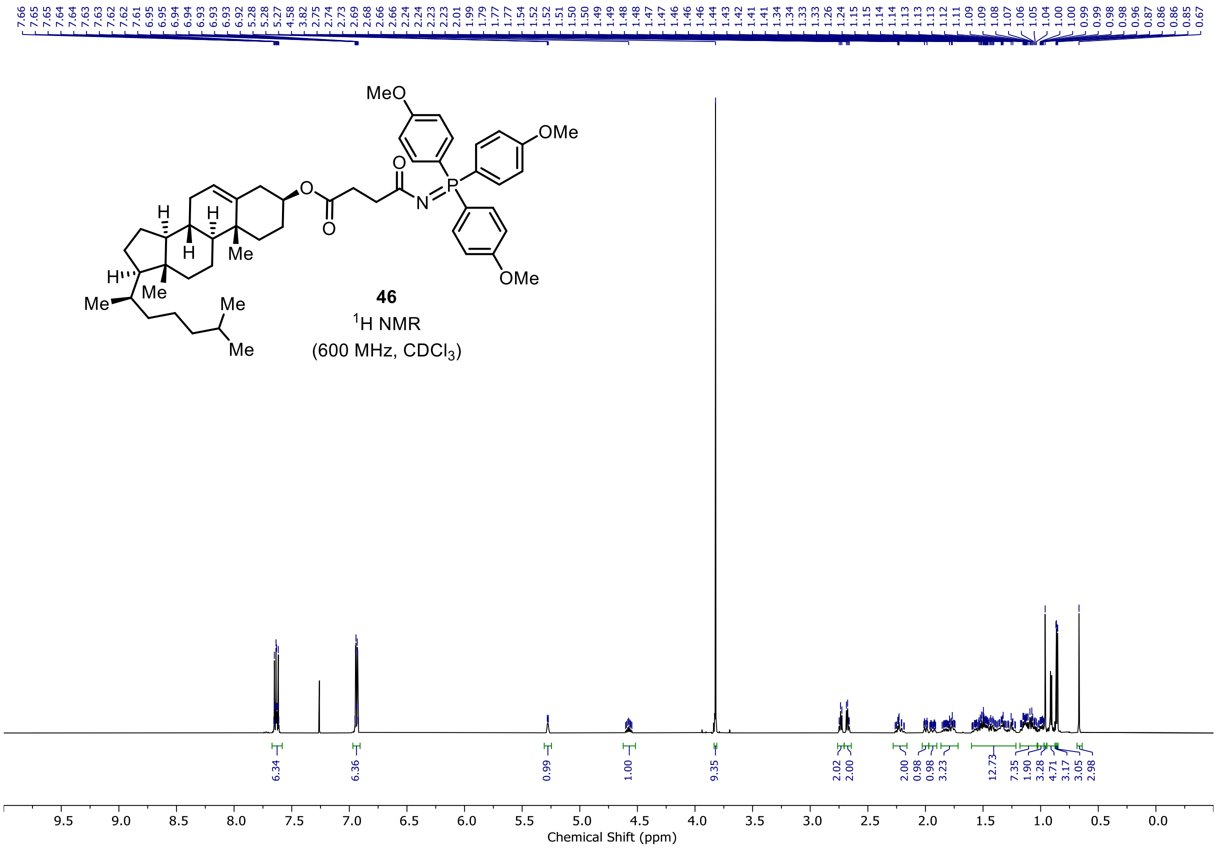
46
^{1}H NMR
(600 MHz, CDCl$_3$)
Chemical Shift (ppm)

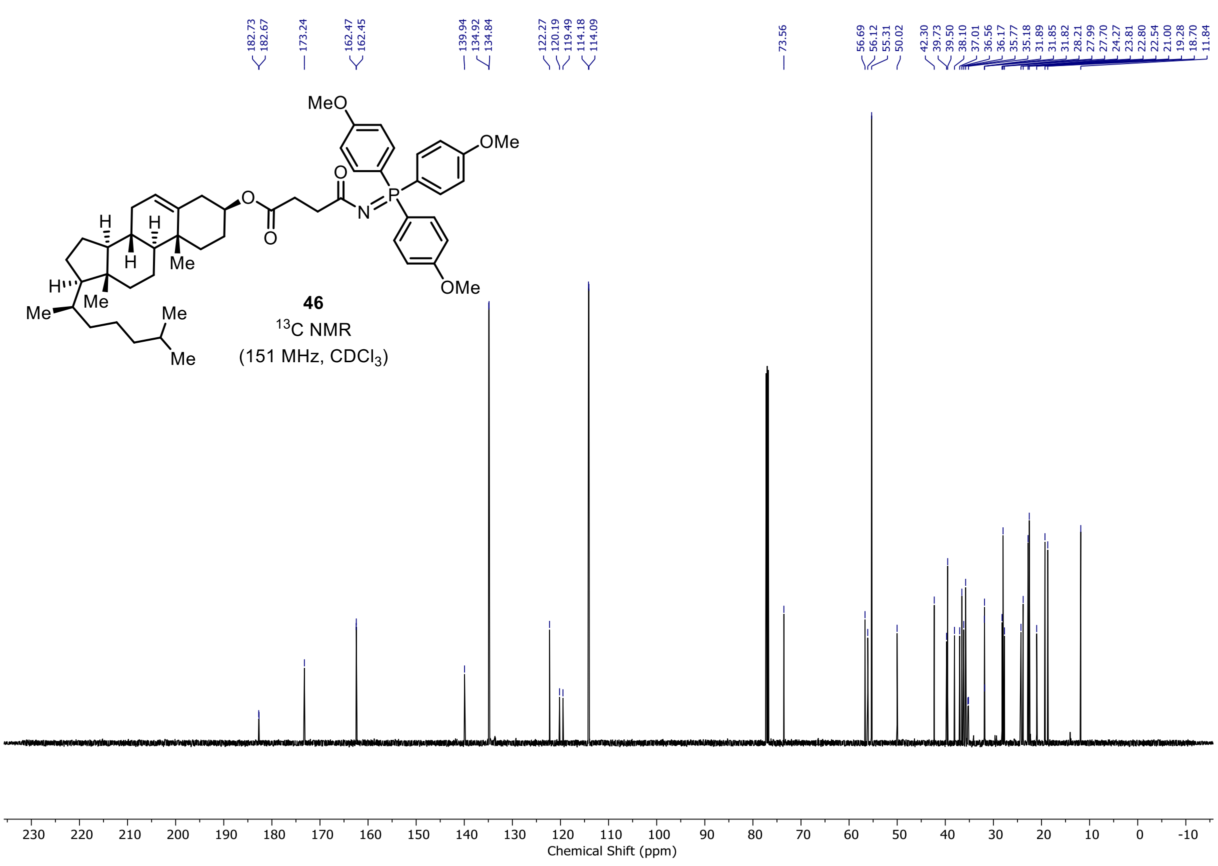
46
^{13}C NMR
(151 MHz, CDCl$_3$)
Chemical Shift (ppm)

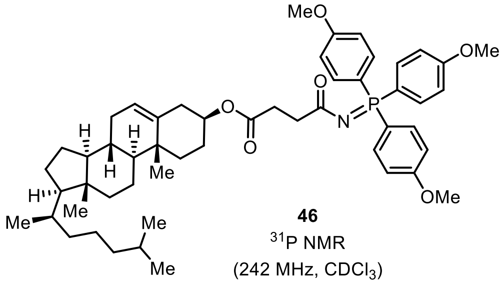

46
^{31}P NMR
(242 MHz, $CDCl_3$)

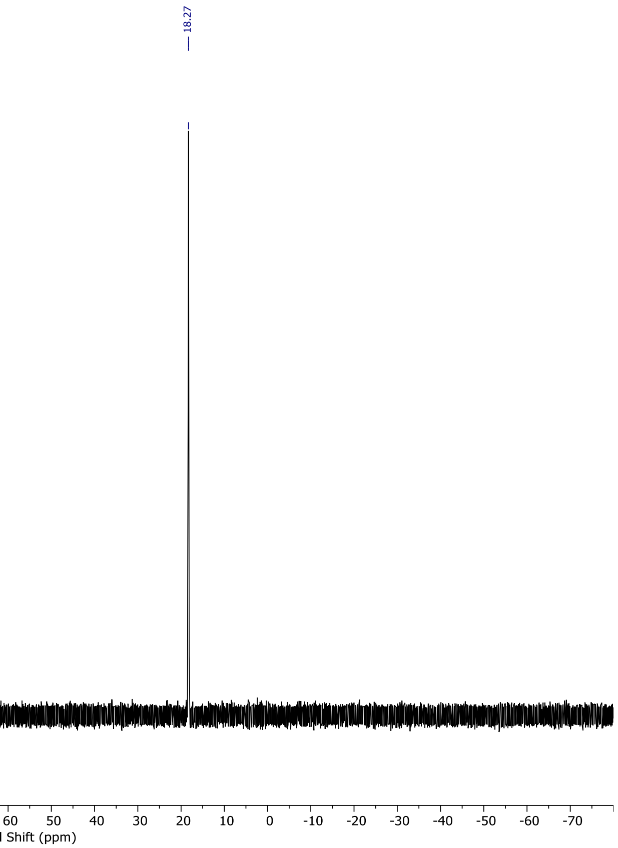

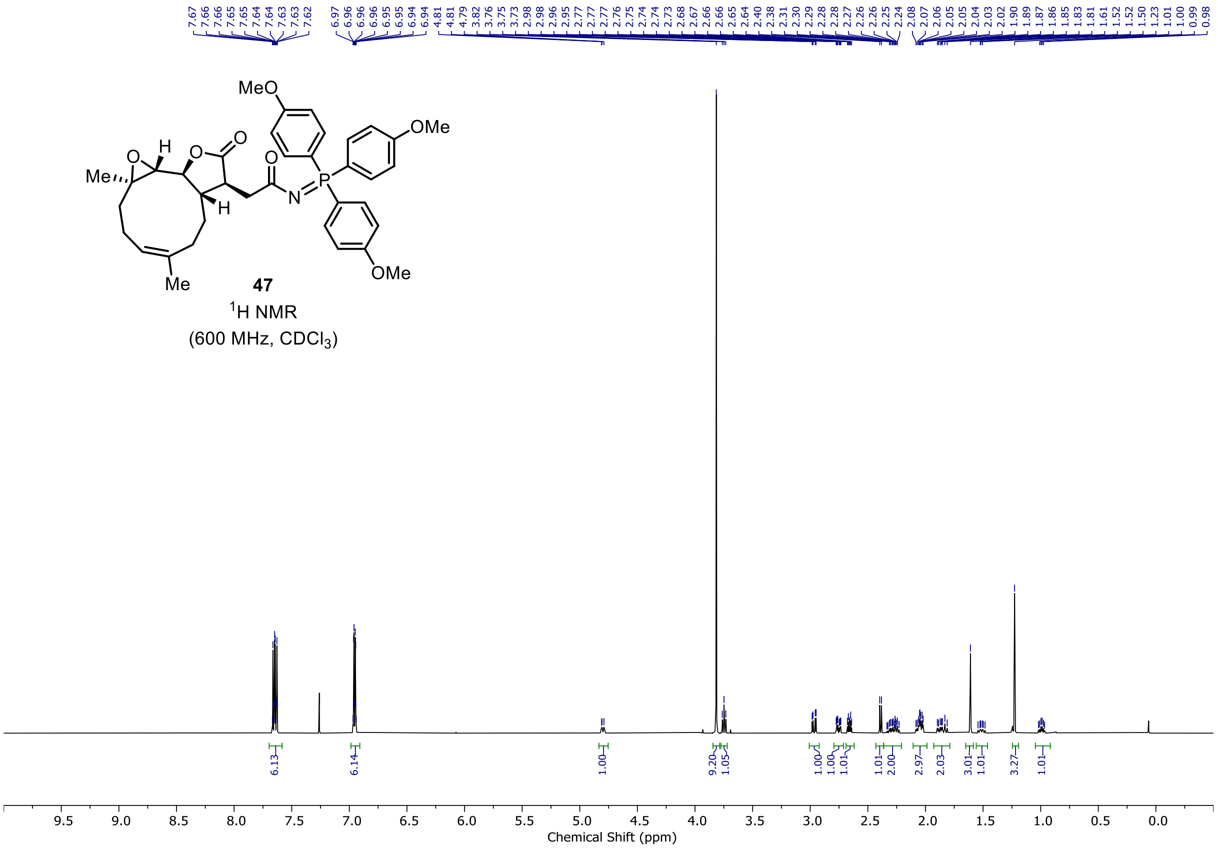

47

^{1}H NMR

(600 MHz, $CDCl_3$)

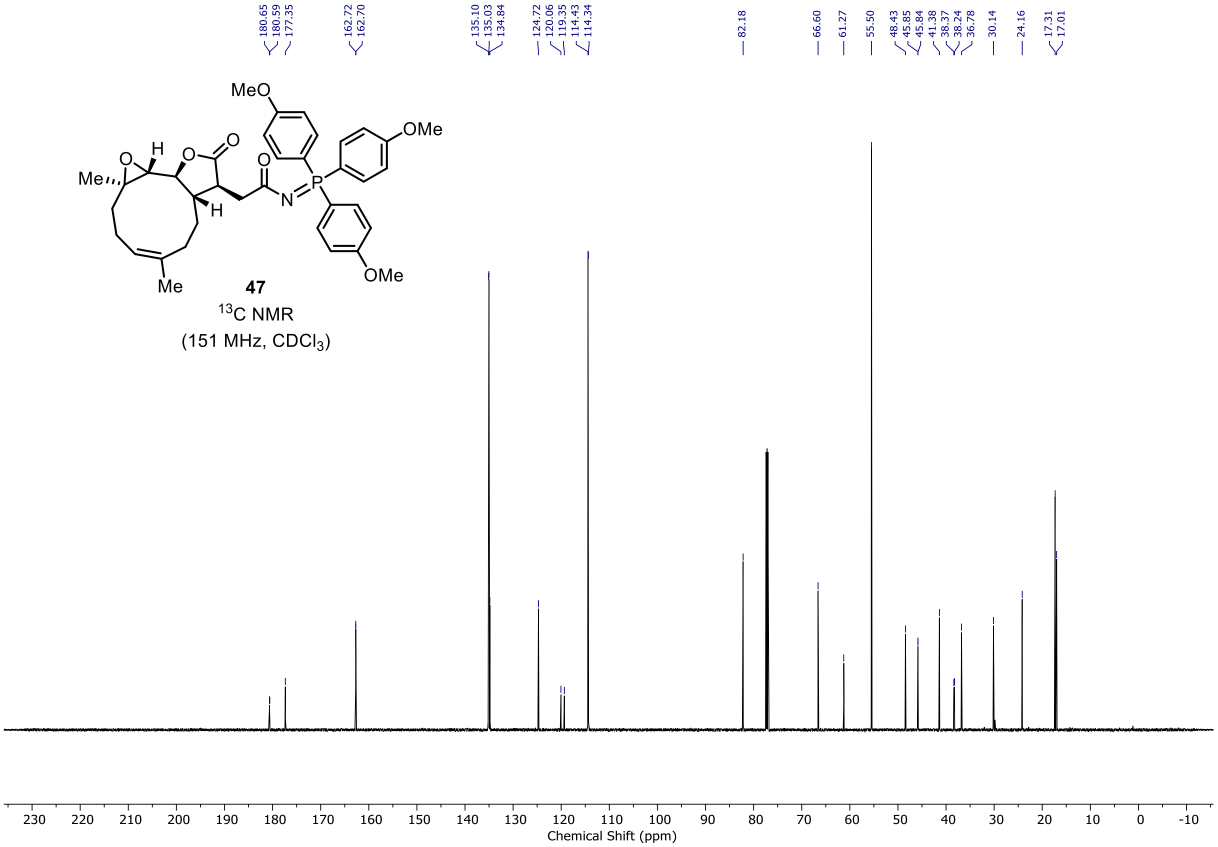

47

^{13}C NMR

(151 MHz, $CDCl_3$)

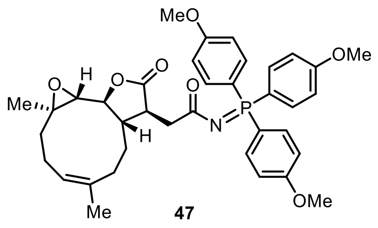

47
^{31}P NMR
(242 MHz, $CDCl_3$)

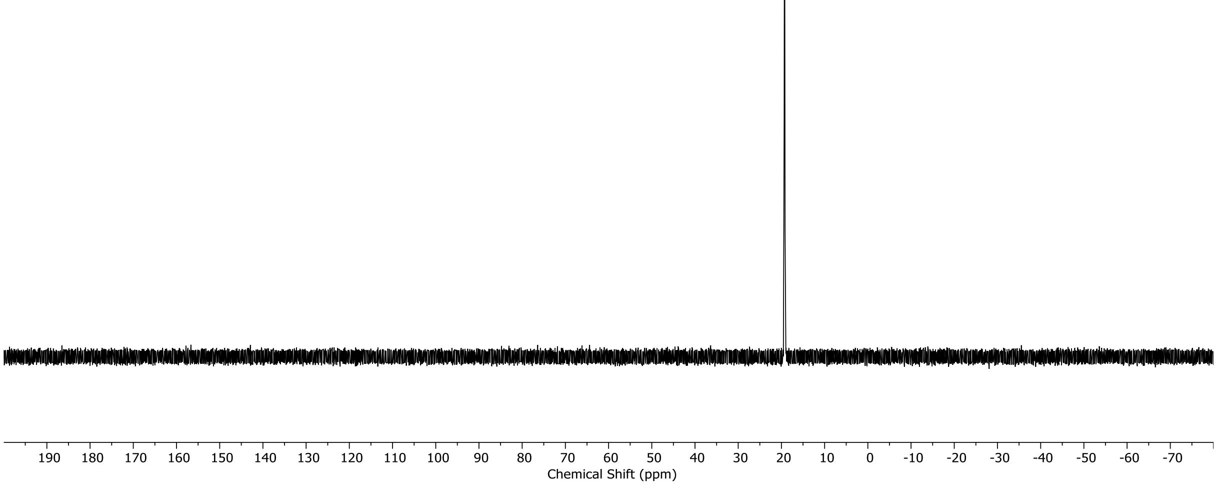

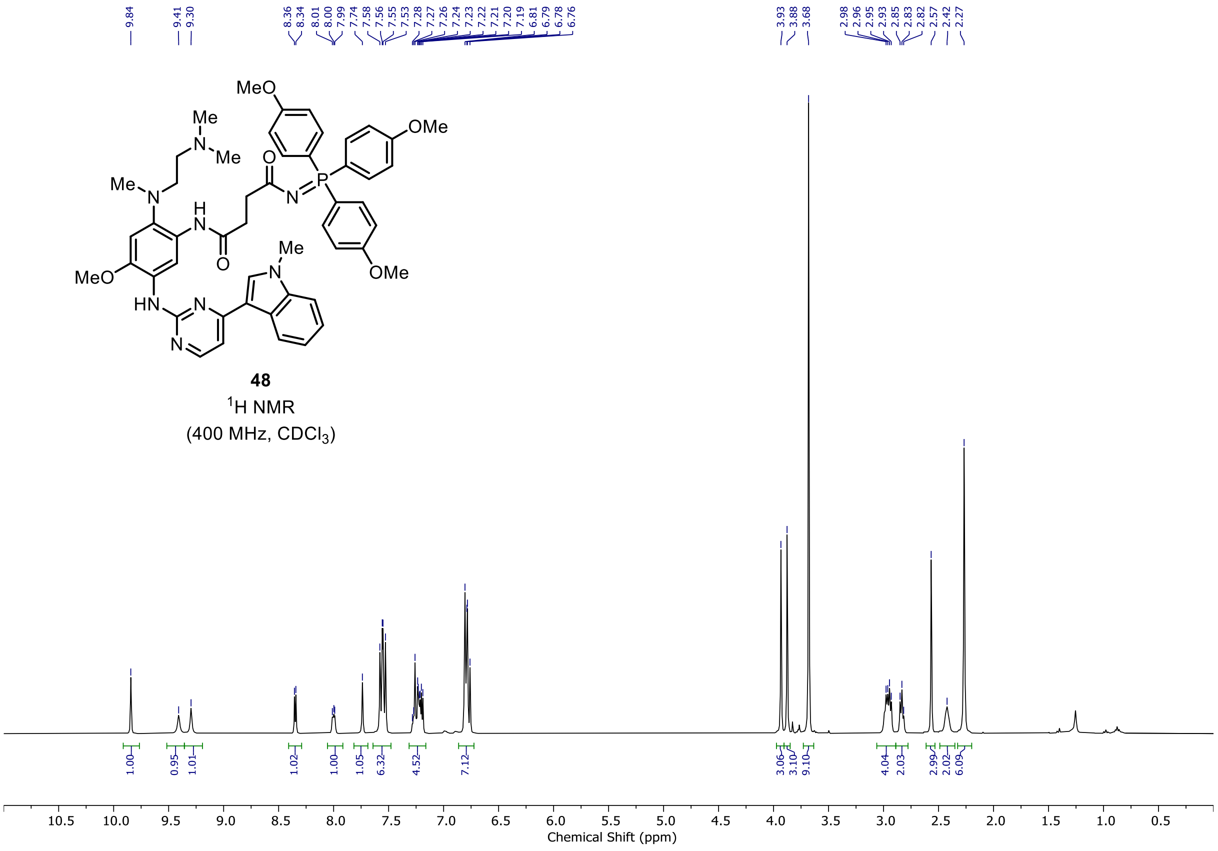

48
^{1}H NMR
(400 MHz, $CDCl_3$)

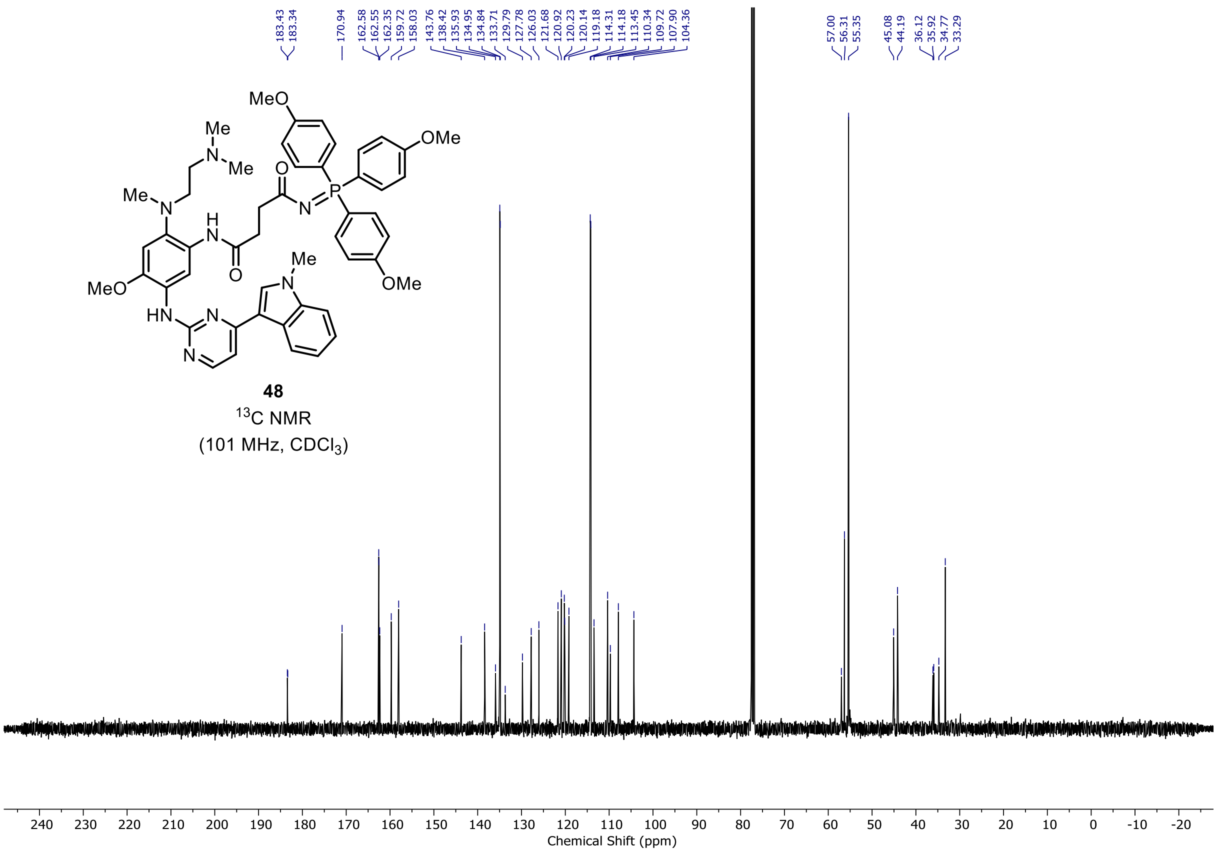

48
^{13}C NMR
(101 MHz, $CDCl_3$)

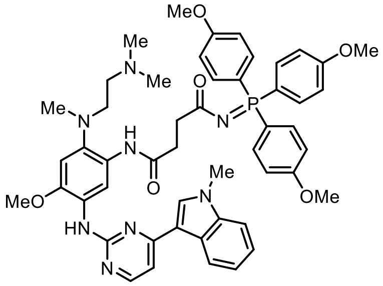

48

^{31}P NMR

(162 MHz, $CDCl_3$)

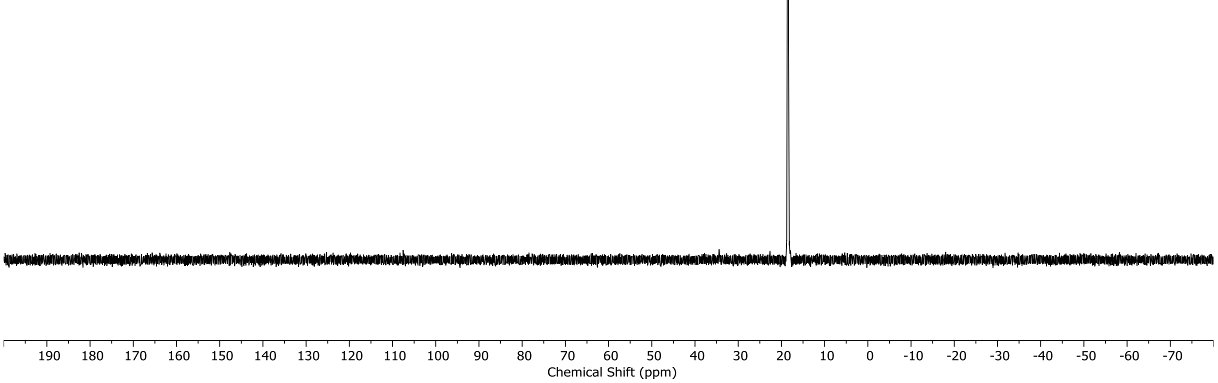

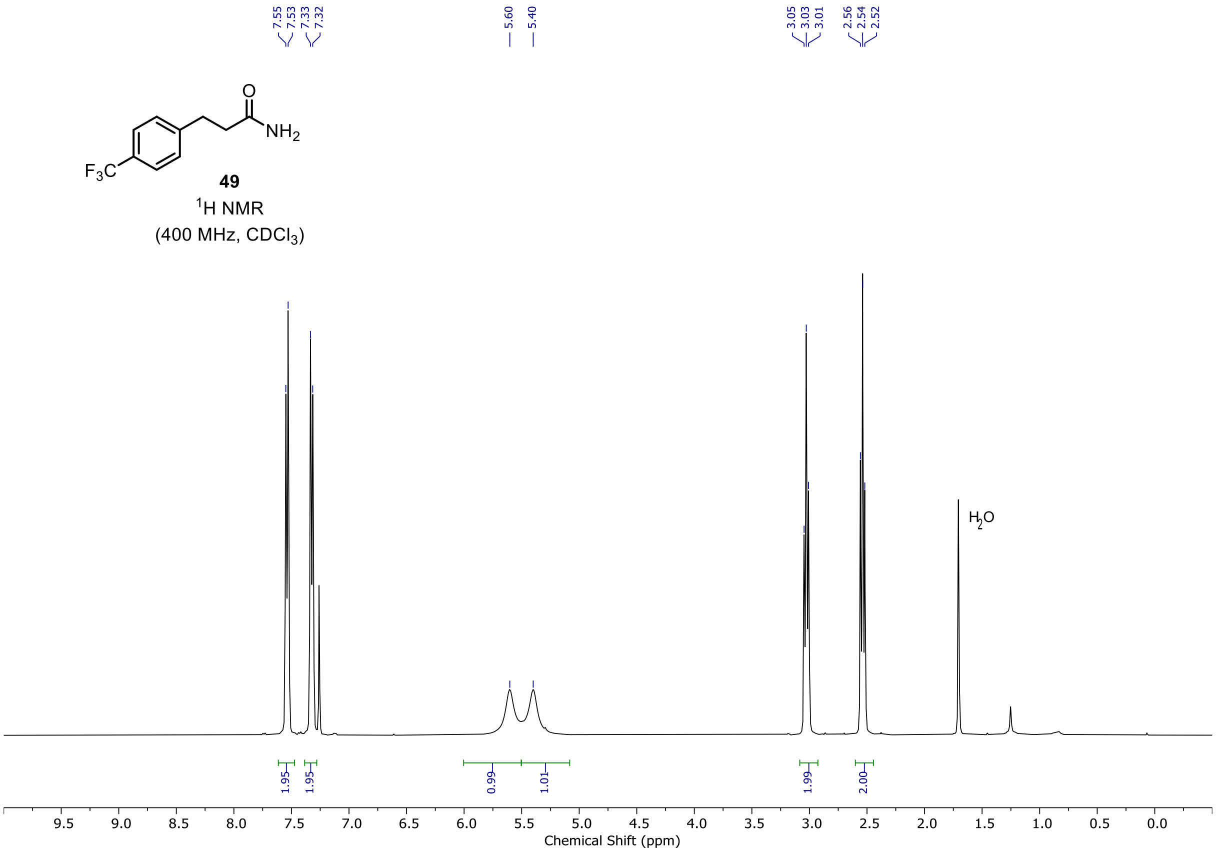
7.55
7.53
7.33
7.32
5.60
5.40
3.05
3.03
3.01
2.56
2.54
2.52
O
NH2
F3C
49
1H NMR
(400 MHz, CDCl3)
H2O
1.95
1.95
0.99
1.01
1.99
2.00
9.5 9.0 8.5 8.0 7.5 7.0 6.5 6.0 5.5 5.0 4.5 4.0 3.5 3.0 2.5 2.0 1.5 1.0 0.5 0.0
Chemical Shift (ppm)

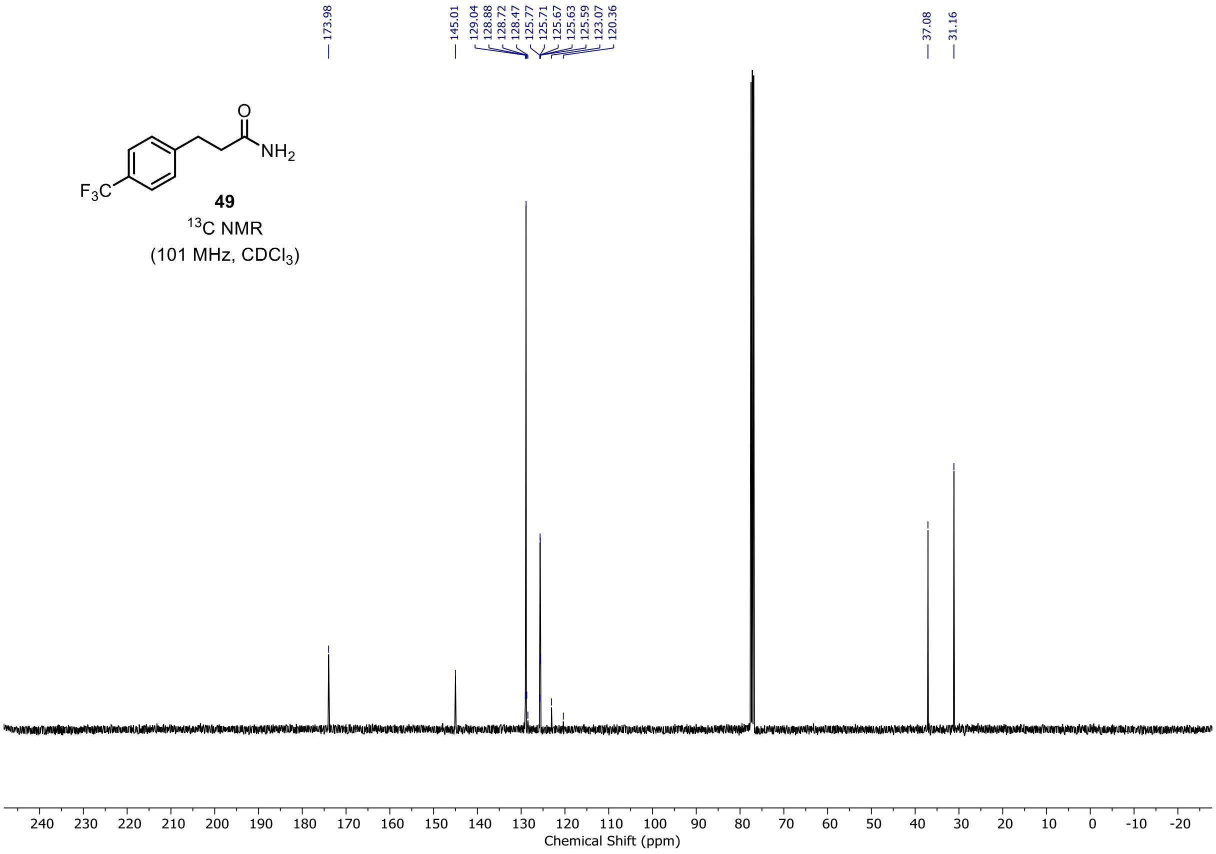
173.98
145.01
129.04
128.88
128.72
128.47
125.77
125.71
125.67
125.63
125.59
123.07
120.36
37.08
31.16
O
NH2
F3C
49
13C NMR
(101 MHz, CDCl3)
240 230 220 210 200 190 180 170 160 150 140 130 120 110 100 90 80 70 60 50 40 30 20 10 0 -10 -20
Chemical Shift (ppm)

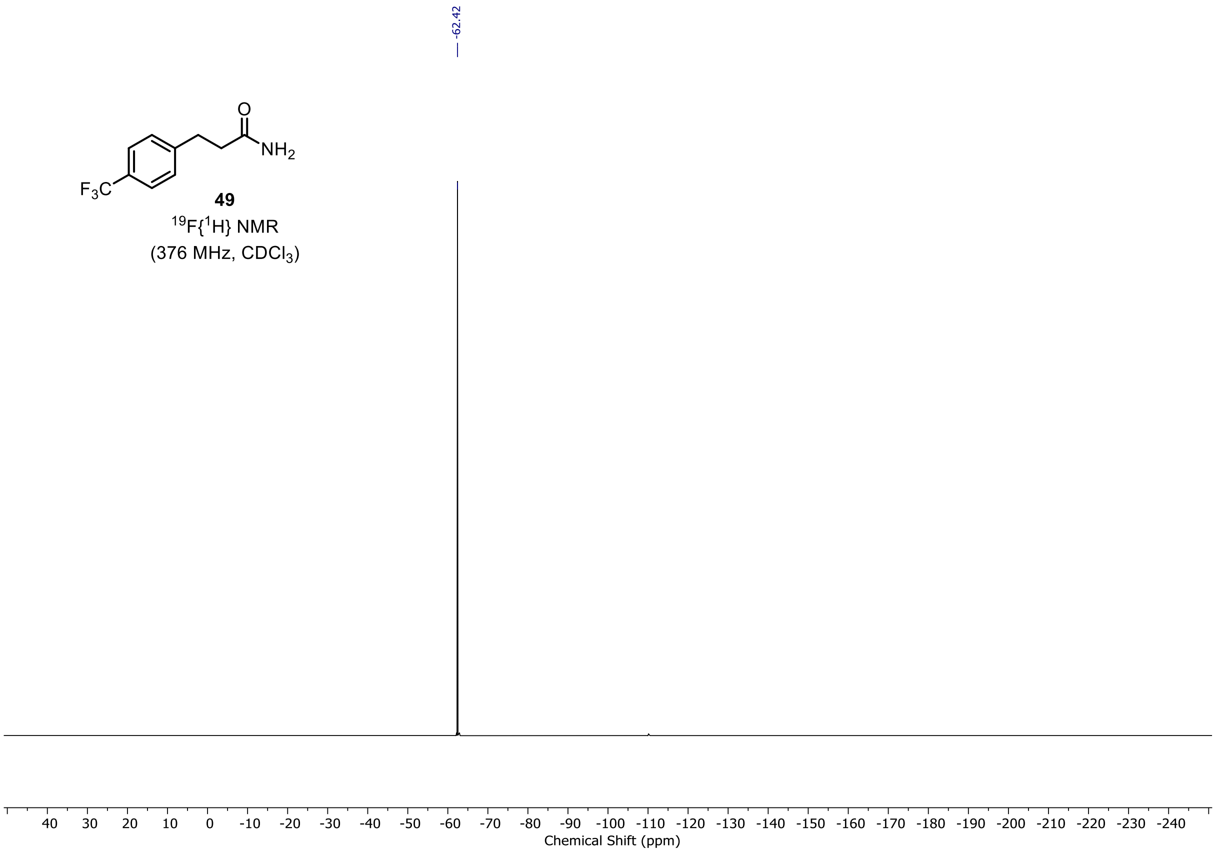
-62.42
F3C
O
NH2
49
19F{1H} NMR
(376 MHz, CDCl3)
40 30 20 10 0 -10 -20 -30 -40 -50 -60 -70 -80 -90 -100 -110 -120 -130 -140 -150 -160 -170 -180 -190 -200 -210 -220 -230 -240
Chemical Shift (ppm)

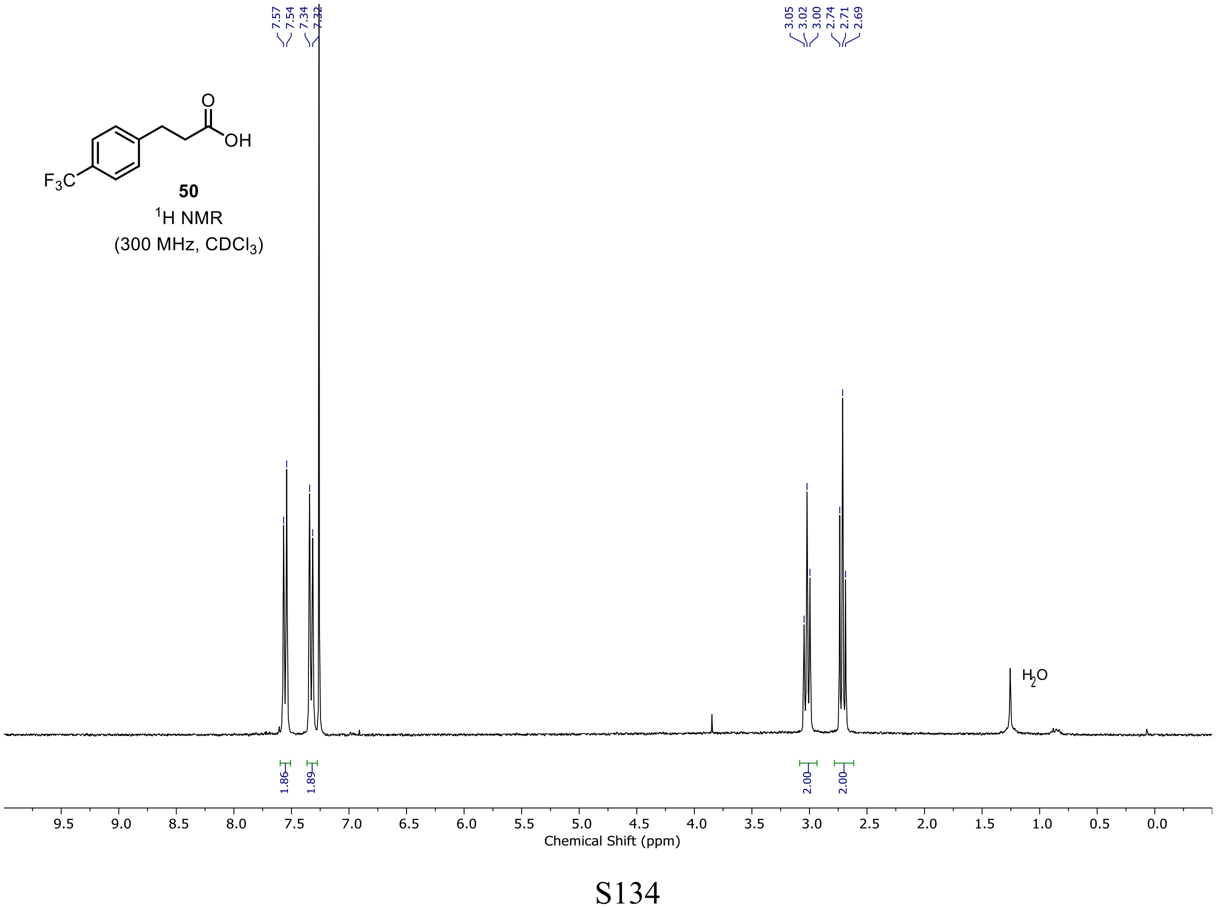
7.57
7.54
7.34
7.32
3.05
3.02
3.00
2.74
2.71
2.69
F3C
O
OH
50
1H NMR
(300 MHz, CDCl3)
H2O
1.86
1.89
2.00
2.00
9.5 9.0 8.5 8.0 7.5 7.0 6.5 6.0 5.5 5.0 4.5 4.0 3.5 3.0 2.5 2.0 1.5 1.0 0.5 0.0
Chemical Shift (ppm)

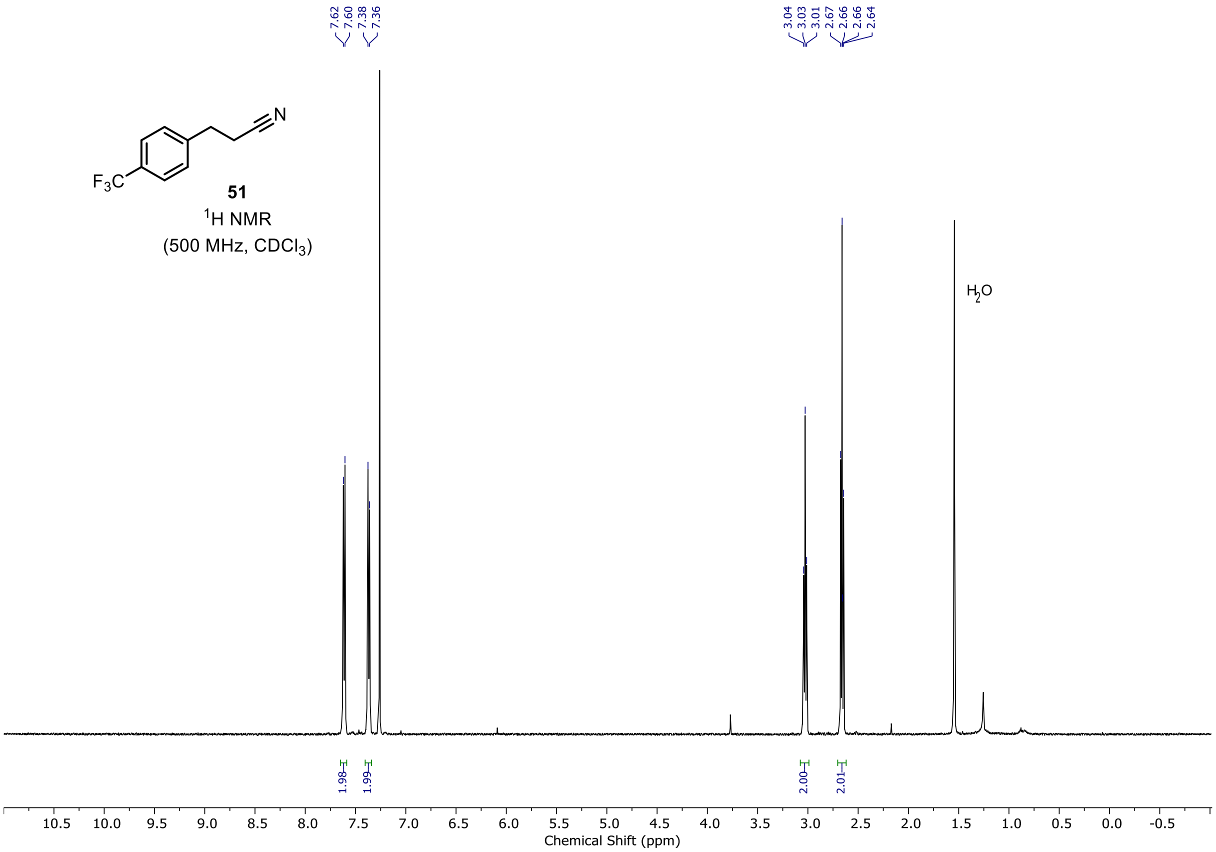

7.62
7.60
7.38
7.36
3.04
3.03
3.01
2.67
2.66
2.66
2.64
F3C
N
51
1H NMR
(500 MHz, CDCl3)
H2O
1.98
1.99
2.00
2.01
10.5 10.0 9.5 9.0 8.5 8.0 7.5 7.0 6.5 6.0 5.5 5.0 4.5 4.0 3.5 3.0 2.5 2.0 1.5 1.0 0.5 0.0 -0.5
Chemical Shift (ppm)

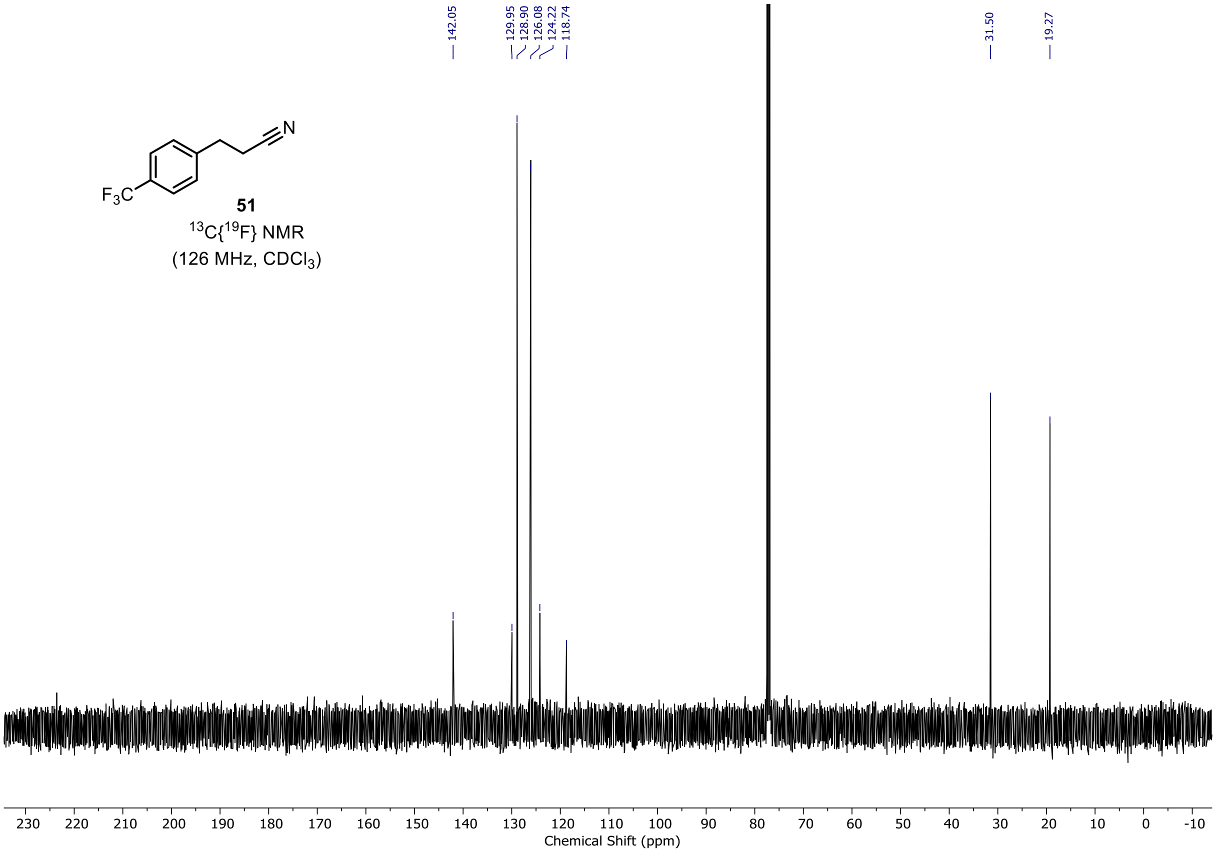

142.05
129.95
128.90
126.08
124.22
118.74
31.50
19.27
F3C
N
51
13C{19F} NMR
(126 MHz, CDCl3)
230 220 210 200 190 180 170 160 150 140 130 120 110 100 90 80 70 60 50 40 30 20 10 0 -10
Chemical Shift (ppm)

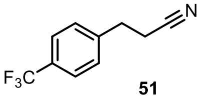

51

$^{19}F\{^{1}H\}$ NMR

(470 MHz, $CDCl_3$)

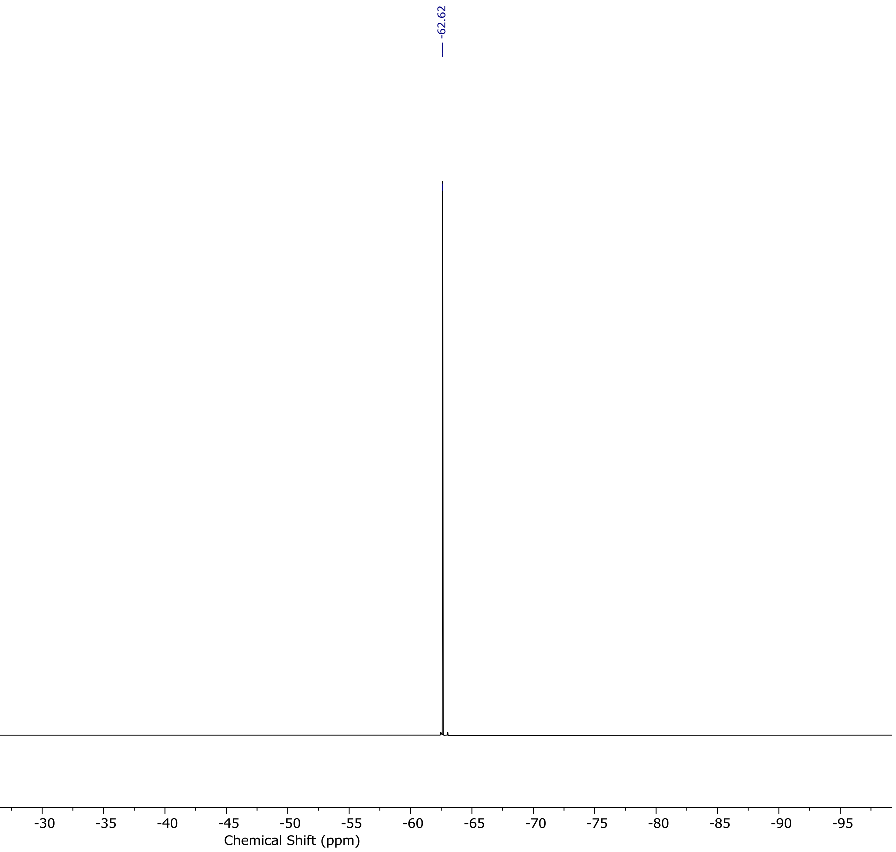

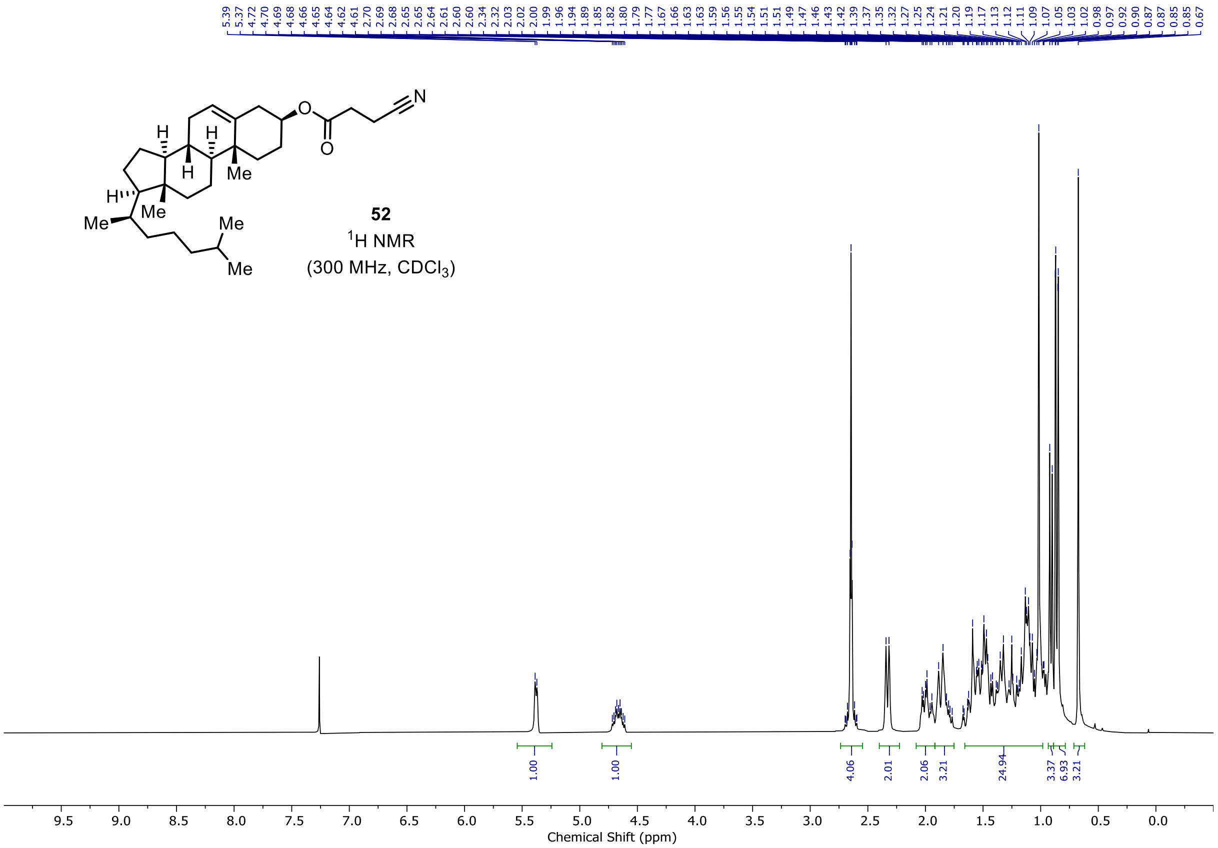

5.39 5.37 4.72 4.70 4.69 4.68 4.66 4.65 4.64 4.62 4.61 2.70 2.69 2.68 2.65 2.65 2.64 2.61 2.60 2.60 2.34 2.32 2.03 2.02 2.00 1.99 1.96 1.94 1.89 1.85 1.82 1.80 1.79 1.77 1.67 1.66 1.63 1.59 1.56 1.55 1.54 1.51 1.51 1.49 1.47 1.46 1.43 1.42 1.39 1.37 1.35 1.32 1.27 1.25 1.24 1.21 1.20 1.19 1.17 1.13 1.12 1.11 1.09 1.07 1.05 1.03 1.02 0.98 0.97 0.92 0.90 0.87 0.85 0.85 0.67
H
H
H
H
Me
Me
Me
Me
Me
O
O
N
52
1H NMR
(300 MHz, CDCl3)
1.00 1.00 4.06 2.01 2.06 3.21 24.94 3.37 6.93 3.21
9.5 9.0 8.5 8.0 7.5 7.0 6.5 6.0 5.5 5.0 4.5 4.0 3.5 3.0 2.5 2.0 1.5 1.0 0.5 0.0
Chemical Shift (ppm)

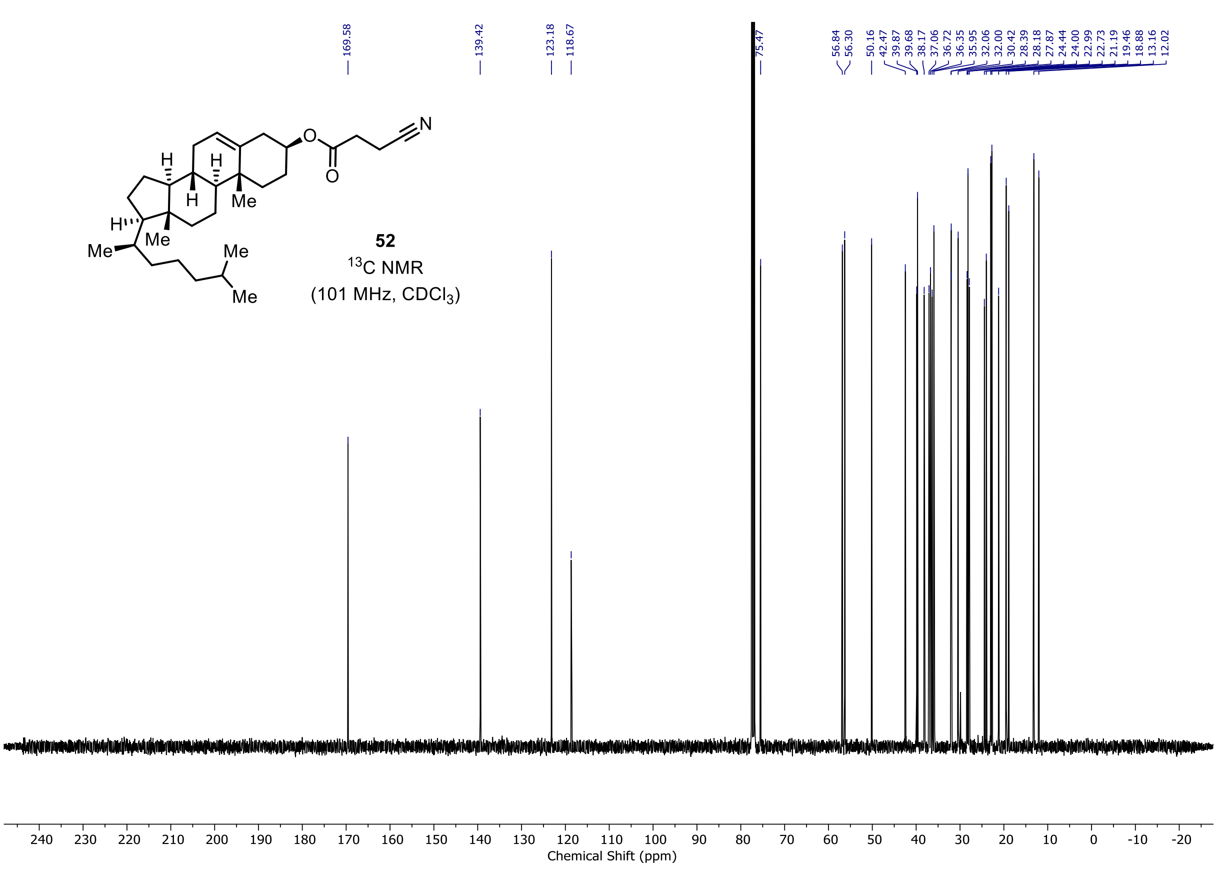

169.58 139.42 123.18 118.67 75.47 56.84 56.30 50.16 42.47 39.87 39.68 38.17 37.06 36.72 36.35 35.95 32.06 32.00 30.42 28.39 28.18 27.87 24.44 24.00 22.99 22.73 21.19 19.46 18.88 13.16 12.02
H
H
H
H
Me
Me
Me
Me
Me
O
O
N
52
13C NMR
(101 MHz, CDCl3)
240 230 220 210 200 190 180 170 160 150 140 130 120 110 100 90 80 70 60 50 40 30 20 10 0 -10 -20
Chemical Shift (ppm)

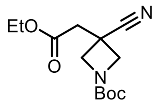

53

^{1}H NMR

(400 MHz, $CDCl_3$)

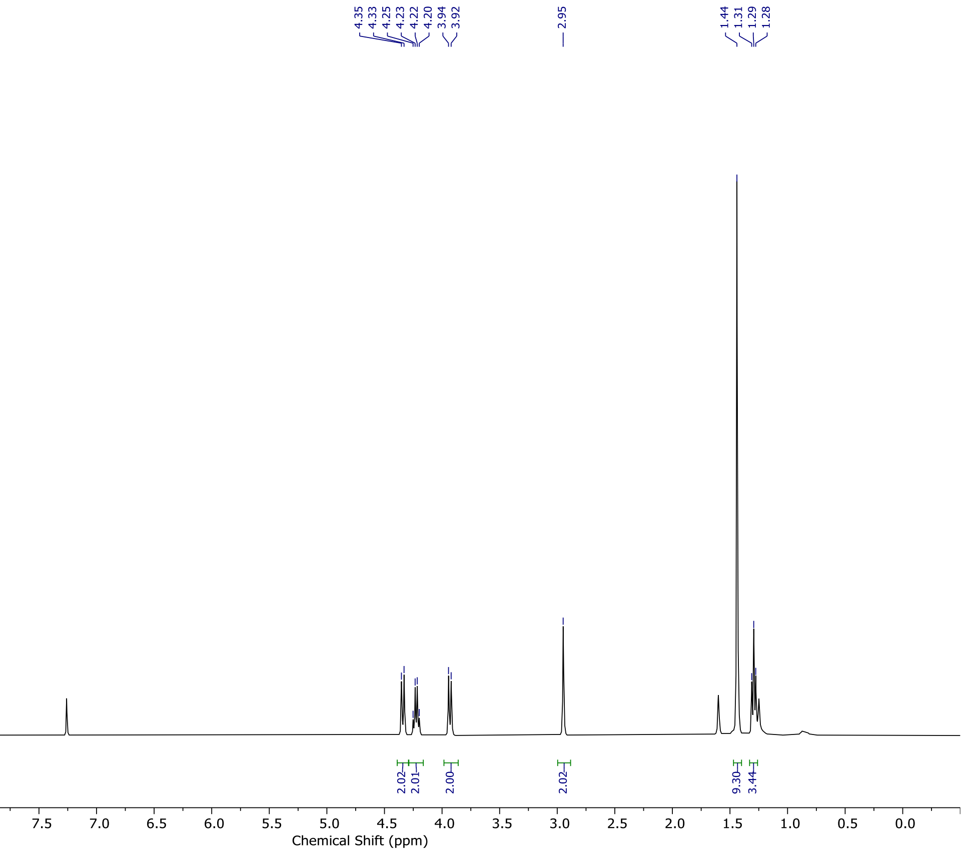

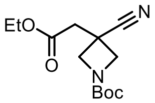

53

^{13}C NMR

(101 MHz, $CDCl_3$)

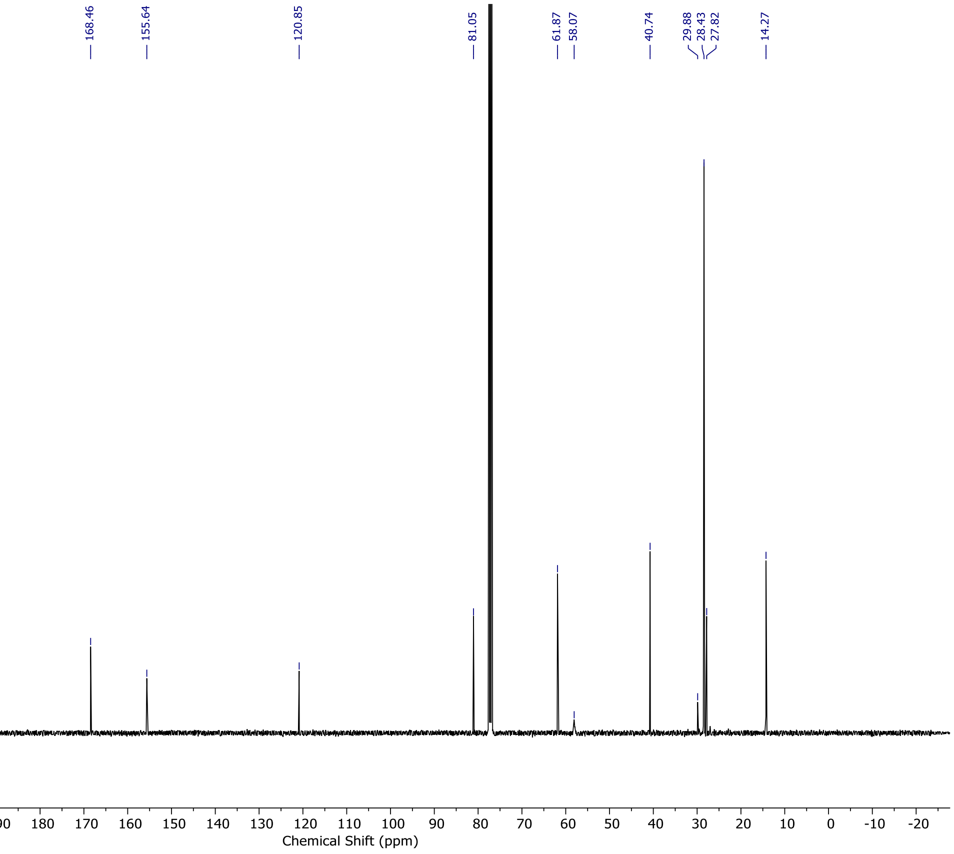

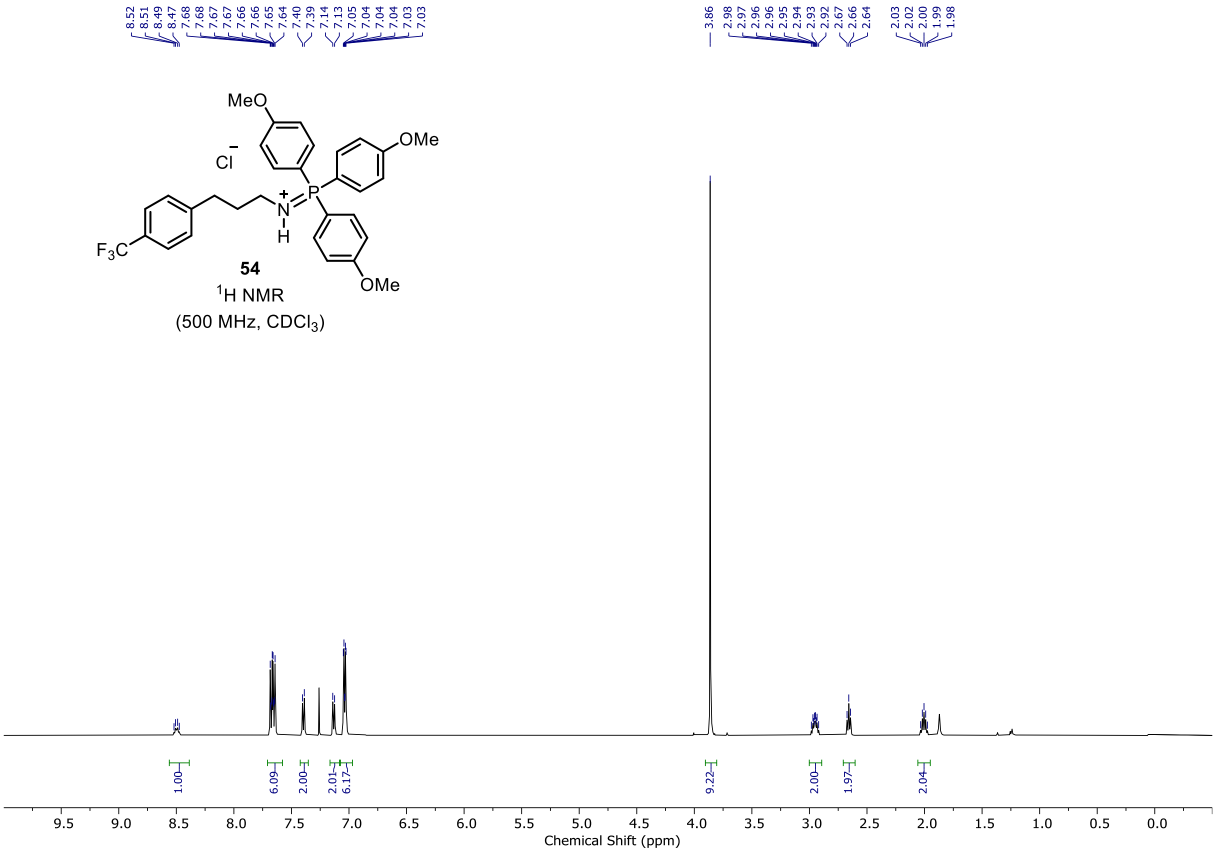
8.52
8.51
8.49
8.47
7.68
7.68
7.67
7.67
7.66
7.66
7.65
7.64
7.40
7.39
7.14
7.13
7.05
7.04
7.04
7.04
7.03
7.03
3.86
2.98
2.97
2.96
2.96
2.95
2.94
2.93
2.92
2.67
2.66
2.64
2.03
2.02
2.00
1.99
1.98
MeO
OMe
OMe
Cl
F3C
N
H
P
54
1H NMR
(500 MHz, CDCl3)
1.00
6.09
2.00
2.01
6.17
9.22
2.00
1.97
2.04
Chemical Shift (ppm)

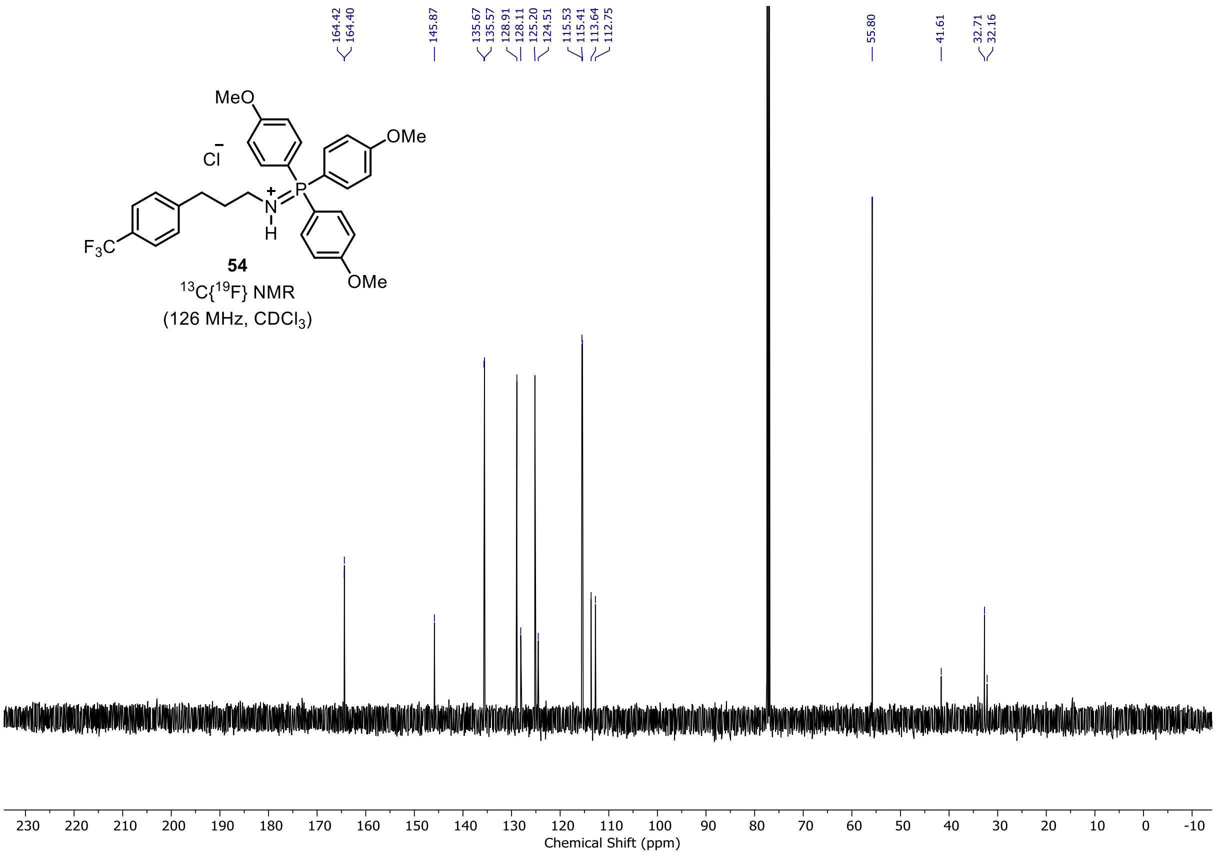
164.42
164.40
145.87
135.67
135.57
128.91
128.11
125.20
124.51
115.53
115.41
113.64
112.75
55.80
41.61
32.71
32.16
MeO
OMe
OMe
Cl
F3C
N
H
P
54
13C{19F} NMR
(126 MHz, CDCl3)
Chemical Shift (ppm)

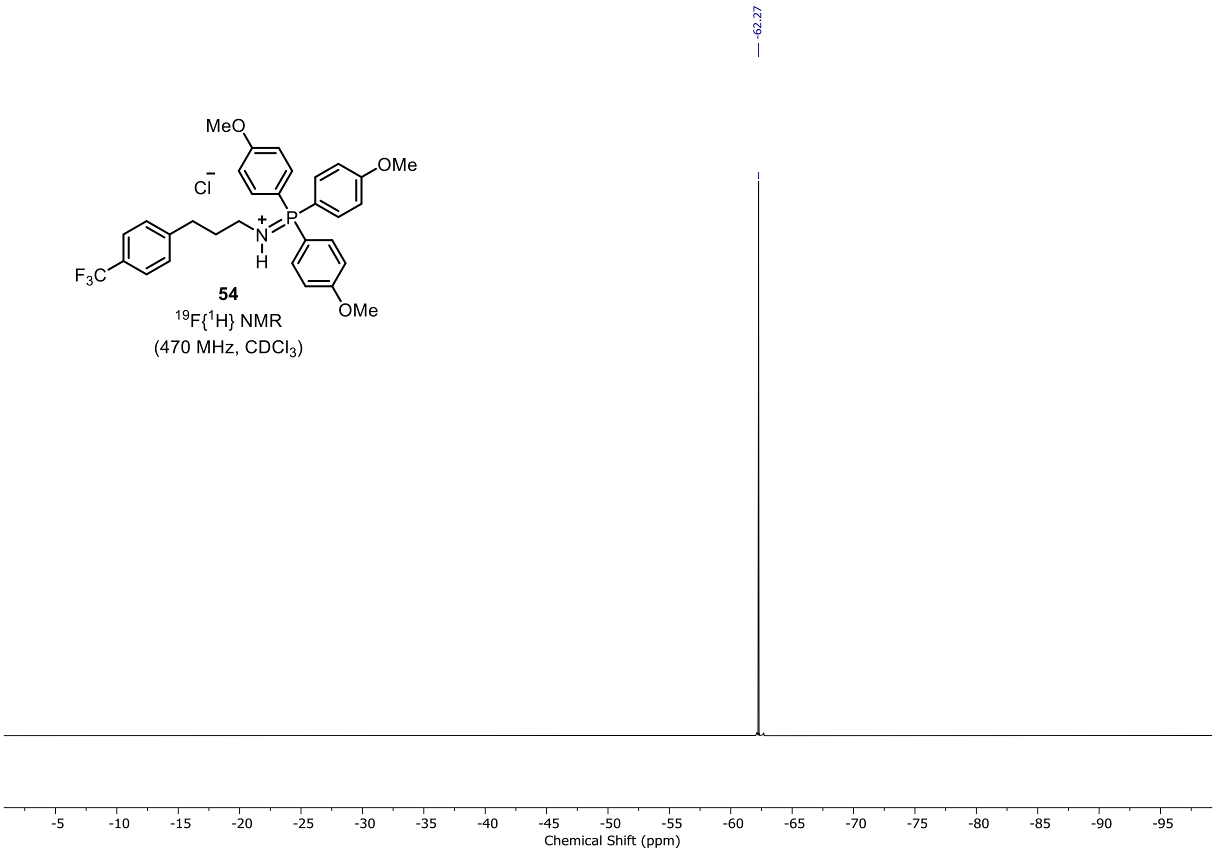

54

$^{19}F\{^{1}H\}$ NMR

(470 MHz, $CDCl_3$)

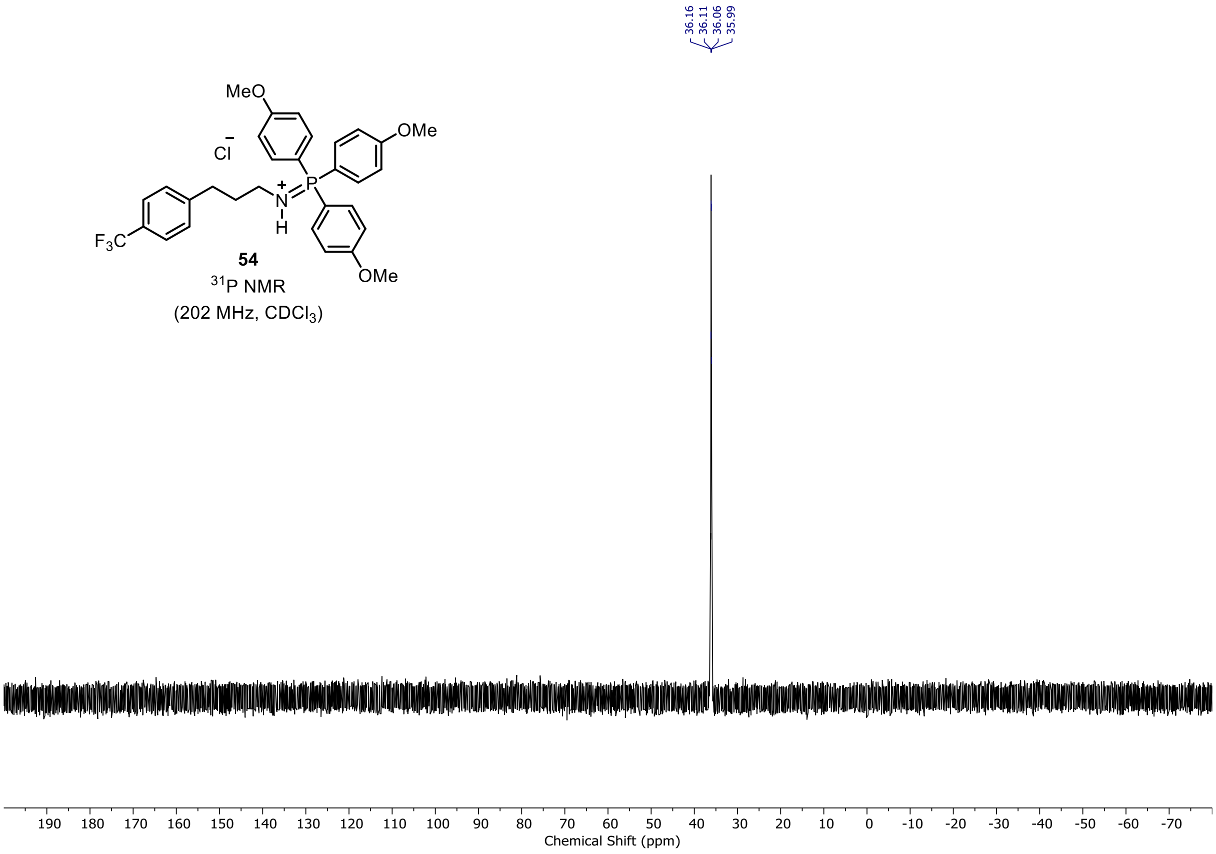

54

^{31}P NMR

(202 MHz, $CDCl_3$)

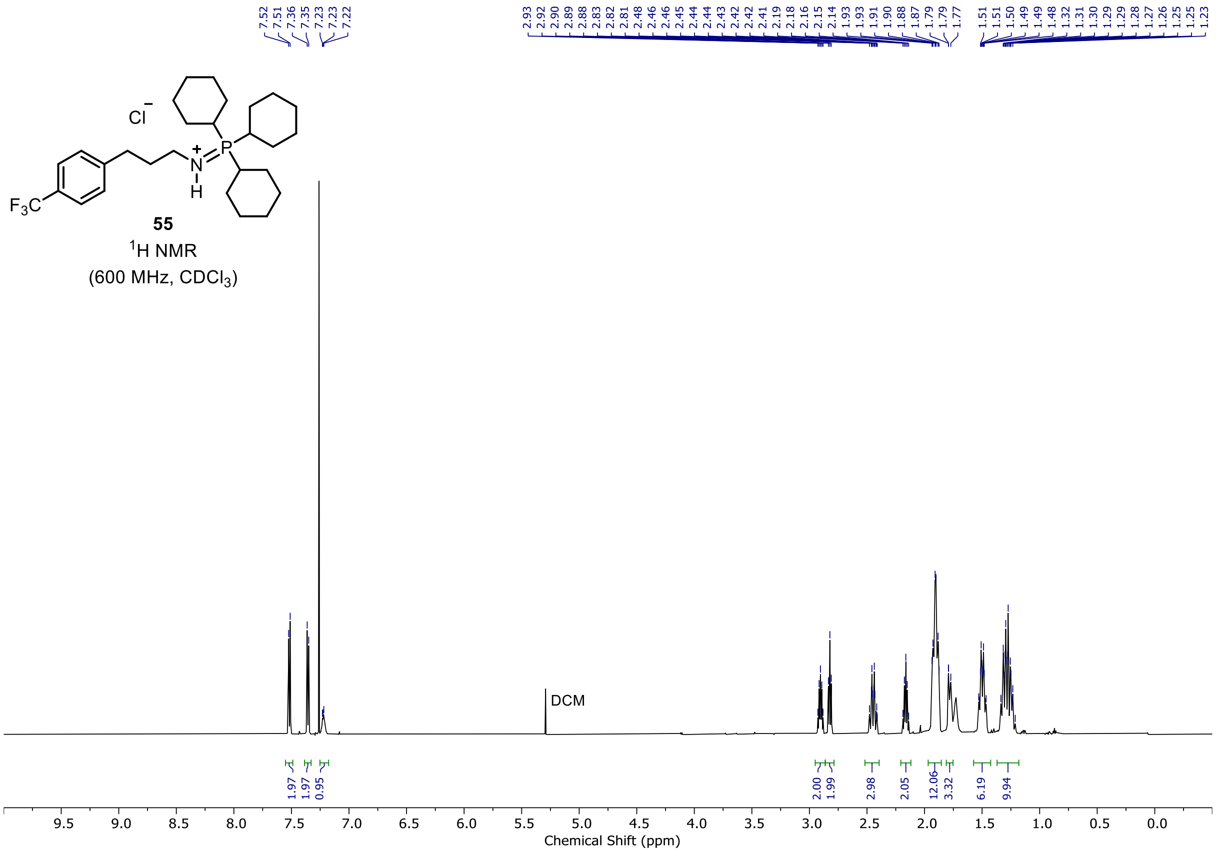

7.52
7.51
7.36
7.35
7.23
7.23
7.22
2.93
2.92
2.90
2.89
2.88
2.83
2.82
2.81
2.48
2.46
2.46
2.45
2.44
2.44
2.43
2.42
2.42
2.41
2.19
2.18
2.16
2.15
2.14
1.93
1.93
1.91
1.90
1.88
1.87
1.79
1.79
1.77
1.51
1.51
1.50
1.49
1.49
1.48
1.32
1.31
1.30
1.29
1.29
1.28
1.27
1.26
1.25
1.25
1.23
Cl
N
H
P
F_3C
55
^1H NMR
(600 MHz, $CDCl_3$)
DCM
1.97
1.97
0.95
2.00
1.99
2.98
2.05
12.06
3.32
6.19
9.94
9.5
9.0
8.5
8.0
7.5
7.0
6.5
6.0
5.5
5.0
4.5
4.0
3.5
3.0
2.5
2.0
1.5
1.0
0.5
0.0
Chemical Shift (ppm)

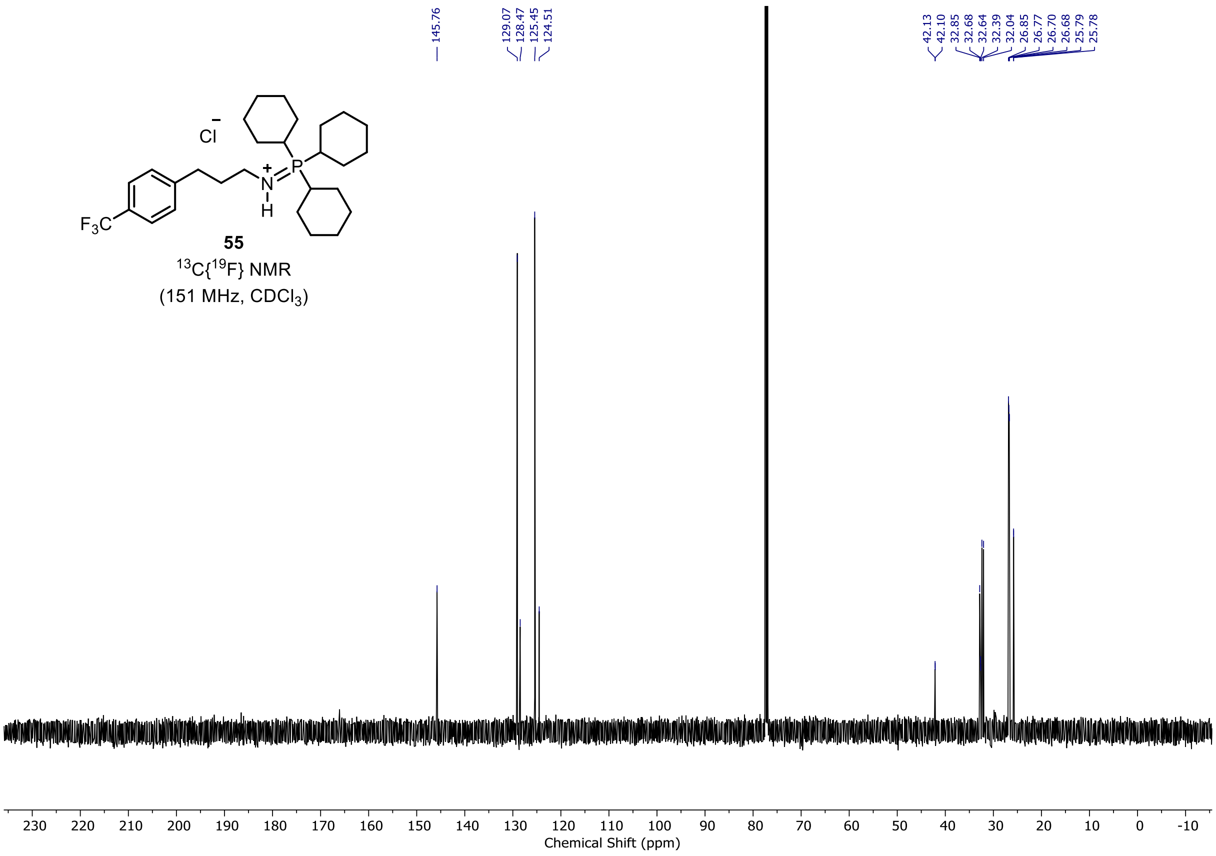

145.76
129.07
128.47
125.45
124.51
42.13
42.10
32.85
32.68
32.64
32.39
32.04
26.85
26.77
26.70
26.68
25.79
25.78
Cl
N
H
P
F_3C
55
$^{13}C\{^{19}F\}$ NMR
(151 MHz, $CDCl_3$)
230
220
210
200
190
180
170
160
150
140
130
120
110
100
90
80
70
60
50
40
30
20
10
0
-10
Chemical Shift (ppm)

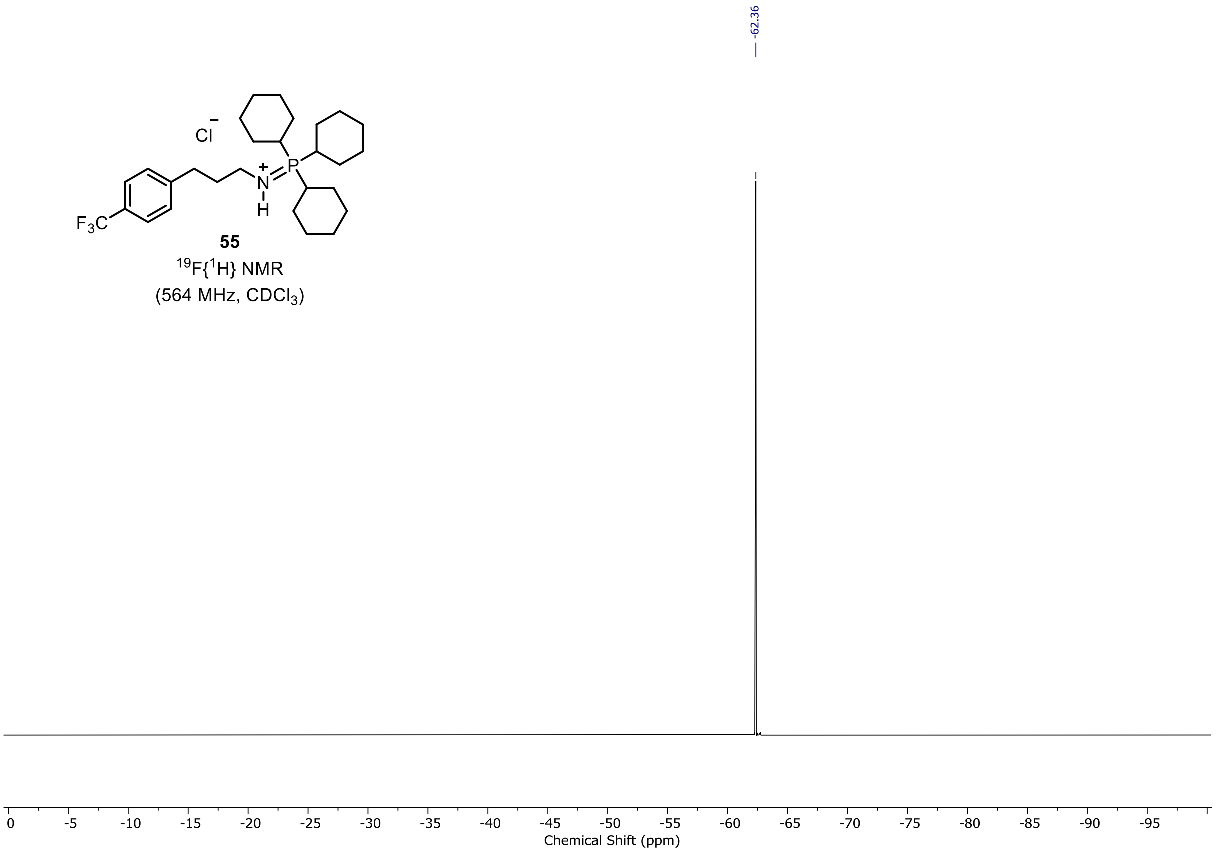
-62.36
Cl⁻
F3C
55
19F{1H} NMR
(564 MHz, CDCl3)
0 -5 -10 -15 -20 -25 -30 -35 -40 -45 -50 -55 -60 -65 -70 -75 -80 -85 -90 -95
Chemical Shift (ppm)

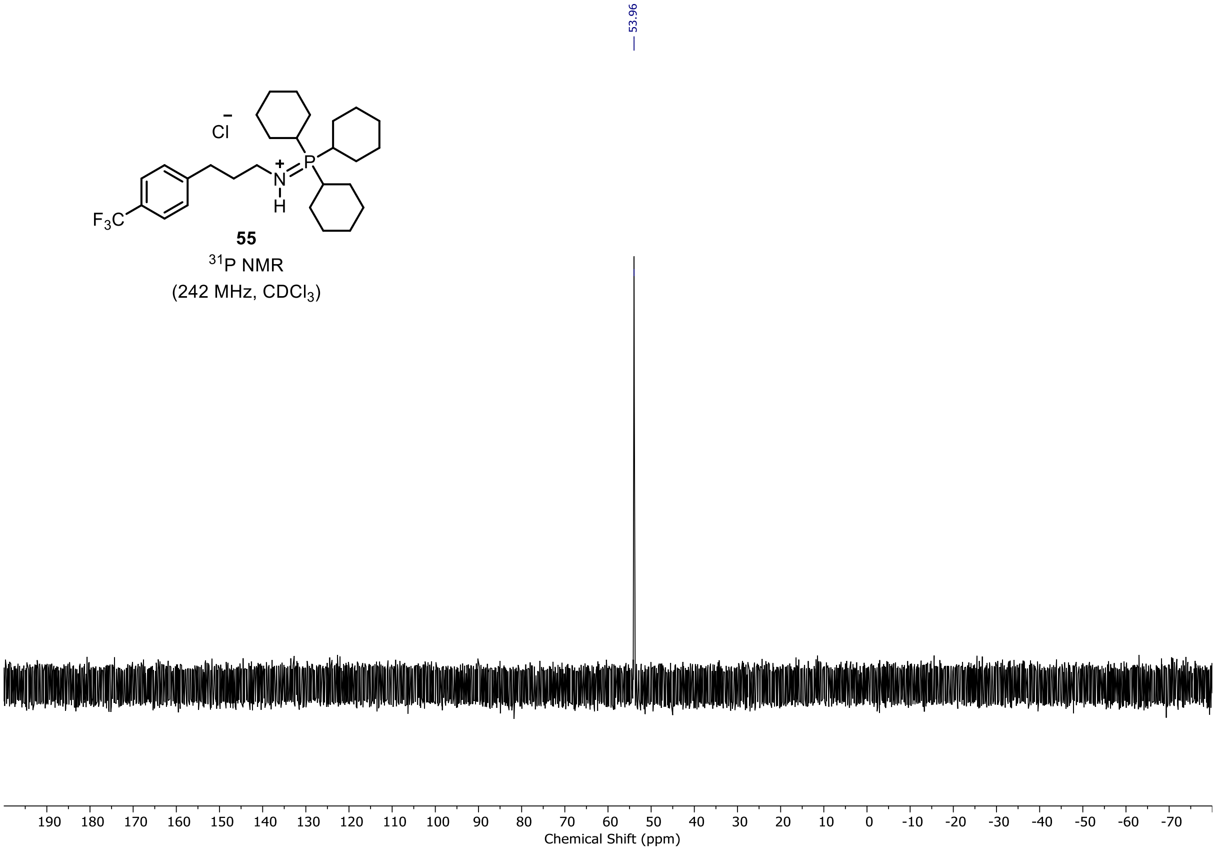
53.96
Cl⁻
F3C
55
31P NMR
(242 MHz, CDCl3)
190 180 170 160 150 140 130 120 110 100 90 80 70 60 50 40 30 20 10 0 -10 -20 -30 -40 -50 -60 -70
Chemical Shift (ppm)

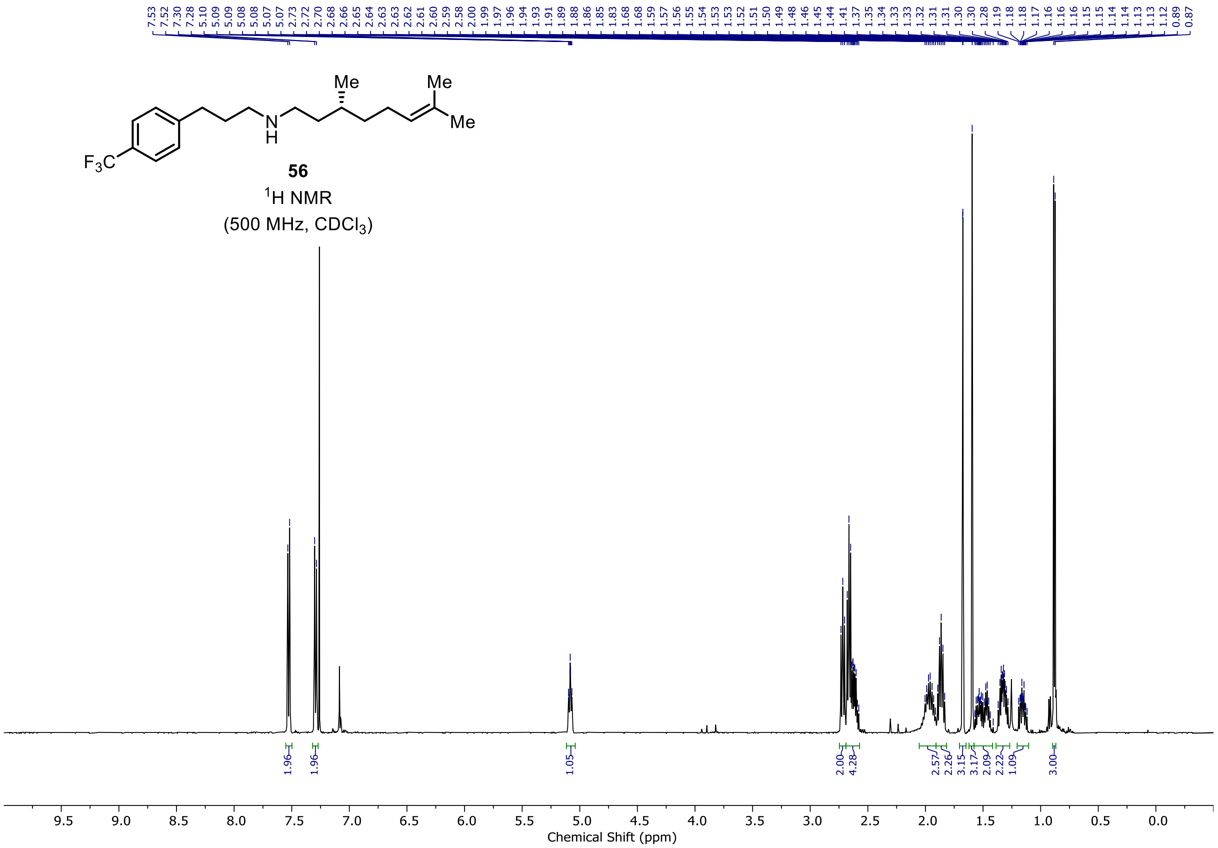

Me
Me
Me
N
H
F_3C
56
^1H NMR
(500 MHz, $CDCl_3$)
7.53 7.52 7.30 7.28 5.10 5.09 5.09 5.08 5.08 5.07 2.73 2.72 2.70 2.68 2.66 2.65 2.64 2.63 2.63 2.62 2.61 2.60 2.59 2.58 2.00 1.99 1.97 1.96 1.94 1.93 1.91 1.89 1.88 1.86 1.85 1.83 1.68 1.68 1.59 1.57 1.56 1.55 1.54 1.53 1.53 1.52 1.51 1.50 1.49 1.48 1.46 1.45 1.44 1.41 1.37 1.35 1.34 1.33 1.33 1.32 1.31 1.31 1.30 1.30 1.28 1.19 1.18 1.18 1.17 1.16 1.16 1.15 1.15 1.14 1.14 1.13 1.13 1.12 0.89 0.87
1.96 1.96 1.05 2.00 4.28 2.57 2.26 3.15 3.17 2.09 2.22 1.09 3.00
9.5 9.0 8.5 8.0 7.5 7.0 6.5 6.0 5.5 5.0 4.5 4.0 3.5 3.0 2.5 2.0 1.5 1.0 0.5 0.0
Chemical Shift (ppm)

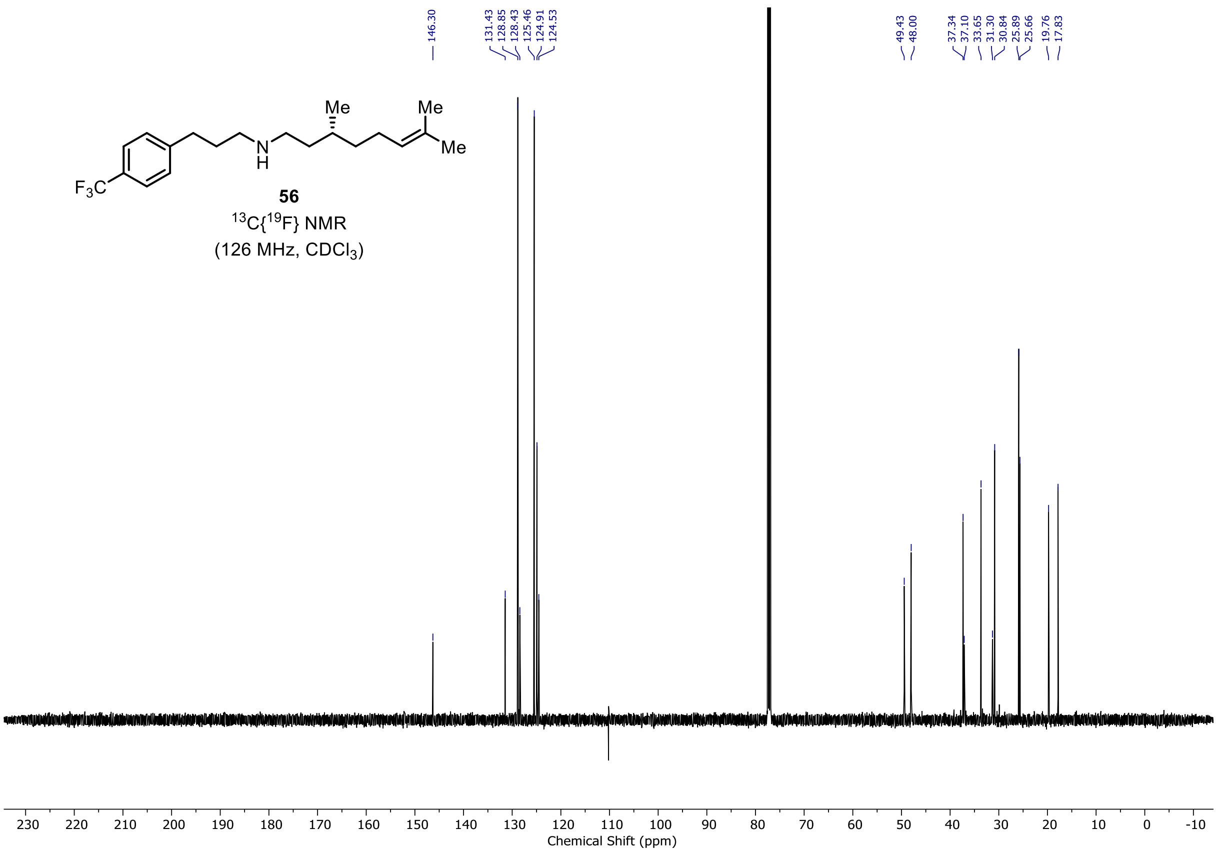

Me
Me
Me
N
H
F_3C
56
$^{13}C\{^{19}F\}$ NMR
(126 MHz, $CDCl_3$)
146.30
131.43 128.85 128.43 125.46 124.91 124.53
49.43 48.00
37.34 37.10 33.65 31.30 30.84 25.89 25.66 19.76 17.83
230 220 210 200 190 180 170 160 150 140 130 120 110 100 90 80 70 60 50 40 30 20 10 0 -10
Chemical Shift (ppm)

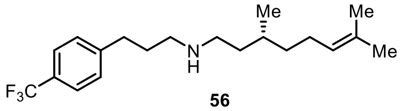

56

$^{19}F\{^1H\}$ NMR

(470 MHz, $CDCl_3$)

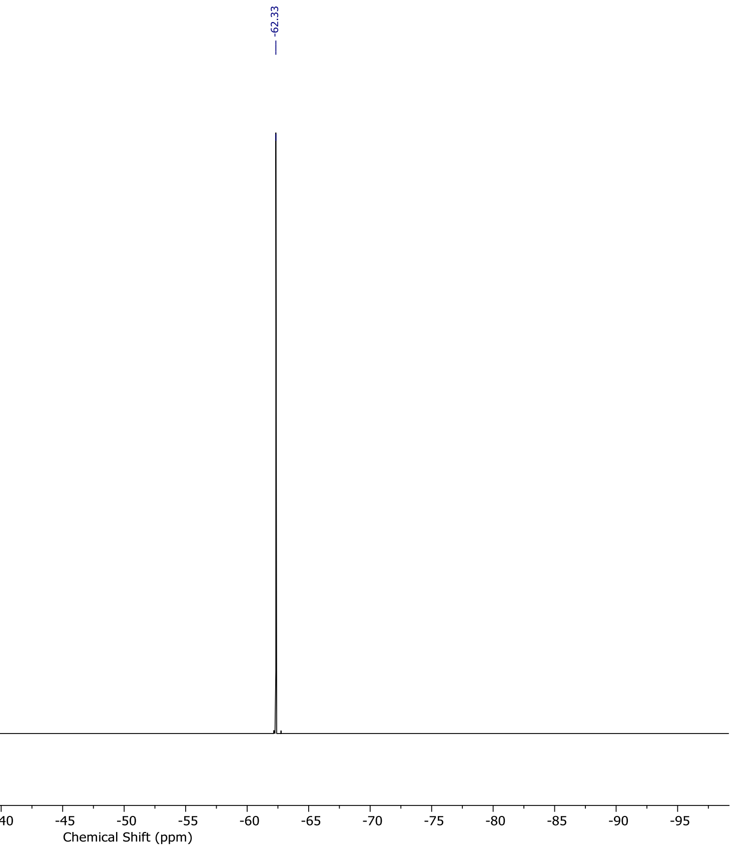

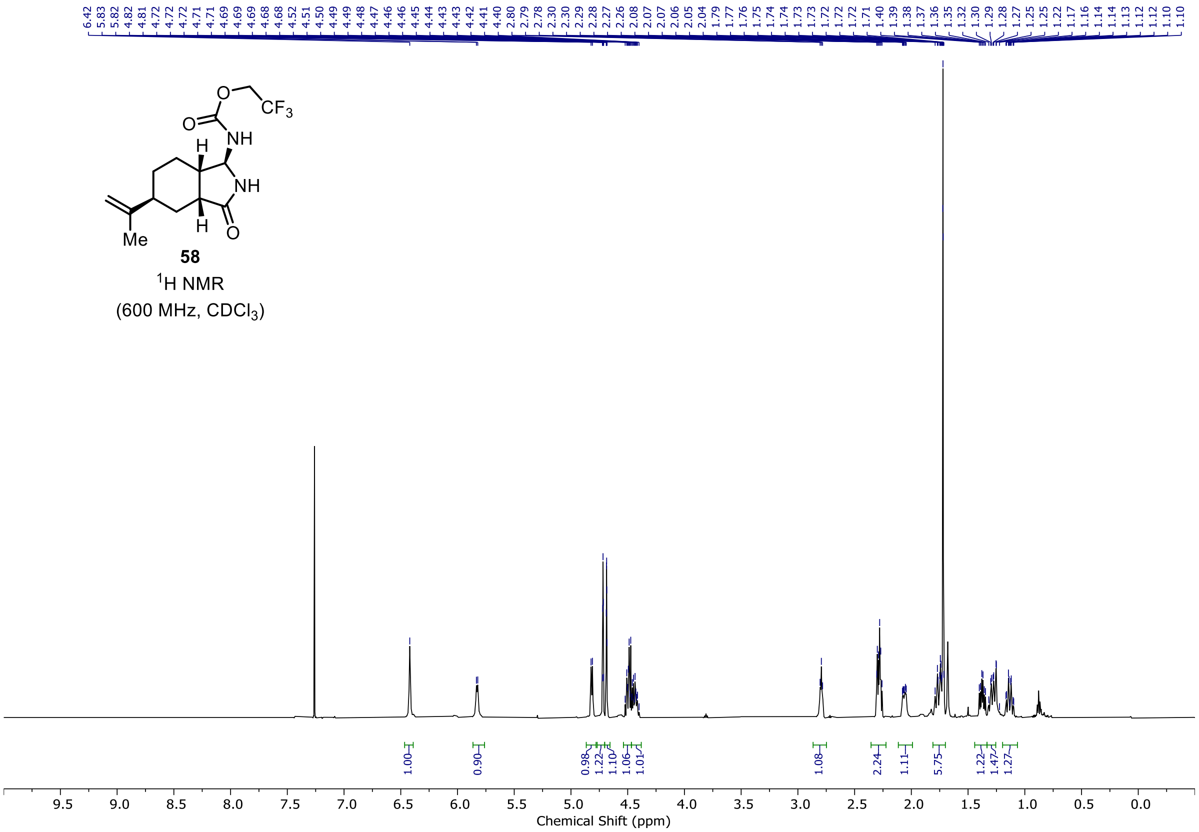
58
^{1}H NMR
(600 MHz, $CDCl_3$)
Chemical Shift (ppm)

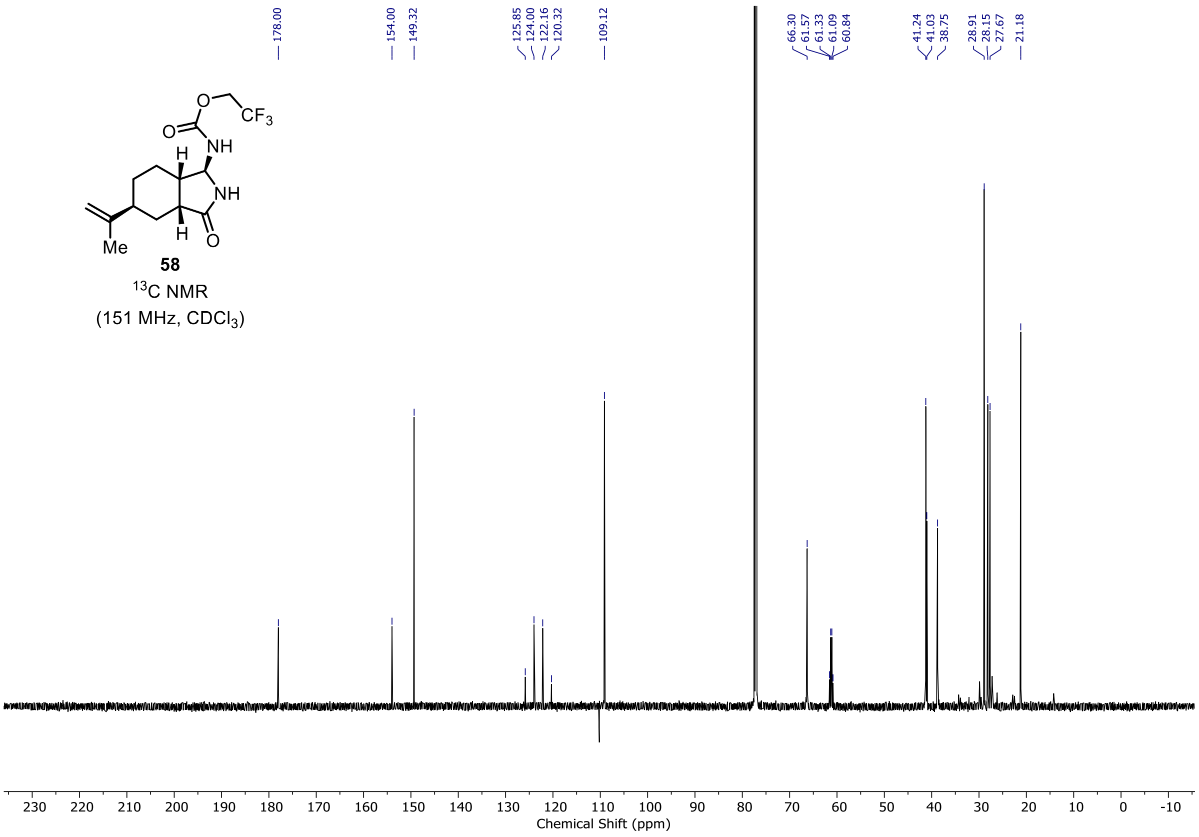
178.00
154.00
149.32
125.85
124.00
122.16
120.32
109.12
66.30
61.57
61.33
61.09
60.84
41.24
41.03
38.75
28.91
28.15
27.67
21.18
58
^{13}C NMR
(151 MHz, $CDCl_3$)
Chemical Shift (ppm)

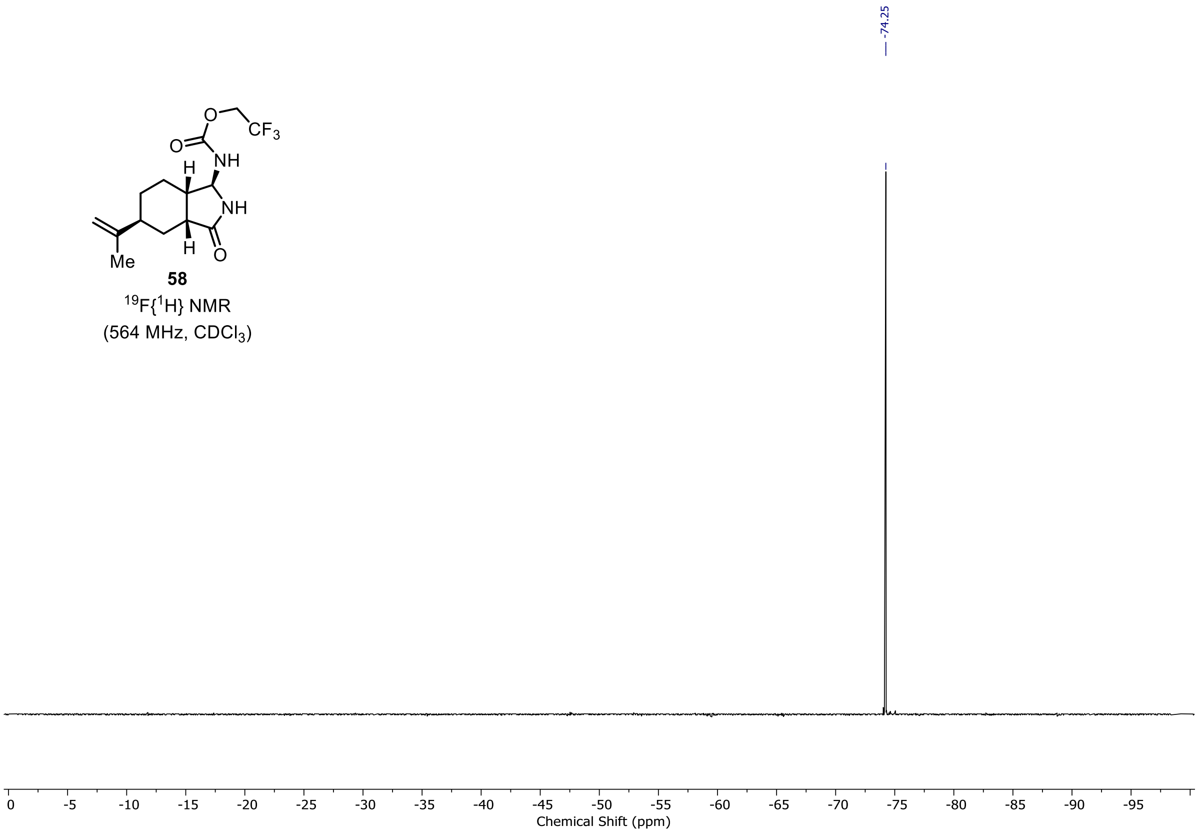
-74.25
58
$^{19}F\{^{1}H\}$ NMR
(564 MHz, $CDCl_3$)
0 -5 -10 -15 -20 -25 -30 -35 -40 -45 -50 -55 -60 -65 -70 -75 -80 -85 -90 -95
Chemical Shift (ppm)

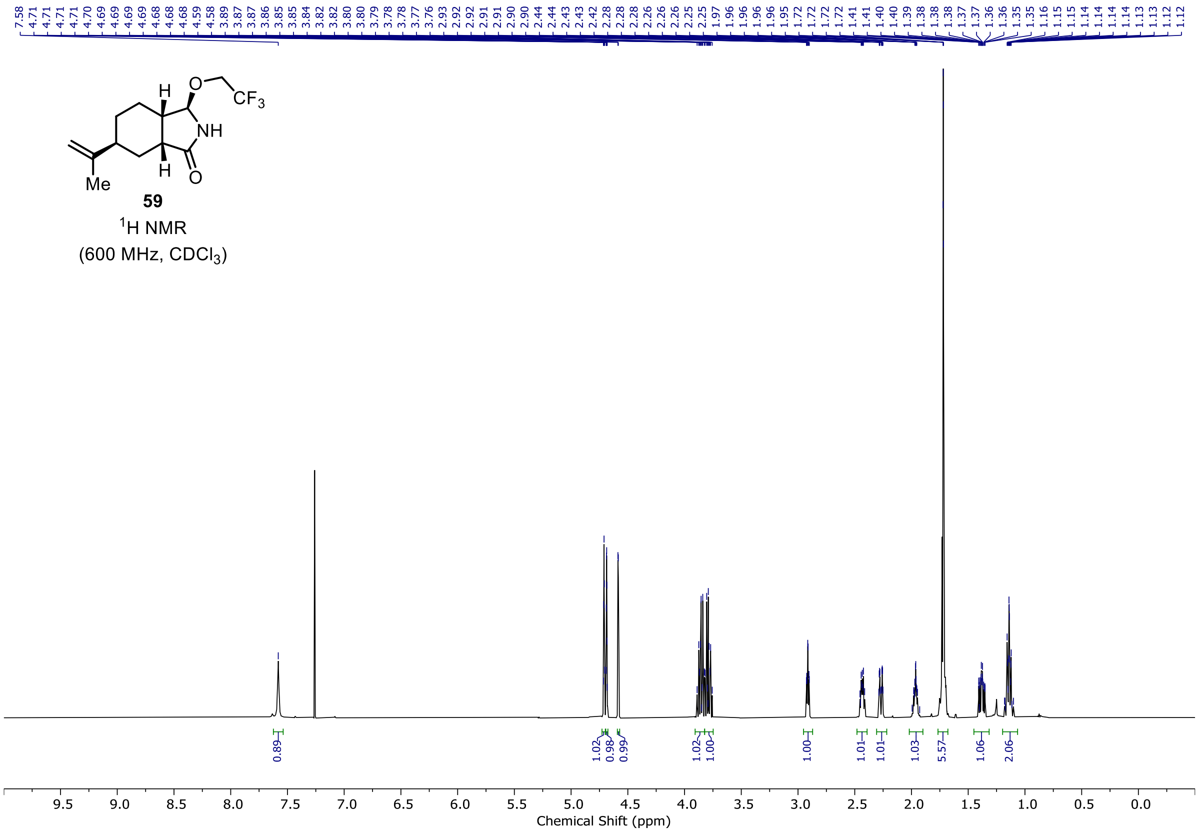

59
^1H NMR
(600 MHz, $CDCl_3$)
Chemical Shift (ppm)

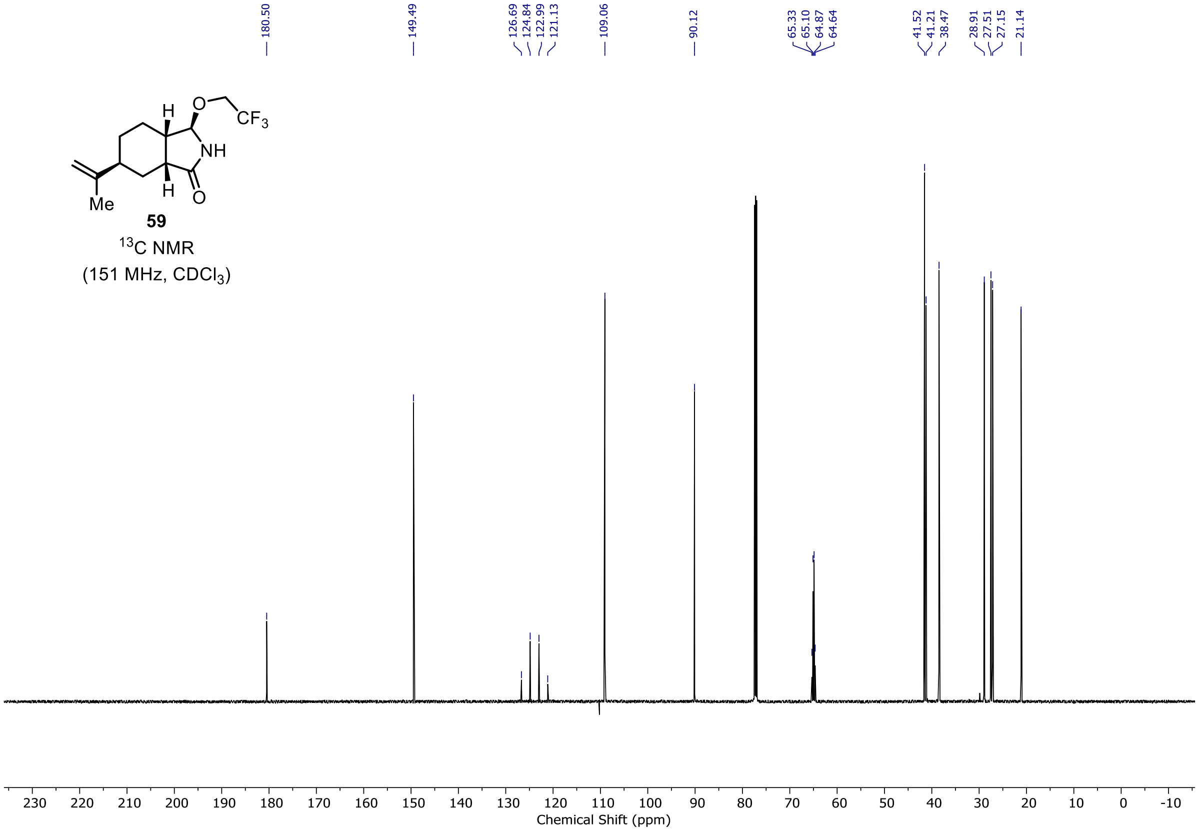

59
^{13}C NMR
(151 MHz, $CDCl_3$)
180.50
149.49
126.69
124.84
122.99
121.13
109.06
90.12
65.33
65.10
64.87
64.64
41.52
41.21
38.47
28.91
27.51
27.15
21.14
Chemical Shift (ppm)

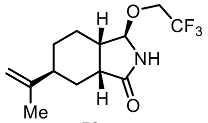

59

$^{19}F\{^1H\}$ NMR

(564 MHz, $CDCl_3$)

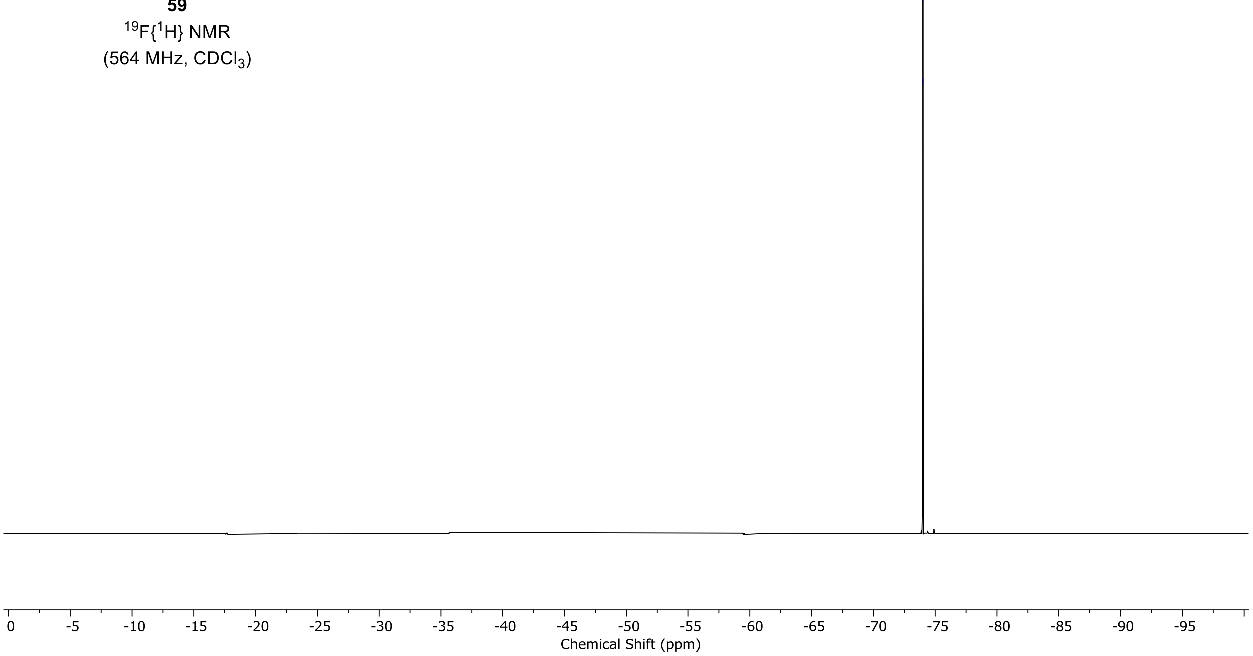

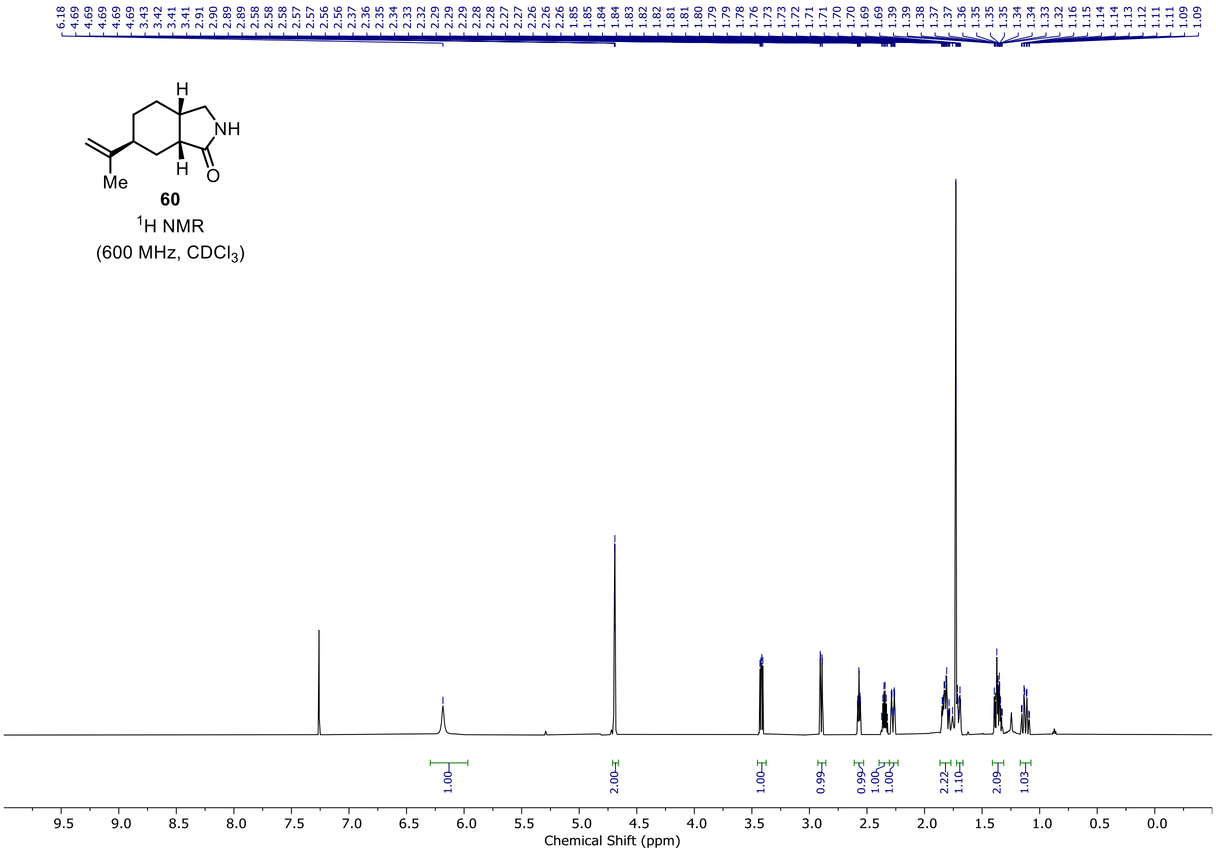
6.18 4.69 4.69 4.69 4.69 3.43 3.42 3.41 3.41 2.91 2.90 2.89 2.58 2.58 2.57 2.57 2.56 2.37 2.36 2.35 2.34 2.33 2.32 2.29 2.29 2.28 2.28 2.27 2.27 2.26 2.26 1.85 1.85 1.84 1.84 1.83 1.82 1.82 1.81 1.81 1.80 1.79 1.79 1.78 1.76 1.73 1.73 1.72 1.71 1.71 1.70 1.69 1.69 1.39 1.39 1.38 1.37 1.37 1.36 1.35 1.35 1.35 1.34 1.34 1.33 1.32 1.16 1.15 1.14 1.14 1.13 1.12 1.11 1.11 1.09 1.09
H
NH
Me
H
O
60
^{1}H NMR
(600 MHz, $CDCl_3$)
1.00 2.00 1.00 0.99 0.99 1.00 1.00 2.22 1.10 2.09 1.03
9.5 9.0 8.5 8.0 7.5 7.0 6.5 6.0 5.5 5.0 4.5 4.0 3.5 3.0 2.5 2.0 1.5 1.0 0.5 0.0
Chemical Shift (ppm)

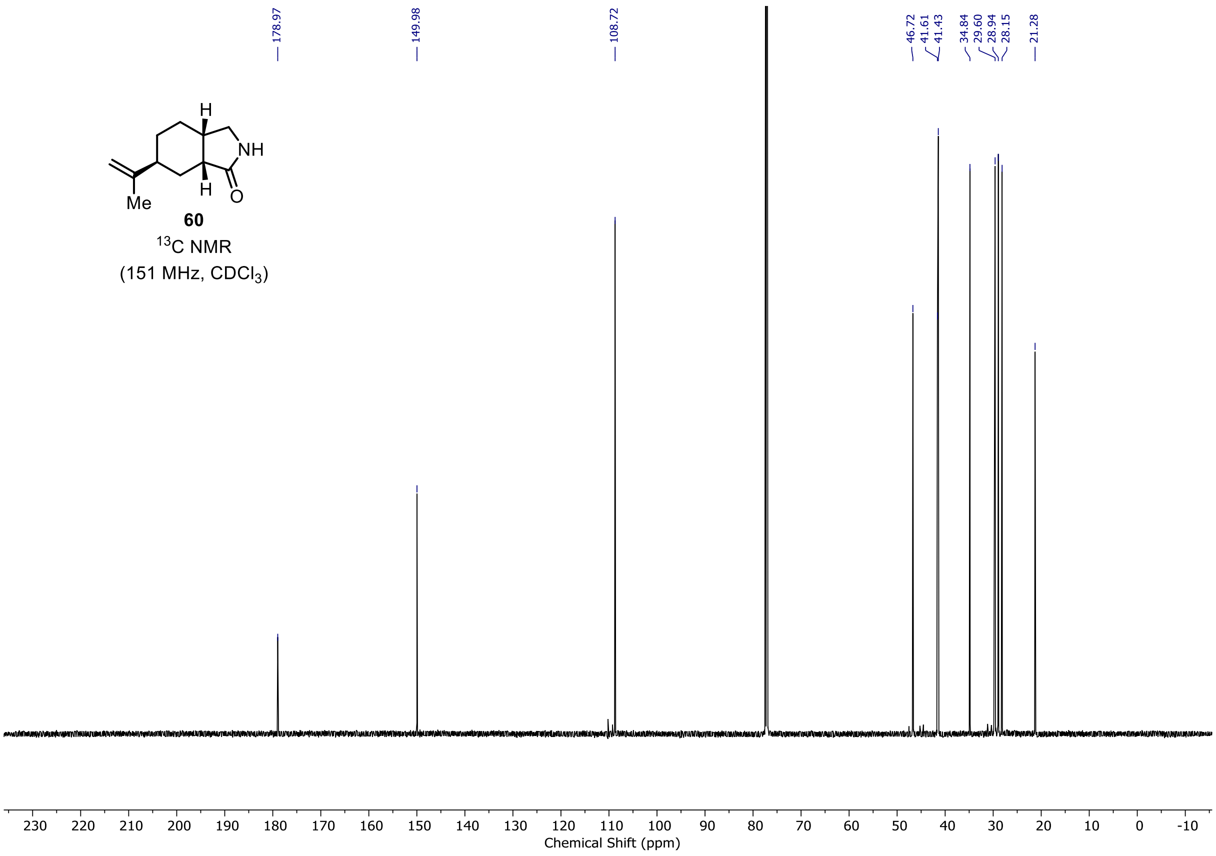
178.97
149.98
108.72
46.72 41.61 41.43 34.84 29.60 28.94 28.15 21.28
H
NH
Me
H
O
60
^{13}C NMR
(151 MHz, $CDCl_3$)
230 220 210 200 190 180 170 160 150 140 130 120 110 100 90 80 70 60 50 40 30 20 10 0 -10
Chemical Shift (ppm)

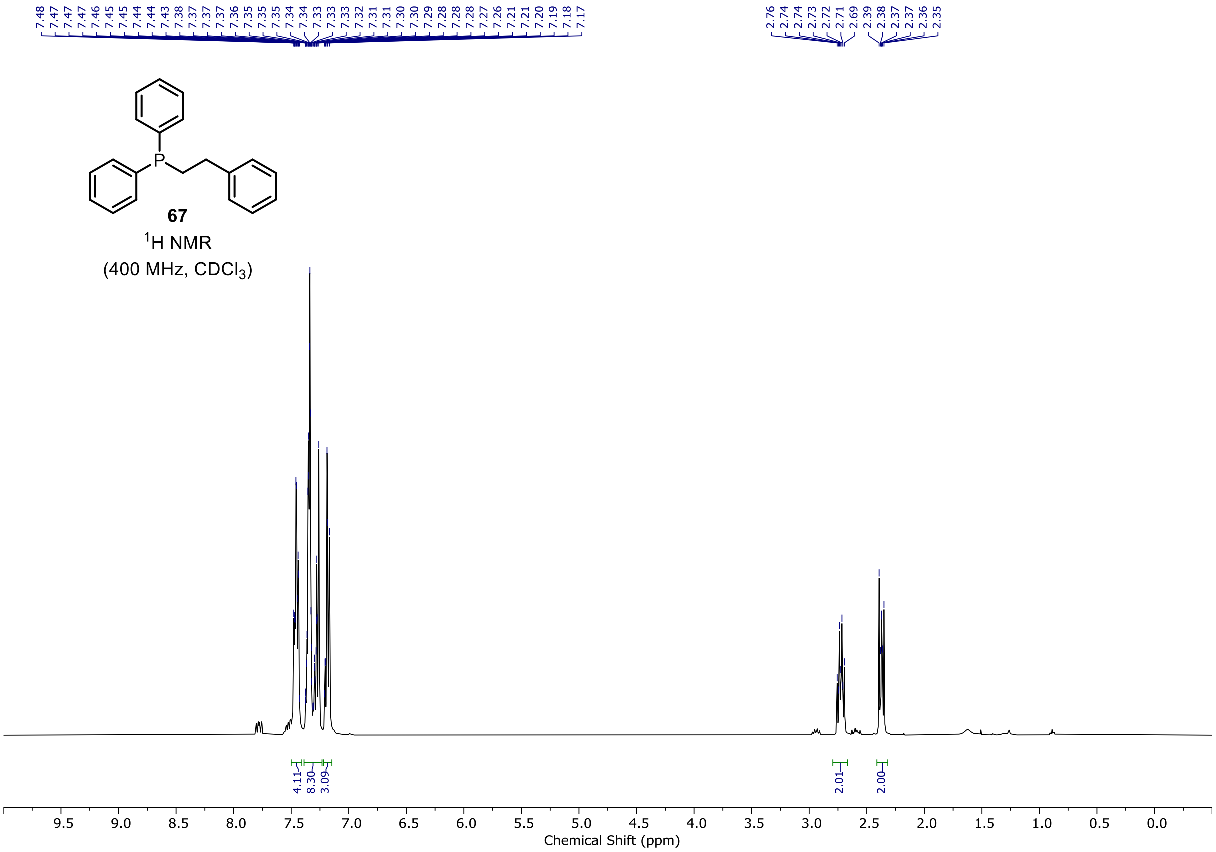
7.48
7.47
7.47
7.47
7.46
7.45
7.45
7.44
7.44
7.43
7.38
7.37
7.37
7.37
7.36
7.35
7.35
7.35
7.34
7.34
7.33
7.33
7.33
7.33
7.32
7.31
7.31
7.30
7.30
7.29
7.28
7.28
7.28
7.27
7.26
7.21
7.21
7.20
7.19
7.18
7.17
2.76
2.74
2.74
2.73
2.72
2.72
2.71
2.69
2.39
2.38
2.38
2.37
2.37
2.37
2.36
2.35
67
^{1}H NMR
(400 MHz, CDCl$_3$)
4.11
8.30
3.09
2.01
2.00
9.5
9.0
8.5
8.0
7.5
7.0
6.5
6.0
5.5
5.0
4.5
4.0
3.5
3.0
2.5
2.0
1.5
1.0
0.5
0.0
Chemical Shift (ppm)

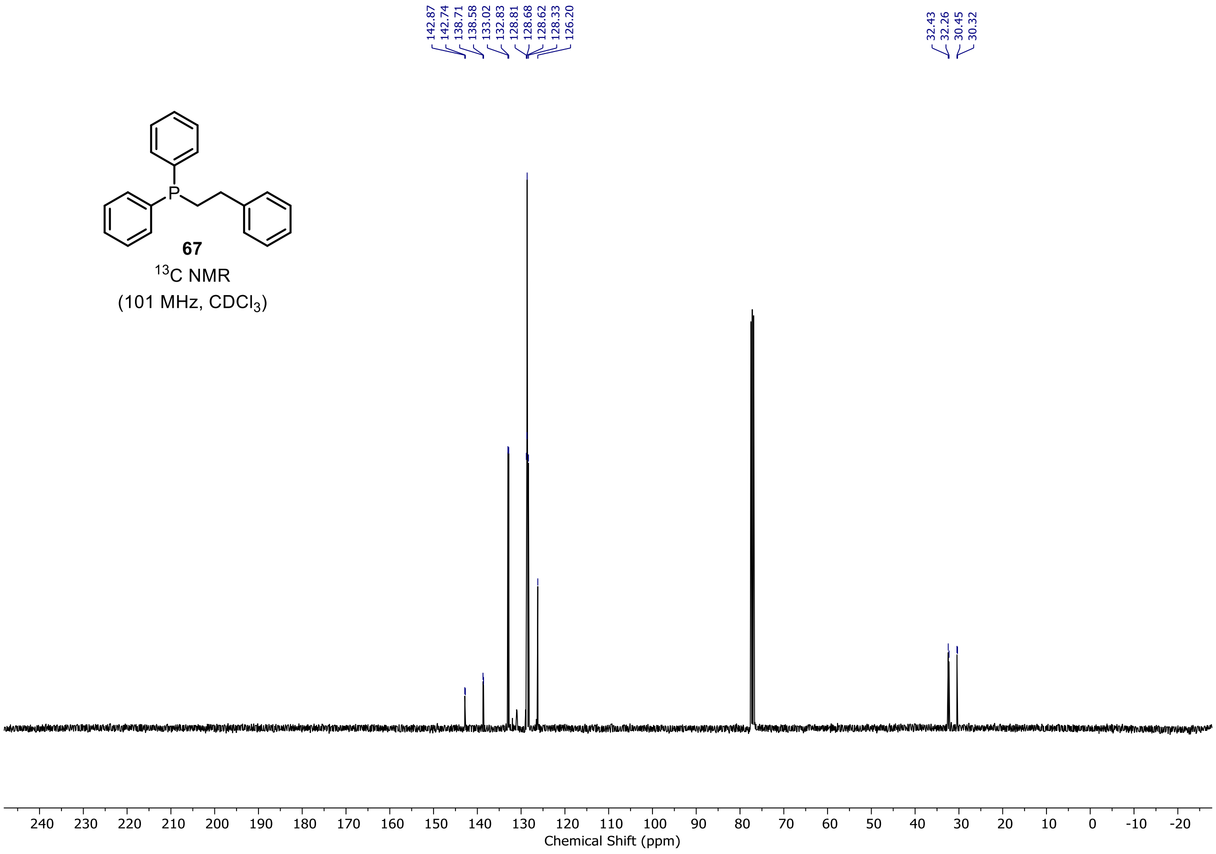
142.87
142.74
138.71
138.58
133.02
132.83
128.81
128.68
128.62
128.33
126.20
32.43
32.26
30.45
30.32
67
^{13}C NMR
(101 MHz, CDCl$_3$)
240
230
220
210
200
190
180
170
160
150
140
130
120
110
100
90
80
70
60
50
40
30
20
10
0
-10
-20
Chemical Shift (ppm)

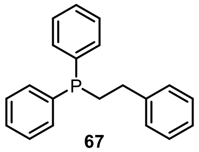

67
^{31}P NMR
(162 MHz, $CDCl_3$)

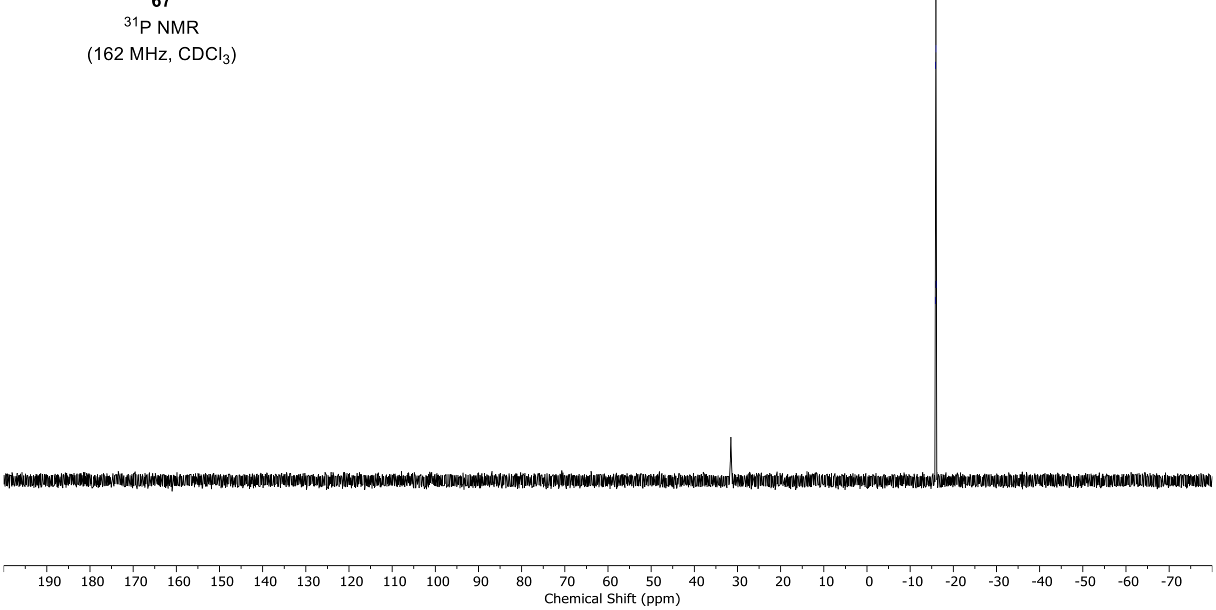

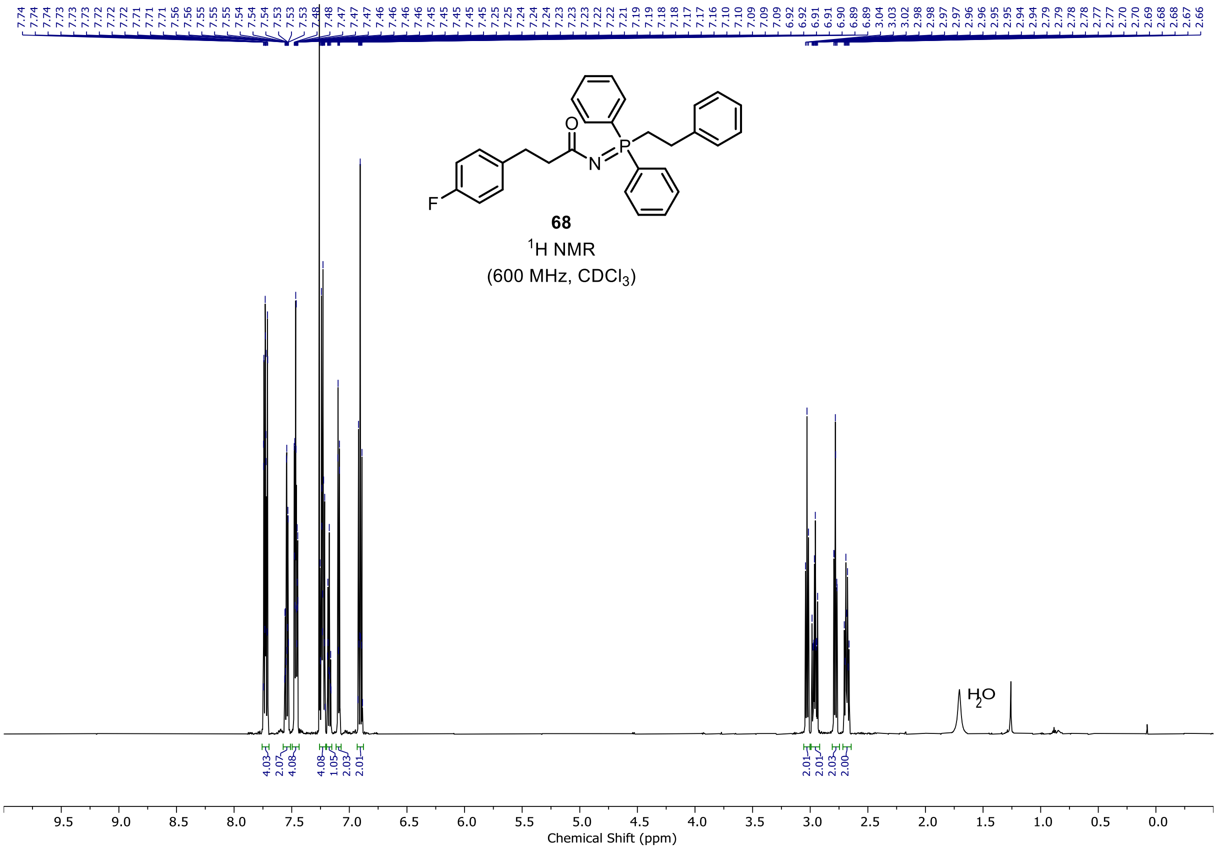
68
^{1}H NMR
(600 MHz, $CDCl_3$)
H_2O
Chemical Shift (ppm)

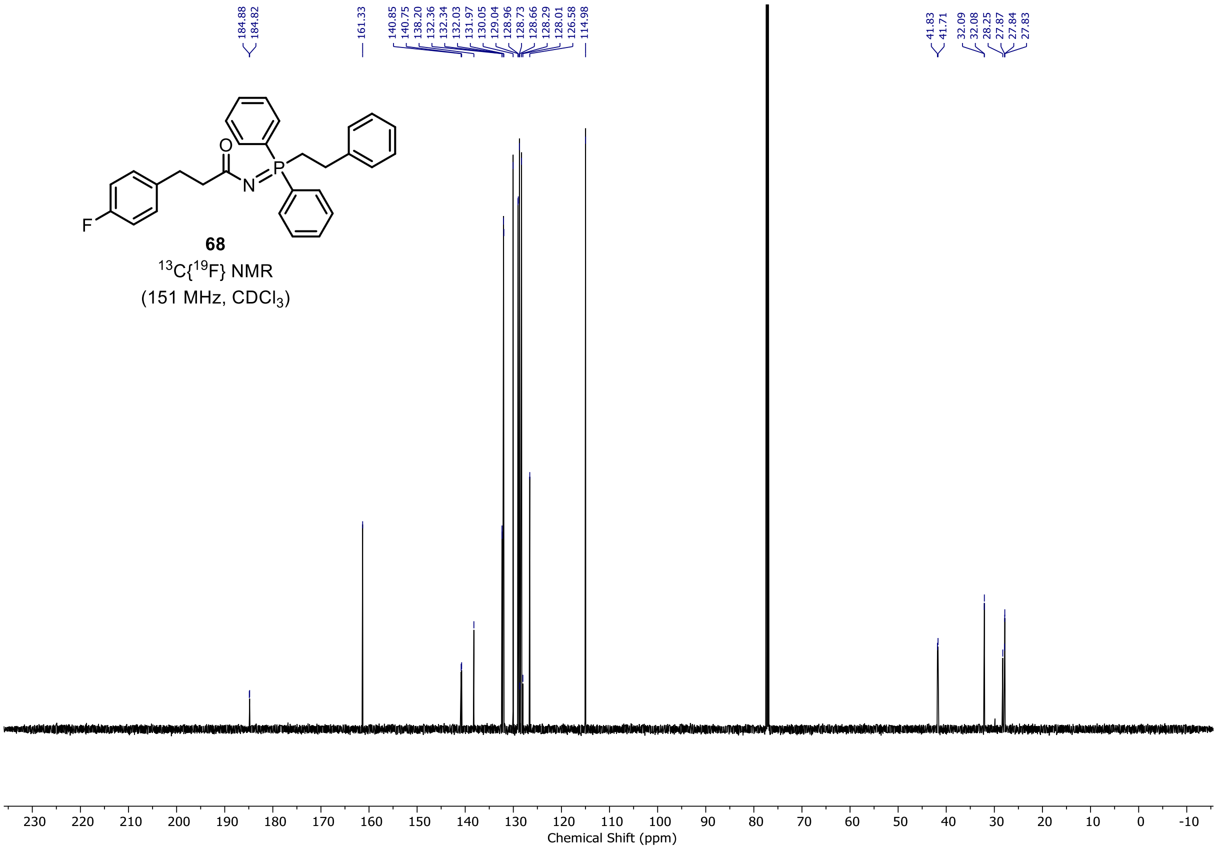
68
$^{13}C\{^{19}F\}$ NMR
(151 MHz, $CDCl_3$)
184.88
184.82
161.33
140.85
140.75
138.20
132.36
132.34
132.03
131.97
130.05
129.04
128.96
128.73
128.66
128.29
128.01
126.58
114.98
41.83
41.71
32.09
32.08
28.25
27.87
27.84
27.83
Chemical Shift (ppm)

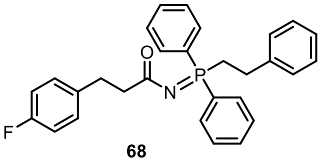

68
$^{19}F\{^1H\}$ NMR
(564 MHz, $CDCl_3$)

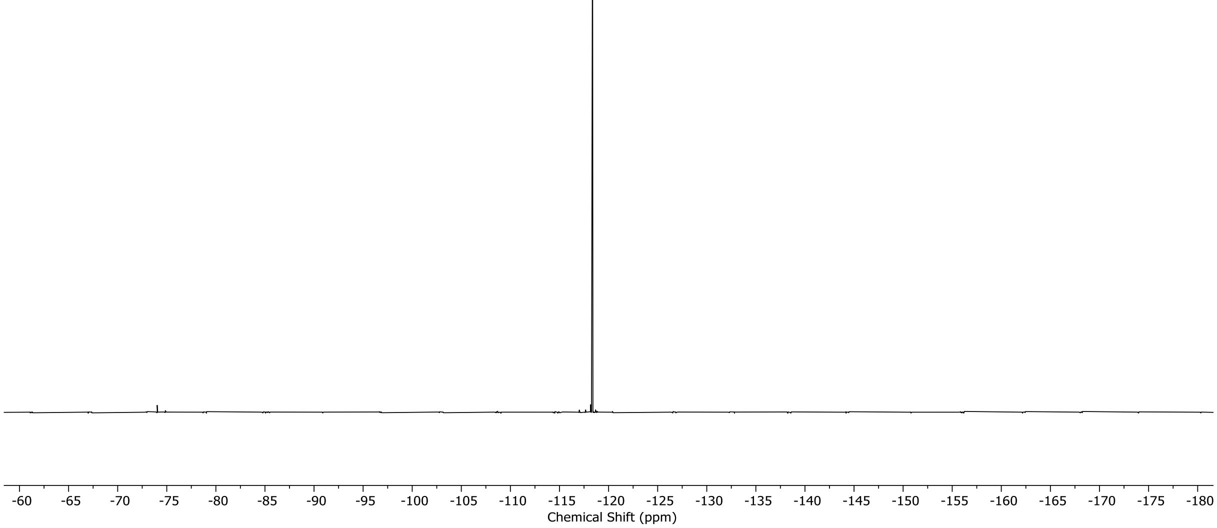

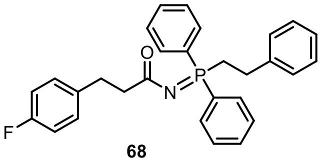

68
^{31}P NMR
(242 MHz, $CDCl_3$)

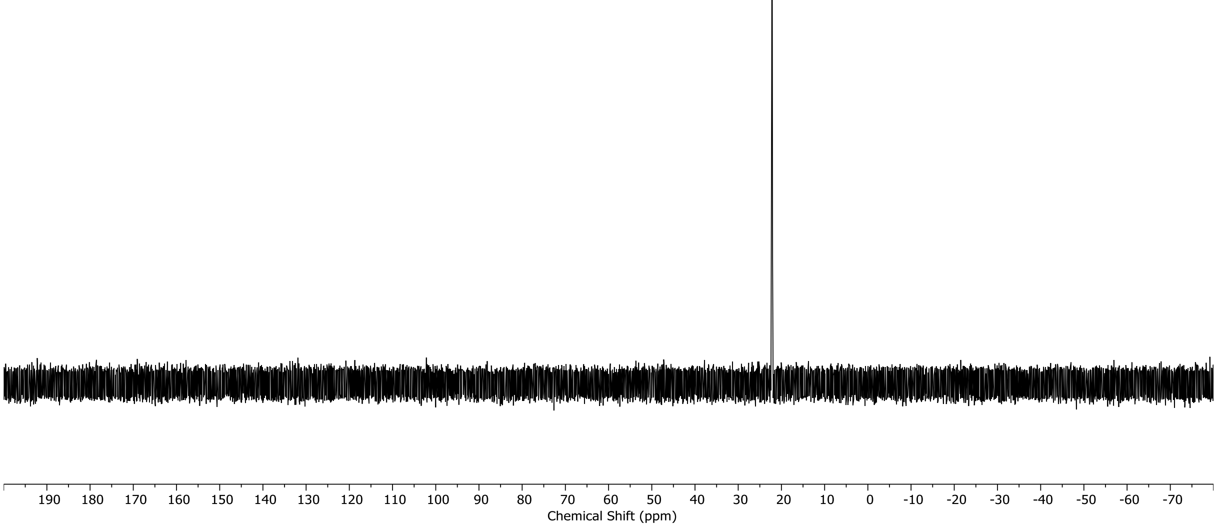

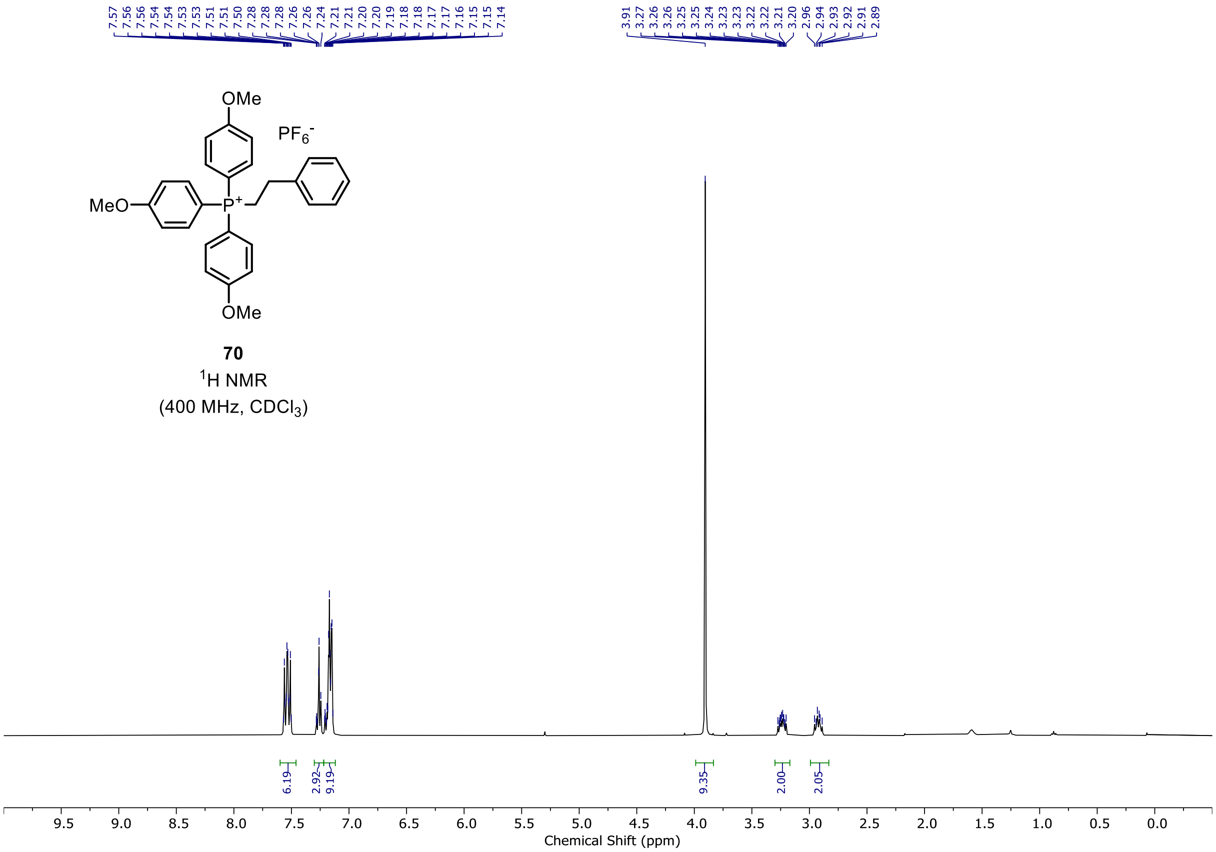

7.57
7.56
7.56
7.54
7.54
7.53
7.53
7.51
7.51
7.50
7.28
7.28
7.28
7.26
7.26
7.24
7.21
7.21
7.20
7.20
7.19
7.18
7.18
7.17
7.17
7.16
7.15
7.15
7.14
3.91
3.27
3.26
3.26
3.25
3.25
3.24
3.23
3.23
3.22
3.22
3.21
3.20
2.96
2.94
2.93
2.92
2.91
2.89
OMe
PF6-
MeO
P+
OMe
70
1H NMR
(400 MHz, CDCl3)
6.19
2.92
9.19
9.35
2.00
2.05
Chemical Shift (ppm)

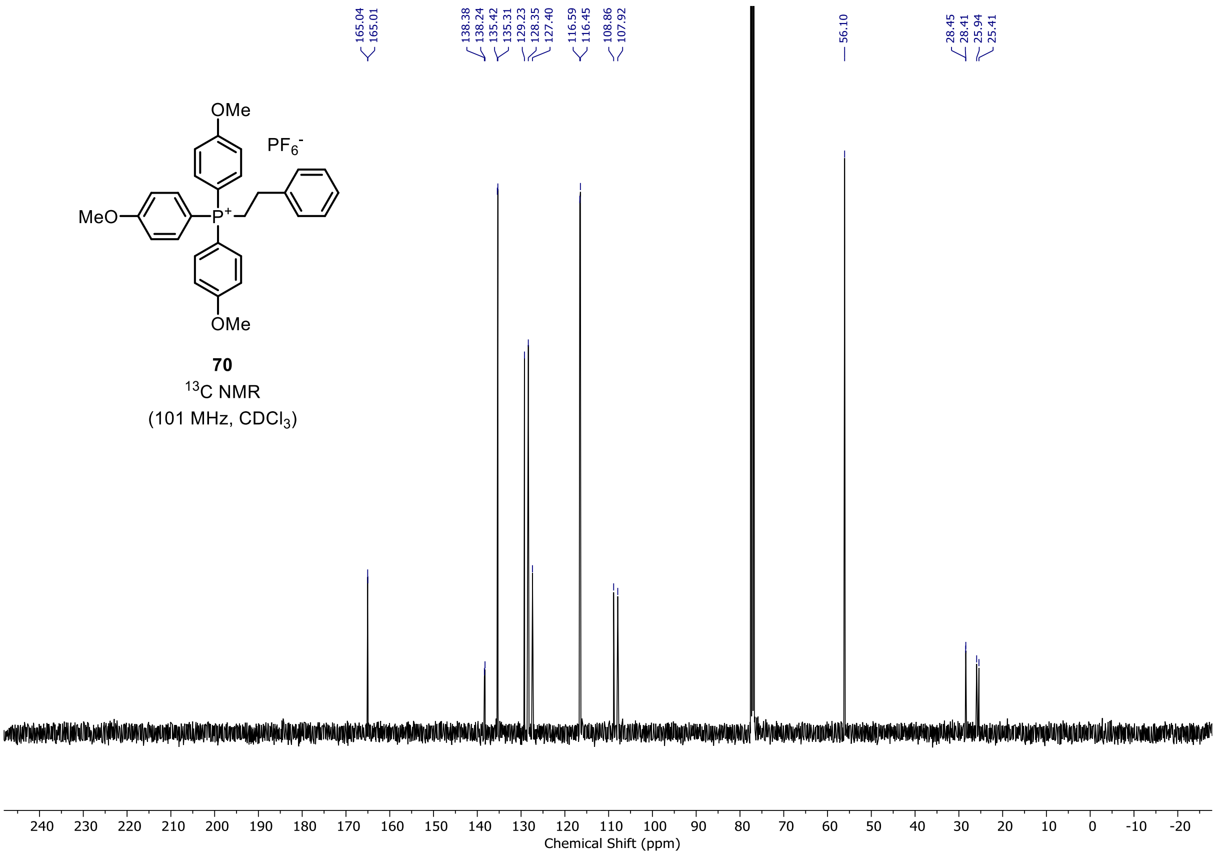

165.04
165.01
138.38
138.24
135.42
135.31
129.23
128.35
127.40
116.59
116.45
108.86
107.92
56.10
28.45
28.41
25.94
25.41
OMe
PF6-
MeO
P+
OMe
70
13C NMR
(101 MHz, CDCl3)
Chemical Shift (ppm)

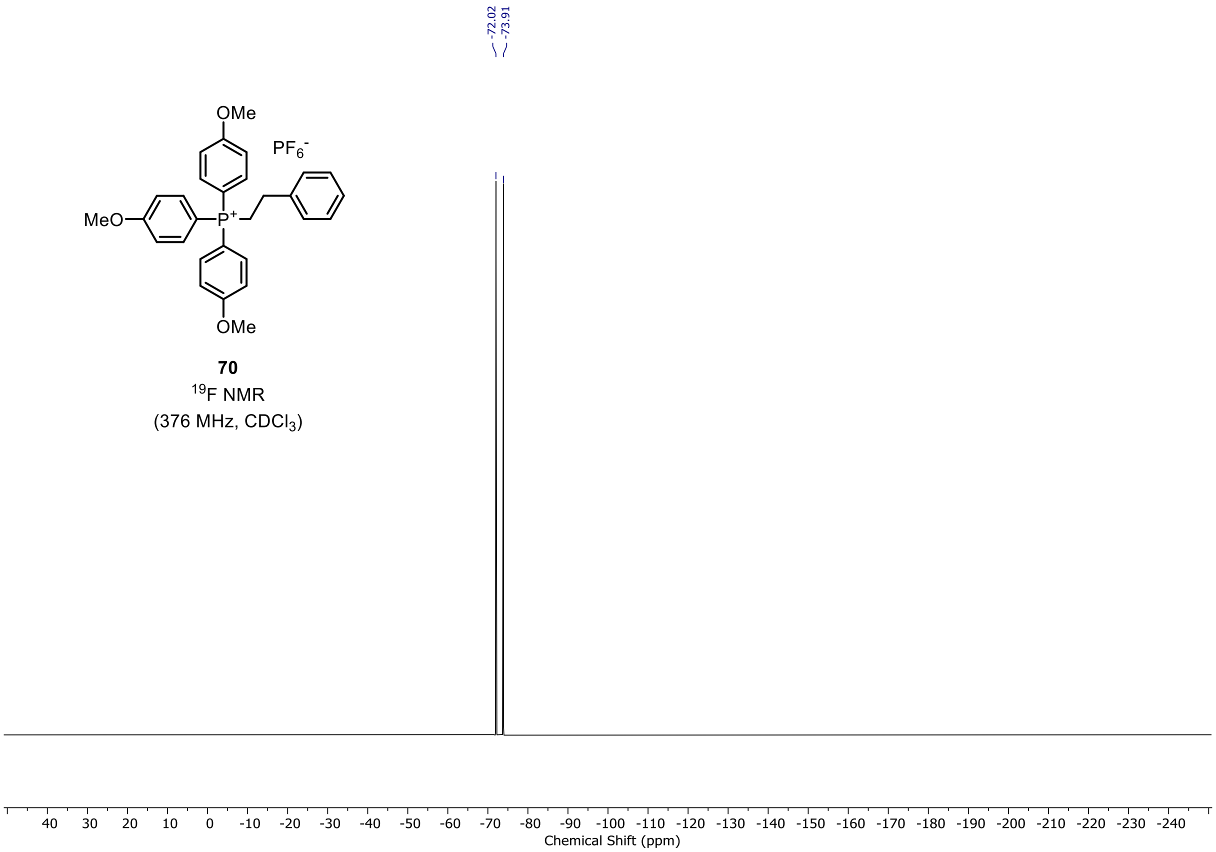

-72.02
-73.91
OMe
PF6-
MeO
P+
OMe
70
19F NMR
(376 MHz, CDCl3)
40 30 20 10 0 -10 -20 -30 -40 -50 -60 -70 -80 -90 -100 -110 -120 -130 -140 -150 -160 -170 -180 -190 -200 -210 -220 -230 -240
Chemical Shift (ppm)

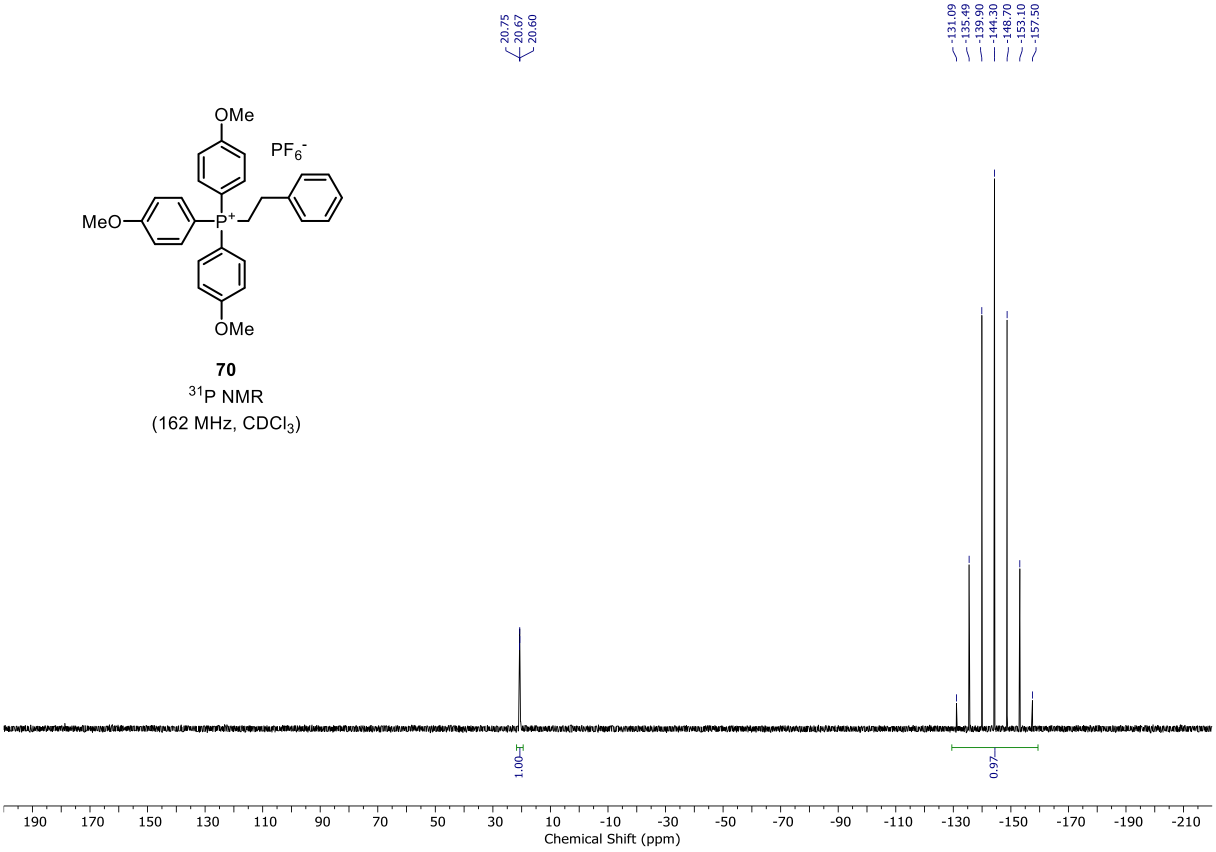

20.75
20.67
20.60
-131.09
-135.49
-139.90
-144.30
-148.70
-153.10
-157.50
OMe
PF6-
MeO
P+
OMe
70
31P NMR
(162 MHz, CDCl3)
1.00
0.97
190 170 150 130 110 90 70 50 30 10 -10 -30 -50 -70 -90 -110 -130 -150 -170 -190 -210
Chemical Shift (ppm)

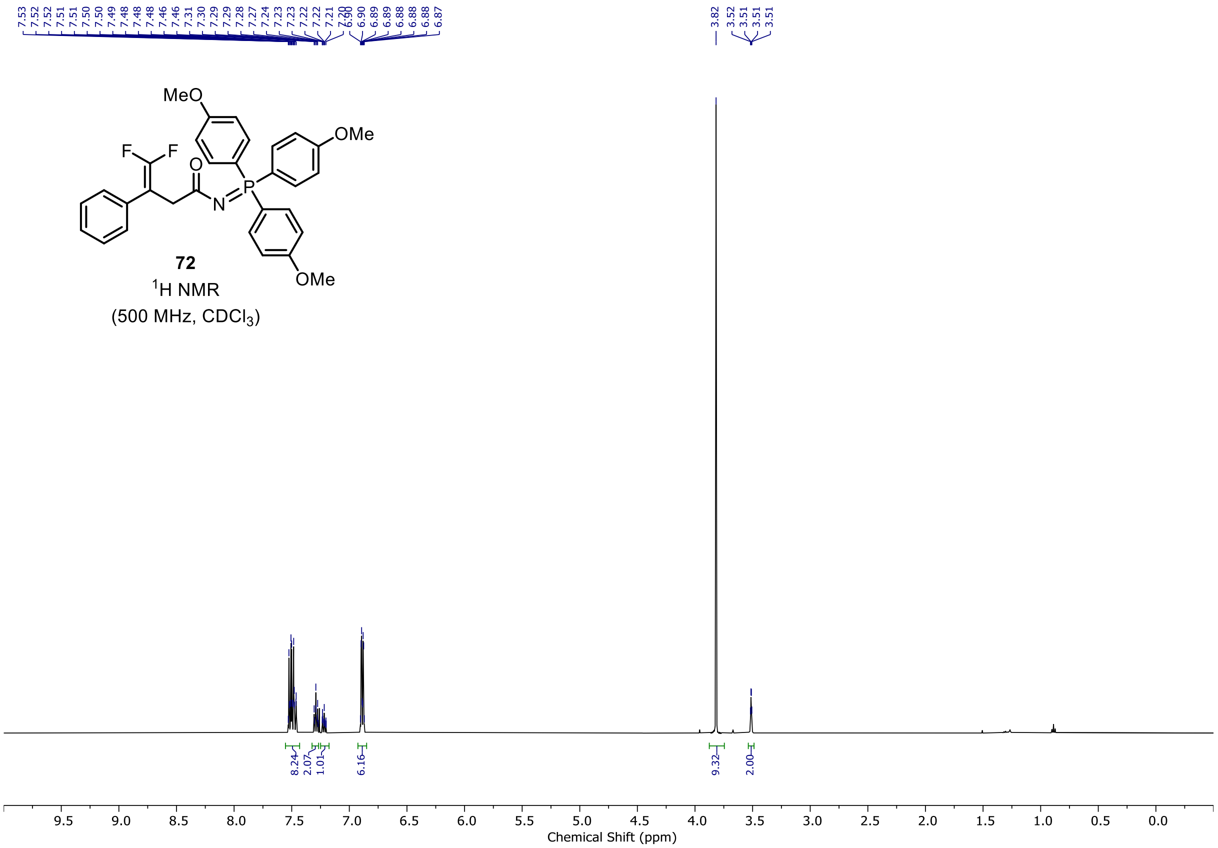
7.53 7.52 7.52 7.51 7.51 7.50 7.50 7.49 7.48 7.48 7.48 7.46 7.46 7.31 7.30 7.29 7.29 7.28 7.27 7.24 7.23 7.23 7.22 7.22 7.21 7.21 6.90 6.90 6.89 6.89 6.88 6.88 6.87
3.82 3.52 3.51 3.51 3.51
MeO
OMe
F F
O
N
P
OMe
72
1H NMR
(500 MHz, CDCl3)
8.24 2.07 1.01 6.16 9.32 2.00
9.5 9.0 8.5 8.0 7.5 7.0 6.5 6.0 5.5 5.0 4.5 4.0 3.5 3.0 2.5 2.0 1.5 1.0 0.5 0.0
Chemical Shift (ppm)

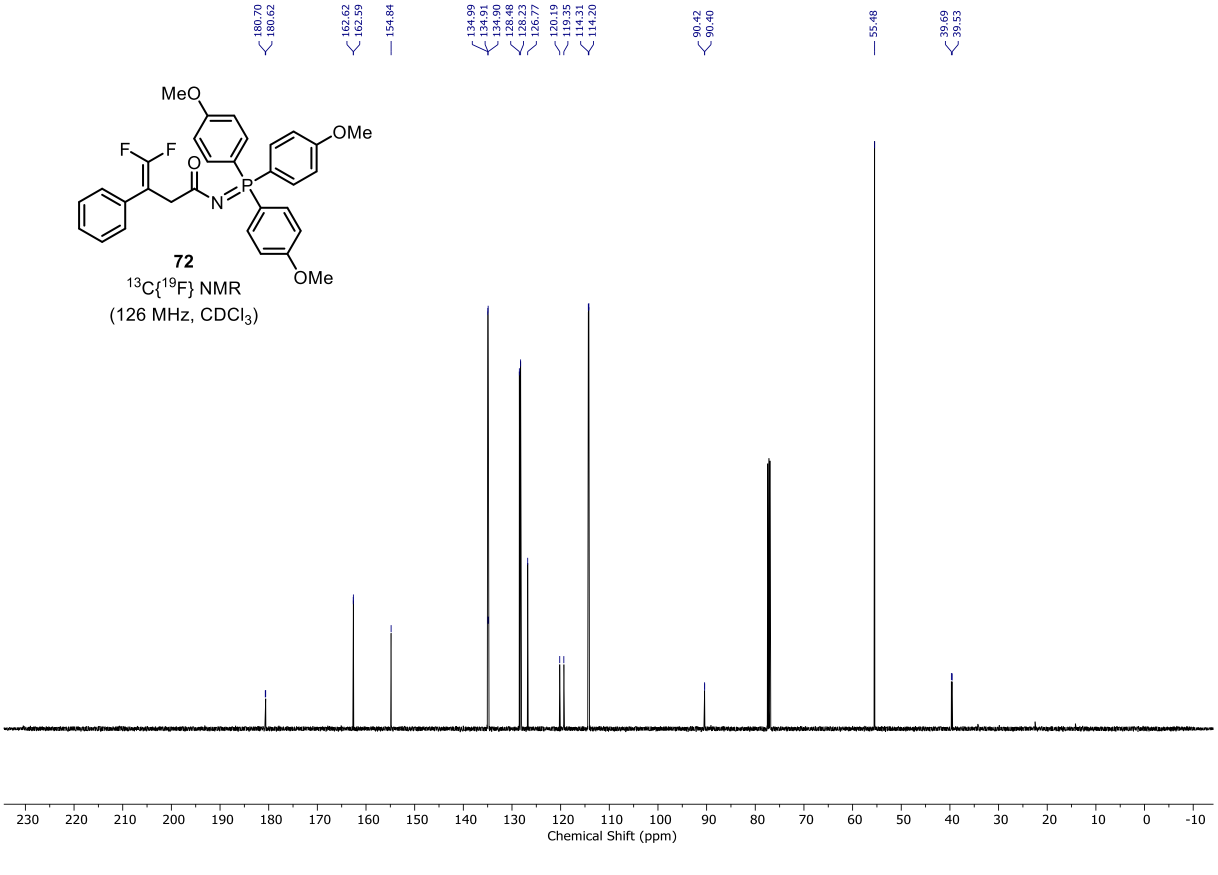
180.70 180.62 162.62 162.59 154.84 134.99 134.91 134.90 128.48 128.23 126.77 120.19 119.35 114.31 114.20 90.42 90.40 55.48 39.69 39.53
MeO
OMe
F F
O
N
P
OMe
72
13C{19F} NMR
(126 MHz, CDCl3)
230 220 210 200 190 180 170 160 150 140 130 120 110 100 90 80 70 60 50 40 30 20 10 0 -10
Chemical Shift (ppm)

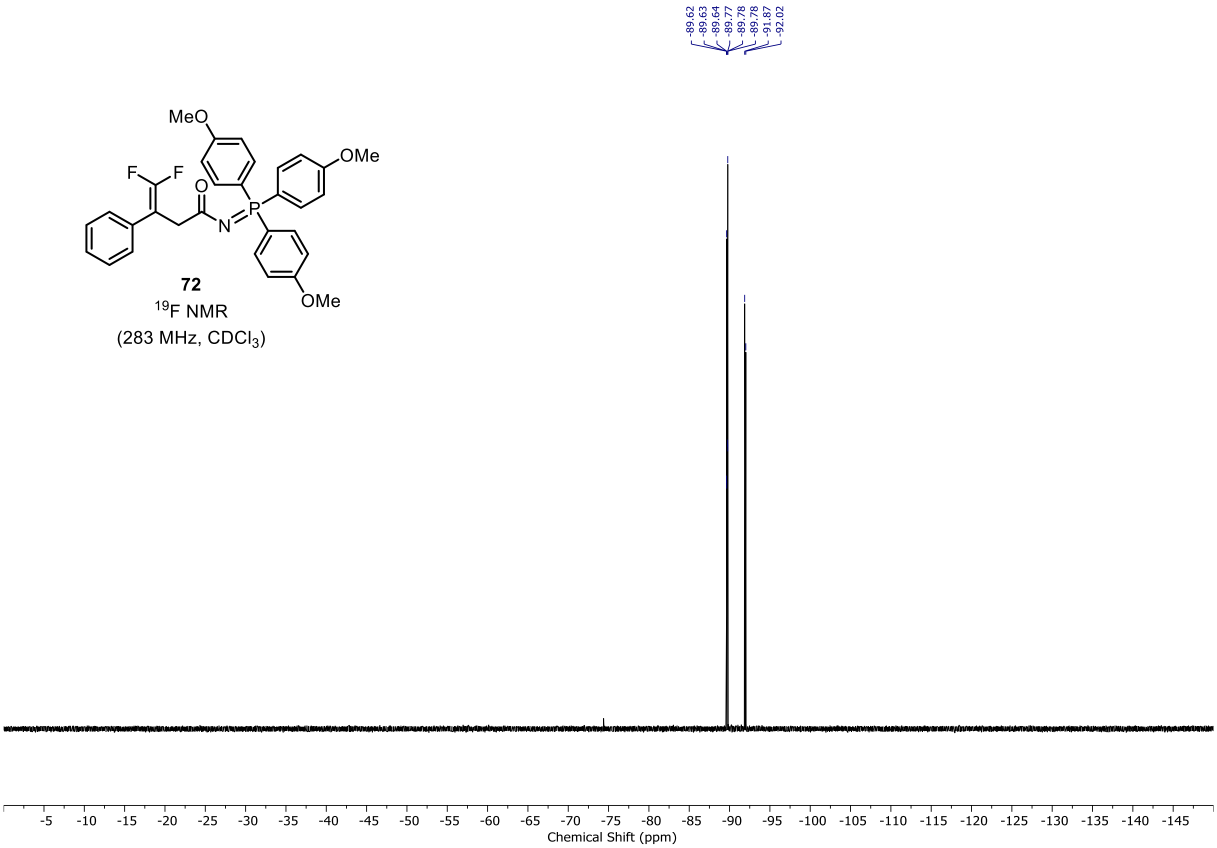

72

^{19}F NMR

(283 MHz, $CDCl_3$)

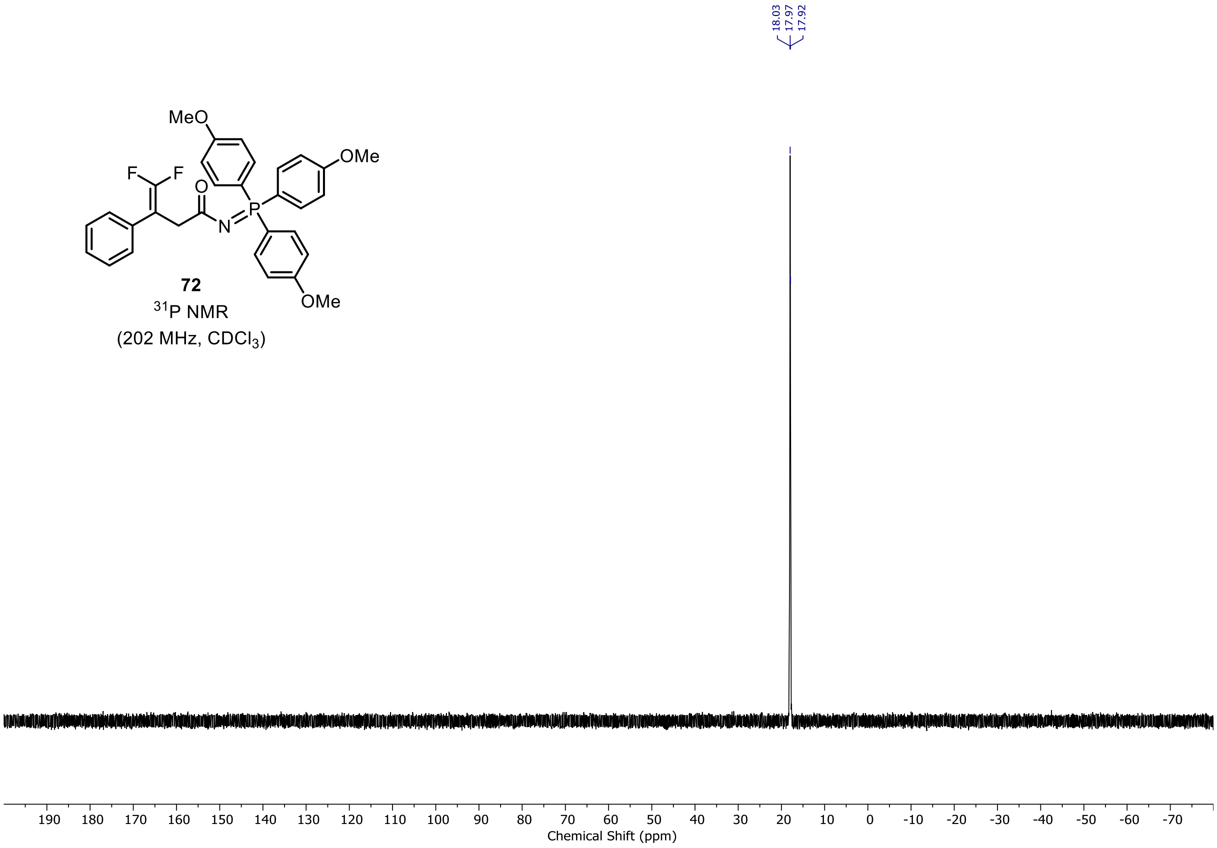

72

^{31}P NMR

(202 MHz, $CDCl_3$)

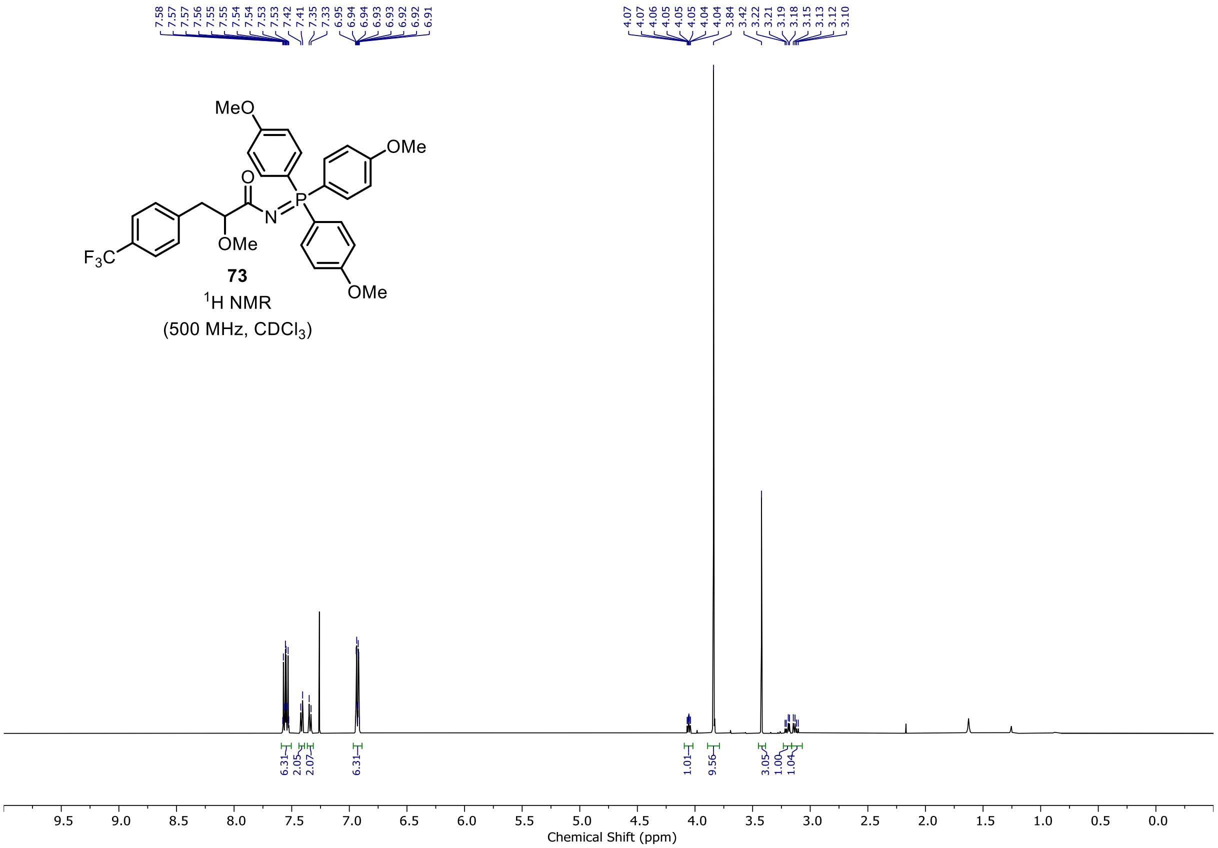
MeO
OMe
O
N
P
F3C
OMe
OMe
73
1H NMR
(500 MHz, CDCl3)
Chemical Shift (ppm)

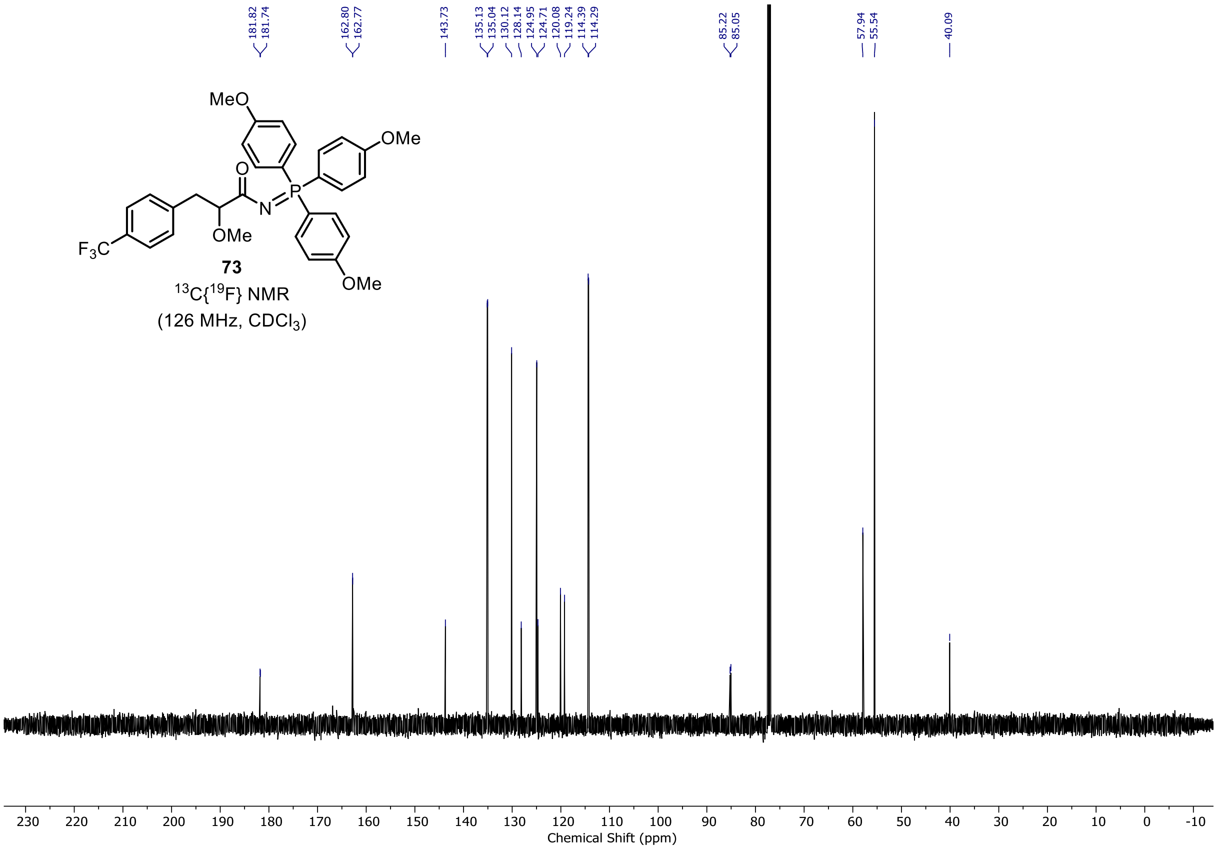
MeO
OMe
O
N
P
F3C
OMe
OMe
73
13C{19F} NMR
(126 MHz, CDCl3)
Chemical Shift (ppm)

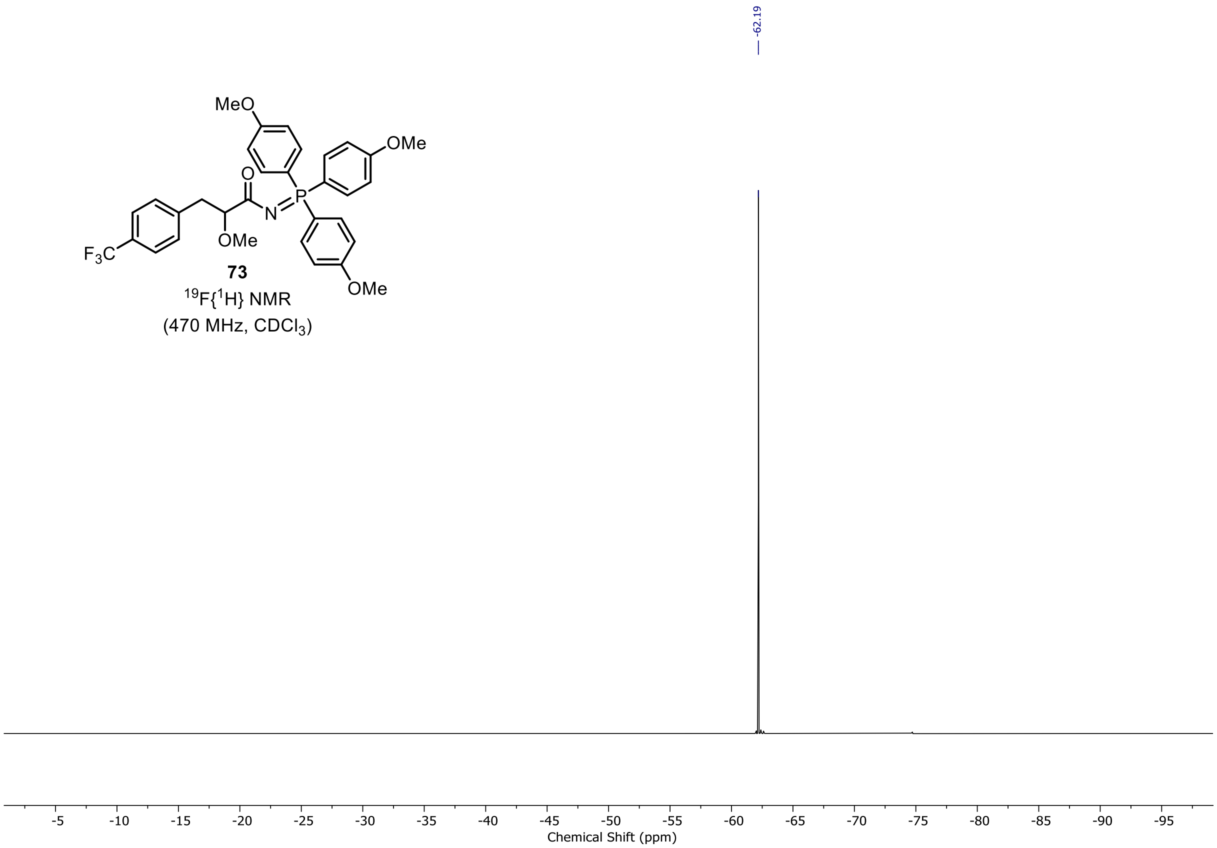
-62.19
MeO
OMe
O
N
P
OMe
F3C
OMe
73
19F{1H} NMR
(470 MHz, CDCl3)
-5 -10 -15 -20 -25 -30 -35 -40 -45 -50 -55 -60 -65 -70 -75 -80 -85 -90 -95
Chemical Shift (ppm)

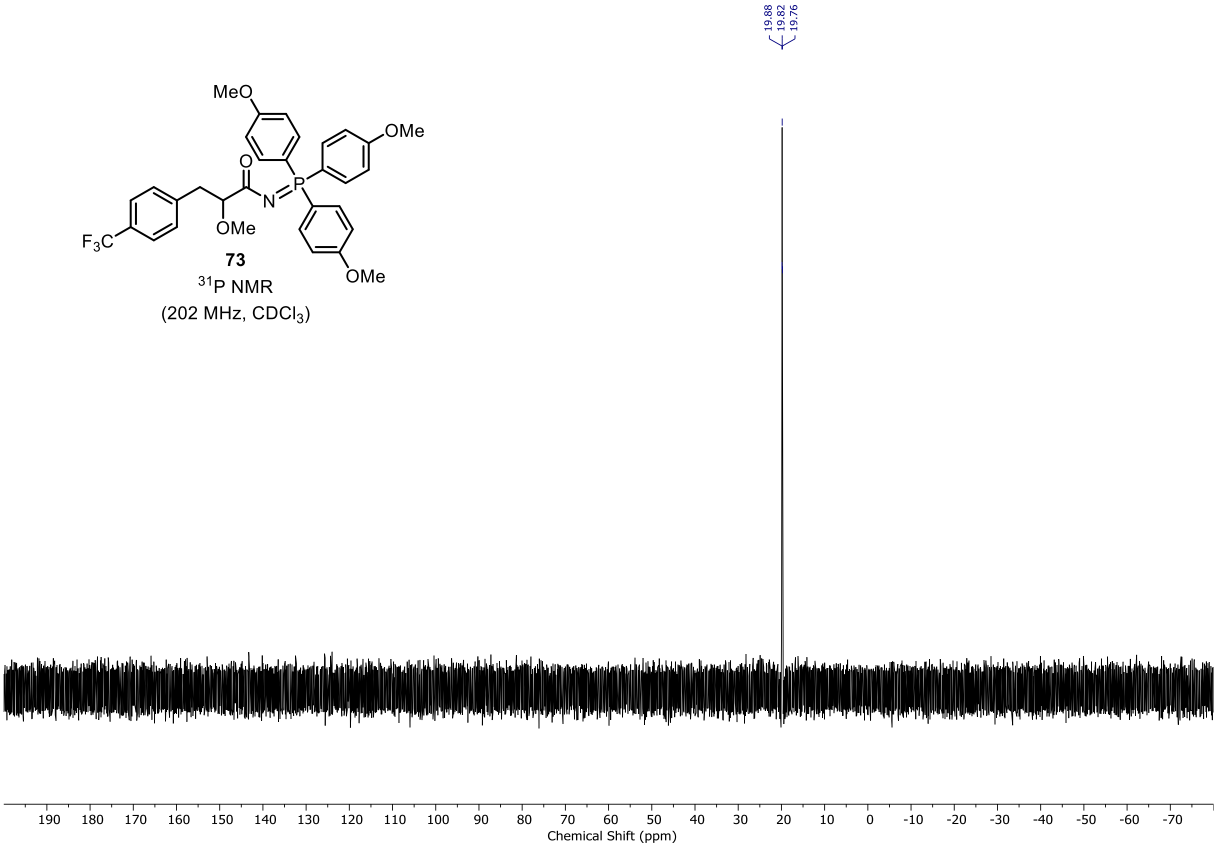
19.88
19.82
19.76
MeO
OMe
O
N
P
OMe
F3C
OMe
73
31P NMR
(202 MHz, CDCl3)
190 180 170 160 150 140 130 120 110 100 90 80 70 60 50 40 30 20 10 0 -10 -20 -30 -40 -50 -60 -70
Chemical Shift (ppm)

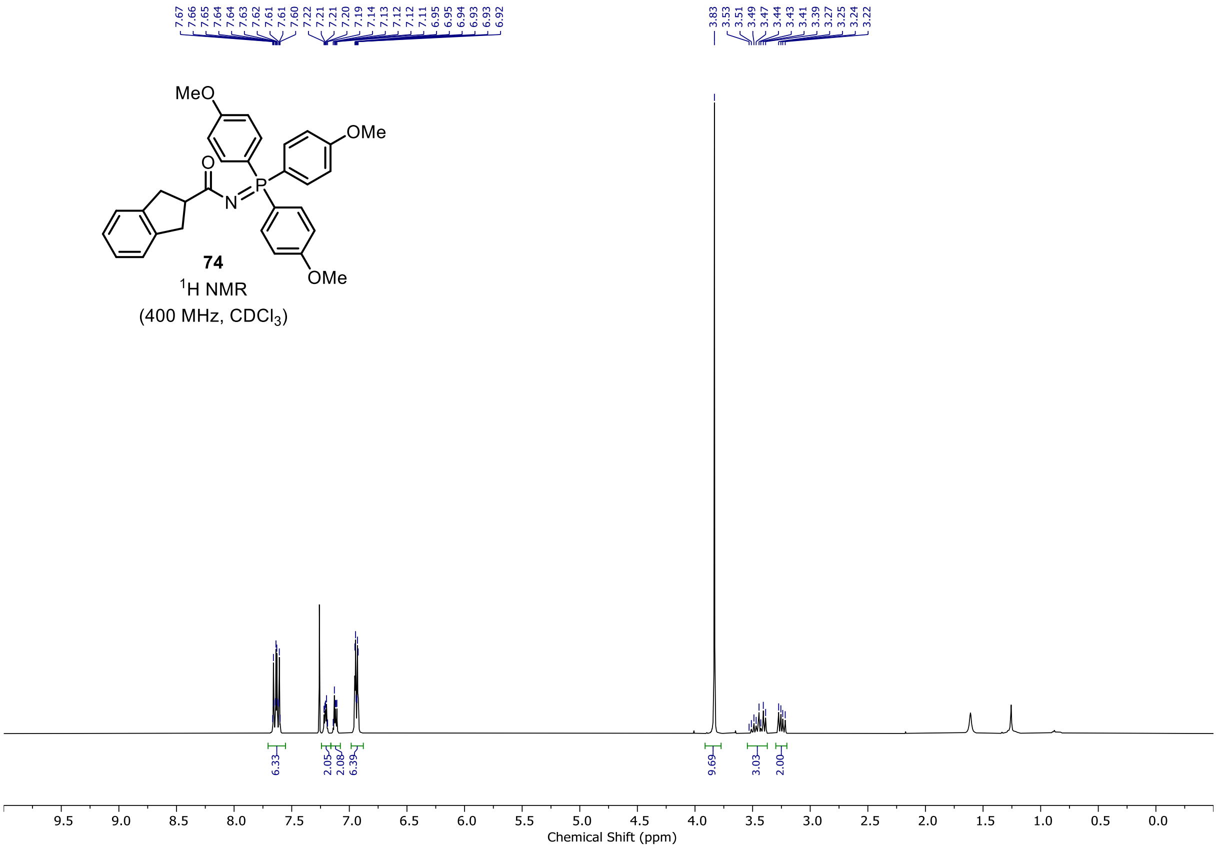
7.67
7.66
7.65
7.64
7.64
7.63
7.62
7.61
7.61
7.60
7.22
7.21
7.21
7.20
7.19
7.14
7.13
7.12
7.12
7.11
6.95
6.95
6.94
6.93
6.93
6.92
3.83
3.53
3.51
3.49
3.47
3.44
3.43
3.41
3.39
3.27
3.25
3.24
3.22
MeO
OMe
O
N
P
OMe
74
^{1}H NMR
(400 MHz, $CDCl_3$)
6.33
2.05
2.08
6.39
9.69
3.03
2.00
Chemical Shift (ppm)

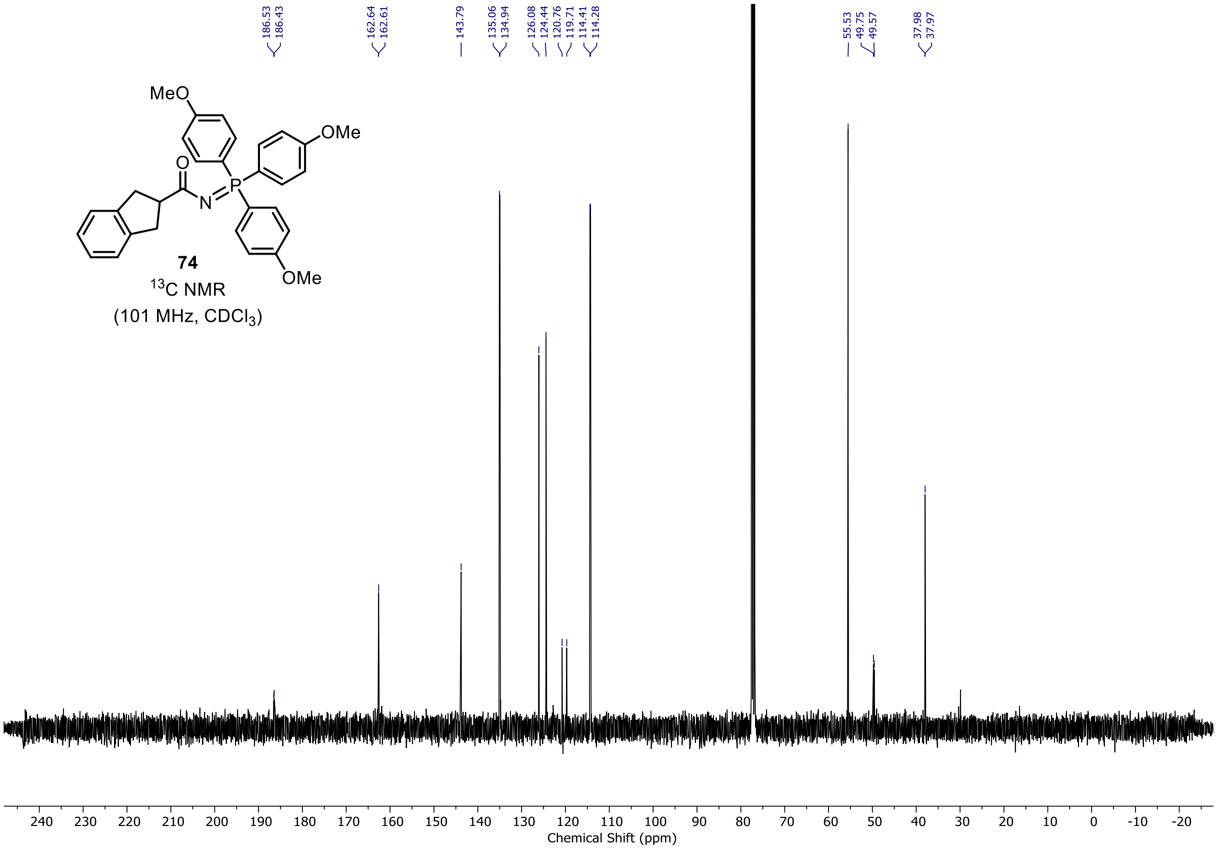
186.53
186.43
162.64
162.61
143.79
135.06
134.94
126.08
124.44
120.76
119.71
114.41
114.28
55.53
49.75
49.57
37.98
37.97
MeO
OMe
O
N
P
OMe
74
^{13}C NMR
(101 MHz, $CDCl_3$)
Chemical Shift (ppm)

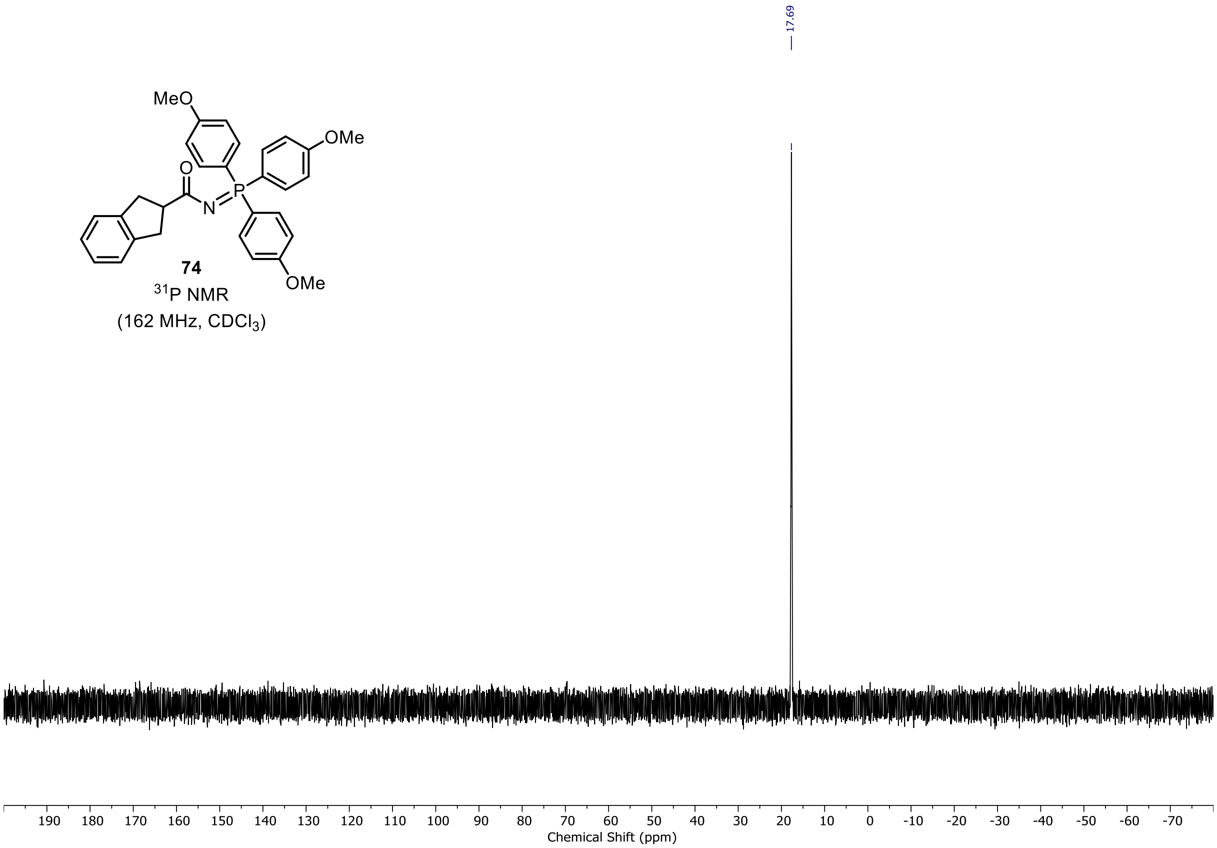
MeO
OMe
OMe
O
N
P
74
^{31}P NMR
(162 MHz, $CDCl_3$)
17.69
190 180 170 160 150 140 130 120 110 100 90 80 70 60 50 40 30 20 10 0 -10 -20 -30 -40 -50 -60 -70
Chemical Shift (ppm)

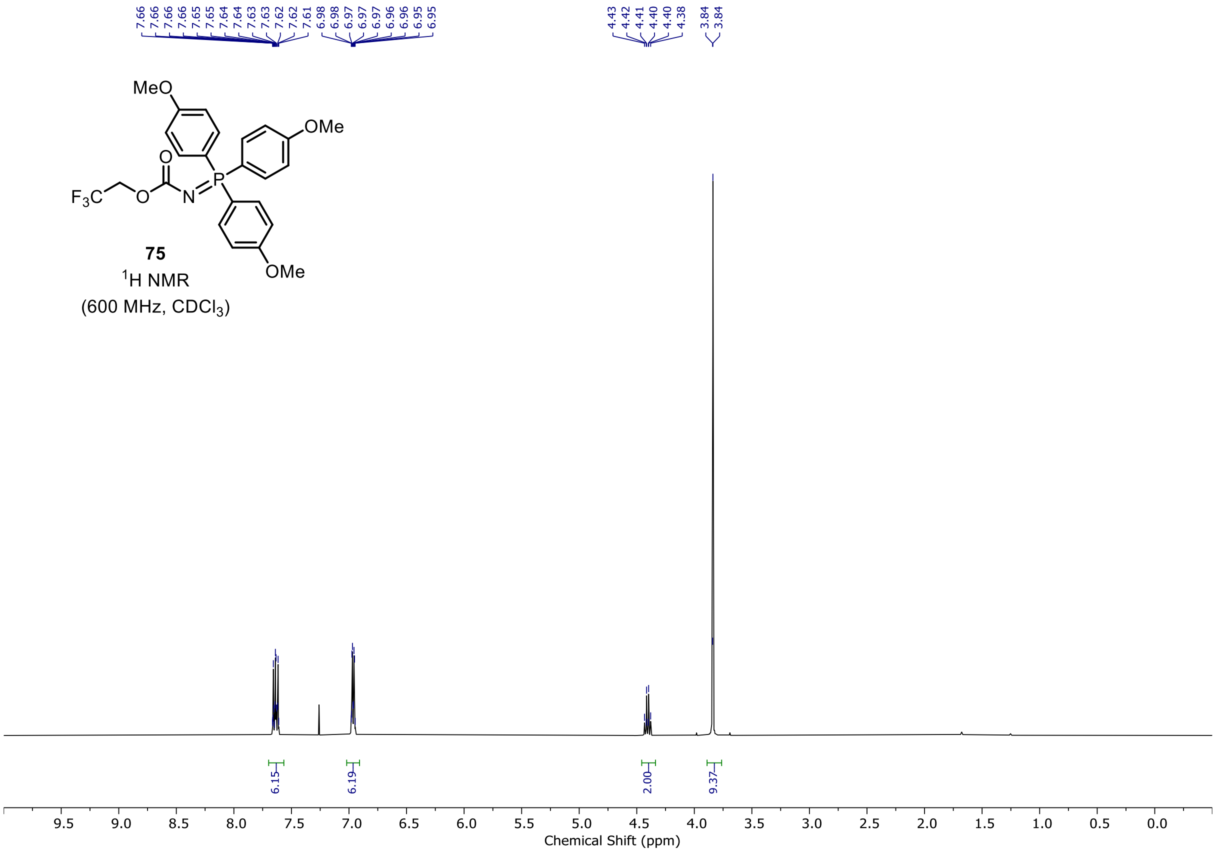

75

^{1}H NMR

(600 MHz, $CDCl_3$)

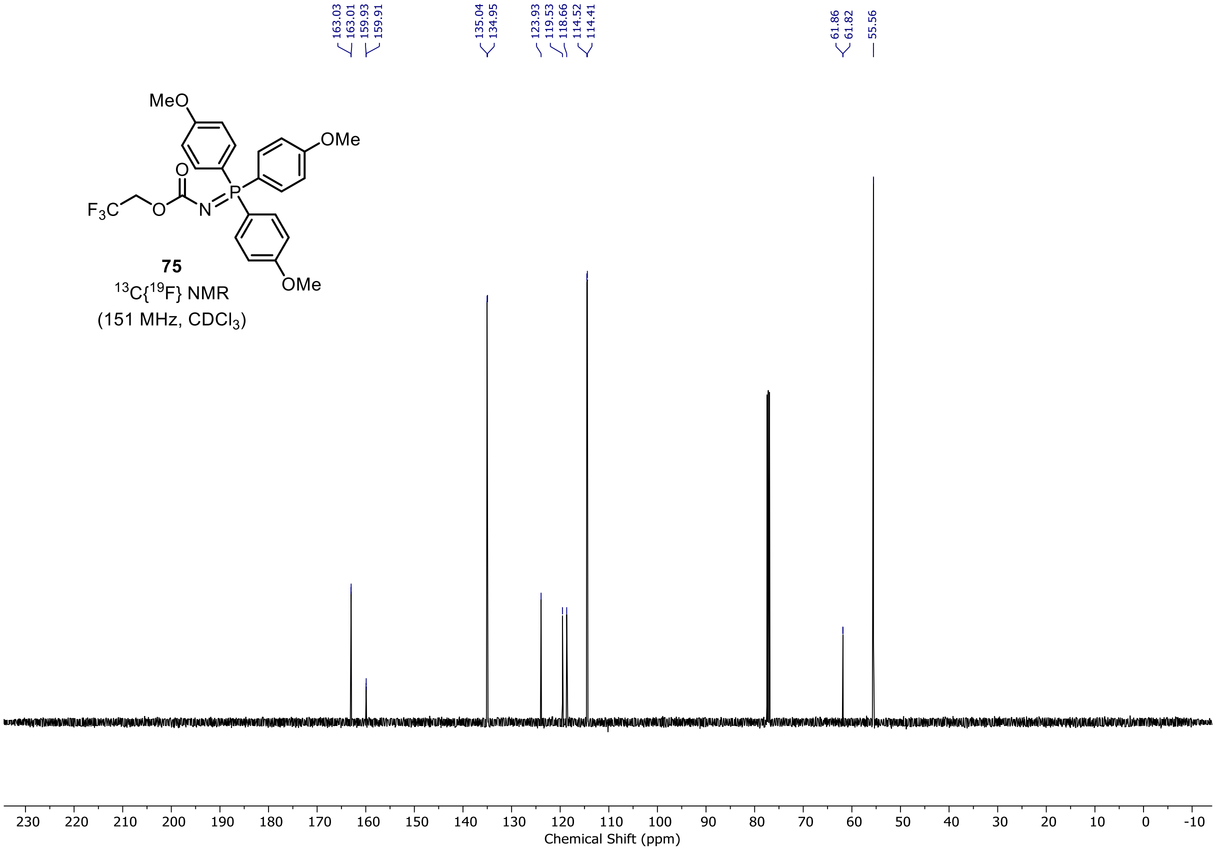

75

$^{13}C\{^{19}F\}$ NMR

(151 MHz, $CDCl_3$)

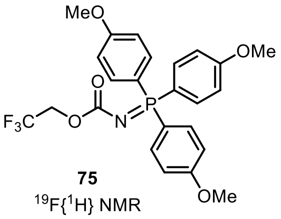

75
$^{19}F\{^{1}H\}$ NMR
(564 MHz, $CDCl_3$)

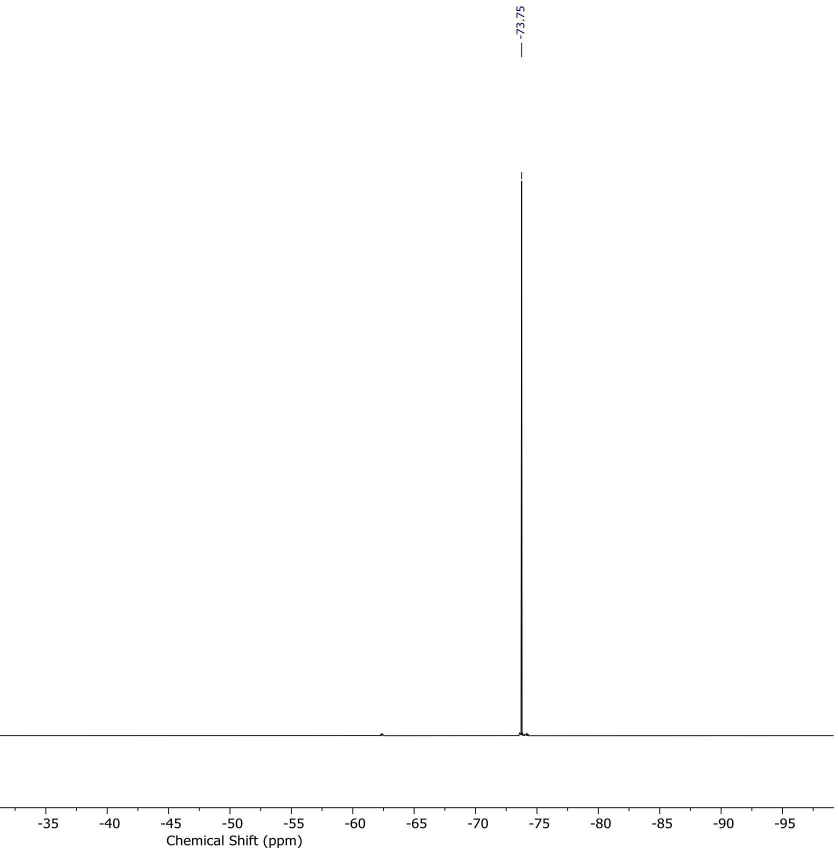

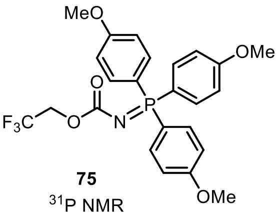

75
^{31}P NMR
(242 MHz, $CDCl_3$)

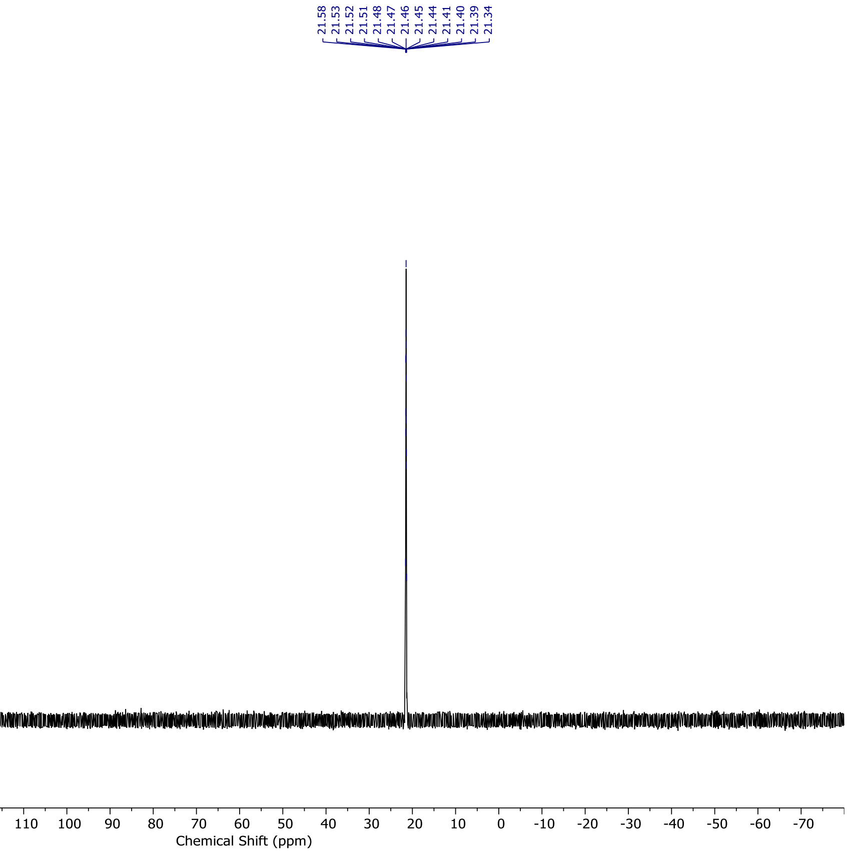

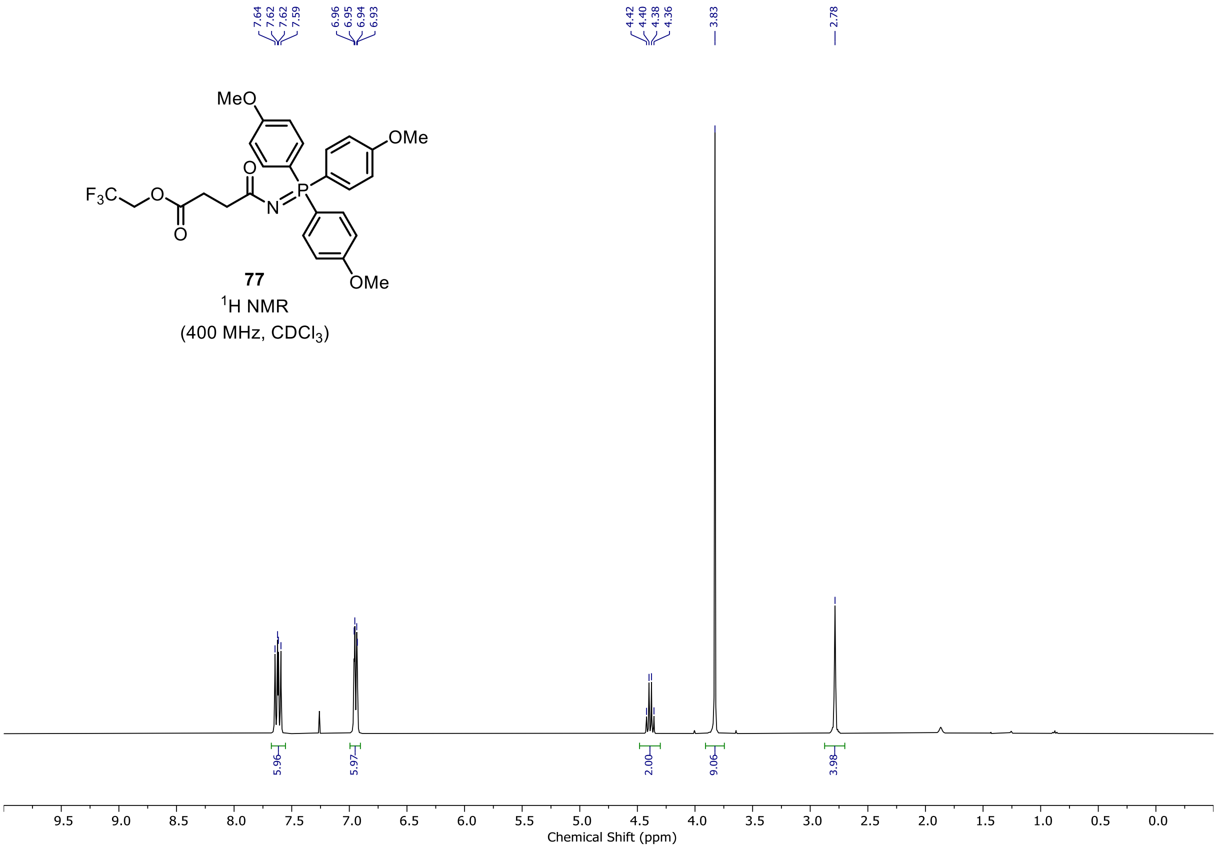

77
^{1}H NMR
(400 MHz, $CDCl_3$)

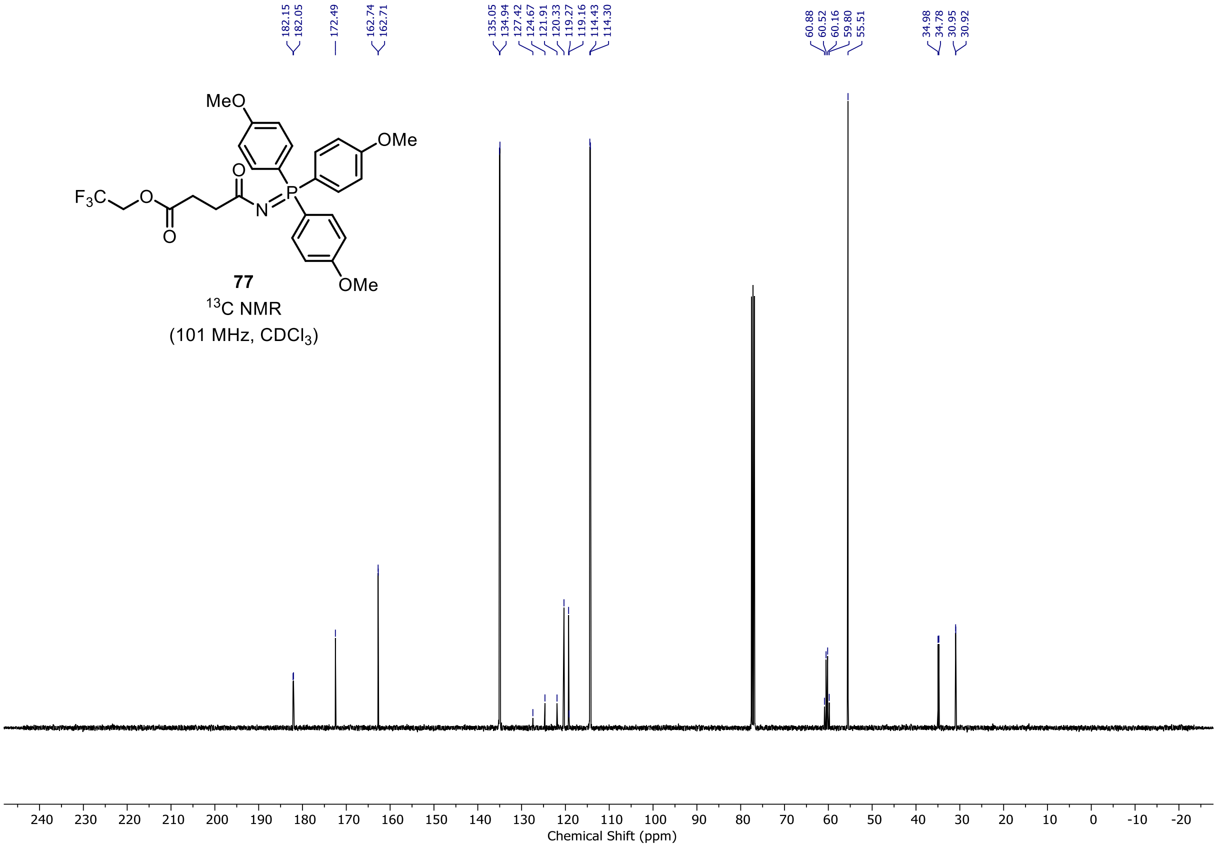

77
^{13}C NMR
(101 MHz, $CDCl_3$)

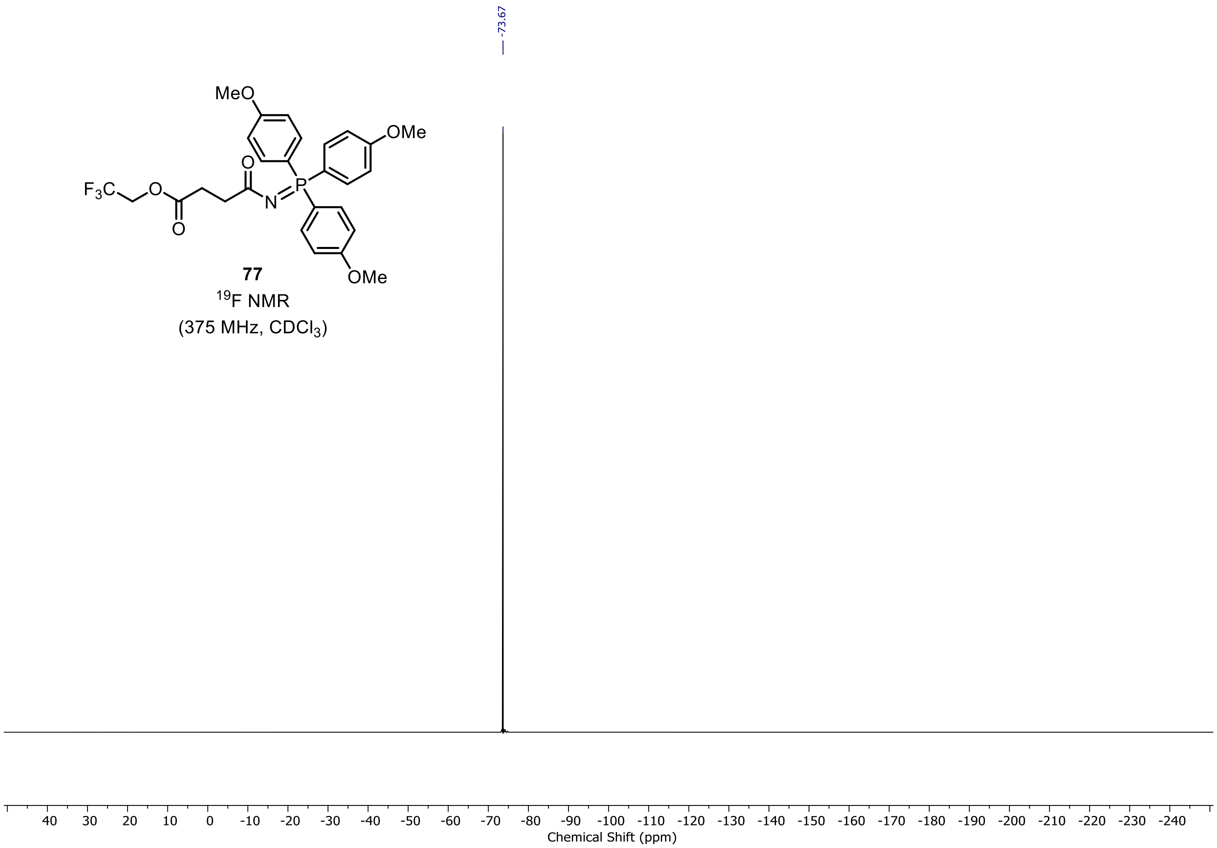
-73.67
MeO
OMe
OMe
F_3C
O
N
P
77
^{19}F NMR
(375 MHz, $CDCl_3$)
40 30 20 10 0 -10 -20 -30 -40 -50 -60 -70 -80 -90 -100 -110 -120 -130 -140 -150 -160 -170 -180 -190 -200 -210 -220 -230 -240
Chemical Shift (ppm)

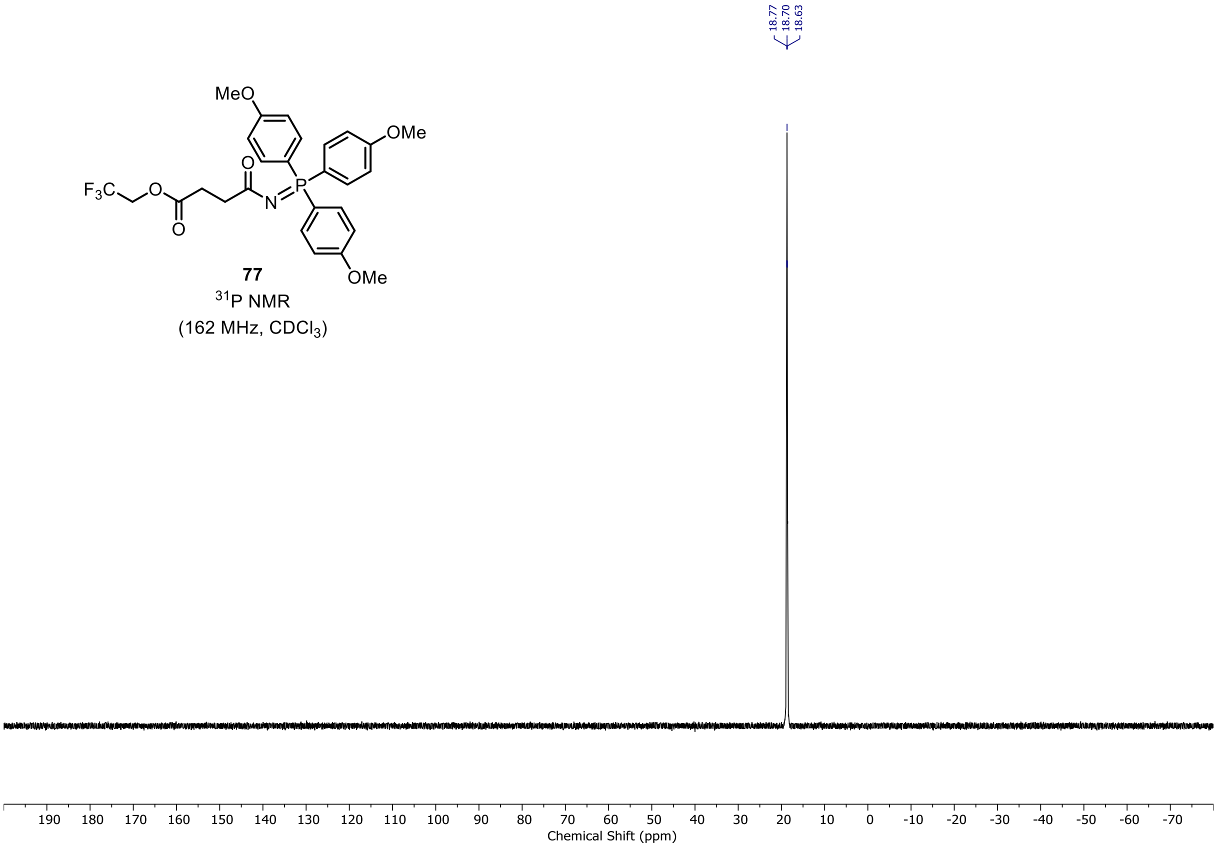
18.77
18.70
18.63
MeO
OMe
OMe
F_3C
O
N
P
77
^{31}P NMR
(162 MHz, $CDCl_3$)
190 180 170 160 150 140 130 120 110 100 90 80 70 60 50 40 30 20 10 0 -10 -20 -30 -40 -50 -60 -70
Chemical Shift (ppm)

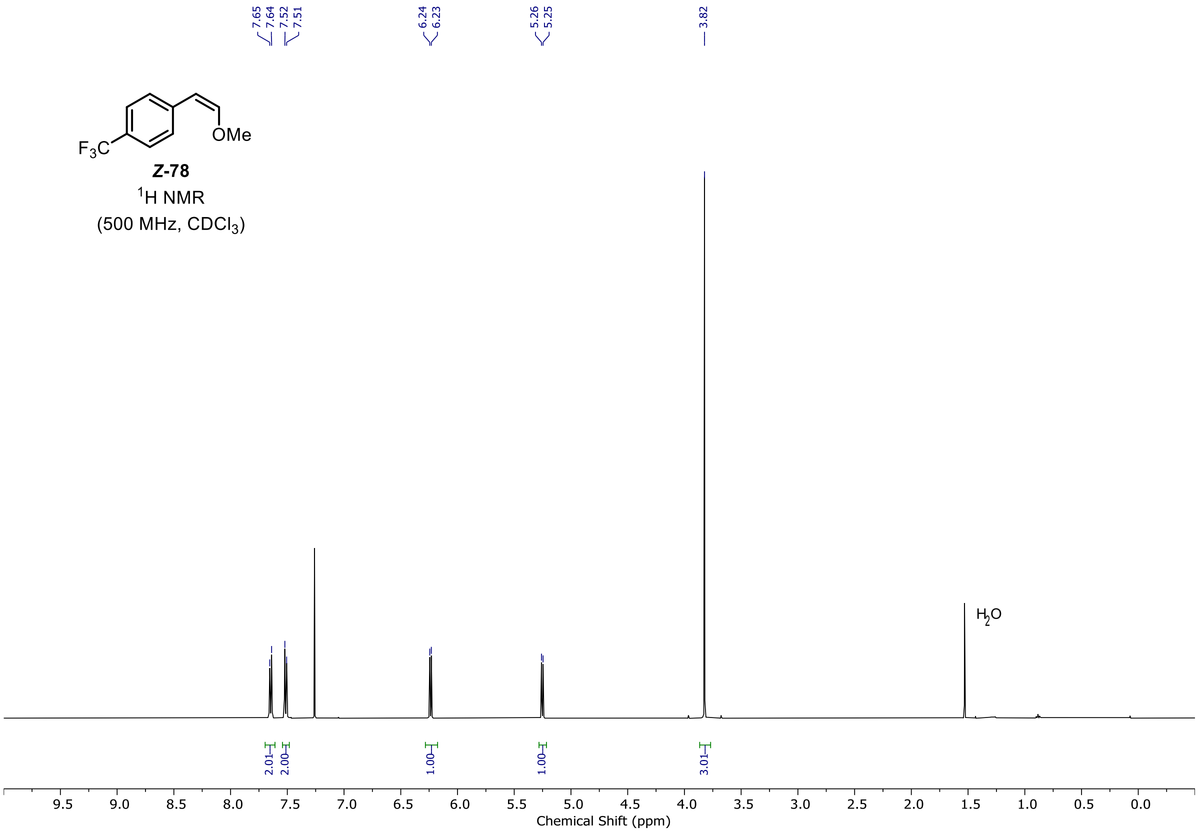
7.65
7.64
7.52
7.51
6.24
6.23
5.26
5.25
3.82
F3C
OMe
Z-78
1H NMR
(500 MHz, CDCl3)
H2O
2.01
2.00
1.00
1.00
3.01
9.5 9.0 8.5 8.0 7.5 7.0 6.5 6.0 5.5 5.0 4.5 4.0 3.5 3.0 2.5 2.0 1.5 1.0 0.5 0.0
Chemical Shift (ppm)

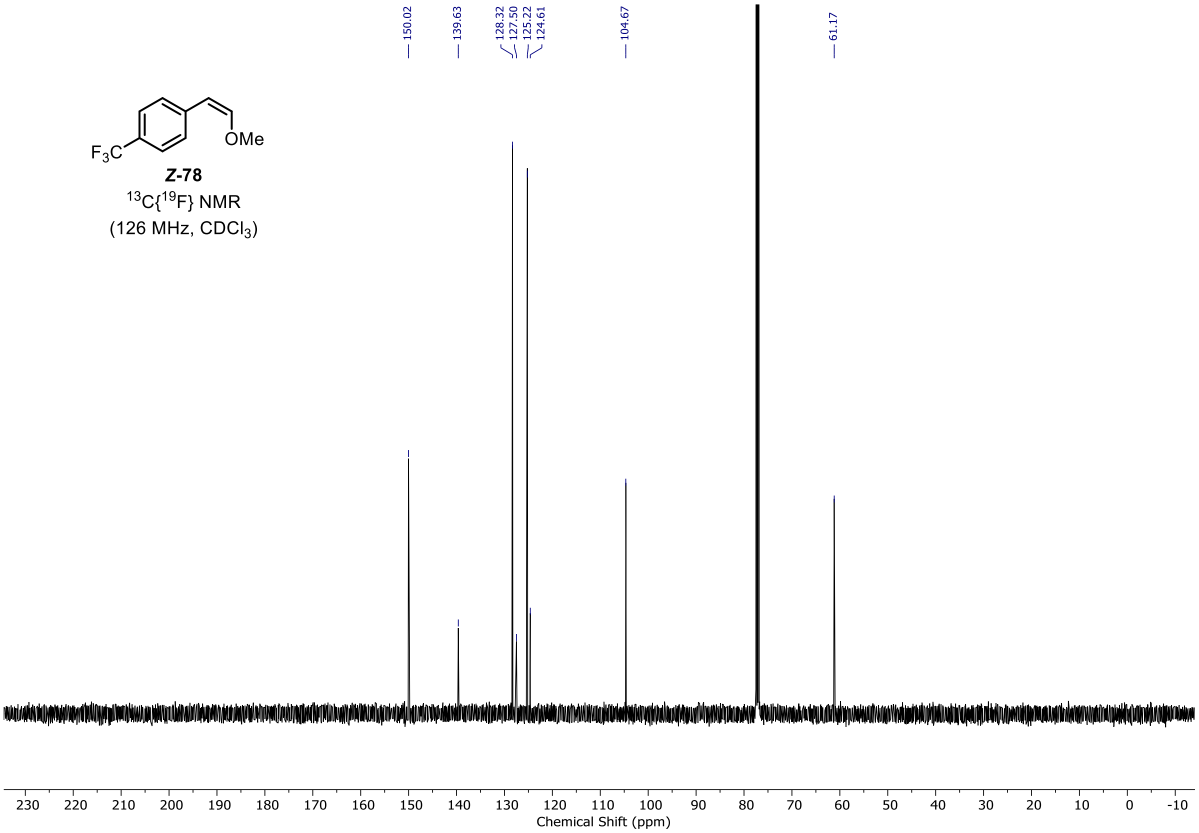
150.02
139.63
128.32
127.50
125.22
124.61
104.67
61.17
F3C
OMe
Z-78
13C{19F} NMR
(126 MHz, CDCl3)
230 220 210 200 190 180 170 160 150 140 130 120 110 100 90 80 70 60 50 40 30 20 10 0 -10
Chemical Shift (ppm)

***Z*-78**
$^{19}F\{^1H\}$ NMR
(470 MHz, $CDCl_3$)

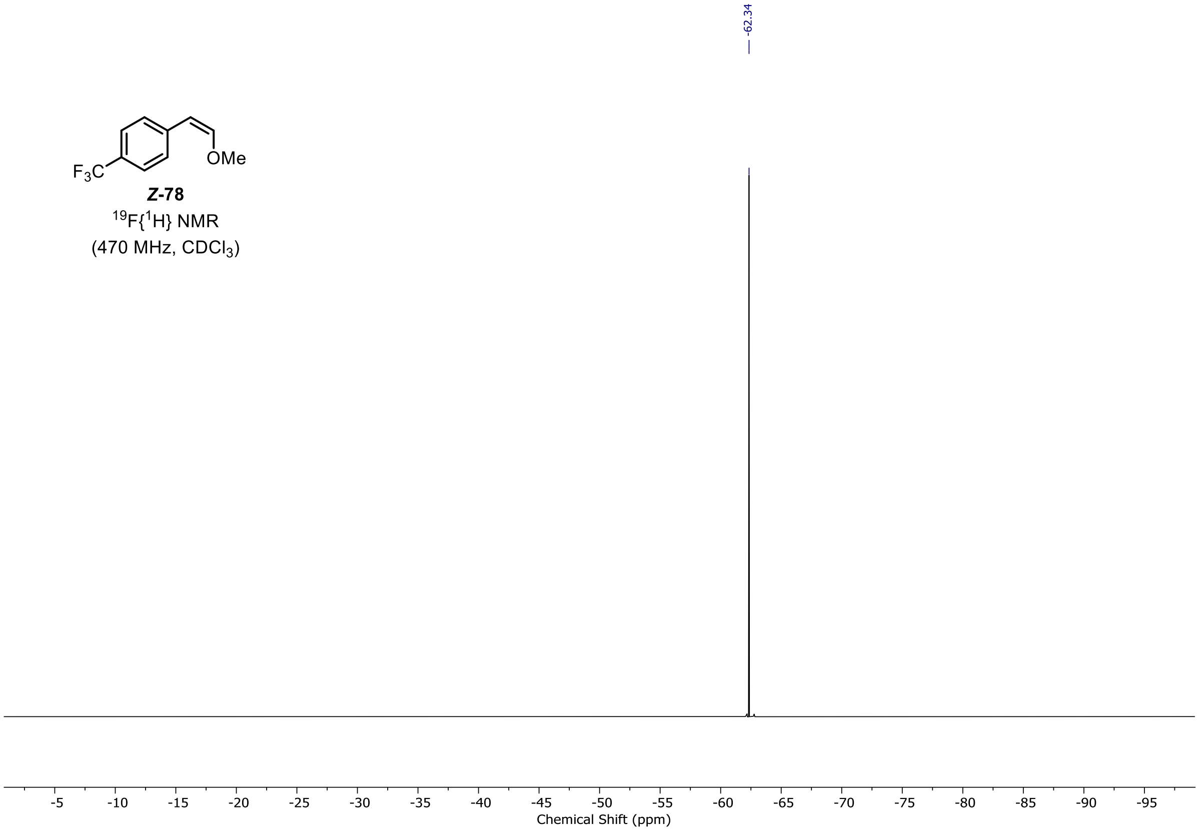

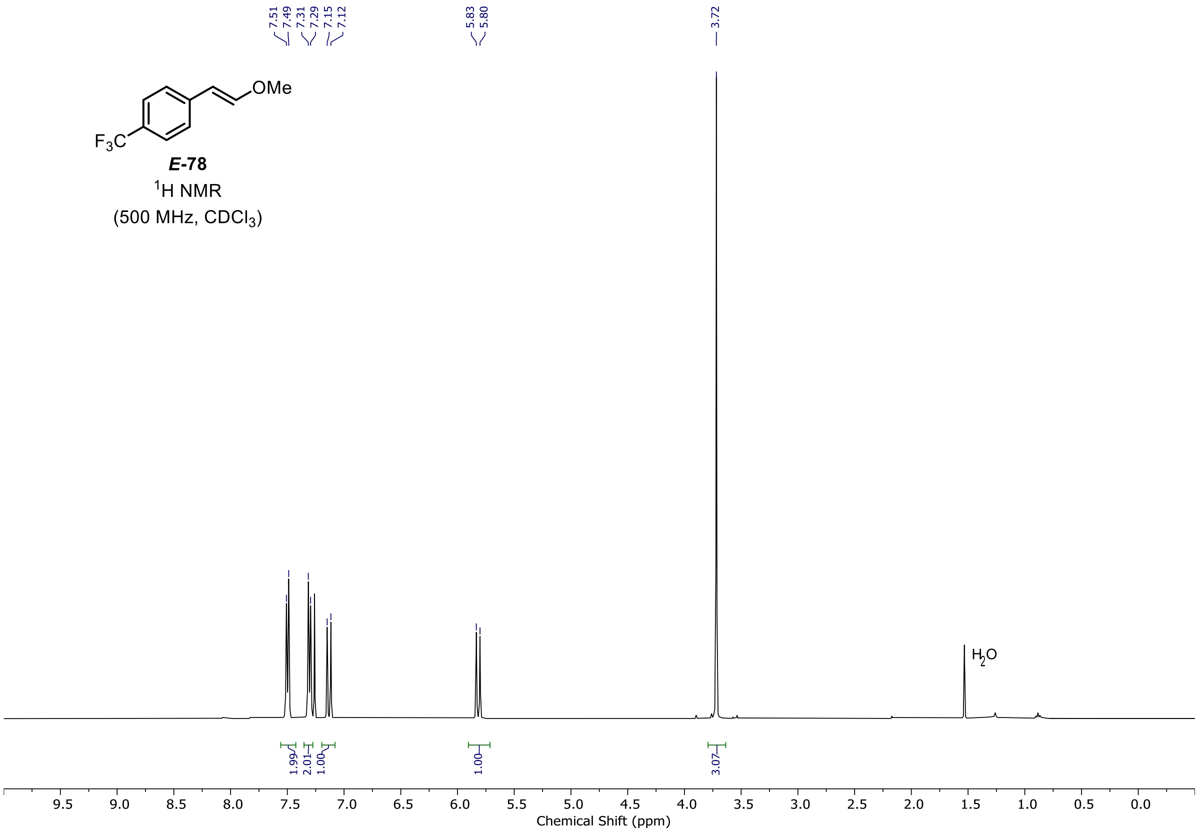

***E*-78**
^{1}H NMR
(500 MHz, $CDCl_3$)

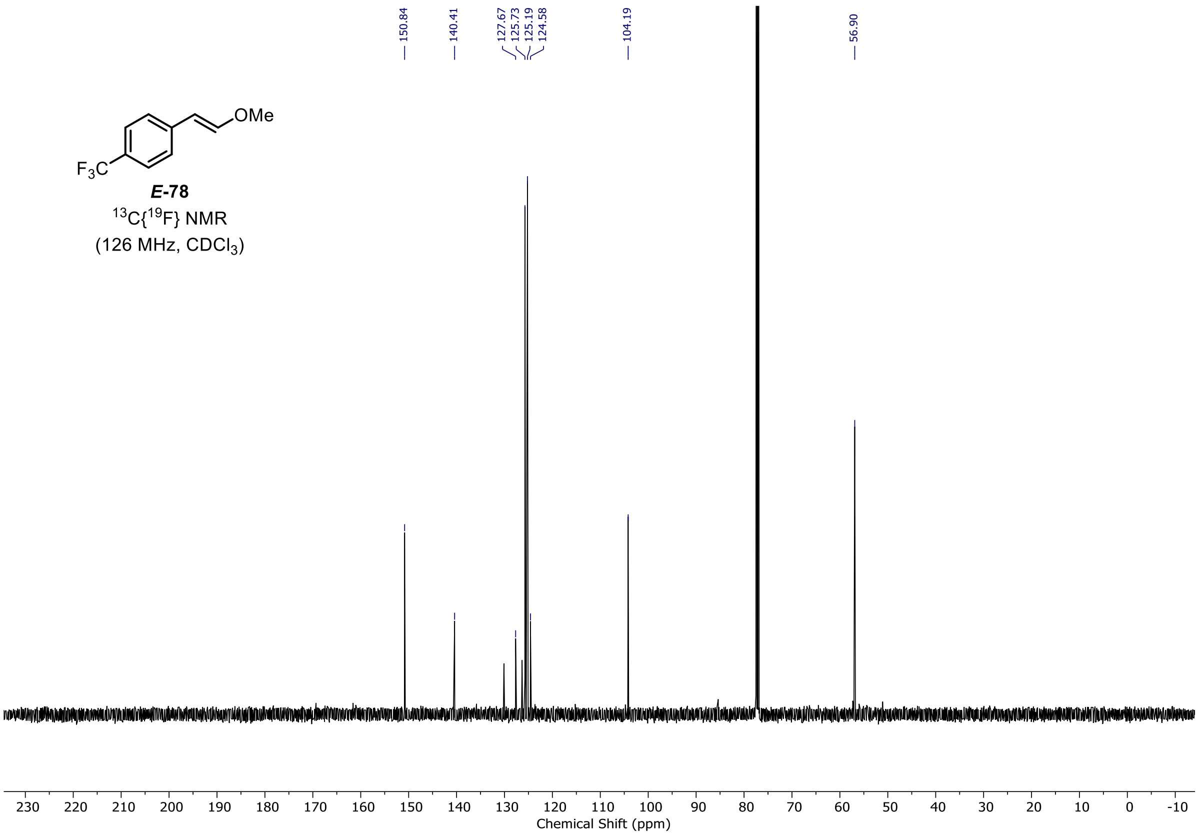

***E*-78**
$^{13}C\{^{19}F\}$ NMR
(126 MHz, $CDCl_3$)

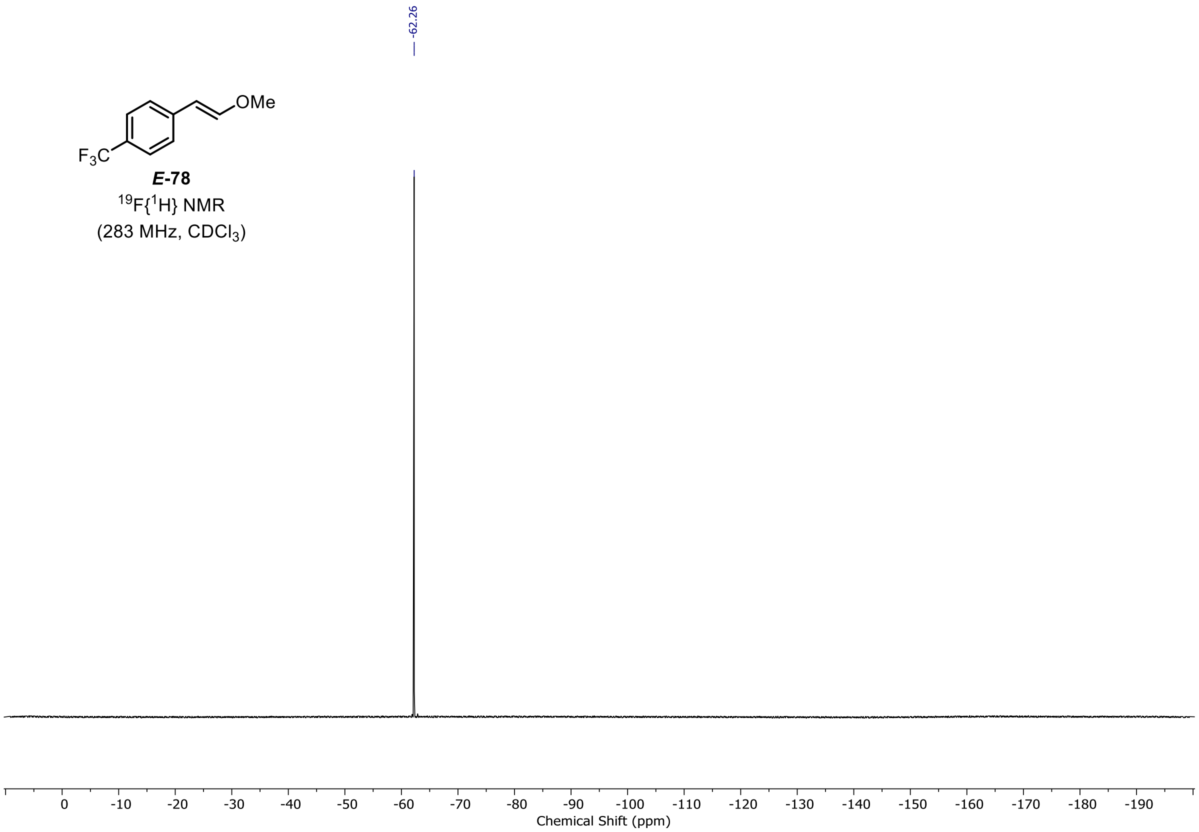

-62.26
OMe
F_3C
***E*-78**
$^{19}F\{^1H\}$ NMR
(283 MHz, $CDCl_3$)
Chemical Shift (ppm)

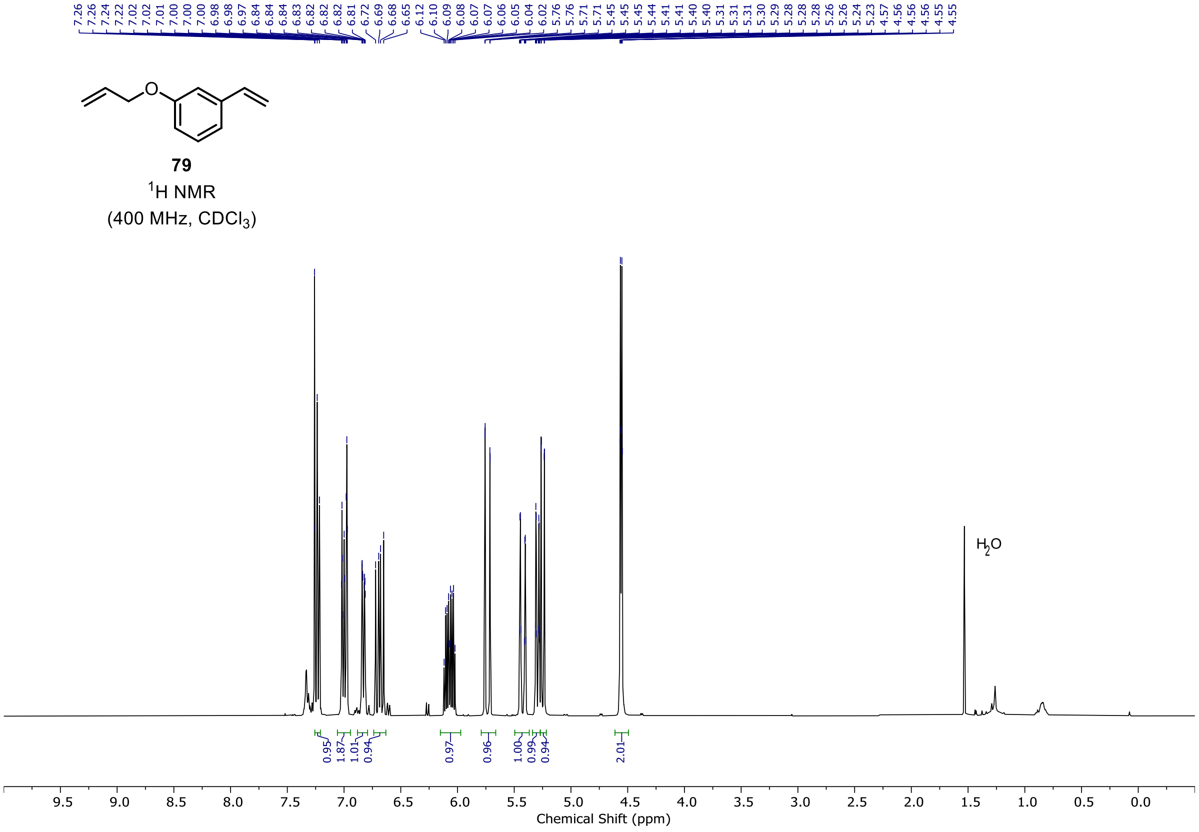

79
^1H NMR
(400 MHz, $CDCl_3$)
H_2O
Chemical Shift (ppm)

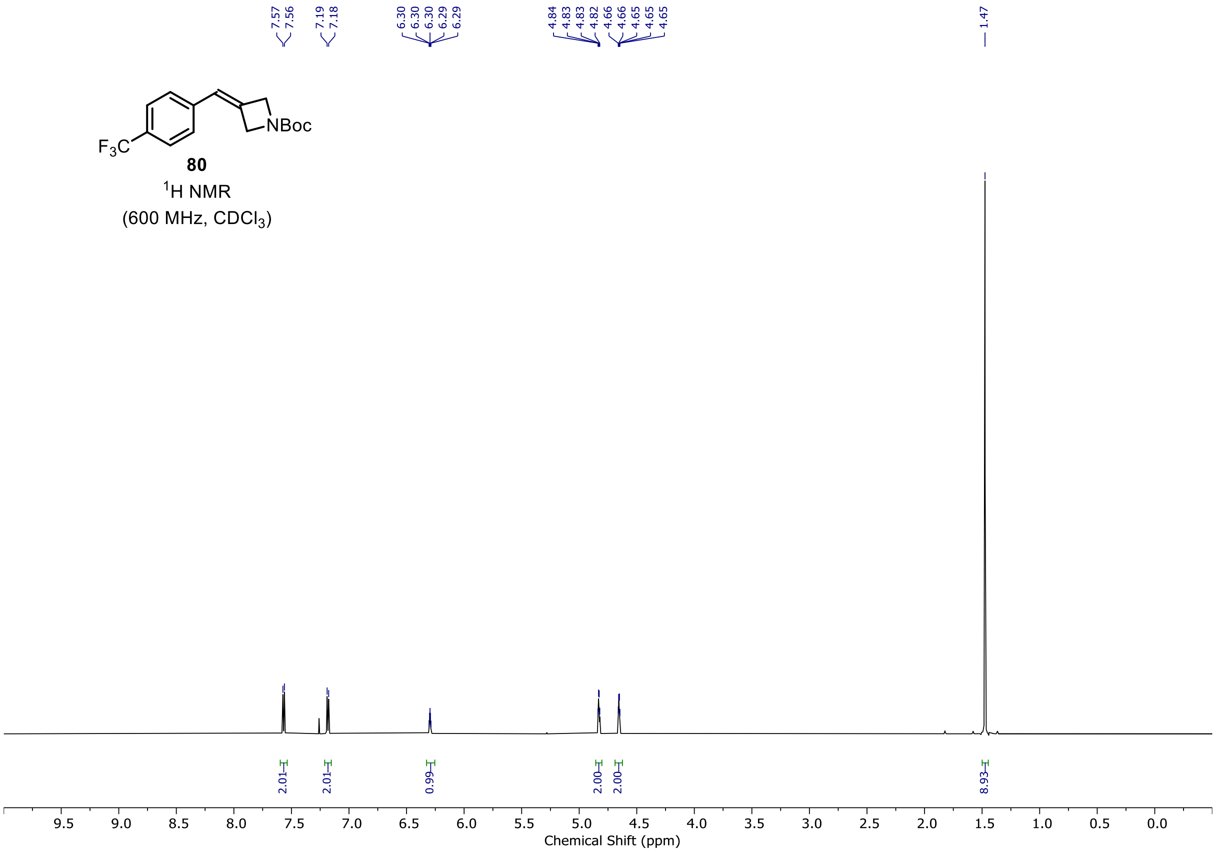

F3C
NBoc
80
1H NMR
(600 MHz, CDCl3)
7.57
7.56
7.19
7.18
6.30
6.30
6.30
6.29
6.29
4.84
4.83
4.83
4.82
4.66
4.66
4.65
4.65
4.65
1.47
2.01
2.01
0.99
2.00
2.00
8.93
9.5 9.0 8.5 8.0 7.5 7.0 6.5 6.0 5.5 5.0 4.5 4.0 3.5 3.0 2.5 2.0 1.5 1.0 0.5 0.0
Chemical Shift (ppm)

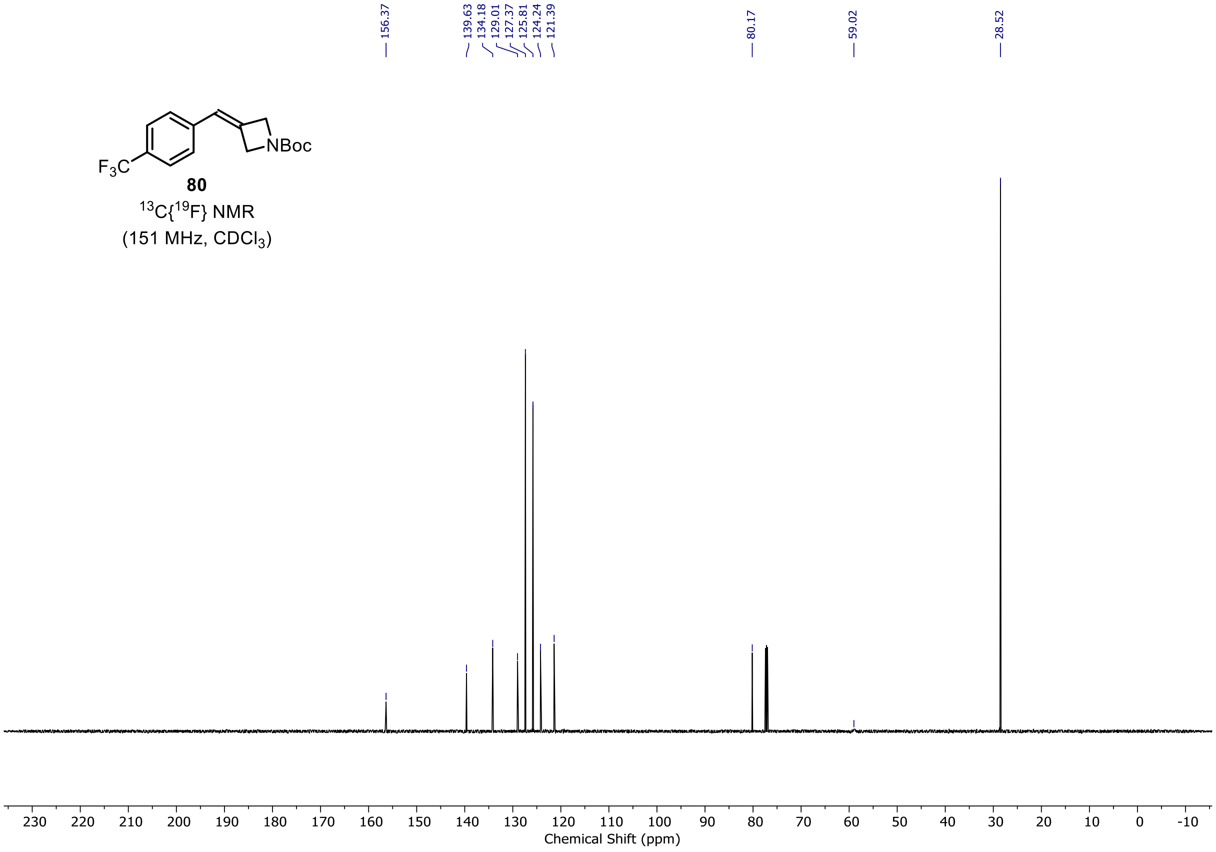

F3C
NBoc
80
13C{19F} NMR
(151 MHz, CDCl3)
156.37
139.63
134.18
129.01
127.37
125.81
124.24
121.39
80.17
59.02
28.52
230 220 210 200 190 180 170 160 150 140 130 120 110 100 90 80 70 60 50 40 30 20 10 0 -10
Chemical Shift (ppm)

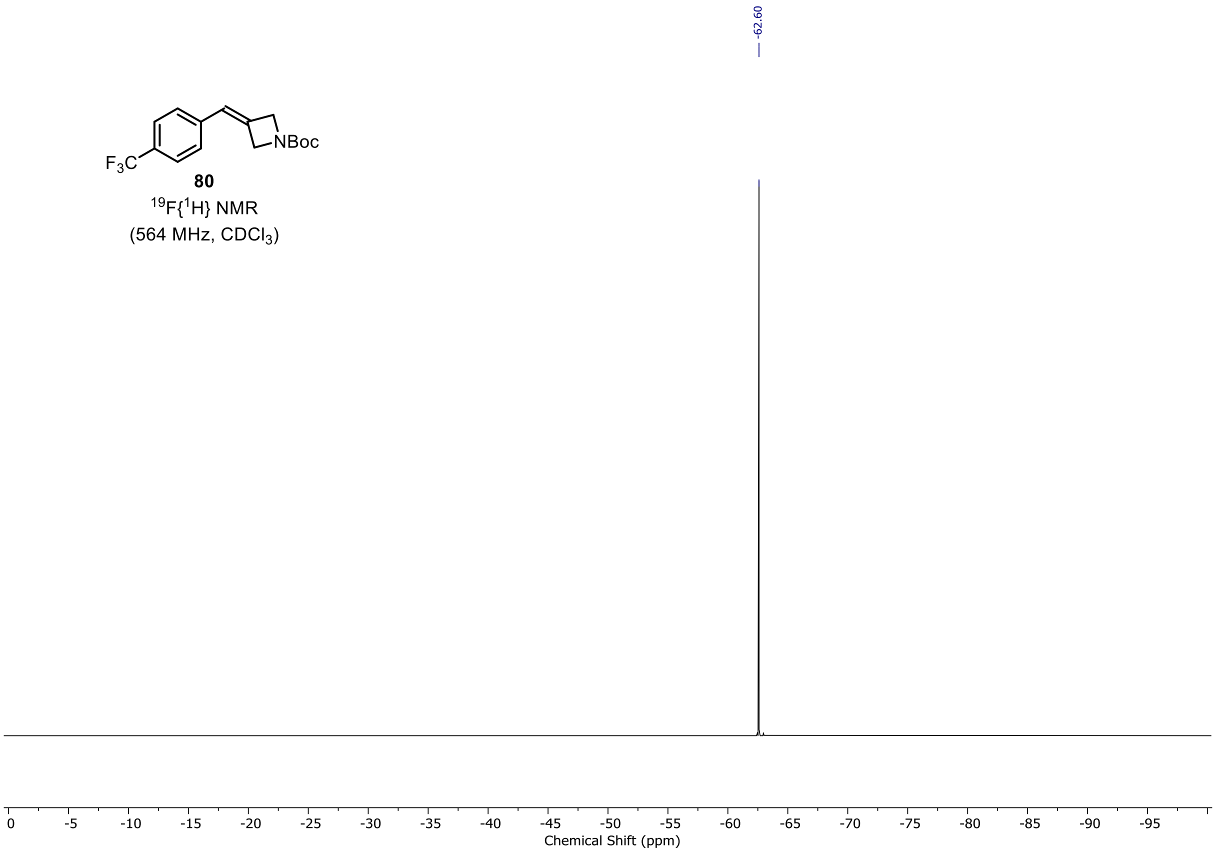
-62.60
F3C
NBoc
80
19F{1H} NMR
(564 MHz, CDCl3)
0 -5 -10 -15 -20 -25 -30 -35 -40 -45 -50 -55 -60 -65 -70 -75 -80 -85 -90 -95
Chemical Shift (ppm)

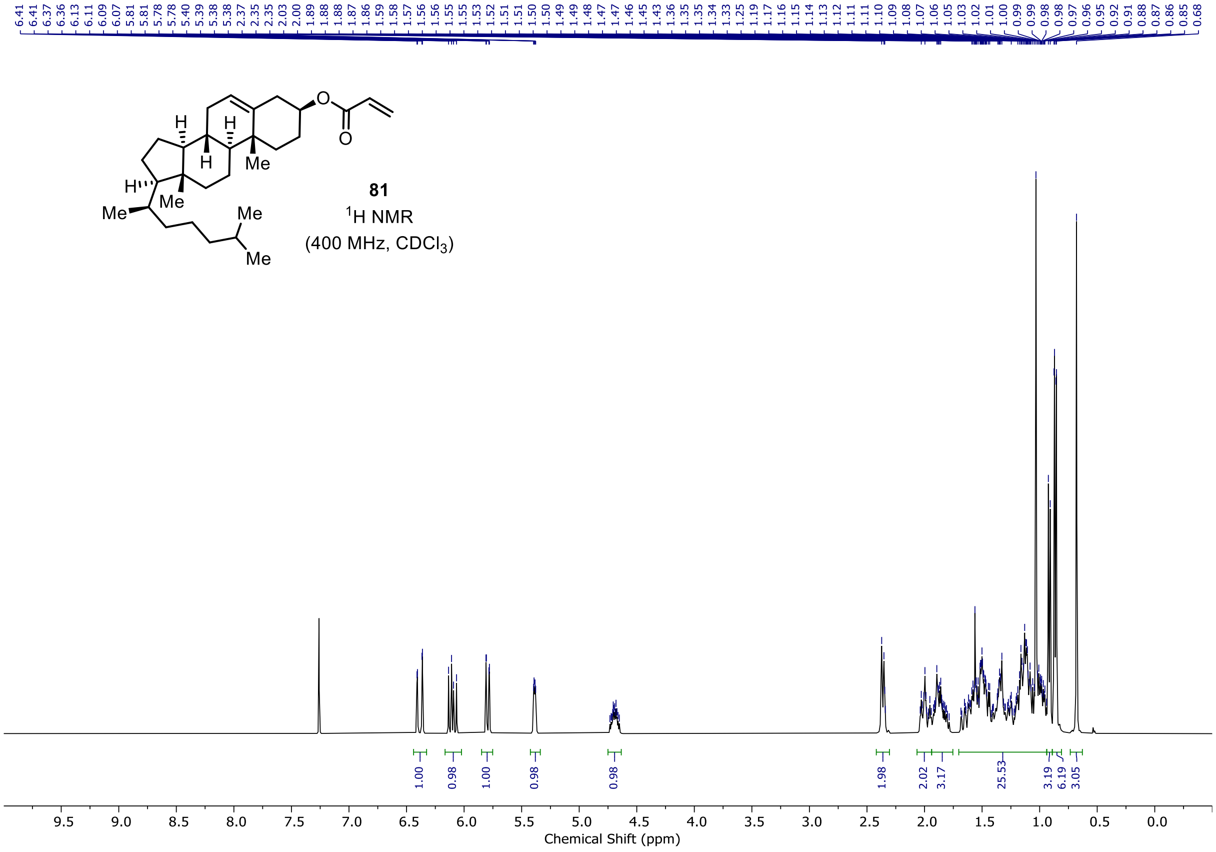
6.41 6.41 6.37 6.36 6.13 6.11 6.09 6.07 5.81 5.81 5.78 5.78 5.40 5.39 5.38 5.38 2.37 2.35 2.35 2.03 2.00 1.89 1.88 1.88 1.87 1.86 1.59 1.58 1.57 1.56 1.56 1.55 1.55 1.53 1.52 1.51 1.51 1.50 1.50 1.49 1.49 1.48 1.47 1.47 1.46 1.45 1.43 1.36 1.35 1.35 1.34 1.33 1.25 1.19 1.17 1.17 1.16 1.15 1.14 1.13 1.12 1.11 1.11 1.10 1.09 1.08 1.07 1.06 1.05 1.03 1.02 1.01 1.00 0.99 0.99 0.98 0.98 0.98 0.97 0.96 0.95 0.92 0.91 0.88 0.87 0.86 0.85 0.68
H
H
H
Me
O
O
H
Me
Me
Me
Me
81
1H NMR
(400 MHz, CDCl3)
1.00 0.98 1.00 0.98 0.98 1.98 2.02 3.17 25.53 3.19 6.19 3.05
9.5 9.0 8.5 8.0 7.5 7.0 6.5 6.0 5.5 5.0 4.5 4.0 3.5 3.0 2.5 2.0 1.5 1.0 0.5 0.0
Chemical Shift (ppm)

References

[1] X.-W. Lan, N.-X. Wang, Y. Xing, "Recent Advances in Radical Difunctionalization of Simple Alkenes" *Eur. J. Org. Chem.* **2017**, *2017*, 5821–5851.

[2] H. Yao, W. Hu, W. Zhang, "Difunctionalization of Alkenes and Alkynes via Intermolecular Radical and Nucleophilic Additions" *Molecules* **2021**, *26*, 105.

[3] B. T. Matsuo, P. H. R. Oliveira, E. F. Pissinati, K. B. Vega, I. S. de Jesus, J. T. M. Correia, M. Paixao, "Photoinduced carbamoylation reactions: unlocking new reactivities towards amide synthesis" *Chem. Commun.* **2022**, *58*, 8322–8339.

[4] M. S. Faculak, A. M. Veatch, E. J. Alexanian, "Cobalt-catalyzed synthesis of amides from alkenes and amines promoted by light" *Science* **2024**, *383*, 77–81.

[5] L. Friedman, H. Shechter, "Amidation and aminoalkylation of olefins: Blocking techniques in radical chain reactions" *Tetrahedron Lett.* **1961**, *2*, 238–242.

[6] D. Elad, J. Rokach, "The Light-Induced Amidation of Terminal Olefins1" *J. Org. Chem.* **1964**, *29*, 1855–1859.

[7] E. de P. Beato, D. Mazzarella, M. Balletti, P. Melchiorre, "Photochemical generation of acyl and carbamoyl radicals using a nucleophilic organic catalyst: applications and mechanism thereof" *Chem. Sci.* **2020**, *11*, 6312–6324.

[8] I. Martin Ogbu, G. Kurtay, F. Robert, Y. Landais, "Oxamic acids: useful precursors of carbamoyl radicals" *Chem. Commun.* **2022**, *58*, 7593–7607.

[9] N. Alandini, L. Buzzetti, G. Favi, T. Schulte, L. Candish, K. D. Collins, P. Melchiorre, "Amide Synthesis by Nickel/Photoredox-Catalyzed Direct Carbamoylation of (Hetero)Aryl Bromides" *Angew. Chem. Int. Ed.* **2020**, *59*, 5248–5253.

[10] K. Pradhan, S. Mandal, R. Jana, "A Visible-Light Organophotoredox-Catalyzed anti-Markovnikov Hydrocarbamoylation of Unactivated Alkene and Cyclization Cascade via Radical Relay" *Org. Lett.* **2025**, *27*, 9687–9692.

[11] W. F. Petersen, R. J. K. Taylor, J. R. Donald, "Photoredox-Catalyzed Reductive Carbamoyl Radical Generation: A Redox-Neutral Intermolecular Addition–Cyclization Approach to Functionalized 3,4-Dihydroquinolin-2-ones" *Org. Lett.* **2017**, *19*, 874–877.

[12] Y. Zhang, K. B. Teuscher, H. Ji, "Direct α-heteroarylation of amides (α to nitrogen) and ethers through a benzaldehyde-mediated photoredox reaction" *Chem. Sci.* **2016**, *7*, 2111–2118.

[13] T. Caronna, C. Gambarotti, L. Palmisano, C. Punta, F. Recupero, "Sunlight induced functionalisation of some heterocyclic bases in the presence of polycrystalline TiO2" *Chem. Commun.* **2003**, 2350–2351.

[14] S. Delanghe, J. R. Delanghe, R. Speeckaert, W. Van Biesen, M. M. Speeckaert, "Mechanisms and consequences of carbamoylation" *Nat. Rev. Nephrol.* **2017**, *13*, 580–593.

[15] S. Hu, A. T. Radosevich, "Electrophilic C(sp2)−H Cyanation with Inorganic Cyanate (OCN−) by PIII/PV=O-Catalyzed Phase Transfer Activation" *Angew. Chem. Int. Ed.* **2024**, *63*, e202409854.

[16] J. A. Rossi-Ashton, A. K. Clarke, W. P. Unsworth, R. J. K. Taylor, "Phosphoranyl Radical Fragmentation Reactions Driven by Photoredox Catalysis" *ACS Catal.* **2020**, *10*, 7250–7261.

[17] A. J. Chinn, K. Sedillo, A. G. Doyle, "Phosphine/Photoredox Catalyzed Anti-Markovnikov Hydroamination of Olefins with Primary Sulfonamides via α-Scission from Phosphoranyl Radicals" *J. Am. Chem. Soc.* **2021**, *143*, 18331–18338.

[18] K. Sedillo, F. Fan, R. R. Knowles, A. G. Doyle, “Cooperative Phosphine-Photoredox Catalysis Enables N–H Activation of Azoles for Intermolecular Olefin Hydroamination” *J. Am. Chem. Soc.* **2024**, *146*, 20349–20356.
[19] D. Pan, G. Nie, S. Jiang, T. Li, Z. Jin, “Radical reactions promoted by trivalent tertiary phosphines” *Org. Chem. Front.* **2020**, *7*, 2349–2371.
[20] J. Feng, L. Nicchio, L. Liu, Y. Wu, K. Lu, S. Protti, X. Zhao, “Recent advances in visible light-driven phosphine-mediated transformations” *Org. Chem. Front.* **2025**, *12*, 1695–1707.
[21] G. Pandey, D. Pooranchand, U. T. Bhalerao, “Photoinduced single electron transfer activation of organophosphines: Nucleophilic trapping of phosphine radical cation” *Tetrahedron* **1991**, *47*, 1745–1752.
[22] J. Zhang, C. Mück-Lichtenfeld, A. Studer, “Photocatalytic phosphine-mediated water activation for radical hydrogenation” *Nature* **2023**, *619*, 506–513.
[23] Z.-Z. Xie, Y. Zheng, Z.-H. Liao, C.-P. Yuan, M.-Z. Li, K.-Y. Deng, H.-Y. Xiang, K. Chen, H. Yang, “Photoredox-catalyzed hydrogenation of alkenes assisted by an in situ generated PPh3(OH) radical and acetic acid” *Org. Chem. Front.* **2024**, *11*, 4187–4193.
[24] J. A. Baban, B. P. Roberts, “Ligand-σ phosphoranyl radicals” *J. Chem. Soc. Chem. Commun.* **1979**, *0*, 537–539.
[25] R. Kumar, R. K. Arigela, B. Kundu, “Unprecedented Transformation of a Directing Group Generated In Situ and Its Application in the One-Pot Synthesis of 2-Alkenyl Benzonitriles” *Chem. – Eur. J.* **2015**, *21*, 11807–11812.
[26] R. K. Arigela, R. Kumar, T. Joshi, R. Mahar, B. Kundu, “Ruthenium(II)-catalyzed C–H activation/C–N bond formation via in situ generated iminophosphorane as the directing group: construction of annulated pyridin-2(1H)-ones” *RSC Adv.* **2014**, *4*, 57749–57753.
[27] R. Bielsa, A. Larrea, R. Navarro, T. Soler, E. P. Urriolabeitia, “Synthesis, Structure, Reactivity, and Catalytic Activity of C,N- and C,N,N-Orthopalladated Iminophosphoranes” *Eur. J. Inorg. Chem.* **2005**, *2005*, 1724–1736.
[28] M. Formica, D. Rozsar, G. Su, A. J. M. Farley, D. J. Dixon, “Bifunctional Iminophosphorane Superbase Catalysis: Applications in Organic Synthesis” *Acc. Chem. Res.* **2020**, *53*, 2235–2247.
[29] J. Park, A. U. Krishnapriya, Y. Park, M. Kim, T. W. Reidl, R. Kuniyil, J. Son, “Copper(I)-Catalyzed Decarboxylative N-Phosphorylation: Modular Preparation of N-Acyl Iminophosphoranes Using Dioxazolones and Phosphines” *Adv. Synth. Catal.* **2023**, *365*, 4495–4501.
[30] A. R. Katritzky, N. M. Khashab, S. Bobrov, M. Yoshioka, “Synthesis of Mono- and Symmetrical Di-N-hydroxy- and N-Aminoguanidines” *J. Org. Chem.* **2006**, *71*, 6753–6758.
[31] H. Staudinger, J. Meyer, “Über neue organische Phosphorverbindungen III. Phosphinmethylenderivate und Phosphinimine” *Helv. Chim. Acta* **1919**, *2*, 635–646.
[32] S. Bittner, Y. Assaf, P. Krief, M. Pomerantz, B. T. Ziemnicka, C. G. Smith, “Synthesis of N-acyl-, N-sulfonyl-, and N-phosphinylphospha(PV)azenes by a redox-condensation reaction using amides, triphenylphosphine, and diethyl azodicarboxylate” *J. Org. Chem.* **1985**, *50*, 1712–1718.
[33] H. Morita, A. Tatami, T. Maeda, B. Ju Kim, W. Kawashima, T. Yoshimura, H. Abe, T. Akasaka, “Generation of Nitrene by the Photolysis of N-Substituted Iminodibenzothiophene” *J. Org. Chem.* **2008**, *73*, 7159–7163.

[34] J.-J. Tang, X. Yu, Y. Wang, Y. Yamamoto, M. Bao, "Interweaving Visible-Light and Iron Catalysis for Nitrene Formation and Transformation with Dioxazolones" *Angew. Chem. Int. Ed.* **2021**, *60*, 16426–16435.
[35] S. Lin, B. Lin, Z. Zhang, J. Chen, Y. Luo, Y. Xia, "Construction of N-Acyliminophosphoranes via Iron(II)-Catalyzed Imidization of Phosphines with N-Acyloxyamides" *Org. Lett.* **2022**, *24*, 3302–3306.
[36] W. Han, J. Su, J.-N. Mo, J. Zhao, "Photoredox Catalytic Phosphine-Mediated Deoxygenation of Hydroxylamines Enables the Construction of N-Acyliminophosphoranes" *Org. Lett.* **2022**, *24*, 6247–6251.
[37] W. Wang, S. Cao, Y. Wang, B. Hu, X. Zhu, Y. Sun, Y. Wang, "Electrochemically-Enabled Construction of N-Acyl Iminophosphoranes" *Eur. J. Org. Chem.* **2024**, *27*, e202400440.
[38] S. N. Alektiar, Z. K. Wickens, "Photoinduced Hydrocarboxylation via Thiol-Catalyzed Delivery of Formate Across Activated Alkenes" *J. Am. Chem. Soc.* **2021**, *143*, 13022–13028.
[39] F. Palacios, C. Alonso, D. Aparicio, G. Rubiales, J. M. de los Santos, "The aza-Wittig reaction: an efficient tool for the construction of carbon–nitrogen double bonds" *Tetrahedron* **2007**, *63*, 523–575.
[40] G. R. Newkome, D. C. Hager, "A new contractive coupling procedure. Convenient phosphorus expulsion reaction" *J. Am. Chem. Soc.* **1978**, *100*, 5567–5568.
[41] Y. Uchida, K. Onoue, N. Tada, F. Nagao, S. Oae, "Ligand coupling reaction on the phosphorus atom" *Tetrahedron Lett.* **1989**, *30*, 567–570.
[42] Y. Uchida, H. Kozawa, "Formation of 2,2′-bipyridyl by ligand coupling on the phosphorus atom" *Tetrahedron Lett.* **1989**, *30*, 6365–6368.
[43] K. D. Reichl, A. T. Radosevich, "A phosphine-mediated stereocontrolled synthesis of Z-enediynes by a vicinal dialkynylation of ethynylphosphonium salts" *Chem. Commun.* **2014**, *50*, 9302–9305.
[44] M. C. Hilton, X. Zhang, B. T. Boyle, J. V. Alegre-Requena, R. S. Paton, A. McNally, "Heterobiaryl synthesis by contractive C–C coupling via P(V) intermediates" *Science* **2018**, *362*, 799–804.
[45] L. Caiger, C. Sinton, T. Constantin, J. J. Douglas, N. S. Sheikh, F. Juliá, D. Leonori, "Radical hydroxymethylation of alkyl iodides using formaldehyde as a C1 synthon" *Chem. Sci.* **2021**, *12*, 10448–10454.
[46] Y.-Q. Guo, R. Wang, H. Song, Y. Liu, Q. Wang, "Visible-Light-Induced Deoxygenation/Defluorination Protocol for Synthesis of γ,γ-Difluoroallylic Ketones" *Org. Lett.* **2020**, *22*, 709–713.
[47] M. A. Short, M. F. Shehata, M. A. Sanders, J. L. Roizen, "Sulfamides direct radical-mediated chlorination of aliphatic C–H bonds" *Chem. Sci.* **2019**, *11*, 217–223.
[48] A. Alkayal, V. Tabas, S. Montanaro, I. A. Wright, A. V. Malkov, B. R. Buckley, "Harnessing Applied Potential: Selective β-Hydrocarboxylation of Substituted Olefins" *J. Am. Chem. Soc.* **2020**, *142*, 1780–1785.
[49] M. Amatore, C. Gosmini, J. Périchon, "CoBr2(Bpy): An Efficient Catalyst for the Direct Conjugate Addition of Aryl Halides or Triflates onto Activated Olefins" *J. Org. Chem.* **2006**, *71*, 6130–6134.
[50] J. Cuthbertson, J. D. Wilden, "*Z*-selective, *anti*-Markovnikov addition of alkoxides to terminal alkynes: an electron transfer pathway?" *Tetrahedron* **2015**, *71*, 4385–4392.

[51] C. E. Paul, A. Rajagopalan, I. Lavandera, V. Gotor-Fernández, W. Kroutil, V. Gotor, "Expanding the regioselective enzymatic repertoire: oxidative mono-cleavage of dialkenes catalyzed by Trametes hirsuta" *Chem. Commun.* **2012**, *48*, 3303–3305.
[52] S. Choudhary, D. M. Cannas, M. Wheatley, I. Larrosa, "A manganese(I)tricarbonyl-catalyst for near room temperature alkene and alkyne hydroarylation" *Chem. Sci.* **2022**, *13*, 13225–13230.
[53] M. Hayashi, Y. Matsuura, Y. Watanabe, "Fluoride-mediated phosphination of alkenes and alkynes by silylphosphines" *Tetrahedron Lett.* **2004**, *45*, 9167–9169.
